# Photo-Stitching of Nitroalkanes via Cross-Denitrative Olefination

**DOI:** 10.1021/jacs.6c06460

**Published:** 2026-07-16

**Authors:** Joshua K. Mitchell, Shrey P. Desai, Joseph M. Bergen, Alexandria Reyes, Maxwell S. McLean, Poulami Ghosh, Ajay H. Bansode, Marvin Parasram

**Affiliations:** Department of Chemistry, 5894 New York University, New York, New York 10003, United States

## Abstract

Despite their centrality in molecular design, stereodefined tri- and tetrasubstituted alkenes remain challenging to access, as most olefinations favor lower substitution and fail under sterically congested settings. Herein, we report the photoinduced cross-denitrative olefination of nitroalkanes to afford tri- and tetrasubstituted alkenes. Visible light excitation of in situ–generated nitronates promotes a photo-stitching event via denitrative cross-coupling of two nitroalkanes. The transformation tolerates a variety of functionalized nitroalkanes, including carbonyl- and heterocycle-containing substrates, complements metathesis and carbonyl olefination, and scales efficiently. Mechanistic studies support a photoinitiated and photopropagated nucleophilic-radical substitution and radical-chain elimination sequence in which nitronates function as photoreductants to generate radical-anion intermediates that undergo a subsequent photodriven β-elimination. These findings establish a previously inaccessible light-driven elimination process for radical-mediated olefination from readily available building blocks.

## Graphical abstract

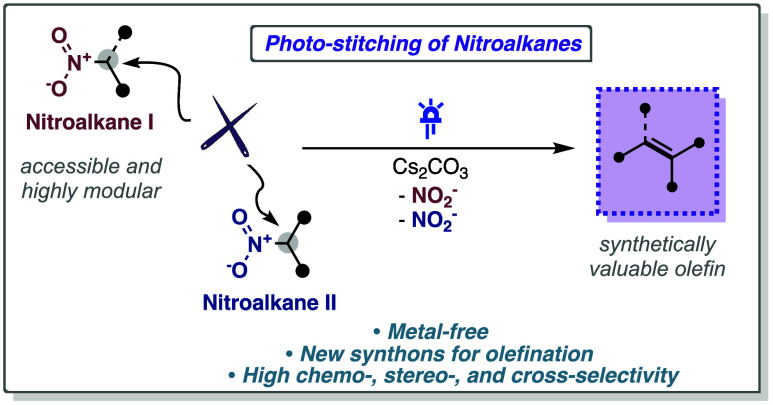

## Introduction

Alkenes are among the most widely used functional groups in organic synthesis,^1,2^ with broad applications in natural product assembly,^3,4^ pharmaceuticals,^5^ and materials science^6^ (Figure 1a, right). Their substitution pattern and geometry strongly influence conformation, reactivity, and noncovalent interactions.^7^ Notably, an analysis of approved drugs illustrates that trisubstituted alkenes are as prevalent as, or more common than, mono- and disubstituted systems,^8^ underscoring their value in tuning pharmacophore geometry, lipophilicity, and binding orientation (Figure 1a, left). Most established synthetic methods, however, overwhelmingly favor mono- and disubstituted alkenes,^9,10^ creating a disconnect between highly substituted alkene motifs in bioactive molecules and those readily accessible from standard methodologies (Figure 1a, left). Although tri- and tetrasubstituted alkenes offer exceptional steric and conformational control for chiral building blocks, rigid scaffolds, and diverse bioactive motifs,^11–13^ their stereoselective construction in acyclic settings remains difficult due to steric congestion and the resulting challenges in controlling *E*/*Z* geometry.

**Figure 1:**
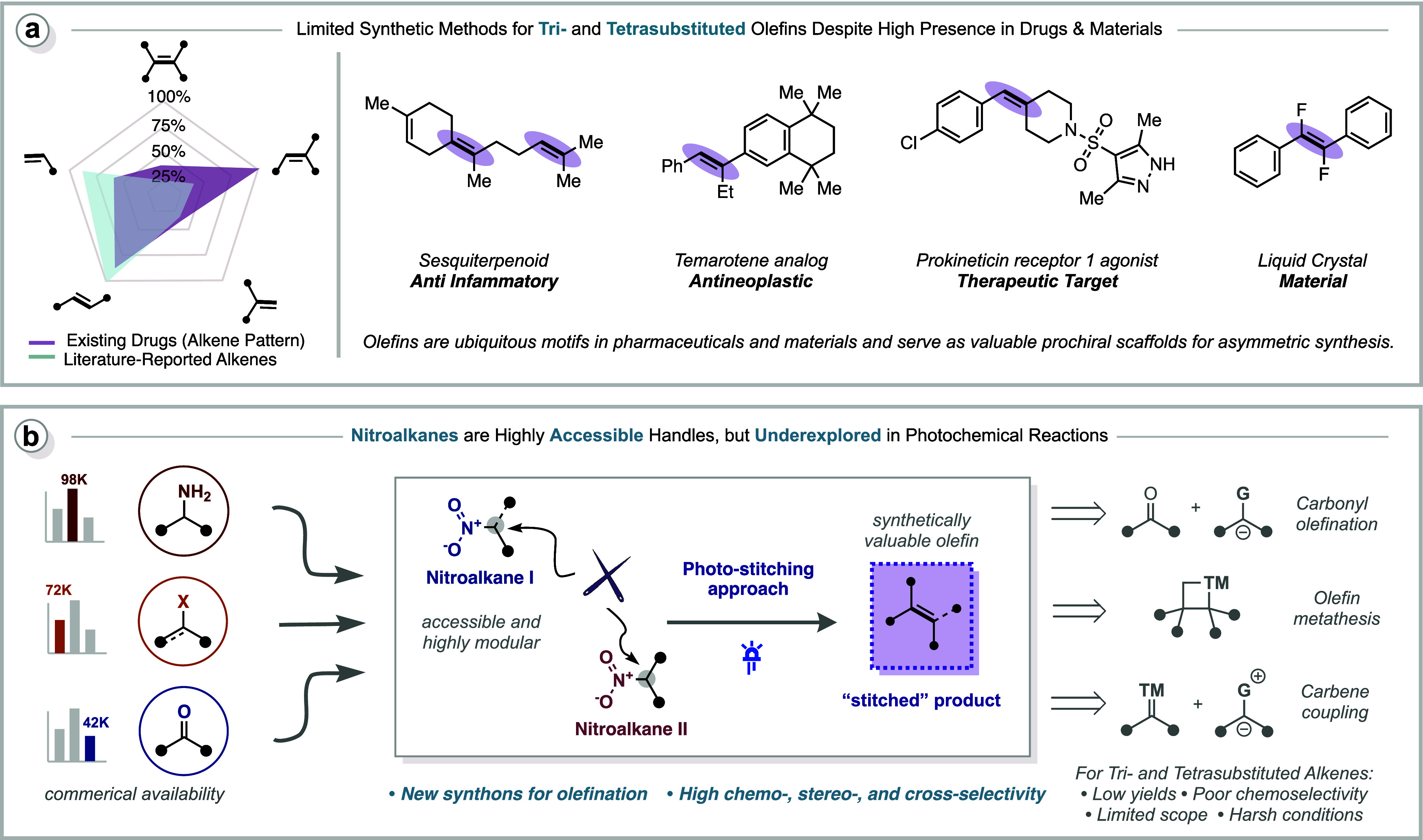
(a) Left: Comparison of normalized frequencies of alkene substitution patterns found in approved drugs (purple) to normalized counts of literature-reported substances bearing those alkene patterns for which synthetic preparations are known (cyan); Right: Bioactive and materials-relevant compounds with acyclic tri- and tetrasubstituted alkenes. (b) Left: commercially available precursors that provide access to nitroalkanes, Middle: our proposed concept, Right: previous olefination methods.

Carbonyl olefination, a cornerstone strategy for alkene formation (Figure 1b), illustrates these limitations clearly. Wittig, Julia, and Peterson olefinations, as well as related reactions, offer modular reagents and broad functional-group tolerance, but predominantly deliver mono- and disubstituted alkenes.^14–16^ Of these, Julia–Kocienski/Lythgoe and Peterson olefinations can access selected tri- and tetrasubstituted alkenes but rely on strong bases and are prone to premature β-elimination, compromising stereocontrol and limiting generality.^14,17,18^ These constraints are amplified in late-stage settings, where ketones pose chemoselectivity challenges and alcohols may be incompatible under strongly basic conditions; both functional groups are prevalent in approved drugs (10–33%).^19^ Metallocarbene-based olefination strategies exhibit similar trends: olefin metathesis reliably forms mono- and disubstituted olefins and selected trisubstituted systems but generally struggles with higher substituted systems due to steric congestion of the metallacyclobutane intermediate.^2,20–22^ Additionally, catalyst deactivation by polar functional groups can pose an issue.^23,24^ Relatedly, carbonyl–olefin metathesis (COM) offers complementary reactivity and has advanced through Lewis acid and organocatalytic approaches, including hydrazine-mediated variants with improved functional-group tolerance and broad applicability.^25–28^ Modular access to highly substituted acyclic alkenes via intermolecular COM, however, remains limited.^26^ In contrast, classic transition-metal-catalyzed cross-coupling reactions can furnish densely substituted alkenes, although they require precious metals and employ an existing alkene electrophile as the coupling partner.^29^ Alternative strategies for olefination involving diazo/carbenoid pathways either require hazardous stabilized precursors^30^ or funnel into dimerization pathways that yield disubstituted alkenes.^31^ Overall, despite decades of innovation, a general, mild, and selective approach to access tri- and tetrasubstituted alkenes is a synthetic challenge. Herein, we report a new olefination strategy to address several of these limitations by leveraging highly accessible and modular nitroalkanes, which can be derived from a broad pool of commercially available precursors,^32–35^ for cross-denitrative olefination under visible light irradiation (Figure 1b). Notably, this approach involves nitronates as new photoreductants to yield tri- and selected tetrasubstituted alkenes without the use of strong bases and/or precious transition metals, providing a complementary approach to existing olefination strategies.

Beyond classical two-electron strategies, single-electron pathways, such as McMurry and Julia–Lythgoe olefinations, as well as Kornblum’s reductive olefination of vicinal dinitroalkanes, offer mechanistically distinct and conceptually appealing routes to sterically congested alkenes.^14,36–39^ McMurry coupling proceeds through single-electron reduction of carbonyl substrates and reductive C–C bond formation, whereas classical Julia olefination converts β-functionalized sulfones to alkenes through a reductive fragmentation sequence (Figure 2a). Although these methods provide important precedents for reductive alkene formation, their use of strongly reducing reagents can constrain functional-group compatibility. Among these precedents, the more closely related nitro-based single-electron-transfer (SET) approaches relied on the reductive fragmentation of vicinal difunctionalized alkanes, including substrates accessed through radical nucleophilic substitution (S_RN_1) chemistry, first defined by Russell and Kornblum (Figure 2b and c, left).^37–39^ Many examples also employed strongly reducing stoichiometric reagents, including alkali metals, metal amalgams, or tin reagents. Kornblum’s sulfide/light variant avoids these metal-based reductants but still requires stoichiometric sulfide, introducing sulfide-derived inorganic waste and potential off-gas handling considerations.^40–43^ Regardless of the reductant, such vicinal difunctionalized nitro substrates remain highly energetic and are often derived from hazardous prefunctionalized precursors under oxidizing conditions (Figure 2c, left), limiting broader adoption of the approach.^33,44,45^

**Figure 2:**
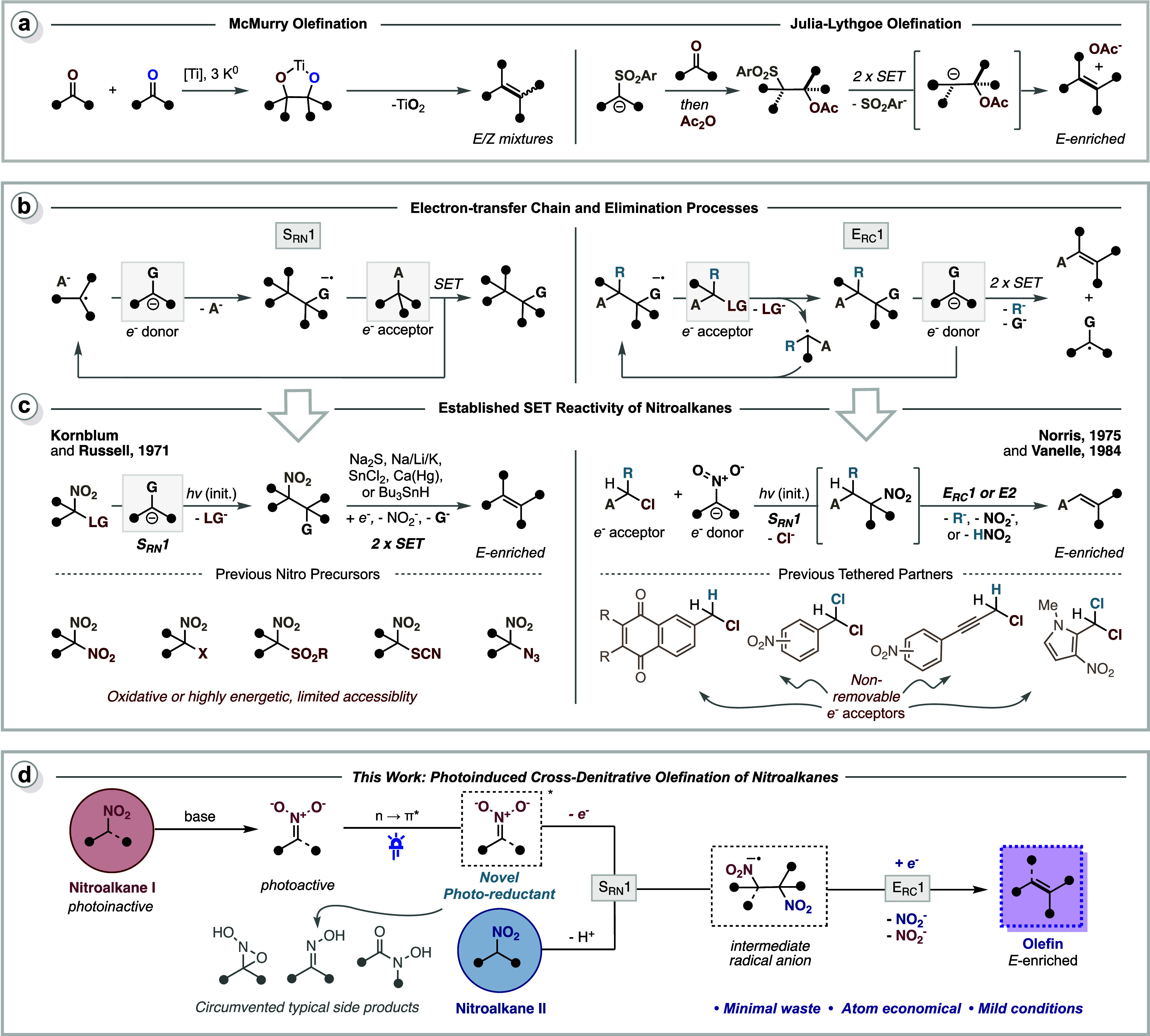
(a) Left: McMurry, Right: Julia–Lythgoe. (b) Traditional S_RN_1 reaction mechanism; Right: E_RC_1 reaction mechanism. (c) Left: two-pot method to access alkenes from S_RN_1 and E_RC_1 pathways; Right: one-pot method to access alkenes from S_RN_1 and E_RC_1/E2 pathways. (d) This work.

A key mechanistic advance emerged from Norris,^46–48^ who showed that sequential SET events in an S_RN_1 process could generate a radical-anion intermediate that undergoes stereodefined radical chain elimination (E_RC_1) in one pot. In this sequence, the resulting chain carriers regenerate key intermediates and propagate the reaction without relying on hazardous prefunctionalized precursors (Figure 2c, right). Vanelle^49–52^ expanded this S_RN_1–E_RC_1 merger, demonstrating that a single anionic substrate could initiate radical substitution, serve as an association partner, and ultimately act as the reductant that induces elimination. Despite these conceptual advances, this strategy is rarely utilized since it necessitates substrates with nonremovable electron-accepting groups and finely tuned redox windows (Figure 2c, right).

We envisioned an approach in which two easily accessible nitroalkanes,^32–35^ rather than prefunctionalized nitro compounds, could be directly “stitched.” We define “stitching” here as a single synthetic operation that joins two previously unconnected reactive centers through the formation of a higher-bond-order linkage, with each center undergoing a formal change in hybridization, in this case forming a C=C linkage from two C­(sp^3^) centers rather than the C–C single bond expected from conventional coupling (Figure 2d). Building on our work and that of others involving photoexcited *N*-centered 1,3-dipoles,^53–64^ we postulated that in situ–generated nitronates, themselves *N*-centered 1,3-dipoles, could be selectively photoexcited under controlled conditions (Figure 2d). We reasoned that excitation under visible light would bypass rearrangements often observed under ultraviolet irradiation and, instead, generate a novel nitronate photoreductant capable of reducing a second nitroalkane through SET. In this design, the photoexcited nitronate performs two sequential roles. First, it acts as a single-electron donor to initiate an S_RN_1-type pathway that enables formal coupling between nucleophilic partners, giving rise to radical–anion intermediates characteristic of Kornblum chemistry. Second, it participates in an E_RC_1 elimination process, where the nitro groups serve as traceless leaving groups. This elimination cascade (radical–anion → carbanion → β-elimination, Figure 2d) provides a stereodefined route to alkenes without requiring highly energetic, prefunctionalized vicinal/geminal nitro substrates or strong reductants. Since nitroalkanes are abundant, modular, and readily prepared,^33–35^ this tandem light-driven S_RN_1–E_RC_1 sequence provides a complementary strategy for the synthesis of substituted alkenes from nitroalkane building blocks.

## Results and Discussion

To test our hypothesis, (nitromethyl)­benzene and 2-nitropropane were subjected to 427 nm irradiation with Cs_2_CO_3_ as a mild base and 1,2-difluorobenzene as solvent. We surmised that (nitromethyl)­benzene would be an ideal coupling partner, as its corresponding nitronate, generated under basic conditions, could selectively absorb visible light to trigger the desired olefination event. Under these reaction conditions, the targeted trisubstituted alkene product, (2-methylprop-1-en-1-yl)­benzene (**P0**), was obtained in 33% ^1^H NMR yield, accompanied by substantial formation of a homodimeric byproduct (see Supporting Information). To suppress this byproduct and favor cross-olefination, excess 2-nitropropane was employed, and *tert*-butanol was introduced as a cosolvent, resulting in improved yield of the desired product (**P0**). Once the optimized reaction conditions were obtained (see Supporting Information), the electronic effect of the photo-stitching reaction between benzylic nitroalkanes (“activated”) and nonbenzylic aliphatic nitroalkanes (“unactivated”) was examined (Figure 3, **P1**–**P12**).

**Figure 3:**
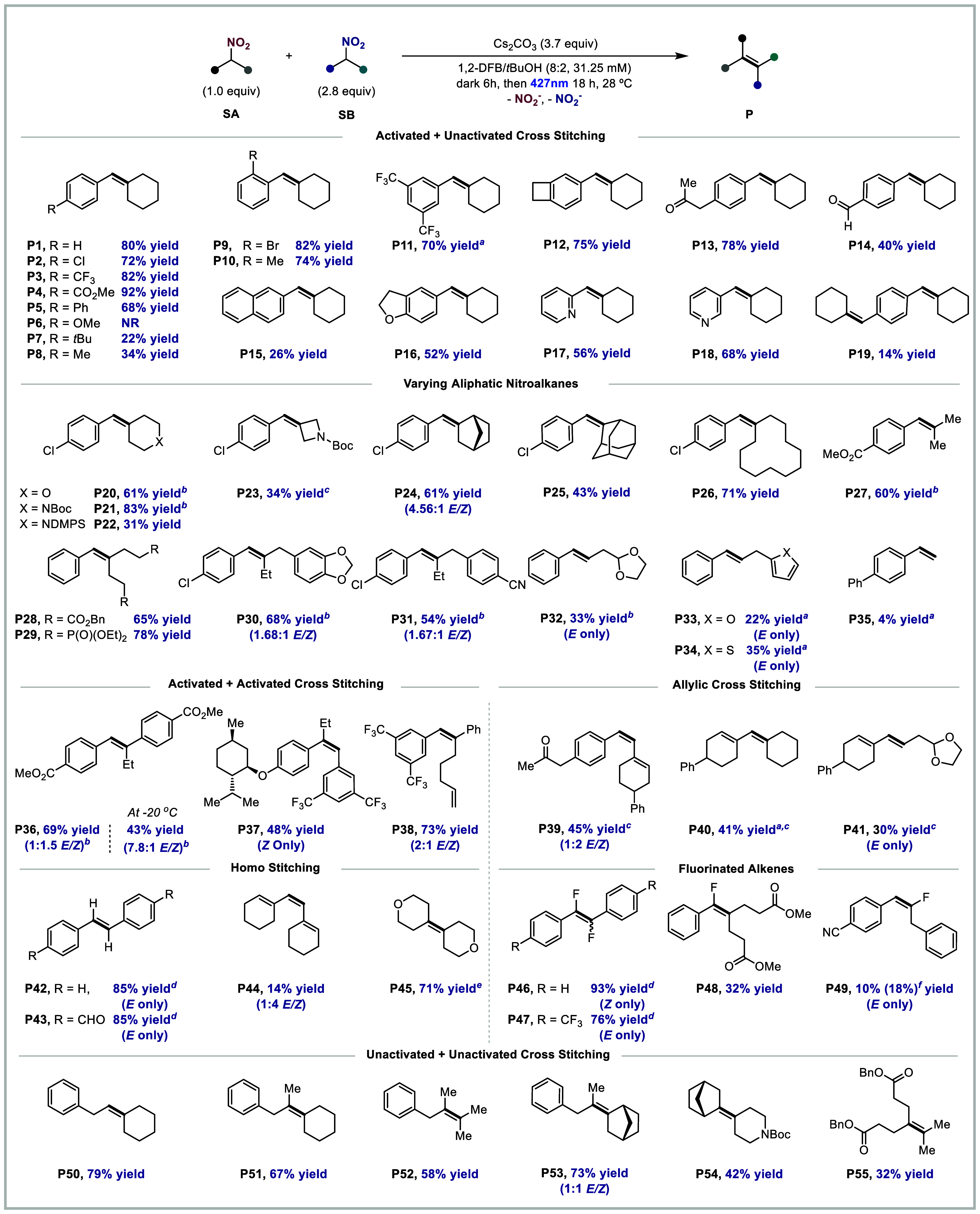
Conditions: SA (1.0 equiv, 31.25 mM; provides the left fragment of the product), SB (2.8 equiv; provides the right fragment of the product), and dried Cs_2_CO_3_ (3.7 equiv) in 1,2-difluorobenzene/*t*BuOH (4:1); the mixture was stirred for 3–6 h and then irradiated for an additional 18 h under 427 nm light. ^*a*^ Denoted as ^1^H NMR yield using CH_2_Br_2_ as an internal standard. ^*b*^ At 50 mM with EtOAc/ *t*BuOH (4:1). ^*c*^ At 31.25 mM with DCE/EtOH (4:1). ^*d*^ Only 1–2 h under 427 nm light. ^*e*^ With 10 mol % 4-(dichloromethyl)­benzonitrile as an additive. ^*f*^ Defluorinated product. DMPS = (3,5-dimethyl-1*H*-pyrazol-4-yl)­sulfonyl.

Activated benzylic nitroalkanes possessing electron-deficient aryl substituents performed efficiently (**P2**–**P4**); conversely, electron-rich aryl substituents resulted in lower to moderate reaction yields (**P5**–**P8**), and para-methoxy substitution (**P6**) resulted in no reaction. These outcomes are likely a consequence of polarity-matching, as electron-deficient benzylic nitroalkanes generate more electrophilic α-nitro radicals; they are better matched for productive association with nucleophilic nitronates, analogous to radical–anion pairing in S_RN_1-type chemistry. Ortho- and meta-substituted substrates provided **P9**–**P12** in moderate to high yields (70–82%). Carbonyl-containing substrates, traditionally problematic in most olefination protocols, were well tolerated under our transformation, resulting in alkenes **P13**–**P14** in moderate to high yields (40–78%) with no Henry products detected. Despite naphthalene’s propensity to quench excited states in photochemical reactions, photo-stitching of a naphthalene-bearing substrate afforded **P15** in modest yield. Nitroalkanes bearing a pyridine moiety, which are often problematic for transition-metal-catalyzed cross-coupling and/or olefin metathesis, underwent olefination smoothly (**P17**–**P18**; 56%, 68%). In contrast, extension of the method to a dinitroalkane with excess nitrocyclohexane and base furnished the desired bis-functionalized product **P19** in low yield, with major unknown decomposition byproducts.

Next, variation of the unactivated nitroalkane partner was explored. Unactivated nitroalkanes containing heterocyclic motifs afforded **P20**–**P21** in 61–83% yield, and a prokineticin receptor 1 agonist derivative (**P22**)^65^ was obtained in 31% yield. A strained azetidine nitroalkane was also tolerated to give **P23** in 34% yield, while bicyclic **P24** was produced in 61% yield with good stereoselectivity (4.56:1 *E*/*Z*). Sterically congested **P25**, which is typically challenging to access through conventional Wittig olefination, was obtained in 43% yield. Larger ring nitrocyclododecane provided **P26** in 71% yield. Olefination of symmetrical acyclic nitroalkanes with (nitromethyl)­arenes delivered **P27**–**P29** in moderate yields, tolerating esters and phosphonates, which are unlikely to persist under harsh basic conditions. Unsymmetrical, unactivated acyclic nitroalkanes furnished trisubstituted alkenes **P30**–**P31**, demonstrating compatibility with heterocycles and acetal groups. Benzylic nitroalkanes coupled with primary nitroalkanes afforded *E*-enriched 1,2-disubstituted alkenes in low to moderate yields (**P32**–**P34**, 22–35%). Unfortunately, attempts to access terminal alkenes via the cross-denitrative olefination of a (nitromethyl)­arene and nitromethane resulted in only 4% ^1^H NMR yield of the desired product (**P35**), and secondary benzylic nitroalkanes, such as (1-nitroethyl)­benzene, failed to deliver significant tri- or tetrasubstituted olefins, instead leading to major Nef-type products (see Supporting Information for details).

We then evaluated whether activated/activated systems could be photo-stitched. Under our standard conditions at 28 °C, product **P36** was obtained in 69% yield at a 1:1.5 *E*/*Z* ratio. In contrast, conducting the reaction at −20 °C afforded **P36** in a 43% yield with improved selectivity (7.8:1 *E*/*Z*). However, subsequent warming of the −20 °C reaction mixture and extended reaction times resulted in erosion of the *E*/*Z* ratio. These observations suggest that the stilbene products are initially formed predominantly as the *E*-isomers and subsequently undergo isomerization to the corresponding *Z*-isomers over time under reaction conditions. The (−)-menthol derivative **P37** was synthesized in only the *Z* form at 48% yield. Furthermore, reaction of 3,5-bis­(trifluoromethyl)­nitromethylbenzene with (1-nitrohex-5-en-1-yl)­benzene, containing a pendant alkene, afforded **P38** in a 73% yield with a 2:1 selectivity for the *E*-alkene.

Allylic nitroalkanes were also examined to determine whether they participate in cross-stitching to afford synthetically useful dienes. Olefination of cyclic allylic nitrocycloalkanes with both secondary and primary nitroalkanes afforded dienes **P39**–**P41** in 30–45% yield. Attempts at acyclic allylic substrates led to unknown decomposition products. Homo-stitching of activated (**P42**–**P43**) and unactivated nitroalkanes (**P45**) occurred in high yields for the activated substrates; high *E*-selectivity was achieved under a time-controlled run. Notably, 10 mol % 4-(dichloromethyl)­benzonitrile promoted otherwise unproductive or lower-yielding dimerization reactions, including examples with nitrocyclohexane and 4-nitrotetrahydro-2*H*-pyran derivatives, suggesting that modest inductive effects on the alkyl chain can facilitate productive coupling (see Supporting Information). Reaction of an allylic nitrocycloalkane resulted in a triene (**P44**) in 14% yield with a 1:4 *E*/*Z* ratio, where the *Z*-selective outcome likely arises from photoisomerization of the *E*-product.^66^

α-Halogen-substituted nitroalkanes displayed halogen-dependent reactivity: benzylic chloride- and bromide-derived nitroalkanes did not produce the desired alkene products, whereas α-fluoro nitroalkanes performed efficiently. Liquid crystal products **P46**–**P47**, which previously required the use of tetrafluoroethylene and Grignard reagents, were formed in high yields (76–93%). For **P46**, irradiation for 30 min afforded the alkene product as a 43:56 *E*/*Z* mixture, demonstrating that the *E*-isomer is initially accessible under the reaction conditions. Continued irradiation for 2 h provided only the *Z*-isomer, consistent with rapid *E → Z* photoisomerization in the presence of nitrite and base. In contrast, with *p*-CF_3_ substitution (**P47**), selective formation of the *E*-alkene could be obtained in 1 h of irradiation (85% yield), as isomerization of the *E* product is slower. Reaction of α-fluoro­(nitromethyl)­benzene with an unactivated partner, dimethyl 4-nitroheptanedioate, furnished **P48** in 32% yield, while unactivated α-fluoro nitroalkanes gave a low yield of the desired product (**P49**, 10%), with mainly the defluorinated product detected (18% ^1^H NMR yield).

Next, we tested whether unactivated nitroalkanes could undergo cross-denitrative olefination. We anticipated that this would be challenging since the formed alkylnitronates are nonconjugated and might be less reactive under photoirradiation. Remarkably, when nitrocyclohexane and (2-nitroethyl)­benzene were subjected to the reaction conditions, trisubstituted alkene **P50** was formed in 79% yield. Similarly, olefination of (2-nitropropyl)­benzene with nitrocyclohexane led to a 67% yield of a tetrasubstituted alkene **P51**, with 25% and 19% ^1^H NMR yields of the homo-dimer byproducts from the limiting reagent and excess substrate, respectively. A tetrasubstituted alkene (**P52**) was formed in a 58% yield via cross-denitrative olefination of an acyclic aliphatic secondary nitroalkane with another acyclic aliphatic nitroalkane. Notably, a norbornene-bound cyclic nitroalkane underwent olefination with a secondary nitroalkane and a Boc-protected nitropiperidine, leading to alkenes **P53** and **P54**, respectively. Lastly, olefination of nitroalkanes possessing a diester functionality, which is challenging to tolerate under Julia conditions, resulted in alkene **P55** in 32% yield.

The observed stereochemical outcomes further define the relationship of this transformation to established olefination methods. Product selectivity may first be influenced by C–NO_2_ bond cleavage from the coupled radical-anion intermediate, with fragmentation potentially favoring formation of the more stabilized carbon-centered radical. The structure and conformational preferences of this radical and, following reduction, the resulting carbanion may then both influence the stereochemical course of elimination. Consistent with this interpretation, selectivity was diminished when competing C–NO_2_ bond-cleavage pathways would generate radicals of similar stability (**P30**–**P31**; **P39**), whereas greater differences in the stabilities of the resulting radicals were associated with higher selectivity (**P32**–**P34**; **P41**). In this respect, the reaction resembles aspects of Julia–Lythgoe olefinations, where alkene geometry can also depend on conformational reorganization of the radical and/or carbanion intermediates prior to alkene formation. Prior Julia–Lythgoe reactions show that phenyl-substituted sulfones can furnish *E*/*Z* mixtures with alkyl aldehydes,^14^ while more electron-rich aryl variants can improve *E*-selectivity in selected cases. In contrast, although examples were obtained in low to moderate yield, the present method provides *E*-selective products from unsubstituted phenyl systems or cyclic allylic systems (**P24**, **P32**–**P34**, **P36**, **P41**, **P42**–**P43**, **P47**, **P49**), suggesting that high *E*-selectivity can be achieved without strong electronic bias from aryl substituents. In this regard, this cross-denitrative olefination may offer a milder but complementary approach to selected *E*-enriched, highly substituted alkenes relative to McMurry-type and Julia–Lythgoe-type olefinations, for which stereochemical control in unsymmetrical intermolecular couplings can be challenging. Nevertheless, more studies are needed to achieve the level of predictable *E*-selectivity achieved in optimized Julia–Kocienski olefinations, and further development will be required to improve stereocontrol across broader aliphatic substrate classes.

A distinct advantage of this method is illustrated through comparison with olefin metathesis (Figure 4a). With Schrock-type catalysts, Type IV olefins are unreactive, whereas Type I and Type II olefins participate readily.^20^ As a result, the selective formation of a trisubstituted alkene over the corresponding 1,2-disubstituted alkene is generally not observed under cross-olefin metathesis conditions. Under our transformation, the trisubstituted product **P56** can be accessed in moderate yield.

**Figure 4:**
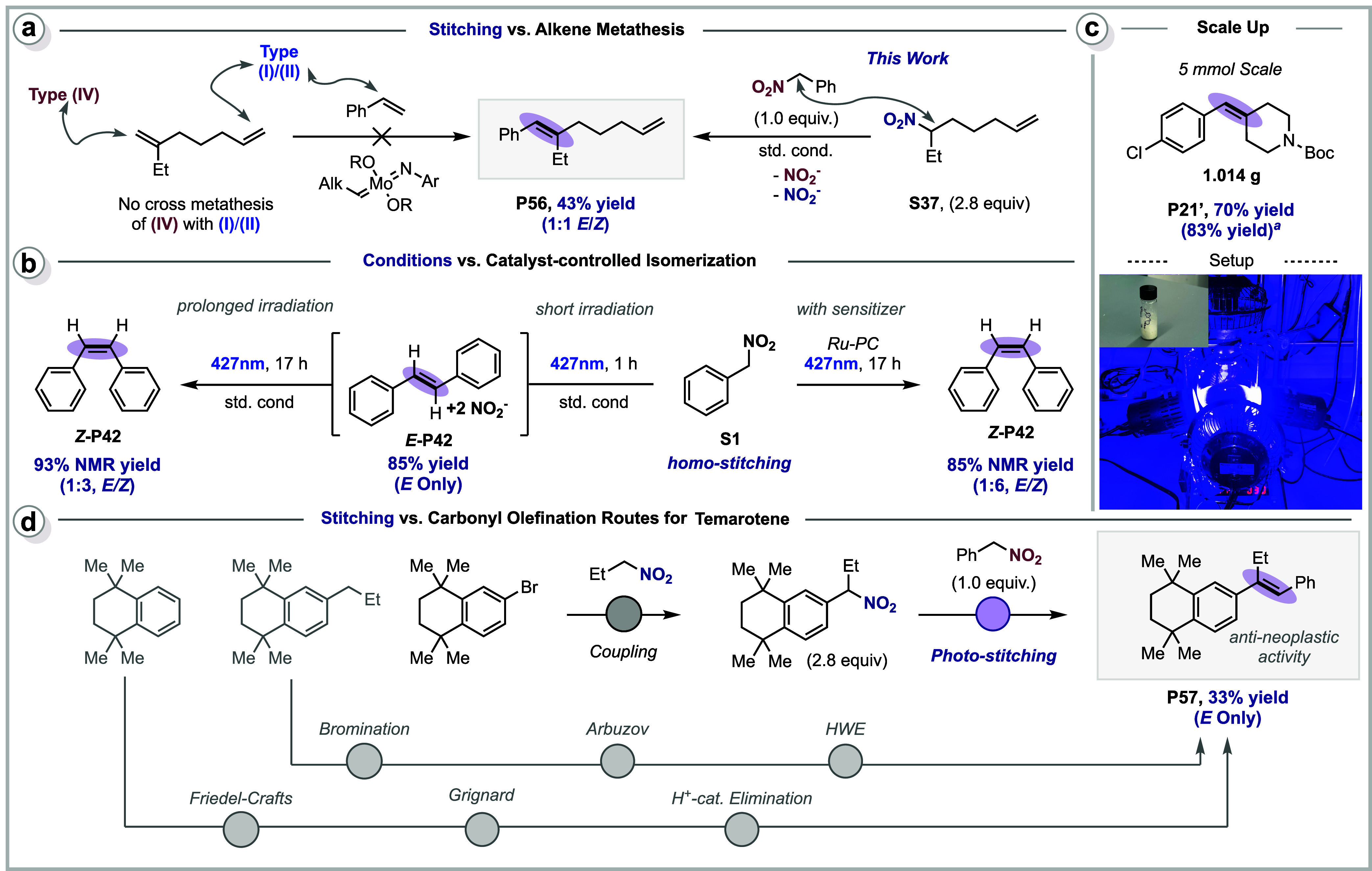
(a) Cross-metathesis olefination reaction versus proposed work for selective access of trisubstituted olefins. (b) Photoisomerization of stilbene products in situ under photocatalytic conditions. (c) Gram-scale example. (d) Synthesis of temarotene analogue. ^*a*^ 0.45 mmol scale.

Although *E*-enriched stilbenes are accessible under controlled reaction times, the *E*-products partially photoisomerize upon prolonged irradiation under the reaction conditions. To determine whether this isomerization could be leveraged to favor the *Z*-isomer, we subjected the stilbene product, **P42**, derived from (nitromethyl)­benzene, to the reaction conditions in the presence of various photocatalysts (see Supporting Information). Under 427 nm irradiation, Tris­(2,2′-bipyridyl)­dichlororuthenium­(II) hexahydrate (Ru­(bpy)_3_Cl_2_·6H_2_O) promoted formation of the *Z*-enriched product in a 1:6 *E*/*Z* ratio, whereas lower *Z*-selectivity was observed in the absence of photocatalyst (Figure 4b). This result is consistent with related work featuring photocatalytic *E* → *Z* alkene isomerization reported by Reiser et al.^67^

The scalability of the reaction was then demonstrated using **P21**, also a precursor to **P22**, as a representative example. Increasing the reaction scale from 0.25 to 5.0 mmol afforded 1.0 g of isolated product (**P21′**, 70% yield) without appreciable loss of efficiency (Figure 4c). Finally, we illustrated a streamlined synthesis of a temarotene analogue **P57** (Figure 4d). Prior syntheses required three steps under harsh reaction protocols; our photo-stitching approach delivers the product in two operationally simple steps from a commercially available aryl bromide under milder conditions.

We began our mechanistic investigation by determining how the reaction is initiated. Control experiments (Table S4) established that light irradiation is required. UV–vis spectroscopy revealed that in situ–generated nitronates are the sole absorbing species within the irradiation window (see Supporting Information), whereas nitroalkanes remain photoinactive (Figure 5a). No discrete charge-transfer complex was detected by UV–vis or NMR titration, although the possibility of dynamic quenching cannot be excluded.

**Figure 5:**
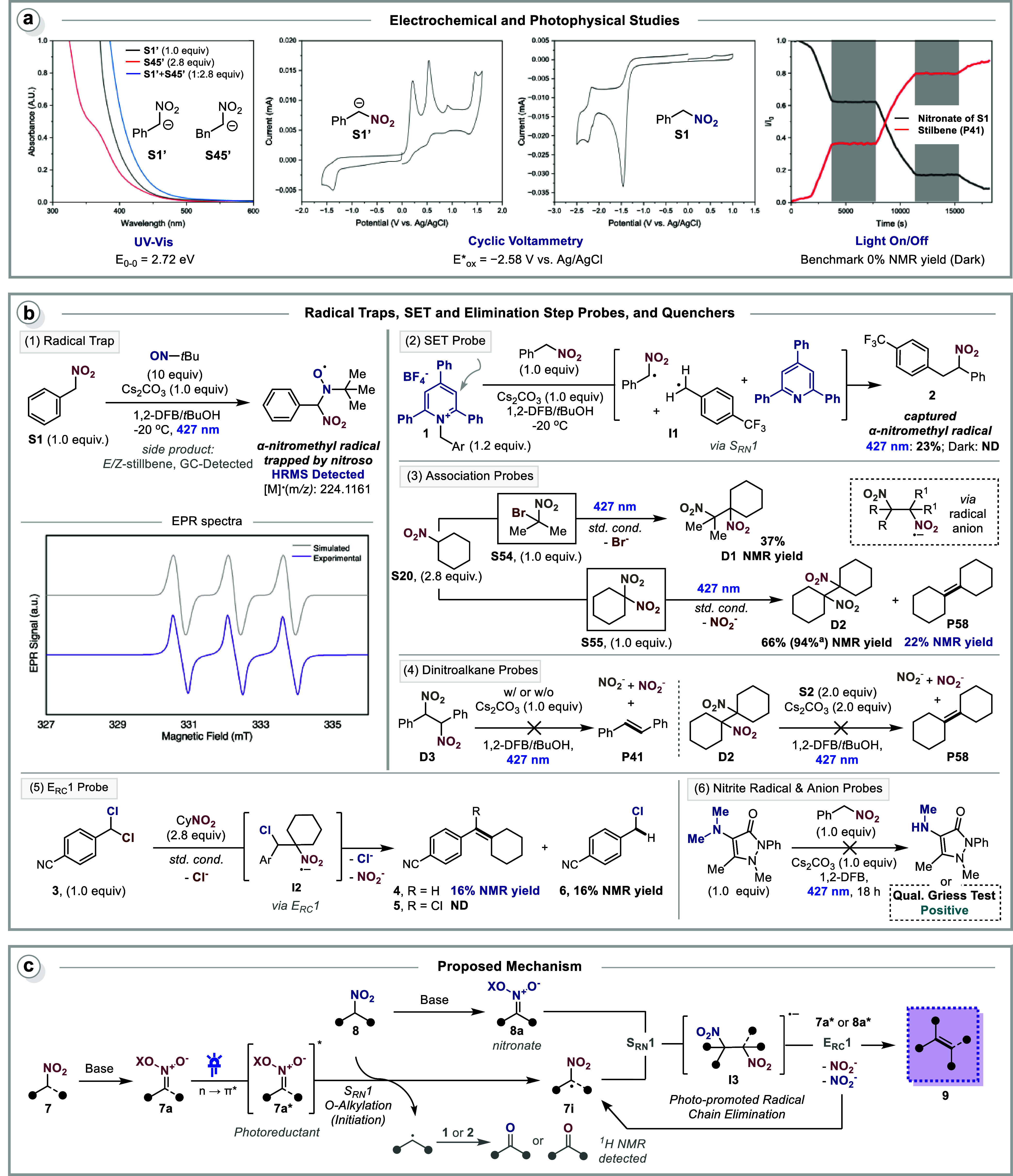
(a) Electrochemical and photophysical studies: UV–vis spectroscopy, cyclic voltammetry, and light on/off studies using continuous PhotoNMR. (b) Radical trap, single-electron-transfer (SET), and elimination probes. ^a^ With 1.0 equiv of **S20**. (c) Proposed mechanism.

Next, we examined whether these photoabsorbing nitronates could promote a SET event, a characteristic step of S_RN_1 initiation, using the homo-stitching reaction to **P42** as a benchmark. To assess whether photoexcited nitronates possess sufficient driving force, we quantified their excited-state redox properties. The excited oxidation potential of the cesium nitronate from (nitromethyl)­benzene was calculated as E*_ox_ (M*/M^+^) = −2.58 V vs Ag/AgCl from its ground-state oxidation potential (*E*_p/2,ox,1_ (M/M^+^) = +0.142 V vs Ag/AgCl) and electronic-transition energy (*E*_0–0_ = 2.72 eV) (Figure 5a).^68^ When paired with the nitroalkane’s reduction potential (*E*_p/2,red_ (A/A^–^) = −1.39 V vs Ag/AgCl), Rehm–Weller analysis^69^ indicates that electron transfer from M* to A is strongly exergonic (Δ*G*° ≈ −114.9 kJ mol^–1^; −1.19 eV), establishing that in situ–generated nitronates can serve as photoreductants. This assignment was further supported computationally (Table S6), and the measured ground-state oxidation potential was comparable to that reported by Savéant for nitronate salts derived from 2-nitropropane (+0.077 V vs SCE).^70^ Additional experiments corroborate a SET pathway, as diagnostic quenchers,^38,71,72^ commonly used to perturb radical or electron-transfer pathways, including catalytic *p*-dinitrobenzene, stoichiometric TEMPO, and O_2_ present in the headspace of a closed reaction vial, markedly reduced product formation (Table S4), which is in agreement with prior S_RN_1 mechanistic findings. Continuous PhotoNMR studies revealed an induction period for the generation of carbon-centered radicals, and control studies support that initiation requires sacrificial nitroalkane (Table S7). Previous work by Norris and Vanelle established that thermal E_RC_1 propagation can occur in systems bearing nonremovable, conjugated electron-accepting groups, such as nitro or quinone substituents, which enable ground-state nitronates to reduce the resulting radical-anion intermediates within a favorable redox window.^46,49^ In the present system, the corresponding ground-state redox events are not sufficiently favorable to support an analogous thermal chain. Consistent with this, light on/off experiments confirmed that constant irradiation is necessary to propagate the reaction (Figure 5a),^70^ thereby ruling out a self-sustaining thermal E_RC_1 chain under the present conditions. The slow fragmentation of the radical-anion intermediate^46,47^ may allow it to persist during dark intervals, accounting for the absence of a second induction period upon reirradiation. Detection of trace 1,2-dinitroalkane by GC–MS upon completion of some of the reactions is consistent with a radical-termination event, potentially through the quenching of the radical-anion intermediate as the nitronate pool is depleted, or to a lesser extent, the dimerization of residual α-nitro radicals.

Next, spin-trapping experiments were conducted to support the formation of the α-nitro radical intermediate via SET oxidation of the photoexcited nitronates. Treatment with 2-Methyl-2-nitrosopropane afforded the corresponding nitroxide spin adduct as verified by HRMS (224.1161 *m*/*z*) and X-band CW EPR spectroscopy (Figure 5b-1).^73^ We then assessed whether the α-nitro radical participates in noncanonical S_RN_1-type chemistry. Hence, we subjected an alternative electron acceptor, Katritzky salt **1**, to an in situ–generated nitronate under photoirradiation and observed cross-coupled product **2** (C–C bond formation) in 23% ^1^H NMR yield (Figure 5b-2), with no cross-stitched product (C=C bond) detected. These observations are consistent with prior photochemical S_RN_1 reactivity involving Katritzky salts.^74^

While the Katritzky salt experiment supports SET-mediated generation of two distinct radical partners followed by productive radical-pair coupling,^74^ the cross-denitrative olefination requires formation of a radical-anion intermediate between an α-nitro radical and a nitronate coupling partner. Due to philicity mismatch, we posited that electron-deficient α-nitro radicals do not readily dimerize; instead, they preferentially undergo an association event with electron-rich/nucleophilic nitronates.^75^ Prior studies show that the efficiency of such pairings depends on sterics and radical-anion stability.^39,43^ To evaluate whether α-nitro radicals generated under our reaction conditions behave similarly (i.e., association), we examined geminally functionalized nitroalkanes **S54** and **S55** as mechanistic probes in the presence of the nitronate salt of **S20** (Figure 5b-3). These substrates are literature-precedented α-nitro radical precursors under photochemical electron-transfer conditions and therefore provide a means to probe radical-anion formation with nitronates.^43,75^ Given that **S54** and **S55** can serve as SET oxidants, the resulting radical-anion can be quenched to afford 1,2-dinitroalkanes, whereas direct dimerization of the α-nitro radical itself is reportedly disfavored.^75^ Consistent with this pathway, **S54** afforded the cross-coupled 1,2-dinitroalkane **D1** in 37% NMR yield. Similarly, **S55** provided the corresponding 1,2-dinitroalkane **D2** in 94% NMR yield when 1 equiv of nitronate salt was employed. Overall, the observation of the 1,2-dinitroalkane products (**D1** and **D2**) supports an association event during the course of the reaction. For the coupling of **S55** with excess nitronate salt (2.8 equiv of **S20**), however, **D2** was detected in 66% NMR yield along with alkene **P58** in 22% NMR yield. This outcome is consistent with increased competition from E_RC_1 fragmentation of the radical-anion intermediate as the SET oxidant was consumed. To determine whether alkene formation arises from reduction of the radical-anion intermediate, reduction of the quenched 1,2-dinitroalkane, or both, control studies were performed under the reaction conditions (Figure 5b-4). These experiments showed that the 1,2-dinitroalkane products are not further reduced to the corresponding alkenes, suggesting alkene formation is likely from the reduction of the radical-anion intermediate rather than from quenched 1,2-dinitroalkane.

Having established that alkene formation likely arises from reduction of the radical-anion intermediate rather than ground-state 1,2-dinitroalkane, we next sought to define the nature of the reduction/elimination sequence. Subsequent SET reduction events that lead to elimination products through radical-chain elimination pathways, termed radical-chain elimination (E_RC_1), are exceedingly rare, as classical E_RC_1 reactions are thermally propagated through redox-favorable carriers. We posited that our reaction proceeds through a photodriven E_RC_1 from the excited nitronate. To probe this, we employed 4-(dichloromethyl)­benzonitrile (**3**) as an E_RC_1 probe (Figure 5b-5). SET from the photoexcited nitronate should generate an α-chloro radical that undergoes association (**I2**) to form a radical anion and follows a pathway analogous to that reported by Kornblum, Norris, and Vanelle.^38,39,48,49^ Under our conditions, **4** was formed in 16% ^1^H NMR yield, supporting an E_RC_1 mechanism. To distinguish homolytic radical fragmentation from β-elimination, we evaluated the nature of the cleaved nitro groups. A qualitative Griess test confirmed nitrite formation during the elimination step, whereas the detection of nitryl radicals was negative (Figure 5b-6). Based on these studies and the *E*-selectivity of the transformation, β-elimination is favored over a radical elimination pathway for alkene formation. An alternative E2 process following a noncanonical S_RN_1 reaction (Figure 5b-2) was ruled out based on our elimination probes (see Supporting Information) and the absence of **5** from the E_RC_1 probe reaction (Figure 5b-5). Cumulatively, these findings support a photoinitiated and photopropagated S_RN_1-E_RC_1 sequence. Other mechanistic possibilities, such as carbene intermediates or nitronate photorearrangements, were experimentally excluded (see Supporting Information). Based on these studies, we propose the following mechanism (Figure 5c). Nitroalkane **7** is deprotonated under mild basic conditions to form nitronate **7a**, which undergoes photoexcitation (**7a** → 7a*) and engages in a SET event with a second nitroalkane, generating the α-nitro radical **7i** along with other carbon-centered radical byproducts. Concurrently, nitroalkane **8** is converted to nitronate **8a**, which associates with **7i** to give radical-anion intermediate **I3**. This intermediate undergoes further reduction by the photoreductant 7a* (or by 8a* if photoexcitable), propagating the chain to regenerate **7i**. Radical recombination pathways or quenching of radical-anion intermediates may terminate the chain, with either pathway furnishing trace 1,2-dinitroalkanes.

## Conclusion

In conclusion, we have devised the first direct cross- and self-olefination of nitroalkanes under simple conditions involving base and visible-light irradiation. The ability to “stitch” two nitroalkanes is enabled by leveraging a historically underexplored class of nitronate photoreductants for the synthesis of valuable alkene cores, primarily tri- and selected tetrasubstituted variants, that are challenging to access through classical olefination methods. These findings establish nitronates as promising photoreductants and coupling partners for the development of new organic transformations.

## Supplementary Material



## Notes

Experimental details, optimization studies, characterization data, and NMR spectra (PDF)

## Abbreviations

S_RN_1: radical-nucleophilic substitution
E_RC_1: radical-chain elimination
CV: cyclic voltammetry
EPR: electron paramagnetic resonance
1,2-DFB: 1,2-difluorobenzene
NMR: nuclear magnetic resonance
UV–vis: ultraviolet–visible
GC–MS: gas chromatography–mass spectrometry
PhotoNMR: photochemical nuclear magnetic resonance
TEMPO: 2,2,6,6-tetramethylpiperidine 1-oxyl
SET: single-electron-transfer
Elim: elimination
Rot: rotation
DMPS: (3,5-dimethyl-1*H*-pyrazol-4-yl)­sulfonyl
