# Supplementary material for: Photo-Stitching of Nitroalkanes via Cross-Denitrative Olefination
Source: J Am Chem Soc. 2026 Jul 16;148(30):31690–700. doi: 10.1021/jacs.6c06460 (PMC13450392; doi:10.1021/jacs.6c06460)
Supplement: Supplementary file 1 [file ja6c06460_si_001.pdf]

Photo-Stitching of Nitroalkanes via Cross-Denitrative Olefination

Joshua K. Mitchell,† Shrey P. Desai,† Joseph M. Bergen, Alexandria Reyes,§ Maxwell S. McLean,§ Poulami Ghosh, Ajay H. Bansode, and Marvin Parasram*

Department of Chemistry, New York University, New York, New York 10003, United States

Supporting Information

Table of Contents

General Information S3

List of Nitroalkanes S4

General Procedures S5

Optimization Studies S9

Table S1. Solvent screen for cross-stitching activated/unactivated nitroalkanes. S9

Table S2. Time, equivalence, and additional solvents screen. S9

Table S3. Additional solvent screen for cross-stitching activated/unactivated nitroalkanes. S10

Table S4. Control experiments. S10

Table S5. Base and concentration screen. S11

Possible Reaction Pathways S12

List of Possible Reaction Pathways S12

Fragmentation Pathways From the Radical Anion Intermediate of Pathway 5 .... S13

Reasoning for Each Reaction Pathway S14

Mechanistic Studies S15

UV–vis S15

Cyclic Voltammetry S17

Computational Calculations S19

Calculated Redox Potentials S20

Cartesian coordinates S22

Light On/Off Studies S29

Assessing Whether Sacrificial Nitroalkane is Required for Reaction Initiation S31

EPR Study S33

SET Probe S35

Elimination Probes S37
Association Probes S38
Geminal Functionalized Nitroalkane Probe Studies S38
Alternative Nucleophile Traps S41
Vicinal Dinitroalkane Probes S43
Photoinduced Reduction of Activated 1,2-Dinitroalkanes in the Absence of Nitronates S43
Assessment of Unactivated 1,2-Dinitroalkane Reduction by Photoexcited Nitronates S44
$E_{RC}1$ Probe S45
Griess Test for the Detection of Nitrite Ions S47
Nitryl Radical Trap S48
Use of Oxaziridines, Nitronic Esters and Alkyl Nitrites to Probe as Potential Intermediates S49
Probing for Carbene Intermediates S50
Carbene Traps S50
Competitive Carbene Reactions S51
Isomerization of in-situ generated E-Stilbene derivatives S53
Photocatalytic isomerization S53
Photoisomerization of Difluorostilbene S54
Additive influence on Cross-Denitrative Olefination S56
Nef byproducts from the reaction conditions S58
Characterization of Alkenes Obtained from Denitrative Olefination S61
Starting Material Characterization S84
NMR Spectra of Olefination Products S90
NMR Spectra of Starting Materials S151
NMR Spectra of Crude Products S167
References S171

General Information

All requisite chemicals were purchased from Fisher Scientific, Sigma Aldrich (Merck), Oakwood Chemical (Oakwood Products), Ambeed, TCI, and used without further purification unless otherwise stated. All reactions were carried out in oven-dried glassware under a nitrogen atmosphere, unless otherwise stated. Solvents were dried and deoxygenated by passing through alumina in a solvent purification system. $CDCl_3$, CD_2Cl_2, DMSO-d_6, $C_2D_4Cl_2$ and CD_3CN were purchased from Cambridge Isotope Laboratories. 1,2-Difluorobenzene was dried over CaH_2, distilled under an inert atmosphere, and degassed by three freeze–pump–thaw cycles prior to use.

All NMR spectra (^1H, ^{13}C, ^{19}F, and ^{31}P) were recorded on Bruker 400 MHz, 500 MHz, 600 MHz, and 800 MHz Avance spectrometer. ^1H NMR spectra were recorded at 400, 500, and 600 MHz; ^{13}C NMR spectra were obtained at 101 MHz, 126 MHz, and 151 MHz; ^{19}F NMR spectra at 377 MHz and 471 MHz; and ^{31}P NMR spectra at 202 MHz. Spectral data was acquired at 298 K. The chemical shifts (δ) are given in parts per million (ppm) and referenced to residual solvent peaks for 1H and ^{13}C NMR spectra: $CDCl_3$, δ_H 7.26 ppm, δ_C 77.16 ppm; DMSO-d_6, δ_H 2.50 ppm, δ_C 39.52 ppm; CD_2Cl_2, δ_H 5.32 ppm, δ_C 53.84 ppm; CD_3CN, δ_H 1.94 ppm, δ_C 1.32, 118.69 ppm; $C_2D_4Cl_2$ δ_H 3.73 ppm; t-BuOD, δ_H 1.28 ppm. Coupling constants (*J*) are reported in Hertz (Hz) to the nearest 0.1 Hz.

GC chromatograms were taken on an Agilent 8890 GC with 5977B MSD, and helium as the carrier gas. High-resolution mass spectra (HRMS) were obtained on a Waters ACQUITY/Xevo G3 LC-QToF MS and Agilent 6224 TOF LC/MS Dual ESI which was acquired through the support of New York University. We utilized 34 W Kessil Lamps with varying wavelengths for the photochemical setups. Thin-layer chromatography (TLC) was performed on 250-μm glass-backed silica gel plates and column chromatography were performed using 200–300 mesh silica gel unless otherwise stated. EPR (Electron Paramagnetic Resonance) measurements were conducted on a Bruker ELEXSYS E500 Spectrometer System in continuous-wave mode in the X-band frequency using a Bruker ER 4122SHQE resonator. EPR simulations were performed using MATLAB R2025b (MathWorks) and EasySpin (v6.2.0).

List of Nitroalkanes

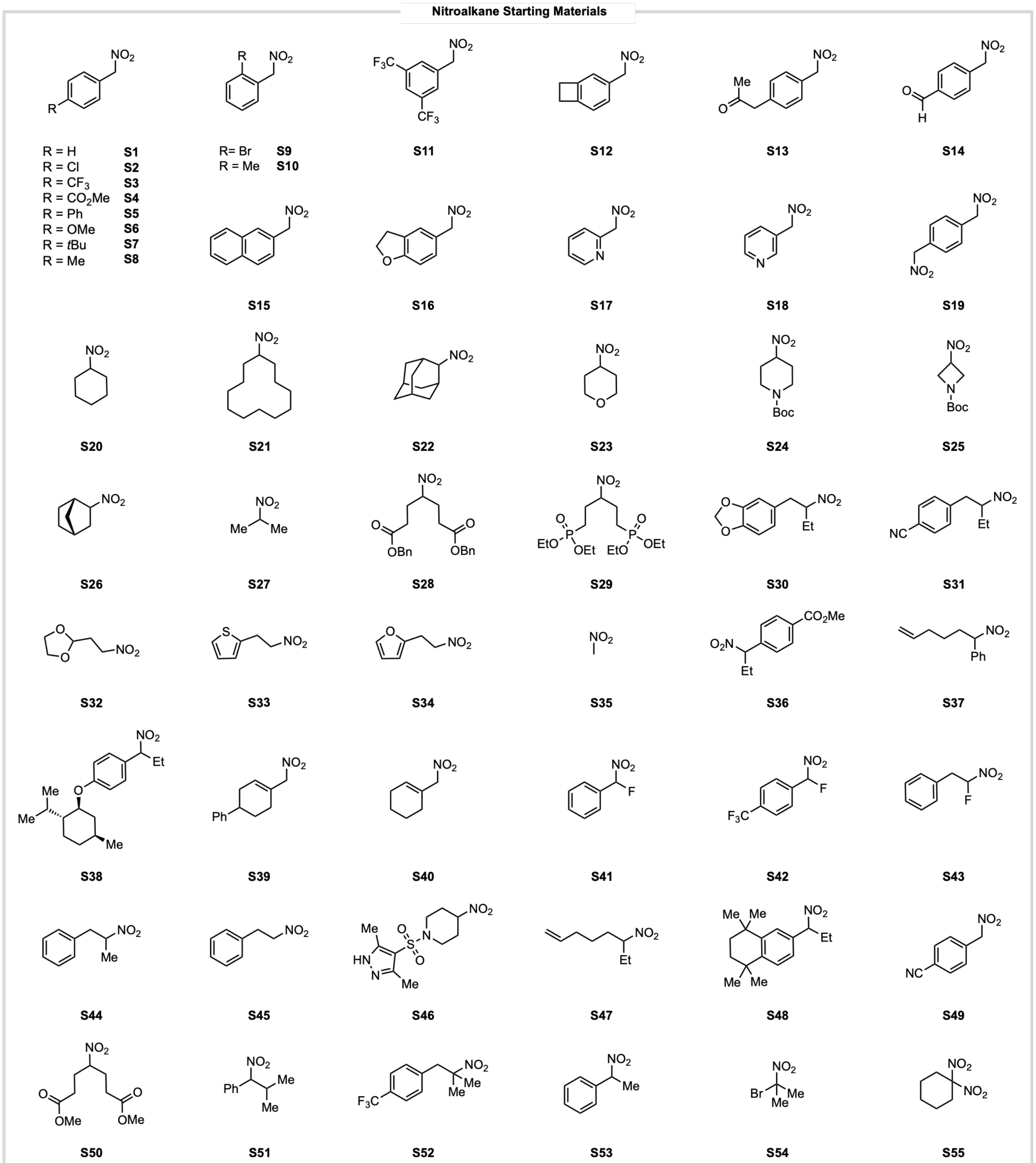

General Procedures

General Procedure A. Conditions for the Photo-stitching of Nitroalkanes.

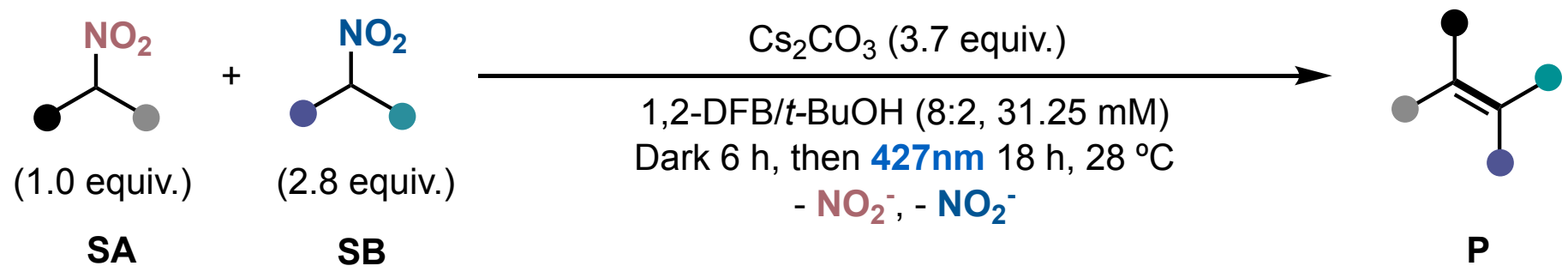

Reaction setup. Except when otherwise stated, in a flame-dried 4-dram vial equipped with a stir-bar, was charged nitroalkane **SA** (0.45 mmol, 1.0 equiv.), nitroalkane **SB** (2.8 equiv.), Cs_2CO_3 (3.7 equiv.) and dry and degassed 1,2-DFB/*t*-BuOH (14.3 mL, 31.25 mM) under N_2 or Ar. The reaction vessel was then capped and left to stir vigorously for 6 h, following which it was then irradiated under a 427 nm Kessil lamp with 2-3 cooling fans for 18 h. Due to the heat of the lamp, the reactions run around 28 ˚C. Completion of the reaction was determined by TLC analysis. After the reaction was complete, the mixture was extracted and concentrated down. The crude product was purified by column chromatography (0–5% EtOAc/Hexanes) to afford the alkene products.

Important: The amount of base must be less than the total amount of nitroalkane added. 1,2-Difluorobenzene was dried over CaH_2*, distilled under an inert atmosphere, and degassed by three freeze–pump–thaw cycles prior to use. Commercially available, degassed tert-butanol was used.*

Caution: Although nitronates are generated in situ in this procedure and no safety incidents were encountered, care should be taken during workup. Aqueous layers should be neutralized slowly to quench any residual nitronate species.

General Procedure B. Gram-scale Conditions for the Photo-stitching of Nitroalkanes.

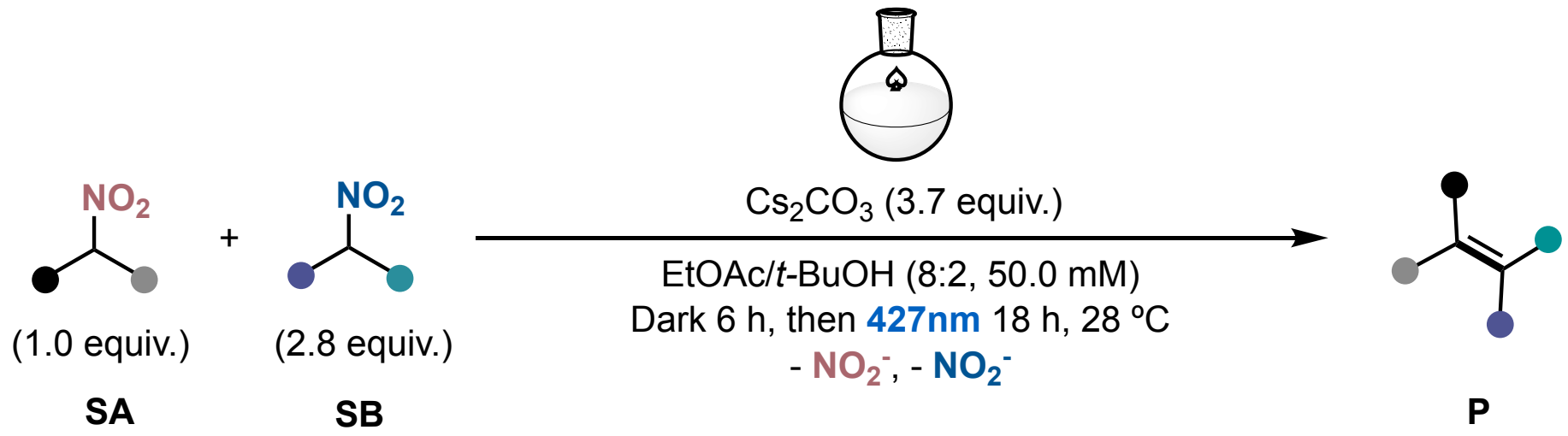

In a flame-dried 250 mL round-bottom flask equipped with a stir-bar, was charged 1-chloro-4-(nitromethyl)benzene (**S2**, 4.66 mmol, 1.0 equiv.), *tert-butyl* 4-nitropiperidine-1-carboxylate (**S24**, 2.8 equiv.), Cs_2CO_3 (3.7 equiv.) and EtOAc/*t*-BuOH (93.3 mL, 50.0 mM) under N_2. The reaction vessel was then capped with a septum and left to stir at 600 rpm for 6 h. The reaction was then irradiated under two 427 nm Kessil lamps with three cooling fans for 18 h. Completion of the reaction was determined by GC–MS analysis. The mixture was then extracted with ethyl acetate and concentrated down. The crude product was purified by column chromatography to afford the alkene product **P21** in 70% yield as a white solid.

Important: The amount of base must be less than the total amount of nitroalkane added. Commercially available, degassed EtOAc and tert-butanol was used.

Caution: Although nitronates are generated in situ in this procedure and no safety incidents were encountered, care should be taken during workup. Aqueous layers should be neutralized slowly to quench any residual nitronate species.

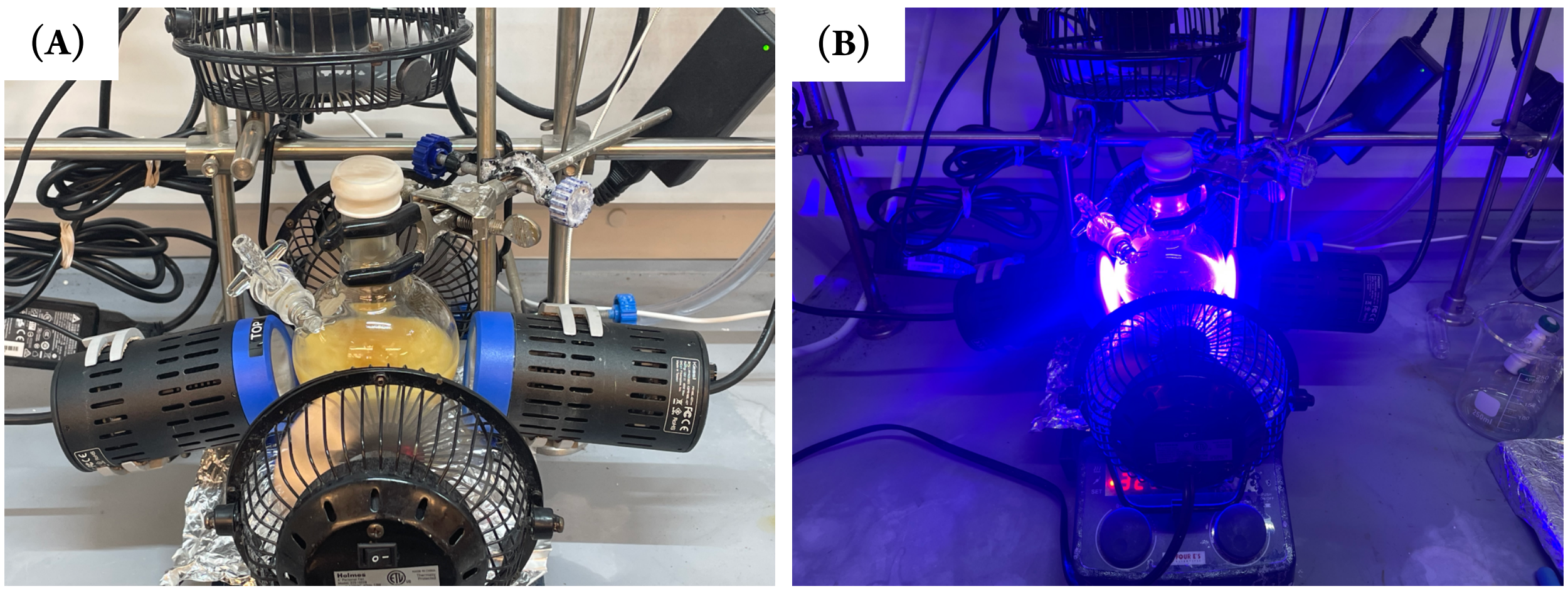

Fig. S0. Images of Scaleup Procedure, without (A) and with (B) Kessil lamps on.

General Procedure C. Synthesis of Nitroalkanes from Aryl Halides

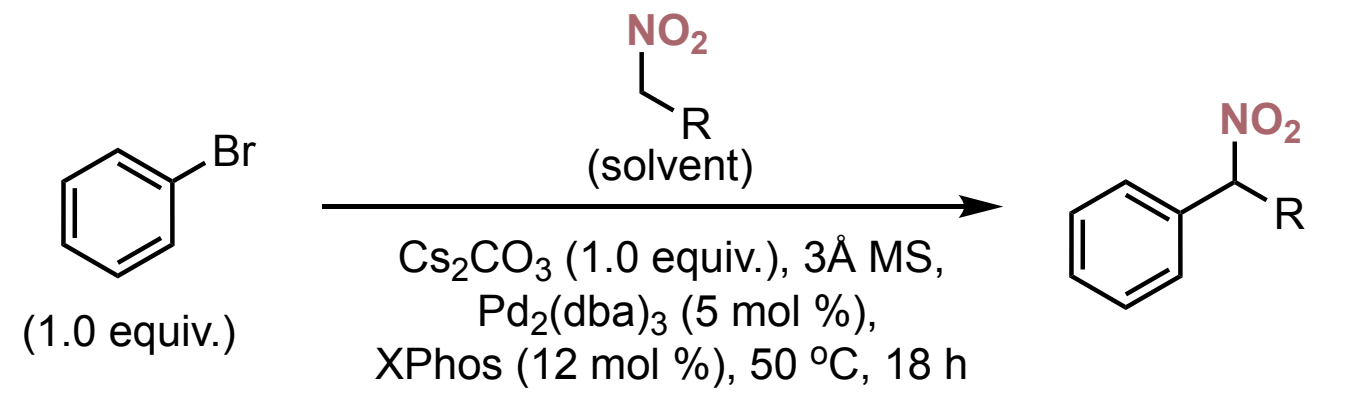

In a glovebox, an oven-dried vial was charged with cesium carbonate (1.1 equiv., 5.500 mmol), powdered 3 Å molecular sieves (100 mg, 200 mg/mmol), Tris((1*E*,4*E*)-1,5-diphenylpenta-1,4-dien-3-one) dipalladium (0.05 equiv.), and 2-(Dicyclohexylphosphanyl)-2',4',6'-tris(isopropyl)biphenyl (0.12 equiv.), nitromethane (50.00 mL) was added, followed by the addition of the aryl halide (1.0 equiv.). The vial was capped, removed from the glovebox, and heated to 50 °C in an IKA heating plate with vigorous stirring. Upon consumption of the aryl halide, as monitored by TLC or GC, the reaction mixture was allowed to cool to rt, diluted with CH_2Cl_2 (50 mL), and washed with saturated aq NH_4Cl (2 x 50 mL). The aqueous layer was extracted with CH_2Cl_2 (2 x 50 mL). The combined organic layers were washed with brine, dried over Na_2SO_4, filtered, and concentrated in vacuo. The product was then isolated via flash column chromatography.

General Procedure D. Synthesis of Nitroalkanes from Benzyl Halides

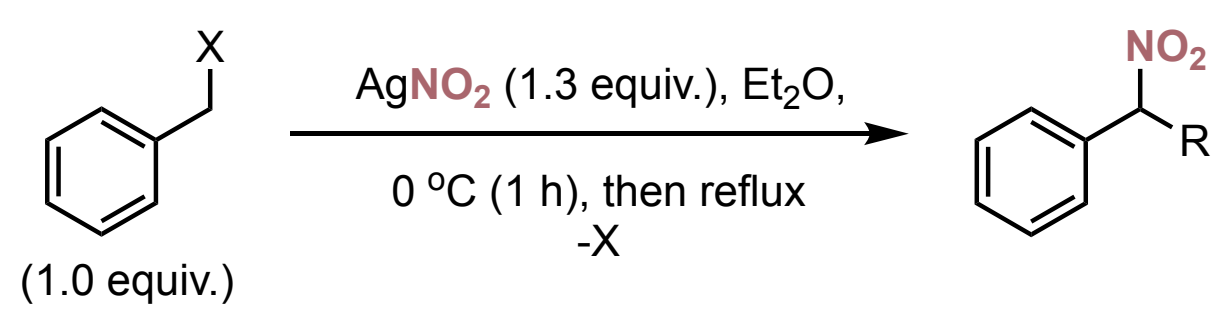

Silver nitrite (763 mg, 1.3 equiv., 4.96 mmol) was added to a round-bottom flask covered in tin foil containing anhydrous diethyl ether (10.0 mL). After stirring at room temperature for 15 minutes, the mixture was then cooled to 0 °C. A solution of benzyl halide (1.0 equiv.) in diethyl ether (1.7 mL) was added dropwise via an addition funnel. The reaction was stirred at 0 °C for 1 h and then heated to reflux for 4 h. The mixture was filtered over Celite using ethyl acetate as eluent. The product was then isolated via flash column chromatography.

General Procedure E. Synthesis of Nitroalkanes from the Reduction of Nitroalkenes

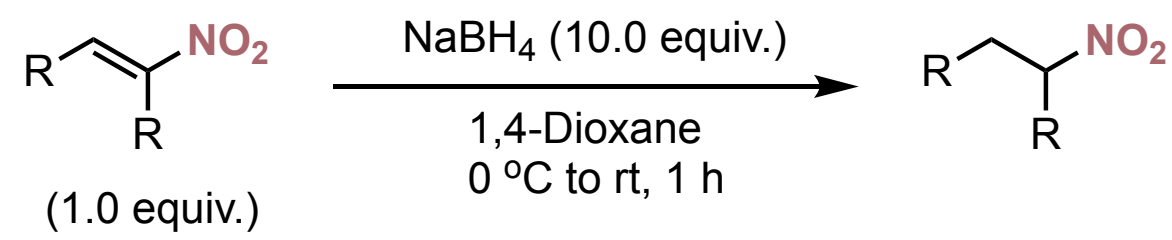

To a solution of nitroalkene (1.0 equiv., 1.45 mmol) in 1,4-dioxane (7.23 mL) in an ice bath was added sodium tetrahydroborate (10 equiv., 14.5 mmol) slowly. After addition, the mixture was allowed to stir at room temperature for 1 h and then concentrated. Ethyl acetate and a saturated solution of NH_4Cl were added to the residue obtained, and the organic layer was separated and washed with brine, dried over Na_2SO_4, and concentrated. The product was then isolated via flash column chromatography.

Optimization Studies

Table S1. Solvent screen for cross-stitching activated/unactivated nitroalkanes.

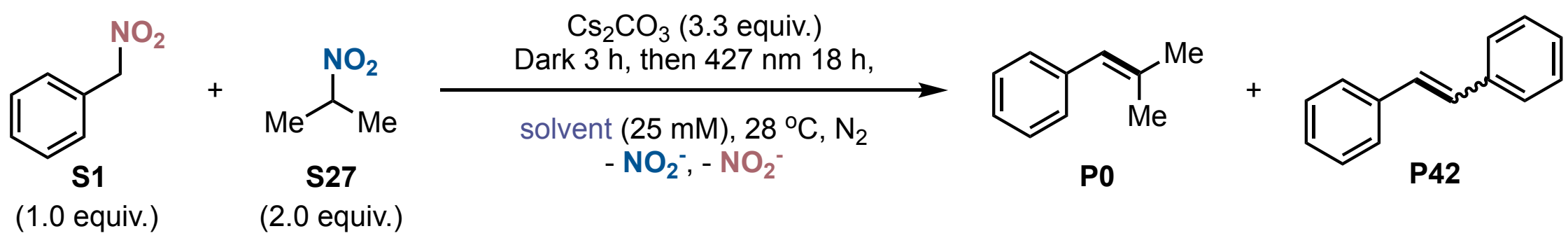

Entry	*Solvent*	*Yield (**P0**)* [a]	*Yield (**P42**)* [a, b]
1	THF	6%	79%
2	$PhCF_3$	1%	32%
3	DCE	10%	34%
4	1,2-DFB	33%	39%
5	*t*-BuOH	14%	10%
6	EtOH	20%	24%
7	MeCN	8%	56%
8	**1,2-DFB/*t*-BuOH (9:1)**	**42%**	**10%**

Reactions were performed on a 0.1 mmol scale. [a] Denotes ^1H NMR yields using CH_2Br_2 as an internal standard. [b] Includes both *E*/*Z* products.

Table S2. Time, equivalence, and additional solvents screen.

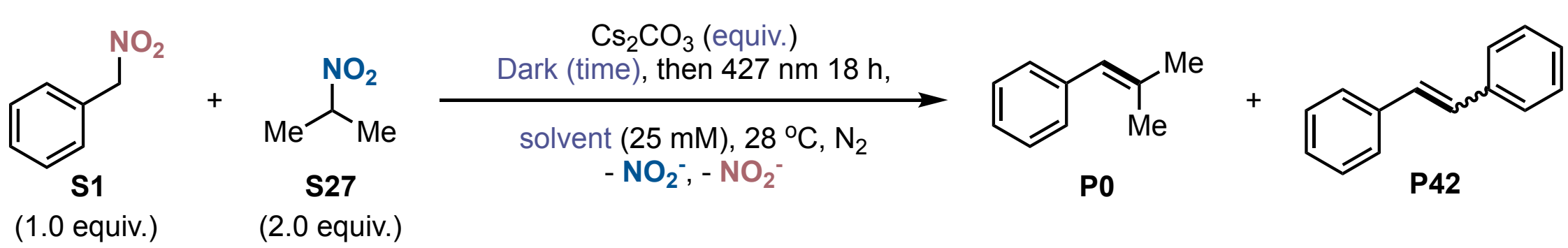

Entry	*Solvent*	*Base (equiv.)*	*Time in Dark (h)*	*Yield (**P0**)* [a]	*Yield (**P42**)* [a, b]
1	1,2-DFB	3.3	1	33%	67%
2	1,2-DFB	3.3	3	33%	39%
3	1,2-DFB/*t*-BuOH (9:1)	3.3	3	42%	10%
4	1,2-DFB/*t*-BuOH (9:1)	3.3	6	19%	10%
5	1,2-DFB/*t*-BuOH (9:1)	2.8	3	55%	8%
6	1,2-DFB/*t*-BuOH (9:1)	2.8	3	61% [c]	18% [c]
7	**1,2-DFB/*t*-BuOH (4:1)**	**2.8**	**6**	**70%**	**6%**

Reactions were performed on a 0.1 mmol scale. [a] Denotes ^1H NMR yields using CH_2Br_2 as an internal standard. [b] Includes both *E*/*Z* products. [c] 37.5 mM concentration.

Table S3. Additional solvent screen for cross-stitching activated/unactivated nitroalkanes.

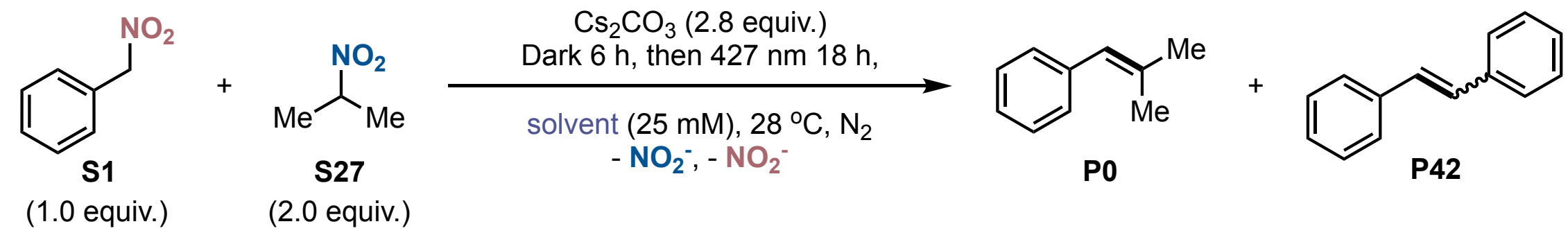

Entry	Solvent	Yield (**P0**)[a]	Yield (**P42**)[a, b]
1	**1,2-DFB/*t*-BuOH (4:1)**	**70%**	**6%**
2	1,2-DFB/DCE/*t*-BuOH (7:1:2)	67%	11%
3	DCE/*t*-BuOH (4:1)	51%	7%
4	1,2-DFB/*t*-BuOH (8.5:1.5)	51%	6%
5	1,2-DFB/EtOAc/*t*-BuOH (7:1:2)	71%	11%
6	1,2-DFB/EtOAc (4:1)	56%	12%
7	EtOAc/*t*-BuOH (4:1)	61%	8%

Reactions were performed on a 0.1 mmol scale. [a] Denotes ^{1}H NMR yields using CH_2Br_2 as an internal standard. [b] Includes both *E*/*Z* products.

Table S4. Control experiments.

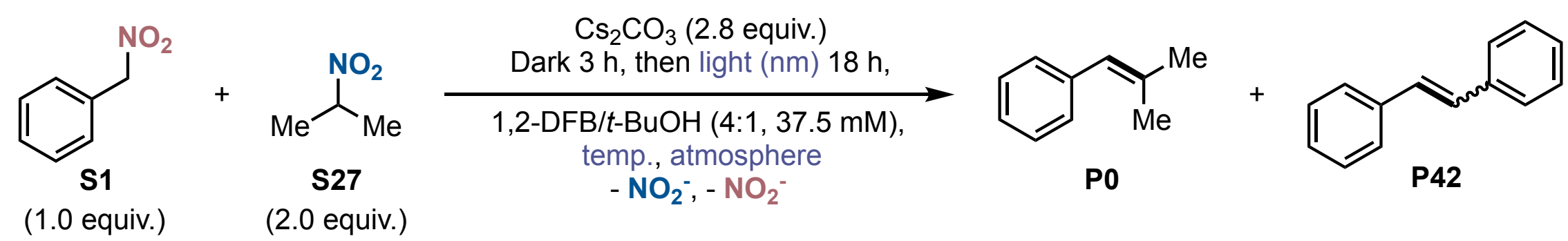

Entry	Temp. (°C)	Atmosphere	Additive	Light	Yield (**P0**)[a]	Yield (**P42**)[a,b]
1	28	N_2	--	390 nm	23%	9%
2	**28**	N_2	**--**	**427 nm**	**61%**	**18%**
3	28	N_2	--	440 nm	11%	11%
4	40	N_2	--	--	ND	ND
5	92	N_2	--	--	ND	4%
6	28	Air	--	427 nm	11%	38%
7	28	N_2	TEMPO[c]	427 nm	0%	0%
8	28	N_2	1,4-DNB[d]	427 nm	0%	0%

Reactions were performed on a 0.1 mmol scale. [a] Denotes ^{1}H NMR yields using CH_2Br_2 as an internal standard. [b] Includes both *E*/*Z* products. ND = not detected. 1,4-DNB = 1,4-Dinitrobenzene. TEMPO = (2,2,6,6-Tetramethylpiperidin-1-yl)oxyl. [c] 2.8 equiv. [d] 0.2 equiv.

Table S5. Base and concentration screen.

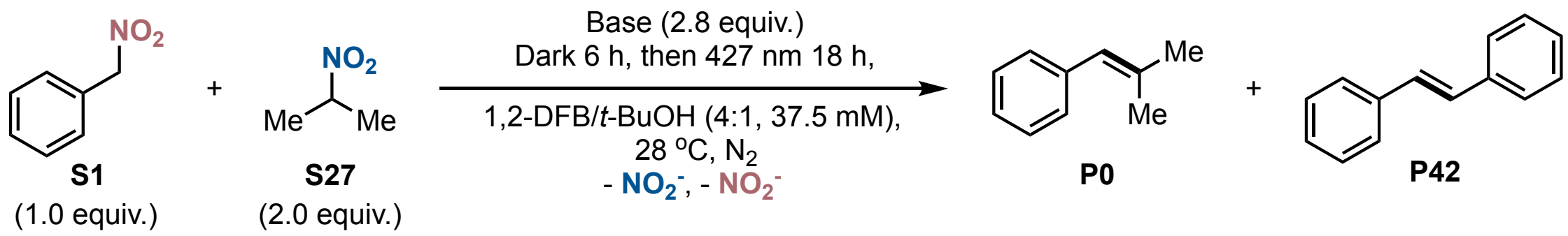

Entry	*Nitroalkane*	*Base*	*Conc. (mM)*	*Yield (**P0**)* [a]	*Yield (**P42**)* [a, b]
1	**S1** + **S27**	K_2CO_3	25	ND	100%
2	**S1** + **S27**	Cs_2CO_3	25	70%	6%
3	**S1** + **S27**	DBU	25	12%	87%
4	**S1** + **S27**	Cs_2CO_3	37.5	60%	18%
5	**S1 + S27**	**Cs_2CO_3**	**31.25**	**91%**	**6%**
6	**S1** + **S20**	Cs_2CO_3	31.25	80% (of **P1**) [c]	7% [c]
7	**S1 + S20**	**Cs_2CO_3**	**31.25**	**90% (80%) [d] (of P1) [c, e]**	**6% [c]**

Reactions were performed on a 0.1 mmol scale. [a] Denotes ^1H NMR yields using CH_2Br_2 as an internal standard. [b] Includes both *E*/*Z* products. [c] Due to volatility of compound **P0**, the synthesis leading to **P1** was used as a new benchmark to confirm reproducibility at a 0.45 mmol scale. [d] Isolated yield. [e] Using 2.8 equiv. of unactivated nitroalkane and 3.7 equiv. of base ensured consistently high yields across different reactions. ND = not detected.

Possible Reaction Pathways

List of Possible Reaction Pathways

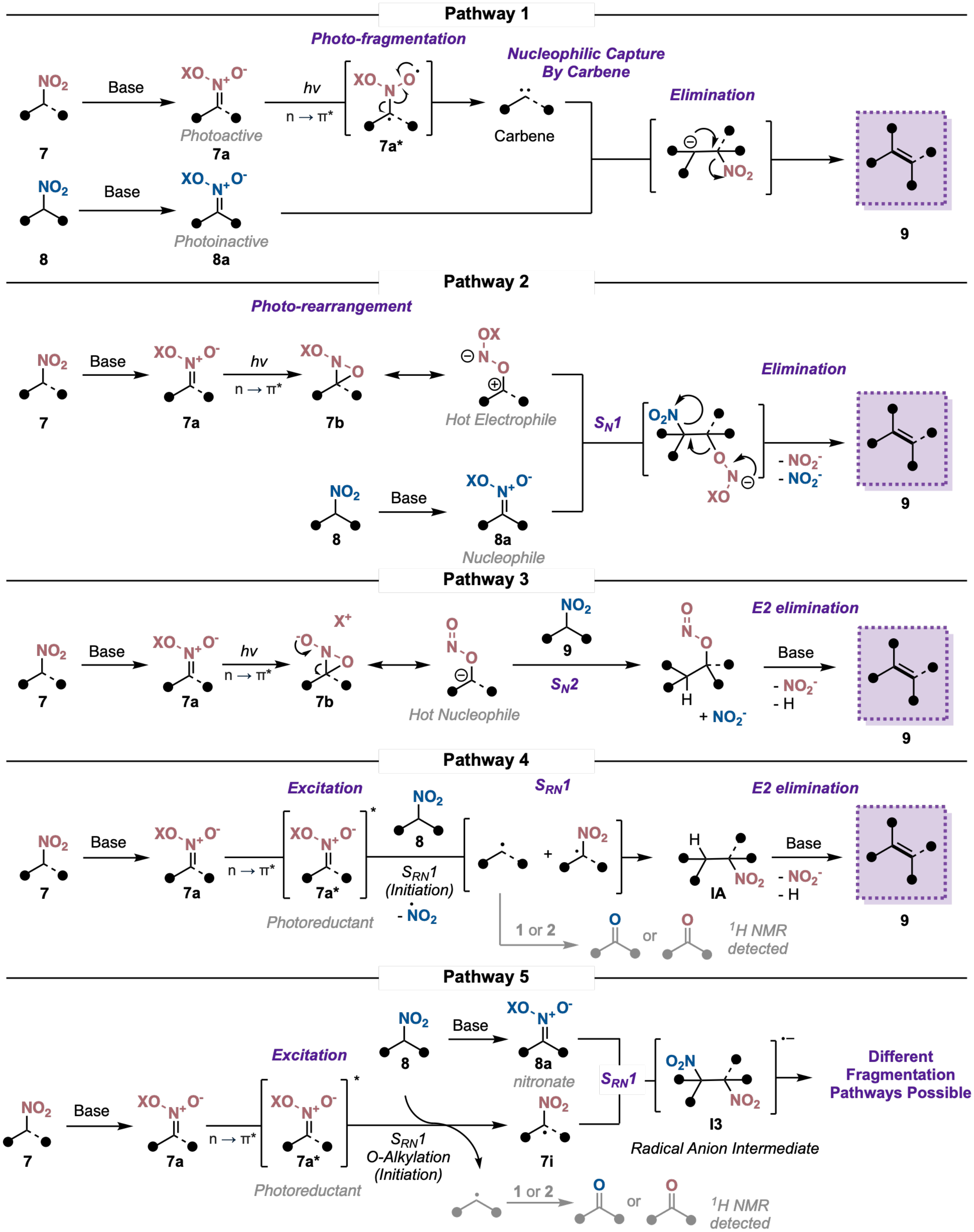

Fragmentation Pathways From the Radical Anion Intermediate of *Pathway 5*

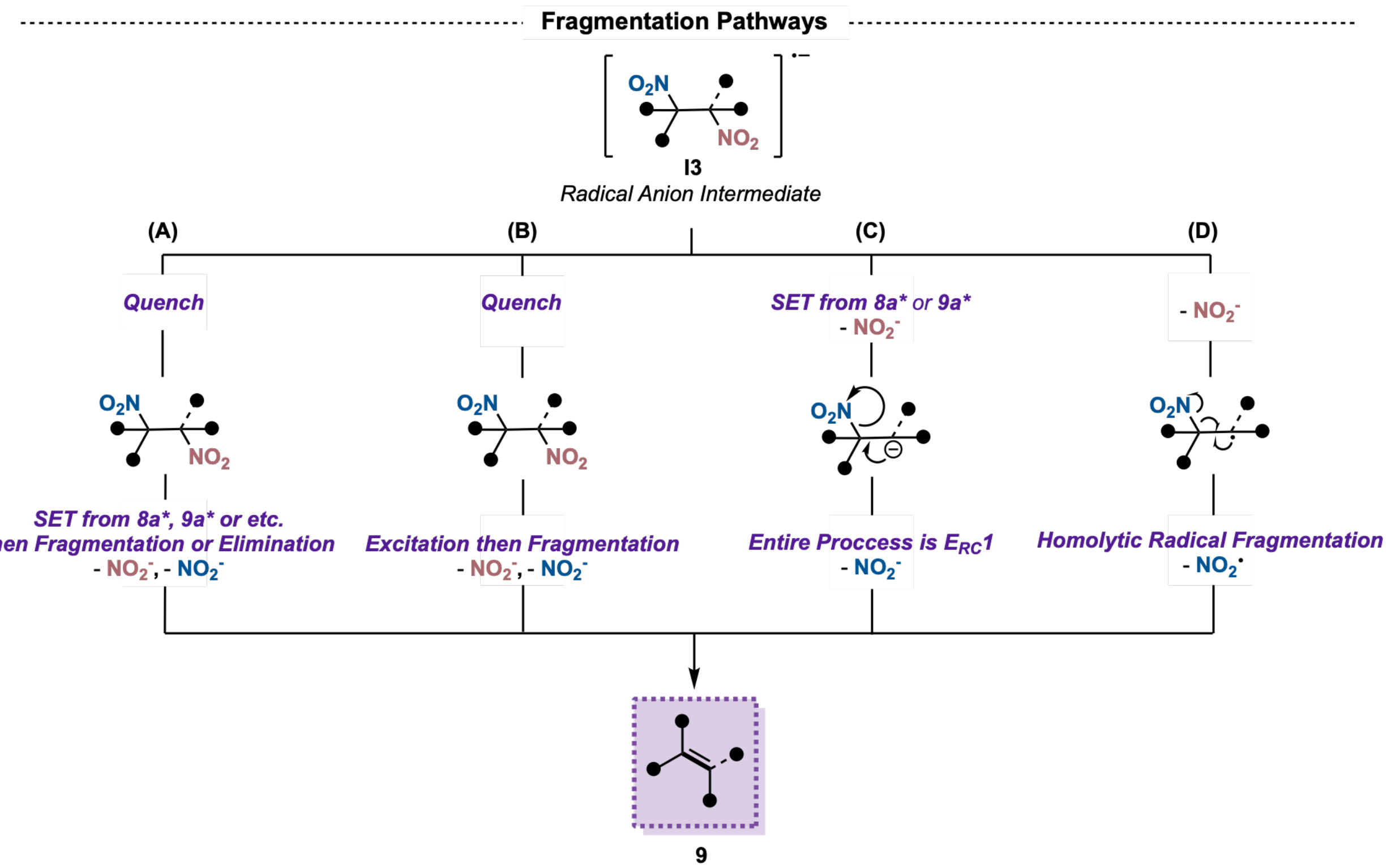

Reasoning for Each Reaction Pathway

Reasoning for ***Pathway 1****.* Based on the control studies and prior literature[1, 2] we considered whether photoexcited nitronates generate carbene intermediates, which could undergo nucleophilic capture of in situ-generated nitronates and thus result in anionic species that can subsequently undergo elimination to afford the observed products.

Reasoning for ***Pathways 2-3****.* Given literature precedent for nitronates to photorearrange to hydroxamic acids via 1,2-oxaziridin-2-olate intermediates[3], we considered whether, under the reaction conditions, we are generating photoexcited nitronates that form 1,2-oxaziridin-2-olates intermediates capable of acting as either hot electrophiles or hot nucleophiles due to the electronic influence of the adjacent substituents. While forming a formal alkyl nitrite is unlikely based on prior literature, we didn't dispute the possibility in its entirety.

Pathway 3 is unlikely based on 1) the electronics on the carbon that would be a part of the oxaziridine core, which drives the rearrangement, and 2) the observed regioselectivity of olefin formation. In regard to regioselectivity, competing base abstraction at alternative β-hydrogen sites would be expected to lead to E2 elimination pathways that would result in alkenes at different sites, which are not observed experimentally. However, probing the possibility is still warranted due to the ease of alkyl nitrites being generated in the synthesis of nitroalkanes.

Reasoning for ***Pathway 4****.* Nitronates bearing electron-accepting partners such as Katritzky salts are known to undergo $S_{RN}1$ reactions under photochemical conditions, furnishing coupled products via non-chain processes[4]. Prior studies also demonstrate that E2 elimination can be operative when acidic β-hydrogen sites are present[5]. We therefore considered whether a classical $S_{RN}1$ coupling followed by base-mediated β-hydrogen abstraction could furnish the observed alkene products. However, this pathway would also be expected to exhibit regioselectivity issues arising from competing β-hydrogen sites, which are not observed in our system.

Reasoning For ***Pathway 5****.* Based on prior literature and the observed reaction scope, we considered whether an $S_{RN}1$ pathway operates to generate radical-anion intermediates. These intermediates could either be quenched to form vicinal dinitroalkanes, which subsequently undergo reduction under photoreducing conditions via nitronates (Fragmentation Pathway A) or photodecomposition (Fragmentation Pathway B)[6], undergo radical-chain elimination (Fragmentation Pathway C)[7], homolytic radical fragmentation (Fragmentation Pathway D)[8], or proceed through a combination of these pathways.

Mechanistic Studies

UV–vis

Reasoning. To identify the photoabsorbing components, UV–vis absorption studies were performed. Spectra were collected on either a Cary 100 UV–vis spectrophotometer using the following parameters: data interval, 1.0 nm; bandwidth, 1.0 nm; response time, 0.1 s; scan rate, 600 nm min^{-1}; and baseline correction at ambient temperature, or on a Cary 3500 UV–vis spectrophotometer using the following parameters: data interval, 1.0 nm; bandwidth, 2.0 nm; scan rate, 3000 nm min^{-1}; baseline correction and using a compact Peltier UV–vis module without stirring at ambient temperature. Spectra were recorded in two-window quartz cuvettes (3.5 mL volume; outer dimensions 45 × 12.5 × 12.5 mm; path length 10 mm; window width 10 mm) equipped with Teflon stoppers (ES quartz glass, 190–2500 nm). Individual absorption spectra are shown in **Fig. S1** and **Fig. S2**.

Reaction Setup. To simulate the reaction conditions, nitroalkanes and, when applicable, the minimum corresponding amount of Cs_2CO_3 (1.02:1 nitroalkane to base) were added to a cuvette containing 1,2-DFB and *t*-BuOH (4:1, v/v, 31.25 mM) under N_2 and sealed. For samples containing base, a micro stir bar was added, and the mixture was stirred in the dark for 6 h to generate the corresponding nitronate. Owing to the limited solubility of the base, cuvettes containing Cs_2CO_3 were subsequently centrifuged using a Labconco Centrivap™ concentrator for 15 min. The cuvettes were then inspected to confirm that any insoluble particulates had settled and that the supernatant solution was visibly clear. This procedure was confirmed using solvent blanks with and without base, with no distortion detected.

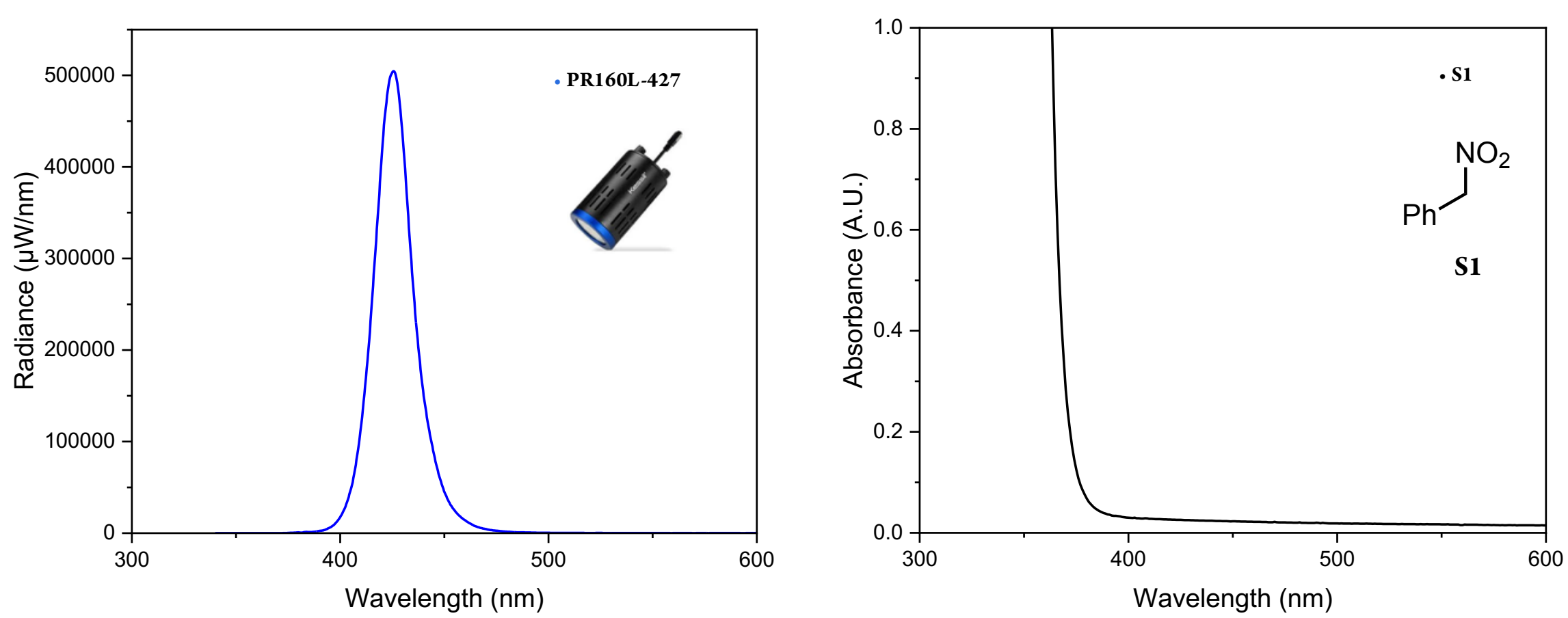

Fig. S1. (Left) Spectral radiance (µW nm^{-1}) versus wavelength for the PR160L-427 Kessil lamps used to irradiate the reaction mixtures. (Right) UV–vis absorption spectrum of (nitromethyl)benzene (**S1**).

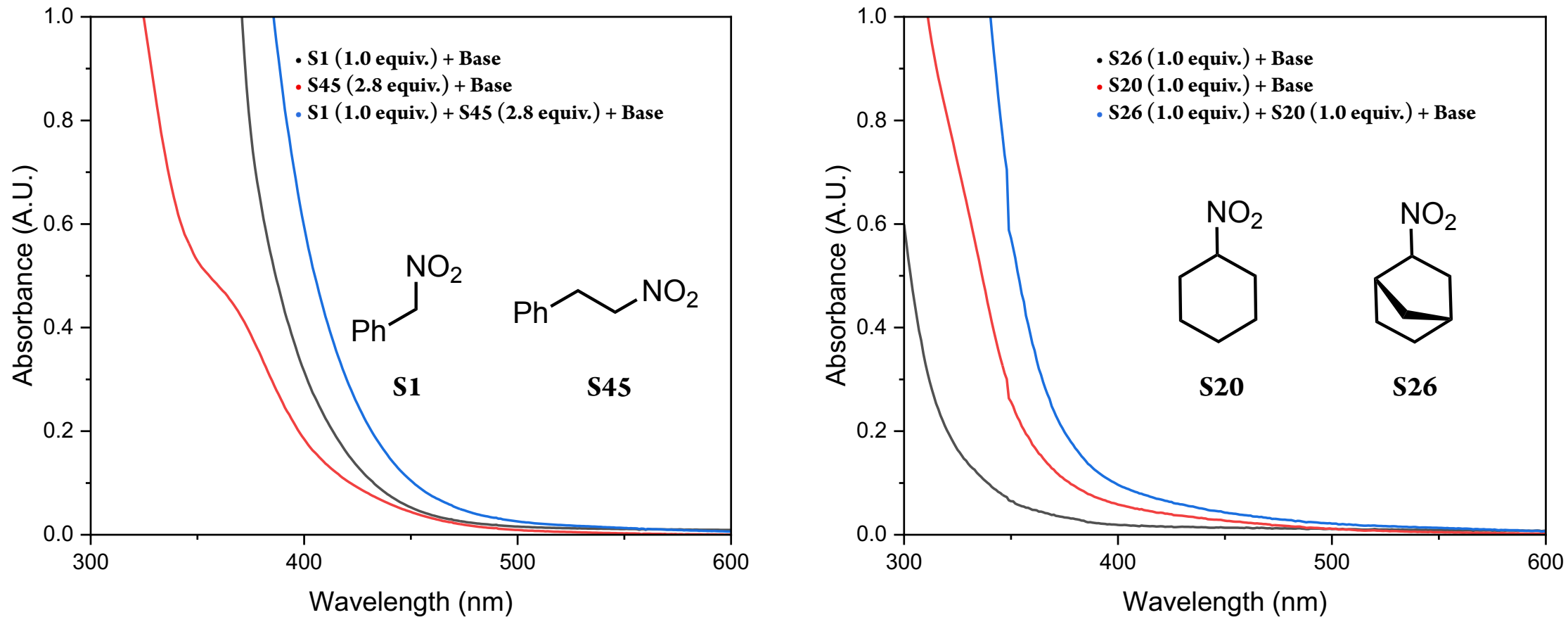

Fig. S2. UV–vis absorption spectra of (Left) activated (0.100 mmol) and unactivated (0.28 mmol) nitroalkanes and (Right) unactivated/unactivated nitroalkanes (0.100 mmol each) under basic conditions (1.02:1, nitroalkane to Cs_2CO_3) in 1,2-DFB/*t*-BuOH (4:1, v/v). A centrifuged base-containing solution was used as the background.

Results. Nitroalkanes do not absorb within the irradiation window of the 427 nm Kessil lamps (390–470 nm) (**Fig. S1**). In the presence of base, however, formation of the corresponding nitronates induces a bathochromic shift that extends absorption into this window (**Fig. S2**), indicating that nitronates are the primary photoabsorbing species under the reaction conditions. This behavior is observed even for unactivated nitroalkanes such as **S20**. In contrast, nitroalkanes such as **S26** do not exhibit appreciable absorption within the irradiation window, which is consistent with their inability to undergo homo-dimerization, despite being used in excess in the scope investigations leading to products **P24** and **P53**.

Cyclic Voltammetry

Reasoning. Cyclic voltammetry studies were conducted to determine whether the nitronates could reduce the nitroalkanes, either in its ground state or excited state.

Reaction Setup. Cyclic voltammetry was performed on a BioLogic SP-50e potentiostat using a glassy carbon working electrode, Pt wire counter electrode, and saturated Ag/AgCl (NaCl) reference electrode. Measurements were conducted at room temperature in anhydrous, degassed MeCN/DMF (9:1, v/v) with tetrabutylammonium hexafluorophosphate (0.1 M) and substrate (1 mM) at a scan rate of 100 mV s^{-1}.

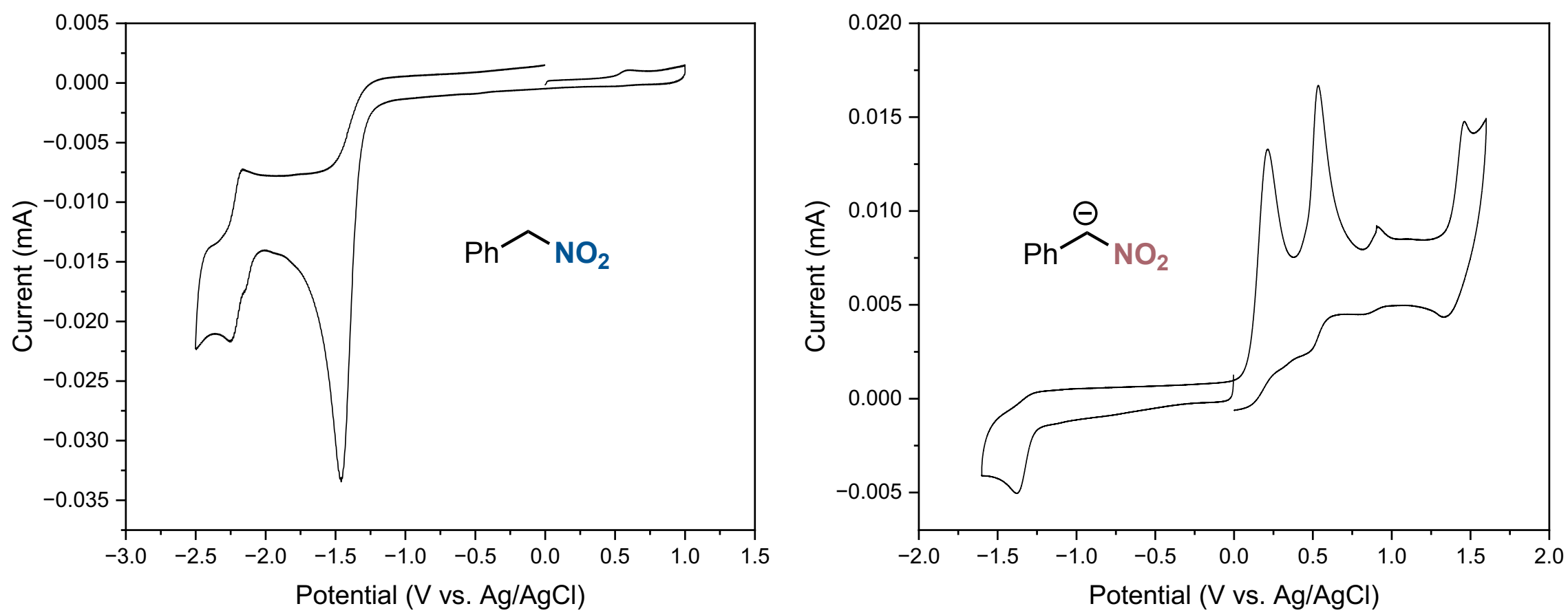

Fig S3. Cyclic voltammograms of (A) (nitromethyl)benzene and (B) sodium (nitromethyl)benzene nitronate.

Results. Cyclic voltammetry measurements were used to determine the ground-state redox potentials of (nitromethyl)benzene and its corresponding sodium nitronate. As the reduction of (nitromethyl)benzene was electrochemically irreversible, a formal $E_{1/2}$ could not be defined. Instead, potentials were estimated as the value at which the cathodic current equals one-half of the peak current defined as $E_{p/2} = E\left(i = i_p/2\right)$, which is measured on the forward scan at the indicated scan rate. The reduction potential of (nitromethyl)benzene (A) was determined as:

$$E_{p/2,red}(A/A^-) = -1.3924 \text{ V (vs. Ag/AgCl)}$$

Two oxidation events were observed for the sodium (nitromethyl)benzene nitronate (M):

$$E_{p/2,ox,1}(M/M^+) = +0.1419 \text{ V (vs. Ag/AgCl)}$$

$$E_{p/2,ox,2}(M/M^{+}) = +0.503 \text{ V (vs. Ag/AgCl)}$$

The zero–zero excitation energy of the nitronate was estimated from the long-wavelength absorption tail of the UV–vis spectrum (λ = 455 nm) (**Fig. S2**)[9]:

$$E_{0-0} = \frac{hc}{\lambda_{\text{tail}}} = \frac{1239.84}{455nm} = 2.72 \text{ eV}$$

The excited-state oxidation potential of the nitronate was calculated using the Rehm–Weller relationship[10]:

$$E_{ox}^{*}(M^{*}/M^{+}) = E_{p/2,ox,1}(M/M^{+}) - E_{0-0}$$

For a one-electron process, the excitation energy expressed in electron volts (eV) is numerically equivalent to volts (V) and can therefore be directly subtracted from the ground-state oxidation potential. Substitution of the experimentally determined values gives:

$$E_{ox}^{*}(M^{*}/M^{+}) = 0.1419\ V - 2.72\ V\ = -2.58 \text{ V (vs. Ag/AgCl)}$$

Rehm–Weller analysis provides the operational framework for estimating the Gibbs free energy change associated with photoinduced electron transfer. Accordingly, the free energy change for electron transfer from the excited nitronate to (nitromethyl)benzene was estimated using the Rehm–Weller expression:

$$\Delta G_{ET}^{\circ} = F[E_{ox}^{*}(M^{*}/M^{+}) - E_{red}(A/A^{-})]$$

$$\Delta G_{ET}^{\circ} = 96.485[-2.58 - (-1.3924)]$$

$$\boxed{\Delta G_{ET}^{\circ} = -114.9 \text{ kJ mol}^{-1} = -1.19 \text{ eV}}$$

This indicates photoexcited nitronate (*M**) to ground-state (nitromethyl)benzene (*A*) is strongly exergonic ($\Delta G° \approx -114.9$ kJ mol^{-1}; −1.19 eV), establishing that in situ-generated nitronates are thermodynamically competent photoreductants under the reaction conditions.

Notes. The sodium salt of the (nitromethyl)benzene nitronate was used instead of the corresponding cesium salt since it was difficult to prepare and isolate in pure form. To verify that the identity of the counter cation did not influence the measured reduction potential, cyclic voltammetry was also performed using cesium hexafluorophosphate (0.1 M) as the supporting electrolyte, which afforded comparable electrochemical behavior.

Computational Calculations

All calculations were performed using the ORCA 6.1.0 software package[11] on the NYU Greene High-Performance Computing (HPC) cluster. A conformation search was carried out using ORCA's GOAT[12, 13, 14] module to identify the global energy minimum for the structure of interest. Geometry optimizations of global minima were conducted at the r^{2}SCAN-3c level of theory[15, 16]. Gibbs free energies values were obtained from frequency calculations performed at the PBE0-D3BJ/6-311+G-(d,p)[17] level of theory using the CPCM solvation model for acetonitrile in accordance with a literature report[18]. Ground and excited state reduction potentials were calculated from free energy differences between redox partners using the Nernst equation. Time-dependent DFT (TD-DFT) calculations were performed as single-point calculations at the ground-state geometries to obtain vertical excitation energies[19]. Molecular structures were visualized using Avogadro (version 2)[20].

Calculations. Ground-state oxidation potentials (E_{ox}) were determined from the Gibbs free energy difference between the anion and radical species. Absolute potentials were converted to the Ag/AgCl (satd. NaCl) scale using the established reference of 4.623 V in acetonitrile. Excited-state redox potentials were derived using the Rehm-Weller equation[10], utilizing TD-DFT for singlet energies and ΔSCF for triplet energies.

The ground state oxidation potential (E_{ox}) was calculated from the Gibbs free energy of the oxidation half-reaction in solution (ΔG_{soln}):

$$M^{-} \rightarrow M^{\cdot} + e^{-}$$

The free energy change is defined as:

$$\Delta G_{soln} = G_{soln}(\text{radical}) - G_{soln}(\text{anion})$$

The absolute oxidation potential (E_{abs}) is then calculated using the Nernst equation (where n = 1 and F is Faraday's constant):

$$E_{abs} = \frac{\Delta G_{soln}}{nF}$$

To convert the absolute potential to the experimental scale (vs. Ag/AgCl; satd. NaCl electrode), the absolute potential of the reference electrode in MeCN (E_{ref} = 4.623 V) was subtracted:

$$E_{ox} = E_{abs} - E_{ref}$$

The excited-state oxidation potentials (E_{ox}^{*}) were calculated using the Rehm-Weller equation:

$$E_{ox}^{*} = E_{ox} - E_{0-0}$$

where E_{0-0} is the vertical excitation energy (in eV) for the respective state (Singlet or Triplet).

Calculated Redox Potentials

Table S6. Calculated redox potentials derived from computational thermochemical parameters, summarizing oxidation half-reactions of photoexcited nitronates and the reduction half-reaction of nitrocyclohexane.

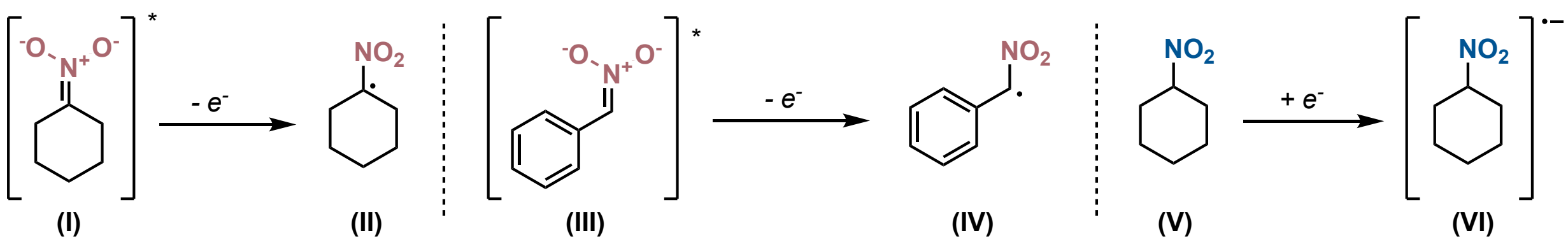

Species	State	State (Q, M)	G_{soln} (Hartree)	$E_{0\text{-}0}$ (eV)	E_{ox} (V vs. Ag/AgCl)	E^*_{ox} (V vs. Ag/AgCl)
(I) to (II)	S_0 (Neutral)	-1 / 1	-439.41895	–	+0.233	–
	D_0 (Radical)	0 / 2	-439.25761	–	–	–
	S_1 (Singlet)	-1 / 1	–	4.333	–	-4.100
	T_1 (Triplet)	-1 / 3	–	2.684	–	**-2.451**
(III) to (IV)	S_0 (Neutral)	-1 / 1	-475.12202	–	+0.0284	–
	D_0 (Radical)	0 / 2	-474.95317	–	–	–
	S_1 (Singlet)	-1 / 1	–	4.095	–	-4.067
	T_1 (Triplet)	-1 / 3	–	2.233	–	**-2.205**
(V) to VI)	S_0 (Neutral)	0 / 1	-439.89609	–	**+1.717**	–
	D_0 (Radical anion)	-1 / 2	-440.00287	–	–	–

Results: These calculations indicate that photoinduced electron transfer from photoexcited nitronates derived from nitrocyclohexane (I) or (nitromethyl)benzene (III) to ground-state nitrocyclohexane (V) is strongly exergonic. This establishes that in situ–generated nitronates are thermodynamically competent photoreductants under the reaction conditions.

For reduction of nitrocyclohexane by photoexcited nitronate (III):

$$\Delta G^{\circ}_{ET} = F[E^{*}_{ox} - E_{red}]$$

$$\Delta G^{\circ}_{ET} = 96.485[-2.205 - (-1.717)]$$

$$\boxed{\Delta G^{\circ}_{ET} = -47.1 \text{ kj mol}^{-1} = -0.488 \text{ eV}}$$

For reduction of nitrocyclohexane by photoexcited nitronate (I):

$$\Delta G^{\circ}_{ET} = F[E^{*}_{ox} - E_{red}]$$

$$\Delta G^{\circ}_{ET} = 96.485[-2.451 - (-1.717)]$$

$$\boxed{\Delta G^{\circ}_{ET} = -70.8 \text{ kj mol}^{-1} = -0.734 \text{ eV}}$$

Note: The computed excited oxidation potentials for photoexcited nitronate (III) differs from the experimentally derived value by approximately 14.5%. This discrepancy arises predominantly from differences in the estimated E_{ox} obtained from computational methods.

Cartesian coordinates

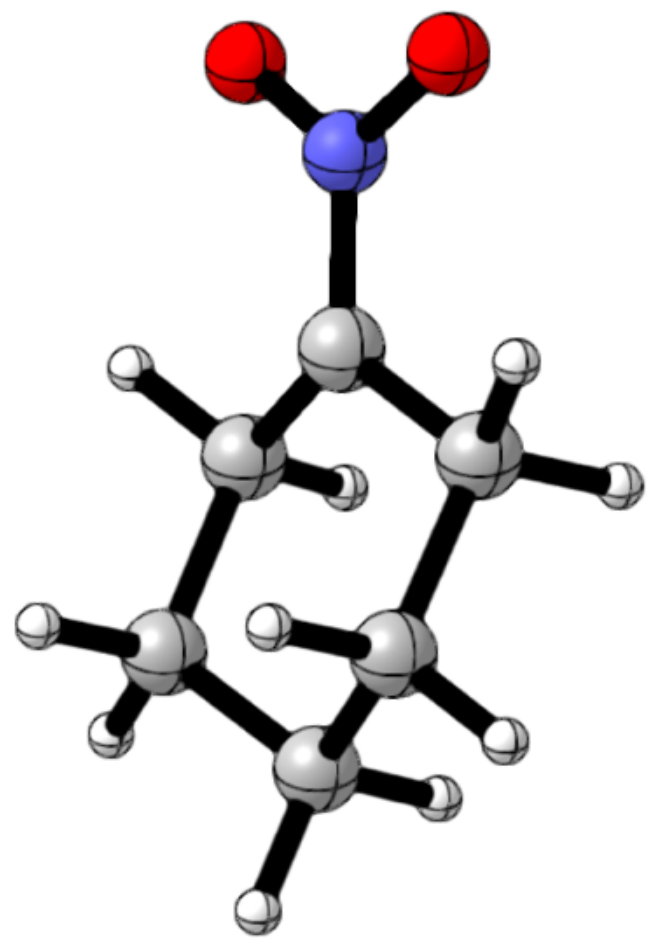

Nitrocyclohexane anion (I)

Electronic energy (E_{el}) = -439.54491385
Zero-point correction = 0.15837640
Thermal correction to Energy = 0.16631386
Thermal correction to Enthalpy = 0.16725806
Thermal correction to Gibbs Free Energy = 0.12596167
Final Gibbs Free Energy = -439.41895219

State	Energy (eV)	λ (nm)	Osc. Strength (f)	Major contributions (%)
S_1	4.333031	286.1	0.127340519	HOMO → LUMO (75.5%), HOMO → LUMO+1 (12.0%)
T_1	2.683547	462.0	0.000000000	HOMO → LUMO (67.4%), HOMO → LUMO + 1 (26.9%)

-1 1
C -4.31875 1.51317 -0.27675
C -4.11659 2.99604 0.04551
H -4.17942 3.13070 1.13564
H -2.54841 4.50733 -0.08687
C -3.21337 0.66753 0.36073
H -2.78490 3.55329 -1.55011
H -5.30949 1.17457 0.06168
H -4.28897 1.37696 -1.37034

C	-1.82303	1.11279	-0.11791
H	-3.26833	0.78314	1.45348
H	-3.37304	-0.39794	0.13508
C	-1.64495	2.59003	0.01648
H	-1.03402	0.60291	0.44257
H	-1.71046	0.78551	-1.17503
C	-2.74560	3.49099	-0.44022
N	-0.41033	3.09664	0.22311
H	-4.92197	3.59538	-0.40596
O	0.57731	2.31331	0.46472
O	-0.21768	4.36504	0.18641

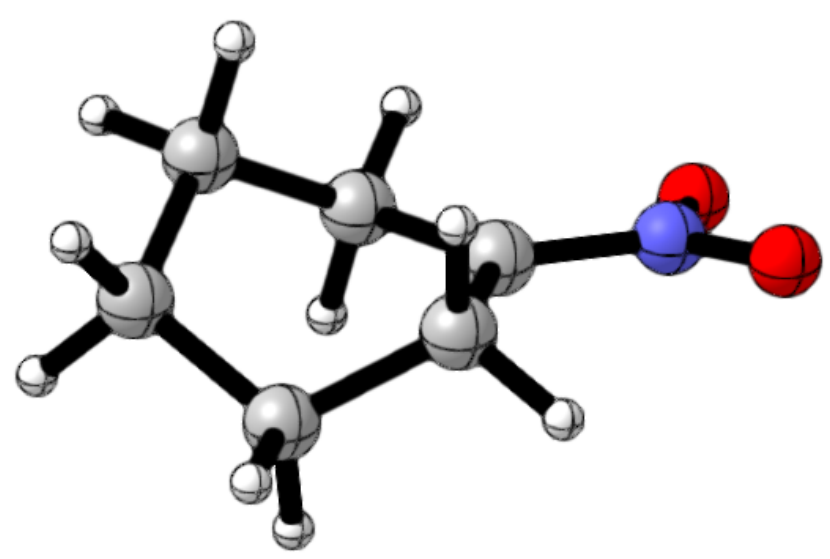

Nitrocyclohexyl radical (II)

Electronic energy (E_{el}) = -439.38286790
Zero-point correction = 0.15868887
Thermal correction to Energy = 0.16693360
Thermal correction to Enthalpy = 0.1678778
Thermal correction to Gibbs Free Energy = 0.12525447
Final Gibbs Free Energy = -439.25761343

0 2

C	-4.28062	1.27322	0.01678
C	-4.14193	2.78138	0.30892
H	-3.97082	2.93639	1.38137
H	-2.88319	4.49683	-0.24323
C	-2.93398	0.61203	-0.34003
H	-3.13429	3.34895	-1.53970
H	-4.71917	0.78163	0.89249
H	-4.98308	1.11409	-0.80909
C	-1.73896	1.30866	0.32559
H	-2.94326	-0.44710	-0.06395

H -2.78031 0.64779 -1.42567
C -1.71788 2.73114 -0.08522
H -1.81908 1.24288 1.42476
H -0.78654 0.83165 0.07364
C -2.97337 3.42584 -0.45014
N -0.48640 3.43181 -0.13082
H -5.07181 3.30497 0.06561
O 0.51500 2.87279 0.34332
O -0.48232 4.56245 -0.64241

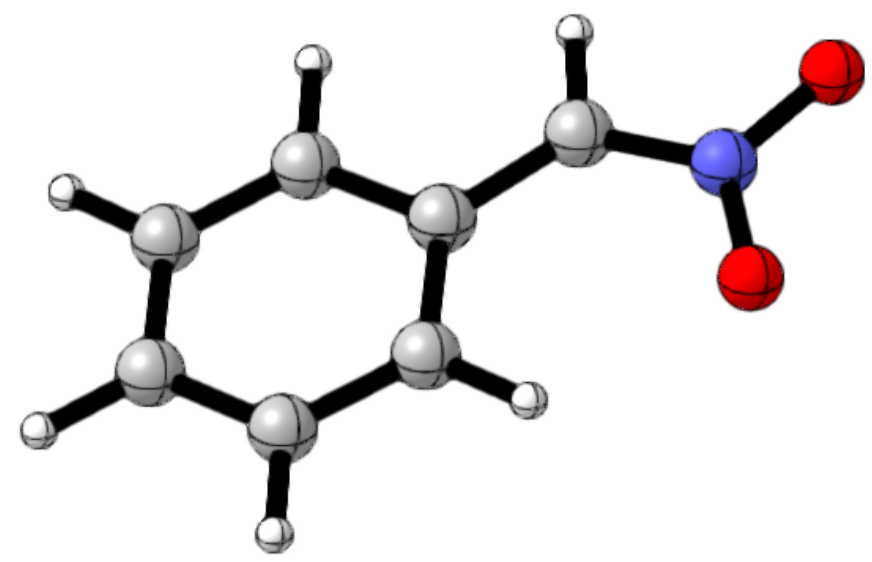

(Nitromethyl)benzene anion (III)

Electronic energy (E_{el}) = -475.20759614
Zero-point correction = 0.11829510
Thermal correction to Energy = 0.12607220
Thermal correction to Enthalpy = 0.12701641
Thermal correction to Gibbs Free Energy = 0.08557730
Final Gibbs Free Energy = -475.12201884

State	Energy (eV)	λ (nm)	Osc. Strength (f)	Major contributions (%)
S_1	4.095	302.8	0.708740077	HOMO → LUMO (60.1%), HOMO → LUMO+1 (32.9%)
T_1	2.233	555.2	0.000000000	HOMO → LUMO (95.3%)

-1 1
H -9.04075 2.25603 -1.54275
C -8.24555 2.17209 -0.80352
C -8.42586 1.36950 0.32733
C -7.37839 1.29238 1.24802
C -6.18754 1.98080 1.06804
C -5.98260 2.80294 -0.07494

C	-7.06508	2.86585	-0.99972
H	-9.35213	0.82334	0.48385
H	-7.49249	0.67438	2.13765
H	-5.38693	1.90916	1.79227
C	-4.81990	3.55911	-0.38337
H	-6.95001	3.48396	-1.88844
N	-3.66987	3.64983	0.34972
O	-3.53533	3.02345	1.44174
H	-4.78942	4.14828	-1.29160
O	-2.73263	4.38298	-0.09474

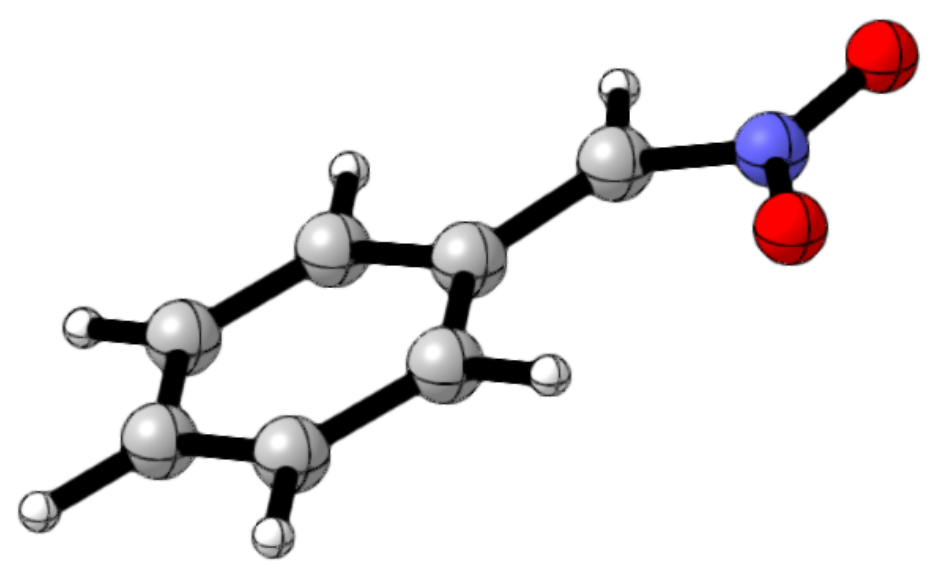

(Nitromethyl)benzyl radical (IV)

Electronic energy (E_{el}) = -475.03837702
Zero-point correction = 0.11872076
Thermal correction to Energy = 0.12663893
Thermal correction to Enthalpy = 0.12758314
Thermal correction to Gibbs Free Energy = 0.08520776
Final Gibbs Free Energy = -474.95316927

0 2

H	-9.04063	2.25648	-1.54539
C	-8.24097	2.18070	-0.81503
C	-8.40156	1.38317	0.32126
C	-7.36831	1.29040	1.25675
C	-6.18106	1.97626	1.08018
C	-5.99874	2.79214	-0.06887
C	-7.06438	2.87349	-1.01043
H	-9.32718	0.83724	0.47606
H	-7.49529	0.67024	2.13903
H	-5.38500	1.90115	1.80704
C	-4.85076	3.54681	-0.38464

H	-6.93954	3.49359	-1.89379
N	-3.65021	3.64587	0.37269
O	-3.53169	3.02909	1.43548
H	-4.80515	4.14175	-1.28878
O	-2.77400	4.37569	-0.11204

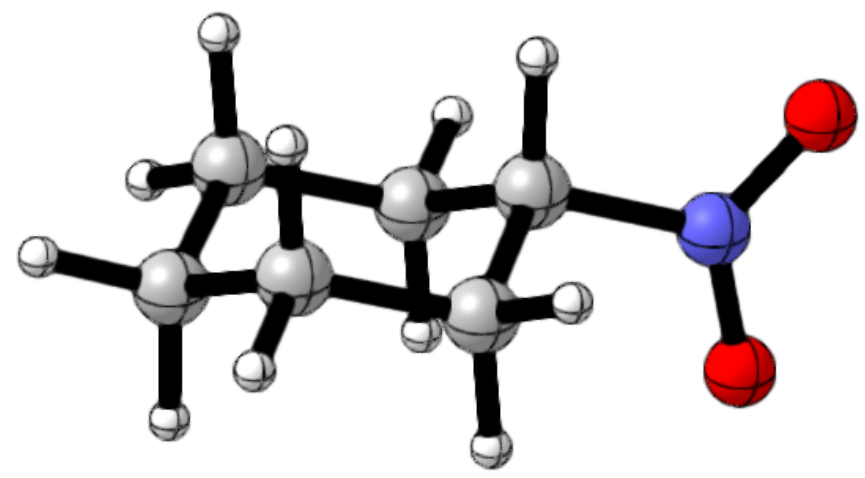

Neutral nitrocyclohexane (V)

Electronic energy (E_{el}) = -440.03649749
Zero-point correction = 0.17294550
Thermal correction to Energy = 0.18097256
Thermal correction to Enthalpy = 0.18191678
Thermal correction to Gibbs Free Energy = 0.14041069
Final Gibbs Free Energy = -439.89608679

0 1

N	1.74083	0.00089	0.01090
O	2.56329	0.00378	0.91571
O	2.00286	-0.00203	-1.18338
C	0.27078	0.00105	0.39746
C	-0.39555	1.26407	-0.14417
C	-1.88065	1.26109	0.23169
C	-2.57886	-0.00124	-0.27866
C	-1.87978	-1.26098	0.23677
C	-0.39473	-1.26450	-0.13921
H	0.27471	0.00321	1.49269
H	0.10574	2.15340	0.25626
H	-0.28196	1.27871	-1.23508
H	-1.98259	1.31878	1.32493
H	-2.36013	2.15829	-0.17626
H	-3.63202	-0.00097	0.02567
H	-2.56426	-0.00341	-1.37786
H	-1.98155	-1.31419	1.33026

H	-2.35871	-2.16020	-0.16736
H	-0.28122	-1.28336	-1.23007
H	0.10721	-2.15189	0.26470

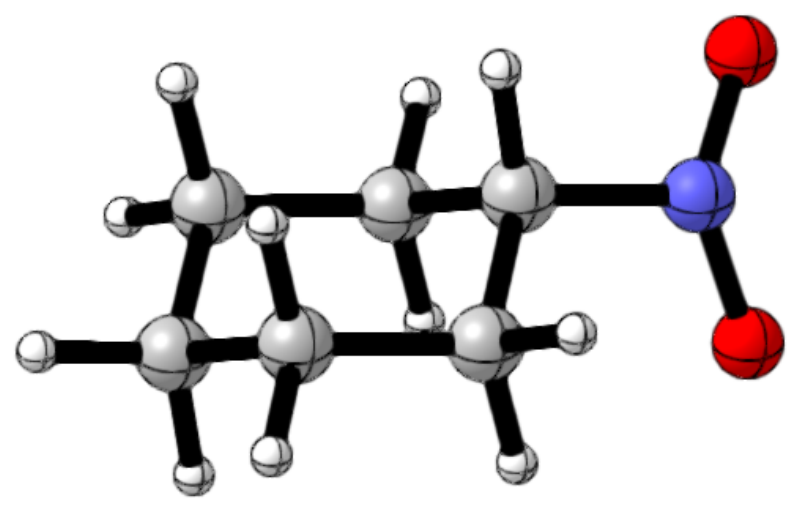

Nitrocyclohexyl radical anion (VI)

Electronic energy (E_{el}) = -440.13964265
Zero-point correction = 0.16996650
Thermal correction to Energy = 0.17809942
Thermal correction to Enthalpy = 0.17904363
Thermal correction to Gibbs Free Energy = 0.13677710
Final Gibbs Free Energy = -440.00286555

0 2

N	1.74762	-0.09806	0.05214
O	2.46232	0.73769	0.76717
O	1.97395	-0.27906	-1.22893
C	0.30465	-0.07914	0.41292
C	-0.35361	1.21239	-0.08658
C	-1.84337	1.25610	0.25736
C	-2.57216	0.02477	-0.29024
C	-1.91431	-1.26484	0.21072
C	-0.42409	-1.29925	-0.14382
H	0.27014	-0.09418	1.51329
H	0.18617	2.06152	0.34900
H	-0.21112	1.25149	-1.17579
H	-1.96520	1.28232	1.35207
H	-2.30869	2.17458	-0.13151
H	-3.63692	0.04861	-0.01471
H	-2.52381	0.04303	-1.38953
H	-2.03601	-1.32347	1.30423
H	-2.42967	-2.14062	-0.20997

H	-0.28192	-1.30008	-1.23187
H	0.04943	-2.21330	0.23905

Light On/Off Studies

Reasoning. Control experiments established that irradiation is required to initiate the reaction. Accordingly, light on/off experiments were conducted using continuous PhotoNMR to determine whether reactivity persists once initiated. Continued stilbene formation or nitronate consumption during dark periods would be consistent with thermally propagated radical chain behavior, whereas suppression of both during dark periods would indicate that continuous irradiation is required beyond initiation.

Reaction setup. (Nitromethyl)benzene (3.43 mg, 0.025 mmol, 1.0 equiv.), Cs_2CO_3 (0.98 equiv.), and DCE-d_4/*t*-BuOD (4:1, v/v; 0.80 mL; 31.25 mM) were added to a reaction vessel under N_2 and stirred for 6 h. Then, the newly formed mixture was transferred to an NMR tube under N_2. Using a PhotoNMR setup, the reaction mixture was irradiated via a fiber-optic cable connected to a 395 nm lamp, and ^{1}H NMR (400 MHz) spectra were acquired every 171 s. Formation of **P42** was monitored by integration of the signal at 7.33 ppm, while nitronate consumption was quantified by integration of the signal at 7.85 ppm (**Fig. S4-S5**). Irradiation was alternately turned off and on at intervals of approximately 21 spectral acquisitions.

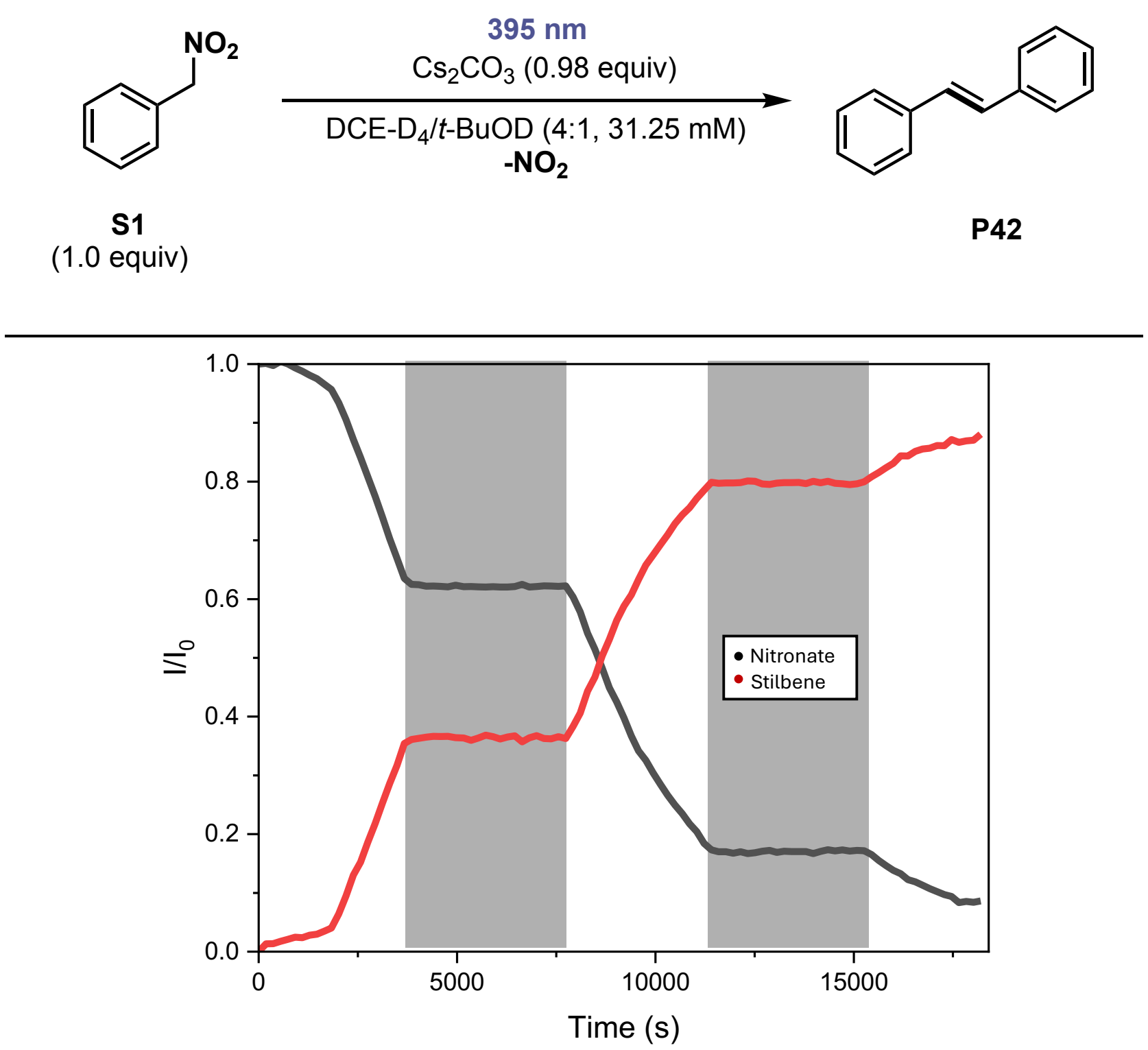

Fig S4. Light on/off study monitored by continuous PhotoNMR showing relative integration versus time for the formation of **P42** (red) and consumption of the cesium nitronate of **S1** during alternating 395 nm irradiation and dark intervals in DCE-d_4/*t*-BuOD (4:1, v/v; 31.25 mM).

^{1}H NMR (400 MHz, $C_2D_4Cl_2$/*t*-BuOD) spectra from Continuous PhotoNMR

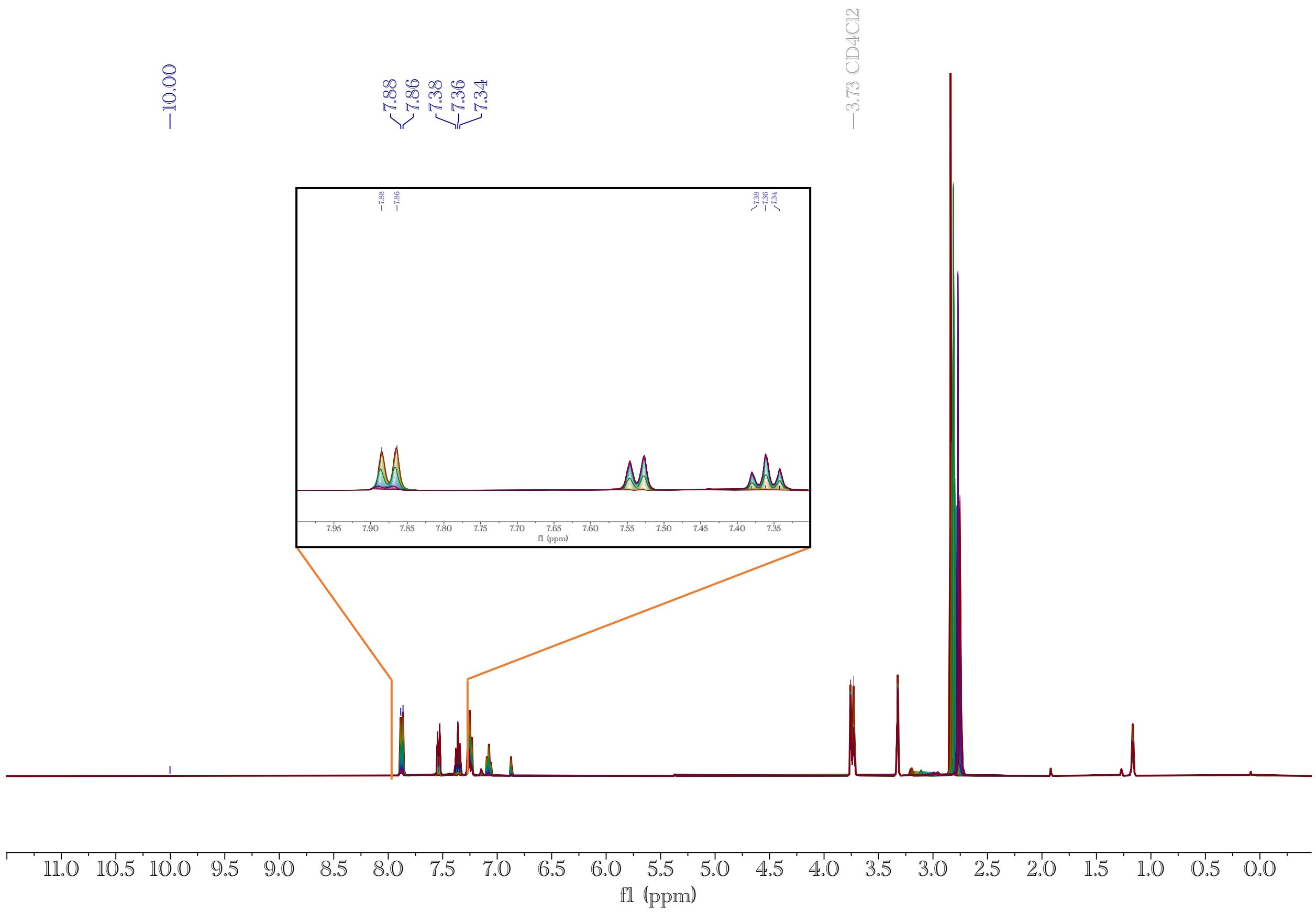

Fig S5. ^{1}H NMR (400 MHz, $C_2D_4Cl_2$/*t*-BuOD) spectra from the continuous PhotoNMR showing **P42** (δ 7.33 (t, *J* = 7.5 Hz, 4H)) and consumption of the cesium nitronate of **S1** (δ 7.85 (d, *J* = 8.3 Hz, 2H)) during alternating 395 nm irradiation and dark intervals in DCE-d_4/t-BuOD (4:1, v/v; 31.25 mM).

Results.

(1) Trace amounts of benzaldehyde (δ 10.00 (s, 1H), **Fig. S5**) (supported by GC–MS) were detected during the initial stages of the reaction concomitant with consumption of the nitronate (δ 7.85 (d, *J* = 8.3 Hz, 2H), **Fig. S5**). Benzaldehyde formation subsequently ceased, coincident with the onset of stilbene growth (δ 7.33 (t, *J* = 7.5 Hz, 4H), **Fig. S5**), thereby suggesting an induction period.

(2) The absence of **P42** formation and the lack of cesium nitronate (**S1**) decay during dark intervals provide evidence against a thermally propagated radical chain mechanism and indicate that continuous irradiation is required for olefin formation.

Assessing Whether Sacrificial Nitroalkane is Required for Reaction Initiation

Reasoning. Cyclic voltammetry studies (**Fig. S3**) indicate that nitronates are unlikely to reduce one another. Consistent with optimization studies showing diminished yields under conditions favoring complete nitronate formation (**Table S2**, **Entry 4**), these results suggest that nitronates alone exhibit little reactivity under the reaction conditions and that an additional, readily reducible substrate is required for efficient product formation. Accordingly, reactions were conducted with nitronates either alone or in the presence of small amounts of the corresponding nitroalkane to assess productive reactivity.

Reaction setup. Following General Procedure A, two reactions were conducted. In the first, the sodium salt of (nitromethyl)benzene (0.100 mmol, 1.0 equiv.) and the sodium salt of nitrocyclohexane (**S20**, 2.8 equiv.) were added to a flame-dried reaction vial under N_2 with a stir bar irradiated at 427 nm for 18 h. In the second, the same conditions as the first were used except with the addition of 0.10 equiv. of nitrocyclohexane (**S20**). Upon completion, each reaction mixture was concentrated, and yields were determined by ^{1}H NMR spectroscopy using CH_2Br_2 (δ 4.93, s, 2H) as an internal standard. The results are shown below. *Note.* Isolated sodium nitroalkane salts were used for stability and accessibility, though strong sodium coordination renders the corresponding nitronates less reactive.

Table S7. Study involving the presence of sacrificial nitroalkanes.

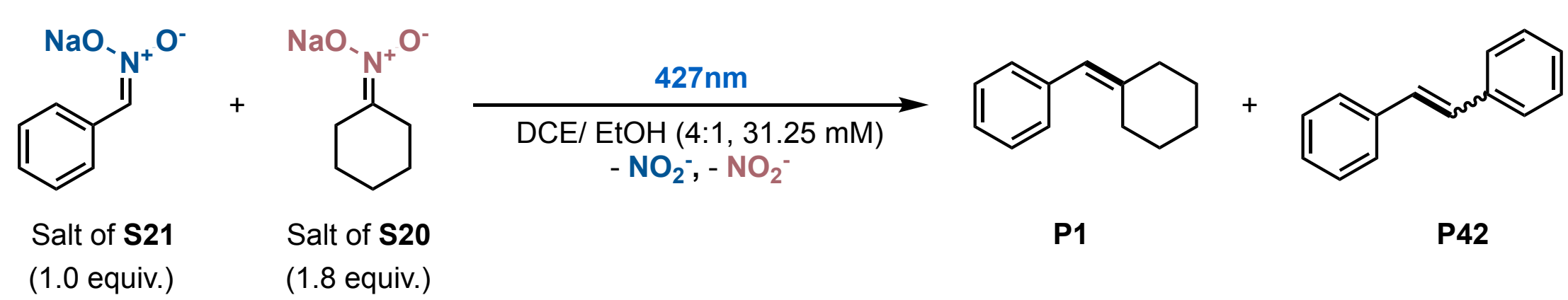

Entry	*Additive*	*Yield (**P1**)* [a]	*Yield (**P42**)* [a, b]
1	--	ND	11% (1.2:1 E/Z)
2	10 mol% **S20**	15%	84% (2.8:1 E/Z)

[a] Denotes ^{1}H NMR yields using CH_2Br_2 as an internal standard. [b] Includes both E/Z products. *Note.* Unreacted Na-nitronates were confirmed by titration with an acid, followed by GC–MS analysis.

Results. The presence of nitroalkane was required to observe appreciable product formation. Crude ^{1}H NMR spectra from entries 1 and 2 showing ***E*-P42** (δ 7.11, s, 2H, E isomer), ***Z*-P42** (δ 6.60, s, 2H, Z isomer), and **P1** (δ 6.23, s, 1H) indicate that small amounts of nitroalkane are required to serve as a sacrificial oxidant.

¹H NMR (500 MHz) of the crude reaction from Entry 1 of Table S7.

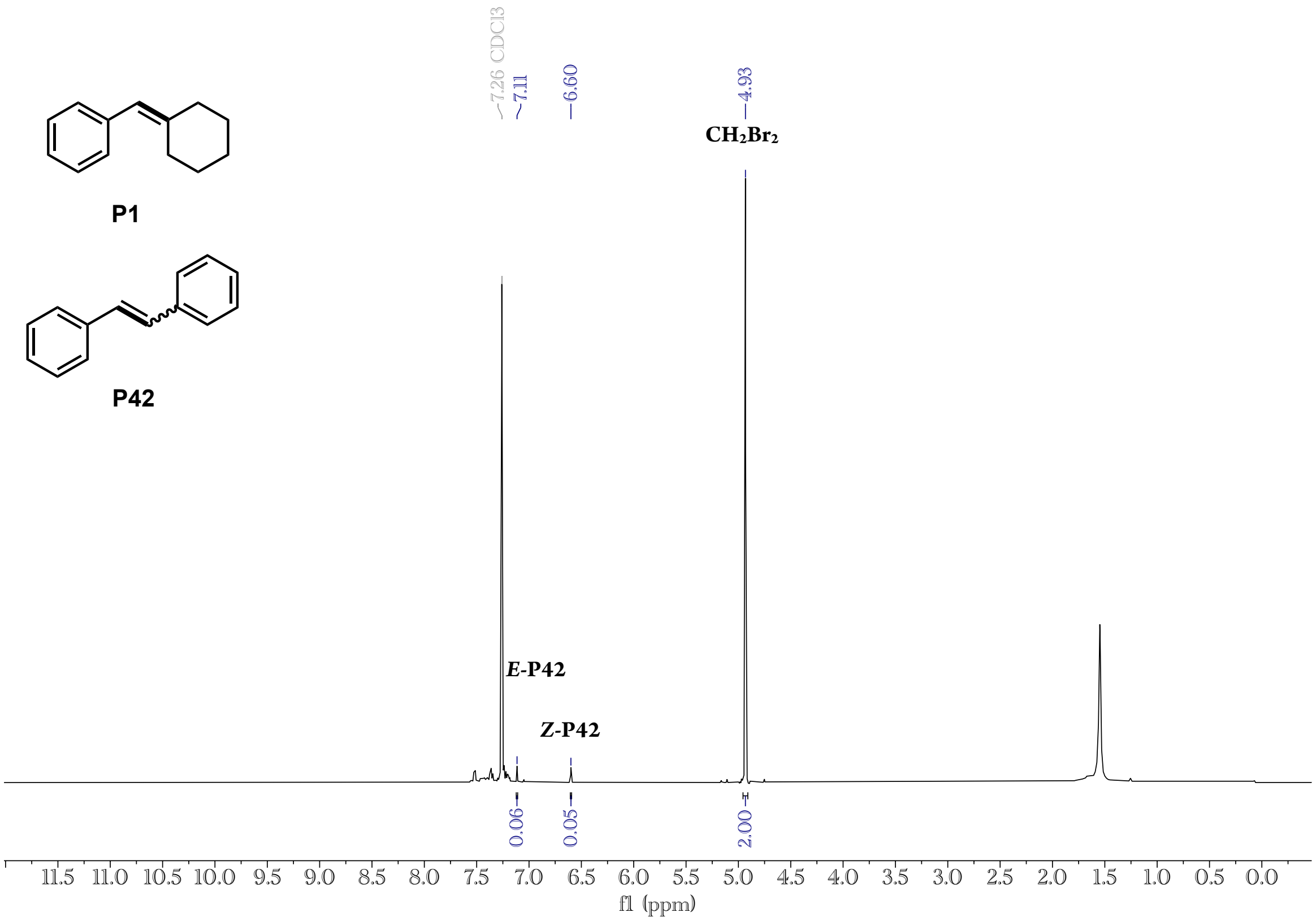

¹H NMR (400 MHz) of the crude reaction from Entry 2 of Table S7.

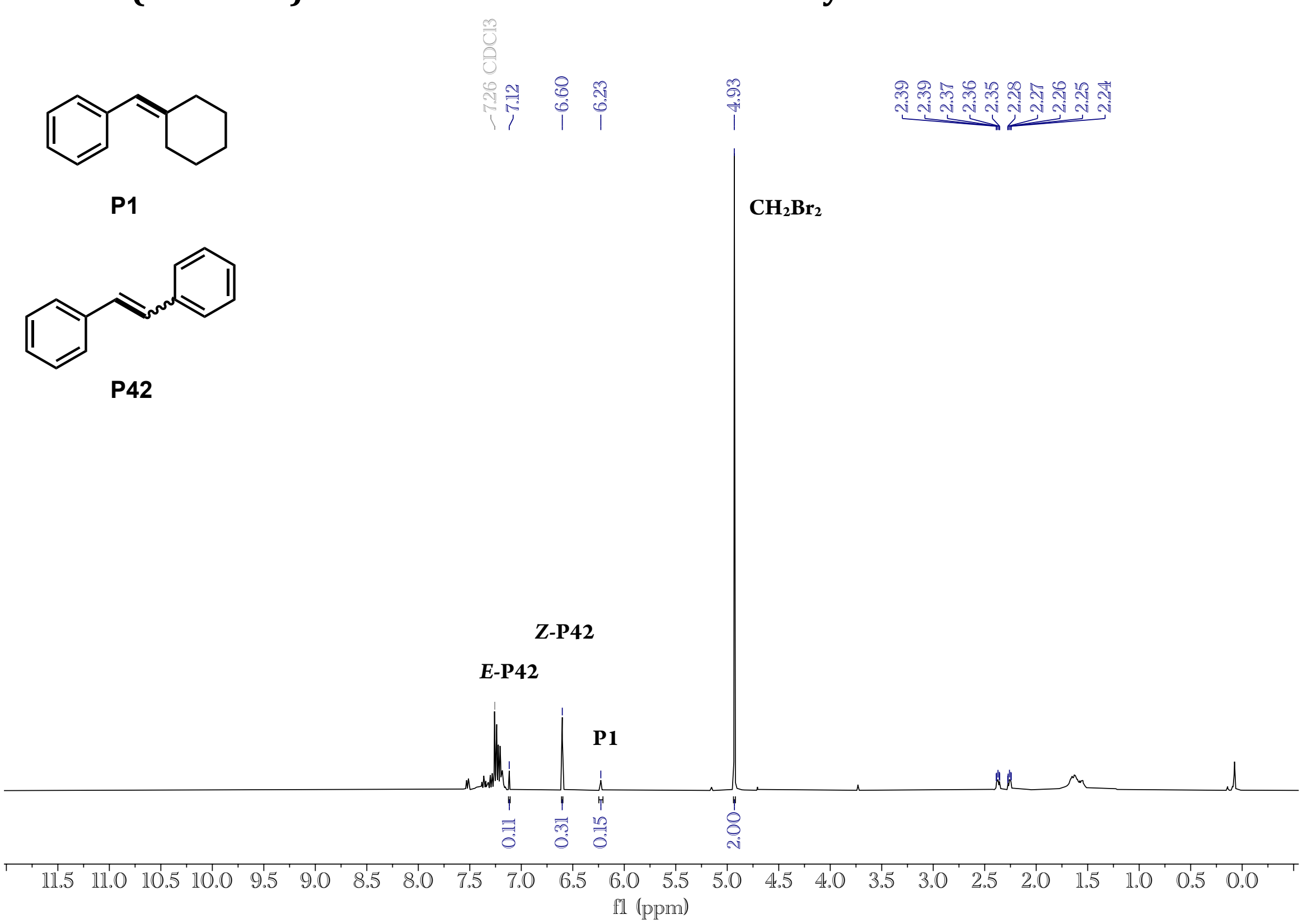

EPR Study

The EPR measurement was conducted on a Bruker ELEXSYS E500 Spectrometer System in continuous-wave mode in the X-band frequency using a Bruker ER 4122SHQE resonator. The parameters for the experiments are: temperature = 298 K, microwave frequency = 9.350080 GHz, power = 0.4743 mW, modulation frequency = 100.00 kHz, modulation amplitude = 0.5 G. EPR simulations were performed using MATLAB R2025b (MathWorks) and EasySpin (v6.2.0).

Reasoning. Photoexcited nitronates can potentially act as photoreductants, confirmed by our cyclic voltammetry studies (**Fig. S3**). Our mechanism proposes a single electron transfer (SET) from a photoexcited nitronate to a nitroalkane to initiate the $S_{RN}1$ cascade. This should result in a nitrobenzyl radical, which we can confirm through a radical trap experiment. Initial attempts to detect the EPR signal for the nitrobenzyl radical itself was unsuccessful, thus we employed *tert*-nitrosobutane (*t*-BuNO) as a radical trap.

Reaction setup. In a glovebox, a one-dram vial was charged with (nitromethyl)benzene (**S1**, 13.7 mg, 0.10 mmol, 1.0 equiv.), Cs_2CO_3 (32.6 mg, 0.10 mmol, 1.0 equiv.), and a co-solvent mixture of 1,2-DFB (900 µL) and *t*-BuOH (100 µL). The vial was transferred outside of the glovebox and 2-Methyl-2-nitrosopropane dimer (87.1 mg, 0.5 mmol, 5.0 equiv.) was added. The mixture was stirred at 23 °C for 3 hours and then placed in an ethanol bath that was chilled using a Julabo FT902 immersion cooler to -20 °C. The mixture was irradiated at using a 427 nm Kessil Lamp for 18 hours. Following the overnight run, the mixture was filtered under a nitrogen flow and transferred to an EPR tube (4 mm Thin Wall Quartz 250 mm L).

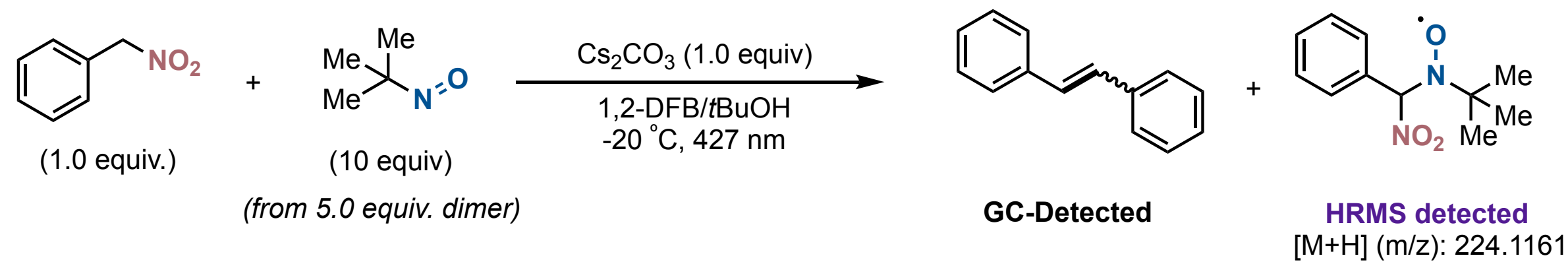

Scheme S1. Denitrative olefination with *t*-BuNO radical trap, forming stilbene and a nitroxide radical.

Results. EPR spectrum of the resulting crude from the above reaction displays three peaks, characteristic of a radical localized on an oxygen atom bound to a nitrogen (**Fig. S6**). The reaction led to detectable amounts of stilbene product by GC, which indicates the denitrative olefination reaction was still occurring and thus nitrobenzyl radical formation is expected if our proposed mechanism is operating. The *t*-BuNO trap was successful in capturing the *in*

situ formed nitrobenzyl radical, which has been previously reported (**HRMS** (ESI-TOF): *m/z* calculated for $C_{11}H_{16}N_2O_3$ [M]• = 224.1163, found 224.1161.)[21].

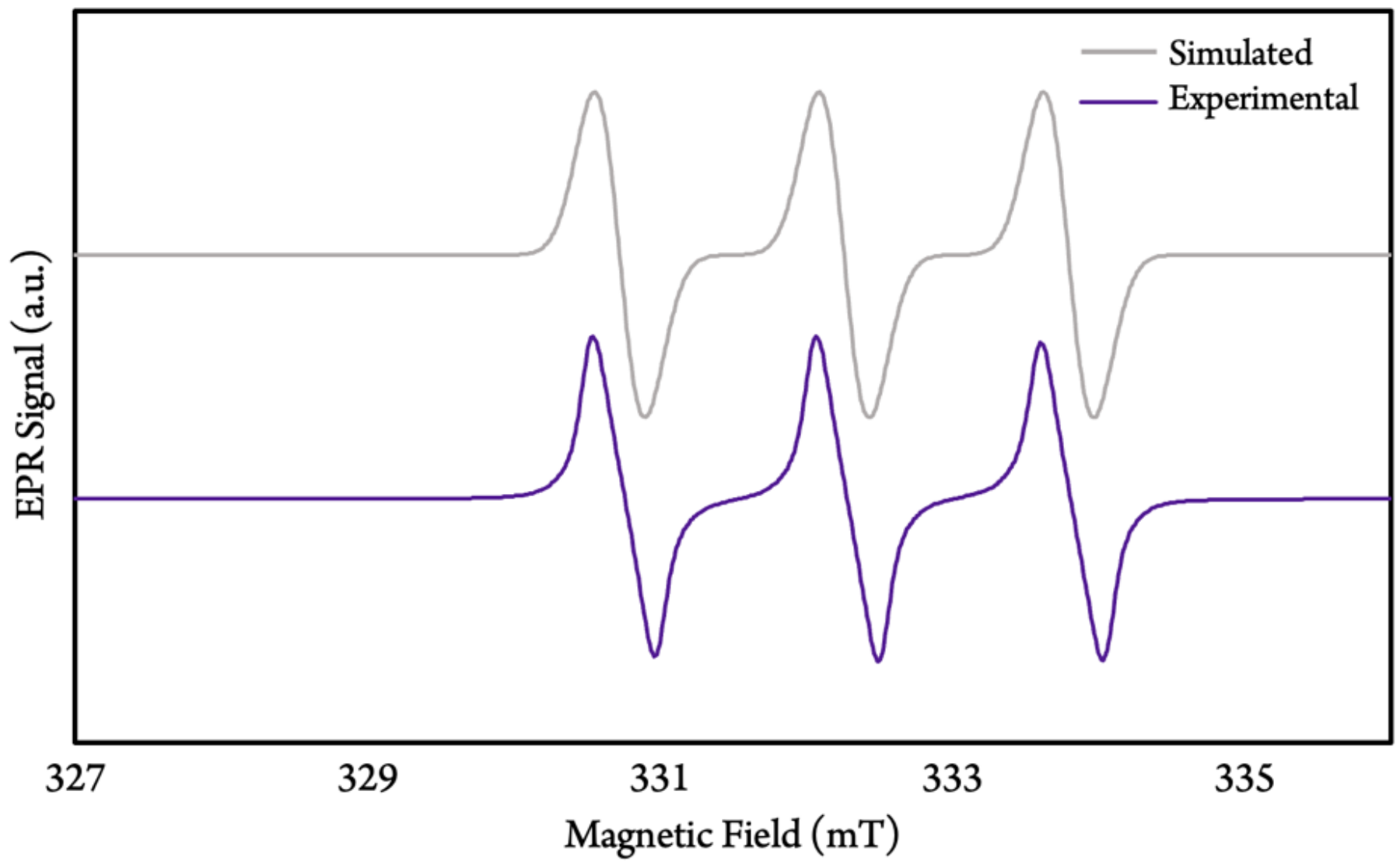

Fig. S6. X-band CW EPR spectrum of trapped **S1** α-nitrobenzyl radical in 1,4-dioxane (purple). Temperature = 298 K, microwave frequency = 9.350080 GHz, power = 0.4743 mW, modulation frequency = 100.00 kHz, modulation amplitude = 0.5 G. The parameters for the experimental spectrum (purple) are as follows: g = 2.0117, A_N = 43.2 MHz, line width = 0.04 mT. The simulated spectrum (gray) uses the following parameters: g = 2.0061, A_N = 43.2 MHz, line width = 0.04 mT.

SET Probe

Reasoning. We therefore assessed whether photoexcited nitronates could engage in single-electron transfer to species such as Katritzky salts (**1, Scheme S2**), providing evidence of their reducing capability. The resulting α-nitrobenzyl radical could then capture the carbon-centered radical derived from **1**, consistent with previously established $S_{RN}1$-type reactivity[4].

Reaction setup. In a glovebox, a one-dram vial was charged with (nitromethyl)benzene (**S1**, 50 μmol, 1.0 equiv.), Katritzky salt (**1**, 0.06 mmol, 1.2 equiv.), Cs_2CO_3 (0.05 mmol, 1.0 equiv.), and 1,2-DFB (1.3 mL) and *t*-BuOH (320 μL). The vial was transferred outside of the glovebox and the mixture was stirred at 23 °C for 6 h followed by irradiation at -20 °C under 427 nm for 18 h. Upon completion, each reaction mixture was concentrated, and yields were determined by ^{1}H NMR spectroscopy using 0.10 mmol CH_2Br_2 (δ 4.93, s, 2H) as an internal standard. The results are shown below.

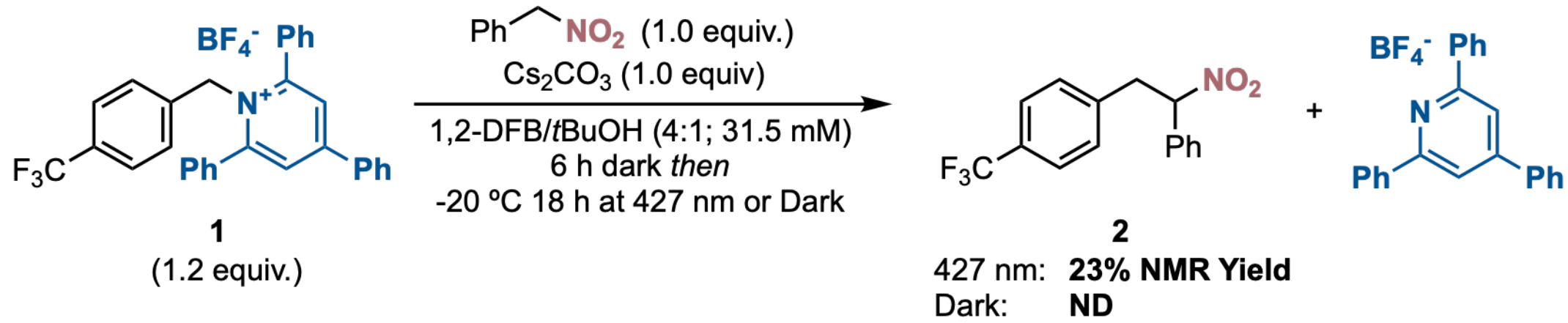

Scheme S2. SET Probe subjected to the reaction conditions.

Results. Under 427 nm irradiation, compound **2** was formed in 23% NMR yield, whereas no product formation was observed under dark conditions. These results are consistent with $S_{RN}1$-type reactivity involving single-electron transfer to reducible substrates such as pyridinium salts, triggering fragmentation of 2,4,6-triphenylpyridine followed by radical–radical coupling between α-nitro and benzylic radicals to afford **2**.

^{1}H NMR (500 MHz) crude spectrum of product 2

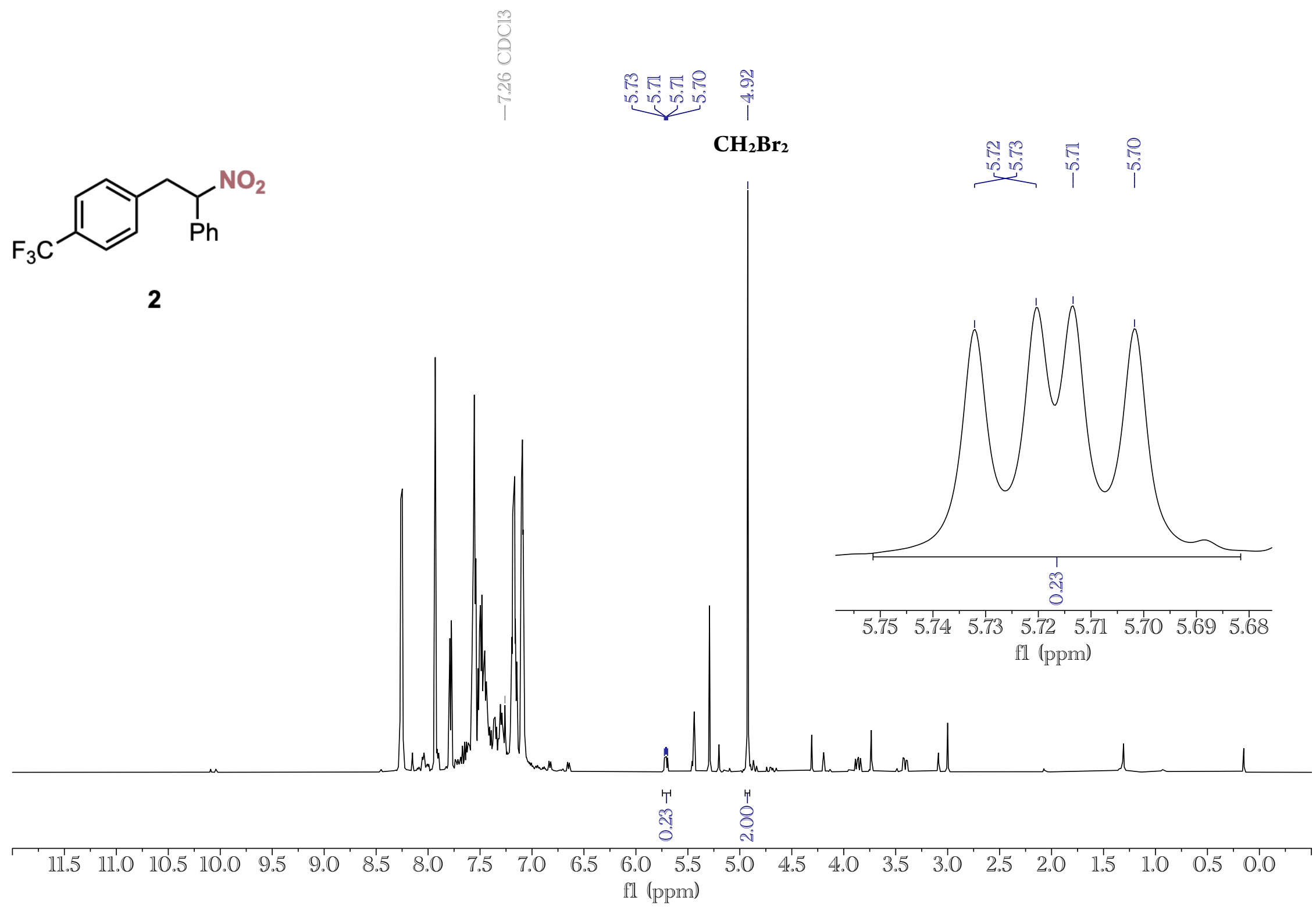

Elimination Probes

Reasoning. To rule out **Pathway 4** for an elimination leading to an alkene, we independently synthesized two nitroalkanes (**S51** and **S52**) through reported literature procedures[22, 23]. A successful conversion to an alkene from either of these probes via base alone would imply that these are chemically competent reaction intermediates, similar to previous literature[5], for compounds with more acidic β-hydrogen sites. Similarly, we aim to see if the presence of nitronate starting materials or nitrite byproducts would allow these potential intermediates to form alkene products via SET pathways.

Reaction setup I. In a glovebox, a one-dram vial was charged with **S51** or **S52** (0.10 mmol, 1.0 equiv.), base (0.20 mmol, 2.0 equiv.), and a co-solvent mixture of 1,2-DFB (800 µL) and *t*-BuOH (200 µL). The vial was transferred outside of the glovebox and the mixture was stirred at 28 °C for 18 hours. Three parallel reactions were performed for each probe: 1) stirring under 427 nm irradiation; 2) stirring without light; and 3) stirring without base.

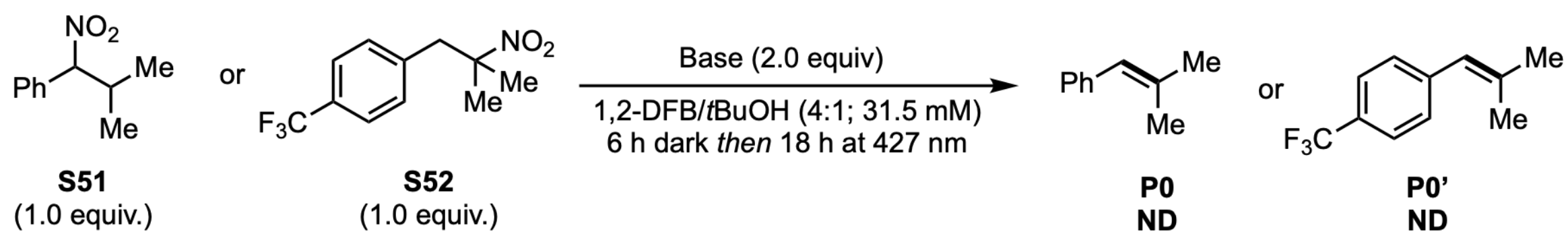

Scheme S3. Elimination probes subjected to varying bases under the conditions.

Reaction setup II. In a glovebox, a one-dram vial was charged with **S51** or **S52** (100 µmol, 1.0 equiv.), base (200 µmol, 1.0 equiv.), and a co-solvent mixture of 1,2-difluorobenzene (900 µL) and *tert*-butanol (100 µL). The vial was transferred outside of the glovebox and the mixture was stirred at 28 °C for 18 h. Three parallel reactions were performed for each probe: 1) stirring under 427 nm irradiation; 2) stirring without light; and 3) stirring without base.

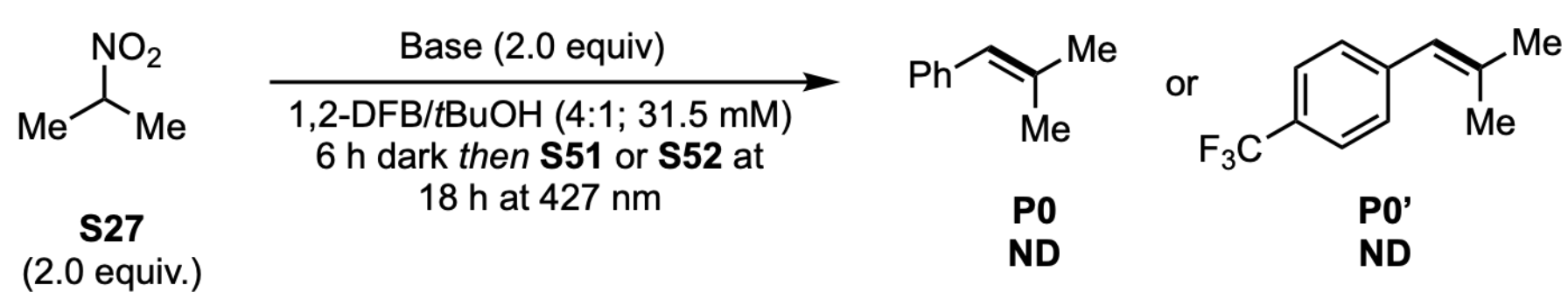

Scheme S4. Elimination Probes subjected to nitronates under the reaction conditions.

Results. A variety of bases were tested: 1,8-diazabicycloundec-7-ene (DBU), $CsHCO_3$, KOH, and *t*-BuOK. Sodium nitrite was also tested as an additive in each setup to gauge whether it can perform SET to the probes. We did not detect any alkene product in any of the reactions described above using GC–MS or ^{1}H NMR spectroscopy. It is likely that these nitroalkane scaffolds are not an intermediate towards olefination. A polar elimination pathway can be cautiously ruled out.

Association Probes

Geminal Functionalized Nitroalkane Probe Studies

Reasoning. To evaluate whether α-nitro radicals generated under our reaction conditions can associate with nitronates, we examined redox-favorable geminally functionalized nitroalkanes as mechanistic probes. Such substrates are known to preferentially form 1,2-dinitroalkanes rather than olefins through radical-anion intermediates[24]. Formation of 1,2-dinitroalkanes under our conditions would indicate that the early steps of the reaction are analogous to the Kornblum–Russell mechanism and that radical–nitronate association is accessible. In the productive olefination manifold, however, the reaction likely diverges at the radical-anion intermediate: rather than being quenched to a ground-state vicinal dinitroalkane, the intermediate undergoes further reduction and fragmentation, redirecting reactivity away from vicinal functionalization and toward alkene formation.

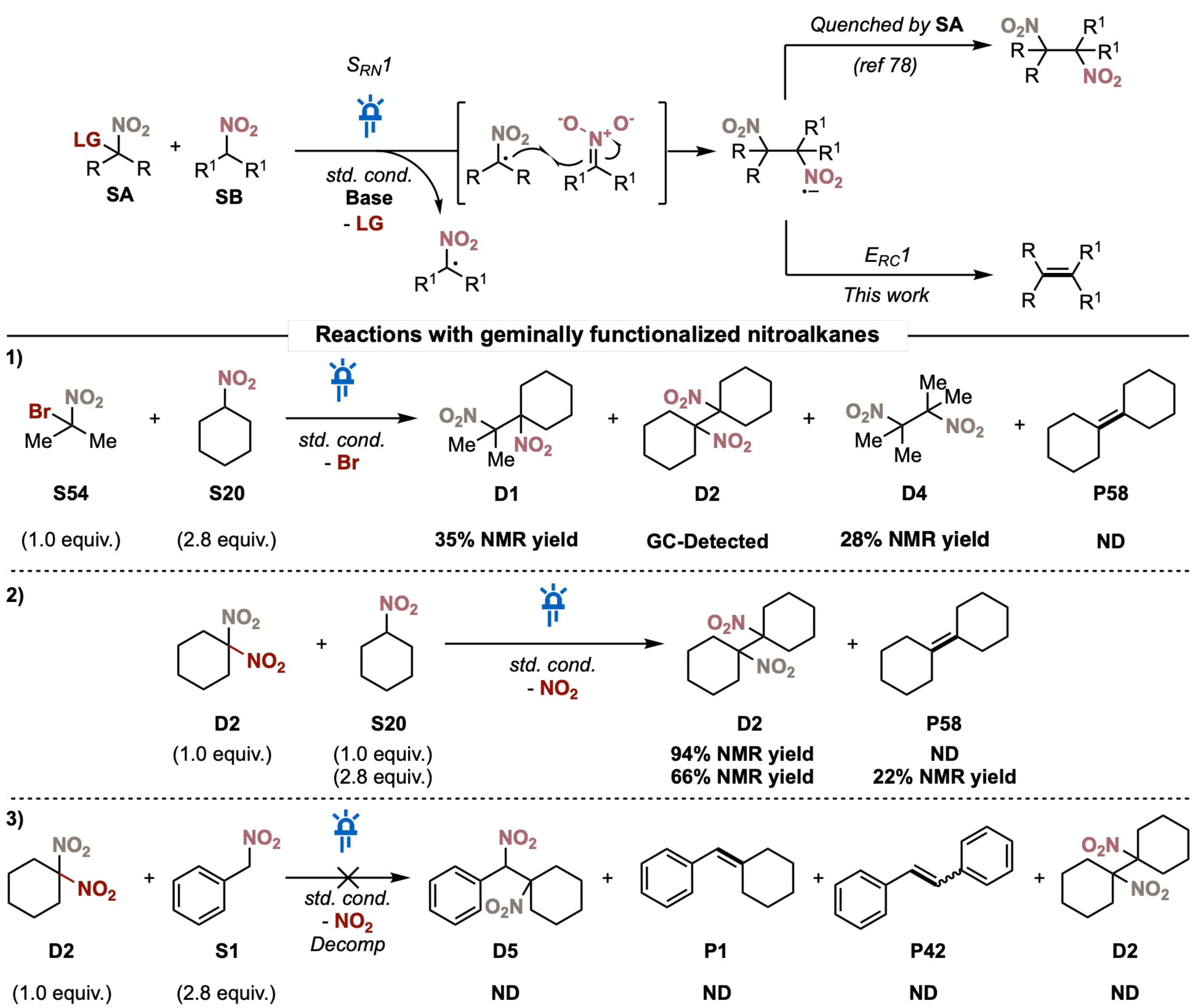

Fig. S7. Reactions of unfunctionalized nitroalkanes with geminally functionalized nitroalkanes under the standard conditions.

Reaction setup. Following General Procedure A, three reactions were conducted. In the first, 2-bromo-2-nitropropane (**S54**, 0.100 mmol, 1.0 equiv.), nitrocyclohexane (**S20**, 2.8 equiv.), and Cs_2CO_3 (2.7 equiv.) were added to a reaction vial under N_2 and stirred in the dark for 6 h, followed by irradiation at 427 nm for 18 h. In the second, 1,1-dinitrocyclohexane (**S55**, 1.00 mmol, 1.0 equiv.), nitrocyclohexane (**S20**, 2.8 equiv.), and Cs_2CO_3 (2.7 equiv.) were treated under identical conditions. In the third, 1,1-dinitrocyclohexane (**S55**, 1.00 mmol, 1.0 equiv.), (nitromethyl)benzene (**S1**, 1.0-2.8 equiv.), and Cs_2CO_3 (1.0-2.7 equiv.) were subjected to the same protocol. Upon completion, each reaction mixture was concentrated, and yields were determined by ^{1}H NMR spectroscopy using CH_2Br_2 (δ 4.93, s, 2H) as an internal standard.

Results. Under the reaction conditions, functionalized nitroalkanes afforded vicinal dinitroalkanes in moderate to high yield, with little to no olefin formation observed for unactivated nitroalkane partners (**Fig. S7**, entries **1–2**). Use of 2-bromo-2-nitropropane (**S54**) produced 1-nitro-1-(2-nitropropan-2-yl)cyclohexane (**D1**, 37%; δ 1.73, s, 12H) as the cross-coupled product and 2,3-dimethyl-2,3-dinitrobutane (**D4**, 28%; δ 1.68, s, 6H) as the homo-coupled product. 1,1′-dinitro-1,1′-bi(cyclohexane) (**D2**) was detected by GC–MS but could not be resolved in the crude 1H NMR spectrum due to signal overlap.

Reaction of 1,1-dinitrocyclohexane with 1.0 equiv. of nitrocyclohexane and base afforded 1,1′-dinitro-1,1′-bi(cyclohexane) (**D2**) in 94% NMR yield with no detectable alkene formation, whereas excess nitrocyclohexane (2.8 equiv.) resulted in 22% alkene formation (**P58**) alongside 66% of the vicinal dinitro-coupled product. Activated nitroalkanes, such as **S1**, predominantly underwent uncharacterized decomposition.

Collectively, these results are consistent with an $S_{RN}1$-type pathway analogous to that reported in the literature[24]; however, it is apparent that our system diverges in that it does not terminate at ground-state vicinal dinitroalkanes, likely indicating a distinct downstream fate of the radical-anion intermediate.

^{1}H NMR (500 MHz) of the crude reaction from Entry 1 of Fig S8.

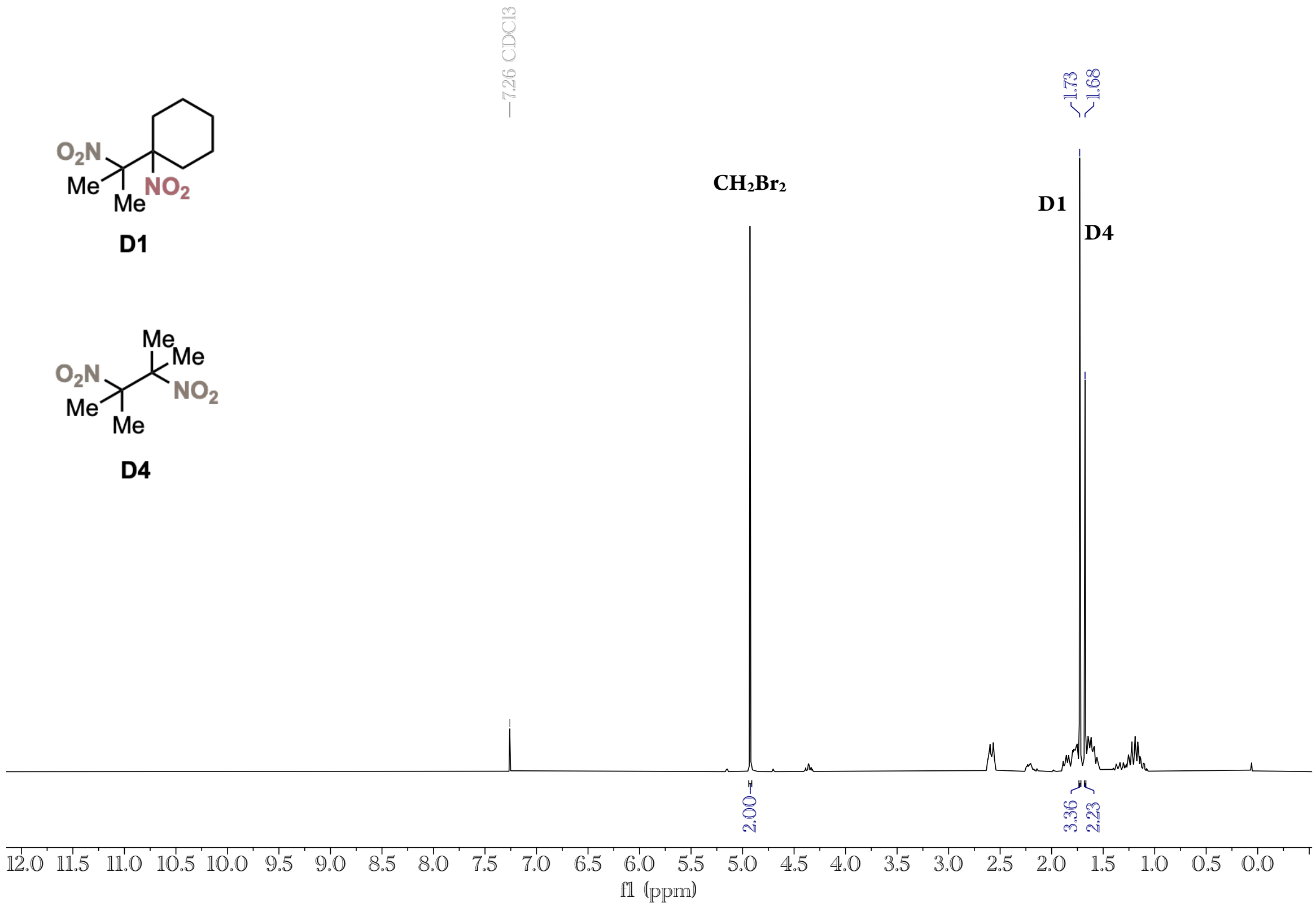

^{1}H NMR (400 MHz) of the crude reaction from Entry 2 of Fig S8. w/ 1.0 equiv. of S20

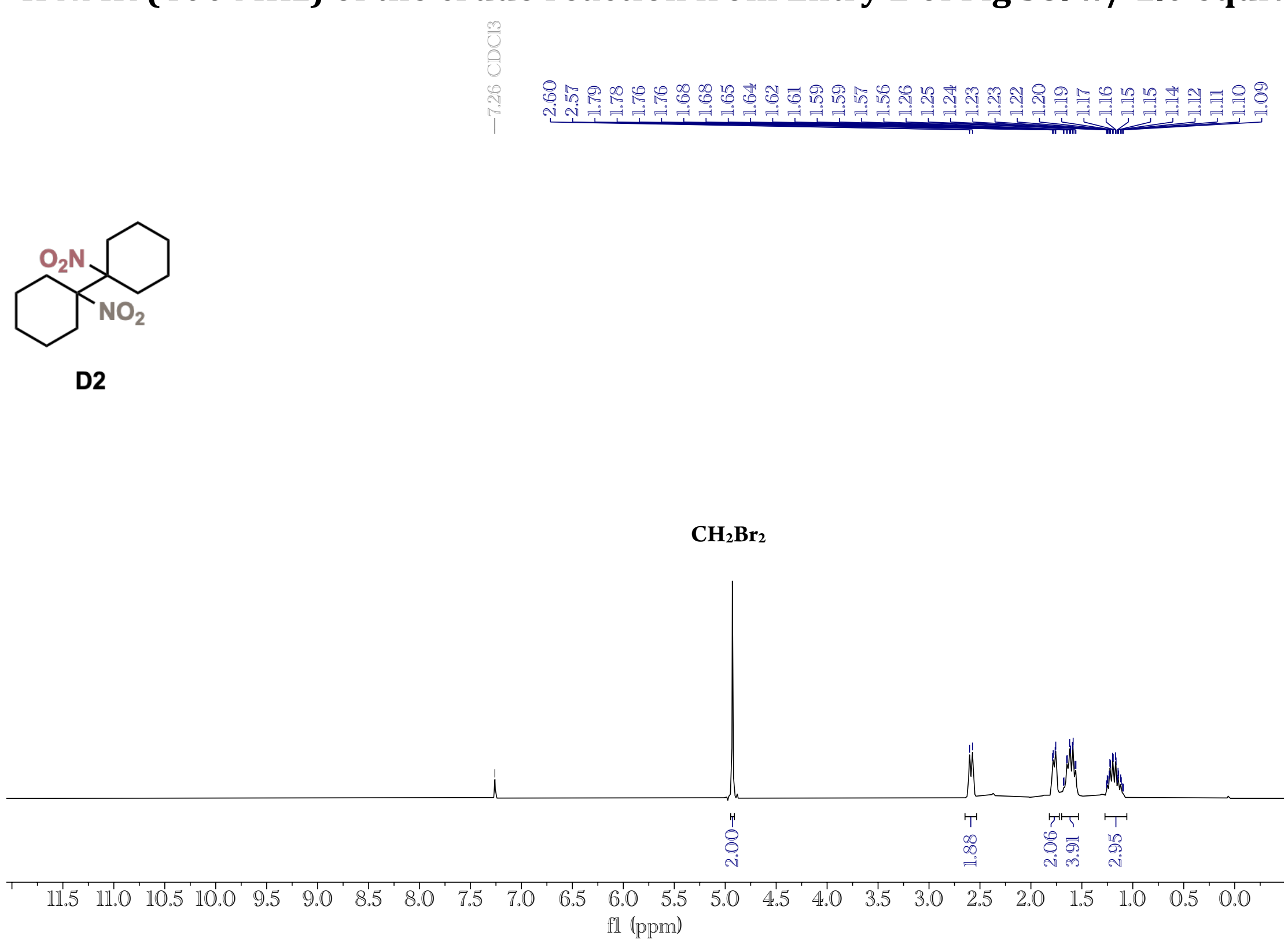

Alternative Nucleophile Traps

Reasoning. Our proposed mechanism initiates with an $S_{RN}1$-type single electron reduction from a nitronate to a nitroalkane to form an α-nitrobenzyl radical. This carbon-centered radical should undergo association with another nitronate to form a radical anion, which leads to the new C–C bond that will eventually form the alkene. We investigated this association step using a nucleophile analogous to a nitronate, i.e. a phosphonium ylide. Unfortunately, analogous sulfur ylides proved difficult due to their short lifetimes and unselective deprotonation.

Reaction setup. In a glovebox, a one-dram vial was charged with a phosphonium bromide salt, (hexyltriphenylphosphonium bromide, 2.8 equiv.), sodium hydride (2.7 equiv.) and 1,2-DFB (0.8 mL), and the reaction mixture was stirred vigorously for 6 h. In a separate vial was added nitroalkane (**S1**, 5 µmol, 1.0 equiv.), Cs_2CO_3 (1.0 equiv.), and 1,2-DFB (0.8 mL) and the reaction mixture was stirred vigorously 6 h. The vials were then combined, sealed, and was transferred outside of the glovebox and the mixture was irradiated under 427 nm for 18 h. Upon completion, each reaction mixture was concentrated, and yields were determined by ^{1}H NMR spectroscopy using CH_2Br_2 (δ 4.93, s, 2H) as an internal standard. The results are shown below.

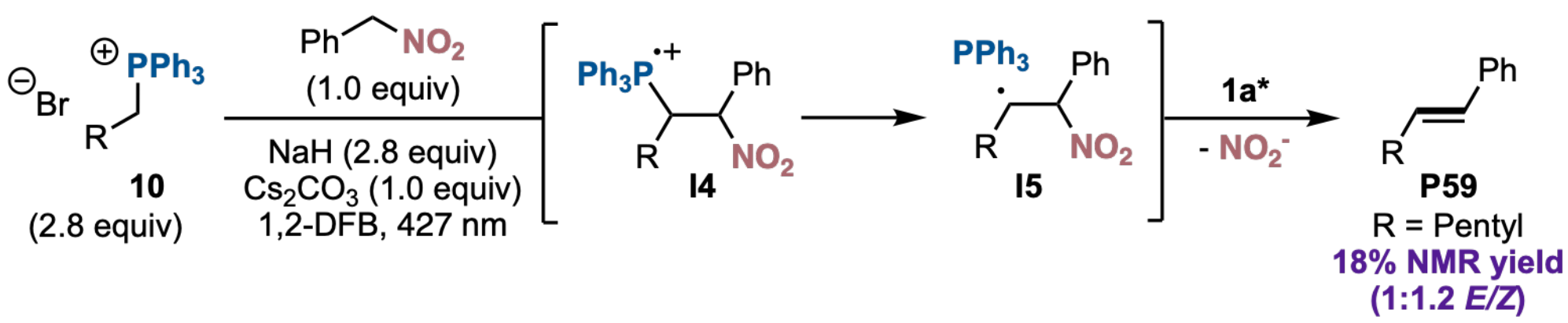

Scheme S5. Association Probe (phosphorous ylide) subjected to the reaction conditions.

Results. Alkene products were obtained, consistent with nucleophilic capture of the α-nitro radical. Alkyl ylides produced alkene **P59** in 18% NMR yield with a 1:1.2 *E/Z* ratio, consistent with a nonselective radical fragmentation pathway. Alternatively, a lack of a substituent capable of stabilizing the intermediate could also result in this unselective outcome. Aryl substituents stabilize the intermediate, enforcing *E*-selective elimination. A Wittig reaction as a result of the ylide reacting with Nef byproducts could not be ruled out.

¹H NMR (500 MHz) of crude product P59

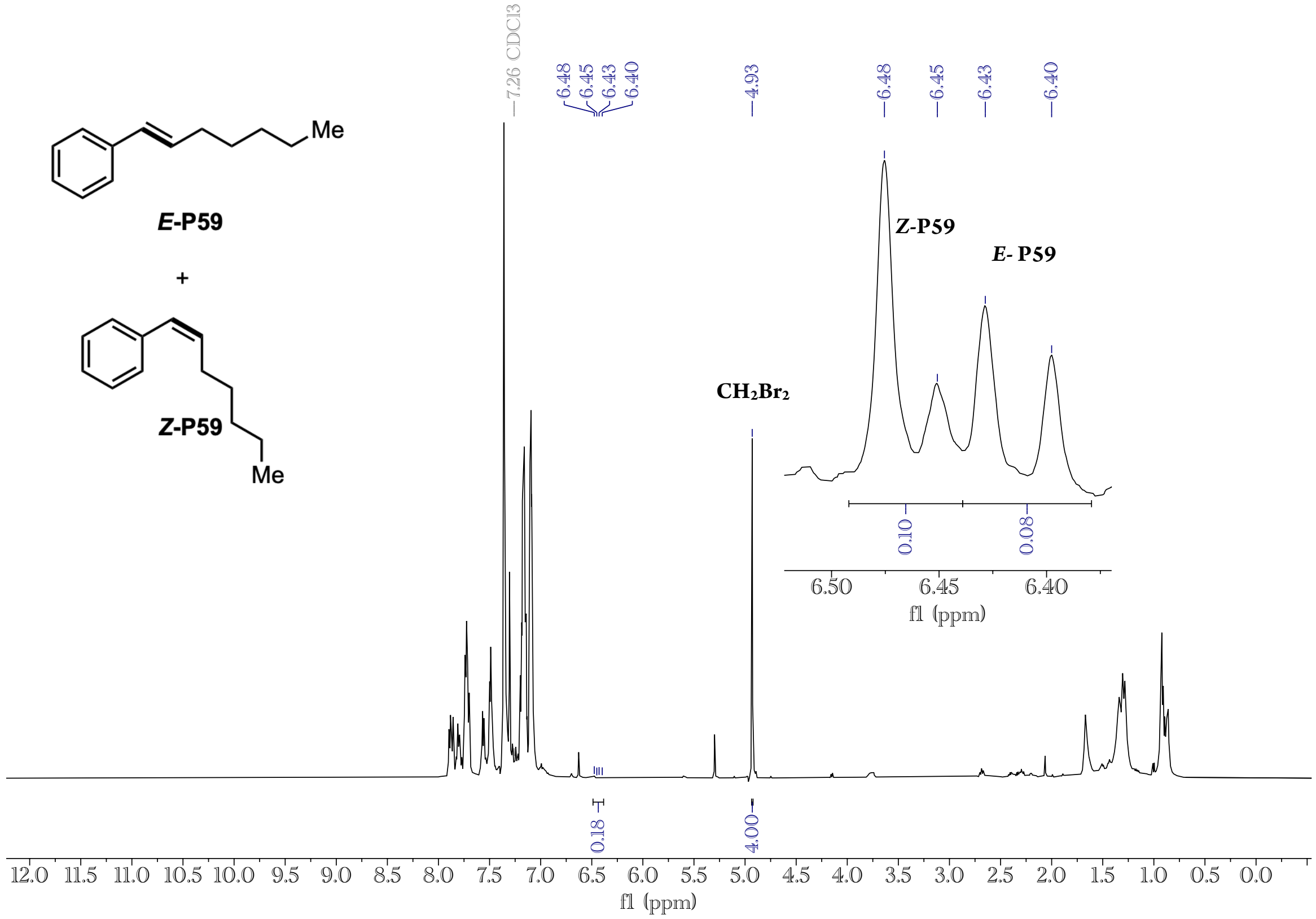

Vicinal Dinitroalkane Probes

Photoinduced Reduction of Activated 1,2-Dinitroalkanes in the Absence of Nitronates

Reasoning. Given the trace detection of 1,2-dinitroalkanes, the possibility that ground-state 1,2-dinitroalkanes are formed as intermediates and subsequently reduced was considered. Activated 1,2-dinitroalkanes, such as 1,2-dinitro-1,2-diphenylethane, have been reported to undergo reduction under photoirradiation in certain solvents, including benzene[6] (**Pathway 5**, **Fragmentation Pathway B**). Accordingly, it was assessed whether a similar reduction pathway operates under our reaction conditions. These experiments were conducted both in the presence and absence of base.

Reaction setup. Following General Procedure A, 1,2-dinitro-1,2-diphenylethane (**D6**, 0.100 mmol, 1.0 equiv.) and Cs_2CO_3 (0.5 equiv.) were added to a reaction vial under N_2 and stirred in the dark for 6 h, followed by irradiation at 427 nm for 18 h. Upon completion, each reaction mixture was filtered and concentrated, and yields were determined by ^{1}H NMR spectroscopy using CH_2Br_2 (δ 4.93, s, 2H) as an internal standard.

Table S8. Evaluation of 1,2-dinitroalkane photoreduction in the absence of nitronate.

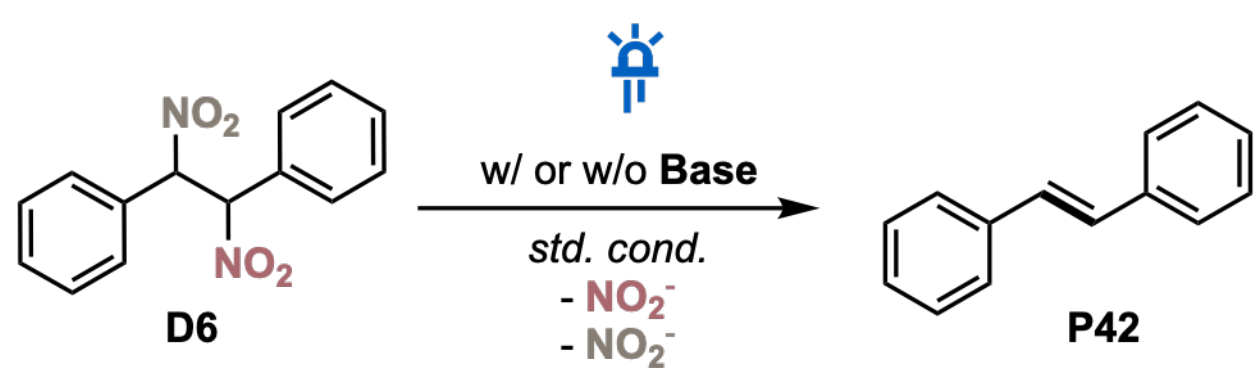

Entry	*Base (equiv.)*	*Yield (**P42**)* [a]
1	--	ND
2	Cs_2CO_3 (0.5 equiv.)	ND

Results. Under the reaction conditions, the activated 1,2-dinitroalkane (**D6**) did not undergo reduction, either in the presence or absence of base. This observation indicates that photodecomposition of ground-state 1,2-dinitroalkanes, whether mediated by solvent, base, or homolytic N–C bond cleavage, is unlikely to constitute a predominant pathway for olefin formation under these conditions.

Assessment of Unactivated 1,2-Dinitroalkane Reduction by Photoexcited Nitronates

Reasoning. Given that photoelectrochemical studies indicate in situ–generated nitronates act as photoreductants and that vicinal 1,2-dinitroalkanes are observed as trace byproducts, this study evaluates whether olefin formation proceeds through accumulation of ground-state 1,2-dinitroalkanes as predominant intermediates that are subsequently reduced by photoexcited nitronates, or instead through direct reduction of the radical-anion intermediate (**Pathway 5**, **Fragmentation Pathway A**). To distinguish between these possibilities, vicinal dinitroalkanes were synthesized and subjected to the reaction conditions.

Reaction setup. Following General Procedure A, 1,1-dinitrocyclohexane (**D2**, 1.00 mmol, 1.0 equiv.), nitroalkane (**S2** or **S21**, 2.8 equiv.), and Cs_2CO_3 (2.7 equiv.) were added to a flame-dried vial under nitrogen and subjected to 427 nm light for 18 h. Upon completion, each reaction mixture was concentrated, and yields were determined by ^1H NMR spectroscopy using CH_2Br_2 (δ 4.93, s, 2H) as an internal standard.

Table S9. Evaluation of 1,2-dinitroalkane reduction via photoexcited nitronates.

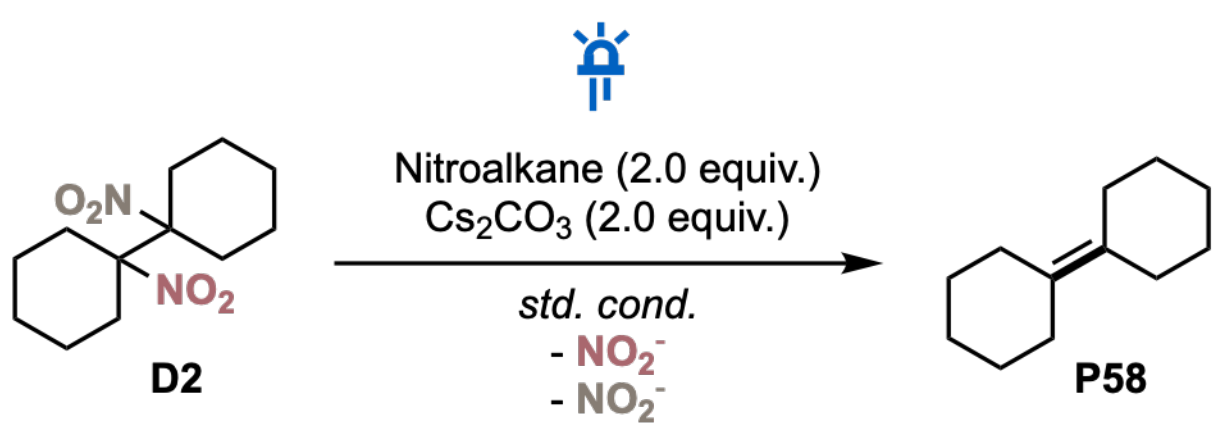

Entry	*Nitroalkane*	*Yield (**P58**)* [a]
1	1-chloro-4-(nitromethyl)benzene (**S2**)	2%
2	Nitrocyclododecane (**S21**)	ND

Results. Under the reaction conditions, activated 1-chloro-4-(nitromethyl)benzene (**S2**) and nitrocyclododecane (**S21**) were employed to generate nitronates in situ that could be photoexcited and potentially reduce the 1,2-dinitroalkane (**D2**). However, no or only trace olefin formation (**P58**) was observed, indicating that formation of ground-state 1,2-dinitroalkanes followed by their reduction by nitronate photoreductants does not constitute a major reaction pathway.

$E_{RC}1$ Probe

Reasoning 1. Classical $E_{RC}1$ reactions are typically thermally propagated by redox-favorable carriers. However, light-on/off experiments (**Fig. S4**) and control studies revealed no continued product formation in the absence of irradiation, indicating that the present system is unlikely to undergo thermal chain propagation, consistent with previously reported nitronate-mediated $S_{RN}1$ activity[4]. Instead, these observations suggest a photo-propagated $E_{RC}1$ pathway (**Pathway 5**, **Fragmentation Pathway C**).

To probe this hypothesis, 4-(dichloromethyl)benzonitrile (**3**) was employed as an $E_{RC}1$ probe. Without an aryl-nitro substituent, **3** is not a favorable ground-state redox carrier, enabling the evaluation of whether it can nonetheless engage a comparable mechanistic pathway under photochemical conditions, as previously reported by Vanelle and Norris with geminal dichloroalkanes[5,7]. Under irradiation, the photoexcited nitronate is expected to undergo single-electron transfer (SET) to **3**, generating an α-chloro benzyl radical, which subsequently associates with another equivalent of ground-state nitronate to form radical anion **I2**. From this intermediate, two divergent pathways are possible. One of which is ***Pathway A*** of **Fig. S8**, which involves a second SET event to generate a carbanion, which, upon elimination affords product **4**.

Reasoning 2. An alternative pathway, ***Pathway B*** of **Fig. S8**, proceeds**,** via quenching of **I2** followed by base-promoted elimination, analogous to a classical $S_{RN}1$ process followed by E2 elimination, leading to the formation of **6**.

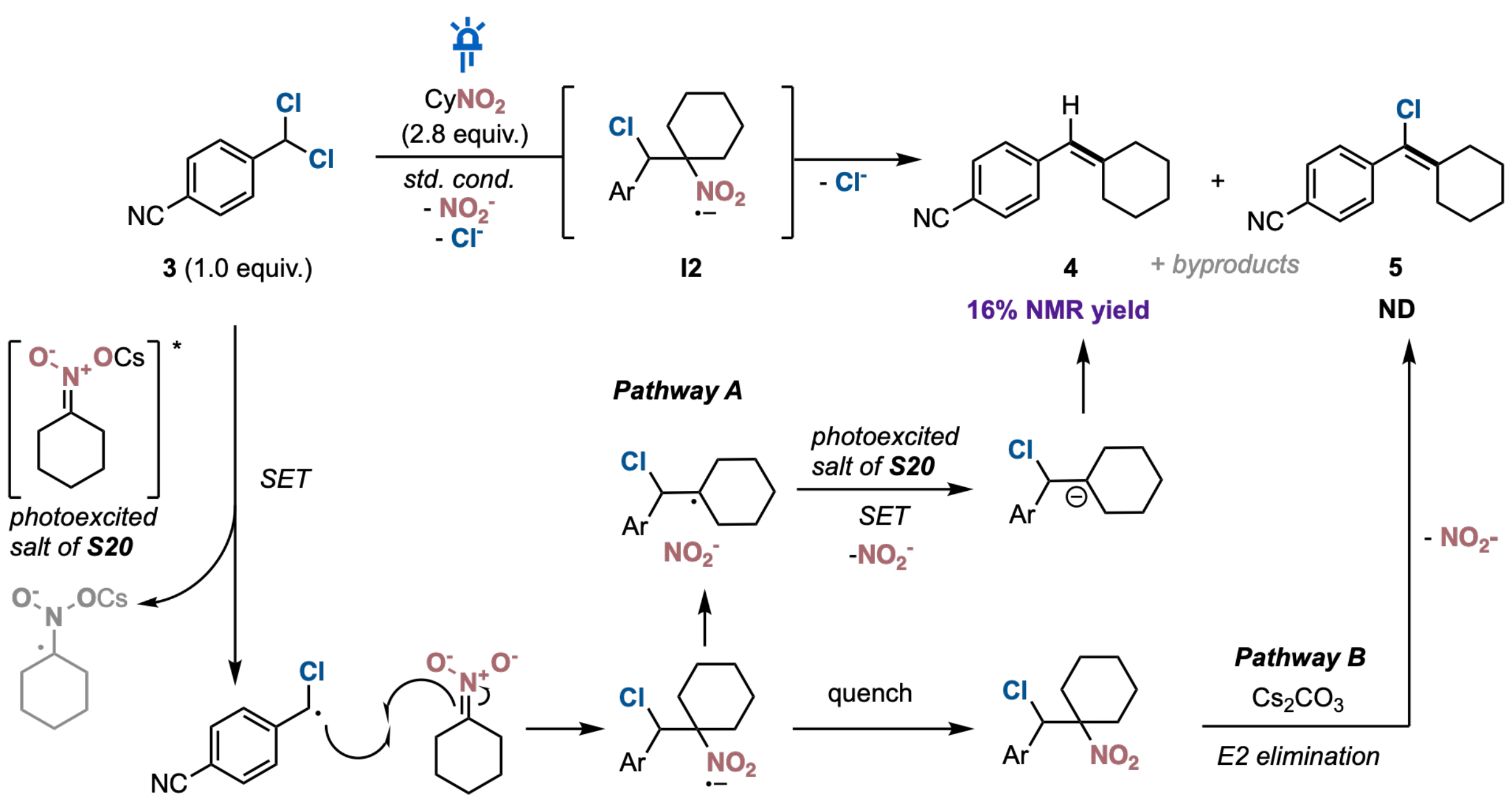

Fig S8. $E_{RC}1$ probe, 4-(dichloromethyl)benzonitrile, subjected to the reaction conditions.

Reaction setup. Following General Procedure A, 4-(dichloromethyl)benzonitrile (1.0 equiv.), nitrocyclohexane (2.8 equiv.), and Cs_2CO_3 (2.6 equiv.) were added to a reaction vessel under N_2 and stirred for 6 h, after which, the reaction vessel was subjected to 427 nm irradiation for 18 h. Upon completion of the reaction, the crude mixture was concentrated down ^{1}H NMR yields were obtained using CH_2Br_2 (δ 4.93 (s, 2H)) as an internal standard.

Results. A 16% ^{1}H NMR yield of **4** (δ 6.21 (s, 1H)) was observed. No E2 product **5** was detected. Other byproducts such as 16% ^{1}H NMR yield of 4-(chloromethyl)benzonitrile (**6**, δ 4.60 (s, 2H)) and 83% ^{1}H NMR yield of 1,1'-bi(cyclohexylidene) (**P58**, δ 2.20 – 2.15 (m, 8H)) with remaining 78% ^{1}H NMR yield (**3**, δ 6.71 (s, 1H)) of 4-(dichloromethyl)benzonitrile starting was detected.

These results support a photo-propagated $E_{RC}1$ mechanism and not an $S_{RN}1$ followed by E2 elimination reaction.

^{1}H NMR (500 MHz, $CDCl_3$) of the crude reaction involving the $E_{RC}1$ Probe

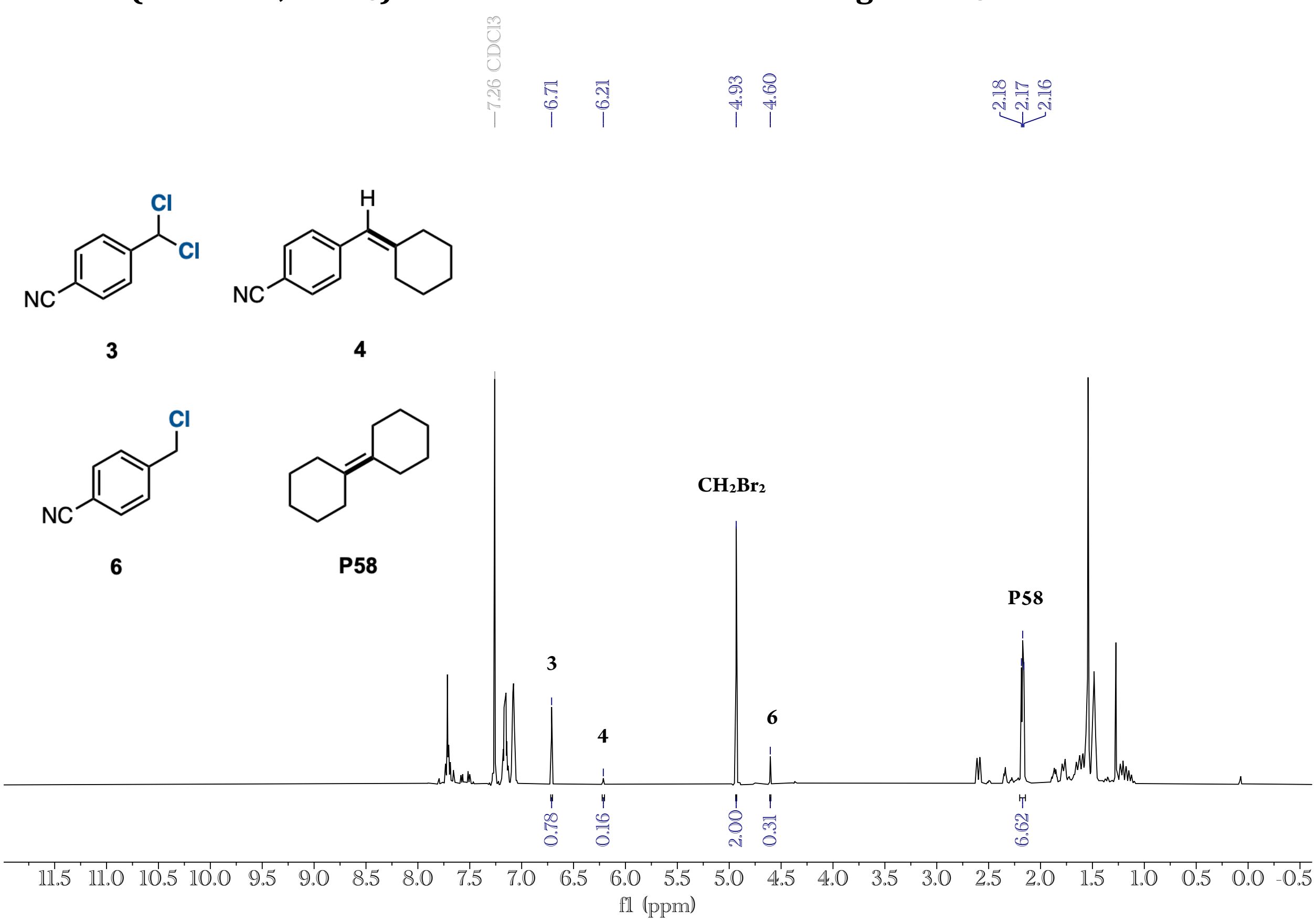

Griess Test for the Detection of Nitrite Ions

Reasoning. Our proposed mechanism (**Pathway 5, Fragmentation Pathway C**) should produce nitrite (NO_2^-) ions as by-products of the cross-denitrative olefination of two nitroalkanes. The Griess test is a sensitive colorimetric assay used to detect and quantify nitrite ions in a sample[25].

Procedure. In a glovebox, a one-dram vial was charged with 1-(nitromethyl)-3,5-bis(trifluoromethyl)benzene (**S11**, 0.10 mmol, 1.0 equiv.), Cs_2CO_3 (0.10 mmol, 1.0 equiv.), and 1,2-DFB (2.67 mL). The vial was transferred outside of the glovebox and the mixture was stirred at 23 °C for 6 h followed by irradiation at 28 °C under 427 nm for 18 h. Following the overnight run, the mixture was concentrated to remove 1,2-DFB and then 1 mL dH_2O (to ensure aq. solution [nitrite] ~ 0.1 M) was added. The crude mixture was covered in aluminum foil and sulfanilic acid (1% in 5% H_3PO_4 – stock stored at 4 °C) was added, and the mixture was stirred at 23 °C for 15 mins. Then, 0.1% *N*-1-naphthylethylenediamine (0.1% in H_2O – stock stored at 4 °C) and the mixture was stirred for 15 mins.

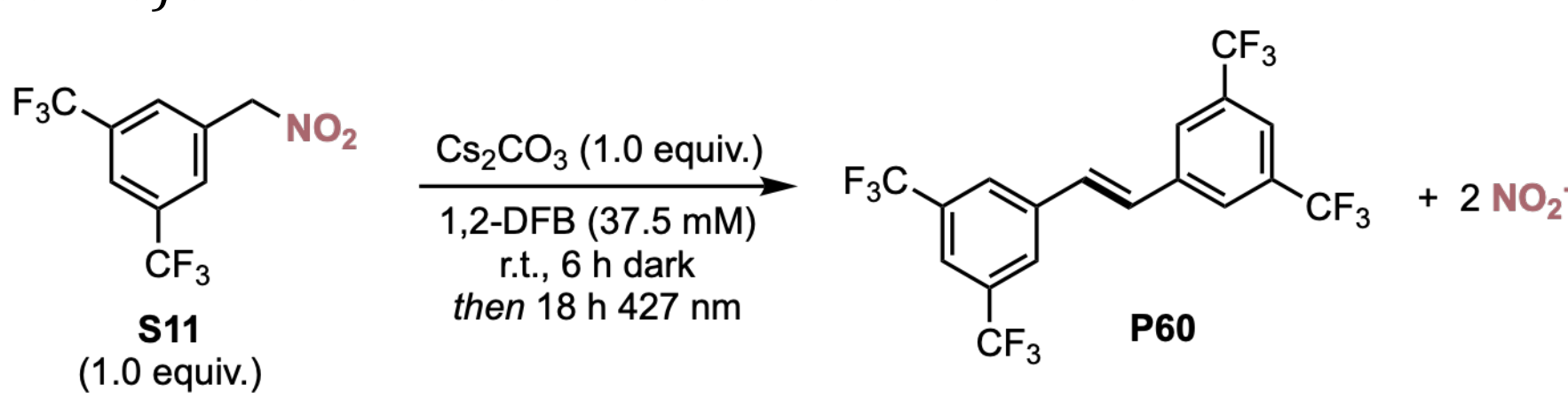

Scheme S6. Nitrite anion probe test.

Results. Following the addition of 0.1% *N*-1-naphthylethylenediamine, a color change from a yellow/orange characteristic of our standard crude solution to a red/magenta color indicated formation of the diazo dye as expected if the solution contained nitrite ions.

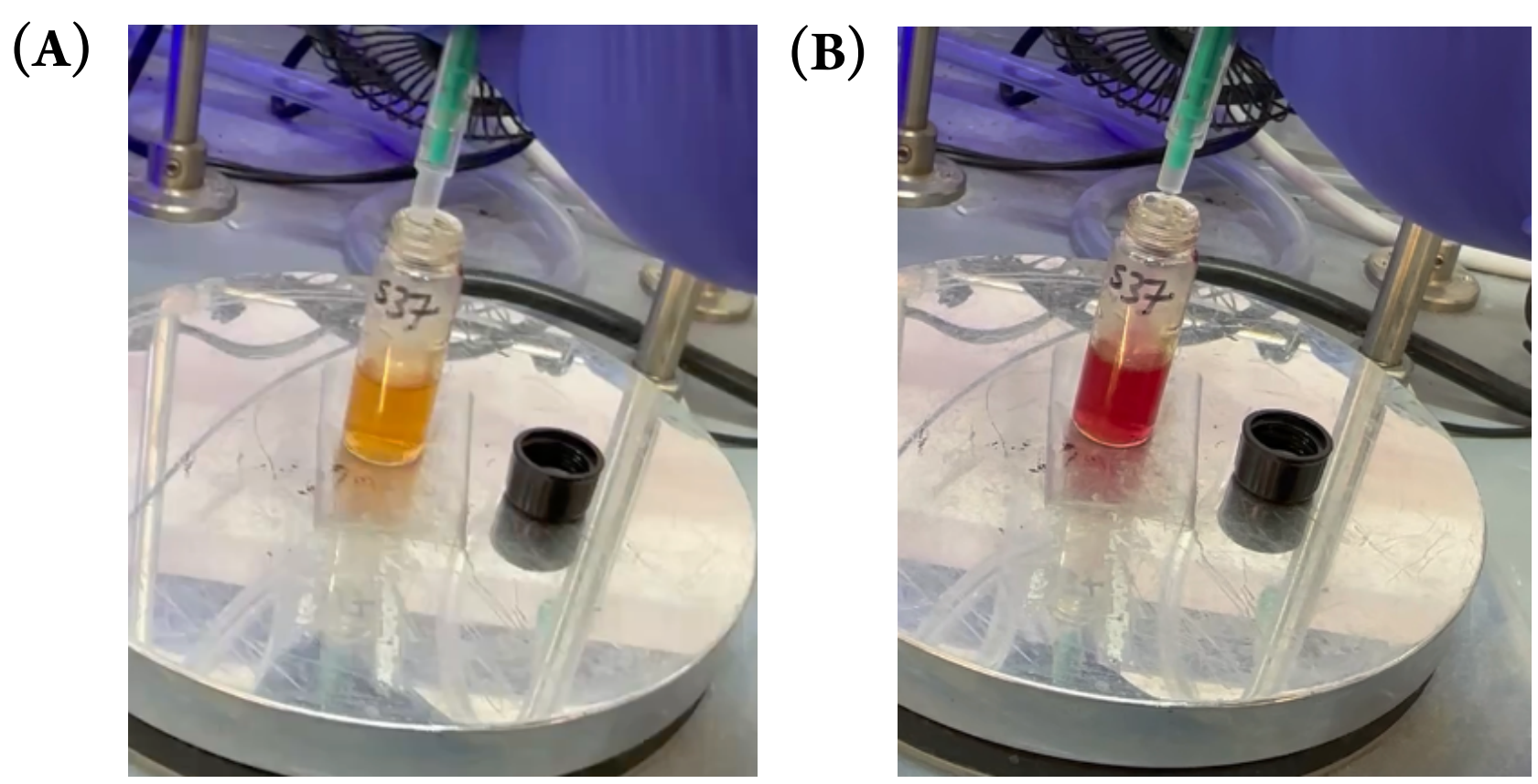

Fig. S9. (A) Reaction mixture after irradiation at 427 nm overnight. (B) Reaction mixture following addition of 0.1% *N*-1-naphthylethylenediamine.

Nitryl Radical Trap

Reasoning. A few of our proposed mechanistic pathways and traditional reactivity with nitroalkanes have the potential to form nitryl ($NO_2^\bullet$) radicals (**Pathway 5**, **Fragmentation Pathway D**). Aminopyrine was used as an effective indicator for the presence of nitryl radical[26, 27, 28].

Procedure. In a glovebox, a one-dram vial was charged with (nitromethyl)benzene (**S1**, 0.10 mmol, 1.0 equiv.), aminopyrine (**11**, 1.0 equiv.), Cs_2CO_3 (0.10 mmol, 1.0 equiv.) and 1,2-DFB (2.67 mL). The vial was transferred outside of the glovebox and the mixture was stirred at 23 °C for 6 h followed by irradiation at 28 °C under 427 nm for 18 h.

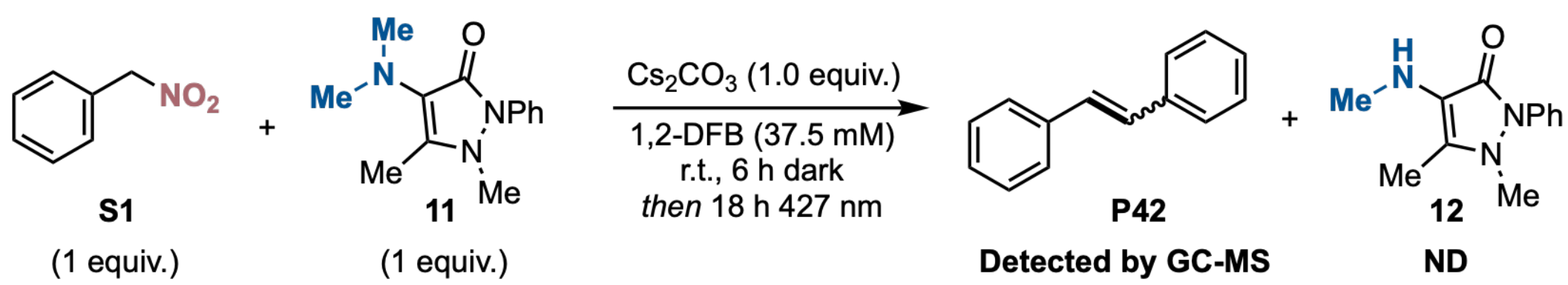

Scheme S7. A nitryl radical probe was added to the reaction conditions.

Results. SET from aminopyrine (**11**) and nitryl radicals is expected to result in the formation of aminopyrine radical cation, which undergoes disproportionation. Subsequent hydrolysis leads to formaldehyde and demethylated aminopyrine (**12**). Additionally, the solution following the reaction is expected to be deep purple/violet if nitryl radicals were present. We did not observe any expected demethylated aminopyrine product or color change despite the reaction producing the expected stilbene dimer from our cross-denitrative olefination. This indicates the absence of nitryl radicals through the course of the reaction.

Use of Oxaziridines, Nitronic Esters and Alkyl Nitrites to Probe as Potential Intermediates

Reasoning. Based on **Pathway 2-3** referenced earlier and the instability of 1,2-oxaziridin-2-olate intermediates, we used the following probes to test for olefin formation.

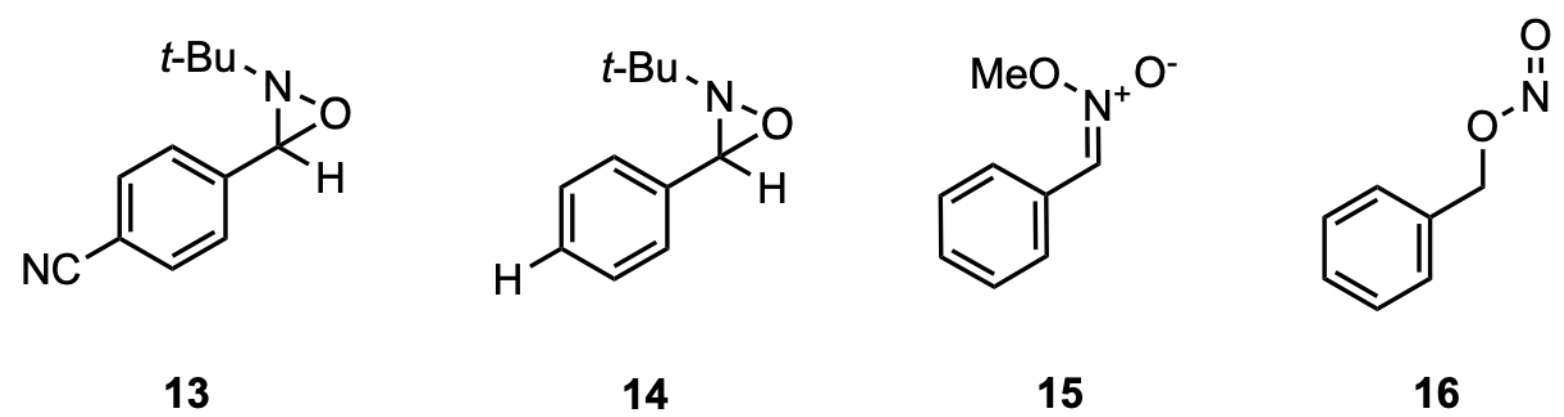

Scheme S8. List of probes subjected to the reaction conditions to help determine potential reaction intermediates.

Reaction setup. Following General Procedure A, in separate reactions, each probe (**13–16**) was combined with either 1-chloro-4-(nitromethyl)benzene (**S2**), nitrocyclohexane (**S20**), or (nitromethyl)benzene (**S1**) (2.8 equiv) and Cs_2CO_3 (3.7 equiv) or *t*BuOK (3.7 equiv) in a reaction vial under N_2. The mixtures were stirred in the dark for 6 h and then irradiated at 427 nm for 18 h. Upon completion, the reaction mixtures were filtered and concentrated, and yields were determined by ^{1}H NMR spectroscopy using CH_2Br_2 (δ 4.93, s, 2H) as an internal standard.

Results. Olefin was not detected as an outcome of these reactions.

Probing for Carbene Intermediates

Carbene Traps

Reasoning. Based on **Pathway 1**, where referenced earlier and the instability of 1,2-oxaziridin-2-olate intermediates, we used the following probes to test for olefin formation.

Reaction setup. Following General Procedure A, (nitromethyl)benzene (**S1**, 0.10 mmol), carbene trap (2.0 equiv.) and Cs_2CO_3 (1.0 equiv.) were added to a reaction vial under N_2, then stirred and irradiated at 427 nm for 18 h. Upon consumption of the starting material, each reaction mixture was filtered and concentrated. Yields were determined by ^{1}H NMR spectroscopy using CH_2Br_2 (δ 4.93, s, 2H) as an internal standard, and aliquots were analyzed by GC–MS.

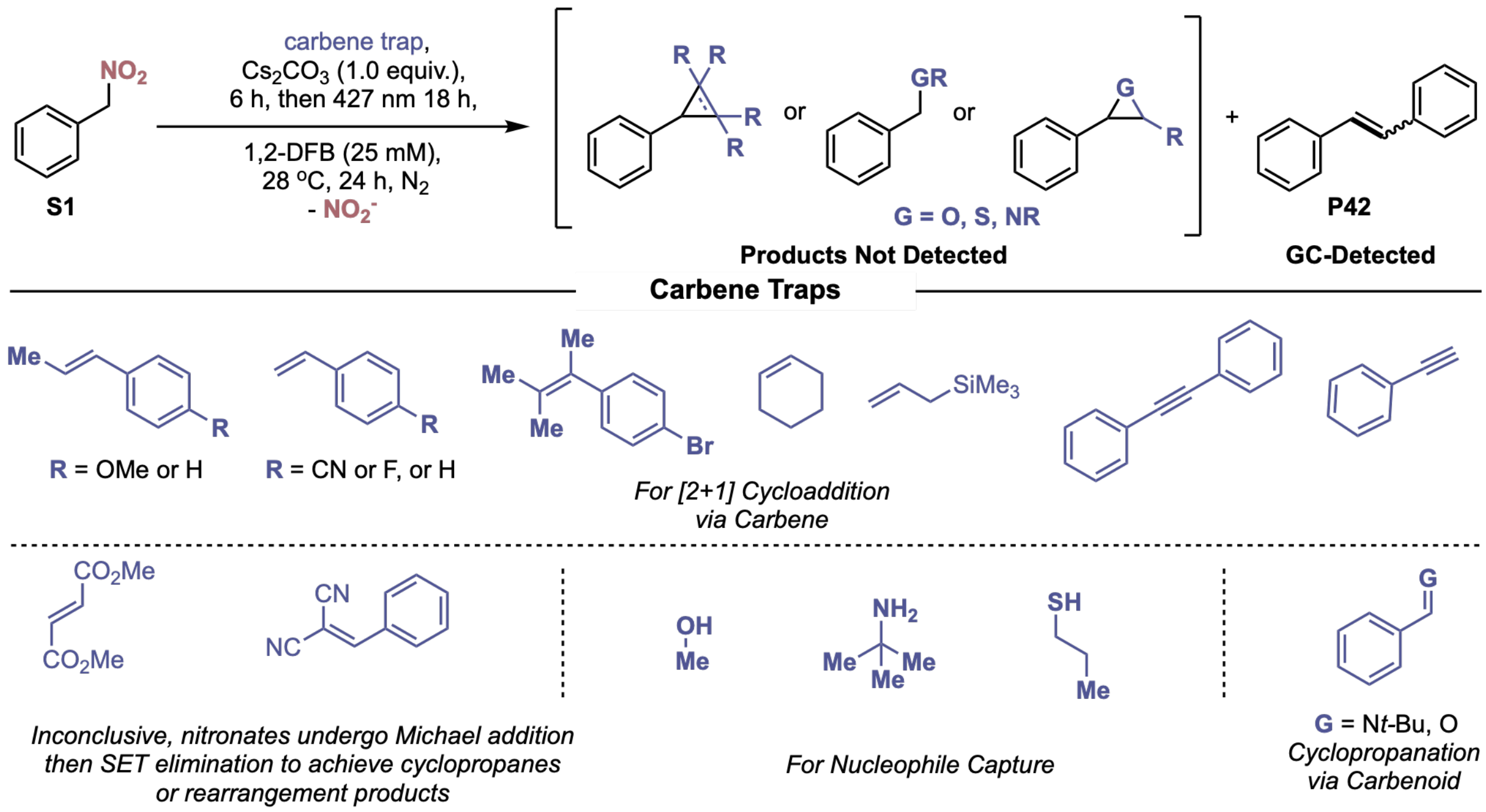

Fig. S10. Reactions of nitroalkanes with varying carbene traps nitroalkanes under the standard conditions.

Results. As depicted in **Fig. S10**, the use of carbene traps did not result in any detectable trapped products either by GC–MS or NMR. We therefore questioned whether nucleophilic capture by the nitronate outcompetes alkene cyclopropanation. Such behavior has been documented for sulfur ylides, prompting additional control studies, as shown below.

Competitive Carbene Reactions

Reasoning. Based on prior literature involving sulfur ylides[2] and the failure of the carbene traps, we questioned whether nucleophilic capture by in situ–generated nitronates via carbene intermediates could outcompete alkene cyclopropanation. To probe this possibility, tosylhydrazones were employed as photochemical carbene precursors in parallel and competitive studies.

Reaction setup. Following General Procedure A, four reactions were performed. (Nitromethyl)benzene (**S1**, 0.100 mmol, 1.0 equiv.), styrene (2.0 equiv.), and Cs_2CO_3 (1.0 equiv.) were combined in 1,2-DFB (25 mM) under N_2 and irradiated at 427 nm for 18 h. The second reaction was conducted using benzaldehyde p-toluenesulfonylhydrazone (**18**) in place of **S1**. The third reaction employed 4-(nitromethyl)benzonitrile (**S49**, 1.0 equiv.) and **18** (1.0 equiv.) under otherwise identical conditions, and the fourth reaction was identical to the third with 4-fluorostyrene (2.0 equiv.) added.

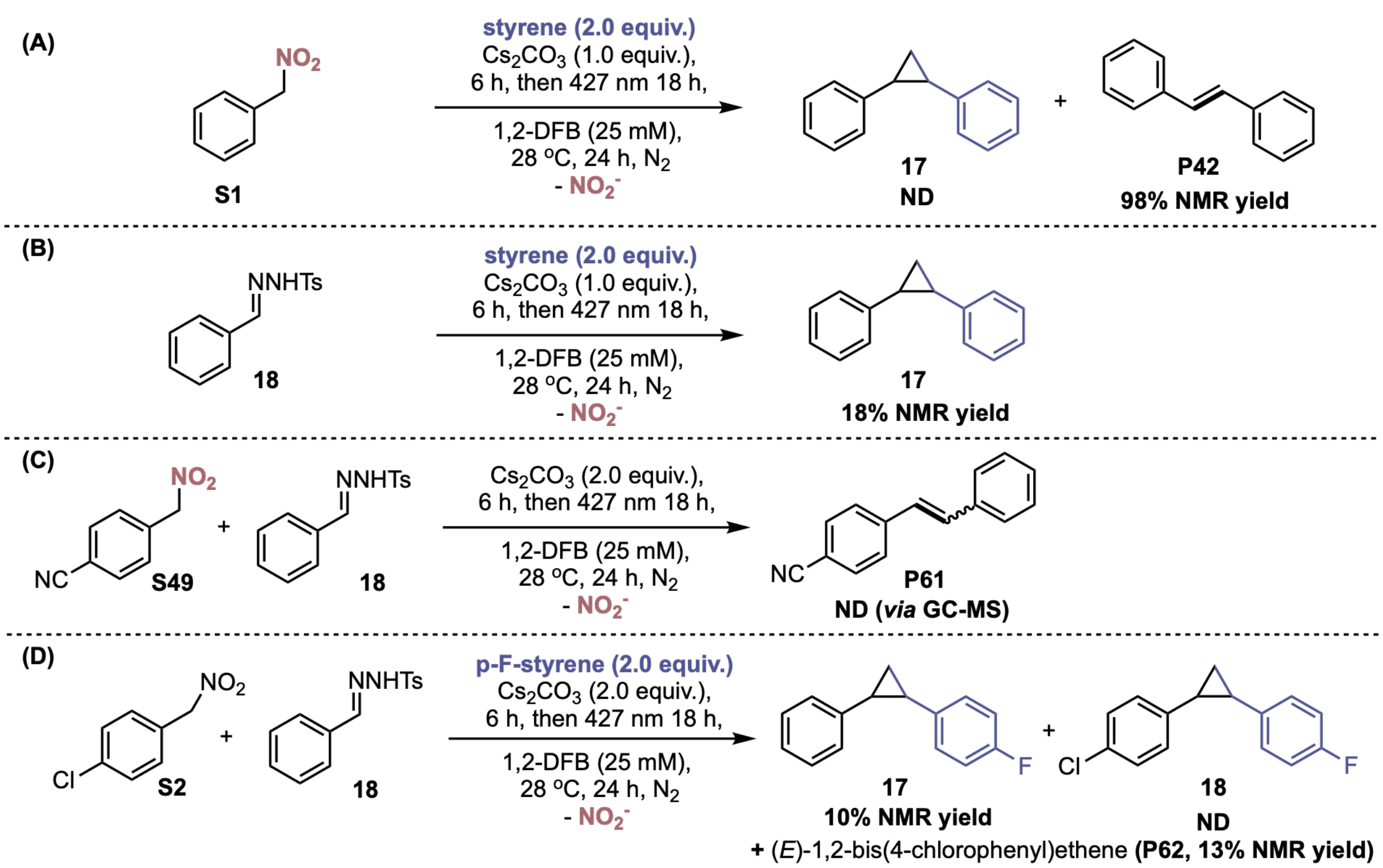

Fig. S11. Reactions of nitroalkanes and carbene precursors with varying carbene traps and with each other under the standard conditions.

Results. From the reactions depicted in **Fig. S11**, nucleophilic capture of carbenes by nitronates did not occur. Accordingly, carbene intermediates are unlikely to be operative under the reaction conditions.

^{1}H NMR (500 MHz) of the crude reaction from Entry (B) of Fig S11.

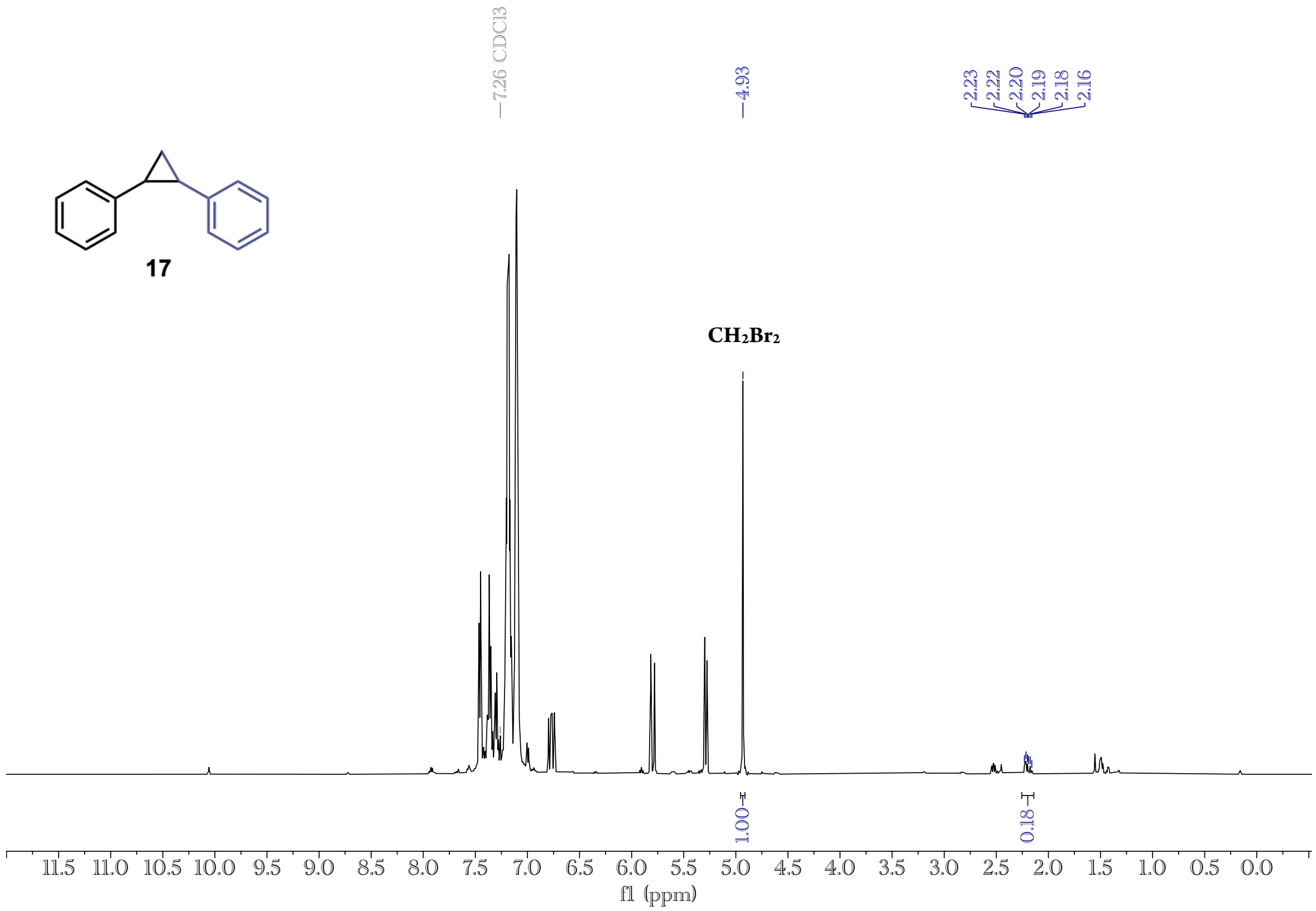

^{1}H NMR (500 MHz) of the crude reaction from Entry (D) of Fig S11.

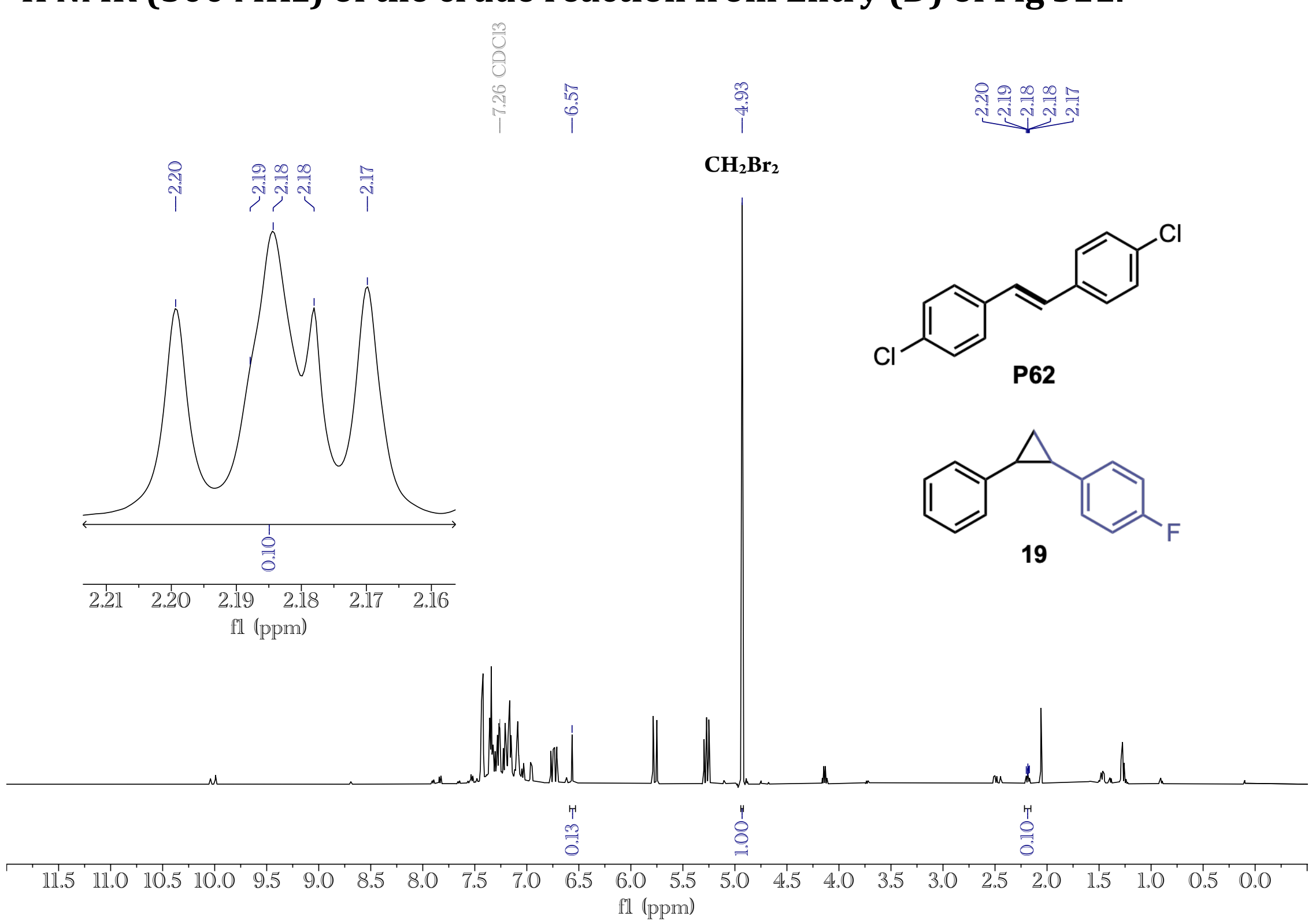

Isomerization of in-situ generated *E*-Stilbene derivatives

Photocatalytic isomerization

Reasoning. We questioned whether photocatalysts would isomerize the *E*-isomers of stilbene under photoirradiation to get *Z*-isomers.

Table S10. Photocatalytic Isomerization.

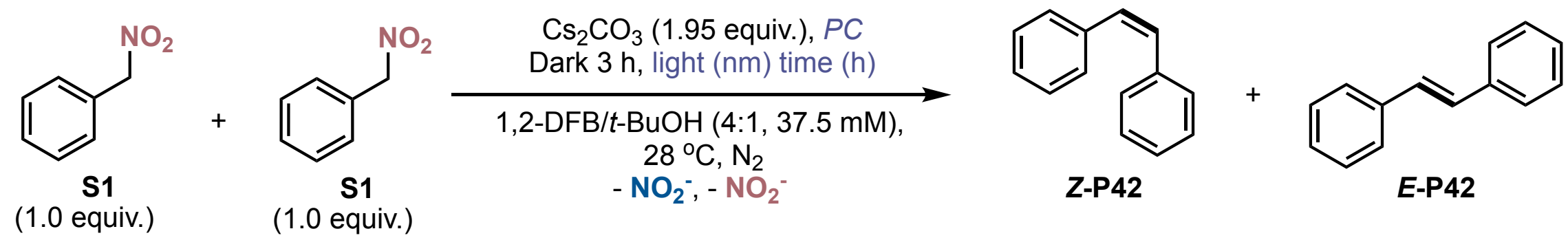

Entry	*PC*	*Light*	*Irradiation Time (h)*	*Yield (**Z-P42**)* [a]	*Yield (**E-P42**)* [a]
1	--	427 nm	2 h	ND	90%
2	--	427 nm	18 h	28%	57%
3	Ru(BPY)$_3$ [b]	427 nm	2 h	13%	80 %
4	Ru(BPY)$_3$ [b]	455 nm	2 h	12%	36 %
5	**Ru(BPY)$_3$** [b]	**427 nm**	**17 h**	**73%**	**12 %**
6	Ru(BPY)$_3$ [b]	455 nm	17 h	36%	30%
7	4CzIPN [c]	455 nm	17 h	78%	20%
8	Ir(Fppy)$_3$ [d]	455 nm	17 h	40%	3%
9	**[(iPr)CuCl]** [d]	**455 nm**	**17 h**	**90%**	**10%**

Reactions were performed on a 0.1 mmol scale. [a] Denotes ^{1}H NMR yields using CH_2Br_2 as an internal standard. [b] 5 mol %, [c] 10 mol %, [d] 2 mol % [d] 10 mol %.

Photoisomerization of Difluorostilbene

Reasoning. Data showing the GC ratio of difluorostilbene product (**P46**) after 30 minutes of irradiation from when **S41** was subjected to the reaction conditions.

Signal : TIC: AR-01-161-30min.D\data.ms

peak #	R.T. min	first scan	max scan	last scan	PK TY	peak height	corr. area	corr. % max.	% of total
1	4.583	819	824	850	M5	26911	593963	100.00%	56.062%
2	4.972	947	953	1001	M3	19451	465520	78.38%	43.938%

Sum of corrected areas: 1059483

V100.M Thu May 21 17:03:28 2026

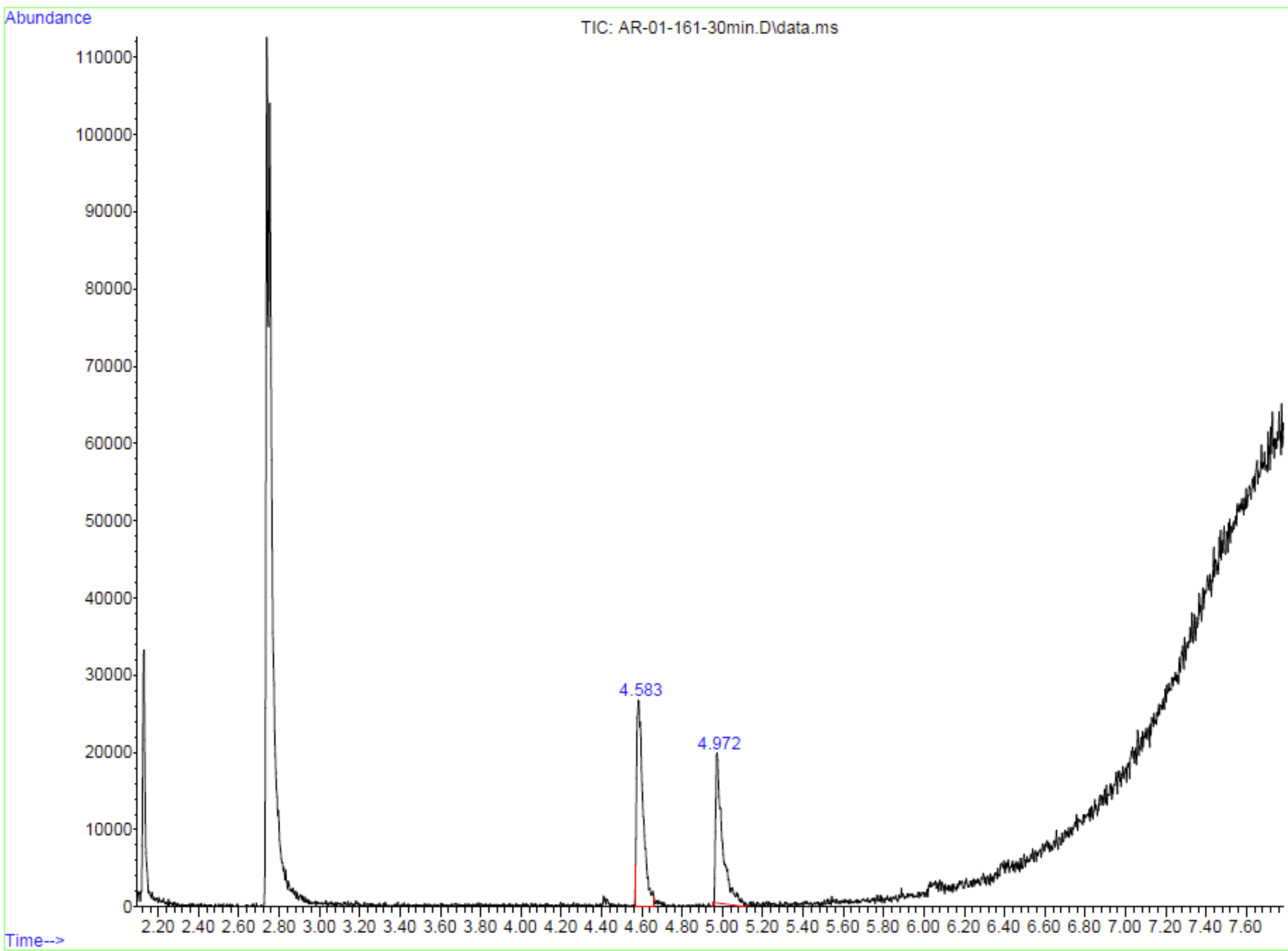

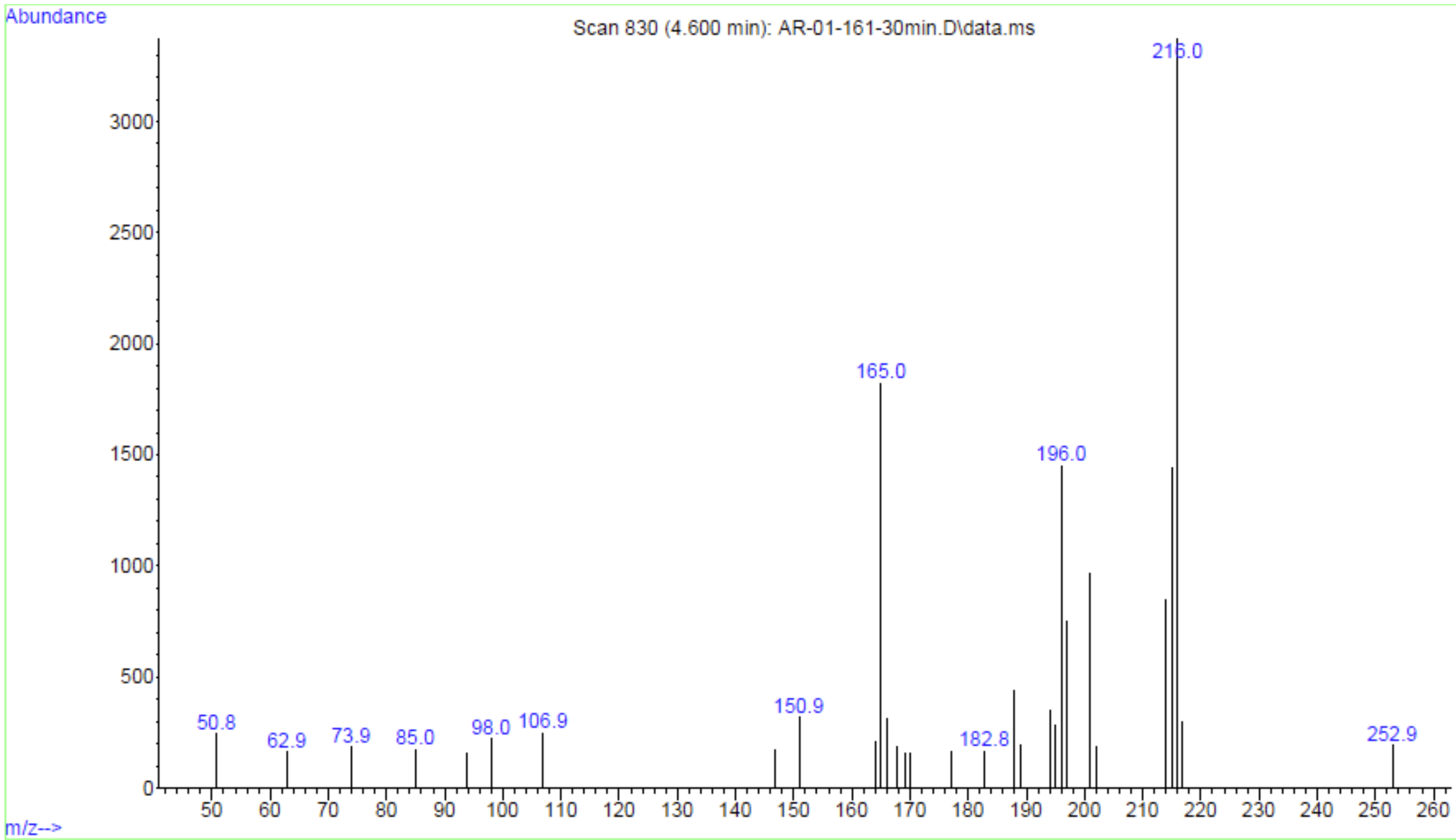

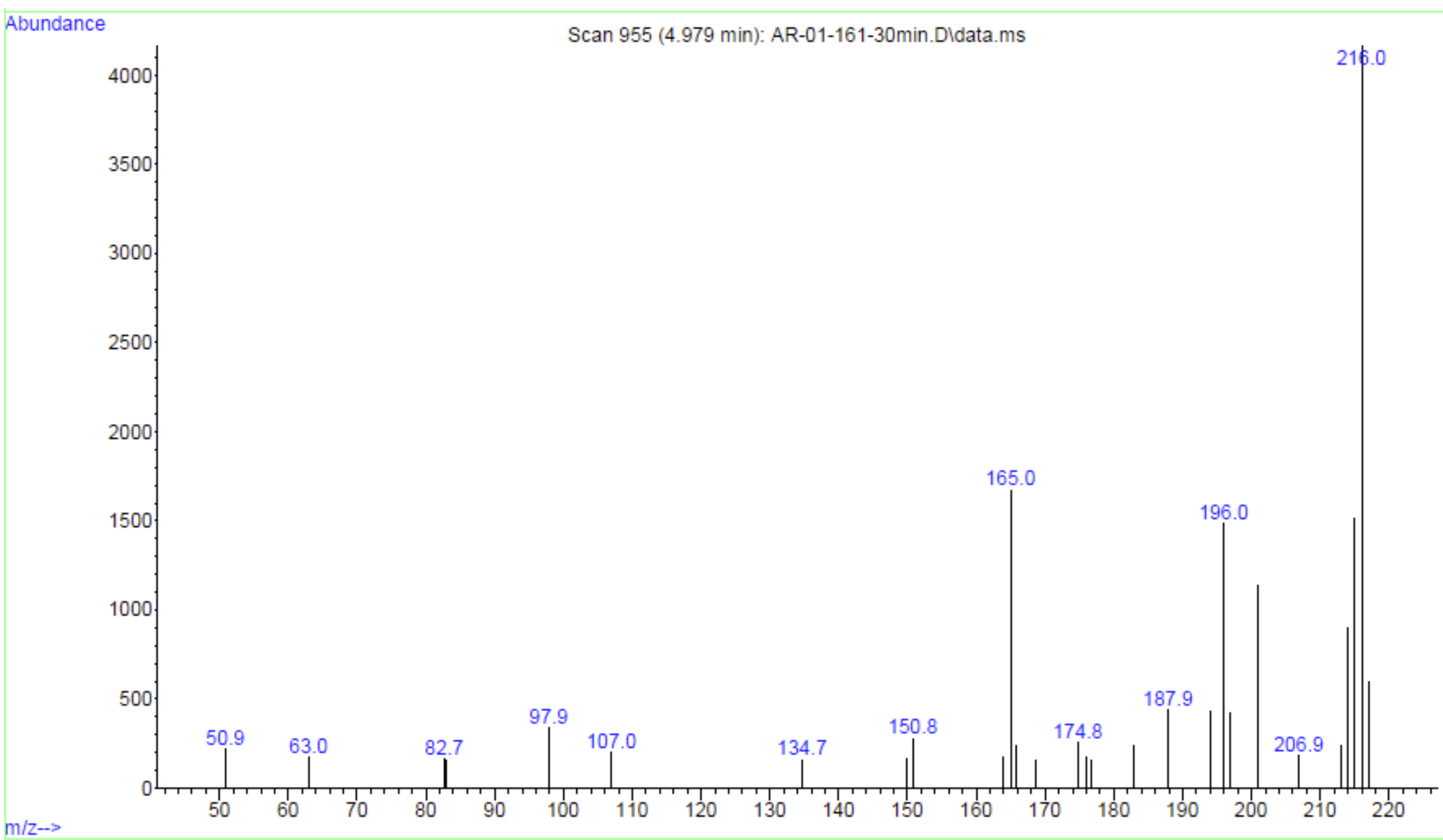

Results. We observed the E-isomer of the difluorostilbene (**P46**), indicating that the *E*-isomer is forming and then rapidly isomerizing to the *Z*-isomer upon prolonged irradiation, where after 2 h, we isolated high yields of only *Z* product.

Additive influence on Cross-Denitrative Olefination

Table S11. Additives employed

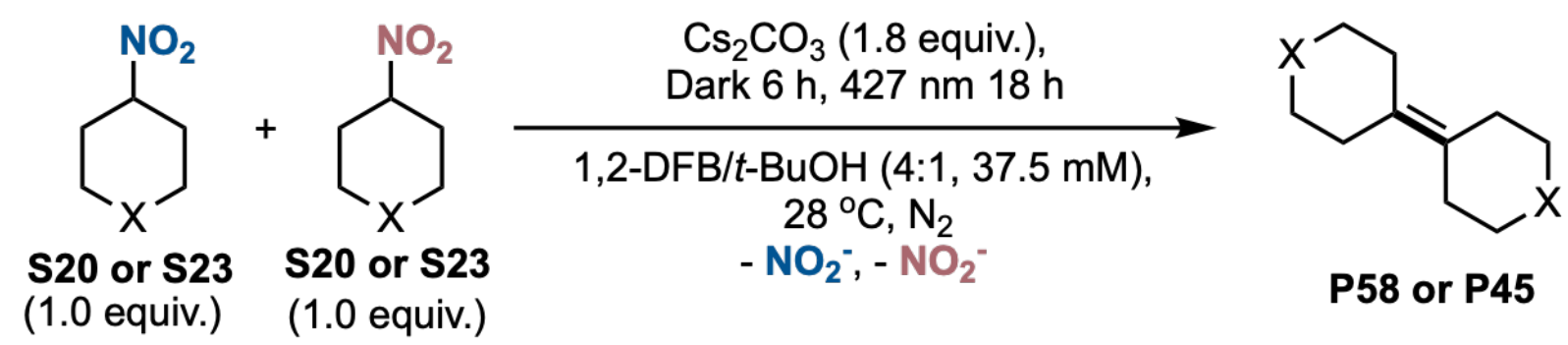

Entry	*Nitroalkane*	*Additive*	*Result (**P58** or **P45**)* [a]
1	**S20**	--	NR
2	**S20**	4-(dichloromethyl)benzonitrile (10 mol %)	GC-Detected (100% Conv of SM)
3	**S23**	--	53% yield
4	**S23**	4-(dichloromethyl)benzonitrile (10 mol %)	80% yield

Reactions were performed on a 0.1 mmol scale. [a] Denotes ^{1}H NMR yields using CH_2Br_2 as an internal standard.

Results. It was observed that 4-(dichloromethyl)benzonitrile (10 mol %) as an additive increased the yield of the reaction for **S23** and allowed product formation from aliphatic nitroalkanes that do not produce productive olefination product under the reaction conditions with **S20**. GC–MS spectra for the reaction of **S20** leading to **P58** is shown below.

```
Sample Name: AHB_JM_968
Misc Info  :
Vial Number: 22
```

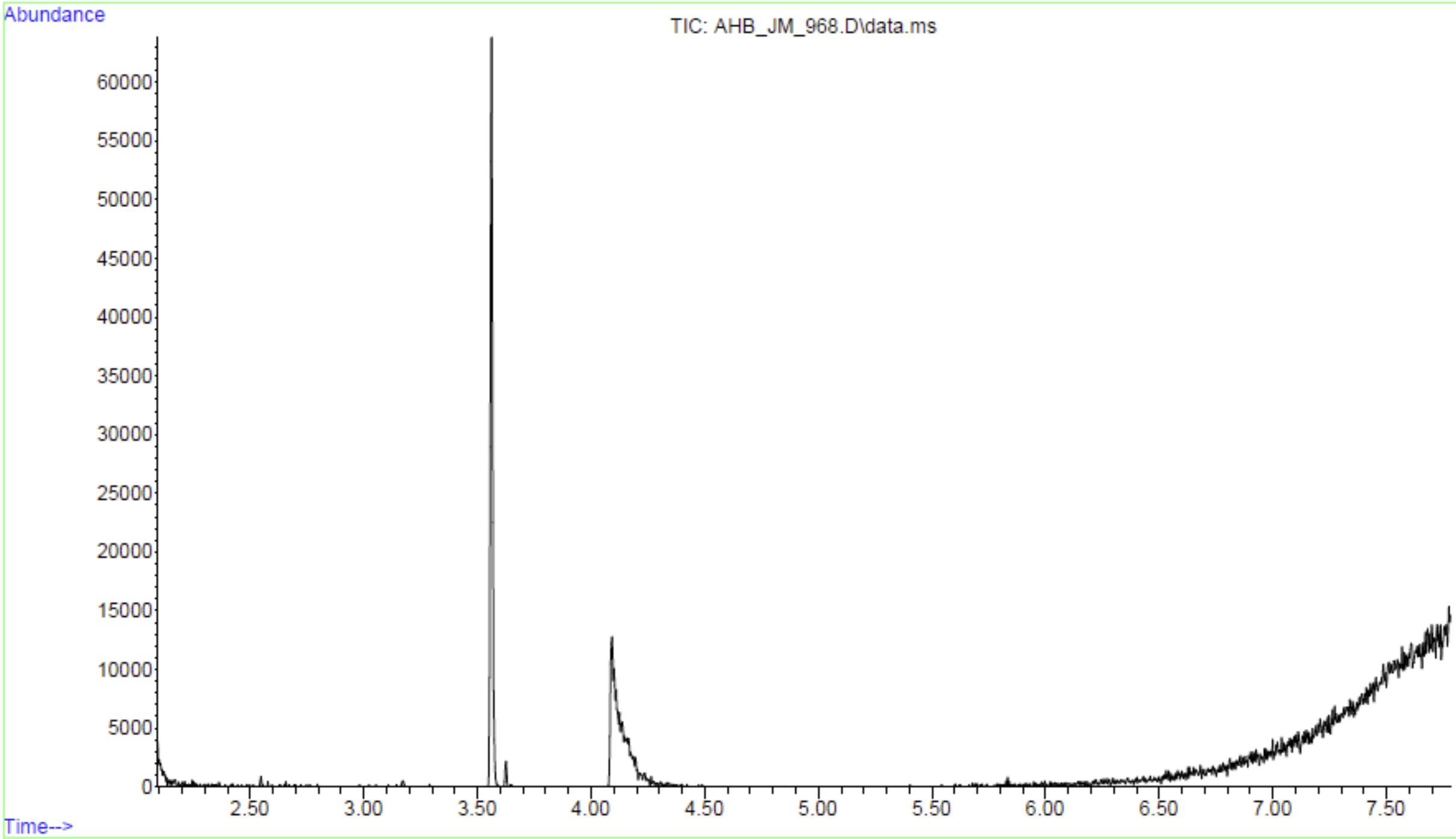

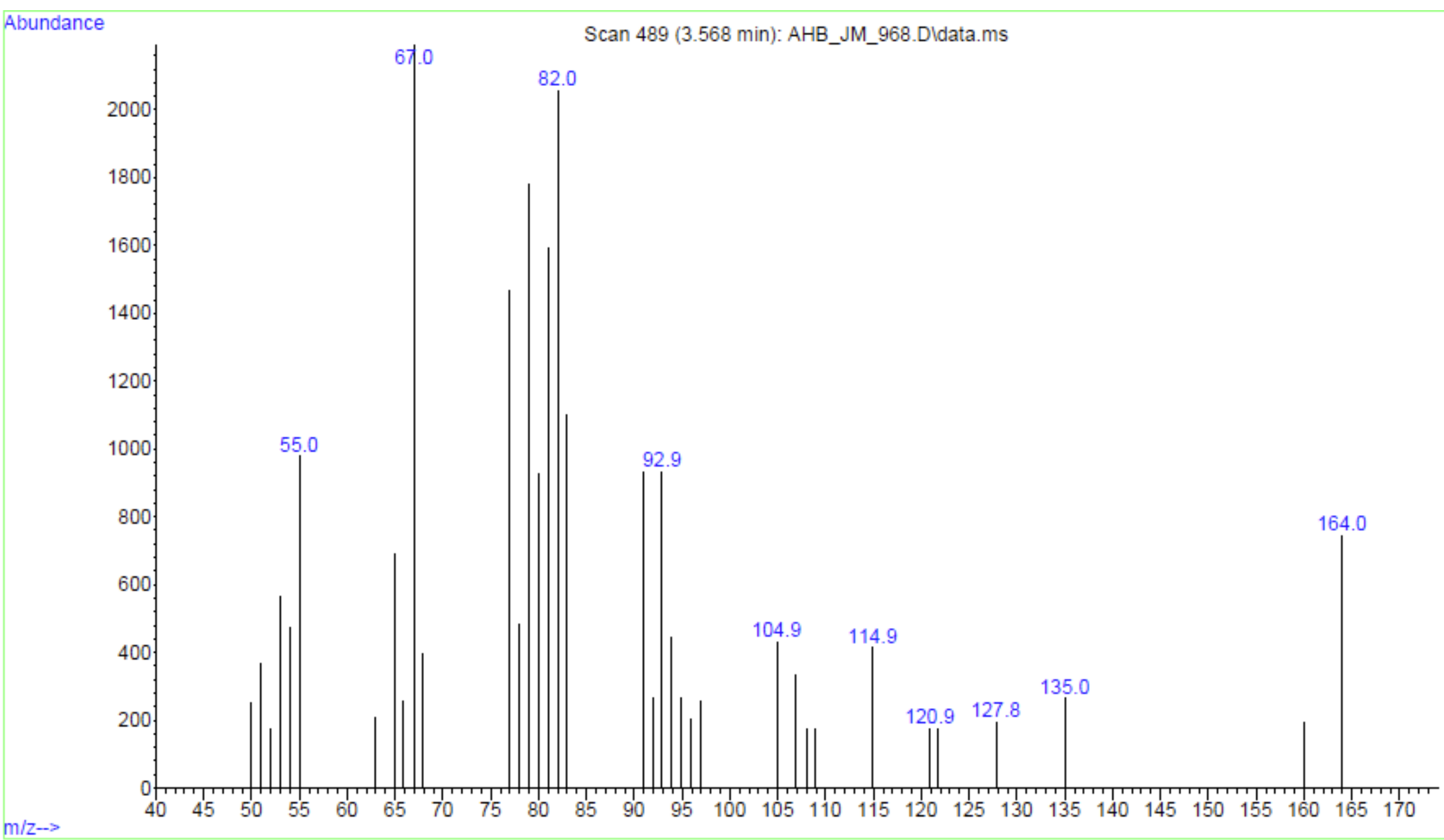

Nef byproducts from the reaction conditions

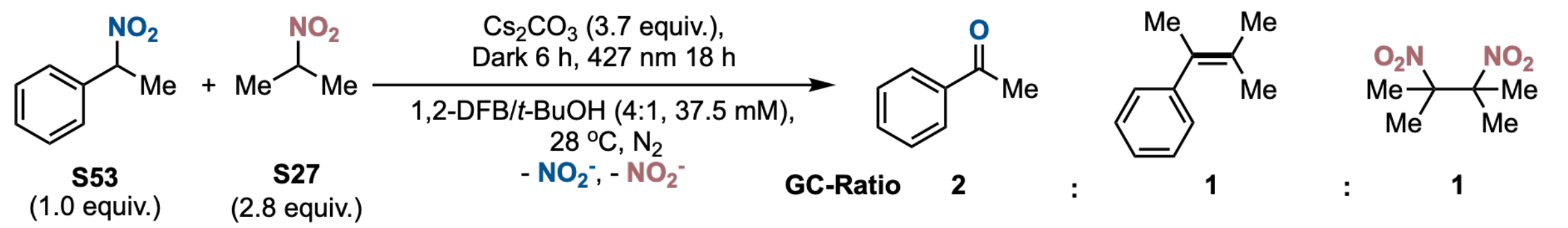

Results. Based on the GC-results below, significant Nef-product (acetophenone) was generated with minor amounts of olefination product and dinitroalkanes byproduct.

Sample Name: AHB_JM_967
Misc Info :
Vial Number: 21

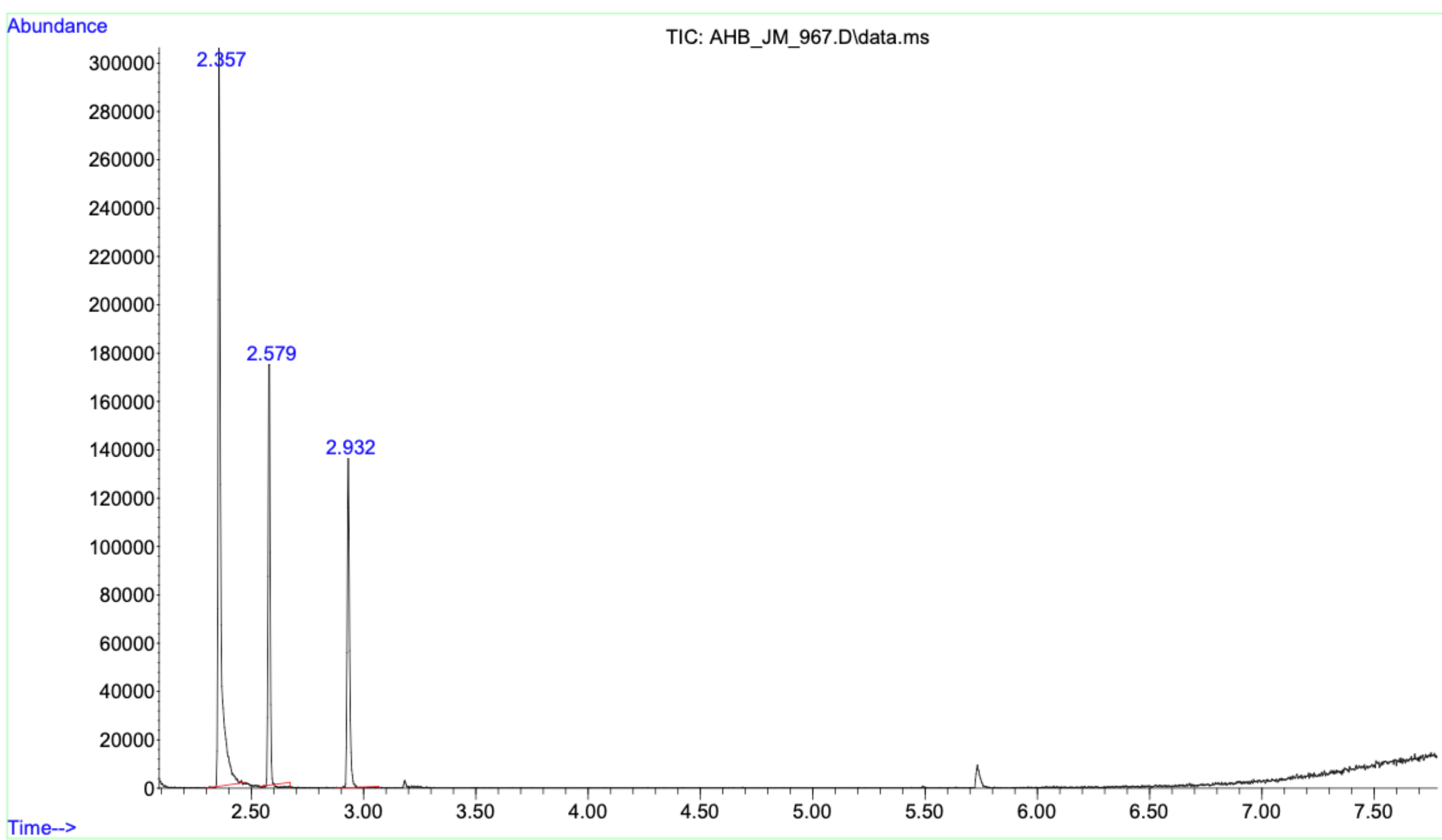

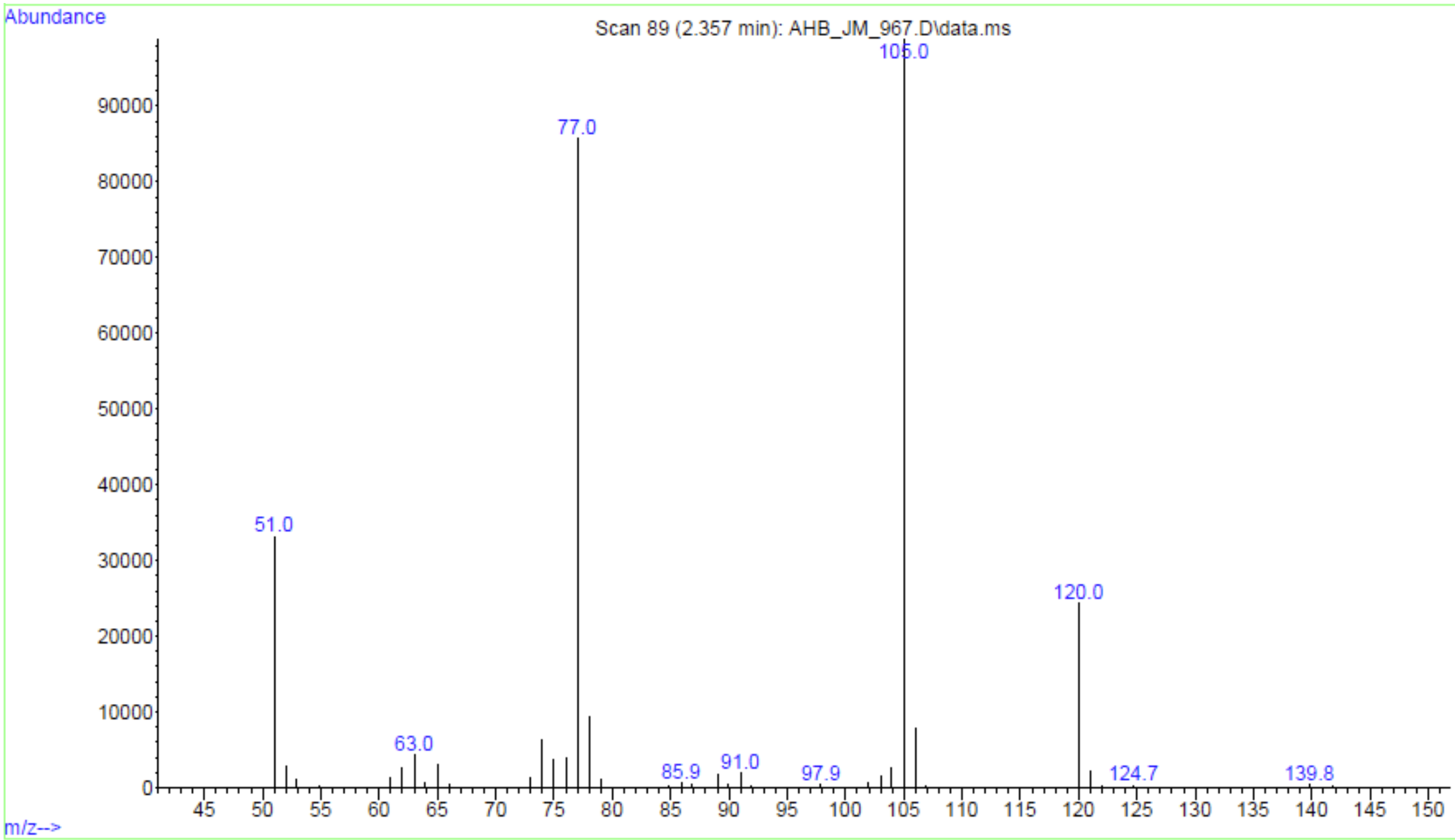
Abundance
Scan 89 (2.357 min): AHB_JM_967.D\data.ms
105.0
77.0
51.0
63.0
85.9
91.0
97.9
120.0
124.7
139.8
m/z-->

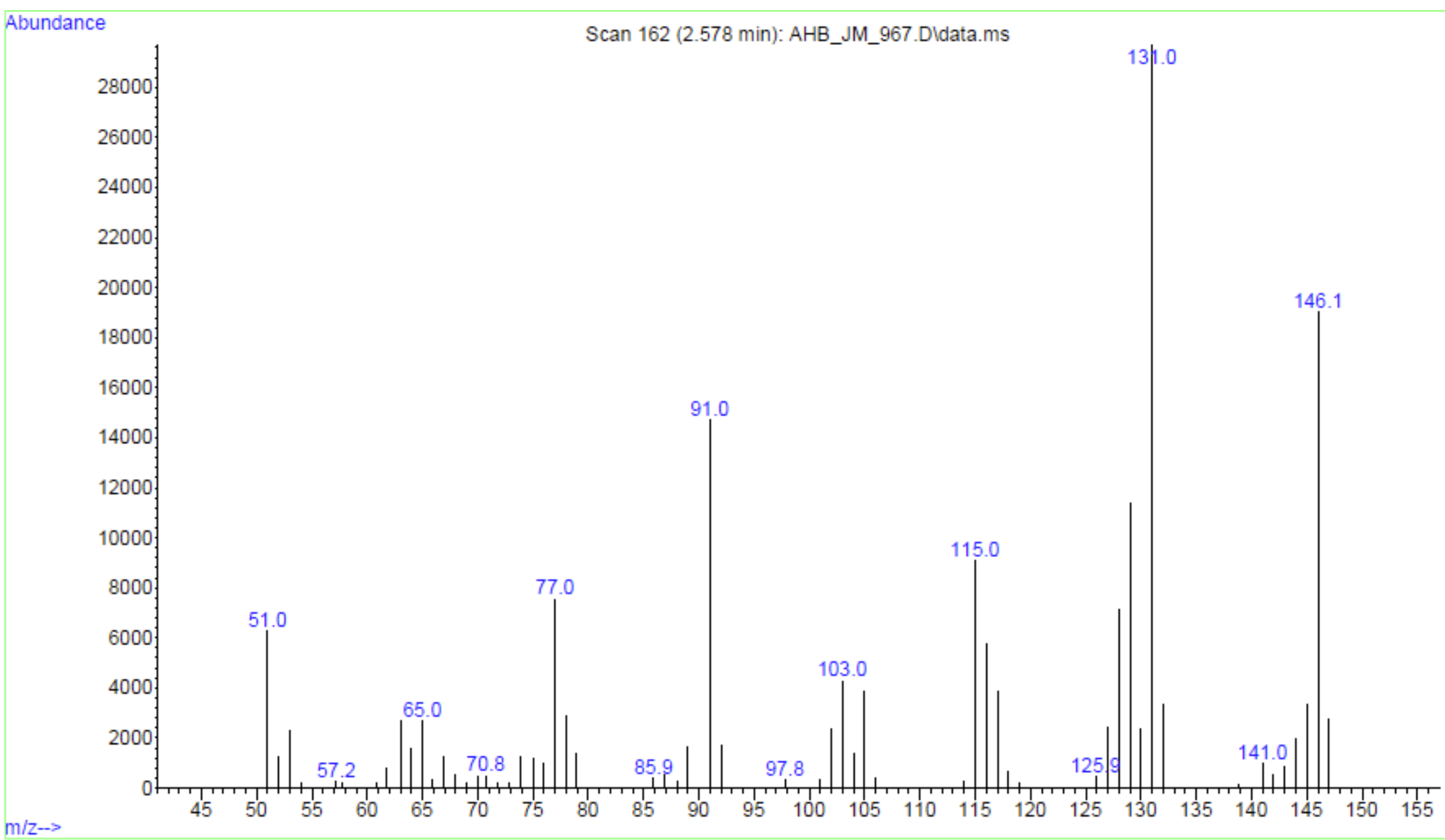
Abundance
Scan 162 (2.578 min): AHB_JM_967.D\data.ms
131.0
146.1
91.0
115.0
77.0
51.0
103.0
65.0
57.2
70.8
85.9
97.8
125.9
141.0
m/z-->

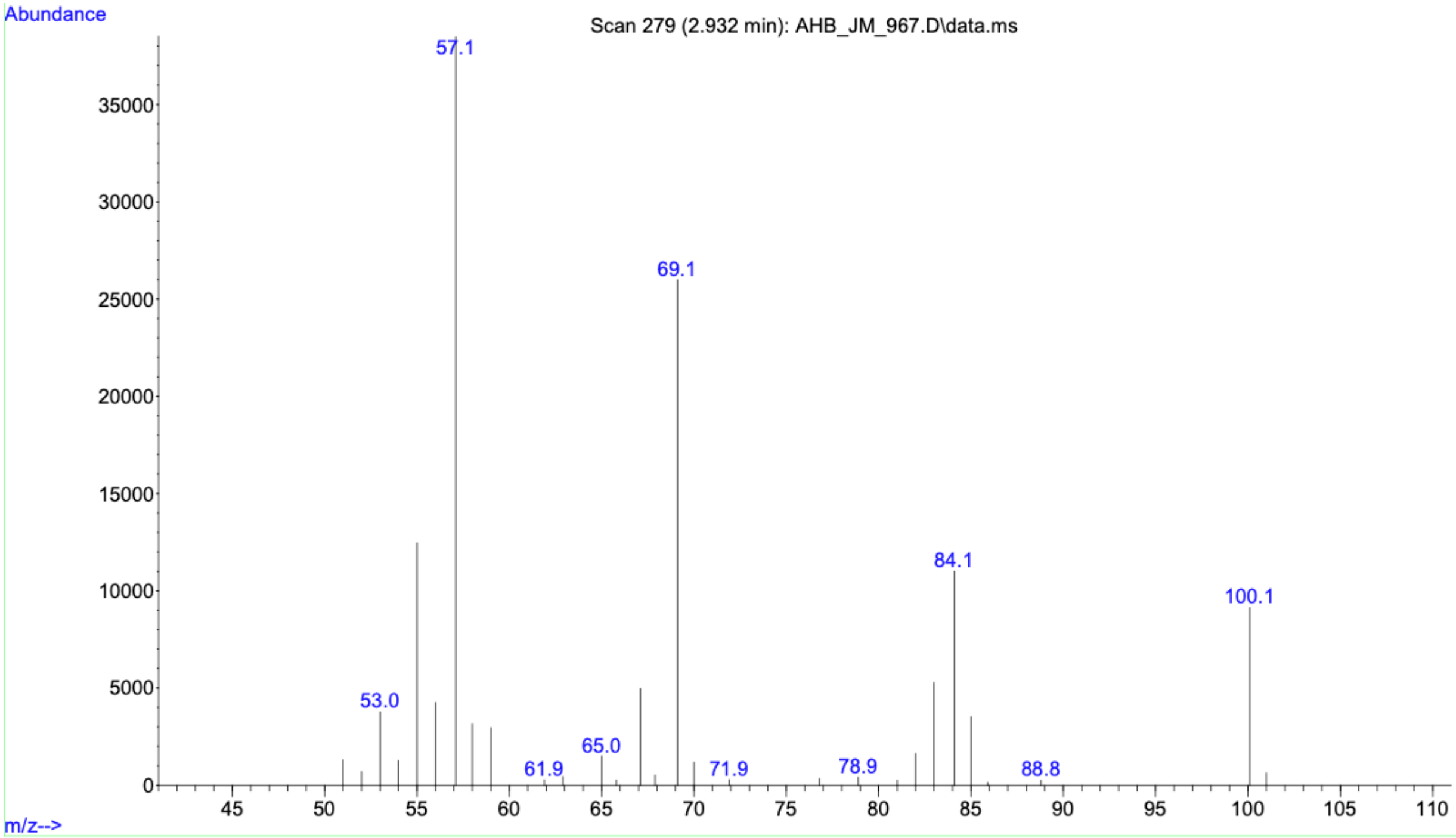
Abundance
Scan 279 (2.932 min): AHB_JM_967.D\data.ms
57.1
69.1
84.1
100.1
53.0
65.0
61.9
71.9
78.9
88.8
35000
30000
25000
20000
15000
10000
5000
0
45
50
55
60
65
70
75
80
85
90
95
100
105
110
m/z-->

Characterization of Alkenes Obtained from Denitrative Olefination

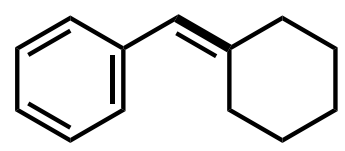

(cyclohexylidenemethyl)benzene (P1) [29]

Prepared following general procedure A using (nitromethyl)benzene (**S1**, 0.45 mmol, 1.0 equiv.), nitrocyclohexane (**S20**, 2.8 equiv.), and Cs_2CO_3 (3.7 equiv.) in 1,2-DFB/*t*-BuOH (4:1, 31.25 mM) at 28 °C. The title compound was isolated via flash column chromatography (100% Hexanes) as a colorless oil (62 mg, 80% yield).

^{1}H NMR (600 MHz, CD_2Cl_2) δ 7.32 – 7.26 (m, 2H), 7.21 – 7.14 (m, 3H), 6.23 (s, 1H), 2.42 – 2.34 (m, 2H), 2.30 – 2.20 (m, 2H), 1.71 – 1.58 (m, 4H), 1.58 – 1.53 (m, 2H).

^{13}C {^{1}H} NMR (151 MHz, CD_2Cl_2) δ 144.0, 138.9, 129.3, 128.4, 126.2, 122.2, 38.1, 29.8, 29.1, 28.3, 27.1.

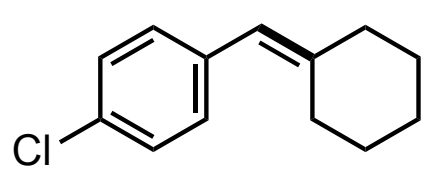

1-chloro-4-(cyclohexylidenemethyl)benzene (P2) [30]

Prepared following general procedure A using 1-chloro-4-(nitromethyl)benzene (**S2**, 0.45 mmol, 1.0 equiv.), nitrocyclohexane (**S20**, 2.8 equiv.), and Cs_2CO_3 (3.7 equiv.) in 1,2-DFB/*t*-BuOH (4:1, 31.25 mM) at 28 °C. The title compound was isolated via flash column chromatography (100% Hexanes) as a pale-yellow solid (67 mg, 72% yield).

TLC (SiO_2) R_f = 0.80 in Hexanes.

^{1}H NMR (400 MHz, CD_2Cl_2) δ 7.27 (d, *J* = 8.5 Hz, 2H), 7.14 (d, *J* = 8.8 Hz, 2H), 6.17 (s, 1H), 2.36 – 2.30 (m, 2H), 2.27 – 2.22 (m, 2H), 1.68 – 1.52 (m, 6H).

^{13}C {^{1}H} NMR (101 MHz, CD_2Cl_2) δ 145.0, 137.4, 131.6, 130.7, 128.4, 121.0, 38.0, 29.8, 29.0, 28.3, 27.0.

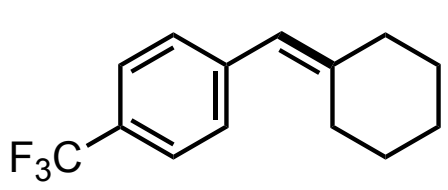

1-(cyclohexylidenemethyl)-4-(trifluoromethyl)benzene (P3) [30]

Prepared following general procedure A using 1-(nitromethyl)-4-(trifluoromethyl)benzene (**S3**, 0.45 mmol, 1.0 equiv.), nitrocyclohexane (**S20**, 2.8 equiv.), and Cs_2CO_3 (3.7 equiv.) in 1,2-DFB/*t*-BuOH (4:1, 31.25 mM) at 28 °C. The title compound was isolated via flash column chromatography (100% Hexanes) as a colorless oil (89 mg, 82% yield).

^{1}H NMR (500 MHz, CD_2Cl_2) δ 7.56 (d, *J* = 8.0 Hz, 2H), 7.32 (d, *J* = 7.9 Hz, 2H), 6.26 (s, 1H), 2.36 (t, *J* = 6.1 Hz, 2H), 2.28 (t, *J* = 5.9 Hz, 2H), 1.70 – 1.59 (m, 4H), 1.59 – 1.54 (m, 2H).

^{13}C {^{1}H} NMR (151 MHz, CD_2Cl_2) δ 146.5, 142.8, 129.6, 127.9 (q, *J* = 32.1 Hz), 125.3 (q, *J* = 3.9 Hz), 125.0 (q, *J* = 271.5 Hz), 121.1, 38.1, 29.9, 29.1, 28.3, 27.0.

^{19}F {^{1}H} NMR (471 MHz, CD_2Cl_2) δ -62.61.

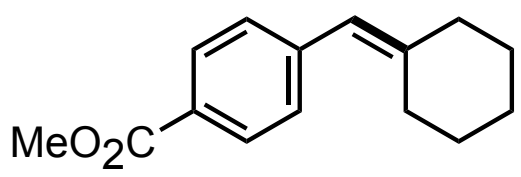

Methyl 4-(cyclohexylidenemethyl)benzoate (P4) [31]

Prepared following general procedure A using methyl 4-(nitromethyl)benzoate (**S4**, 0.45 mmol, 1.0 equiv.), nitrocyclohexane (**S20**, 2.8 equiv.), and Cs_2CO_3 (3.7 equiv.) in 1,2-DFB/*t*-BuOH (4:1, 31.25 mM) at 28 °C. The title compound was isolated via flash column chromatography (0–10% EtOAc/Hexanes) as a colorless oil (95 mg, 92% yield).

TLC (SiO_2) R_f = 0.58 in Hexanes.

^{1}H NMR (400 MHz, CD_2Cl_2) δ 7.94 (d, *J* = 8.3 Hz, 2H), 7.27 (d, *J* = 8.0 Hz, 2H), 6.26 (s, 1H), 3.87 (s, 3H), 2.42 – 2.35 (m, 2H), 2.33 – 2.22 (m, 2H), 1.69 – 1.54 (m, 6H).

^{13}C {^{1}H} NMR (101 MHz, CD_2Cl_2) δ 167.2, 146.5, 143.7, 129.6, 129.2, 127.9, 121.5, 52.2, 38.2, 30.0, 29.1, 28.3, 27.0.

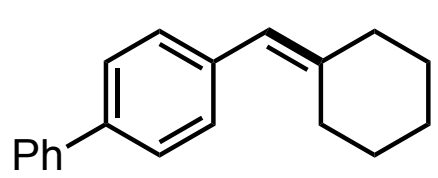

4-(cyclohexylidenemethyl)-1,1'-biphenyl (P5) [32]

Prepared following general procedure A using 4-(nitromethyl)-1,1'-biphenyl (**S5**, 0.45 mmol, 1.0 equiv.), nitrocyclohexane (**S20**, 2.8 equiv.), and Cs_2CO_3 (3.7 equiv.) in 1,2-DFB/*t*-BuOH (4:1, 31.25 mM) at 28 °C. The title compound was isolated via flash column chromatography (100% Hexanes) as a pale-yellow oil (75 mg, 67% yield).

TLC (SiO_2) R_f = 0.37 in Hexanes.

^{1}H NMR (400 MHz, $CDCl_3$) δ 7.67 – 7.59 (m, 2H), 7.59 – 7.53 (m, 2H), 7.49 – 7.40 (m, 2H), 7.39 – 7.32 (m, 1H), 7.32 – 7.28 (m, 2H), 6.27 (s, 1H), 2.44 (t, *J* = 5.8 Hz, 2H), 2.30 (t, *J* = 5.5 Hz, 2H), 1.70 – 1.59 (m, 6H).

^{13}C {^{1}H} NMR (101 MHz, $CDCl_3$) δ 144.0, 141.2, 138.7, 137.6, 129.5, 128.9, 127.2, 127.1, 126.9, 121.7, 37.9, 29.7, 28.8, 28.0, 26.8.

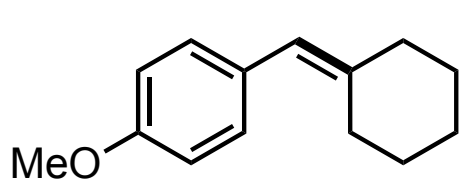

1-(cyclohexylidenemethyl)-4-methoxybenzene (P6)

Following general procedure A using 1-methoxy-4-(nitromethyl)benzene (**S6**, 0.45 mmol, 1.0 equiv.), nitrocyclohexane (**S20**, 2.8 equiv.), and Cs_2CO_3 (3.7 equiv.) in 1,2-DFB/*t*-BuOH (4:1, 31.25 mM) at 28 °C. The title compound was not produced in any detectable yield.

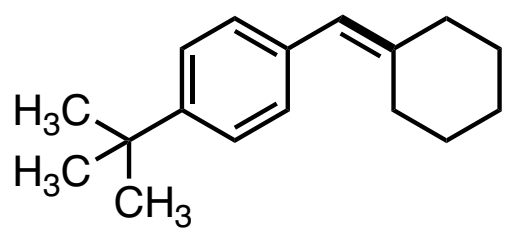

1-(*tert*-butyl)-4-(cyclohexylidenemethyl)benzene (P7) [33]

Prepared following general procedure A using 1-(*tert*-butyl)-4-(nitromethyl)benzene (**S7**, 0.45 mmol, 1.0 equiv.), nitrocyclohexane (2.8 equiv.), and Cs_2CO_3 (3.7 equiv.) in 1,2-DFB/*t*-BuOH (4:1, 31.25 mM) at 28 °C. The title compound was isolated via flash column chromatography (100% Hexanes) as a colorless oil (23 mg, 22% yield).

TLC (SiO_2) R_f = 0.61 in Hexanes.

^{1}H NMR (400 MHz, $CDCl_3$) δ 7.37 – 7.29 (m, 2H), 7.18 – 7.11 (m, 2H), 6.19 (s, 1H), 2.53 – 2.34 (m, 2H), 2.25 (t, *J* = 5.5 Hz, 2H), 1.72 – 1.48 (m, 6H), 1.32 (s, 9H).

^{13}C {^{1}H} NMR (101 MHz, $CDCl_3$) δ 148.7, 143.1, 135.7, 128.7, 125.1, 121.8, 37.8, 34.6, 31.5, 29.6, 28.8, 28.0, 26.9.

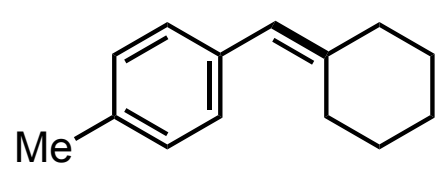

1-(cyclohexylidenemethyl)-4-methylbenzene (P8) [34]

Prepared following general procedure A using 1-methyl-4-(nitromethyl)benzene (**S8**, 0.45 mmol, 1.0 equiv.), nitrocyclohexane (**S20**, 2.8 equiv.), and Cs_2CO_3 (3.7 equiv.) in 1,2-DFB/*t*-BuOH (4:1, 31.25 mM) at 28 °C. The title compound was isolated via flash column chromatography (100% Hexanes) as a colorless oil (29 mg, 34% yield).

TLC (SiO_2) R_f = 0.74 in Hexanes.

^{1}H NMR (400 MHz, $CDCl_3$) δ 7.14 – 7.07 (m, 4H), 6.19 (s, 1H), 2.37 (t, *J* = 6.6 Hz, 2H), 2.33 (s, 3H), 2.25 (t, *J* = 6.1 Hz, 2H), 1.68 – 1.50 (m, 6H).

^{13}C {^{1}H} NMR (101 MHz, $CDCl_3$) δ 143.0, 135.6, 135.5, 129.0, 128.9, 121.9, 37.8, 29.6, 28.8, 28.0, 26.9, 21.3.

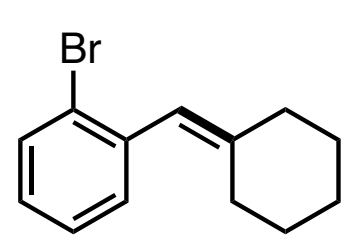

1-bromo-2-(cyclohexylidenemethyl)benzene (P9)

Prepared following general procedure A using 1-bromo-2-(nitromethyl)benzene (**S9**, 0.45 mmol, 1.0 equiv.), nitrocyclohexane (**S20**, 2.8 equiv.), and Cs_2CO_3 (3.7 equiv.) in 1,2-DFB/*t*-BuOH (4:1, 31.25 mM) at 28 °C. The title compound was isolated via flash column chromatography (100% Hexanes) as a colorless oil (92 mg, 82% yield).

TLC (SiO_2) R_f = 0.68 in Hexanes.

^{1}H NMR (400 MHz, $CDCl_3$) δ 7.56 (dd, *J* = 8.0, 1.2 Hz, 1H), 7.30 – 7.15 (m, 2H), 7.09 – 7.03 (m, 1H), 6.17 (s, 1H), 2.39 – 2.27 (m, 2H), 2.27 – 2.15 (m, 2H), 1.69 – 1.53 (m, 6H).

^{13}C {^{1}H} NMR (101 MHz, $CDCl_3$) δ 144.6, 138.6, 132.5, 131.2, 127.8, 126.9, 124.5, 121.7, 37.4, 29.9, 28.8, 28.0, 26.8.

HRMS (ESI-TOF): *m/z* calculated for $C_{13}H_{15}Br_2$ $[M+Br]^-$ = 330.9526, found 330.9511.

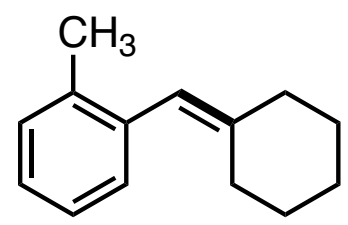

1-(cyclohexylidenemethyl)-2-methylbenzene (P10)

Prepared following general procedure A using 1-methyl-2-(nitromethyl)benzene (**S10**, 0.45 mmol, 1.0 equiv.), nitrocyclohexane (**S20**, 2.8 equiv.), and Cs_2CO_3 (3.7 equiv.) in 1,2-DFB/*t*-BuOH (4:1, 31.25 mM) at 28 °C. The title compound was isolated via flash column chromatography (100% Hexanes) as a colorless oil (62 mg, 74% yield).

TLC (SiO_2) R_f = 0.63 in Hexanes

^{1}H NMR (400 MHz, $CDCl_3$) δ 7.20 – 6.99 (m, 4H), 6.16 (s, 1H), 2.33 – 2.26 (m, 2H), 2.25 (s, 3H), 2.21 – 2.14 (m, 2H), 1.68 – 1.58 (m, 4H), 1.56 – 1.46 (m, 2H).

^{13}C {^{1}H} NMR (101 MHz, $CDCl_3$) δ 143.1, 137.8, 136.6, 129.7, 129.6, 126.4, 125.3, 121.0, 37.5, 29.8, 29.0, 28.1, 26.9, 20.1.

HRMS (ESI-TOF): *m/z* calculated for $C_{14}H_{17}$ $[M-H]^-$ = 185.1336, found 185.1332.

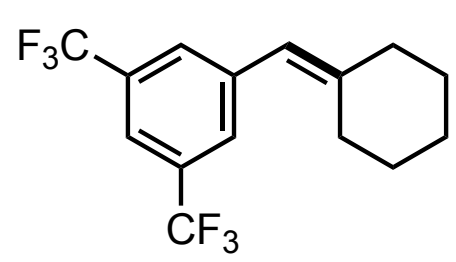

1-(cyclohexylidenemethyl)-3,5-bis(trifluoromethyl)benzene (P11)

Prepared following general procedure A using 1-(nitromethyl)-3,5-bis(trifluoromethyl)benzene (**S11**, 0.45 mmol, 1.0 equiv.), nitrocyclohexane (**S20**, 2.8 equiv.), and Cs_2CO_3 (3.7 equiv.) in 1,2-DFB/*t*-BuOH (4:1, 31.25 mM) at 28 °C. Due to inseparability of the title compound from byproducts by chromatography, ^{1}H NMR yield determined using CH_2Br_2 as an internal standard 70% NMR yield.

TLC (SiO_2) R_f = 0.925 in 4:1 Hexanes:EtOAc.

^{1}H NMR (500 MHz, $CDCl_3$) (crude) δ 6.23 (s, 1H, olefin proton).

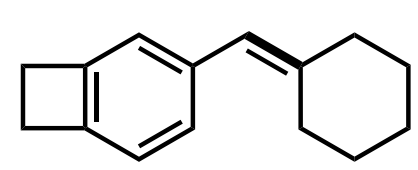

3-(cyclohexylidenemethyl)bicyclo[4.2.0]octa-1(6),2,4-triene (P12)

Prepared following general procedure A using 3-(nitromethyl)bicyclo[4.2.0]octa-1(6),2,4-triene (**S12**, 0.45 mmol, 1.0 equiv.), nitrocyclohexane (**S20**, 2.8 equiv.), and Cs_2CO_3 (3.7 equiv.) in 1,2-DFB/*t*-BuOH (4:1, 31.25 mM) at 28 °C. The title compound was isolated via flash column chromatography (100% Hexanes) as a colorless oil (66 mg, 75% yield).

TLC (SiO_2) R_f = 0.60 in Hexanes

^{1}H NMR (500 MHz, $CDCl_3$) δ 7.02 – 6.98 (m, 2H), 6.90 (s, 1H), 6.22 (s, 1H), 3.16 (s, 4H), 2.35 (t, *J* = 6.0 Hz, 2H), 2.24 (t, *J* = 5.8 Hz, 2H), 1.64 – 1.53 (m, 6H).

^{13}**C {^{1}H} NMR** (126 MHz, $CDCl_3$) δ 145.6, 143.5, 142.6, 137.2, 127.8, 123.1, 122.9, 122.2, 37.8, 29.61, 29.59, 29.5, 28.8, 28.0, 26.9.
HRMS (ESI-TOF): *m/z* calculated for $C_{17}H_{19}O$ $[M+CH_3CO_2-(H_2O)]^-$ = 239.1441, found 239.1434.

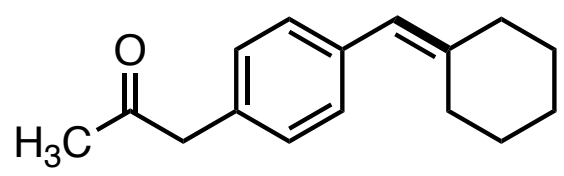

1-(4-(cyclohexylidenemethyl)phenyl)propan-2-one (P13)
Prepared following general procedure A using 1-(4-(nitromethyl)phenyl)propan-2-one (**S13**, 0.45 mmol, 1.0 equiv.), nitrocyclohexane (**S20**, 2.8 equiv.), and Cs_2CO_3 (3.7 equiv.) in 1,2-DFB/*t*-BuOH (4:1, 31.25 mM) at 28 °C. The title compound was isolated via flash column chromatography (8:1:1 Hexanes/DCM/Diethyl Ether) as a colorless oil (80.7 mg, 78% yield).
TLC (SiO_2) R_f = 0.31 in 8:1:1 Hexanes:DCM:Diethyl Ether.
^{1}**H NMR** (400 MHz, $CDCl_3$) δ 7.18 – 7.12 (m, 4H), 6.19 (s, 1H), 3.67 (s, 2H), 2.44 – 2.32 (m, 2H), 2.31 – 2.22 (m, 2H), 2.16 (s, 3H), 1.66 – 1.53 (m, 6H).
^{13}**C {^{1}H} NMR** (101 MHz, $CDCl_3$) δ 206.8, 143.8, 137.4, 131.8, 129.5, 129.2, 121.6, 50.9, 37.8, 29.6, 29.4, 28.8, 28.0, 26.8.
HRMS (ESI-TOF): *m/z* calculated for $C_{17}H_{21}O_3$ $[M+HCO_2]^-$ = 273.1496, found 273.1498.

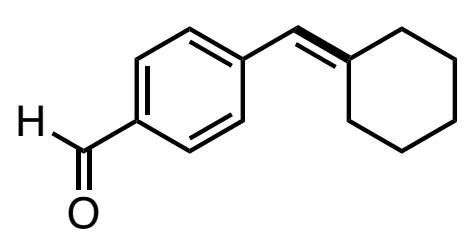

4-(cyclohexylidenemethyl)benzaldehyde (P14)
Prepared following general procedure A using 4-(nitromethyl)benzaldehyde (**S14**, 0.45 mmol, 1.0 equiv.), nitrocyclohexane (**S20**, 2.8 equiv.), and Cs_2CO_3 (3.7 equiv.) in 1,2-DFB/*t*-BuOH (4:1, 31.25 mM) at 28 °C. The title compound was isolated via flash column chromatography (10–30% DCM/Hexanes) as a colorless oil (36 mg, 40% yield).
TLC (SiO_2) R_f = 0.21 in 7:3 Hexanes:DCM
^{1}**H NMR** (400 MHz, $CDCl_3$) δ 9.97 (s, 1H), 7.81 (d, *J* = 8.2 Hz, 2H), 7.35 (d, *J* = 8.1 Hz, 2H), 6.26 (s, 1H), 2.42 – 2.36 (m, 2H), 2.32 – 2.25 (m, 2H), 1.67 – 1.57 (m, 6H).
^{13}**C {^{1}H} NMR** (101 MHz, $CDCl_3$) δ 192.0, 146.9, 145.2, 134.2, 129.8, 129.6, 121.4, 38.0, 29.9, 28.7, 28.0, 26.7.
HRMS (ESI-TOF): *m/z* calculated for $C_{16}H_{19}O_3$ $[M+CH_3CO_2]^-$ = 259.1340, found 259.1344.

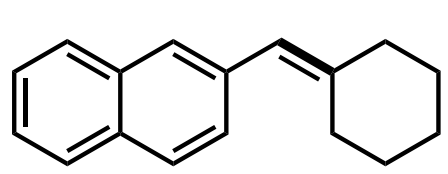

2-(cyclohexylidenemethyl)naphthalene (P15) [35]
Prepared following general procedure A using 2-(nitromethyl)naphthalene (**S15**, 0.267 mmol, 1.0 equiv.), nitrocyclohexane (**S20**, 2.8 equiv.), and Cs_2CO_3 (3.7 equiv.) in 1,2-DFB/*t*-

BuOH (4:1, 31.25 mM) at 28 °C. The title compound was isolated via flash column chromatography (100% Hexanes) as a pale-yellow oil (16 mg, 26% yield).
TLC (SiO_2) R_f = 0.53 in Hexanes
^{1}H NMR (500 MHz, $CDCl_3$) δ 7.87 – 7.72 (m, 3H), 7.64 (s, 1H), 7.44 – 7.39 (m, 2H), 7.36 (dd, *J* = 8.5, 1.7 Hz, 1H), 6.38 (s, 1H), 2.46 (t, *J* = 5.9 Hz, 2H), 2.32 (t, *J* = 6.0 Hz, 2H), 1.69 – 1.56 (m, 6H).
^{13}C {^{1}H} NMR (101 MHz, $CDCl_3$) δ 144.2, 136.1, 133.6, 132.0, 127.92, 127.86, 127.7, 127.5, 127.3, 126.0, 125.5, 122.1, 37.9, 29.8, 28.8, 28.1, 26.9.

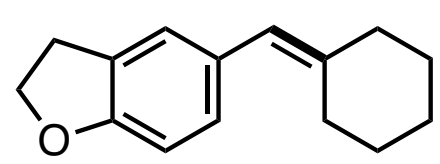

5-(cyclohexylidenemethyl)-2,3-dihydrobenzofuran (P16)
Prepared following general procedure A using 5-(nitromethyl)-2,3-dihydrobenzofuran (**S16**, 0.45 mmol, 1.0 equiv.), nitrocyclohexane (**S20**, 2.8 equiv.), and Cs_2CO_3 (3.7 equiv.) in 1,2-DFB/*t*-BuOH (4:1, 31.25 mM) at 28 °C. The title compound was isolated via flash column chromatography (100:0:0 to 98:1:1 Hexanes:Diethyl Ether:DCM) as a colorless oil (50 mg, 52% yield).
TLC (SiO_2) R_f = 0.34 in 98:1:1 Hexanes:Diethyl Ether:DCM.
^{1}H NMR (400 MHz, $CDCl_3$) δ 7.04 (s, 1H), 6.96 – 6.93 (m, 1H), 6.73 (d, *J* = 8.2 Hz, 1H), 6.16 (s, 1H), 4.56 (t, *J* = 8.7 Hz, 2H), 3.19 (t, *J* = 8.6 Hz, 2H), 2.38 – 2.34 (m, 2H), 2.25 – 2.22 (m, 2H), 1.63 – 1.54 (m, 6H).
^{13}C {^{1}H} NMR (101 MHz, $CDCl_3$) δ 158.3, 142.0, 131.0, 128.8, 126.8, 125.5, 121.8, 108.9, 71.3, 37.7, 30.0, 29.6, 28.8, 28.0, 26.9.
HRMS (ESI-TOF): *m/z* calculated for $C_{15}H_{17}O$ $[M-H]^-$ = 213.1285, found 213.1295.

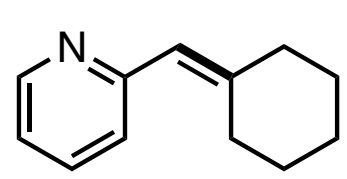

2-(cyclohexylidenemethyl)pyridine (P17) [36]
Prepared following general procedure A using 2-(nitromethyl)pyridine (**S17**, 0.23 mmol, 1.0 equiv.), nitrocyclohexane (**S20**, 2.8 equiv.), and Cs_2CO_3 (3.7 equiv.) in 1,2-DFB/*t*-BuOH (4:1, 31.25 mM) at 28 °C. The title compound was isolated via flash column chromatography (0–10% EtOAc/Hexanes) as a colorless oil (22 mg, 56% yield).
TLC (SiO_2) R_f = 0.47 in 4:1 Hexanes:EtOAc.
^{1}H NMR (400 MHz, $CDCl_3$) δ 8.56 (ddd, *J* = 4.9, 1.9, 0.9 Hz, 1H), 7.60 (td, *J* = 7.7, 1.9 Hz, 1H), 7.16 (d, *J* = 7.9 Hz, 1H), 7.05 (ddd, *J* = 7.5, 4.9, 1.2 Hz, 1H), 6.28 (s, 1H), 2.67 – 2.64 (m, 2H), 2.31 – 2.22 (m, 2H), 1.67 – 1.58 (m, 6H).
^{13}C {^{1}H} NMR (101 MHz, $CDCl_3$) δ 157.5, 149.2, 148.3, 136.0, 124.1, 122.1, 120.7, 38.2, 29.7, 28.7, 27.9, 26.7.

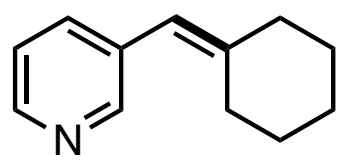

3-(cyclohexylidenemethyl)pyridine (P18) [37]
Prepared following general procedure A using 3-(nitromethyl)pyridine (**S18**, 0.45 mmol, 1.0 equiv.), nitrocyclohexane (**S20**, 2.8 equiv.), and Cs_2CO_3 (3.7 equiv.) in 1,2-DFB/*t*-BuOH (4:1, 31.25 mM) at 28 °C. The title compound was isolated via flash column chromatography (100:0:0 to 89:10:1 Hexanes:EtOAc:Triethylamine) as a colorless oil (53 mg, 68% yield).
TLC (SiO_2) R_f = 0.32 in 4:1 Hexanes:EtOAc.
^{1}H NMR (400 MHz, $CDCl_3$) δ 8.53 – 8.45 (m, 1H), 8.41 (dd, *J* = 4.9, 1.7 Hz, 1H), 7.50 – 7.40 (m, 1H), 7.24 – 7.21 (m, 1H), 6.16 (s, 1H), 2.35 – 2.30 (m, 2H), 2.29 – 2.24 (m, 2H), 1.66 – 1.56 (m, 6H).
^{13}C {^{1}H} NMR (101 MHz, $CDCl_3$) δ 150.1, 146.9, 146.4, 136.3, 134.2, 123.1, 118.4, 37.8, 29.6, 28.7, 28.0, 26.7.

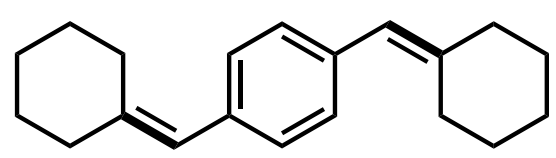

1,4-bis(cyclohexylidenemethyl)benzene (P19) [30]
Prepared following general procedure A using 1,4-bis(nitromethyl)benzene (**S19**, 0.45 mmol, 1.0 equiv.), nitrocyclohexane (**S20**, 5.6 equiv.), and Cs_2CO_3 (7.4 equiv.) in 1,2-DFB/*t*-BuOH (4:1, 31.25 mM) at 28 °C. The title compound was isolated via flash column chromatography (100% Hexanes) as a white solid (16 mg, 14% yield).
TLC (SiO_2) R_f = 0.51 in Hexanes.
^{1}H NMR (400 MHz, $CDCl_3$) δ 7.14 (s, 4H), 6.20 (s, 2H), 2.41 – 2.34 (m, 4H), 2.32 – 2.11 (m, 4H), 1.66 – 1.55 (m, 12H).
^{13}C {^{1}H} NMR (101 MHz, $CDCl_3$) δ 143.3, 136.1, 128.7, 122.0, 37.9, 29.7, 28.8, 28.0, 26.9.

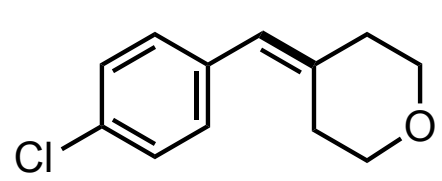

4-(4-chlorobenzylidene)tetrahydro-2*H*-pyran (P20)
Prepared following general procedure A using 1-chloro-4-(nitromethyl)benzene (**S2**, 0.45 mmol, 1.0 equiv.), 4-nitrotetrahydro-2*H*-pyran (**S23**, 2.8 equiv.), and Cs_2CO_3 (3.7 equiv.) in EtOAc/*t*-BuOH (4:1, 50.0 mM) at 28 °C. The title compound was isolated via flash column chromatography (0–10% EtOAc/Hexanes) as a colorless oil (57 mg, 61% yield).
TLC (SiO_2) R_f = 0.5 in 9:1 Hexanes:EtOAc.
^{1}H NMR (500 MHz, CD_2Cl_2) δ 7.29 (d, *J* = 8.5 Hz, 2H), 7.15 (d, *J* = 8.4 Hz, 2H), 6.28 (s, 1H), 3.73 (t, *J* = 5.5 Hz, 2H), 3.62 (t, *J* = 5.5 Hz, 2H), 2.48 – 2.46 (m, 2H), 2.38 – 2.36 (m, 2H).
^{13}C {^{1}H} NMR (126 MHz, CD_2Cl_2) δ 139.5, 136.6, 132.1, 130.7, 128.6, 122.8, 69.7, 68.8, 37.7, 31.1.
HRMS (ESI-TOF): *m/z* calculated for $C_{13}H_{14}ClO_3$ $[M+HCO_2]^-$ = 253.0637, found 253.0637.

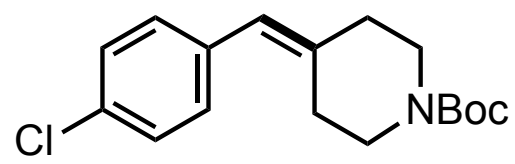

tert-butyl 4-(4-chlorobenzylidene)piperidine-1-carboxylate (P21) [38]

Prepared following general procedure A using 1-chloro-4-(nitromethyl)benzene (**S2**, 0.45 mmol, 1.0 equiv.), *tert*-butyl 4-nitropiperidine-1-carboxylate (**S24**, 2.8 equiv.), and Cs_2CO_3 (3.7 equiv.) in EtOAc/*t*-BuOH (4:1, 50.0 mM) at 28 °C. The title compound was isolated via flash column chromatography (0–10% EtOAc/Hexanes) as a white solid (115 mg, 83% yield).

TLC (SiO_2) R_f = 0.39 in 9:1 Hexanes:EtOAc.

^{1}H NMR (500 MHz, $CDCl_3$) δ 7.28 (d, *J* = 8.4 Hz, 2H), 7.11 (d, *J* = 8.4 Hz, 2H), 6.30 (s, 1H), 3.52 – 3.48 (m, 2H), 3.43 – 3.34 (m, 2H), 2.42 (t, *J* = 6.0 Hz, 2H), 2.32 (t, *J* = 6.5 Hz, 2H), 1.48 (s, 9H).

^{13}C {^{1}H} NMR (126 MHz, $CDCl_3$) δ 154.9, 139.4, 136.0, 132.2, 130.3, 128.5, 123.5, 79.8, 45.5, 44.6, 36.3, 29.3, 28.6.

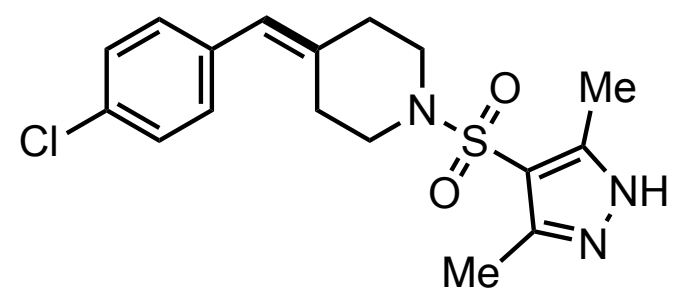

4-(4-chlorobenzylidene)-1-((3,5-dimethyl-1H-pyrazol-4-yl)sulfonyl)piperidine (P22) [39]

Prepared following general procedure A using 1-chloro-4-(nitromethyl)benzene (**S2**, 0.45 mmol, 1.0 equiv.), 1-((3,5-dimethyl-1*H*-pyrazol-4-yl)sulfonyl)-4-nitropiperidine (**S46**, 2.8 equiv.), and Cs_2CO_3 (3.7 equiv.) in 1,2-DFB/*t*-BuOH (4:1, 31.25 mM) at 28 °C. The title compound was isolated via flash column chromatography (0–50% EtOAc/Hexanes) as a white solid (51 mg, 31% yield).

TLC (SiO_2) R_f = 0.40 in 19:1 DCM:MeOH.

^{1}H NMR (600 MHz, DMSO) δ 13.02 (br s, 1H), 7.35 (d, *J* = 8.0 Hz, 2H), 7.20 (d, *J* = 8.1 Hz, 2H), 6.31 (s, 1H), 3.02 (t, *J* = 5.7 Hz, 2H), 2.93 (t, *J* = 5.8 Hz, 2H), 2.48 (t, *J* = 5.8 Hz, 2H), 2.39 (t, *J* = 5.8 Hz, 2H), 2.30 (s, 6H).

^{13}C {^{1}H} NMR (151 MHz, DMSO) δ 137.7, 135.5, 131.0, 130.5, 128.2, 123.3, 110.5, 46.9, 46.1, 34.9, 27.8.

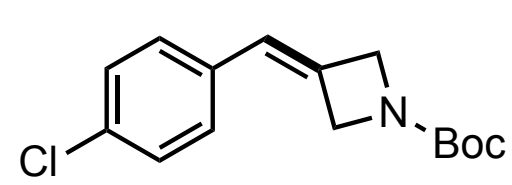

tert-butyl 3-(4-chlorobenzylidene)azetidine-1-carboxylate (P23) [40]

Prepared following general procedure A using 1-chloro-4-(nitromethyl)benzene (**S2**, 0.45 mmol, 1.0 equiv.), *tert*-butyl 3-nitroazetidine-1-carboxylate (**S25**, 2.8 equiv.), and Cs_2CO_3 (3.7 equiv.) in DCE/EtOH (4:1, 31.25 mM) at 28 °C. The title compound was isolated via flash column chromatography (0–10% EtOAc/Hexanes) as a white solid (43 mg, 34% yield).

TLC (SiO_2) R_f = 0.37 in 9:1 Hexanes:EtOAc.

^{1}H NMR (400 MHz, $CDCl_3$) δ 7.30 (d, *J* = 8.5 Hz, 2H), 7.02 (d, *J* = 8.5 Hz, 2H), 6.22 (s, 1H), 4.80 (q, *J* = 2.8 Hz, 2H), 4.63 (q, *J* = 2.7 Hz, 2H), 1.48 (s, 9H).
^{13}C {^{1}H} NMR (101 MHz, $CDCl_3$) δ 156.4, 134.7, 132.9, 131.7, 129.1, 128.4, 121.4, 80.1, 59.0 (br s), 28.6.

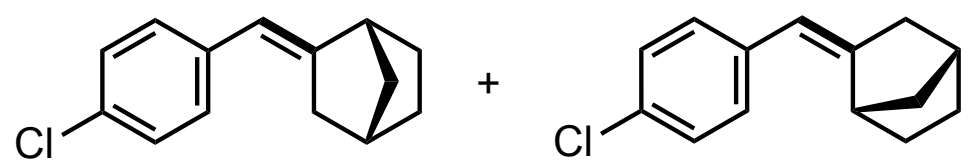

(1R,4S)-2-((*E*)-4-chlorobenzylidene)bicyclo[2.2.1]heptane (*E*-P24), and (1S,4R)-2-((*Z*)-4-chlorobenzylidene)bicyclo[2.2.1]heptane (*Z*-P24)
Prepared following general procedure A using 1-chloro-4-(nitromethyl)benzene (**S2**, 0.45 mmol, 1.0 equiv.), (1R,2R,4S)-2-nitrobicyclo[2.2.1]heptane (**S26**, 2.8 equiv.), and Cs_2CO_3 (3.7 equiv.) in EtOAc/*t*-BuOH (4:1, 50.0 mM) at 28 °C. The title compound was isolated via flash column chromatography (100 % Hexanes) as a white solid (60 mg, 61% yield).
TLC (SiO_2) R_f = 0.80 in Hexanes.
^{1}H NMR (500 MHz, CD_2Cl_2) δ 7.30 – 7.17 (m, 4H, *E*+*Z*), 6.25 (s, 0.82H, *E*), 6.06 (s, 0.18H, *Z*), 3.19 (d, *J* = 4.3 Hz, 0.18H, *Z*), 2.82 (d, *J* = 4.0 Hz, 0.82H, *E*), 2.50 (d, *J* = 4.4 Hz, 0.82H, *E*), 2.44 (dq, *J* = 16.2, 3.3 Hz, 0.82H, *E*), 2.40 – 2.31 (m, 0.54H, *Z*), 2.21 – 2.17 (m, 0.82H, *Z*), 2.04 (dt, *J* = 15.1, 2.2 Hz, 0.18H, *Z*), 1.87 – 1.57 (m, 2.18H, *E*+*Z*), 1.53 – 1.35 (m, 4H, *E*+*Z*).
^{13}C {^{1}H} NMR (126 MHz, CD_2Cl_2) δ 151.4, 150.7, 137.95, 137.92, 131.3, 131.2, 129.6, 129.3, 128.7, 128.6, 117.9, 117.0, 48.3, 41.9, 40.8, 40.1, 39.4, 39.1, 37.7, 36.3, 29.9, 29.1, 28.7, 28.6.
HRMS (ESI-TOF): *m/z* calculated for $C_{16}H_{13}ClF_3O$ $[M+CF_3CO_2-(H_2O)]^-$ = 313.0613, found 313.0617.

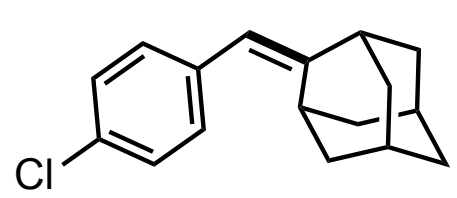

2-(4-chlorobenzylidene)adamantane (P25)
Prepared following general procedure A using 1-chloro-4-(nitromethyl)benzene (**S2**, 0.45 mmol, 1.0 equiv.), 2-nitroadamantane (**S22**, 2.8 equiv.), and Cs_2CO_3 (3.7 equiv.) in 1,2-DFB/*t*-BuOH (4:1, 31.25 mM) at 28 °C. The title compound was isolated via flash column chromatography (100 % Hexanes) as a white solid (50 mg, 43% yield).
TLC (SiO_2) R_f = 0.86 in Hexanes.
^{1}H NMR (400 MHz, CD_2Cl_2) δ 7.26 (d, *J* = 8.5 Hz, 2H), 7.13 (d, *J* = 8.6 Hz, 2H), 6.14 (s, 1H), 3.08 (s, 1H), 2.48 (s, 1H), 2.01 – 1.93 (m, 4H), 1.92 – 1.85 (m, 6H), 1.84 – 1.76 (m, 2H).
^{13}C {^{1}H} NMR (101 MHz, CD_2Cl_2) δ 153.0, 137.4, 131.5, 130.5, 128.5, 116.2, 41.5, 40.3, 39.4, 37.5, 32.8, 28.9.
HRMS (ESI-TOF): *m/z* calculated for $C_{17}H_{18}Cl$ $[M-H]^-$ = 257.1103, found 257.1103.

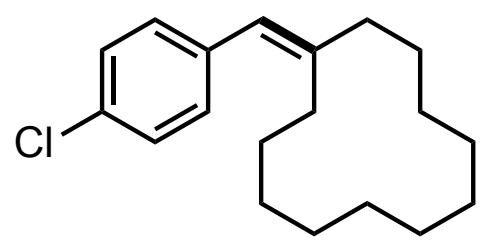

(4-chlorobenzylidene)cyclododecane (P26)

Prepared following general procedure A using 1-chloro-4-(nitromethyl)benzene (**S2**, 0.45 mmol, 1.0 equiv.), nitrocyclododecane (**S21**, 2.8 equiv.), and Cs_2CO_3 (3.7 equiv.) in EtOAc/*t*-BuOH (4:1, 50.0 mM) at 28 °C. The title compound was isolated via flash column chromatography (100 % Hexanes) as a white solid (93 mg, 71% yield).

TLC (SiO_2) R_f = 0.88 in Hexanes.

^{1}H NMR (500 MHz, CD_2Cl_2) δ 7.27 (d, *J* = 8.5 Hz, 2H), 7.17 (d, *J* = 8.5 Hz, 2H), 6.31 (s, 1H), 2.27 – 2.08 (m, 4H), 1.65 – 1.60 (m, 2H), 1.57 – 1.47 (m, 2H), 1.45 – 1.26 (m, 14H).

^{13}C {^{1}H} NMR (126 MHz, CD_2Cl_2) δ 144.2, 137.9, 131.7, 130.4, 128.5, 124.7, 33.0, 28.3, 25.6, 25.3, 24.8, 24.7, 24.6, 24.5, 24.3, 23.4, 22.9.

HRMS (ESI-TOF): *m/z* calculated for $C_{19}H_{27}ClBr$ $[M+Br]^-$ = 371.0969, found 371.0970.

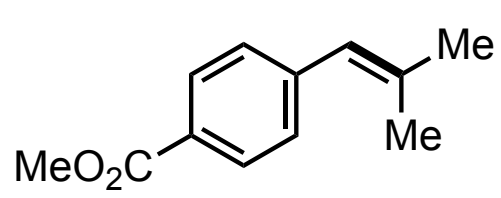

methyl 4-(2-methylprop-1-en-1-yl)benzoate (P27) [41]

Prepared following general procedure A using methyl 4-(nitromethyl)benzoate (**S4**, 0.45 mmol, 1.0 equiv.), 2-nitropropane (**S27**, 2.8 equiv.), and Cs_2CO_3 (3.7 equiv.) in EtOAc/*t*-BuOH (4:1, 50.0 mM) at 28 °C. The title compound was isolated via flash column chromatography (0–20% EtOAc/Hexanes) as a colorless oil (55 mg, 60% yield).

TLC (SiO_2) R_f = 0.57 in 9:1 Hexanes:EtOAc.

^{1}H NMR (500 MHz, CD_2Cl_2) δ 7.95 (d, *J* = 8.4 Hz, 2H), 7.30 (d, *J* = 8.2 Hz, 2H), 6.31 (s, 1H), 3.88 (s, 3H), 1.93 (s, 3H), 1.89 (s, 3H).

^{13}C {^{1}H} NMR (126 MHz, CD_2Cl_2) δ 167.2, 143.9, 138.7, 129.7, 129.0, 127.9, 124.7, 52.2, 27.2, 19.8.

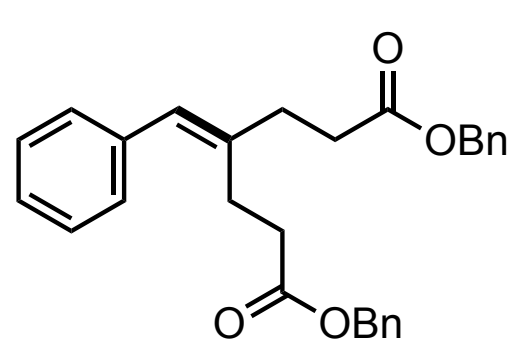

dibenzyl 4-benzylideneheptanedioate (P28)

Prepared following general procedure A using (nitromethyl)benzene (**S1**, 0.25 mmol, 1.0 equiv.), dibenzyl 4-nitroheptanedioate (**S28**, 2.8 equiv.), and Cs_2CO_3 (3.7 equiv.) in 1,2-DFB/*t*-BuOH (4:1, 31.25 mM) at 28 °C. The title compound was isolated via flash column chromatography (0–20% EtOAc/Hexanes) as a colorless oil (70 mg, 65% yield).

TLC (SiO_2) R_f = 0.26 in 9:1 Hexanes:EtOAc.

^{1}**H NMR** (500 MHz, $CDCl_3$) δ 7.37 – 7.27 (m, 12H), 7.20 (t, *J* = 7.4 Hz, 1H), 7.14 (d, *J* = 7.5 Hz, 2H), 6.33 (s, 1H), 5.14 (s, 2H), 5.08 (s, 2H), 2.62 – 2.57 (m, 4H), 2.54 – 2.47 (m, 4H).
^{13}**C {^{1}H} NMR** (126 MHz, $CDCl_3$) δ 172.9, 172.8, 139.1, 137.6, 136.1, 136.0, 128.72, 128.69, 128.67, 128.43, 128.41, 128.39, 128.36, 128.3, 127.4, 126.6, 66.50, 66.48, 33.2, 33.1, 31.9, 26.2.
HRMS (ESI-TOF): *m/z* calculated for $C_{28}H_{28}O_4$ [M] $^{\bullet-}$ = 428.1993, found 428.1996.

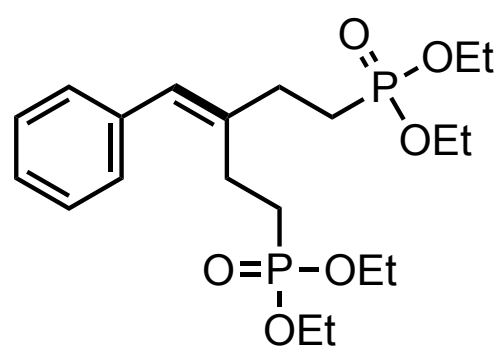

tetraethyl (3-benzylidenepentane-1,5-diyl)bis(phosphonate) (P29)
Prepared following general procedure A using (nitromethyl)benzene (**S1**, 0.18 mmol, 1.0 equiv.), tetraethyl (3-nitropentane-1,5-diyl)bis(phosphonate) (**S29**, 2.6 equiv.), and Cs_2CO_3 (3.4 equiv.) in EtOAc/*t*-BuOH (4:1, 50.0 mM) at 28 °C. The title compound was isolated via flash column chromatography (0–20% EtOAc/Hexanes) as a colorless oil (61 mg, 78% yield).
TLC (SiO_2) R_f = 0.77 in DCM.
^{1}**H NMR** (500 MHz, CD_2Cl_2) δ 7.33 (t, *J* = 7.5 Hz, 2H), 7.21 (d, *J* = 7.6 Hz, 3H), 4.13 – 4.05 (m, 4H), 4.03 – 3.96 (m, 4H), 2.55 – 2.38 (m, 4H), 1.96 – 1.88 (m, 2H), 1.88 – 1.79 (m, 2H), 1.32 (t, *J* = 7.1 Hz, 6H), 1.26 (t, *J* = 7.1 Hz, 6H).
^{13}**C {^{1}H} NMR** (126 MHz, CD_2Cl_2) δ 164.2, 161.3, 152.3, 152.0, 150.2, 150.1, 85.3 (t, *J* = 6.0 Hz), 53.0 (d, *J* = 4.1 Hz), 48.1 (d, *J* = 139.5 Hz), 47.2 (d, *J* = 4.5 Hz), 40.1 (d, *J* = 6.0 Hz), 40.0 (d, *J* = 6.0 Hz).
HRMS (ESI-TOF): *m/z* calculated for $C_{20}H_{34}O_6P_2$ [M] $^{\bullet+}$ = 432.1825, found 432.1792.

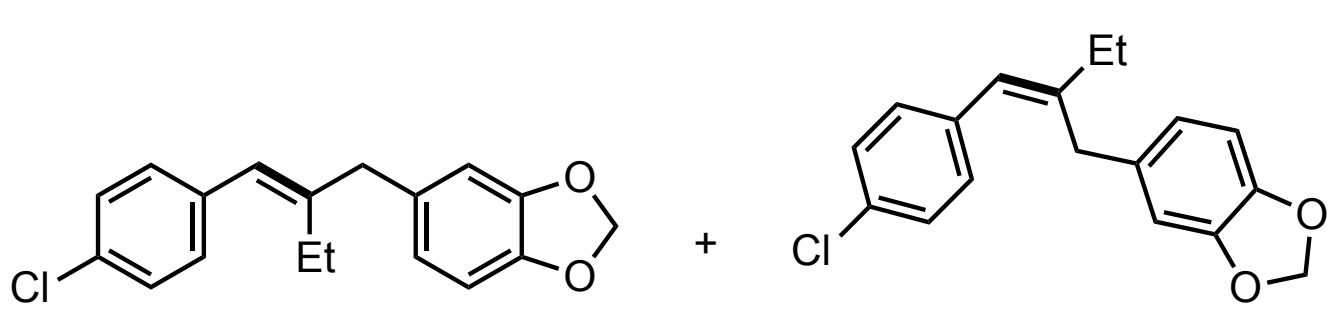

(*E/Z*)-5-(2-(4-chlorobenzylidene)butyl)benzo[d][1,3]dioxole (P30)
Prepared following general procedure A using 1-chloro-4-(nitromethyl)benzene (**S2**, 0.2 mmol, 1.0 equiv.), 5-(2-nitrobutyl)benzo[d][1,3]dioxole (**S30**, 2.8 equiv.), and Cs_2CO_3 (3.7 equiv.) in EtOAc/*t*-BuOH (4:1, 50 mM) at 28 °C. The title compound was isolated via flash column chromatography (0–30% EtOAc/Hexanes) as a colorless oil (41 mg, 68% yield).
TLC (SiO_2) R_f = 0.82 in 4:1 Hexanes:EtOAc.
^{1}**H NMR** (600 MHz, $CDCl_3$) δ 7.29 – 7.24 (m, 2H, *E*+*Z*), 7.17 (d, *J* = 8.4 Hz, 0.68H, *E*), 7.15 (d, *J* = 8.4 Hz, 1.32H, *Z*), 6.76 – 6.72 (m, 1.66H, *E*+*Z*), 6.69 (dd, *J* = 7.9, 1.7 Hz, 0.66H, *E*), 6.64 – 6.59 (m, 0.68H, *Z*), 6.43 (s, 0.34H, *Z*), 6.20 (s, 0.66H, *E*), 5.94 (s, 1.32H, *E*), 5.93 (s, 0.68H, *Z*), 3.50 (s, 0.68H, *Z*), 3.40 (s, 1.32H, *E*), 2.19 – 2.13 (m, 1.32H, *E*), 2.10 – 2.07 (m, 0.68H, *Z*), 1.08 (t, *J* = 7.5 Hz, 1.02H, *Z*), 1.05 (t, *J* = 7.5 Hz, 1.98H, *E*).

^{13}C {^{1}H} NMR (151 MHz, $CDCl_3$) δ 147.9, 147.8, 146.1, 146.0, 145.1, 143.6, 136.8, 133.6, 133.5, 132.1, 132.0, 130.0, 129.9, 128.5, 128.4, 125.5, 124.7, 122.1, 121.4, 109.5, 109.0, 108.4, 108.3, 101.0, 43.2, 36.5, 29.9, 29.8, 23.4, 14.3, 13.0, 12.7.
HRMS (ESI-TOF): *m/z* calculated for $C_{18}H_{14}ClO$ [M-H-(H_2O)]$^-$ = 281.0739, found 281.0741.

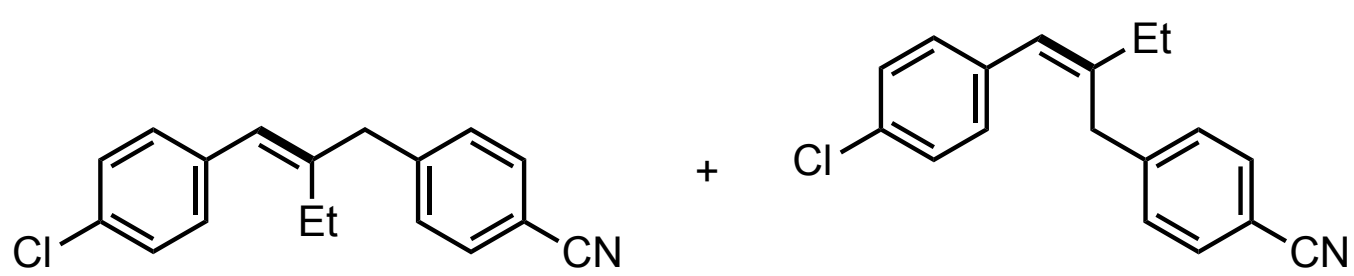

(*E/Z*)-4-(2-(4-chlorobenzylidene)butyl)benzonitrile (P31)
Prepared following general procedure A using 1-chloro-4-(nitromethyl)benzene (**S2**, 0.2 mmol, 1.0 equiv.), 4-(2-nitrobutyl)benzonitrile (**S31**, 2.8 equiv.), and Cs_2CO_3 (3.7 equiv.) in EtOAc/*t*-BuOH (4:1, 50.0 mM) at 28 °C. The title compound was isolated via flash column chromatography (0–30% EtOAc/Hexanes) as a colorless oil (30 mg, 54% yield).
TLC (SiO_2) R_f = 0.75 in 4:1 Hexanes:EtOAc.
^{1}H NMR (600 MHz, $CDCl_3$) δ 7.61 – 7.08 (m, 8H, *E*+*Z*), 6.49 (s, 0.3H, *Z*), 6.18 (s, 0.7H, *E*), 3.62 (s, 0.6H, *Z*), 3.52 (s, 1.4H, *E*), 2.17 – 2.09 (m, 1.4H, *E*), 2.08 – 2.00 (m, 0.6H, *Z*), 1.13 – 0.94 (m, 3H, *E*+*Z*).
^{13}C {^{1}H} NMR (151 MHz, $CDCl_3$) δ 145.6, 143.4, 141.8, 136.4, 136.2, 132.52, 132.46, 132.4, 130.0, 129.8, 129.4, 128.7, 128.5, 126.8, 125.9, 119.13, 119.10, 110.4, 110.3, 43.5, 37.1, 29.8, 23.7, 13.0, 12.6.
HRMS (ESI-TOF): *m/z* calculated for $C_{18}H_{15}ClN$ [M-H]$^-$ = 280.0899, found 280.0911.

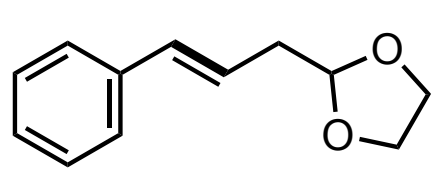

2-cinnamyl-1,3-dioxolane (P32) [42]
Prepared following general procedure A using (nitromethyl)benzene (**S1**, 0.25 mmol, 1.0 equiv.), 2-(2-nitroethyl)-1,3-dioxolane (**S32**, 2.6 equiv.), and Cs_2CO_3 (3.4 equiv.) in 1,2-DFB/*t*-BuOH (4:1, 31.25 mM) at 28 °C. The title compound was isolated via flash column chromatography (0–2% EtOAc/Hexanes) as a colorless oil (16 mg, 33% yield).
TLC (SiO_2) R_f = 0.15 in Hexanes.
^{1}H NMR (600 MHz, $CDCl_3$) δ 7.40 – 7.34 (m, 2H), 7.29 (t, *J* = 7.7 Hz, 2H), 7.22 – 7.18 (m, 1H), 6.51 (d, *J* = 16.0 Hz, 1H), 6.24 (dt, *J* = 15.9, 7.2 Hz, 1H), 4.99 (t, *J* = 4.7 Hz, 1H), 4.05 – 3.97 (m, 2H), 3.93 – 3.85 (m, 2H), 2.60 (ddd, *J* = 7.2, 4.7, 1.5 Hz, 2H).
^{13}C {^{1}H} NMR (151 MHz, $CDCl_3$) δ 137.5, 133.3, 128.6, 127.4, 126.3, 123.9, 104.0, 65.2, 38.1.

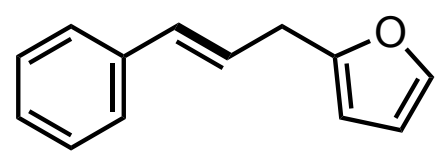

2-cinnamylfuran (P33) [43]

Prepared following general procedure A using (nitromethyl)benzene (**S1**, 0.45 mmol, 1.0 equiv.), 2-(2-nitroethyl)furan (**S34**, 2.6 equiv.), and Cs_2CO_3 (3.4 equiv.) in 1,2-DFB/*t*-BuOH (4:1, 31.25 mM) at 28 °C. Due to inseparability of the title compound by chromatography, ^{1}H NMR yield determined using CH_2Br_2 as an internal standard 34% NMR yield.

^{1}H NMR (500 MHz, $CDCl_3$) (crude) δ 6.50 (d, *J* = 15.8 Hz, 1H).

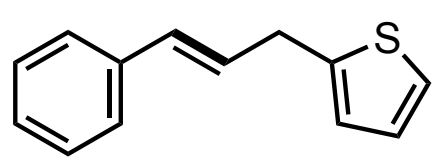

2-cinnamylthiophene (P34) [44]

Prepared following general procedure A using (nitromethyl)benzene (**S1**, 0.18 mmol, 1.0 equiv.), 2-(2-nitroethyl)thiophene (**S33**, 2.2 equiv.), and Cs_2CO_3 (3.0 equiv.) in 1,2-DFB/*t*-BuOH (4:1, 31.25 mM) at 28 °C. Due to inseparability of the title compound by chromatography, ^{1}H NMR yield determined using CH_2Br_2 as an internal standard 35% NMR yield.

^{1}H NMR (600 MHz, $CDCl_3$) (crude) δ 6.51 (dt, *J* = 15.7, 1.6 Hz, 1H), 6.37 (dt, *J* = 15.7, 6.9 Hz, 1H).

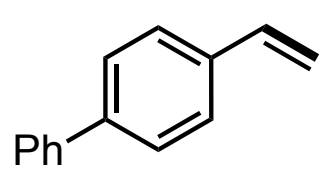

4-vinyl-1,1'-biphenyl (P35) [45]

Prepared following general procedure A using 4-(nitromethyl)-1,1'-biphenyl (**S5**, 0.353 mmol, 1.0 equiv.), nitromethane (**S35**, 2.8 equiv.), and Cs_2CO_3 (3.7 equiv.) in 1,2-DFB/*t*-BuOH (4:1, 31.25 mM) at 28 °C. Due to low yield, ^{1}H NMR yield determined using CH_2Br_2 as an internal standard as 4% NMR yield.

^{1}H NMR (500 MHz, $CDCl_3$) (crude) δ 6.76 (dd, *J* = 17.6, 10.9 Hz, 1H, olefin proton).

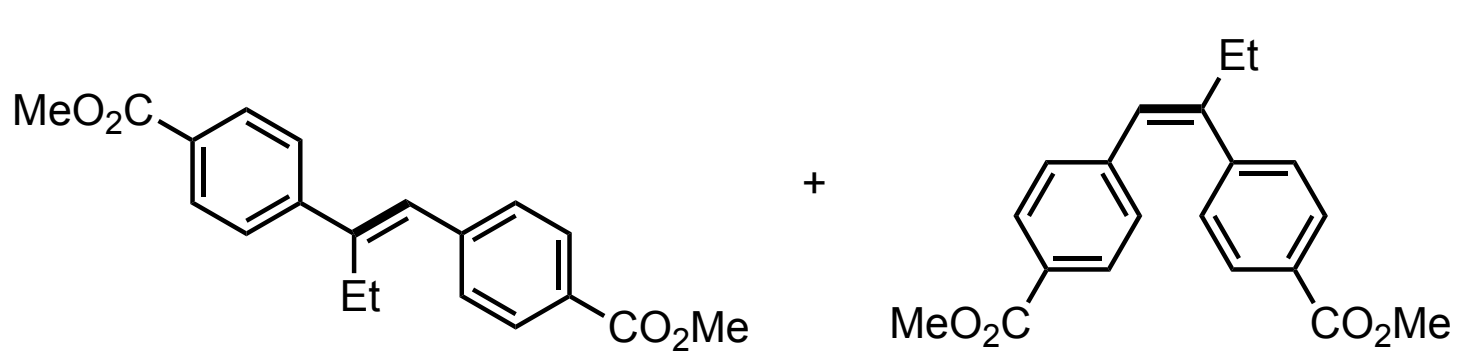

dimethyl 4,4'-(but-1-ene-1,2-diyl)(*E*)-dibenzoate and **dimethyl 4,4'-(but-1-ene-1,2-diyl)(*Z*)-dibenzoate (P36)**

Prepared following general procedure A using methyl 4-(nitromethyl)benzoate (**S4**, 0.10 mmol, 1.0 equiv.), methyl 4-(1-nitropropyl)benzoate (**S36**, 2.8 equiv.), and Cs_2CO_3 (3.7 equiv.) in EtOAc/*t*-BuOH (4:1, 50.0 mM) at 28 °C. The title compound was isolated via flash column chromatography (0–20% EtOAc/Hexanes) as a white solid (22 mg, 69% yield, 1:1 .5 *E*/*Z* mixture).

TLC (SiO_2) R_f = 0.5 in 10% EtOAc/Hexanes

^{1}H NMR (500 MHz, $CDCl_3$) δ 8.05 (d, *J* = 8.2 Hz, 4H, *E*), 7.96 (d, *J* = 8.0 Hz, 1.3 H, *Z*), 7.75 (d, *J* = 8.2 Hz, 1.3H, *Z*), 7.53 (d, *J* = 8.4 Hz, 2H, *E*), 7.40 (d, *J* = 8.1 Hz, 2H, *E*), 7.20 (d, *J* = 8.3 Hz, 1.3H, *Z*), 6.95 (d, *J* = 8.1 Hz, 1.3H, *Z*), 6.77 (s, 1H, *E*), 6.52 (s, 0.65H, *Z*), 3.93 (s, 6H, *E*), 3.92 (s, 2H, *Z*), 3.85 (s, 2H, *Z*), 2.76 (q, *J* = 7.5 Hz, 2H, *E*), 2.55 (q, *J* = 7.4 Hz, 1.3H, *Z*), 1.09 – 1.04 (m, 5H, *E*+*Z*).

^{13}C {^{1}H} NMR (101 MHz, $CDCl_3$) δ 167.1, 167.0, 147.1, 146.7, 146.2, 145.7, 142.6, 142.0, 130.1, 129.9, 129.8, 129.4, 129.1, 128.9, 128.7, 128.6, 128.4, 128.0, 126.8, 125.5, 52.3, 52.1, 33.3, 29.9, 23.5, 13.5, 12.9, 11.5.

HRMS (ESI-TOF): *m/z* calculated for $C_{20}H_{20}O_4Na$ [M+Na] $^+$ = 348.1288, found 348.1297.

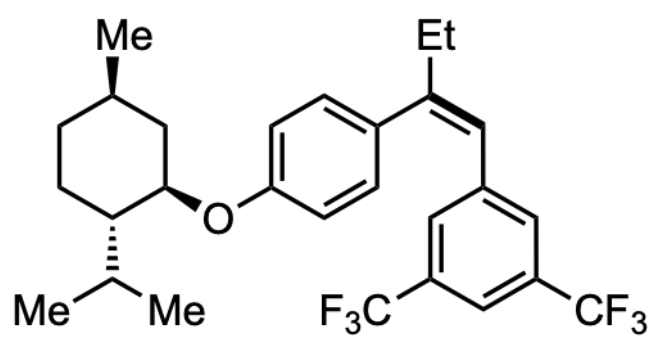

1-((*Z*)-2-(4-(((1*R*,2*S*,5*R*)-2-Isopropyl-5-methylcyclohexyl)oxy)phenyl)but-1-en-1-yl)-3,5-bis(trifluoromethyl)benzene (P37)

Prepared following general procedure A using 1-(nitromethyl)-3,5-bis(trifluoromethyl)benzene (**S11**, 0.25 mmol, 1.0 equiv.), 1-(((1S,2R,5S)-2-isopropyl-5-methylcyclohexyl)oxy)-4-(1-nitropropyl)benzene (**S38**, 2.8 equiv.), and Cs_2CO_3 (3.7 equiv.) in 1,2-DFB/*t*-BuOH (4:1, 31.25 mM) at 28 °C. The title compound was isolated via flash column chromatography (100% Hexanes) as a white solid (59 mg, 48% yield).

TLC (SiO_2) R_f = 0.50 in Hexanes

^{1}H NMR (500 MHz, $CDCl_3$) δ 7.51 (s, 1H), 7.32 (d, *J* = 1.7 Hz, 2H), 6.98 (d, *J* = 8.6 Hz, 2H), 6.85 (d, *J* = 8.6 Hz, 2H), 6.40 (s, 1H), 4.01 (td, *J* = 10.6, 4.1 Hz, 1H), 2.53 (qd, *J* = 7.4, 1.4 Hz, 2H), 2.20 (td, *J* = 7.0, 2.8 Hz, 1H), 2.16 – 2.08 (m, 1H), 1.76 – 1.66 (m, 2H), 1.29 – 1.21 (m, 5H), 1.10 (t, *J* = 7.4 Hz, 3H), 0.93 (t, *J* = 6.4 Hz, 6H), 0.78 (d, *J* = 6.9 Hz, 3H).

^{13}C {^{1}H} NMR (101 MHz, $CDCl_3$) δ 158.2, 149.6, 140.1, 131.9, 131.1, 130.9, 129.4, 127.9, 124.6, 122.2, 116.5, 48.2, 40.1, 34.7, 33.4, 31.6, 29.9, 26.3, 24.0, 22.2, 20.8, 16.6, 12.8.

^{19}F {^{1}H} NMR (471 MHz, $CDCl_3$) δ -62.9.

HRMS (ESI-TOF): *m/z* calculated for $C_{28}H_{30}F_6O$ [M-2H] $^{2-}$ = 248.1106, found 248.1117.

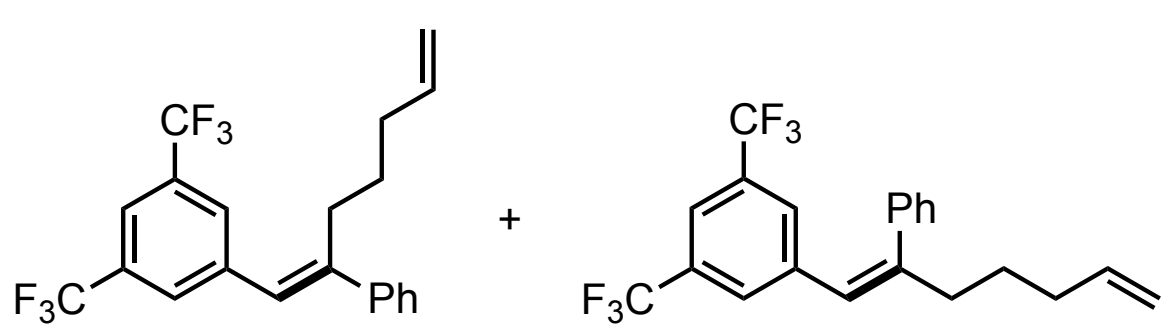

(*E*/*Z*)-1-(2-Phenylhepta-1,6-dien-1-yl)-3,5-bis(trifluoromethyl)benzene (P38)

Prepared following general procedure A using 1-(nitromethyl)-3,5-bis(trifluoromethyl)benzene (**S11**, 0.25 mmol, 1.0 equiv.), (1-nitrohex-5-en-1-yl)benzene (**S37**, 2.8 equiv.), and Cs_2CO_3 (3.7 equiv.) in 1,2-DFB/*t*-BuOH (4:1, 31.25 mM) at 28 °C. The

title compound was isolated via flash column chromatography (100% Hexanes) as a colorless oil (64 mg, 73% yield, 2:1 *E*/*Z* mixture).

TLC (SiO_2) R_f = 0.5 in Hexanes

^{1}H NMR (500 MHz, $CDCl_3$) δ 7.76 (s, 0.67H, *Z*), 7.74 (s, 1.33H, *E*), 7.52 (s, 0.33H, *Z*), 7.46 – 7.43 (m, 1.33H, *E*), 7.41 – 7.37 (m, 1.33H, *E*), 7.36 – 7.30 (m, 1.67H, *Z*+*E*), 7.28 (s, 0.67H, *E*), 7.10 (dd, *J* = 7.6, 1.9 Hz, 0.67H, *Z*), 6.69 (s, 0.67H, *E*), 6.47 (s, 0.33H, *Z*), 5.85 – 5.75 (m, 0.33H, *Z*), 5.76 – 5.66 (m, 0.67H, *E*), 5.04 – 4.91 (m, 2H, *Z*+*E*), 2.69 – 2.64 (m, 1.33H, *E*), 2.58 – 2.53 (m, 0.67H, *Z*), 2.12 (q, *J* = 7.1 Hz, 0.67H, *Z*), 2.05 (q, *J* = 7.1 Hz, 1.33H, *E*), 1.58 – 1.51 (m, 2H, *Z*+*E*).

^{13}C {^{1}H} NMR (126 MHz, $CDCl_3$) δ 148.1, 147.0, 142.0, 140.3, 139.9, 139.6, 138.4, 137.9, 132.1, 131.9, 131.6, 131.4, 131.2, 130.97, 129.1, 128.7, 128.2, 128.1, 127.9, 126.8, 125.4, 124.6, 123.8, 122.5, 115.5, 115.1, 40.0, 33.7, 33.3, 29.9, 28.0, 27.0 (includes splitting by CF3).

^{19}F {^{1}H} NMR (471 MHz, $CDCl_3$) δ -63.55, -63.19.

HRMS (ESI-TOF): *m/z* calculated for $C_{23}H_{19}F_6O$ [M+CH_3CO_2-(H_2O)] $^-$ = 425.1346, found 425.1337.

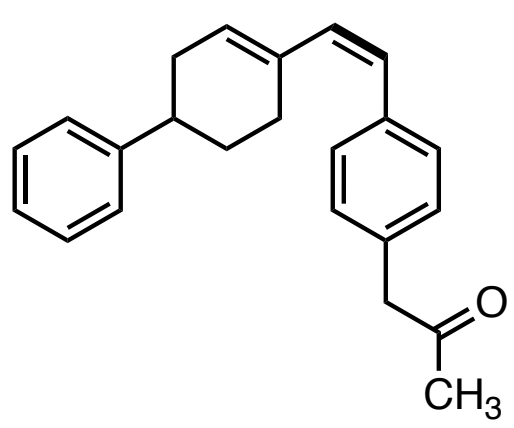

(*Z*)-1-(4-(2-(1,2,3,6-tetrahydro-[1,1'-biphenyl]-4-yl)vinyl)phenyl)propan-2-one (*Z*-P39)

Prepared following general procedure A using 4-(nitromethyl)-1,2,3,6-tetrahydro-1,1'-biphenyl (**S39**, 0.1 mmol, 1.0 equiv.), 1-(4-(nitromethyl)phenyl)propan-2-one (**S13**, 2.8 equiv.), and Cs_2CO_3 (3.7 equiv.) in 1,2-DCE/EtOH (4:1, 31.25 mM) at 28 °C. The title compound was isolated via column chromatography (95:4:1 Hexanes:ethyl acetate:dichloromethane) as a colorless oil (9.6 mg, 30% yield).

TLC (SiO_2) Rf = 0.49 in 4:1 Hexanes:EtOAc.

^{1}H NMR (400 MHz, $CDCl_3$) δ 7.34 – 7.28 (m, 4H), 7.24 – 7.18 (m, 3H), 7.13 (d, J = 8.0 Hz, 2H), 6.34 (d, *J* = 12.2 Hz, 1H), 6.14 (d, *J* = 12.2 Hz, 1H), 5.86 (s, 1H), 3.68 (s, 2H), 2.84 – 2.72 (m, 1H), 2.46 – 2.34 (m, 1H), 2.30 – 2.17 (m, 1H), 2.15 (s, 3H), 1.99 (dt, *J* = 17.1, 3.7 Hz, 1H), 1.93 – 1.85 (m, 1H), 1.69 (ddd, *J* = 24.4, 11.5, 5.1 Hz, 1H).

^{13}C {1H} NMR (101 MHz, $CDCl_3$) δ 206.6, 147.0, 137.4, 135.5, 133.4, 132.7, 129.4, 129.0, 128.7, 128.5, 127.4, 127.0, 126.2, 51.0, 39.8, 34.0, 30.1, 29.4, 28.7.

HRMS (ESI-TOF): *m/z* calculated for $C_{23}H_{24}ONa_2$ [M+2Na] $^{2+}$ = 181.0806 found 181.0789.

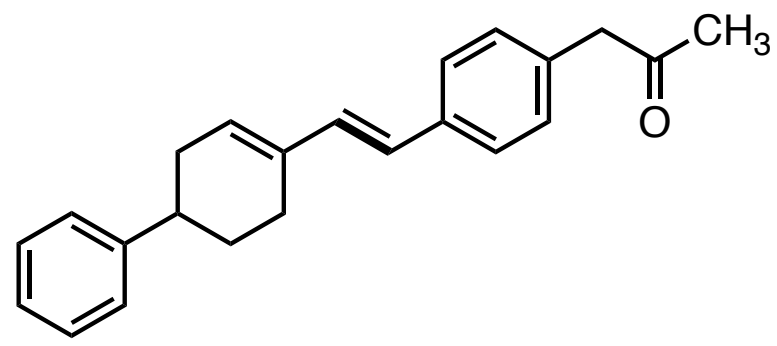

(*E*)-1-(4-(2-(1,2,3,6-tetrahydro-[1,1'-biphenyl]-4-yl)vinyl)phenyl)propan-2-one (*E*-P39)

The title compound was isolated via flash column chromatography (95:4:1 Hexanes:ethyl acetate:dichloromethane) as a white solid (4.9 mg, 15% yield).

TLC (SiO_2) Rf = 0.46 in 4:1 Hexanes:EtOAc.

^{1}H NMR (400 MHz, $CDCl_3$) δ 7.39 (d, *J* = 7.9 Hz, 2H), 7.32 (t, *J* = 7.5 Hz, 3H), 7.25 – 7.21 (m, 2H), 7.16 (d, *J* = 7.9 Hz, 2H), 6.82 (d, *J* = 16.1 Hz, 1H), 6.46 (d, *J* = 16.2 Hz, 1H), 5.99 (s, 1H), 3.68 (s, 2H), 2.86 (s, 1H), 2.62 – 2.45 (m, 2H), 2.37 (d, *J* = 10.6 Hz, 2H), 2.16 (s, 3H), 2.14 – 2.04 (m, 1H), 1.85 (qd, *J* = 11.7, 5.5 Hz, 1H).

^{13}C {1H} NMR (101 MHz, $CDCl_3$) δ 206.6, 146.9, 137.0, 135.8, 133.0, 132.2, 130.3, 129.8, 128.6, 127.0, 126.7, 126.3, 125.0, 51.0, 40.3, 34.4, 29.8, 29.4, 25.3.

HRMS (ESI-TOF): *m/z* calculated for $C_{23}H_{24}ONa$ $[M+Na]^+$ = 340.1753, found 340.1768.

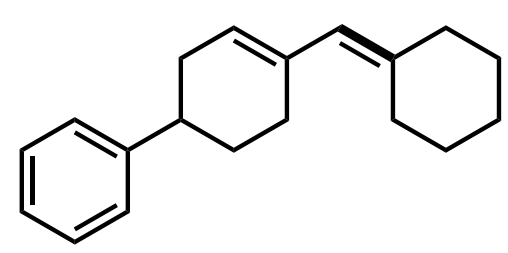

4-(cyclohexylidenemethyl)-1,2,3,6-tetrahydro-1,1'-biphenyl (P40)

Prepared following general procedure A using 4-(nitromethyl)-1,2,3,6-tetrahydro-1,1'-biphenyl (**S39**, 0.25 mmol, 1.0 equiv.), nitrocyclohexane (**S20**, 2.8 equiv.), and Cs_2CO_3 (3.7 equiv.) in 1,2-DCE/EtOH (4:1, 31.25 mM) at 28 °C. Due to inseparability of the title compound by chromatography, ^{1}H NMR yield determined using CH_2Br_2 as an internal standard 41% NMR yield.

TLC (SiO_2) Rf = 0.52 in Hexanes

^{1}H NMR (500 MHz, $CDCl_3$) (crude) δ 5.58 – 5.55 (m, 2H).

HRMS (ESI-TOF): *m/z* calculated for $C_{21}H_{24}F_3O_2$ $[M+CF_3CO_2]^-$ = 365.1734, found 365.1733.

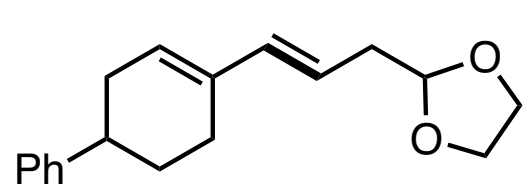

(*E*)-2-(3-(1,2,3,6-tetrahydro-[1,1'-biphenyl]-4-yl)allyl)-1,3-dioxolane (P41)

Prepared following general procedure A using 4-(nitromethyl)-1,2,3,6-tetrahydro-1,1'-biphenyl (**S39**, 0.25 mmol, 1.0 equiv.), 2-(2-nitroethyl)-1,3-dioxolane (**S32**, 2.8 equiv.), and Cs_2CO_3 (3.7 equiv.) in 1,2-DCE/EtOH (4:1, 31.25 mM) at 28 °C. The title compound was isolated via flash column chromatography (0–5% EtOAc/Hexanes) as a light pink oil (20 mg, 31% yield).

TLC (SiO_2) Rf = 0.33 in 9:1 Hexanes:EtOAc.

^{1}H NMR (400 MHz, $CDCl_3$) δ 7.36 – 7.27 (m, 2H), 7.25 – 7.15 (m, 3H), 6.21 (d, *J* = 15.7 Hz, 1H), 5.77 (d, *J* = 5.2 Hz, 1H), 5.58 (dt, *J* = 15.1, 7.2 Hz, 1H), 4.91 (t, *J* = 4.8 Hz, 1H), 4.09 – 3.94 (m, 2H), 3.92 – 3.84 (m, 2H), 2.79 (tdd, *J* = 12.4, 5.2, 2.7 Hz, 1H), 2.65 – 2.43 (m, 2H), 2.46 – 2.32 (m, 2H), 2.32 – 2.20 (m, 2H), 2.03 (ddd, *J* = 10.2, 5.2, 2.5 Hz, 1H), 1.89 – 1.70 (m, 1H).
^{13}C {^{1}H} NMR (101 MHz, $CDCl_3$) δ 147.0, 136.4, 135.4, 128.5, 127.9, 127.0, 126.2, 120.2, 104.2, 65.2, 40.4, 38.0, 34.1, 29.8, 25.3.
HRMS (ESI-TOF): *m/z* calculated for $C_{18}H_{20}O$ [M-(H_2O)] $^{•+}$ = 252.1509, found 252.1513

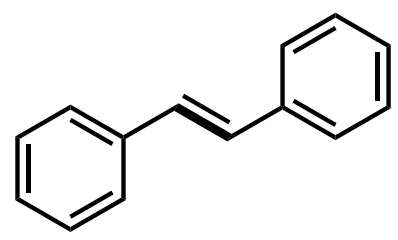

(*E*)-1,2-diphenylethene (P42) [46]
Prepared following general procedure A using (nitromethyl)benzene (**S1**, 0.45 mmol, 1.0 equiv.), and Cs_2CO_3 (1.0 equiv.) in 1,2-DFB/*t*-BuOH (4:1, 31.25 mM) at 28 °C for 1 h. The title compound was isolated via flash column chromatography (100% Hexanes) as a white solid (34.6 mg, 85% yield).
TLC (SiO_2) R_f = 0.84 in 1:1 Hexanes:DCM.
^{1}H NMR (400 MHz, $CDCl_3$) δ 7.56 – 7.48 (m, 4H), 7.41 – 7.33 (m, 4H), 7.28 – 7.24 (m, 2H), 7.12 (s, 2H).
^{13}C {^{1}H} NMR (101 MHz, $CDCl_3$) δ 137.5, 128.84, 128.83, 127.8, 126.7.

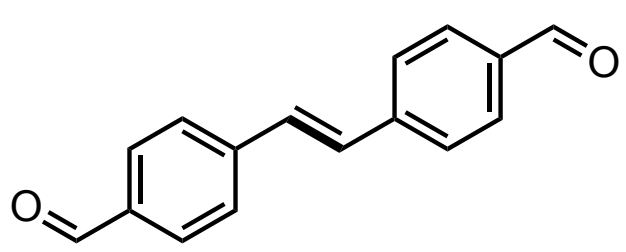

(*E*)-4,4'-(ethene-1,2-diyl)dibenzaldehyde (P43) [47]
Prepared following general procedure A using 4-(nitromethyl)benzaldehyde (**S14**, 0.45 mmol, 1.0 equiv.) and Cs_2CO_3 (1.0 equiv.) in 1,2-DFB/*t*-BuOH (4:1, 31.25 mM) at 28 °C for 1 h. The title compound was isolated via flash column chromatography (0–10% EtOAc/Hexanes) as an off-white solid (45 mg, 85% yield).
TLC (SiO_2) R_f = 0.14 in 1:1 Hexanes:DCM.
^{1}H NMR (500 MHz, $CDCl_3$) δ 10.02 (s, 2H), 7.91 (d, *J* = 8.3 Hz, 4H), 7.70 (d, *J* = 8.3 Hz, 4H), 7.30 (s, 2H).
^{13}C {^{1}H} NMR (126 MHz, $CDCl_3$) δ 191.7, 142.6, 136.1, 130.9, 130.4, 127.5.

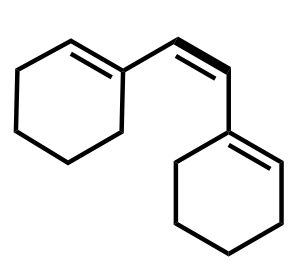

(*E*/*Z*)-1,2-di(cyclohex-1-en-1-yl)ethene (P44) [48]
Prepared following general procedure A using 1-(nitromethyl)cyclohex-1-ene (**S40**, 0.45 mmol, 1.0 equiv.), 4-(dichloromethyl)benzonitrile) (0.1 equiv.), and Cs_2CO_3 (1.0 equiv.) in

1,2-DFB/*t*-BuOH (4:1, 31.25 mM) at 28 °C. The title compound was isolated by flash column chromatography (100% Hexanes) as a colorless oil (12 mg, 14% yield).
TLC (SiO_2) R_f = 0.70 in Hexanes
^{1}H NMR (400 MHz, $CDCl_3$) δ 6.13 (s, 0.34H), 5.73 (d, *J* = 4.2 Hz, 0.34H), 5.71 (s, 1.66H), 5.64 – 5.58 (m, 1.66H), 2.20 – 2.12 (m, 1.36H), 2.08 (tdd, *J* = 9.8, 6.5, 3.1 Hz, 6.64H), 1.68 (tdd, *J* = 7.7, 4.9, 2.3 Hz, 1.36H), 1.64 – 1.54 (m, 6.64H).
^{13}C {^{1}H} NMR (101 MHz, $CDCl_3$) δ 136.5 (*Z*), 136.1 (*E*), 130.9 (*Z*), 128.9 (*E*), 128.8 (*E*), 127.8 (*Z*), 28.6 (*Z*), 26.2 (*E*), 25.8 (*Z*), 24.7 (*E*), 23.1 (*E*), 22.8 (*Z*), 22.7(*E*), 22.4 (*Z*).

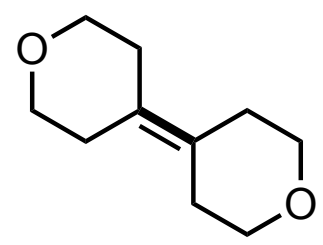

2,2',3,3',5,5',6,6'-octahydro-4,4'-bipyranylidene (P45)
Prepared following general procedure A using 4-nitrotetrahydro-2*H*-pyran (**S23**, 0.45 mmol, 1.0 equiv.) and Cs_2CO_3 (0.975 equiv.) in 1,2-DFB/*t*-BuOH (4:1, 31.25 mM) at 28 °C. The title compound was isolated by flash column chromatography (100% Hexanes) as an off-white solid (55 mg, 71% yield).
TLC (SiO_2) R_f = 0.07 in DCM.
^{1}H NMR (400 MHz, $CDCl_3$) δ 3.66 (dd, *J* = 6.0, 4.8 Hz, 8H), 2.31 (t, *J* = 5.4 Hz, 8H).
^{13}C {^{1}H} NMR (101 MHz, $CDCl_3$) δ 126.2, 69.2, 30.5.
HRMS (ESI-TOF): *m/z* calculated for $C_{10}H_{16}O_2Na$ $[M+Na]^+$ = 191.1043, found 191.1048.

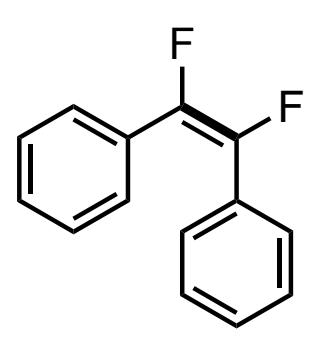

(*Z*)-1,2-difluoro-1,2-diphenylethene (P46) [49]
Prepared following general procedure A using 1-fluoro-1-phenylnitromethane (**S41**, 0.45 mmol, 1.0 equiv.) and Cs_2CO_3 (1.0 equiv.) in 1,2-DFB/*t*-BuOH (4:1, 31.25 mM) at 28 °C for 1 h. The title compound was isolated via flash column chromatography (100% Hexanes) as a colorless oil (45 mg, 93% yield).
TLC (SiO_2) R_f = 0.84 in 1:1 Hexanes:DCM.
^{1}H NMR (500 MHz, $CDCl_3$) δ 7.77 (d, *J* = 7.9 Hz, 4H), 7.46 (t, *J* = 7.7 Hz, 4H), 7.38 (td, *J* = 7.1, 1.3 Hz, 2H).
^{13}C {^{1}H} NMR (126 MHz, $CDCl_3$) δ 150.54 – 146.45 (m), 130.47 (t, *J* = 9.1 Hz), 126.01 (t, *J* = 8.6 Hz).
^{19}F {^{1}H} NMR (471 MHz, $CDCl_3$) δ -128.24

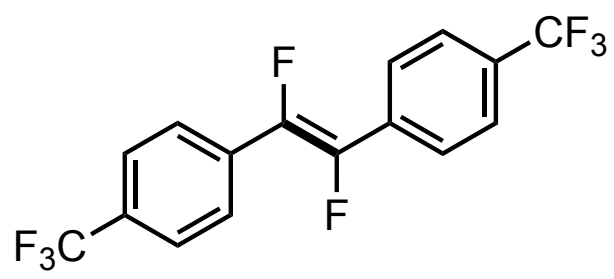

(*E*)-1,2-difluoro-1,2-bis(4-(trifluoromethyl)phenyl)ethene (P47) [50]

Prepared following general procedure A using 1-(fluoro(nitro)methyl)-4-(trifluoromethyl)benzene (**S42**, 0.45 mmol, 1.0 equiv.) and Cs_2CO_3 (1.0 equiv.) in 1,2-DFB/*t*-BuOH (4:1, 31.25 mM) at 28 °C for 1 h. The title compound was isolated via flash column chromatography (100% Hexanes) as a colorless oil (45 mg, 76% yield).

TLC (SiO_2) R_f = 0.86 in 1:1 Hexanes:DCM.

^{1}H NMR (400 MHz, $CDCl_3$) δ 7.90 (d, *J* = 8.3 Hz, 4H), 7.73 (d, *J* = 8.4 Hz, 4H).

^{13}C {^{1}H} NMR (101 MHz, $CDCl_3$) δ 150.9 – 146.2 (m), 133.2 (t, *J* = 9.1 Hz), 131.3 (q, *J* = 32.7 Hz), 126.4 (t, *J* = 9.1 Hz), 125.7 (q, *J* = 3.8 Hz), 123.9 (q, *J* = 272.2 Hz).

^{19}F {^{1}H} NMR (377 MHz, $CDCl_3$) δ -62.94, -149.99.

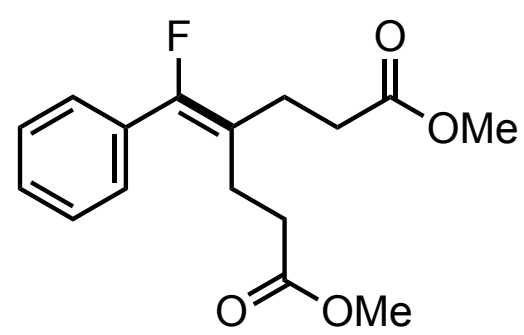

dimethyl 4-(fluoro(phenyl)methylene)heptanedioate (P48)

Prepared following general procedure A using (fluoro(nitro)methyl)benzene (**S41**, 0.2 mmol, 1.0 equiv.), dimethyl 4-nitroheptanedioate (**S50**, 2.8 equiv.), and Cs_2CO_3 (3.7 equiv.) in 1,2-DFB/*t*-BuOH (4:1, 31.25 mM) at 28 °C. The title compound was isolated via flash column chromatography (0–20% EtOAc/Hexanes) as a colorless oil (19 mg, 32% yield).

TLC (SiO_2) R_f = 0.36 in DCM.

^{1}H NMR (600 MHz, CD_2Cl_2) δ 7.43 – 7.35 (m, 5H), 3.68 (s, 3H), 3.60 (s, 3H), 2.57 – 2.50 (m, 4H), 2.45 – 2.40 (m, 4H).

^{13}C {^{1}H} NMR (151 MHz, CD_2Cl_2) δ 173.6, 173.2, 155.2 (d, *J* = 243.3 Hz), 132.9, 132.7, 129.44, 129.43, 128.78, 128.76, 128.7, 116.6, 116.4, 51.9, 33.0, 32.97, 32.91, 32.90, 25.8, 25.7, 23.6, 23.5.

^{19}F {^{1}H} NMR (471 MHz, CD_2Cl_2) δ -99.99.

HRMS (ESI-TOF): *m/z* calculated for $C_{16}H_{19}FO_4Na$ $[M+Na]^+$ = 317.1160, found 317.1163.

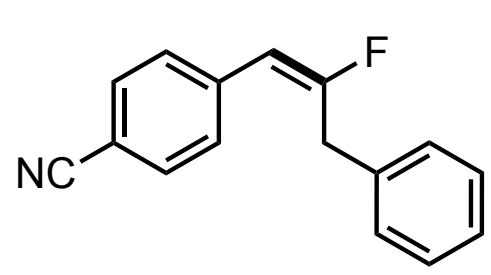

(*E*)-4-(2-fluoro-3-phenylprop-1-en-1-yl)benzonitrile (P49)

Prepared following general procedure A using 4-(nitromethyl)benzonitrile (**S49**, 0.45 mmol, 1.0 equiv.), (2-fluoro-2-nitroethyl)benzene (**S43**, 2.8 equiv.), and Cs_2CO_3 (3.7 equiv.) in 1,2-

DFB/*t*-BuOH (4:1, 31.25 mM) at 28 °C. The title compound was isolated via flash column chromatography (0–4% EtOAc/Hexanes) as a yellow oil (11 mg, 10% yield).
TLC (SiO_2) R_f = 0.82 in DCM.
^{1}H NMR 500 MHz, CD_2Cl_2) δ 7.65 – 7.48 (m, 4H), 7.40 – 7.26 (m, 5H), 5.60 (d, *J* = 37.9 Hz, 1H), 3.69 (d, *J* = 17.6 Hz, 2H).
^{13}C {^{1}H} NMR (151 MHz, CD_2Cl_2) δ 162.8 (d, *J* = 272.3 Hz), 138.6 (d, *J* = 2.8 Hz), 136.0, 132.6, 129.4, 129.2, 129.1, 127.6, 119.3, 110.6, 106.6 (d, *J* = 7.7 Hz), 39.95 (d, *J* = 27.1 Hz).
^{19}F {^{1}H} NMR (471 MHz, CD_2Cl_2) δ -95.54.
HRMS (ESI-TOF): *m/z* calculated for $C_{16}H_{13}FN$ [M+H] $^+$ = 238.1027, found 238.1043.

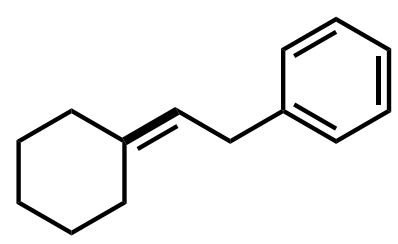

(2-Cyclohexylideneethyl)benzene (P50)
Prepared following general procedure A using nitrocyclohexane (**S20**, 0.45 mmol, 1.0 equiv.), (2-nitroethyl)benzene (**S45**, 2.8 equiv.), and Cs_2CO_3 (3.7 equiv.) in 1,2-DFB/*t*-BuOH (4:1, 31.25 mM) at 28 °C. The title compound was isolated via flash column chromatography (100% Hexanes) as a yellow oil (54 mg, 79% yield).
TLC (SiO_2) R_f = 0.70 in Hexanes
^{1}H NMR (500 MHz, $CDCl_3$) δ 7.30 – 7.17 (m, 5H), 5.28 (t, *J* = 7.3 Hz, 1H), 3.37 (d, *J* = 7.5 Hz, 2H), 2.27 – 2.12 (m, 5H), 1.57 – 1.55 (m, 5H).
^{13}C {^{1}H} NMR (101 MHz, $CDCl_3$) δ 142.1, 140.8, 128.50, 128.48, 125.8, 119.9, 37.4, 33.6, 28.9, 28.8, 28.0, 27.1.
HRMS (ESI-TOF): *m/z* calculated for $C_{14}H_{17}$ [M-H] $^-$ = 185.1330, found 185.1332.

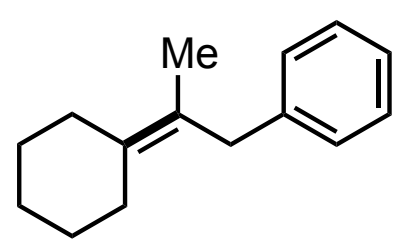

(2-Cyclohexylidenepropyl)benzene (P51)
Prepared following general procedure A using (2-nitropropyl)benzene (**S44**, 0.45 mmol, 1.0 equiv.), nitrocyclohexane (**S20**, 2.8 equiv.), and Cs_2CO_3 (3.7 equiv.) in 1,2-DFB/*t*-BuOH (4:1, 31.25 mM) at 28 °C. The title compound was isolated via flash column chromatography (100% Hexanes) as a yellow oil (50 mg, 67% yield).
TLC (SiO_2) R_f = 0.70 in Hexanes
^{1}H NMR (500 MHz, $CDCl_3$) δ 7.29 – 7.17 (m, 5H), 3.42 (s, 2H), 2.31 (t, *J* = 5.7 Hz, 2H), 2.24 (t, *J* = 5.7 Hz, 2H), 1.62 (s, 3H), 1.60 – 1.53 (m, 6H).
^{13}C {^{1}H} NMR (101 MHz, $CDCl_3$) δ 141.4, 134.6, 128.6, 128.3, 125.8, 123.2, 39.7, 28.6, 28.2, 27.1, 18.0.
HRMS (ESI-TOF): *m/z* calculated for $C_{17}H_{18}OF_3$ [M+CF_3CO_2-(H_2O)] $^-$ = 295.1315, found 295.1313.

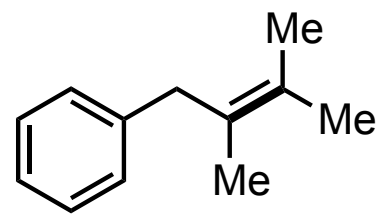

(2,3-Dimethylbut-2-en-1-yl)benzene (P52) [51]

Prepared following general procedure A using (2-nitropropyl)benzene (**S44**, 0.25 mmol, 1.0 equiv.), 2-nitropropane (**S27**, 2.8 equiv.), and Cs_2CO_3 (3.7 equiv.) in 1,2-DFB/*t*-BuOH (4:1, 31.25 mM) at 28 °C. The title compound was isolated via flash column chromatography (100% Hexanes) as a colorless oil (28 mg, 58% yield).

TLC (SiO_2) R_f = 0.60 in Hexanes

^{1}H NMR (500 MHz, $CDCl_3$) δ 7.31 – 7.24 (m, 2H), 7.21 – 7.13 (m, 3H), 3.41 (s, 2H), 1.80 (s, 3H), 1.74 (s, 3H), 1.60 (s, 3H).

^{13}C {^{1}H} NMR (101 MHz, $CDCl_3$) δ 141.2, 128.6, 128.4, 126.5, 125.9, 125.8, 40.3, 20.82, 20.77, 18.5.

HRMS (ESI-TOF): *m/z* calculated for $C_{12}H_{16}$ [M] $^{\bullet-}$ = 160.1257, found 160.1250.

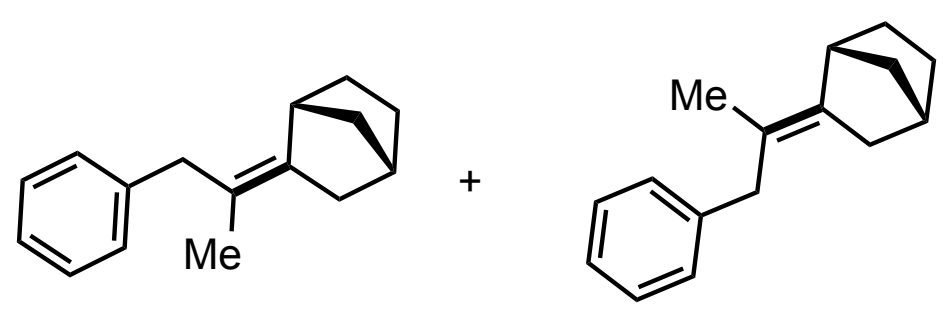

(1R,4S,Z)-2-(1-phenylpropan-2-ylidene)bicyclo[2.2.1]heptane (*Z*-P53) and **(1R,4S,E)-2-(1-phenylpropan-2-ylidene)bicyclo[2.2.1]heptane (*E*-P53)**

Prepared following general procedure A using (2-nitropropyl)benzene (**S44**, 0.3 mmol, 1.0 equiv.), (1R,2R,4S)-2-nitrobicyclo[2.2.1]heptane (**S26**, 2.8 equiv.), and Cs_2CO_3 (3.7 equiv.) in 1,2-DFB/*t*-BuOH (4:1, 31.25 mM) at 28 °C. The title compound was isolated via flash column chromatography (100% Hexanes) as a yellow oil (50 mg, 73% yield, 1:1 E/Z mixture).

TLC (SiO_2) R_f = 0.60 in Hexanes

^{1}H NMR (500 MHz, $CDCl_3$) (as an E/Z mixture) δ 7.20 – 7.03 (m, 5H), 3.31 (s, 1H), 3.16 (s, 1H), 2.97 (d, *J* = 3.5 Hz, 0.5H), 2.91 – 2.75 (m, 0.5H), 2.31 (d, *J* = 4.8 Hz, 1H), 2.19 – 2.02 (m, 1H), 1.86 (d, *J* = 15.1 Hz, 0.5H), 1.76 – 1.69 (m, 0.5H), 1.59 – 1.43 (m, 3.5H), 1.37 (d, *J* = 1.3 Hz, 1.5H), 1.32 – 1.12 (m, 4H).

^{13}C {^{1}H} NMR (101 MHz, $CDCl_3$) δ 141.4, 141.1, 140.8, 140.7, 128.8, 128.6, 128.43, 128.38, 128.34, 125.8, 121.2, 121.0, 41.29, 41.24, 41.2, 40.6, 39.8, 39.5, 38.0, 37.8, 37.0, 36.9, 29.6, 29.3, 28.8, 28.7, 18.4, 18.0.

HRMS (ESI-TOF): *m/z* calculated for $C_{18}H_{20}O_2F_3$ [M+CF3CO$_2$] $^-$ = 325.1421, found 325.1410.

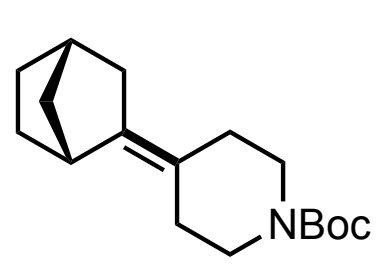

***tert*-butyl 4-((1R,4S)-bicyclo[2.2.1]heptan-2-ylidene)piperidine-1-carboxylate (P54)**

Prepared following general procedure A using (1R,2R,4S)-2-nitrobicyclo[2.2.1]heptane (**S26**, 0.15 mmol, 1.0 equiv.), *tert*-butyl 4-nitropiperidine-1-carboxylate (**S24**, 2.8 equiv.), and Cs_2CO_3 (3.7 equiv.) in 1,2-DFB/*t*-BuOH (4:1, 31.25 mM) at 28 °C. The title compound was isolated via flash column chromatography (0-10% EtOAc/Hexanes) as a white solid (17 mg, 42% yield).

TLC (SiO_2) R_f = 0.30 in 19:1 Hexanes:EtOAc.

^{1}H NMR (500 MHz, $CDCl_3$) δ 3.45 – 3.36 (m, 2H), 3.35 – 3.27 (m, 2H), 2.89 (d, *J* = 3.3 Hz, 1H), 2.36 (s, 1H), 2.21 (t, *J* = 5.9 Hz, 2H), 2.14 – 1.98 (m, 3H), 1.82 – 1.75 (m, 1H), 1.65 – 1.52 (m, 2H), 1.46 (s, 9H), 1.35 – 1.15 (m, 4H).

^{13}C {^{1}H} NMR (101 MHz, $CDCl_3$) δ 155.1, 138.3, 121.9, 79.4, 40.5, 39.3, 37.1, 36.6, 30.7, 30.2, 29.7, 28.7, 28.6.

HRMS (ESI-TOF): *m/z* calculated for $C_{17}H_{26}NO_2$ $[M-H]^-$ = 276.1969 found 276.1964.

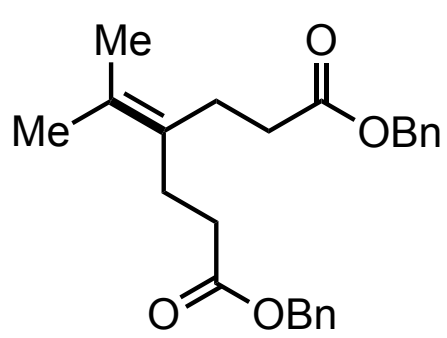

Dibenzyl 4-(propan-2-ylidene)heptanedioate (P55)

Prepared following general procedure A using dibenzyl 4-nitroheptanedioate (**S28**, 0.30 mmol, 1.0 equiv.), 2-nitropropane (**S27**, 2.8 equiv.), and Cs_2CO_3 (3.7 equiv.) in 1,2-DFB/*t*-BuOH (4:1, 31.25 mM) at 28 °C. The title compound was isolated via flash column chromatography (5–15% EtOAc/Hexanes) as a colorless oil (36 mg, 32% yield).

TLC (SiO_2) R_f = 0.50 in 9:1 Hexanes:EtOAc.

^{1}H NMR (500 MHz, $CDCl_3$) δ 7.35 (s, 10H), 5.10 (s, 4H), 2.37 (d, *J* = 2.7 Hz, 8H), 1.63 (s, 6H).

^{13}C {^{1}H} NMR (101 MHz, $CDCl_3$) δ 173.3, 136.2, 128.7, 128.5, 128.4, 128.3, 66.4, 33.5, 27.5, 20.4.

HRMS (ESI-TOF): *m/z* calculated for $C_{24}H_{26}O_3$ $[M-(H_2O)]^{\bullet-}$ = 362.1887, found 362.1887.

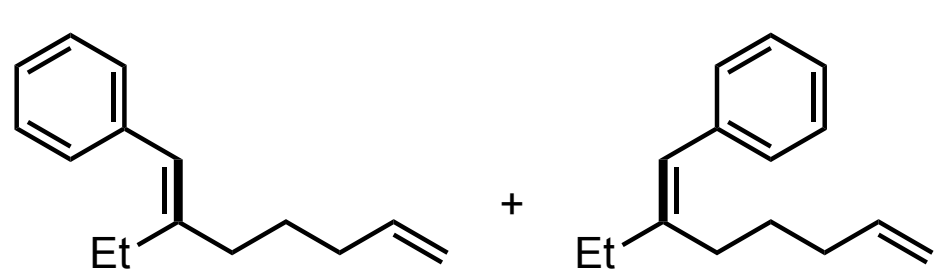

(*E*/*Z*)-(2-ethylhepta-1,6-dien-1-yl)benzene (P56)

Prepared following general procedure A using (nitromethyl)benzene (**S1**, 0.250 mmol, 1.0 equiv.), 6-nitrooct-1-ene (**S47**, 2.8 equiv.), and Cs_2CO_3 (3.7 equiv.) in 1,2-DFB/*t*-BuOH (9:1, 37.25 mM) at 28 °C. The title compound was isolated via flash column chromatography (Hexanes) as a colorless oil (21 mg, 43% yield, 1:1 *E*/*Z* mixture).

TLC (SiO_2) R_f = 0.40 in Hexanes

^{1}H NMR (500 MHz, $CDCl_3$) (as an E/Z mixture) δ 7.31 (t, *J* = 7.6 Hz, 2H), 7.20 (td, *J* = 7.9, 2.3 Hz, 3H), 6.28 (s, 0.5H), 6.25 (s, 0.5H), 5.92 – 5.74 (m, 1H), 5.09 – 4.90 (m, 2H), 2.32 – 2.23 (m,

2H), 2.23 – 2.17 (m, 2H), 2.16 – 2.09 (m, 1H), 2.05 (d, *J* = 7.2 Hz, 1H), 1.58 (ddd, *J* = 25.4, 15.6, 7.7 Hz, 2H), 1.12 (t, *J* = 7.5 Hz, 1.5H), 1.08 (t, J = 7.5 Hz, 1.5H).
^{13}C {^{1}H} NMR (101 MHz, $CDCl_3$) δ 145.0, 144.8, 139.0, 138.8, 138.7, 138.7, 128.8, 128.7, 128.2, 128.2, 126.0, 126.0, 124.8, 124.1, 114.8, 114.7, 36.3, 34.0, 33.7, 30.5, 30.1, 27.8, 27.5, 23.7, 13.2, 13.0.
HRMS (ESI-TOF): *m/z* calculated for $C_{15}H_{20}$ [M] $^{\bullet -}$ = 200.1570 found 200.1563.

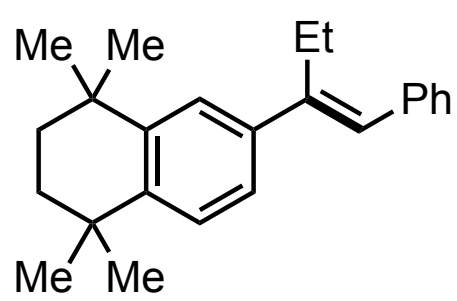

(*E*)-1,1,4,4-tetramethyl-6-(1-phenylbut-1-en-2-yl)-1,2,3,4-tetrahydronaphthalene (P57)

Prepared following general procedure A using (nitromethyl)benzene (**S1**, 0.100 mmol, 1.0 equiv.), 1,1,4,4-tetramethyl-6-(1-nitropropyl)-1,2,3,4-tetrahydronaphthalene (**S48**, 2.8 equiv.), and Cs_2CO_3 (3.7 equiv.) in 1,2-DFB/*t*-BuOH (4:1, 31.25 mM) at 28 °C. The title compound was isolated via flash column chromatography (0–10% DCM/Hexanes) as a white solid (11 mg, 33% yield).
TLC (SiO_2) R_f = 0.30 in 19:1 Hexanes:DCM.
^{1}H NMR (500 MHz, $CDCl_3$) δ 7.54 – 7.49 (m, 2H), 7.40 – 7.32 (m, 5H), 7.12 (s, 1H), 6.69 (s, 1H), 2.73 (q, *J* = 7.5 Hz, 2H), 1.70 (s, 4H), 1.32 (s, 6H), 1.30 (s, 6H), 1.09 (t, *J* = 7.5 Hz, 3H).
^{13}C {^{1}H} NMR (151 MHz, $CDCl_3$) δ 144.8, 144.6, 144.1, 137.5, 128.88, 128.86, 128.83, 128.4, 127.8, 126.7, 124.7, 123.9, 35.4, 35.3, 34.5, 34.3, 32.1, 32.0, 23.3, 13.8.
HRMS (ESI-TOF): *m/z* calculated for $C_{24}H_{29}$ [M-H] $^{-}$ = 317.2275, found 317.2290.

Starting Material Characterization

The spectral data for the following starting materials is in agreement with literature: [**S1-S4, S7**, **S10**, **S45**][52], **S5**[53], [**S6**, **S8**, **S13**] [54], **S9**[55], **S15**[56], [**S17**, **S18**][57], **S19**[58], [**S20**, **S22**, **S23**][59], [**S21**, **S26**][60], [**S24**, **S25**][61], **S32**[62], [**S33**, **S34**][63], **S36**[64], **S39**[65], [**S41**, **S43**][66], and **S44**[67].

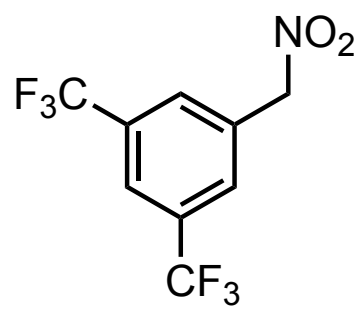

1-(nitromethyl)-3,5-bis(trifluoromethyl)benzene (S11)

Prepared following General Procedure E, the title compound was isolated via flash column chromatography on silica gel (0-10% EtOAc/Hexanes) and a colorless oil (523 mg, 50% yield).

TLC (SiO2) R_f = 0.30 in 9:1 Hexanes:DCM.

^{1}H NMR (500 MHz, $CDCl_3$) δ 7.99 (s, 1H), 7.94 (s, 2H), 5.57 (s, 2H).

^{13}C {^{1}H} NMR (126 MHz, $CDCl_3$) δ 133.0 (q, *J* = 34.0 Hz), 131.7, 130.5 (d, *J* = 3.8 Hz), 124.3 (septet, *J* = 3.8 Hz), 122.9 (d, *J* = 272.9 Hz), 78.5.

HRMS (ESI-TOF): *m/z* calculated for $C_9H_9F_6N_2O_2$ $[M+NH_4]^+$ = 291.0563, found 291.0568.

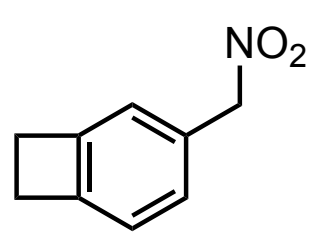

3-(nitromethyl)bicyclo[4.2.0]octa-1(6),2,4-triene (S12)

Prepared following General Procedure C, the title compound was isolated via flash column chromatography (0–5% DCM/Hexanes) as a colorless oil (648 mg, 79% yield).

TLC (SiO_2) R_f = 0.44 in 9:1 Hexanes:EtOAc.

^{1}H NMR (400 MHz, CD_3CN) δ 7.29 (dd, *J* = 7.5, 1.5 Hz, 1H), 7.21 – 7.14 (m, 1H), 7.12 (dd, *J* = 7.5, 0.9 Hz, 1H), 5.49 (s, 2H), 3.19 (s, 4H).

^{13}C {^{1}H} NMR (101 MHz, CD_3CN) δ 148.7, 147.5, 130.2, 130.1, 125.4, 123.9, 81.2, 30.0.

HRMS (ESI-TOF): *m/z* calculated for $C_9H_{13}N_2O_2$ $[M+NH_4]^+$ = 181.0972, found 181.0970.

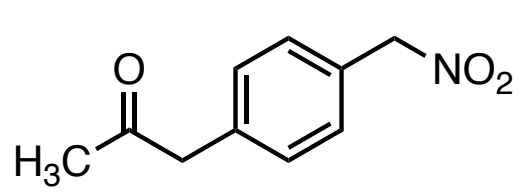

1-(4-(nitromethyl)phenyl)propan-2-one (S13)

Prepared following General Procedure C on a 1.5 mmol scale, the title compound was isolated via flash column chromatography (10–20% EtOAc/Hexanes) as a white solid (153 mg, 53% isolated).

TLC (SiO_2) R_f = 0.16 in 1:1 Hexanes:DCM.

^{1}H NMR (400 MHz, $CDCl_3$) δ 7.50 – 7.39 (m, 2H), 7.27 (d, *J* = 7.1 Hz, 2H), 5.43 (s, 2H), 3.75 (s, 2H), 2.19 (s, 3H).
^{13}C {^{1}H} NMR (101 MHz, $CDCl_3$) δ 205.6, 136.4, 130.5, 130.4, 128.7, 79.8, 50.6, 29.7.
HRMS (ESI-TOF): *m/z* calculated for $C_{12}H_{11}NO_4F_3$ $[M+CF_3CO_2]^-$ = 306.0595, found 306.0598.

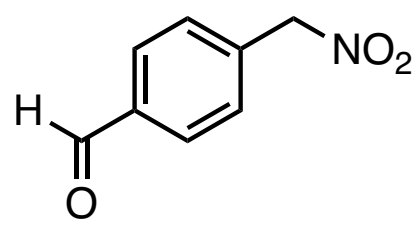

4-(nitromethyl)benzaldehyde (S14)
Prepared following General Procedure D, the title compound was isolated via flash column chromatography (10–20% EtOAc/Hexanes) as a white solid (63% yield).
TLC (SiO_2) R_f = 0.20 in 4:1 Hexanes:EtOAc.
^{1}H NMR (400 MHz, $CDCl_3$) δ 10.06 (s, 1H), 7.96 (d, *J* = 8.2 Hz, 2H), 7.64 (d, *J* = 8.2 Hz, 2H), 5.53 (s, 2H).
^{13}C {^{1}H} NMR (101 MHz, $CDCl_3$) δ 191.48, 137.46, 135.43, 130.82, 130.41, 79.55.
HRMS (ESI-TOF): *m/z* calculated for $C_9H_8NO_5$ $[M+HCO_2]^-$ = 210.0408, found 210.0408.

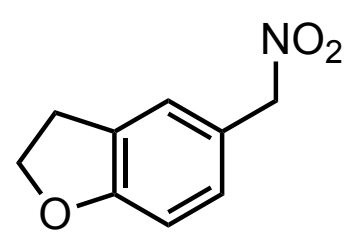

5-(nitromethyl)-2,3-dihydrobenzofuran (S16)
Prepared following General Procedure C on a 2.0 mmol scale, the title compound was isolated via flash column chromatography (0–5% EtOAc/Hexanes) as a colorless oil (222 mg, 62% yield).
TLC (SiO_2) R_f = 0.24 in 19:1 EtOAc in Hexanes.
^{1}H NMR (400 MHz, $CDCl_3$) δ 7.29 (s, 1H), 7.19 (dd, *J* = 8.1, 1.9 Hz, 1H), 6.80 (d, *J* = 8.2 Hz, 1H), 5.35 (s, 2H), 4.62 (t, *J* = 8.7 Hz, 2H), 3.24 (t, *J* = 8.7 Hz, 2H).
^{13}C {^{1}H} NMR (101 MHz, $CDCl_3$) δ 161.7, 130.7, 128.3, 122.0, 109.1, 80.1, 71.8, 29.5.
HRMS (ESI-TOF): *m/z* calculated for $C_{10}H_9NO_3$ $[M+HCO_2]^-$ = 210.0534, found 210.0536.

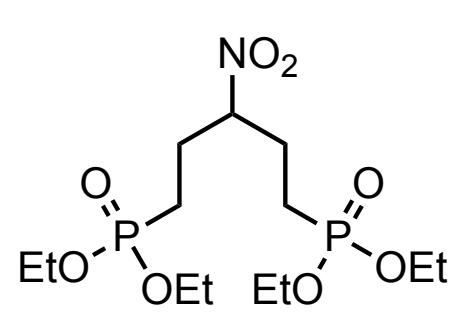

tetraethyl (3-nitropentane-1,5-diyl)bis(phosphonate) (S29)
A solution of 1-[ethoxy(vinyl)phosphoryl]oxyethane (492 mg, 463 µL, 1.0 equiv., 3.00 mmol) and nitromethane (916 mg, 809 µL, 5 equiv., 15.0 mmol) was treated with TBAF (157 mg, 0.20 equiv., 60.0 mmol) and stirred at room temperature for 18 h. The reaction mixture was then washed with water (2 × 200 mL). and concentrated under reduced pressure to leave a yellow oil. The title compound was isolated via flash column chromatography (0–2% MeOH/DCM) as a light brown oil (128 mg, 11% yield).

TLC (SiO_2) R_f = 0.50 in 19:1 DCM:MeOH.
^{1}H NMR (600 MHz, $CDCl_3$) δ 4.63 (tt, *J* = 9.2, 4.2 Hz, 1H), 4.18 – 4.02 (m, 8H), 2.29 – 2.18 (m, 2H), 2.12 – 2.02 (m, 4H), 1.32 (t, *J* = 7.0 Hz, 12H).
^{13}C {^{1}H} NMR (151 MHz, $CDCl_3$) δ 88.3 (t, *J* = 15.8 Hz), 62.13 (d, *J* = 2.7 Hz), 62.08 (d, *J* = 2.6 Hz), 27.0 (d, *J* = 4.2 Hz), 22.3 (d, *J* = 143.5 Hz), 16.59 (d, *J* = 1.6 Hz), 16.55 (d, *J* = 1.7 Hz).
^{31}P {^{1}H} NMR (202 MHz, $CDCl_3$) δ 28.76.
HRMS (ESI-TOF): *m/z* calculated for $C_{13}H_{29}NO_8P_2$ [M+Na-(H_2O)] $^+$ = 394.115, found 394.1136.

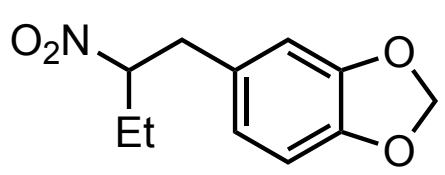

5-(2-nitrobutyl)benzo[d][1,3]dioxole (*S*30)
Prepared following General Procedure E, the title compound was isolated via flash column chromatography (0–5% EtOAc/Hexanes) in 72% yield.
TLC (SiO_2) R_f = 0.65 in 4:1 Hexanes:EtOAc.
^{1}H NMR (500 MHz, $CDCl_3$) δ 6.73 (d, *J* = 7.8 Hz, 1H), 6.63 (s, 1H), 6.60 (d, *J* = 7.8 Hz, 1H), 5.93 (s, 2H), 4.60 – 4.52 (m, 2H), 3.16 (dd, *J* = 14.3, 8.7 Hz, 1H), 2.94 (dd, *J* = 14.3, 5.7 Hz, 1H), 2.06 – 1.93 (m, 1H), 1.88 – 1.77 (m, 1H), 0.98 (t, *J* = 7.4 Hz, 3H).
^{13}C {^{1}H} NMR (101 MHz, $CDCl_3$) δ 148.1, 147.0, 129.4, 122.2, 109.3, 108.6, 101.2, 91.8, 39.6, 26.9, 10.4.
HRMS (ESI-TOF): *m/z* calculated for $C_{13}H_{13}NO_6F_3$ [M+CF_3CO_2] $^-$ = 336.0700, found 336.0689.

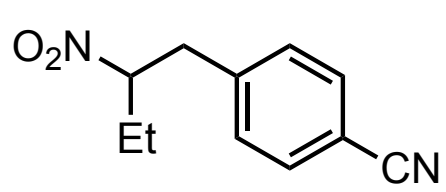

4-(2-nitrobutyl)benzonitrile (*S*31)
Prepared following General Procedure E, the title compound was isolated via flash column chromatography (0–5% EtOAc/Hexanes) in 80% yield.
TLC (SiO_2) R_f = 0.375 in 4:1 Hexanes:EtOAc.
^{1}H NMR (500 MHz, CD_2Cl_2) δ 7.62 (d, *J* = 7.4 Hz, 2H), 7.30 (d, *J* = 7.4 Hz, 2H), 4.70 – 4.59 (m, 1H), 3.30 (dd, *J* = 14.5, 9.4 Hz, 1H), 3.11 (dd, *J* = 14.5, 4.6 Hz, 1H), 2.10 – 1.97 (m, 1H), 1.94 – 1.81 (m, 1H), 0.99 (t, *J* = 8.6 Hz, 3H).
^{13}C {^{1}H} NMR (151 MHz, $CDCl_3$) δ 141.7, 133.0, 130.1, 118.9, 111.8, 91.2, 39.8, 27.5, 10.4.
HRMS (ESI-TOF): *m/z* calculated for $C_{13}H_{15}N_2O_4$ [M+CH_3CO_2] $^-$ = 263.1037, found 263.1029.

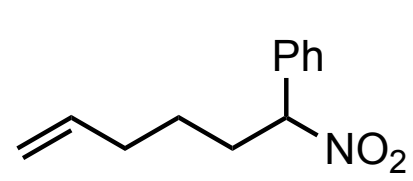

(1-nitrohex-5-en-1-yl)benzene (*S*37)

Prepared according to General Procedure C as substrate **S12** with 6-nitrohex-1-ene (2.00 mmol) and bromobenzene (2.20 mmol). The title compound was isolated via flash column chromatography (Hexanes) as a colorless oil (112 mg, 48% yield).
^{1}H NMR (500 MHz, $CDCl_3$) δ 7.47 (dd, *J* = 6.8, 3.1 Hz, 2H), 7.41 (q, *J* = 3.7 Hz, 3H), 5.75 (dd, *J* = 17.0, 10.3, 6.7 Hz, 1H), 5.45 (dd, *J* = 8.8, 6.5 Hz, 1H), 5.05-4.97 (m, 2H), 2.49 (dddd, *J* = 14.2, 10.2, 8.8, 5.4 Hz, 1H), 2.15-2.05 (m, 3H), 1.43 (dddd, *J* = 21.5, 11.2, 4.6, 1.8 Hz, 2H).
^{13}C {^{1}H} NMR (126 MHz, $CDCl_3$) δ 137.5, 134.7, 130.0, 129.1, 127.8, 115.7, 91.6, 33.4, 33.1, 25.3.
HRMS (ESI-TOF): *m/z* calculated for $C_{12}H_{14}NO$ [M+H-(H_2O)] $^+$ = 188.1070, found 188.1064.

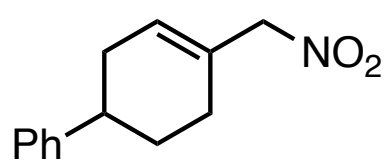

4-(nitromethyl)-1,2,3,6-tetrahydro-1,1'-biphenyl (S39) [68]
A solution of 4-phenylcyclohexan-1-one (1.74 g, 1 equiv., 10.0 mmol) and 1,2-diaminoethane (0.05 equiv.) in nitromethane (6.67 mL) was heated to 110 °C for 10 h under N_2 atmosphere. The title compound was isolated by flash column chromatography (0–5% EtOAc/Hexanes) as a fluffy white solid (1.51 g, 70% yield).
TLC (SiO_2) R_f = 0.13 in Hexanes.
^{1}H NMR (400 MHz, $CDCl_3$) δ 7.40 – 7.28 (m, 2H), 7.24 – 7.17 (m, 3H), 6.19 – 5.85 (m, 1H), 4.88 (s, 2H), 2.82 (ddd, *J* = 18.7, 8.0, 2.8 Hz, 1H), 2.44 (d, *J* = 18.6 Hz, 1H), 2.38 – 2.18 (m, 3H), 2.14 – 1.97 (m, 1H), 1.96 – 1.73 (m, 1H).
^{13}C {^{1}H} NMR (101 MHz, $CDCl_3$) δ 146.1, 133.1, 128.7, 128.6, 127.0, 126.5, 82.5, 39.3, 33.6, 29.5, 27.4.

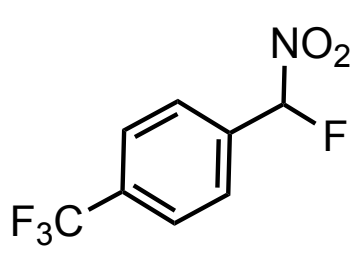

1-(fluoro(nitro)methyl)-4-(trifluoromethyl)benzene (S42)
Prepared using 1.1 mL of aqueous 0.5 N KOH solution added to a mixture of 1-(nitromethyl)-4-(trifluoromethyl)benzene (317.2 mg, 1 Eq, 1.546 mmol) in acetonitrile (5 mL) and water (5 mL). The reaction mixture was stirred at 25 °C for 2 h. Selectfluor™ (821.7 mg, 1.5 equiv., 2.319 mmol) was added to the reaction mixture in one portion, and stirring was continued for 16 h. The resulting mixture was poured into 20 mL of water and extracted with methylene chloride (3 × 15 mL). The extracts were washed with 20 mL of brine, dried (Na_2SO_4), and filtered. The solvent was evaporated, and the resulting residue was chromatographed. The title compound was isolated via flash column chromatography (0–5% EtOAc/Hexanes) as a colorless oil (300 mg, 87% yield).
TLC (SiO_2) R_f = 0.66 in 1:1 Hexanes:DCM.
^{1}H NMR (500 MHz, $CDCl_3$) δ 7.82 – 7.71 (m, 4H), 6.68 (d, J = 48.4 Hz, 1H).

^{13}C {^{1}H} NMR (126 MHz, $CDCl_3$) δ 134.2 (q, *J* = 32.9 Hz), 133.8 (d, *J* = 21.1 Hz), 127.2 (d, *J* = 6.6 Hz), 126.4 (q, *J* = 3.8 Hz), 123.5 (q, *J* = 272.9 Hz), 109.0 (d, *J* = 240.9 Hz).
^{19}F {^{1}H} NMR (471 MHz, $CDCl_3$) δ -63.28, -142.36.
HRMS (ESI-TOF): *m/z* calculated for $C_9H_6F_4NO_4$ $[M+HCO_2]^-$ = 268.0233 found 268.0233.

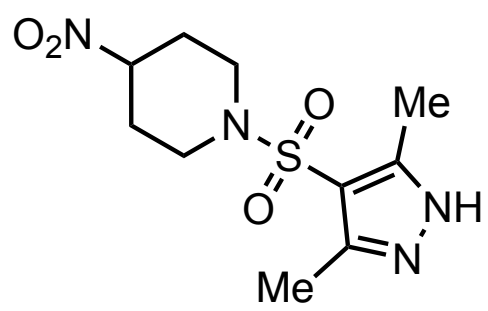

1-((3,5-dimethyl-1*H*-pyrazol-4-yl)sulfonyl)-4-nitropiperidine (S46)
To a stirred solution of 4-nitropiperidine hydrochloride (1.88 g, 1.0 equiv., 11.3 mmol) dissolved in DCM (18.8 mL) was added 3,5-dimethyl-1H-pyrazole-4-sulfonylchloride (2.42 g, 1.1 equiv., 12.4 mmol) and triethylamine (1.71 g, 2.36 mL, 1.5 equiv., 16.9 mmol) at 0 °C simultaneously and stirred at room temperature for 6 h. The reaction mixture was quenched with ice water (100 mL), extracted with DCM (2 x 100 mL), organic layers were washed with water (2 x 100 ml), brine solution (100 mL), dried over sodium sulfate (Na_2SO_4), and evaporated under reduced pressure. The resulting residue was chromatographed the title compound was isolated via flash column chromatography (0–50% EtOAc/Hexanes) as a white solid (1.70 g, 51% yield).
TLC (SiO_2) R_f = 0.33 in 19:1 DCM:MeOH.
^{1}H NMR (500 MHz, CD_2Cl_2) δ 4.47 – 4.36 (m, 1H), 3.57 – 3.46 (m, 2H), 2.91 – 2.78 (m, 2H), 2.42 (s, 6H), 2.35 – 2.15 (m, 4H).
^{13}C {^{1}H} NMR (151 MHz, CD_2Cl_2) δ 147.0, 112.7, 80.9, 43.5, 29.5, 12.7.
HRMS (ESI-TOF): *m/z* calculated for $C_{10}H_{16}N_4O_4SBr$ $[M+Br]^-$ = 335.0360, found 335.0367.

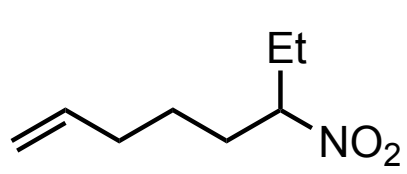

6-nitrooct-1-ene (S47) [69]
Prepared according to a modified literature procedure. A suspension of magnesium (134 mg, 80.6 µL, 1.1 equiv., 5.50 mmol) in THF (20.0 mL) was treated with 5-bromopent-1-ene (745 mg, 1 equiv., 5.00 mmol), and the resulting Grignard reagent was refluxed for 1 h. At 0 °C, propionaldehyde (871 mg, 1.08 mL, 3 equiv., 15.0 mmol) was added dropwise over 10 min, and the reaction mixture was stirred for 2 hours at 23 °C. Following aqueous workup, the crude alcohol product was carried forward to the next step. The alcohol was converted to bromide using phosphorus tribromide and then to nitroalkane using $NaNO_2$. The title compound was isolated via flash column chromatography (Hexanes) as a yellow oil (340 mg, 43% yield).
TLC (SiO_2) R_f = 0.3 in Hexanes.

^{1}H NMR (500 MHz, $CDCl_3$) δ 5.75 (ddt, *J* = 17.0, 10.2, 6.7 Hz, 1H), 5.06-4.94 (m, 2H), 4.39 (tt, *J* = 9.3, 4.6 Hz, 1H), 2.11-2.05 (m, 2H), 2.01-1.92 (m, 2H), 1.81-1.67 (m, 2H), 1.45-1.38 (m, 2H), 0.95 (t, *J* = 7.4 Hz, 3H).
^{13}C {^{1}H} NMR (126 MHz, $CDCl_3$) δ 137.6, 115.6, 90.4, 33.1, 33.0, 27.3, 25.1, 10.4.
HRMS (ESI-TOF): *m/z* calculated for $C_8H_{13}NO$ [M-(H_2O)] $^{\bullet-}$ = 139.1003, found 139.1000.

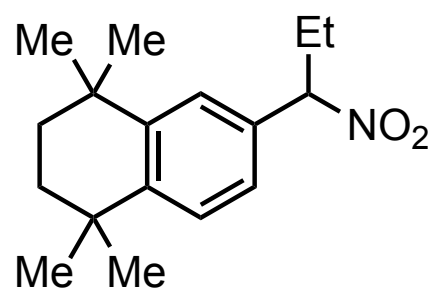

1,1,4,4-tetramethyl-6-(1-nitropropyl)-1,2,3,4-tetrahydronaphthalene (S48)
Prepared following General Procedure C with 6-bromo-1,1,4,4-tetramethyl-1,2,3,4-tetrahydronaphthalene (2.50 mmol, 1.0 equiv.) and 1-nitropropane (10.0 equiv.). The title compound was isolated via flash column chromatography (Hexanes) as a yellow oil (390 mg, 56% yield).
TLC (SiO_2) R_f = 0.4 in 10% DCM/Hexanes.
^{1}H NMR (500 MHz, $CDCl_3$) δ 7.36-7.30 (m, 2H), 7.22 (dd, *J* = 8.2, 2.1 Hz, 1H), 5.33 (dd, *J* = 9.4, 5.9 Hz, 1H), 2.51 (ddq, *J* = 14.8, 9.4, 7.2 Hz, 1H), 2.07 (dtd, *J* = 14.8, 7.5, 5.9 Hz, 1H), 1.68 (s, 4H), 1.30-1.26 (m, 12H), 1.00 (t, *J* = 7.3 Hz, 3H).
^{13}C NMR (126 MHz, $CDCl_3$) δ 146.8, 145.7, 131.8, 127.3, 126.2, 124.6, 93.4, 35.1, 35.0, 34.5, 34.4, 31.9, 31.9, 31.9, 31.9, 27.4, 10.9.
HRMS (ESI-TOF): *m/z* calculated for $C_{17}H_{23}NO$ [M-(H_2O)] $^{\bullet-}$ = 257.1785, found 257.1778.

NMR Spectra of Olefination Products

^{1}H NMR of (cyclohexylidenemethyl)benzene (P1)

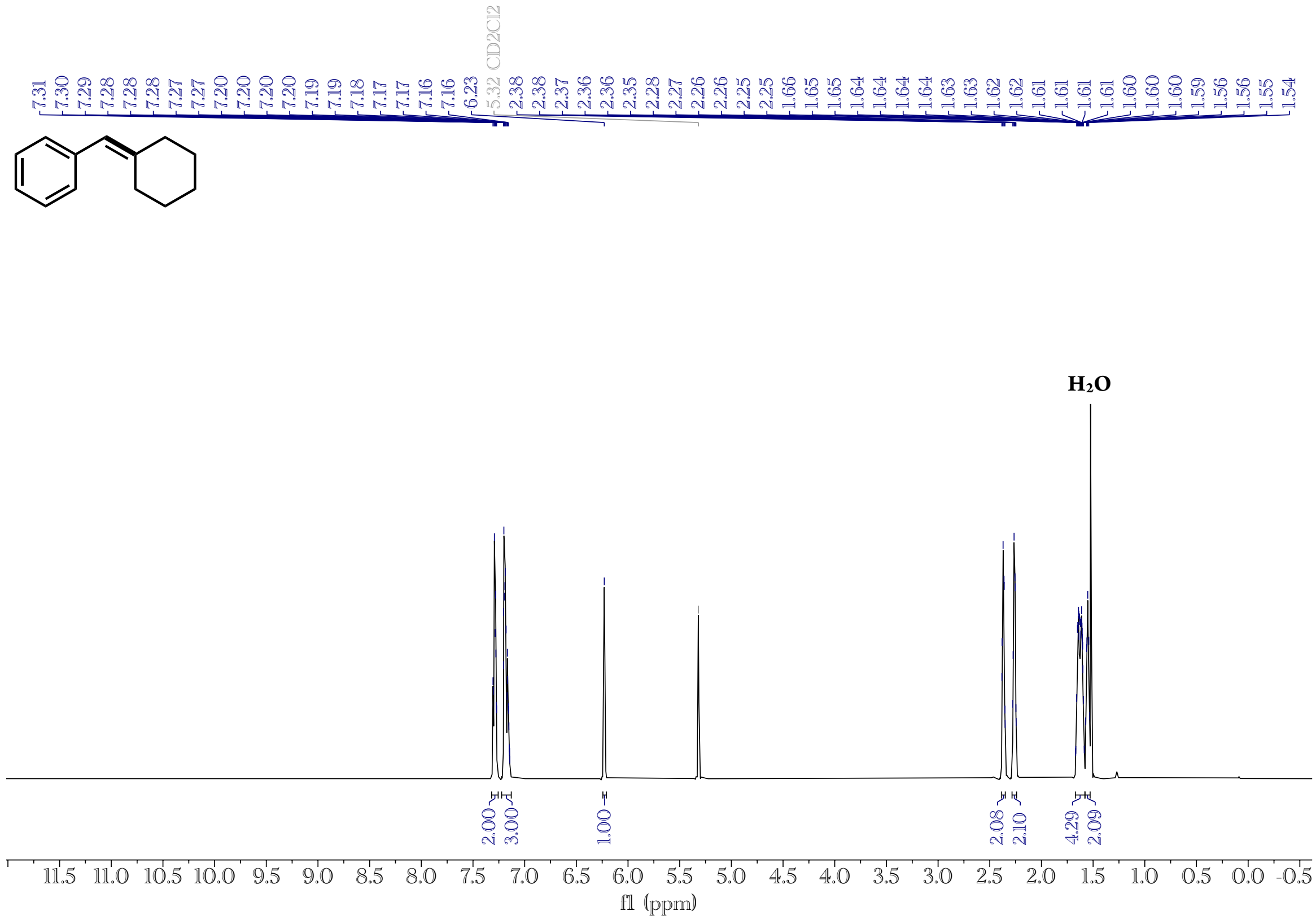

^{13}C {^{1}H} NMR of (cyclohexylidenemethyl)benzene (P1)

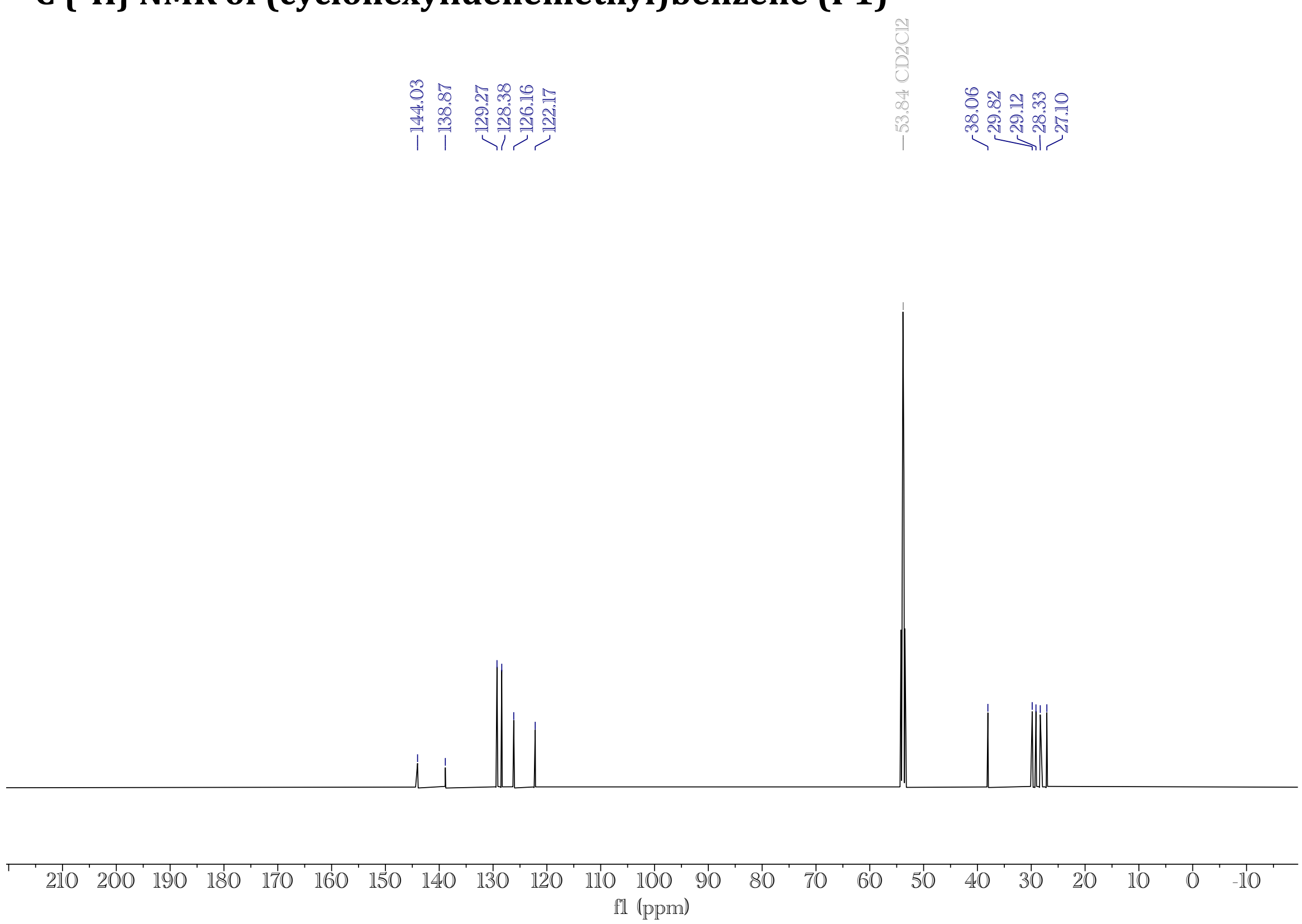

^{1}H NMR of 1-chloro-4-(cyclohexylidenemethyl)benzene (P2)

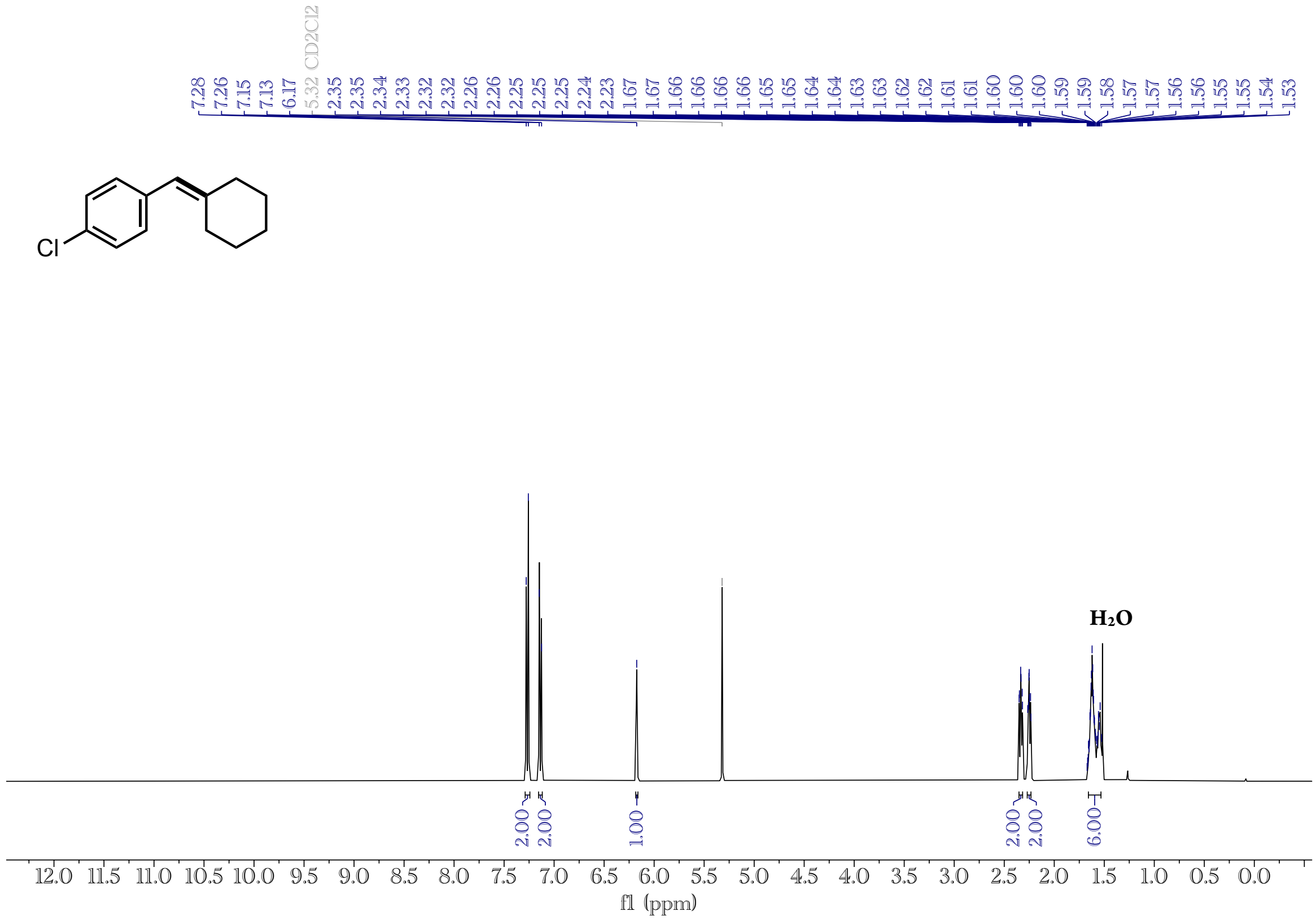

^{13}C {^{1}H} NMR of 1-chloro-4-(cyclohexylidenemethyl)benzene(P2)

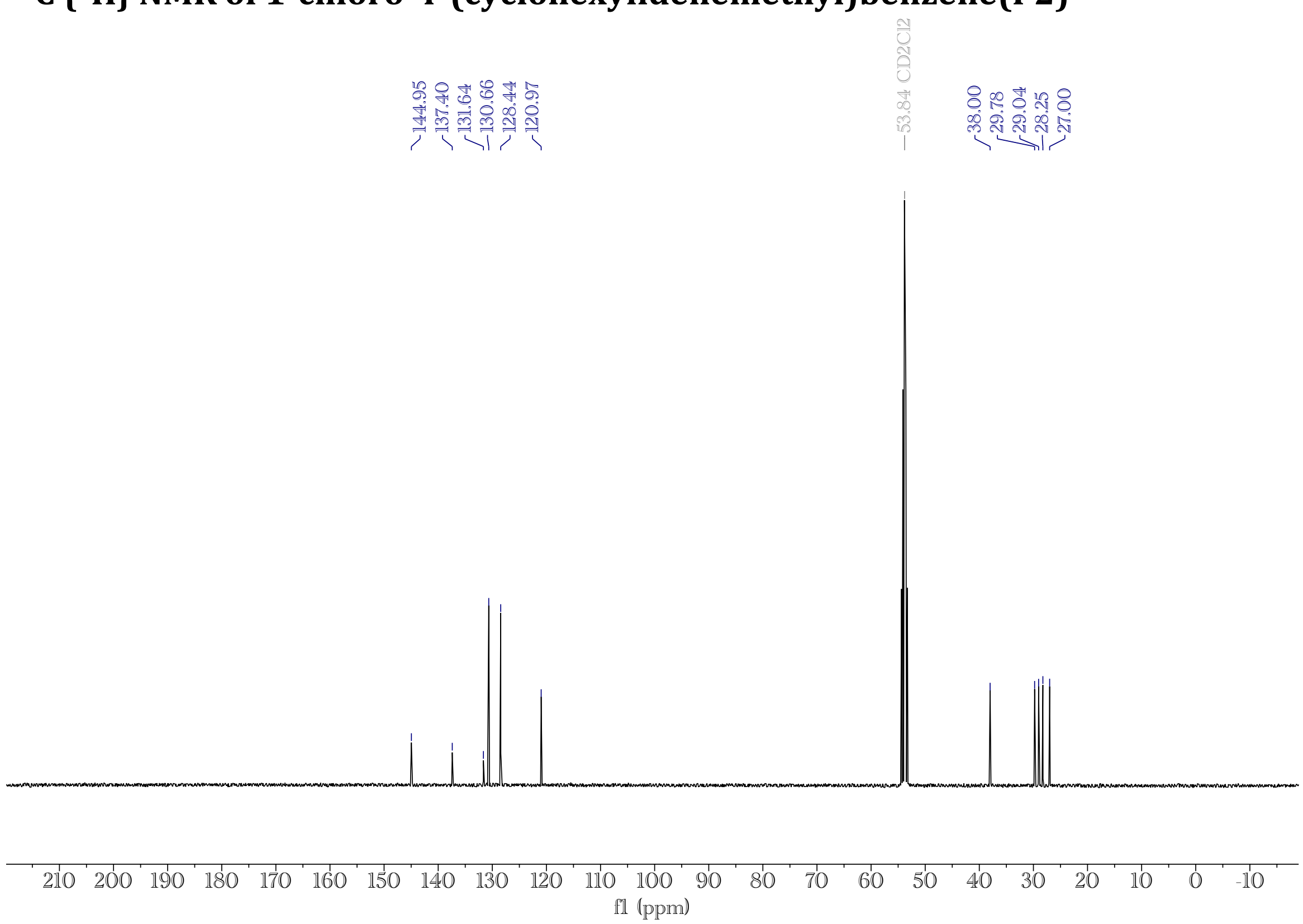

^{1}H NMR of 1-(cyclohexylidenemethyl)-4-(trifluoromethyl)benzene (P3)

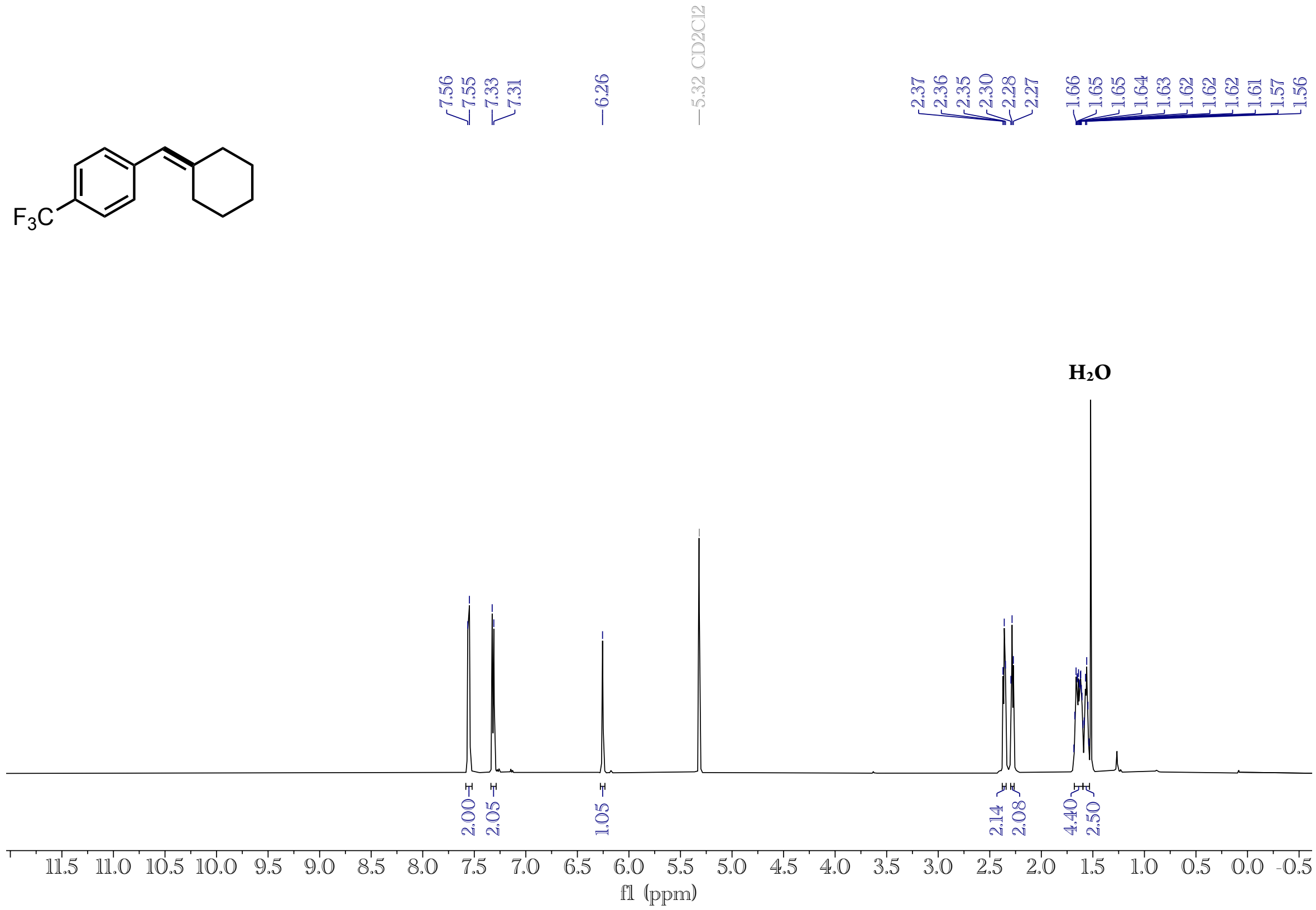

^{13}C {^{1}H} NMR of 1-(cyclohexylidenemethyl)-4-(trifluoromethyl)benzene (P3)

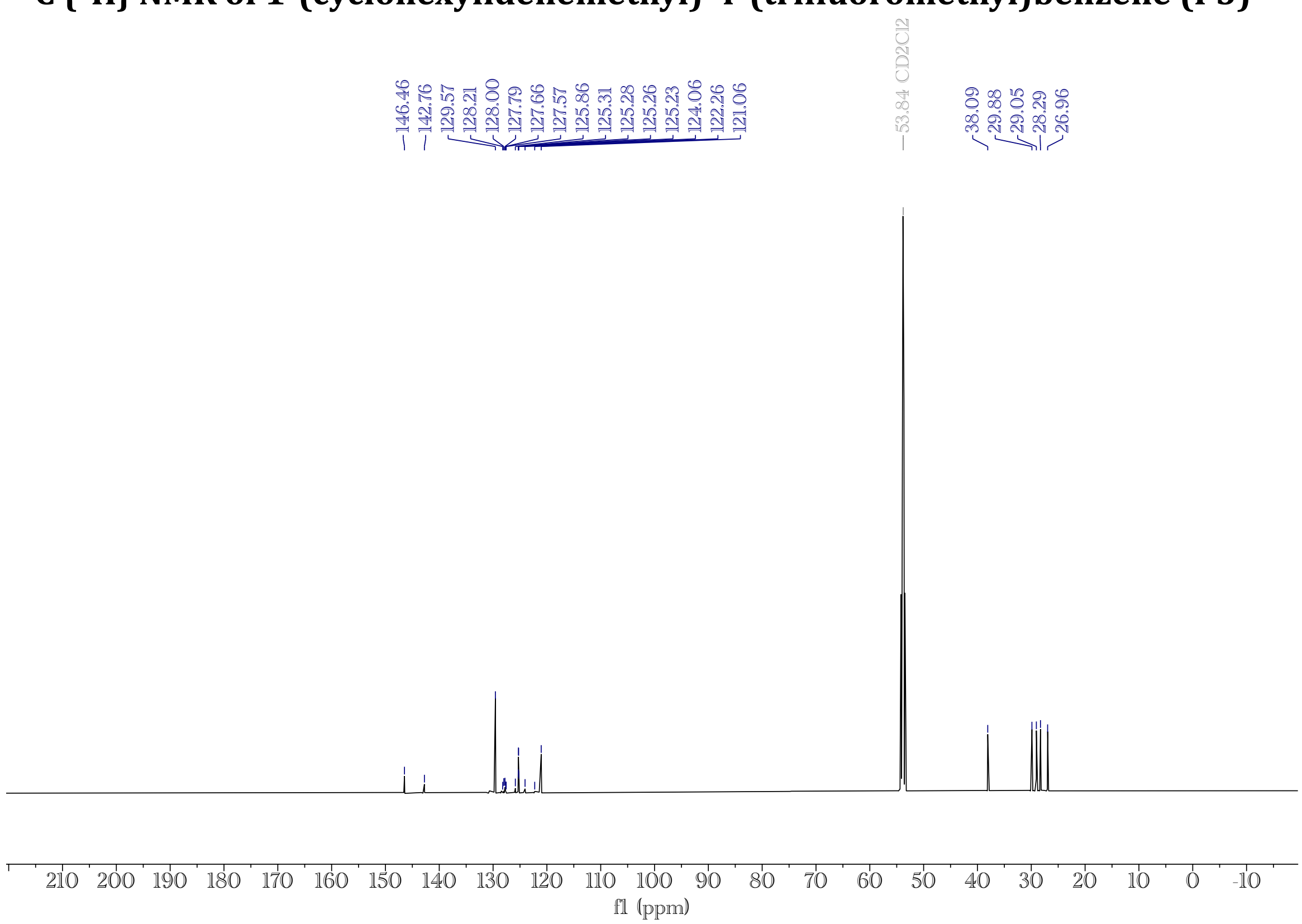

$^{19}F\ \{^{1}H\}$ NMR of 1-(cyclohexylidenemethyl)-4-(trifluoromethyl)benzene (P3)

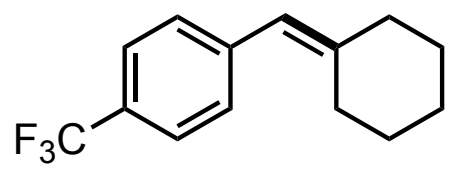

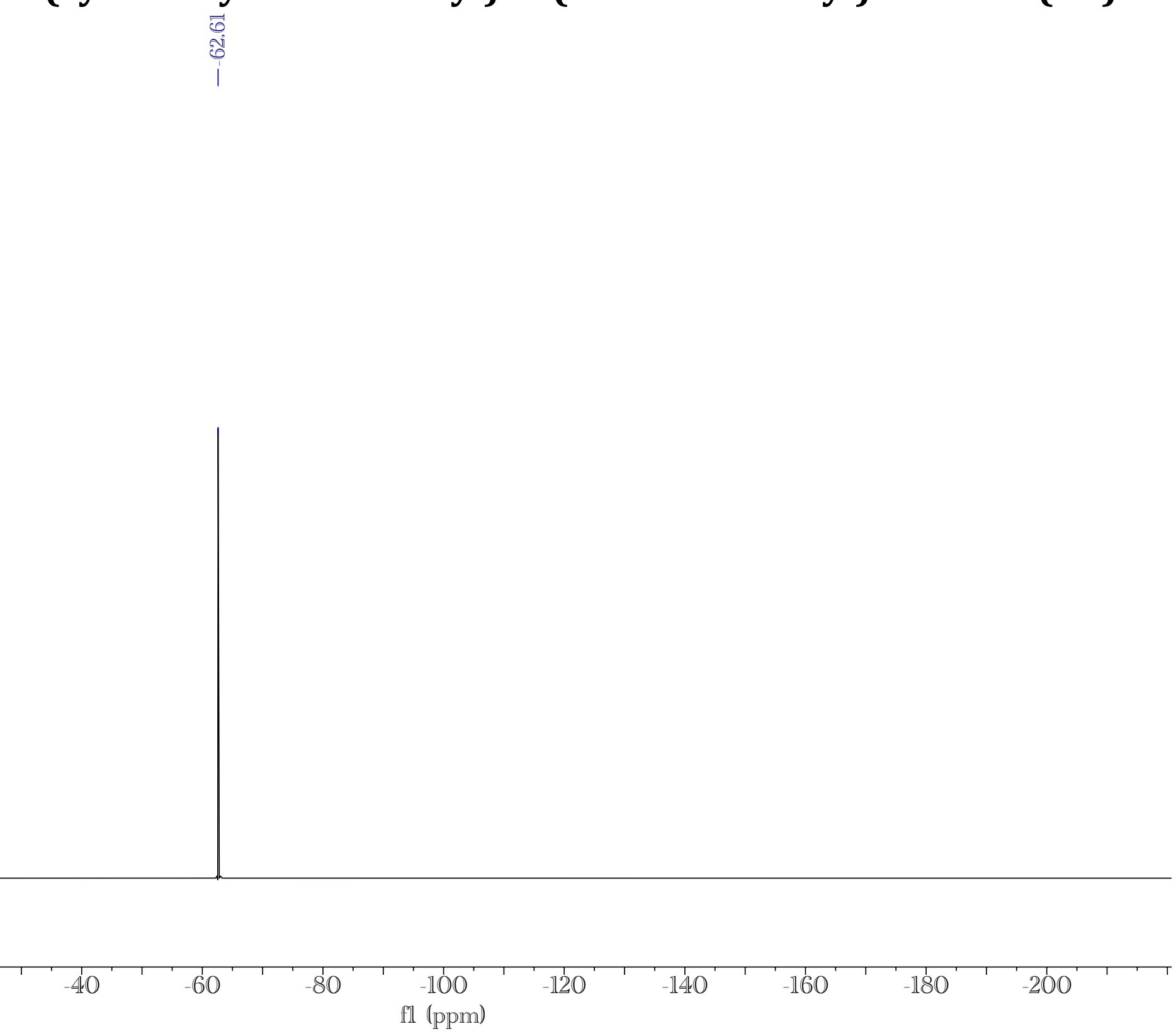

^{1}H NMR of Methyl 4-(cyclohexylidenemethyl)benzoate (P4)

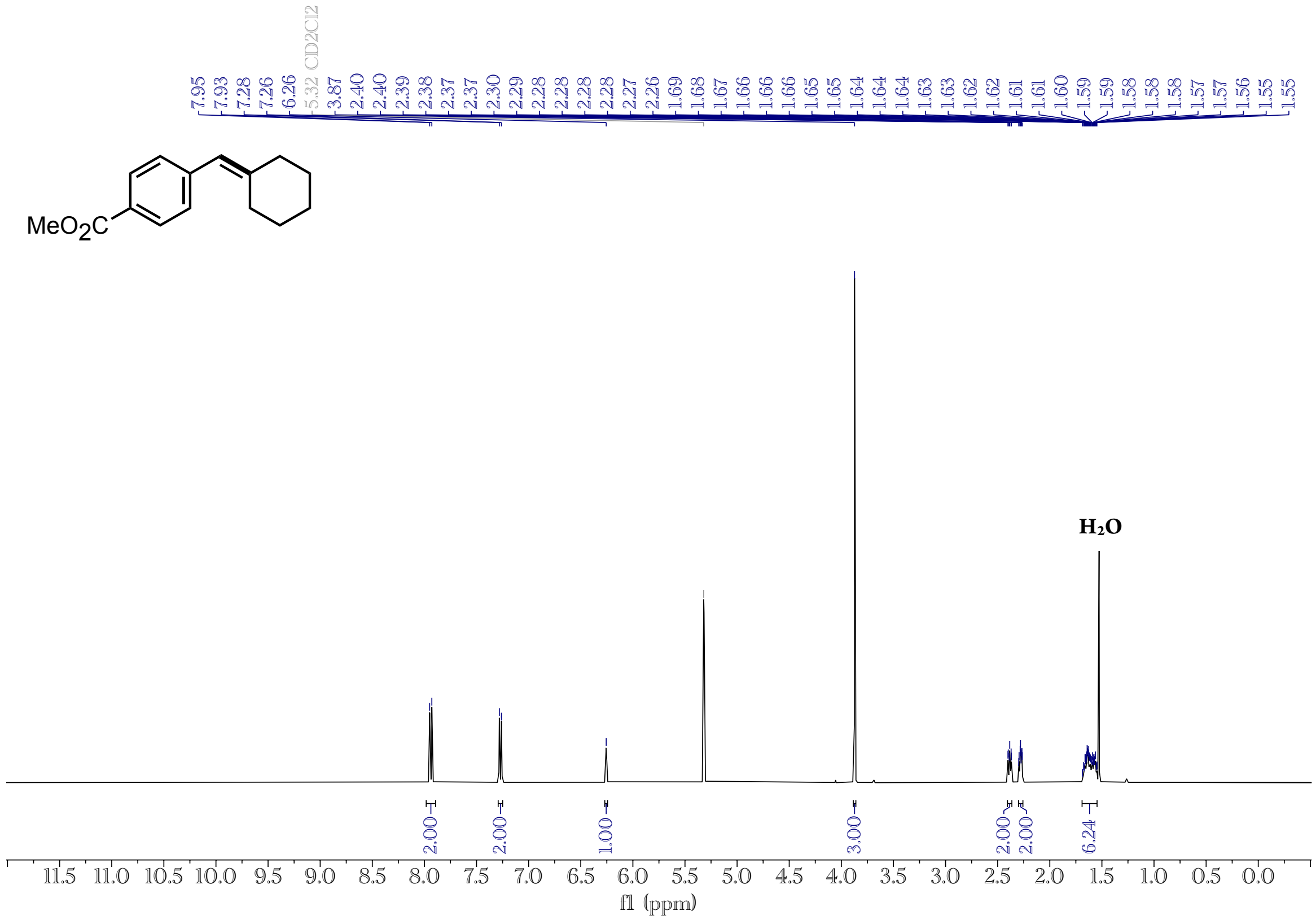

^{13}C {^{1}H} NMR of Methyl 4-(cyclohexylidenemethyl)benzoate (P4)

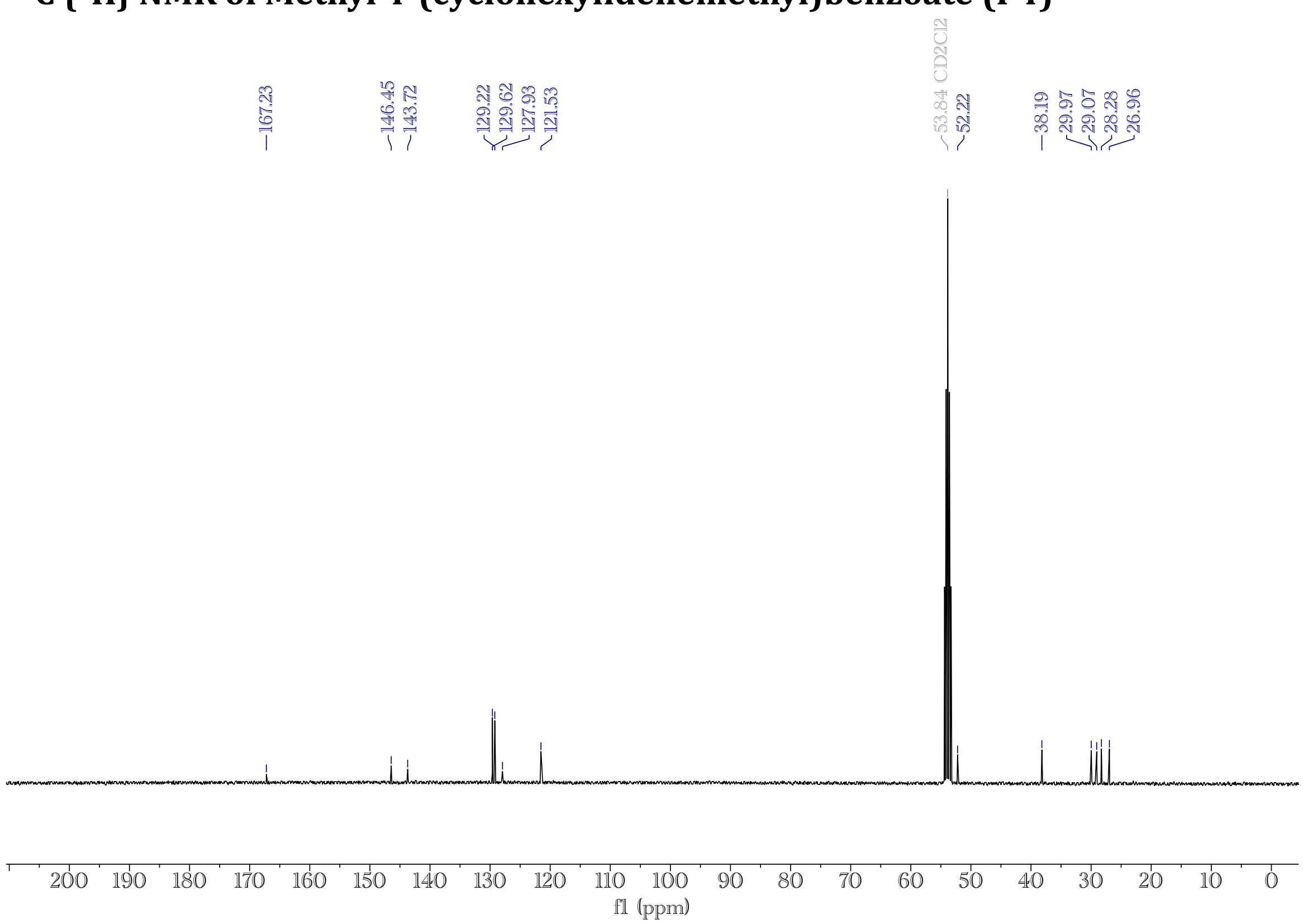

^{1}H NMR of 4-(cyclohexylidenemethyl)-1,1'-biphenyl (P5)

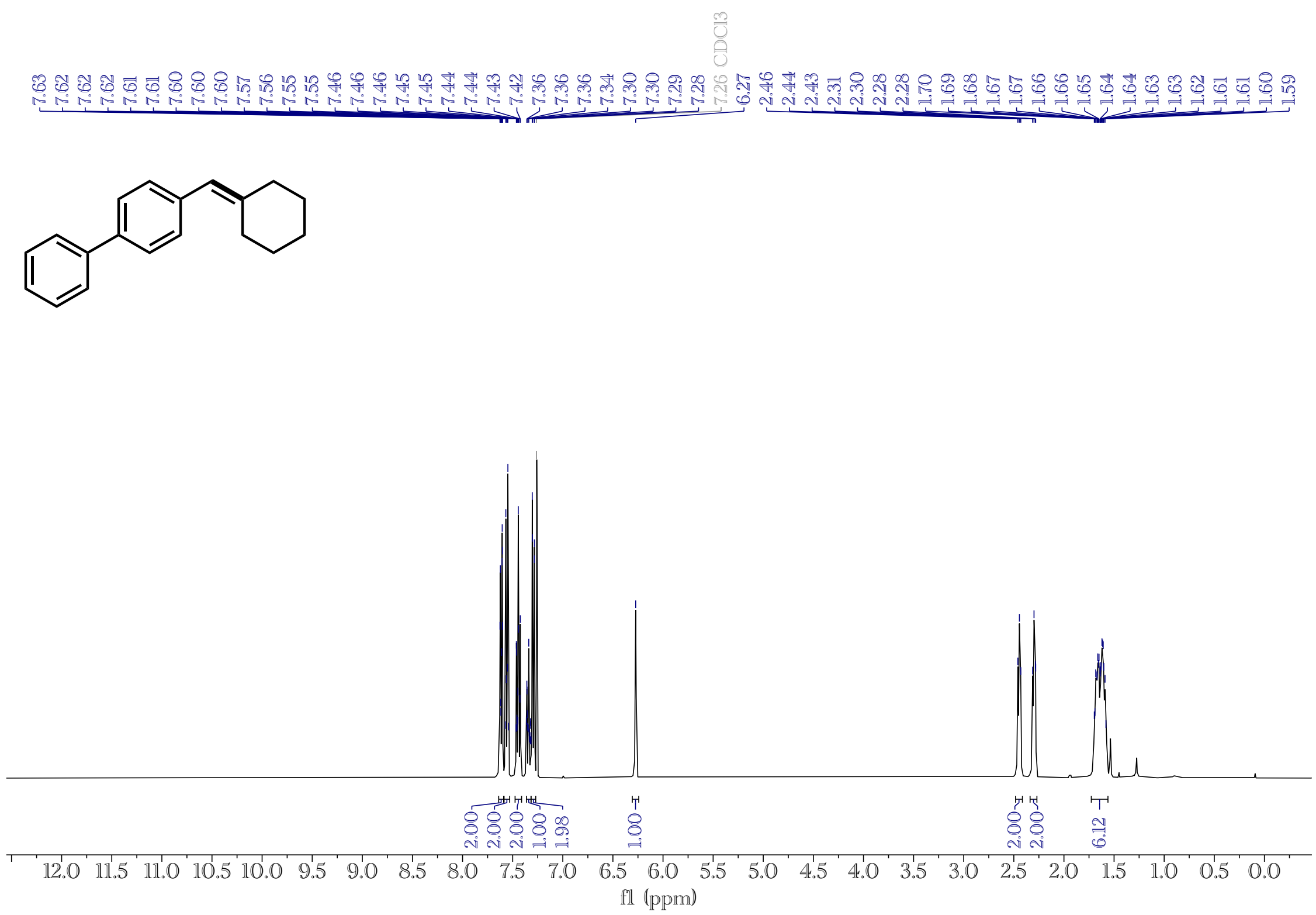

^{13}C {^{1}H} NMR of 4-(cyclohexylidenemethyl)-1,1'-biphenyl (P5)

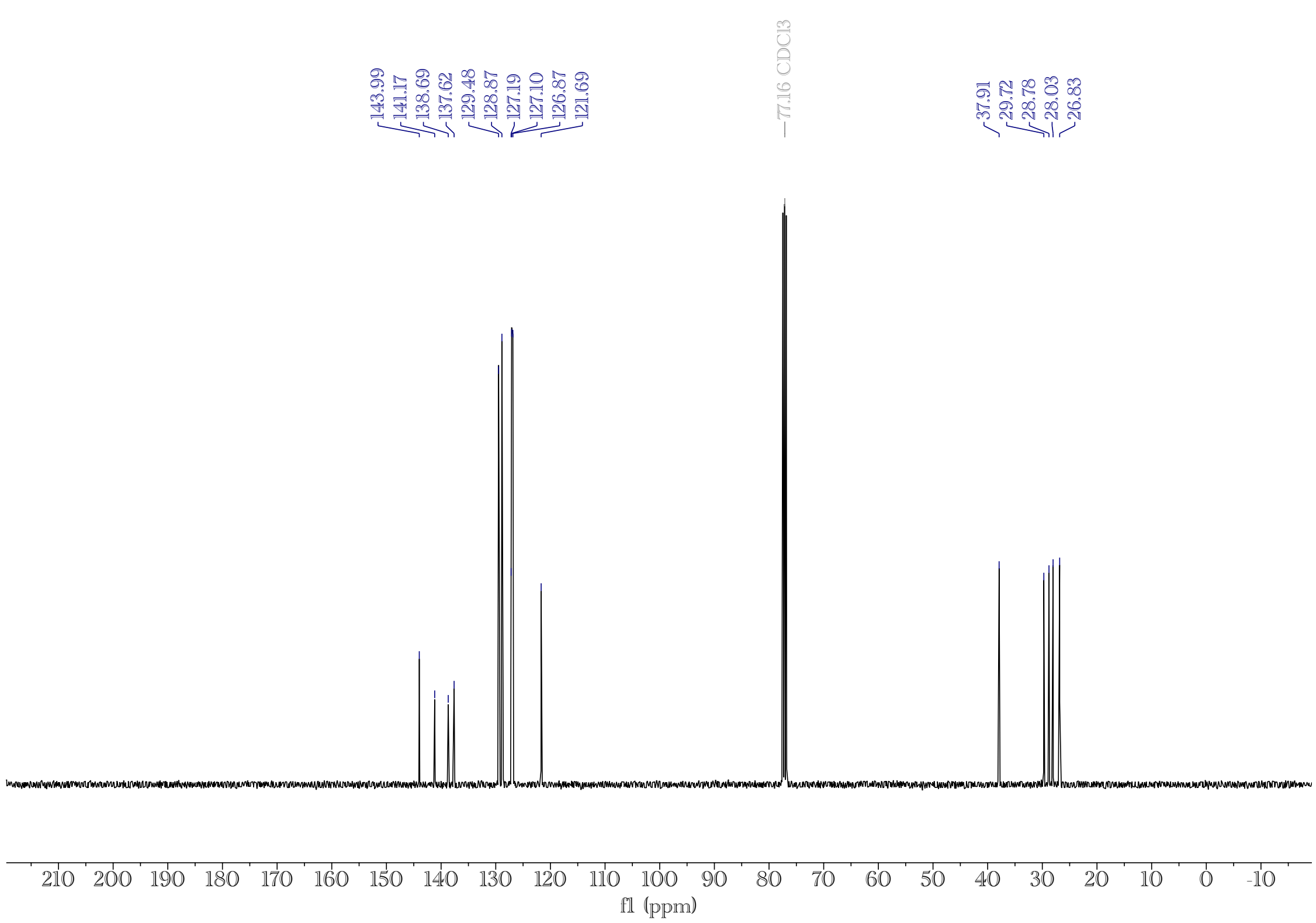

^{1}H NMR of 1-(tert-butyl)-4-(cyclohexylidenemethyl)benzene (P7)

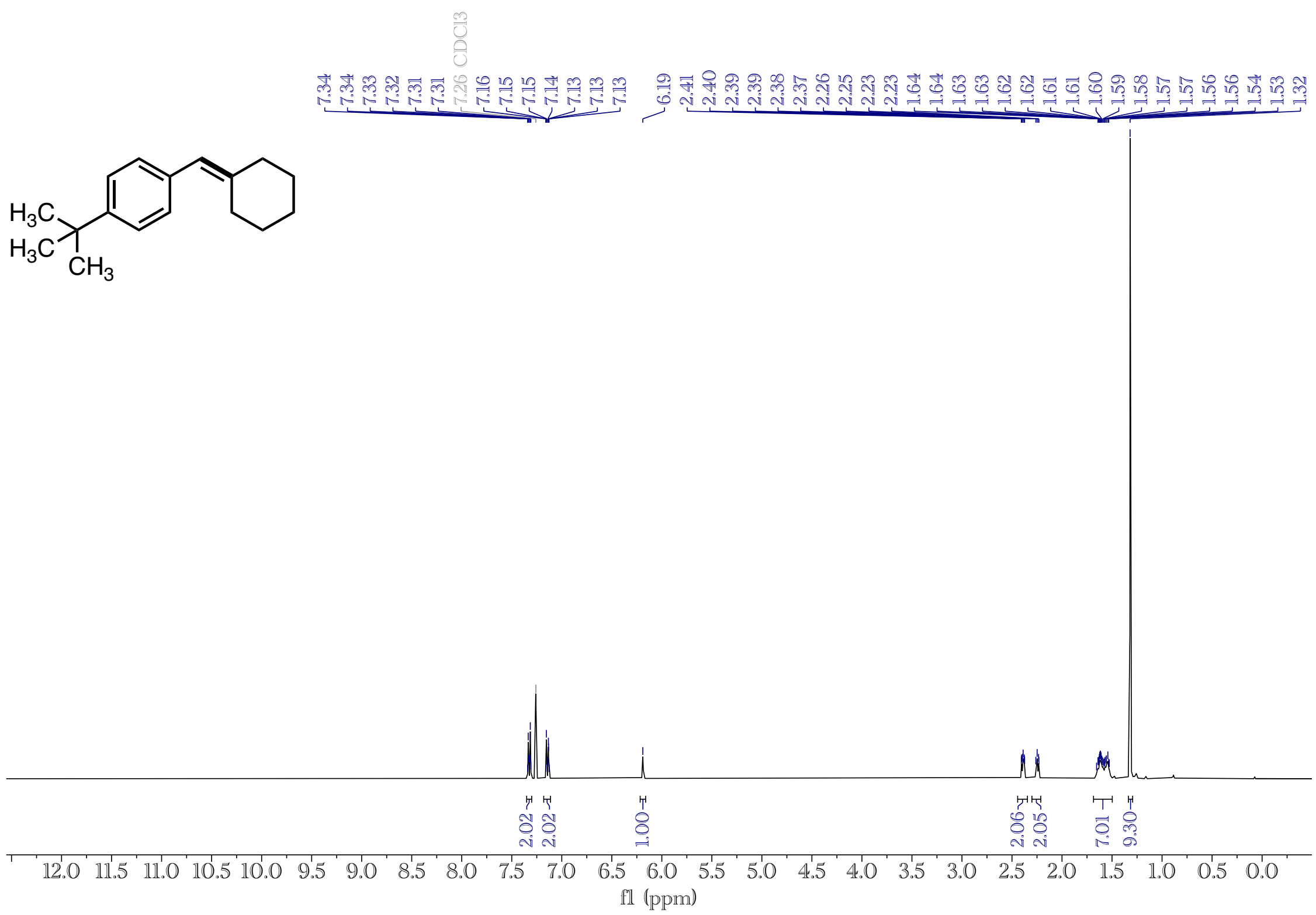

^{13}C {^{1}H} NMR of 1-(tert-butyl)-4-(cyclohexylidenemethyl)benzene (P7)

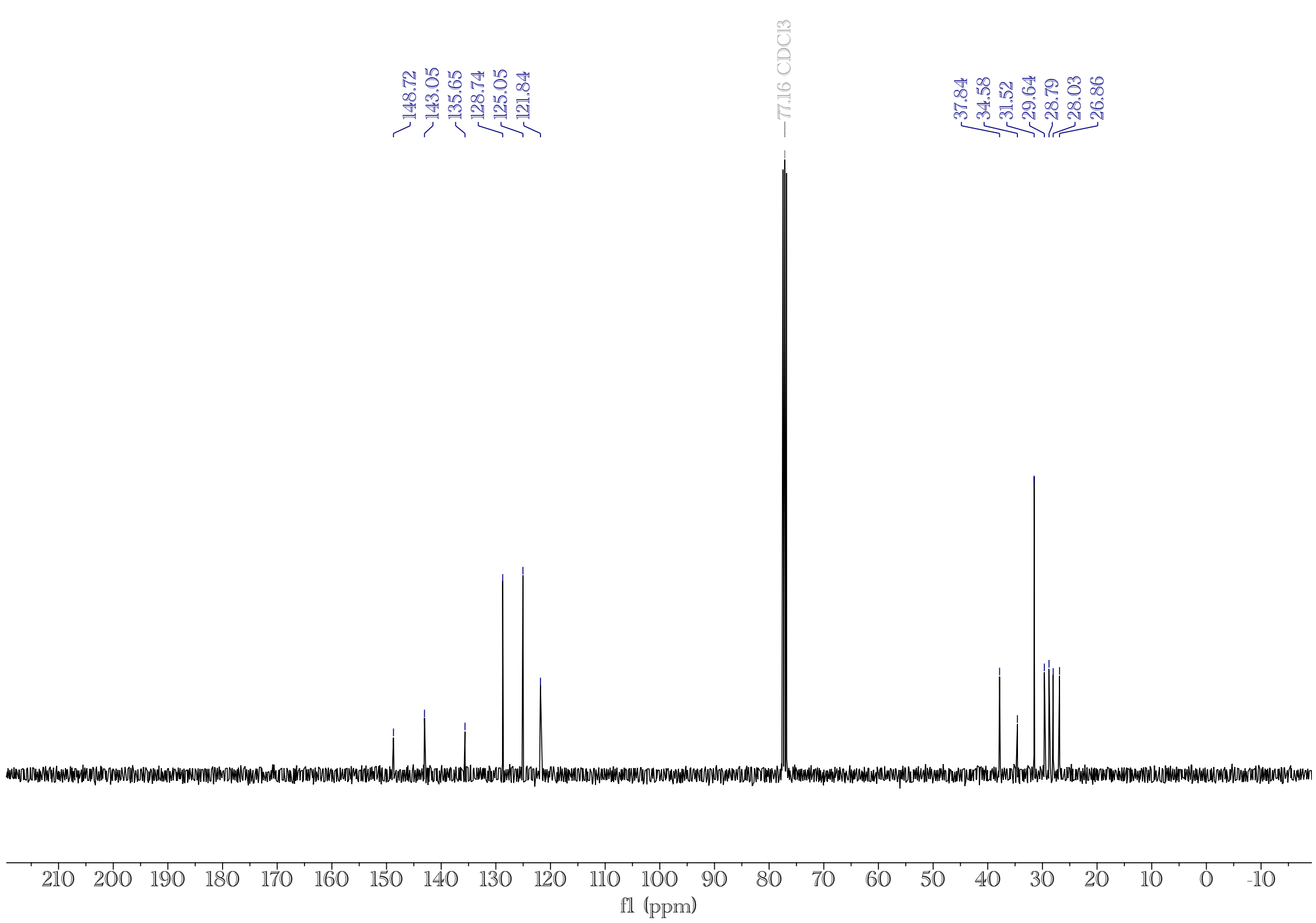

^{1}H NMR of 1-(cyclohexylidenemethyl)-4-methylbenzene (P8)

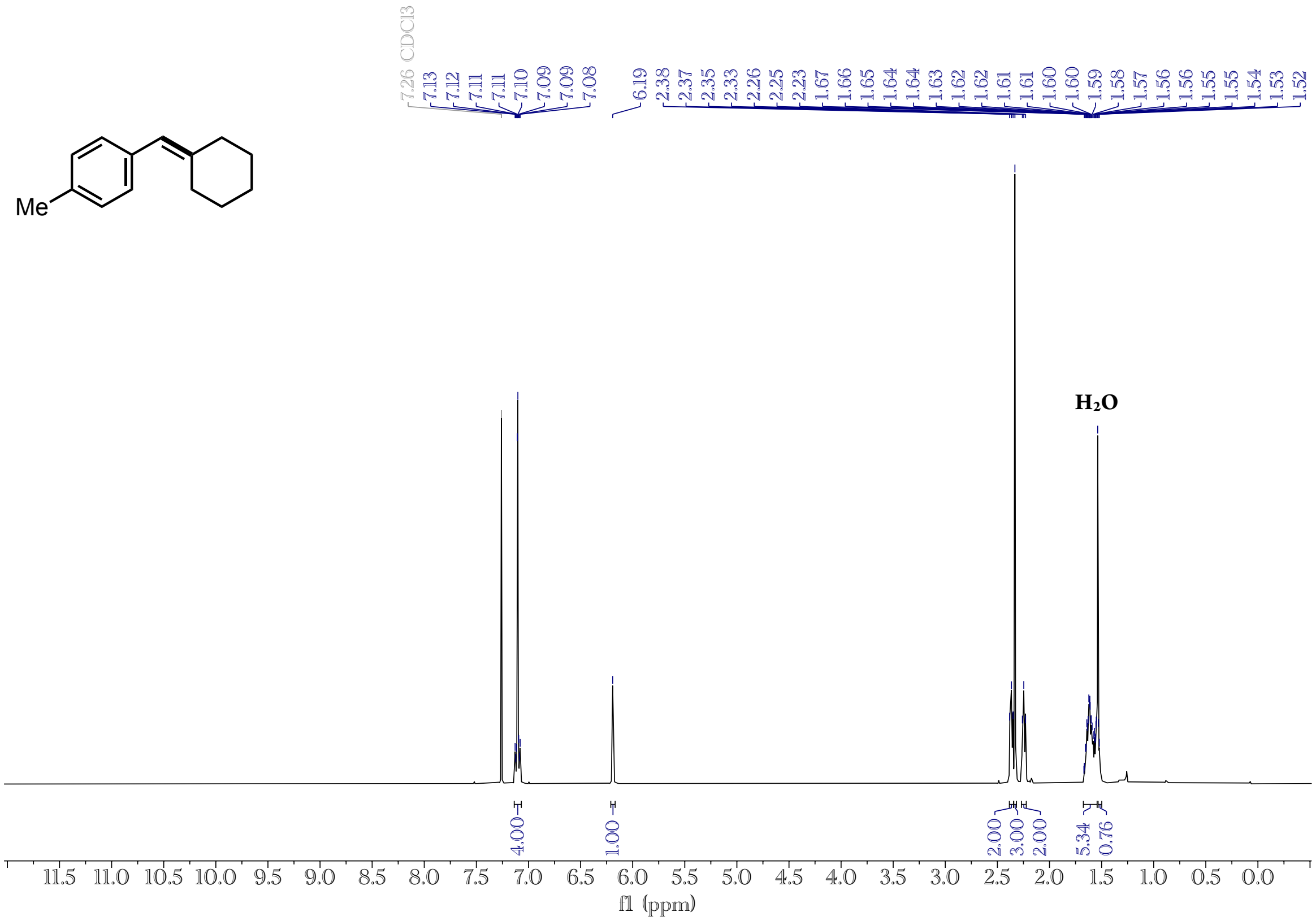

$^{13}C\{^{1}H\}$ NMR of 1-(cyclohexylidenemethyl)-4-methylbenzene (P8)

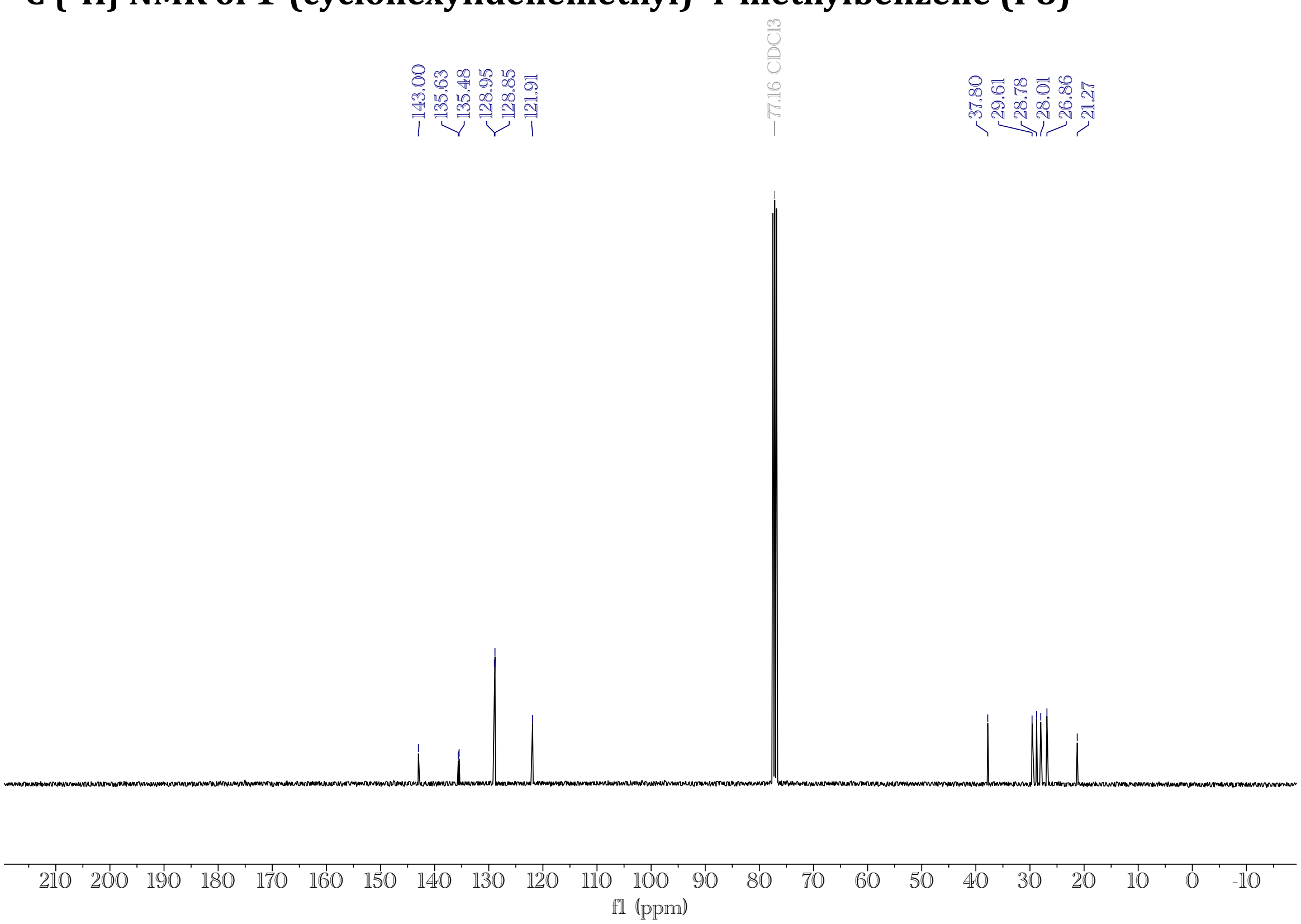

¹H NMR of 1-bromo-2-(cyclohexylidenemethyl)benzene (P9)

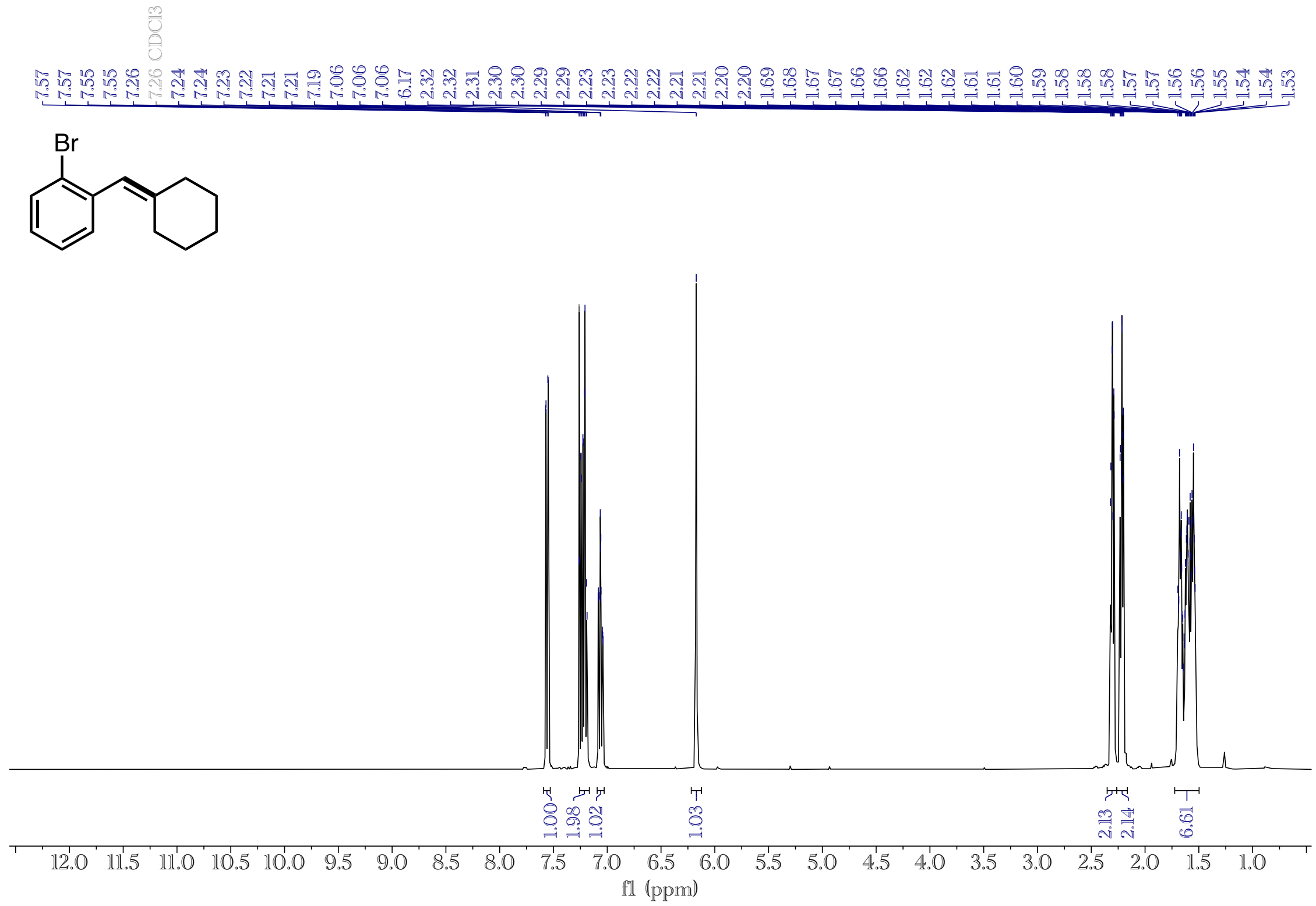

¹³C {¹H} NMR of 1-bromo-2-(cyclohexylidenemethyl)benzene (P9)

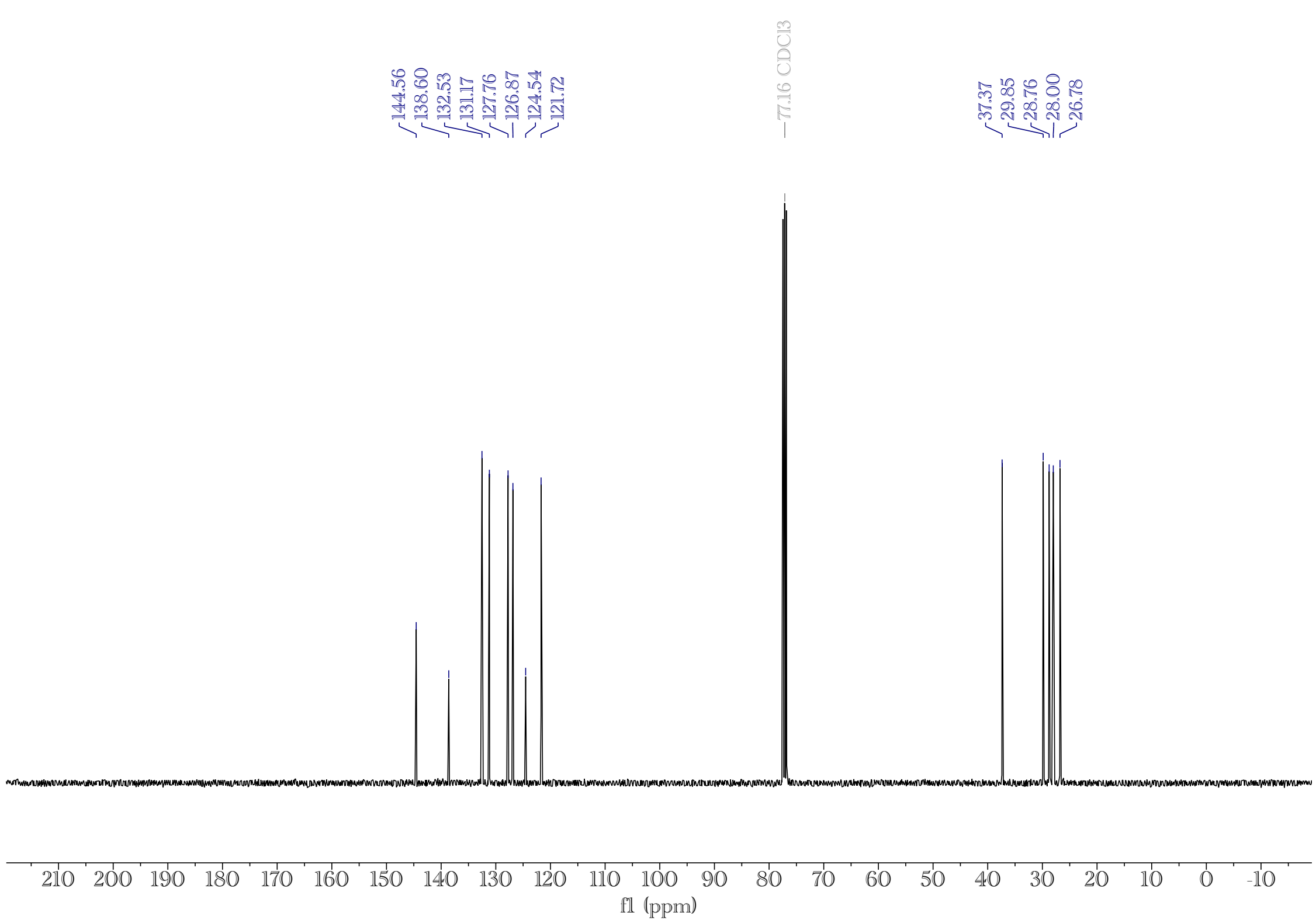

^{1}H NMR of 1-(cyclohexylidenemethyl)-2-methylbenzene (P10)

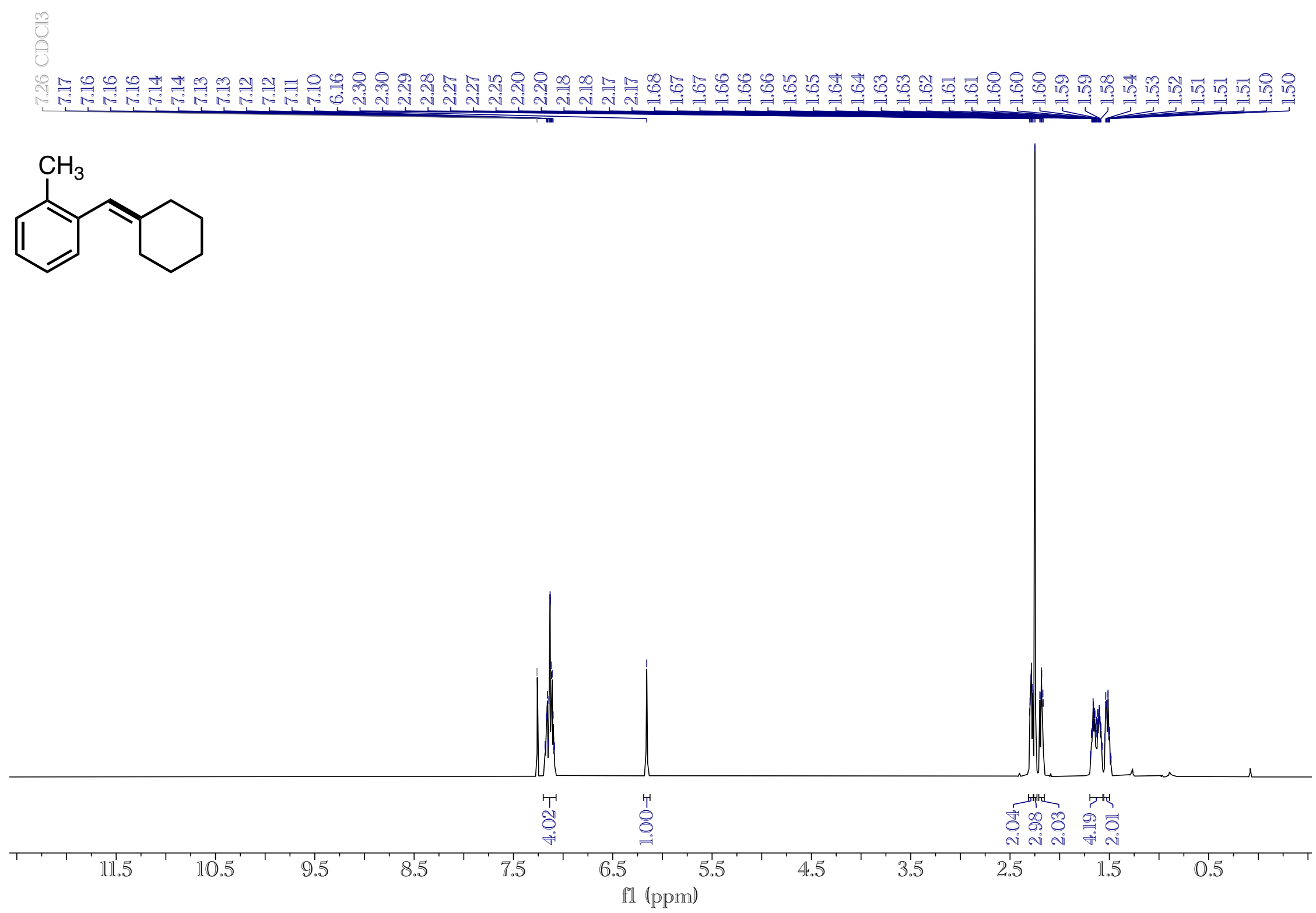

^{13}C {^{1}H} NMR of 1-(cyclohexylidenemethyl)-2-methylbenzene (P10)

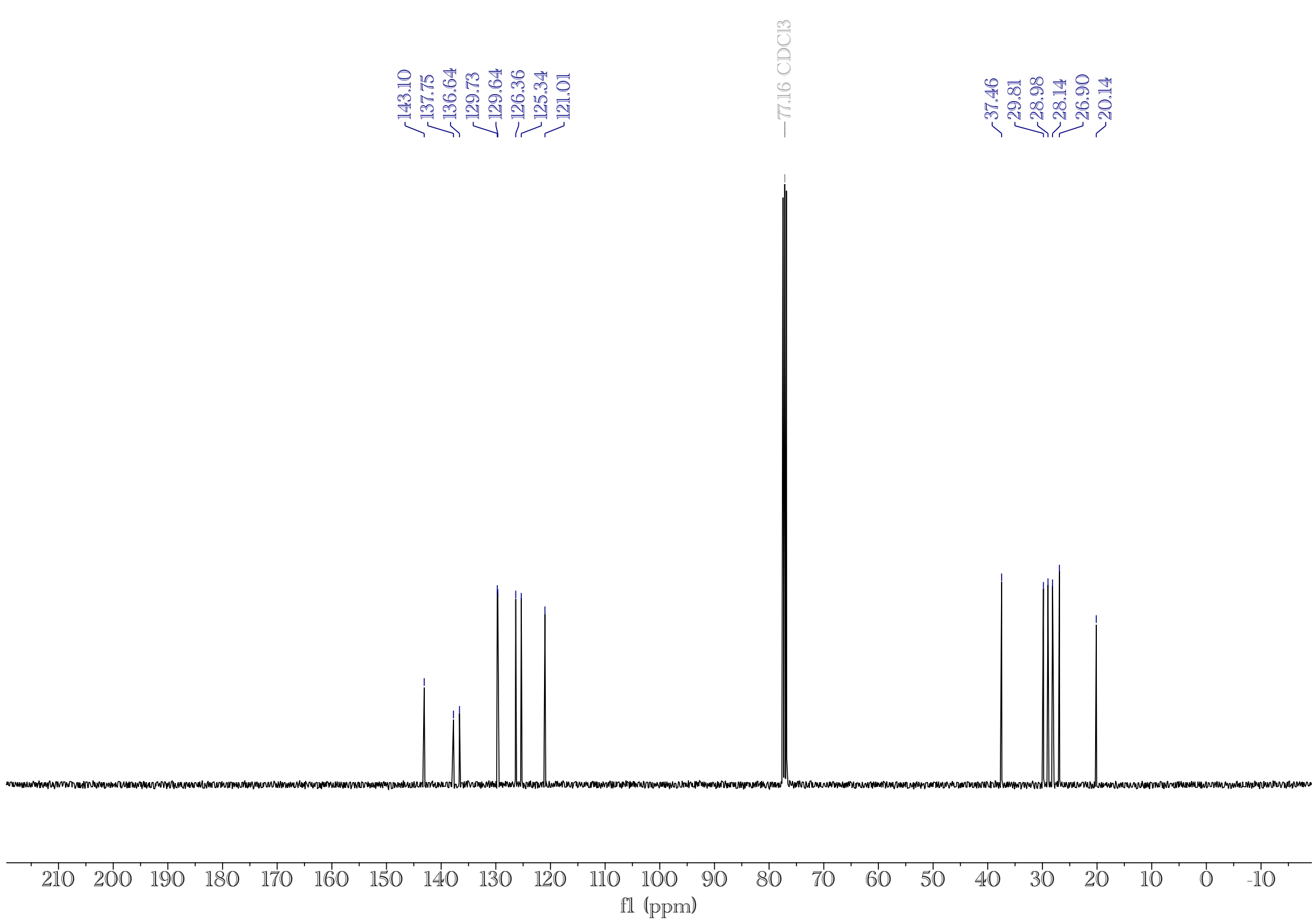

¹H NMR of 3-(cyclohexylidenemethyl)bicyclo[4.2.0]octa-1(6),2,4-triene (P12)

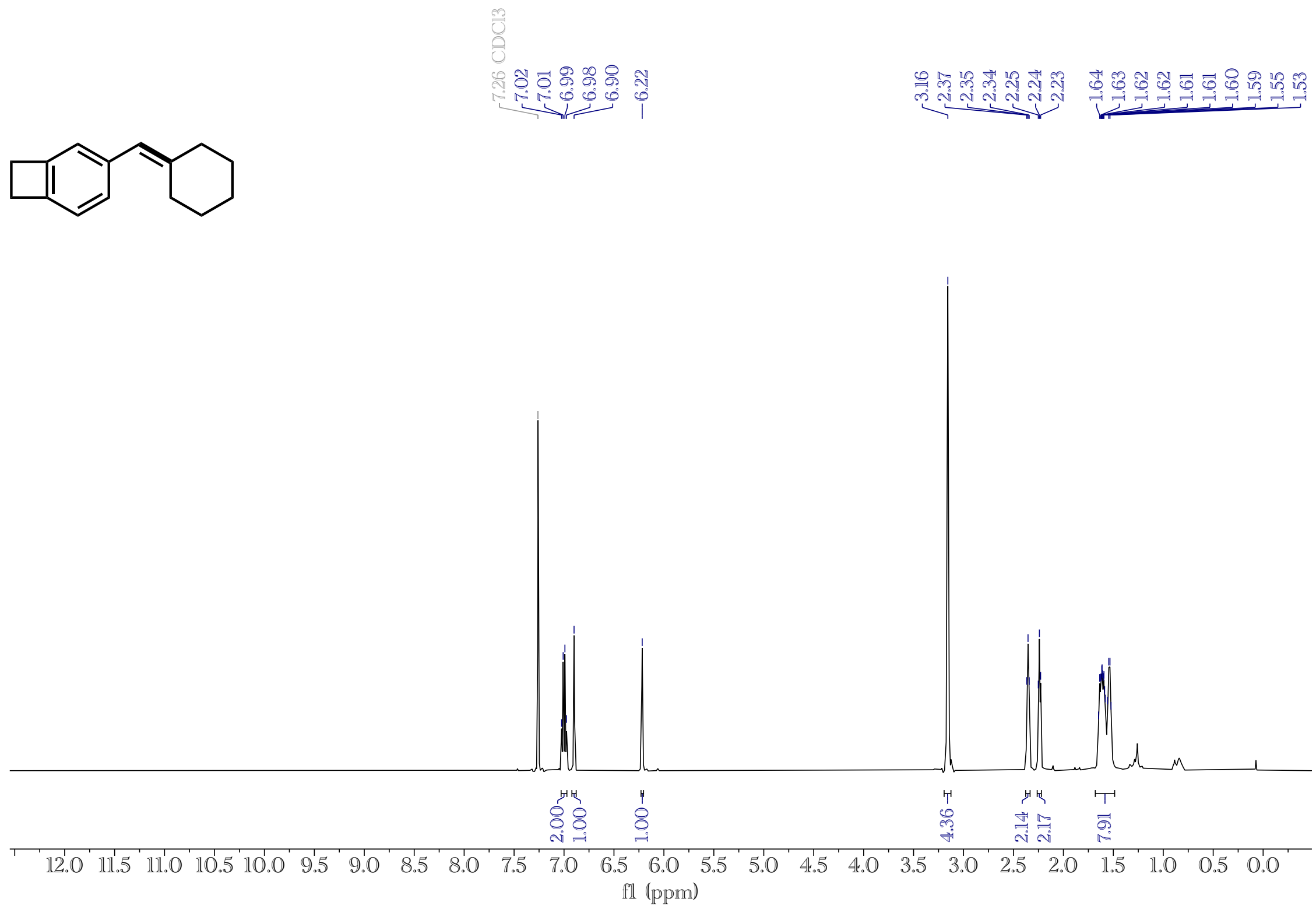

¹³C {¹H} NMR of 3-(cyclohexylidenemethyl)bicyclo[4.2.0]octa-1(6),2,4-triene (P12)

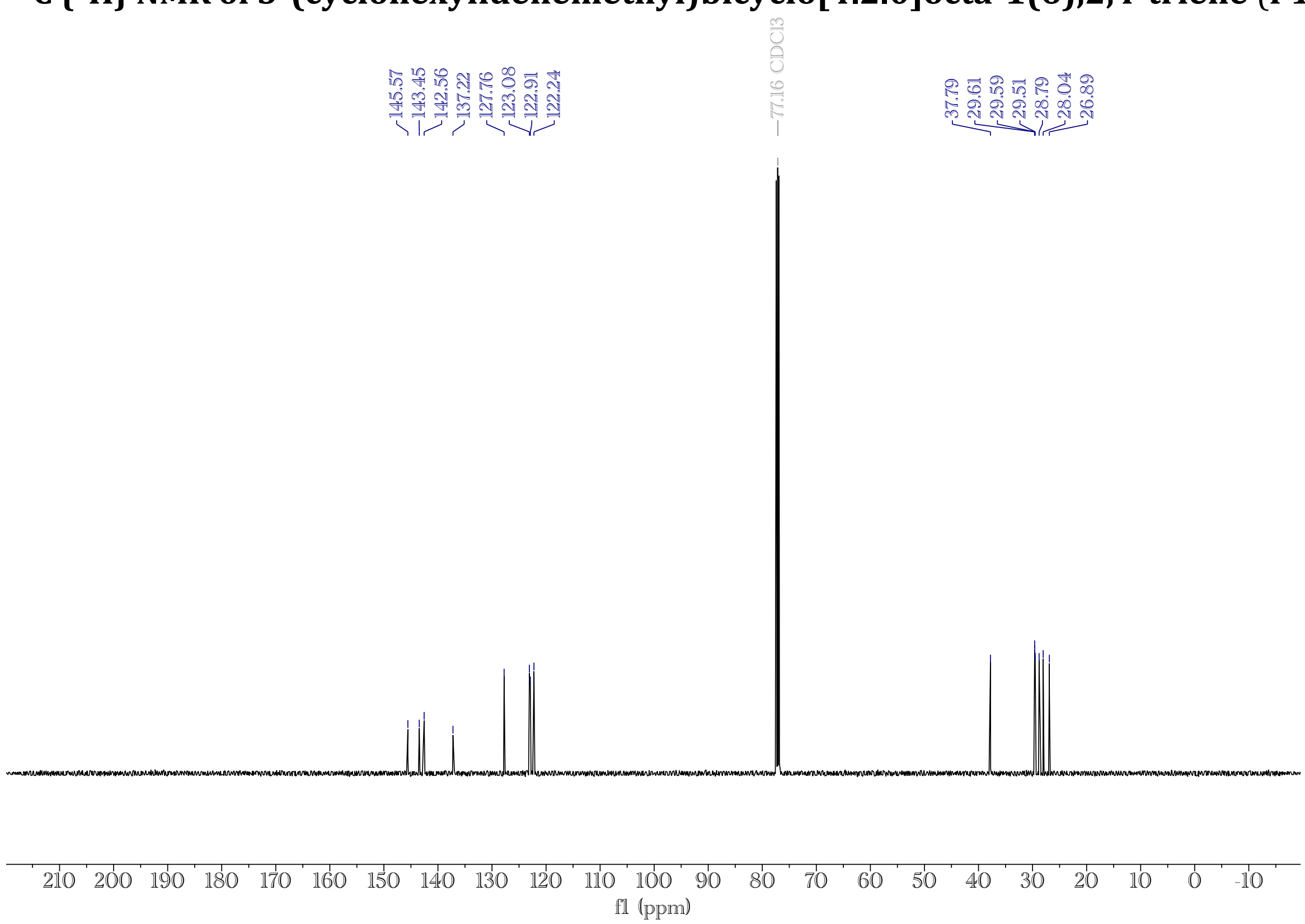

¹H NMR of 1-(4-(cyclohexylidenemethyl)phenyl)propan-2-one (P13)

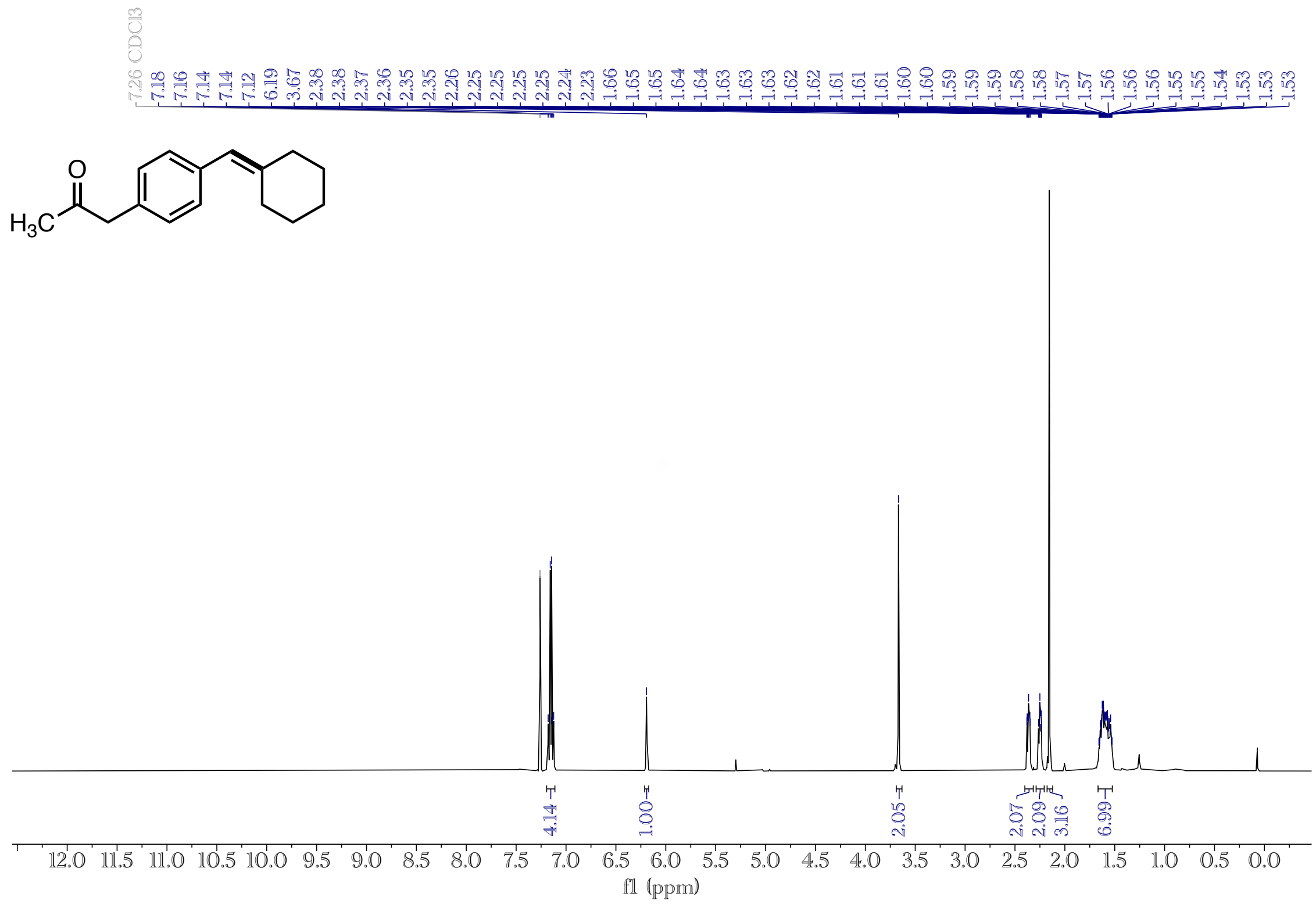

¹³C {¹H} NMR of 1-(4-(cyclohexylidenemethyl)phenyl)propan-2-one (P13)

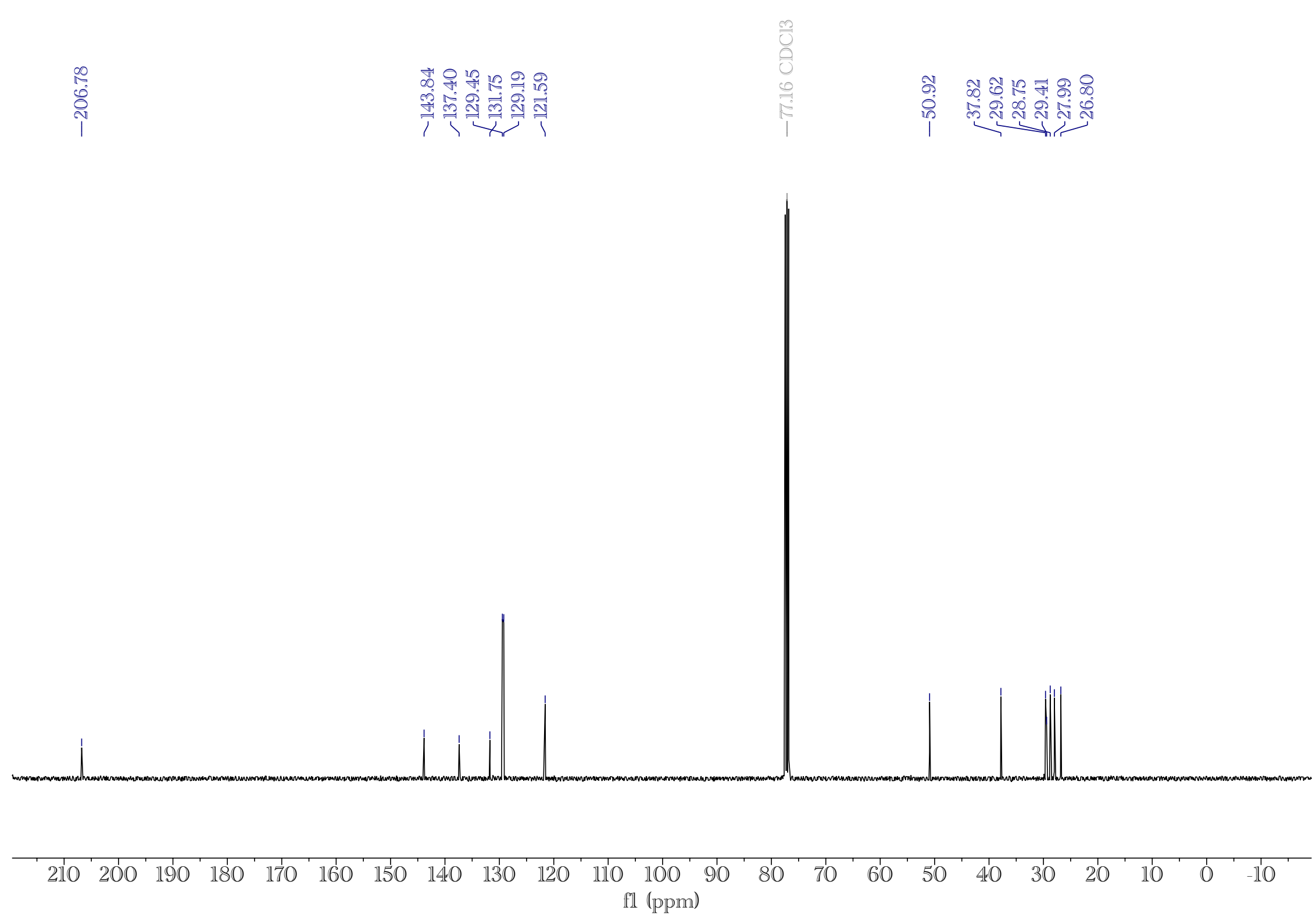

^{1}H NMR of 4-(cyclohexylidenemethyl)benzaldehyde (P14)

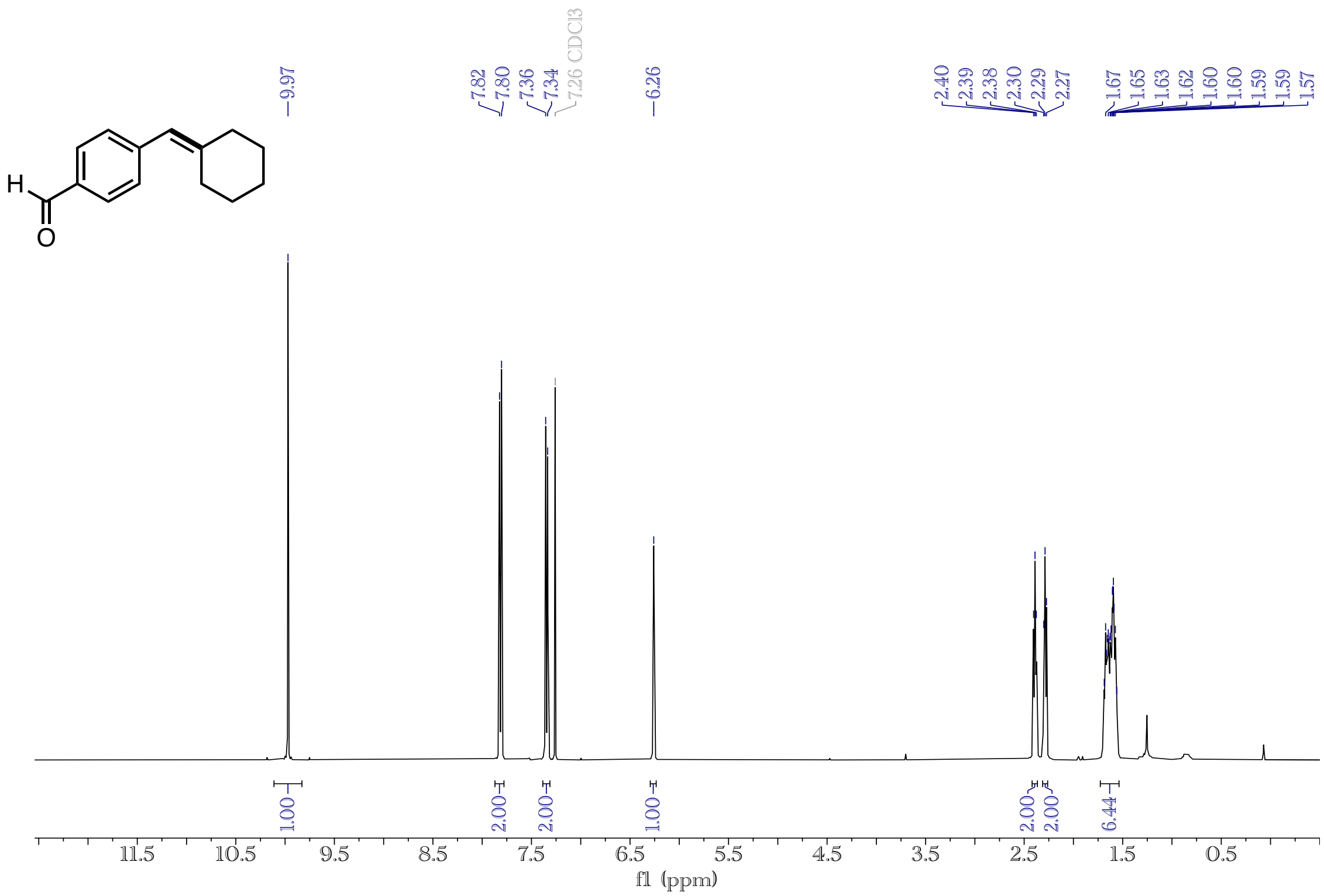

^{13}C {^{1}H} NMR of 4-(cyclohexylidenemethyl)benzaldehyde (P14)

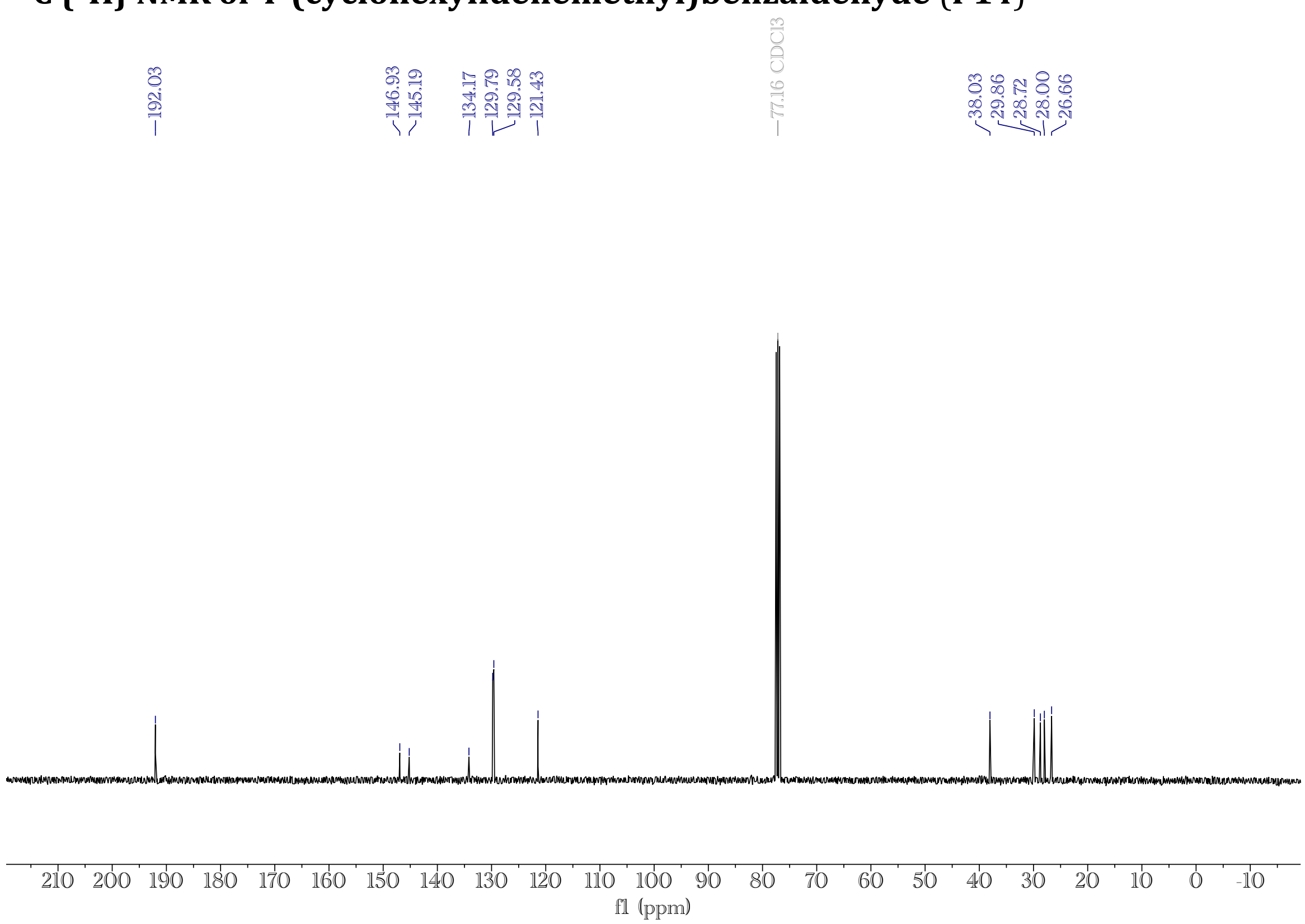

¹H NMR of 2-(cyclohexylidenemethyl)naphthalene (P15)

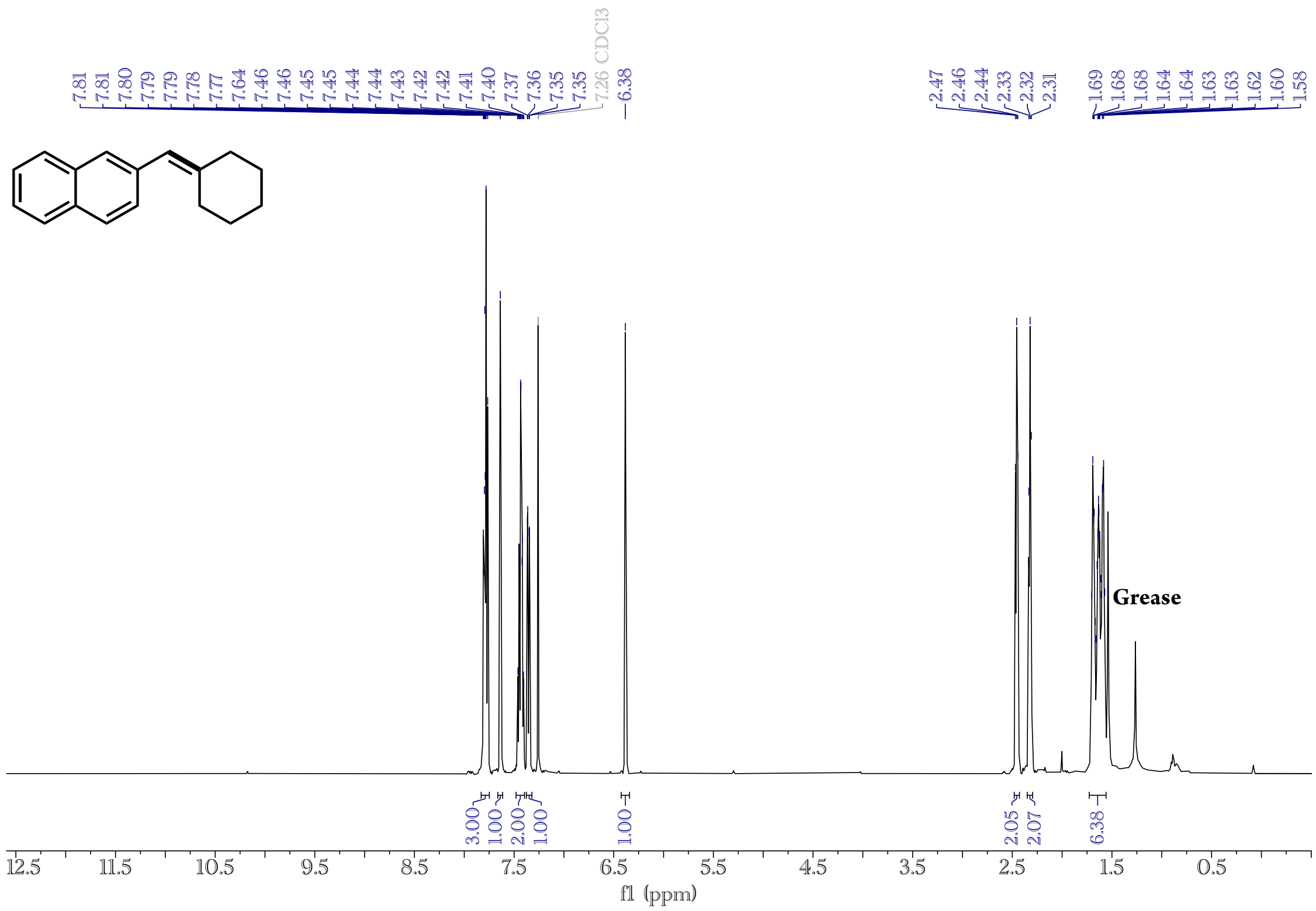

¹³C {¹H} NMR of 2-(cyclohexylidenemethyl)naphthalene (P15)

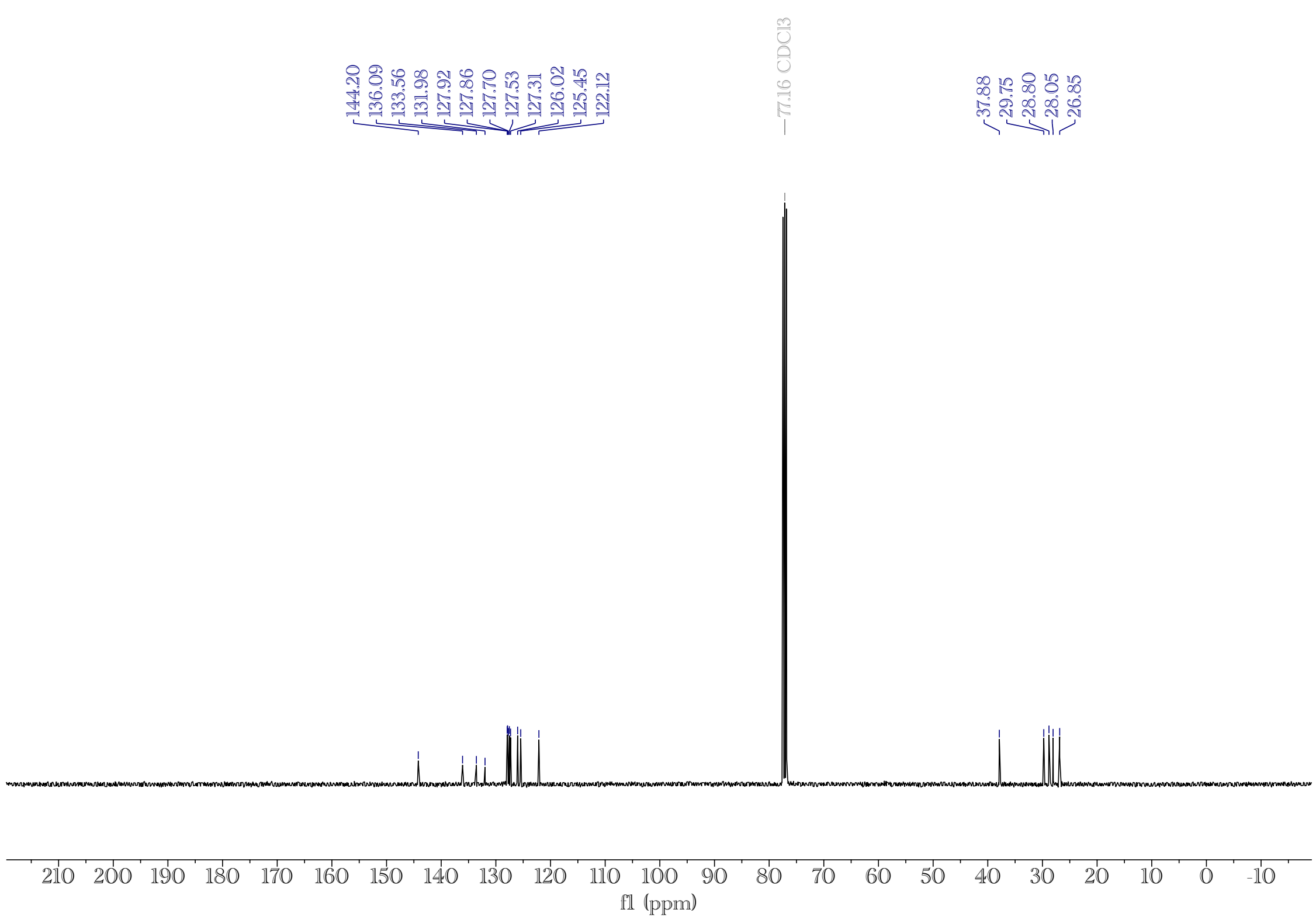

^{1}H NMR of 5-(cyclohexylidenemethyl)-2,3-dihydrobenzofuran (P16)

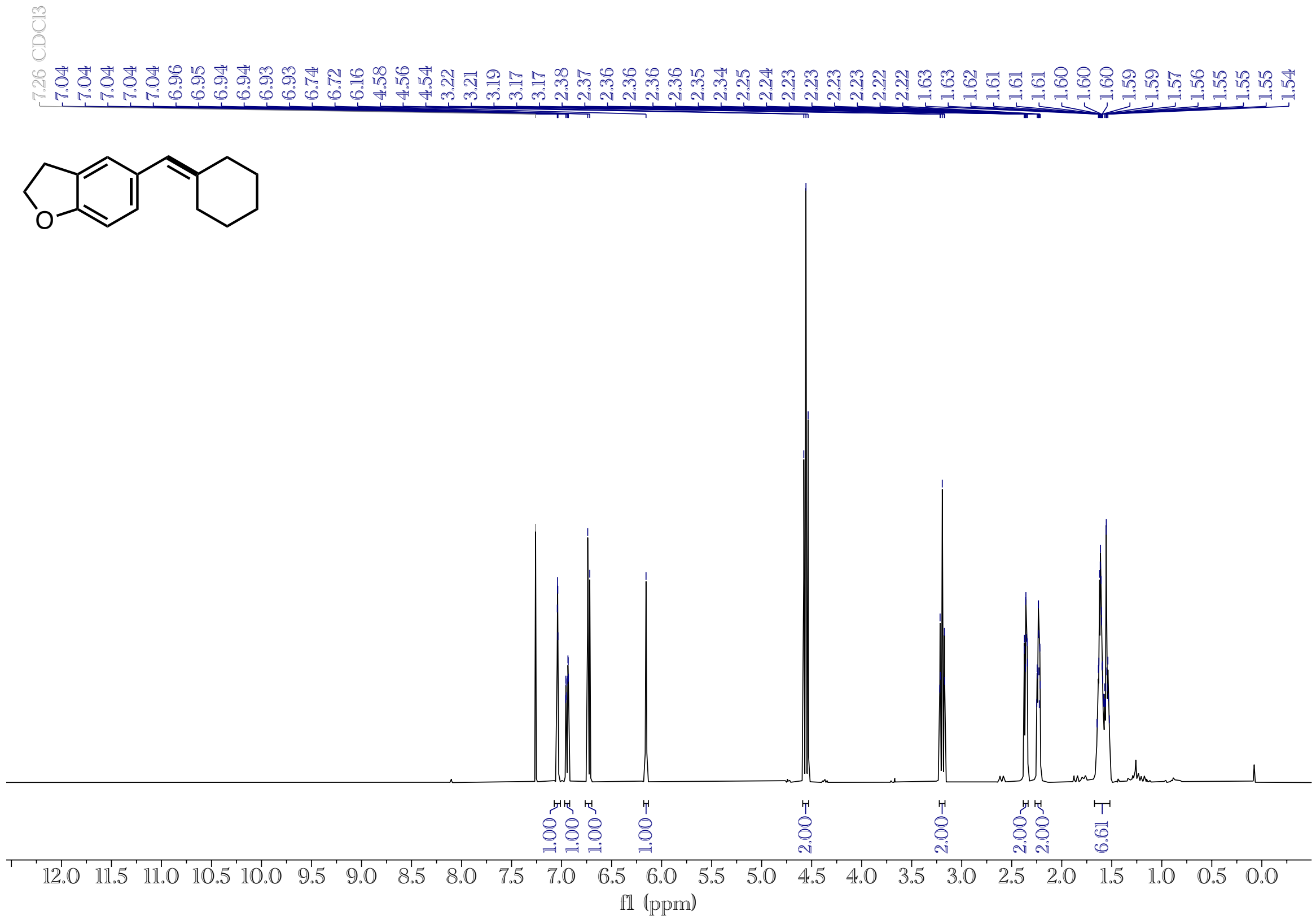

^{13}C {^{1}H} NMR of 5-(cyclohexylidenemethyl)-2,3-dihydrobenzofuran (P16)

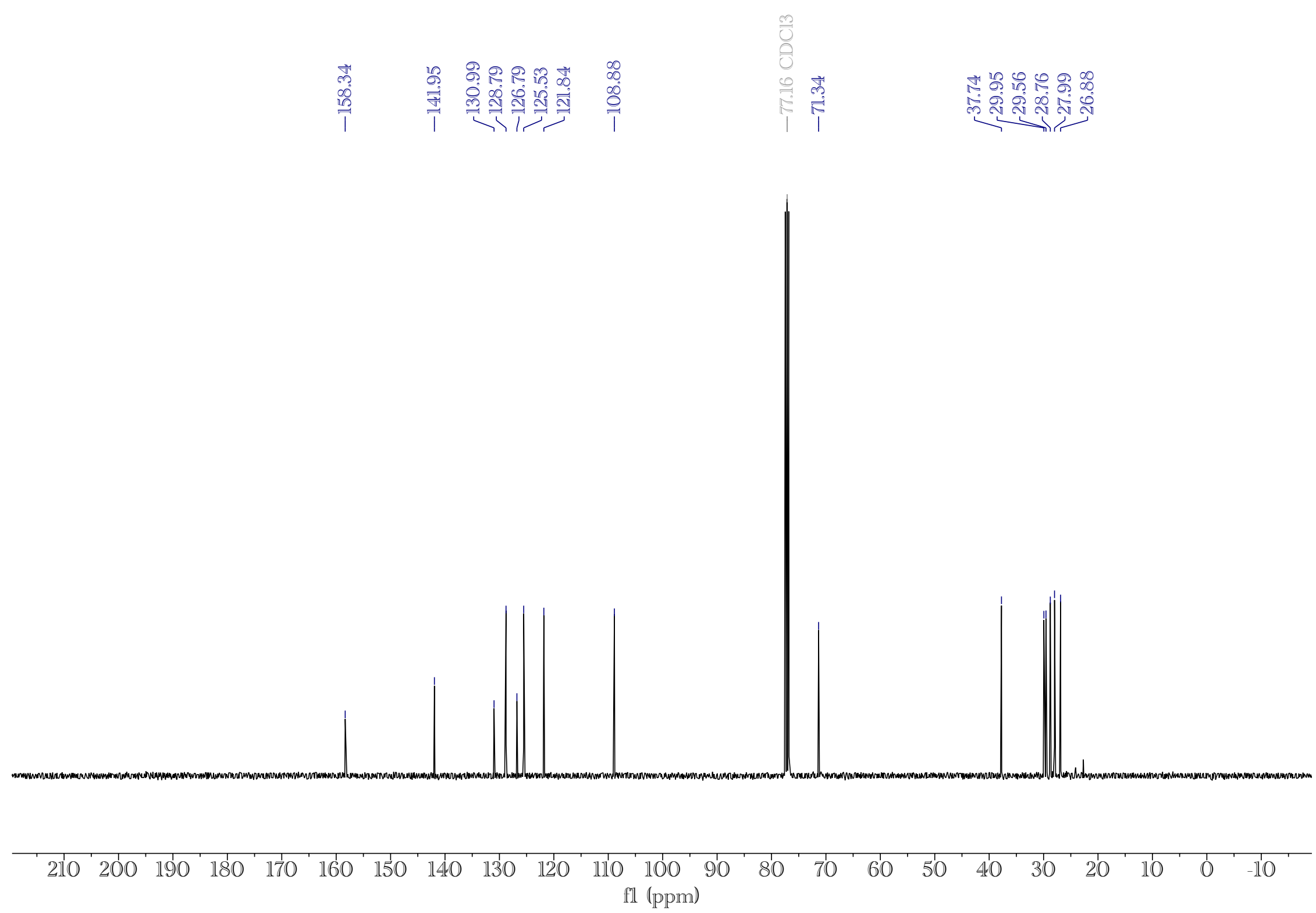

^{1}H NMR of 2-(cyclohexylidenemethyl)pyridine (P17)

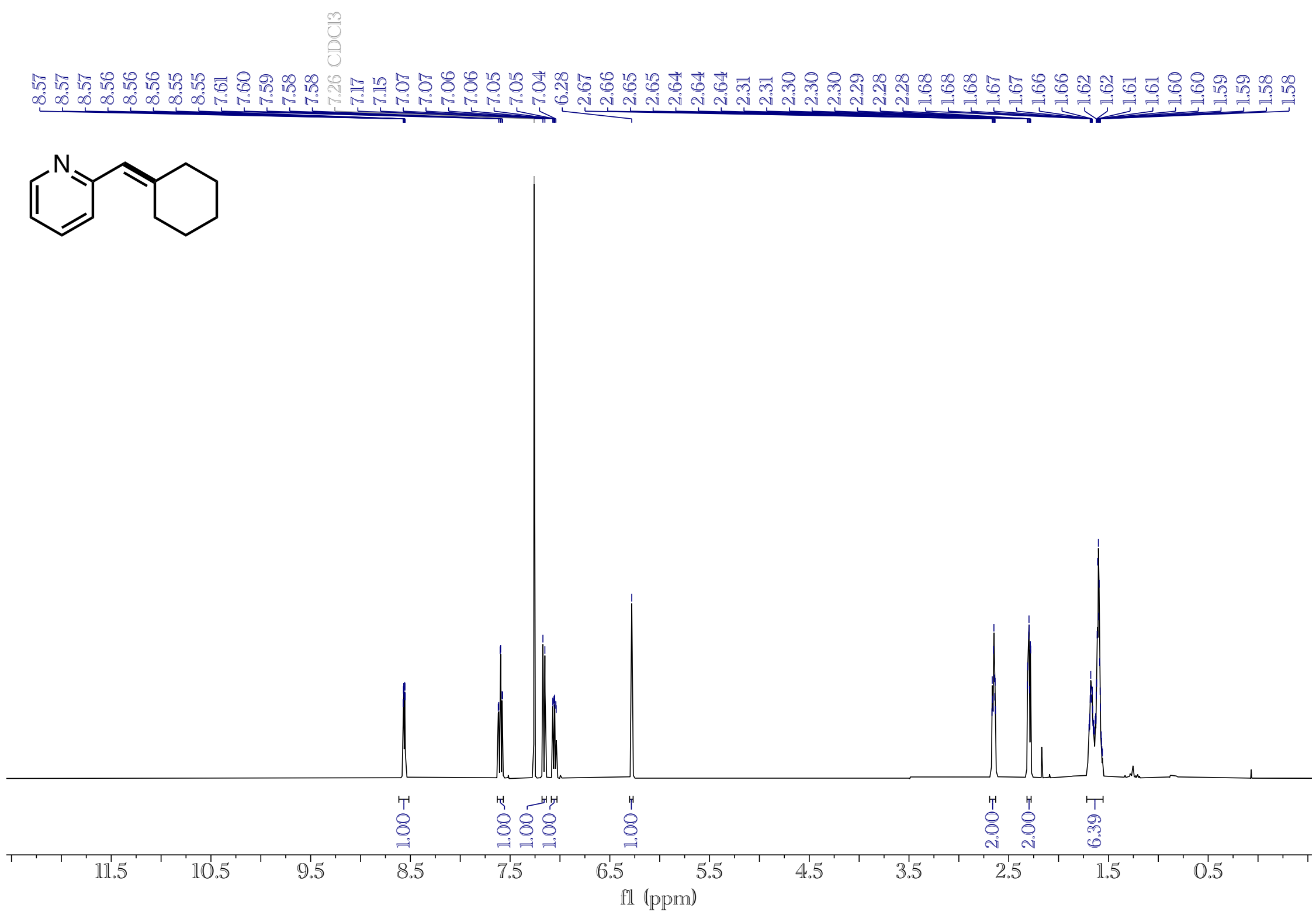

^{13}C {^{1}H} NMR of 2-(cyclohexylidenemethyl)pyridine (P17)

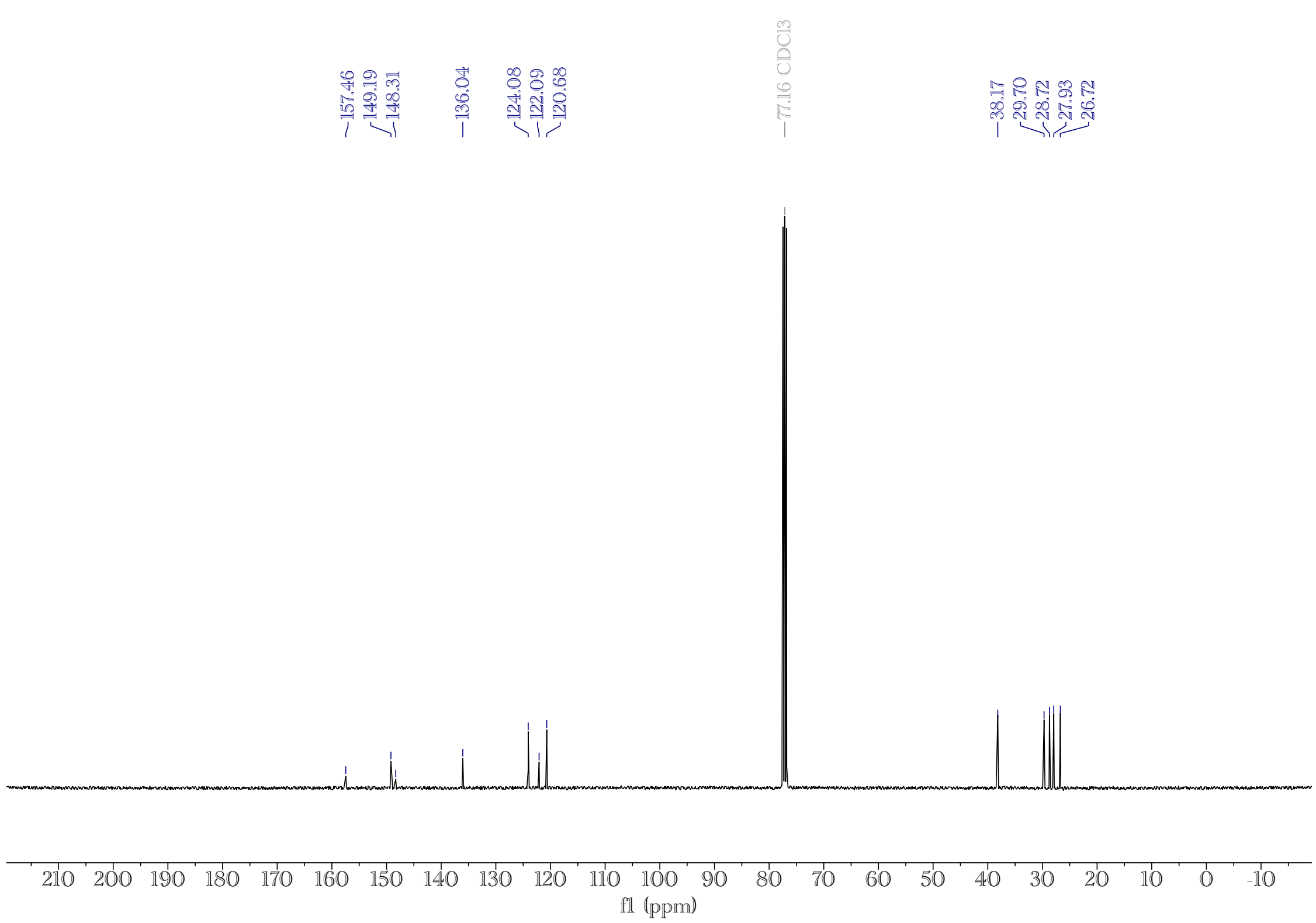

^{1}H NMR of 3-(cyclohexylidenemethyl)pyridine (P18)

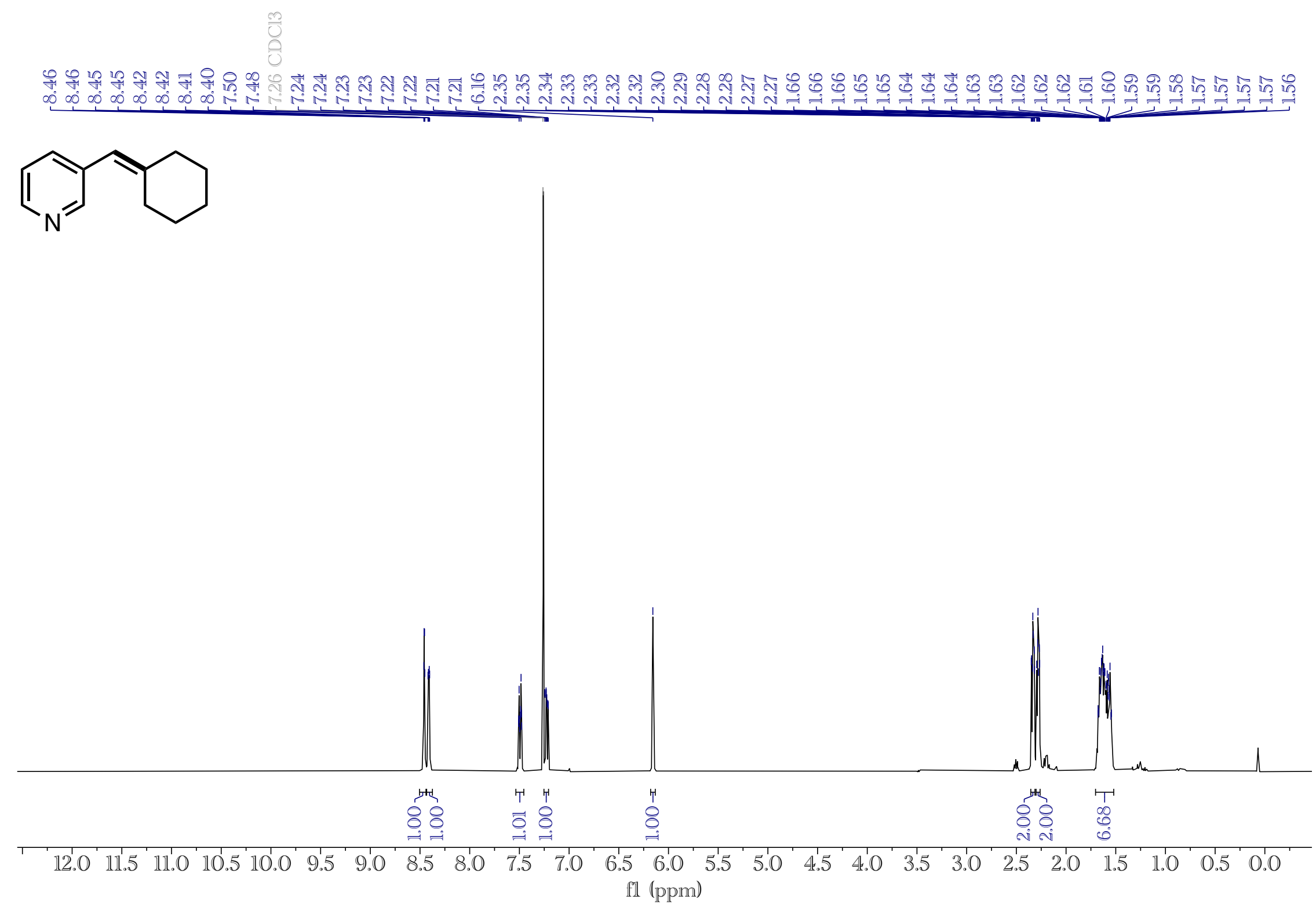

^{13}C {^{1}H} NMR of 3-(cyclohexylidenemethyl)pyridine (P18)

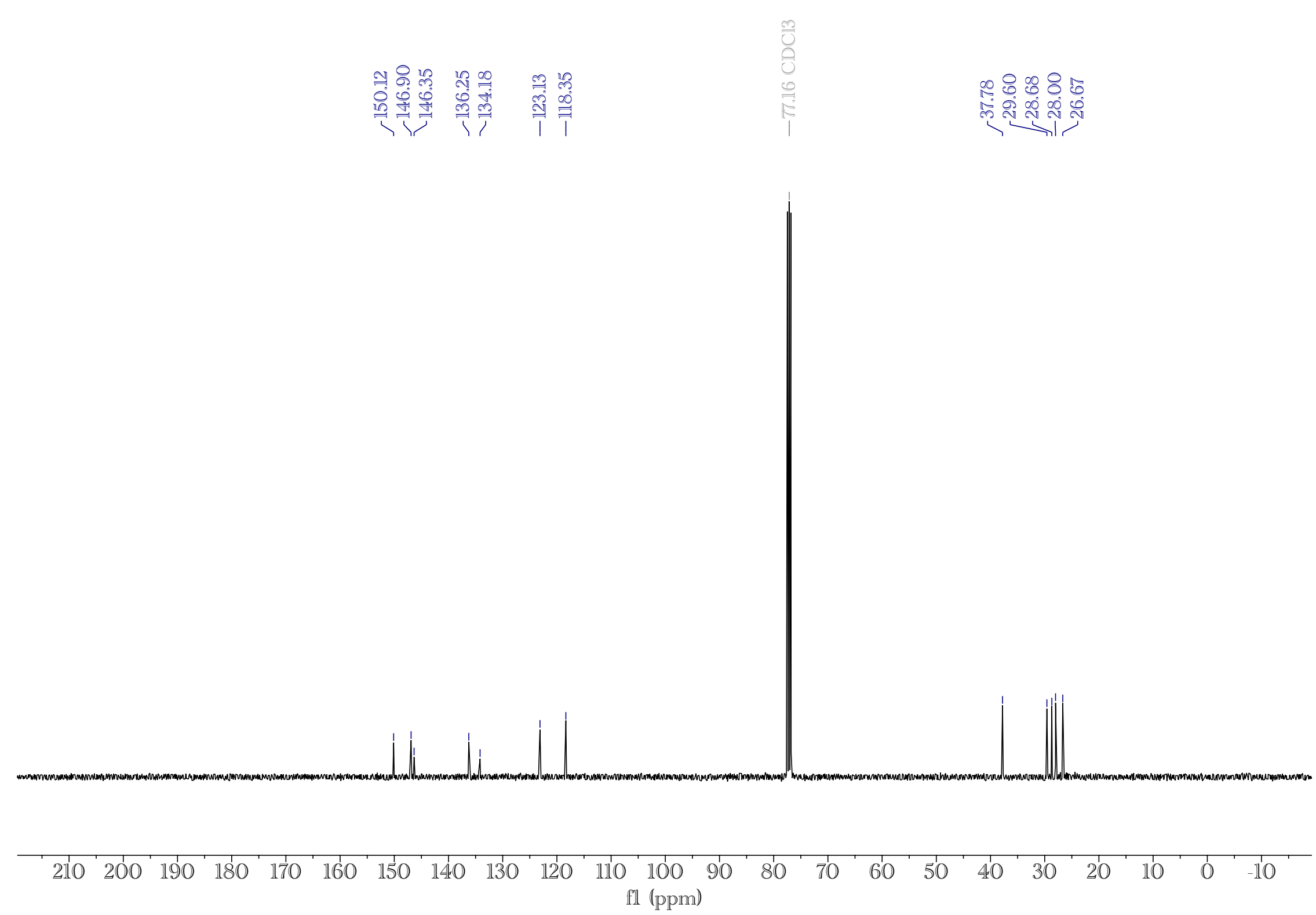

^{1}H NMR of 1,4-bis(cyclohexylidenemethyl)benzene (P19)

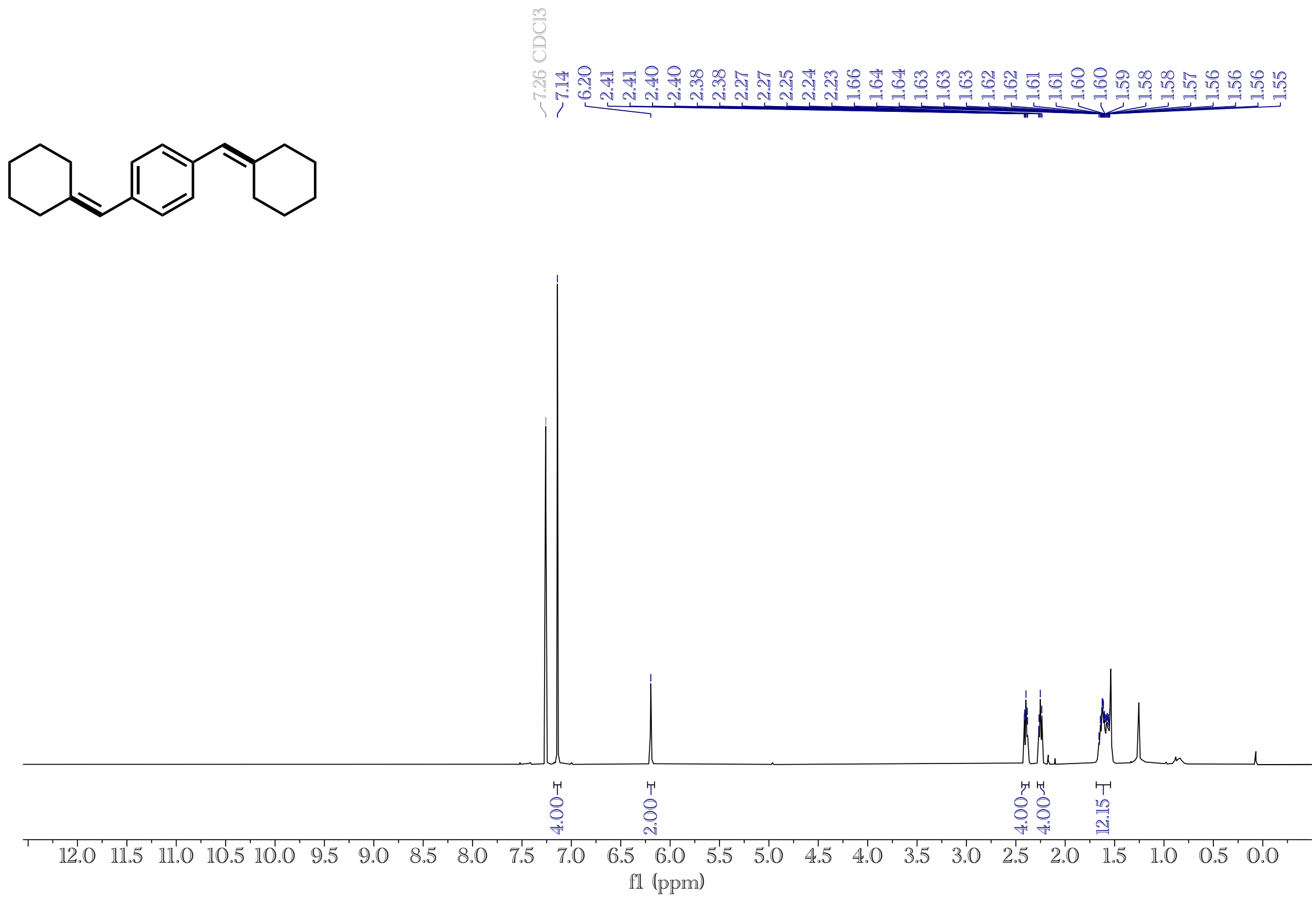

^{13}C {^{1}H} NMR of 1,4-bis(cyclohexylidenemethyl)benzene (P19)

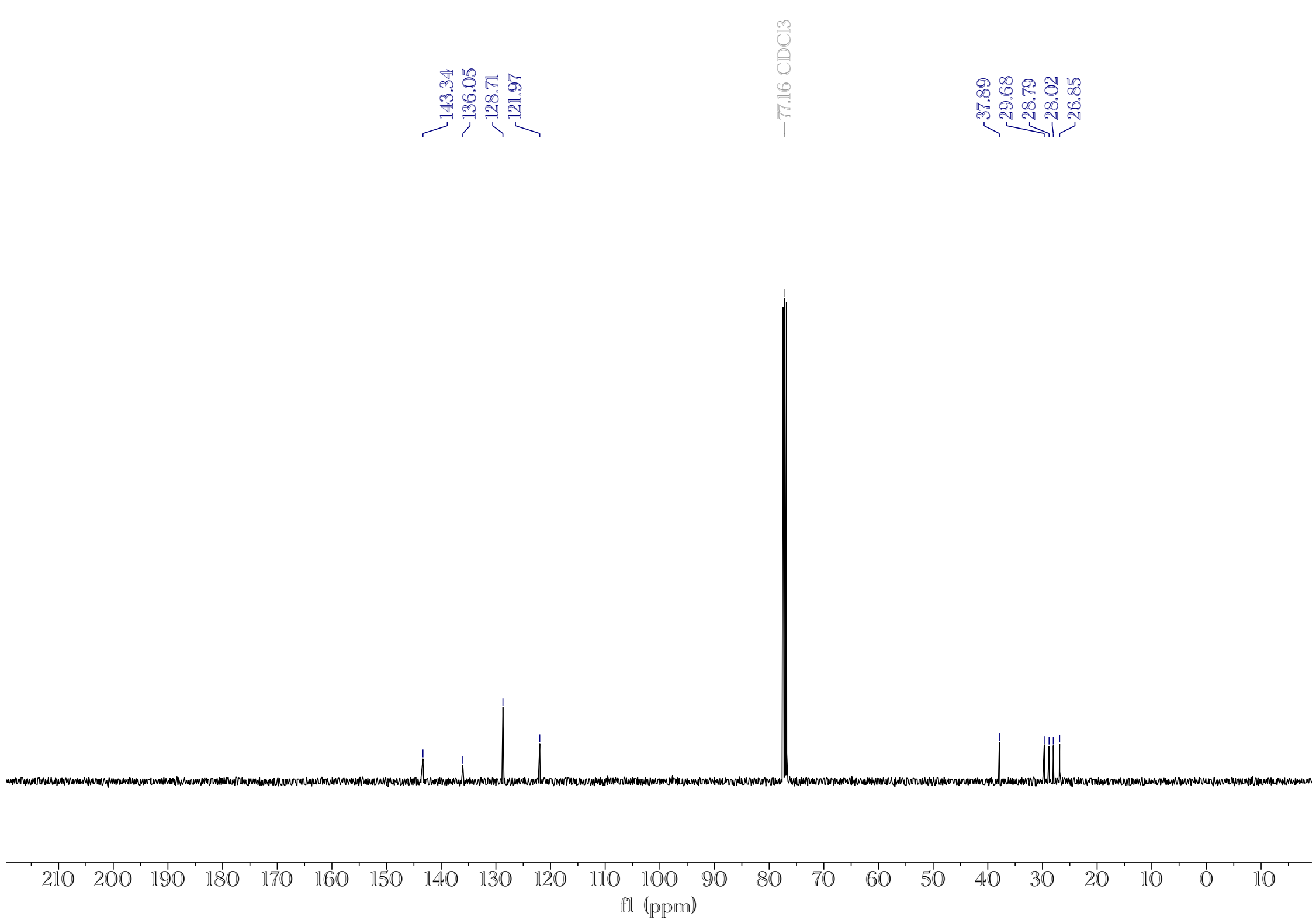

^{1}H NMR of 4-(4-chlorobenzylidene)tetrahydro-2*H*-pyran (P20)

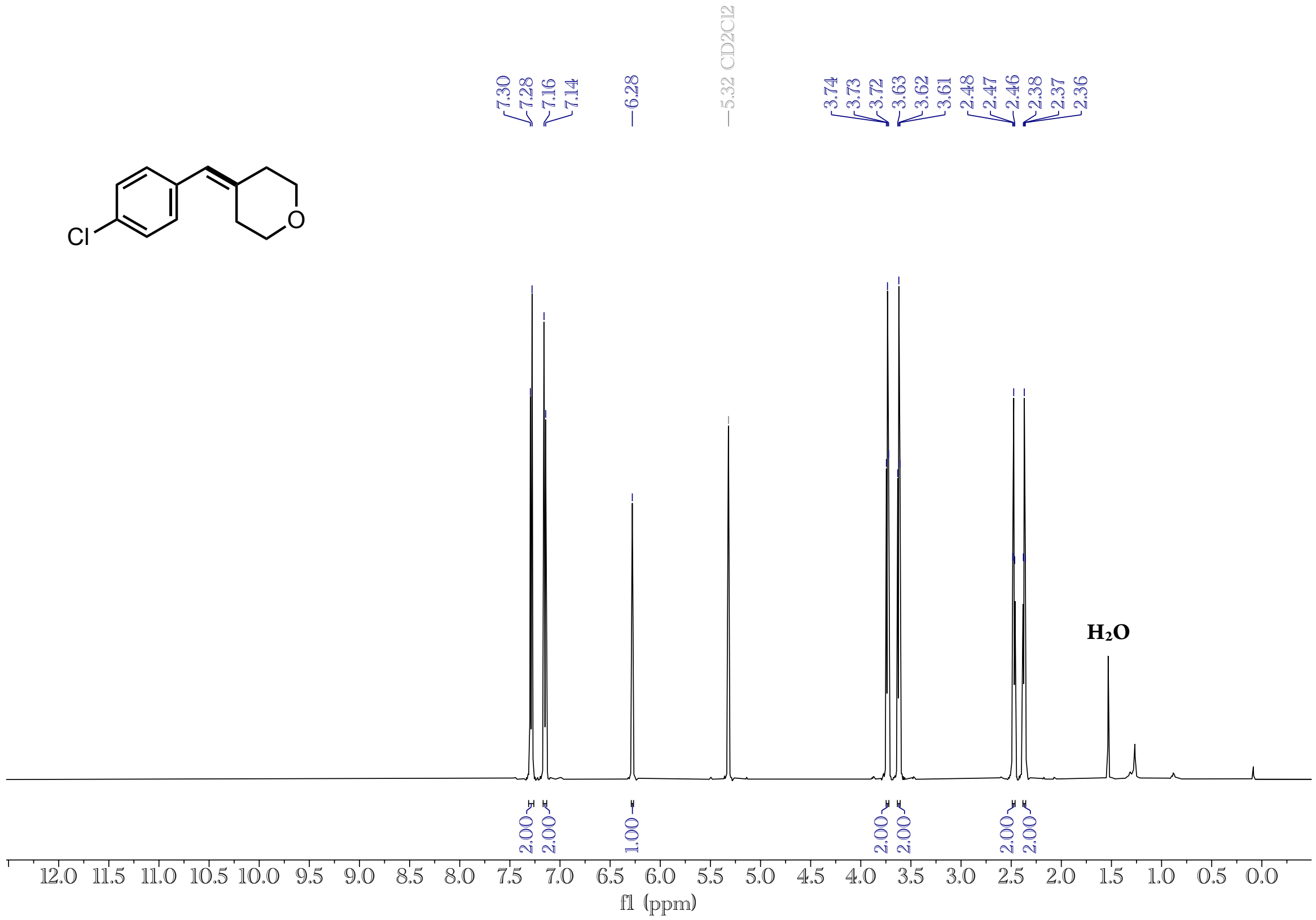

^{13}C NMR of 4-(4-chlorobenzylidene)tetrahydro-2*H*-pyran (P20)

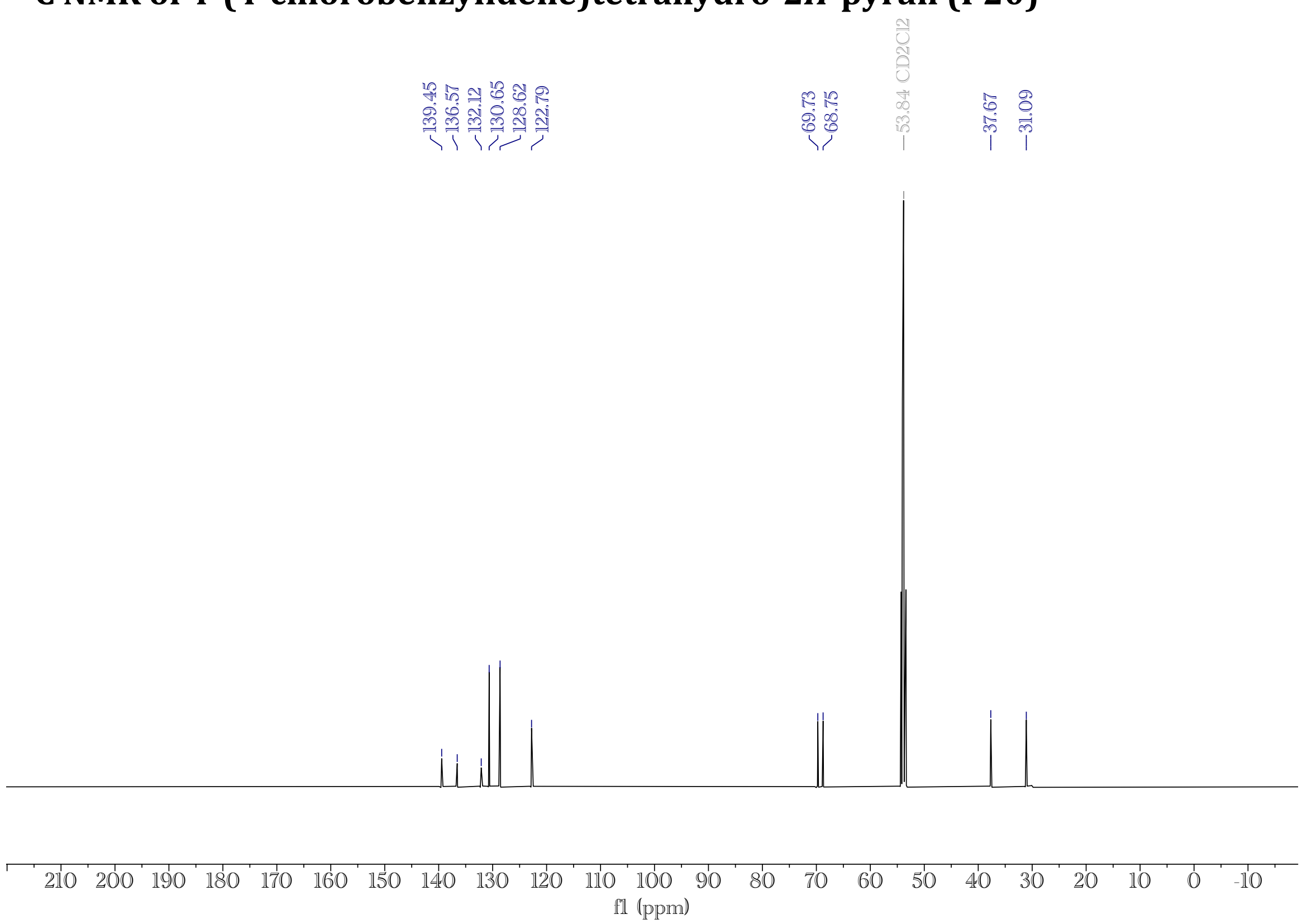

¹H NMR of *tert*-butyl 4-(4-chlorobenzylidene)piperidine-1-carboxylate (P21)

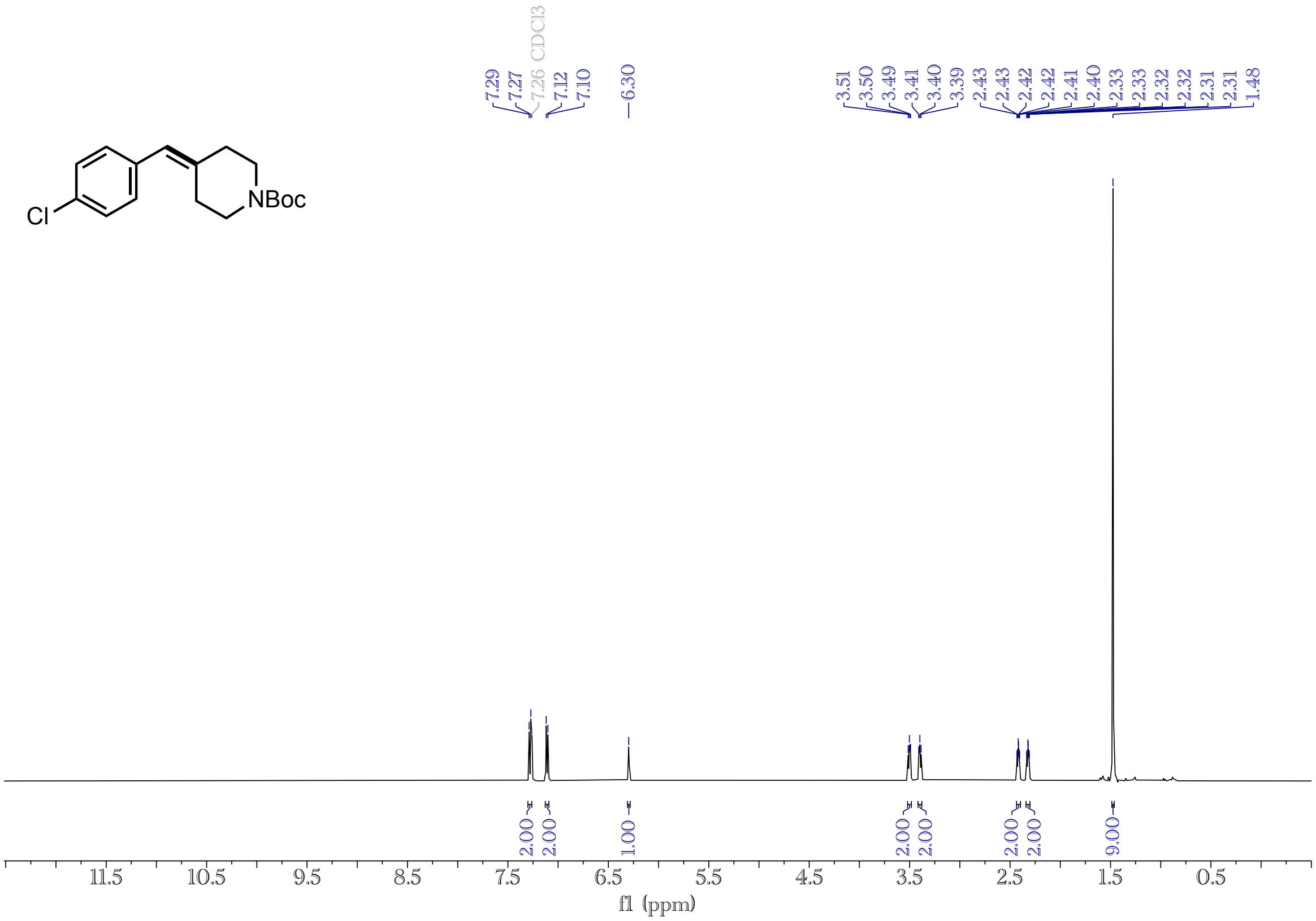

¹³C {¹H} NMR of *tert*-butyl 4-(4-chlorobenzylidene)piperidine-1-carboxylate (P21)

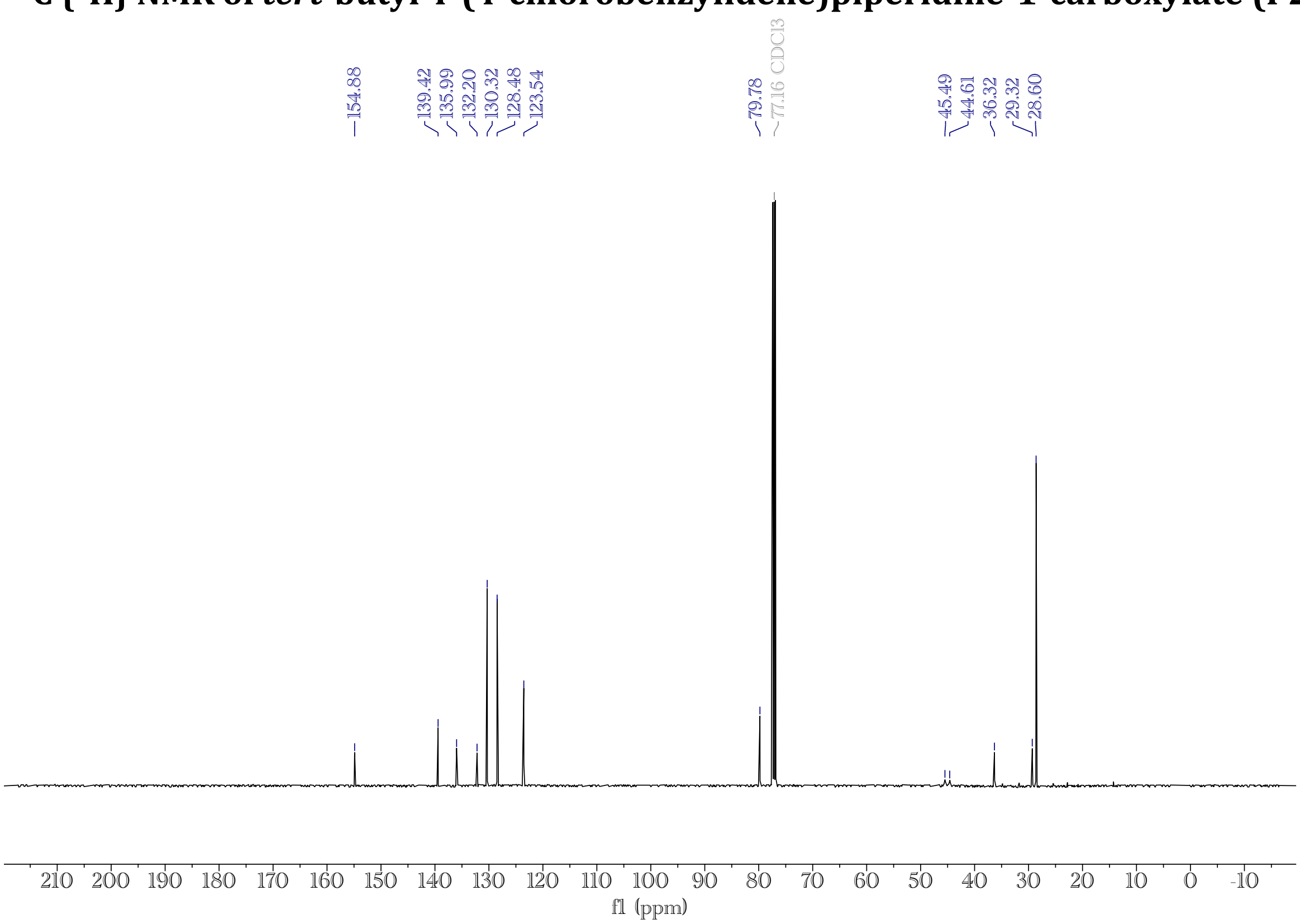

^{1}H NMR of 4-(4-chlorobenzylidene)-1-((3,5-dimethyl-1H-pyrazol-4-yl)sulfonyl)piperidine (P22)

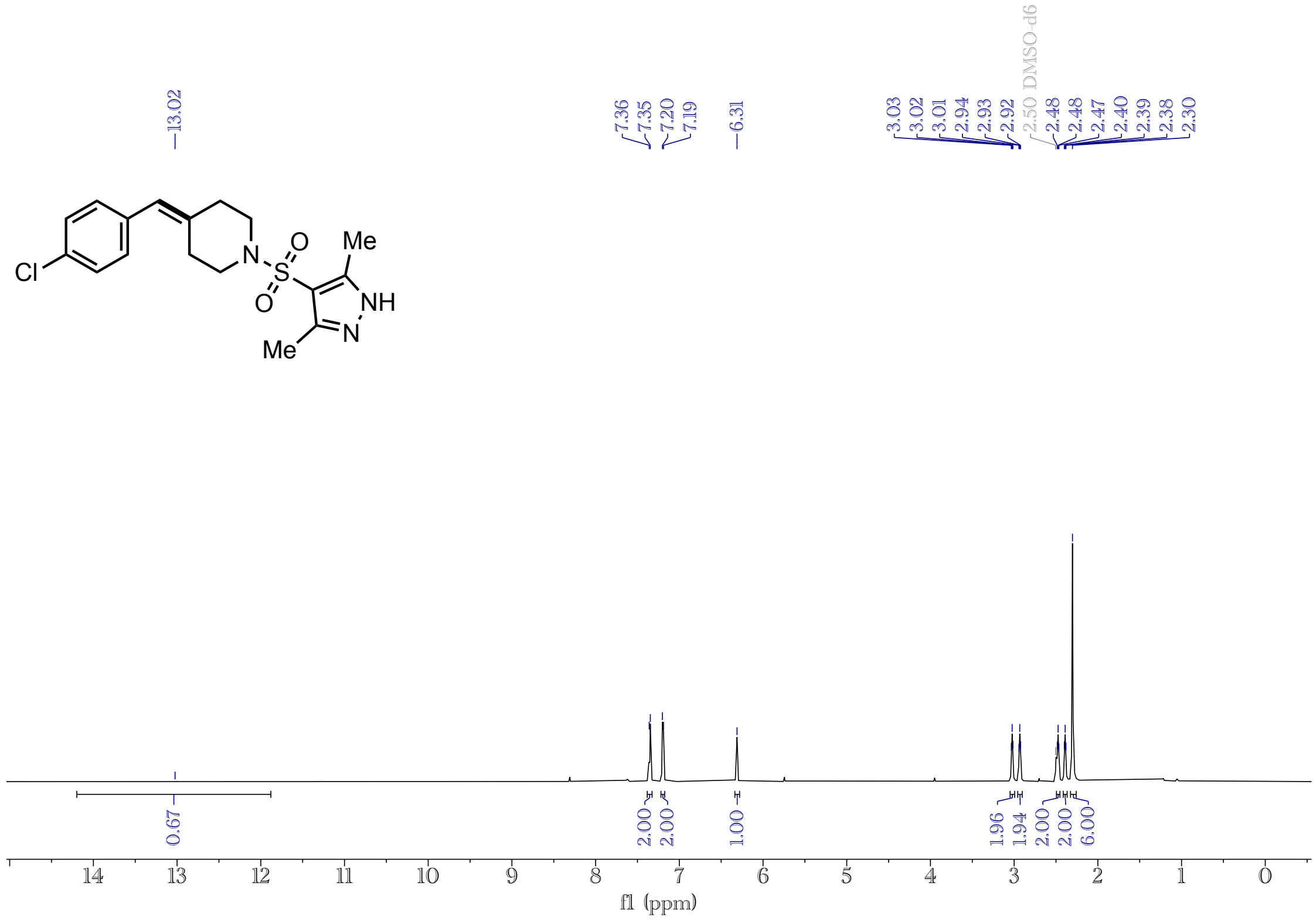

^{13}C {^{1}H} NMR of 4-(4-chlorobenzylidene)-1-((3,5-dimethyl-1H-pyrazol-4-yl)sulfonyl)piperidine (P22)

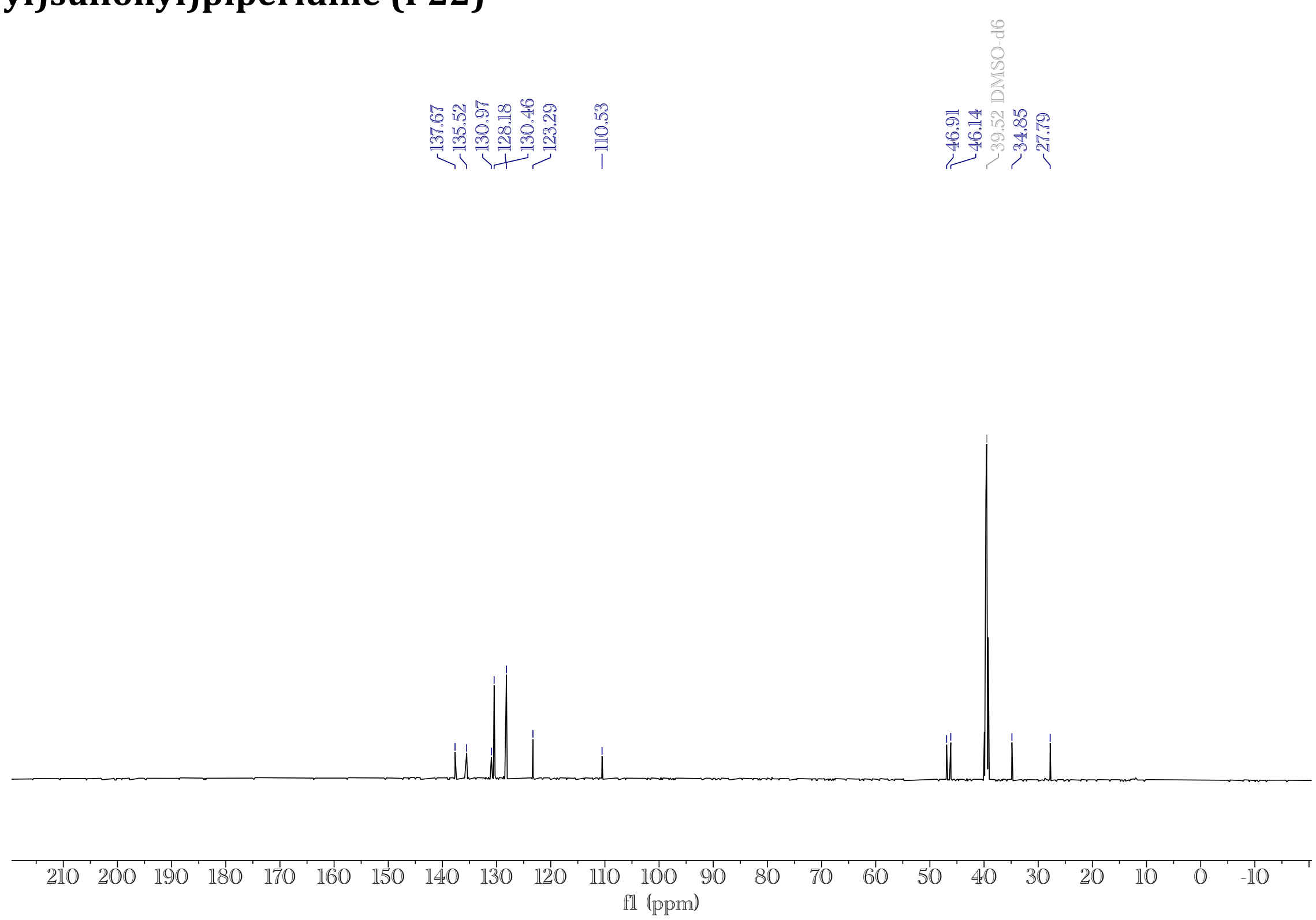

^{1}H NMR of *tert*-butyl 3-(4-chlorobenzylidene)azetidine-1-carboxylate (P23)

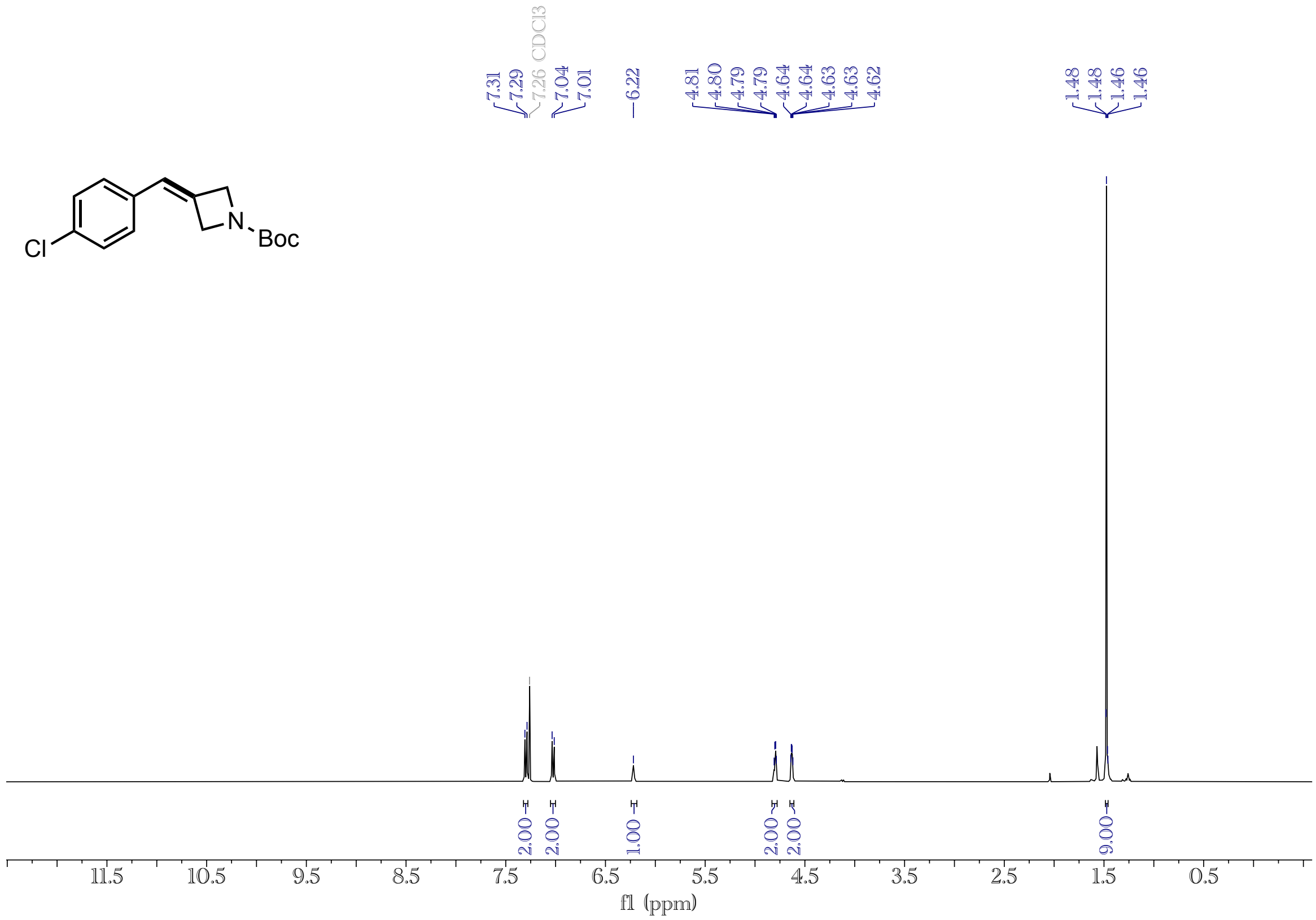

^{13}C {^{1}H} NMR of *tert*-butyl 3-(4-chlorobenzylidene)azetidine-1-carboxylate (P23)

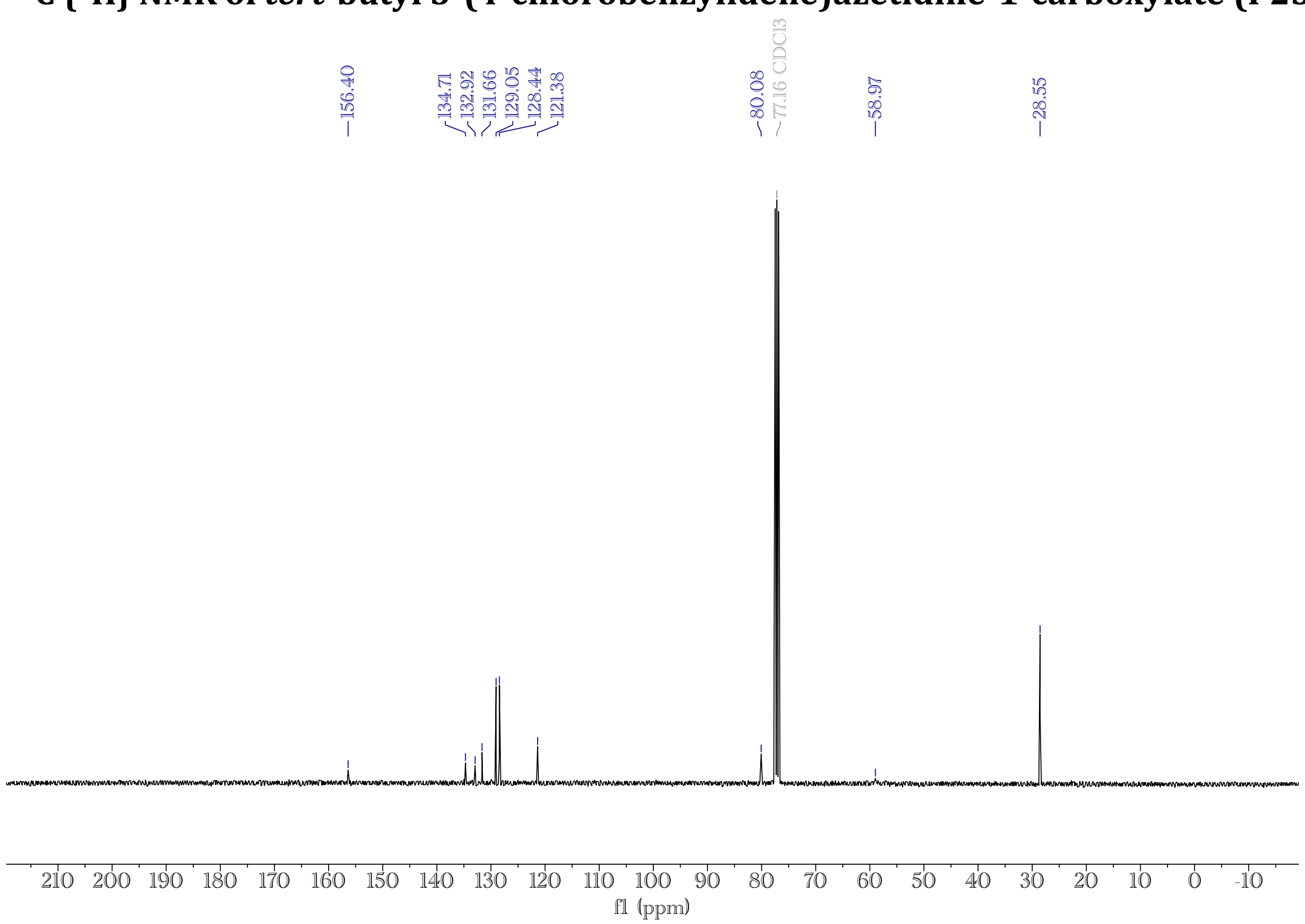

^{1}H NMR of (1R,4*S*)-2-((*E*/*Z*)-4-chlorobenzylidene)bicyclo[2.2.1]heptane (P24)

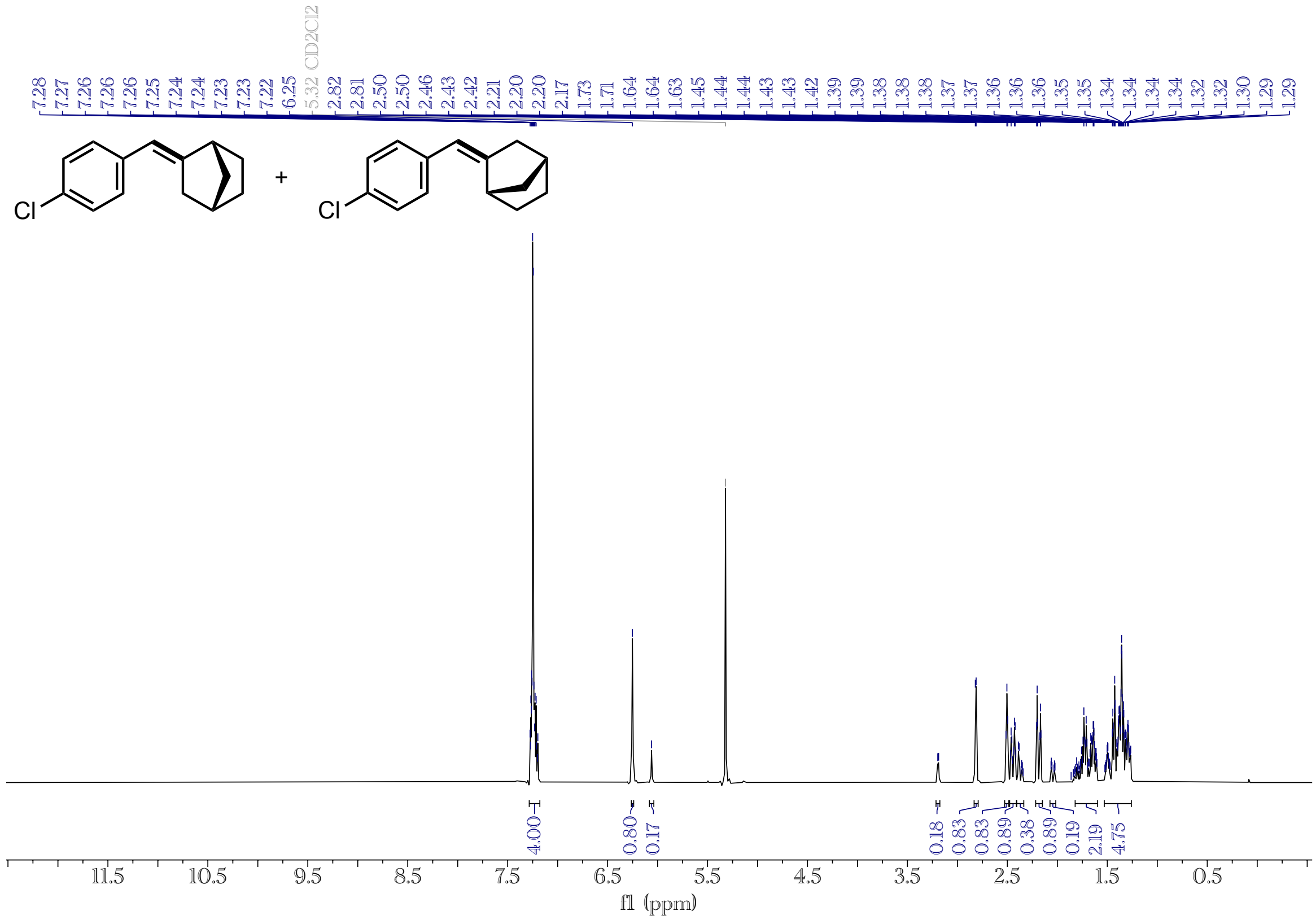

^{13}C NMR (1R,4S)-2-((E/Z)-4-chlorobenzylidene)bicyclo[2.2.1]heptane (P24)

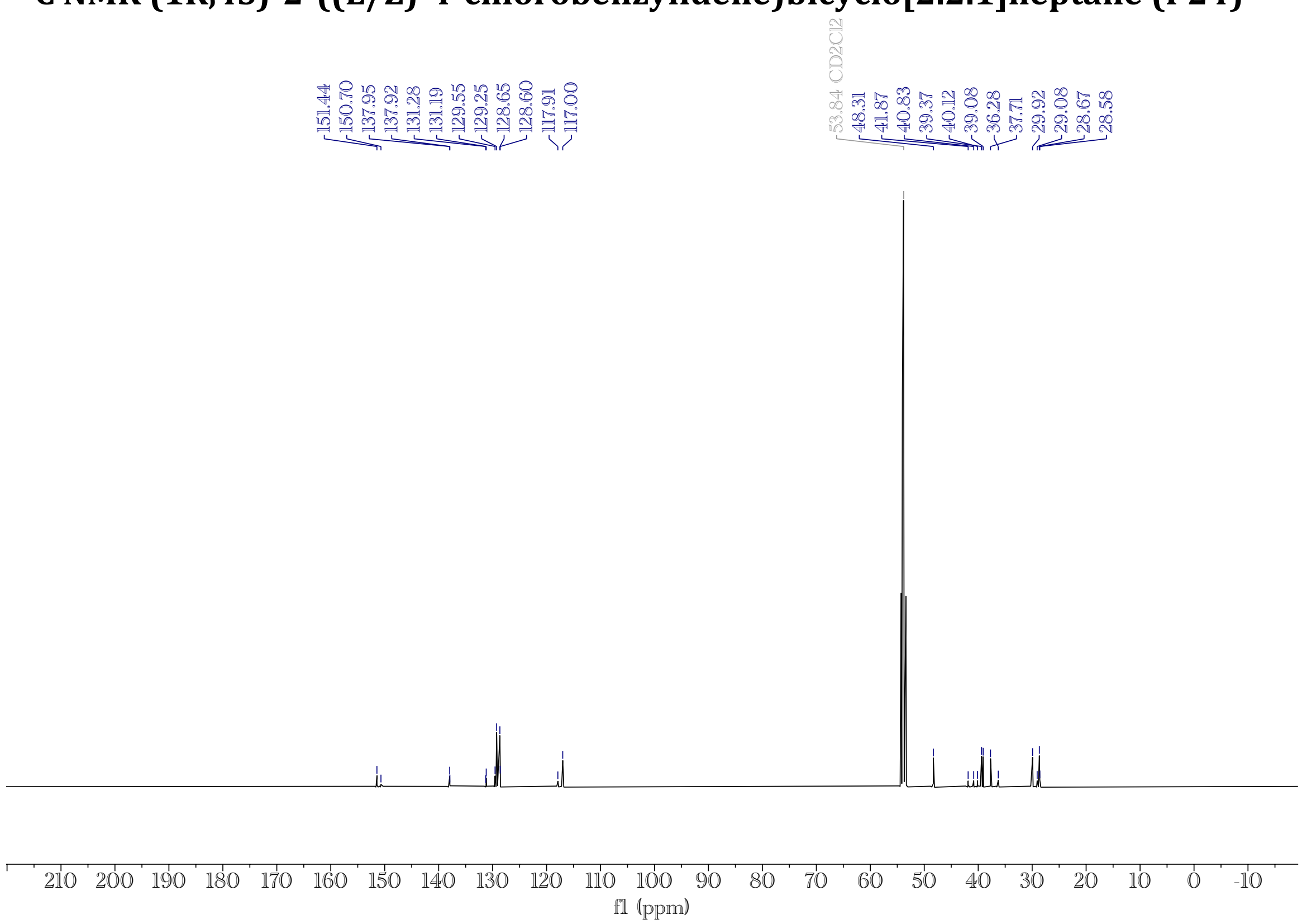

^{1}H NMR of 2-(4-chlorobenzylidene)adamantane (P25)

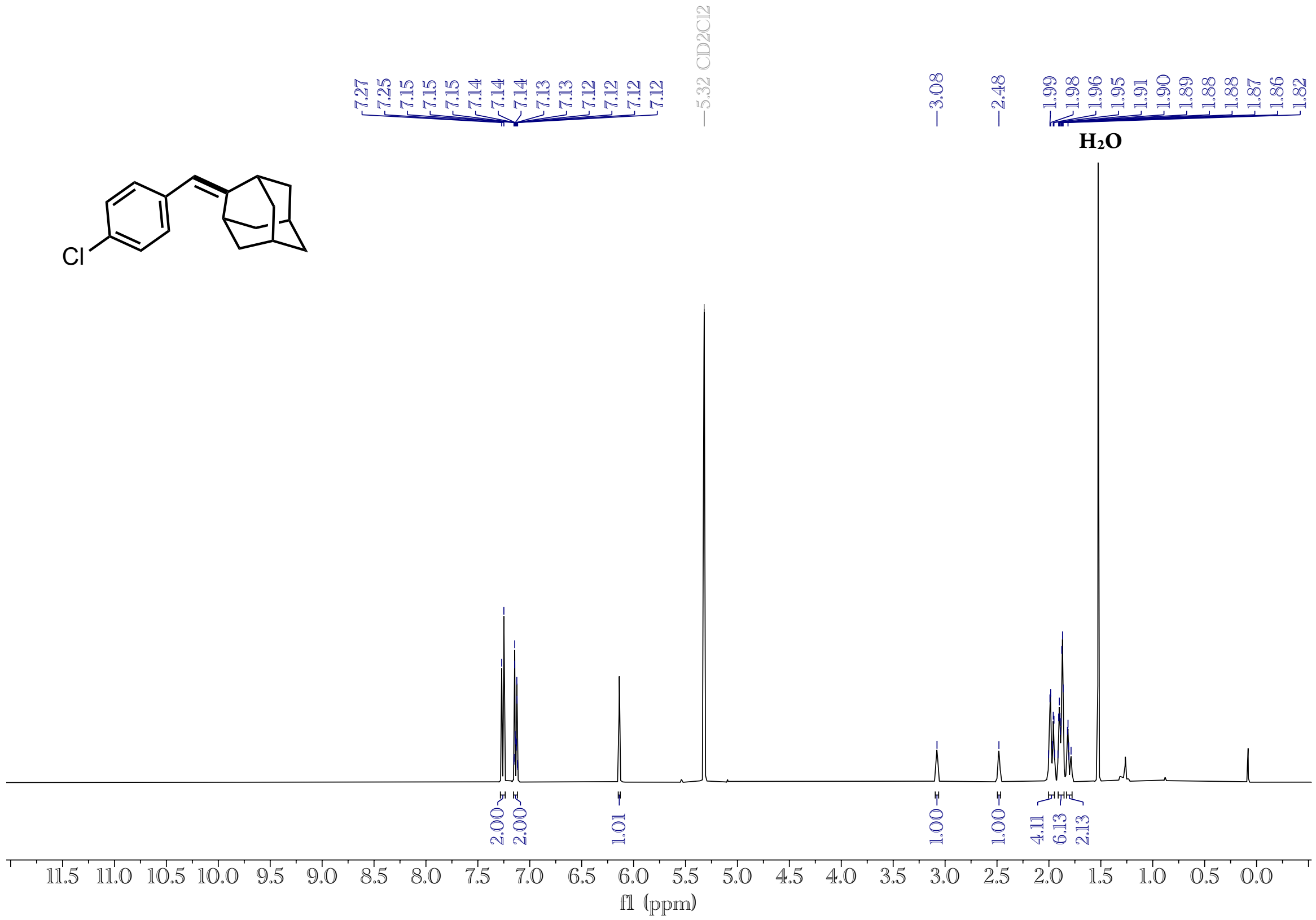

$^{13}C\{^{1}H\}$ NMR of 2-(4-chlorobenzylidene)adamantane (P25)

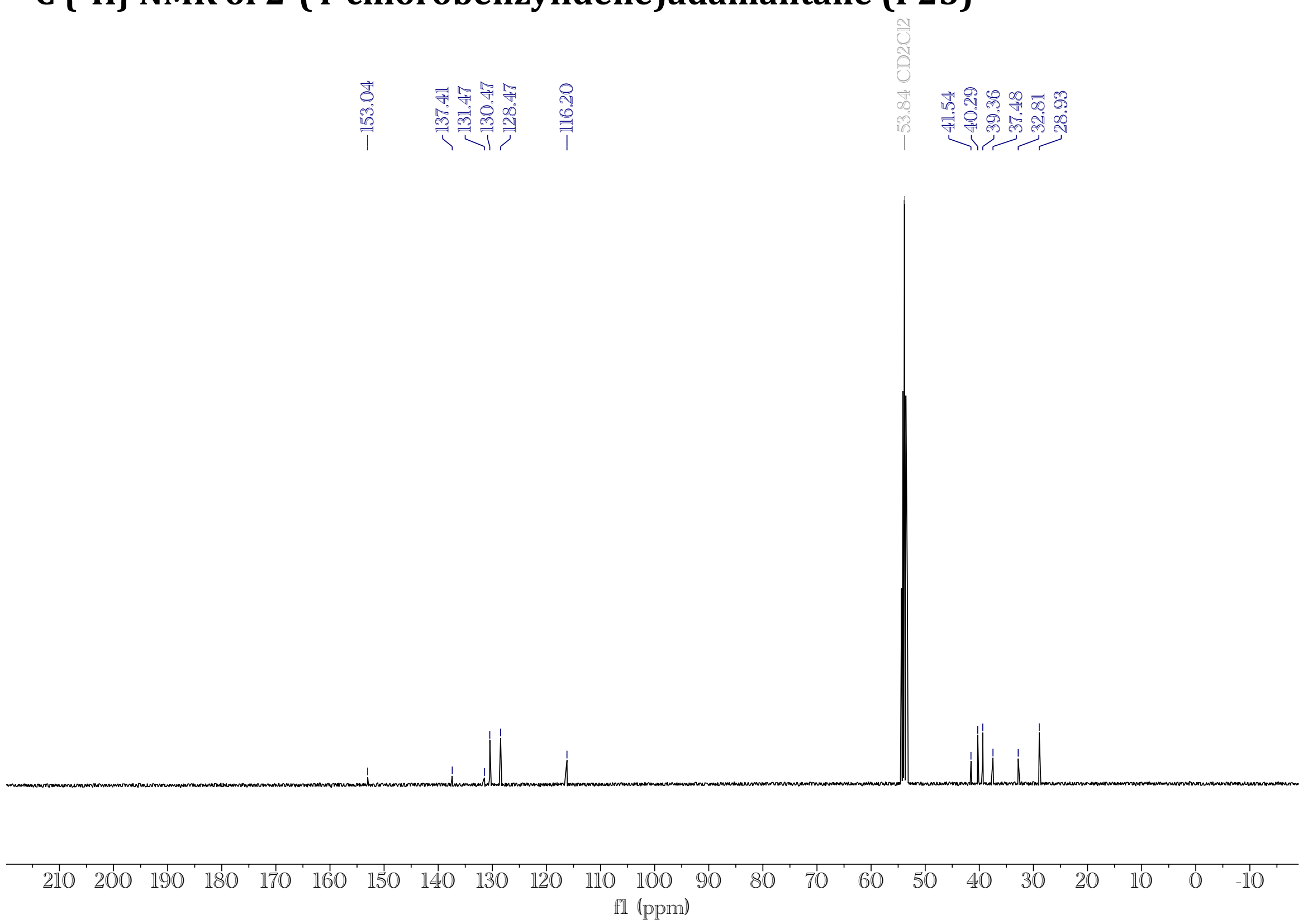

¹H NMR of (4-chlorobenzylidene)cyclododecane (P26)

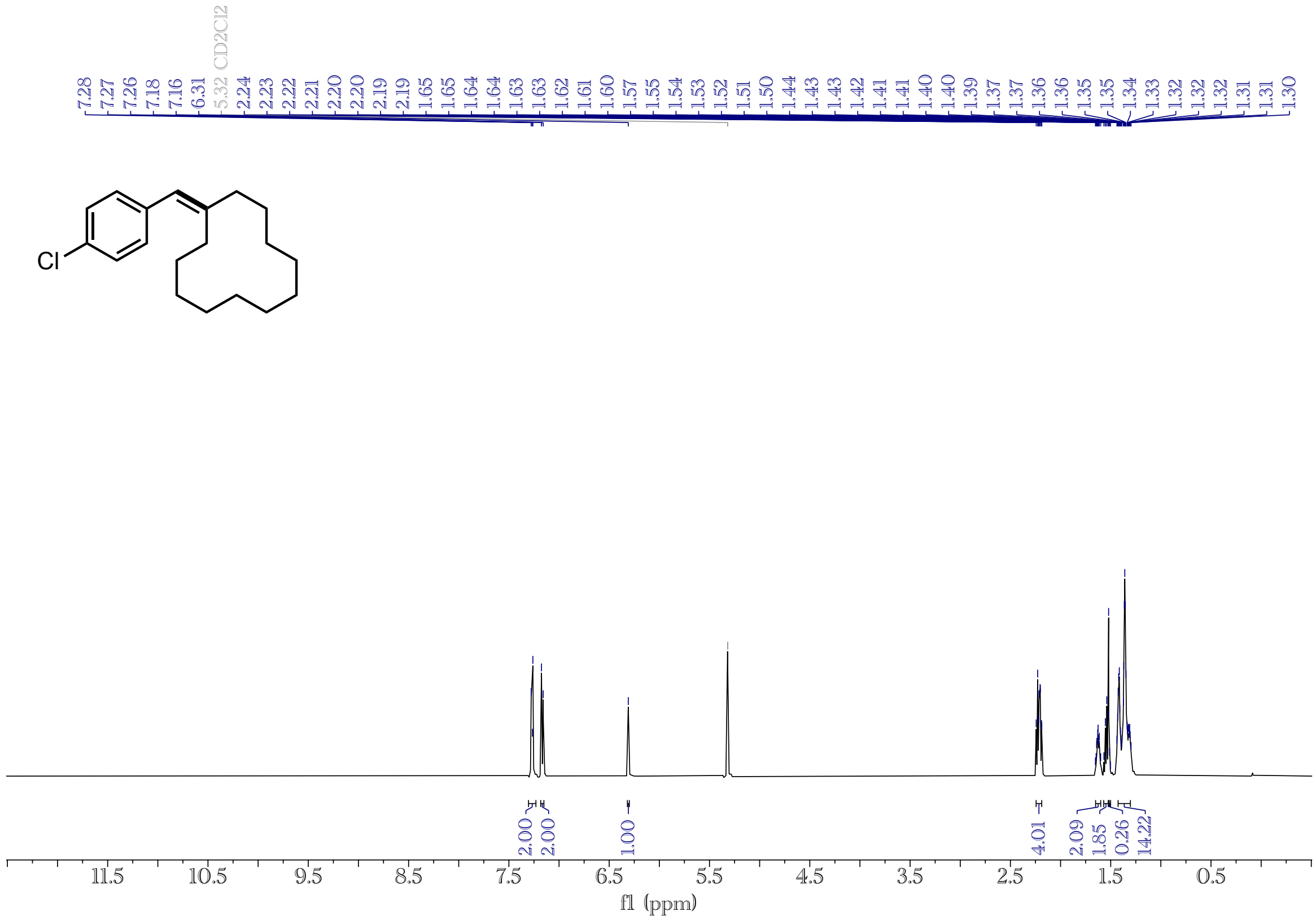

¹³C {¹H} NMR of (4-chlorobenzylidene)cyclododecane (P26)

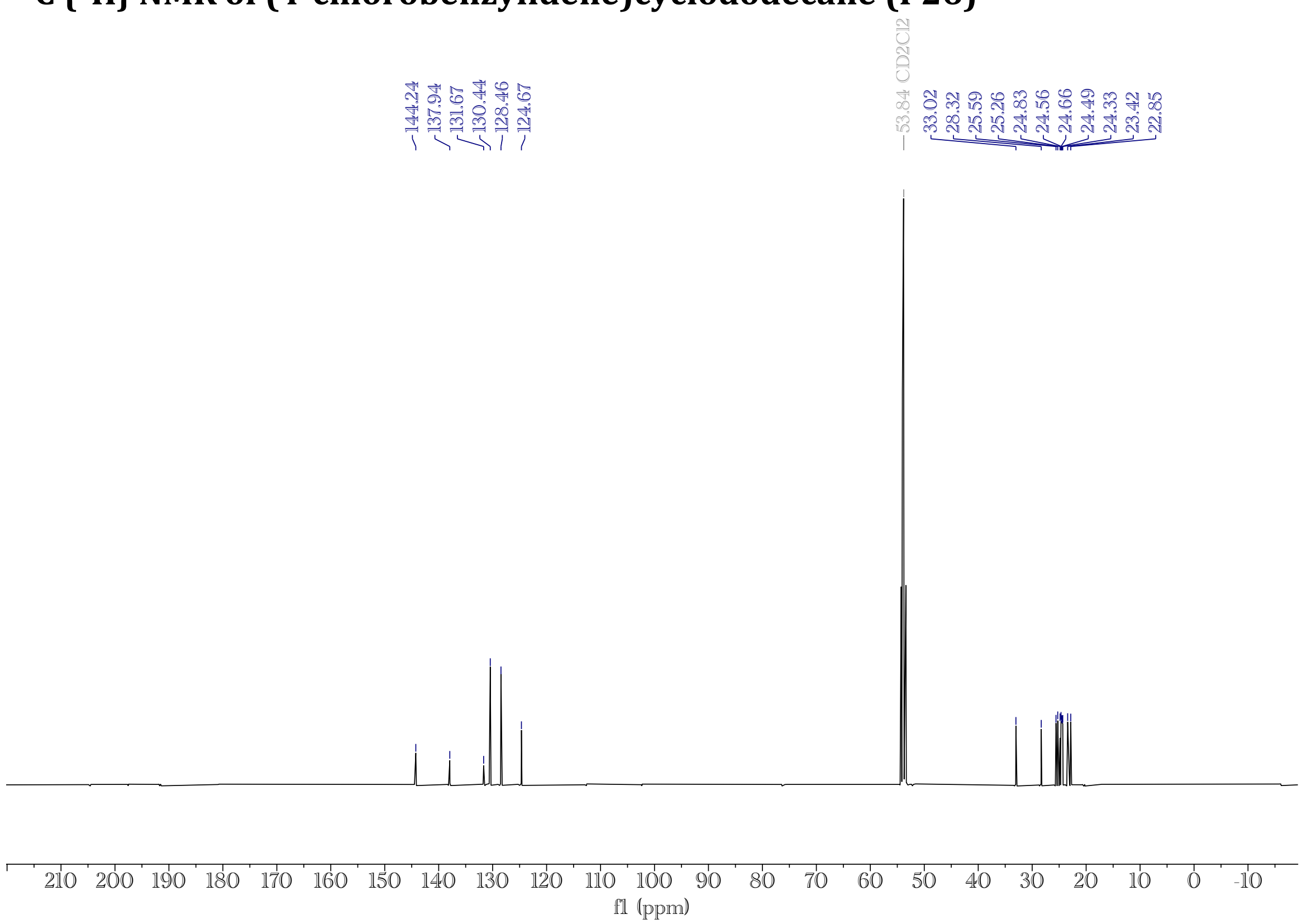

^1H NMR of methyl 4-(2-methylprop-1-en-1-yl)benzoate (P27)

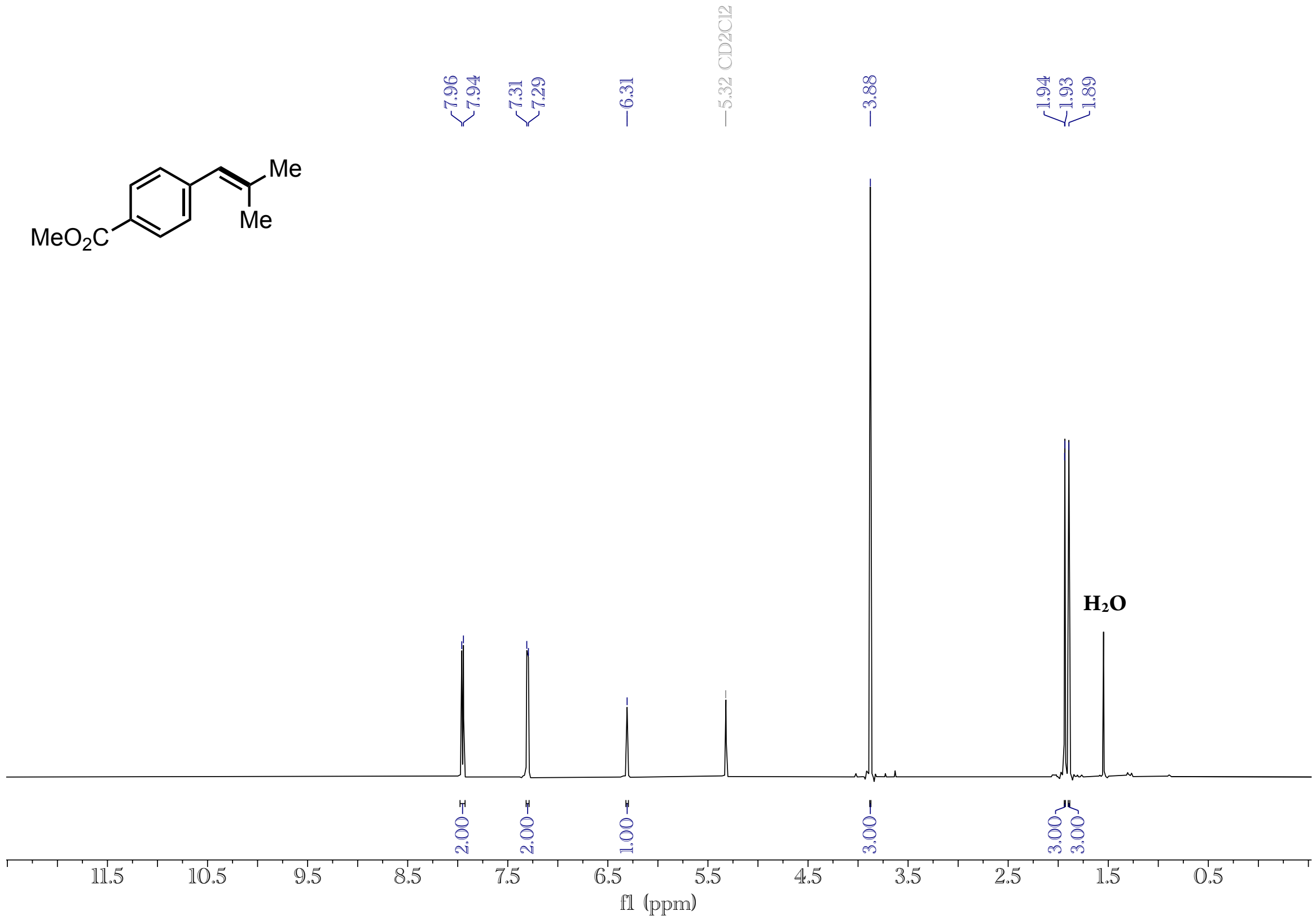

^{13}C {^1H} NMR of methyl 4-(2-methylprop-1-en-1-yl)benzoate (P27)

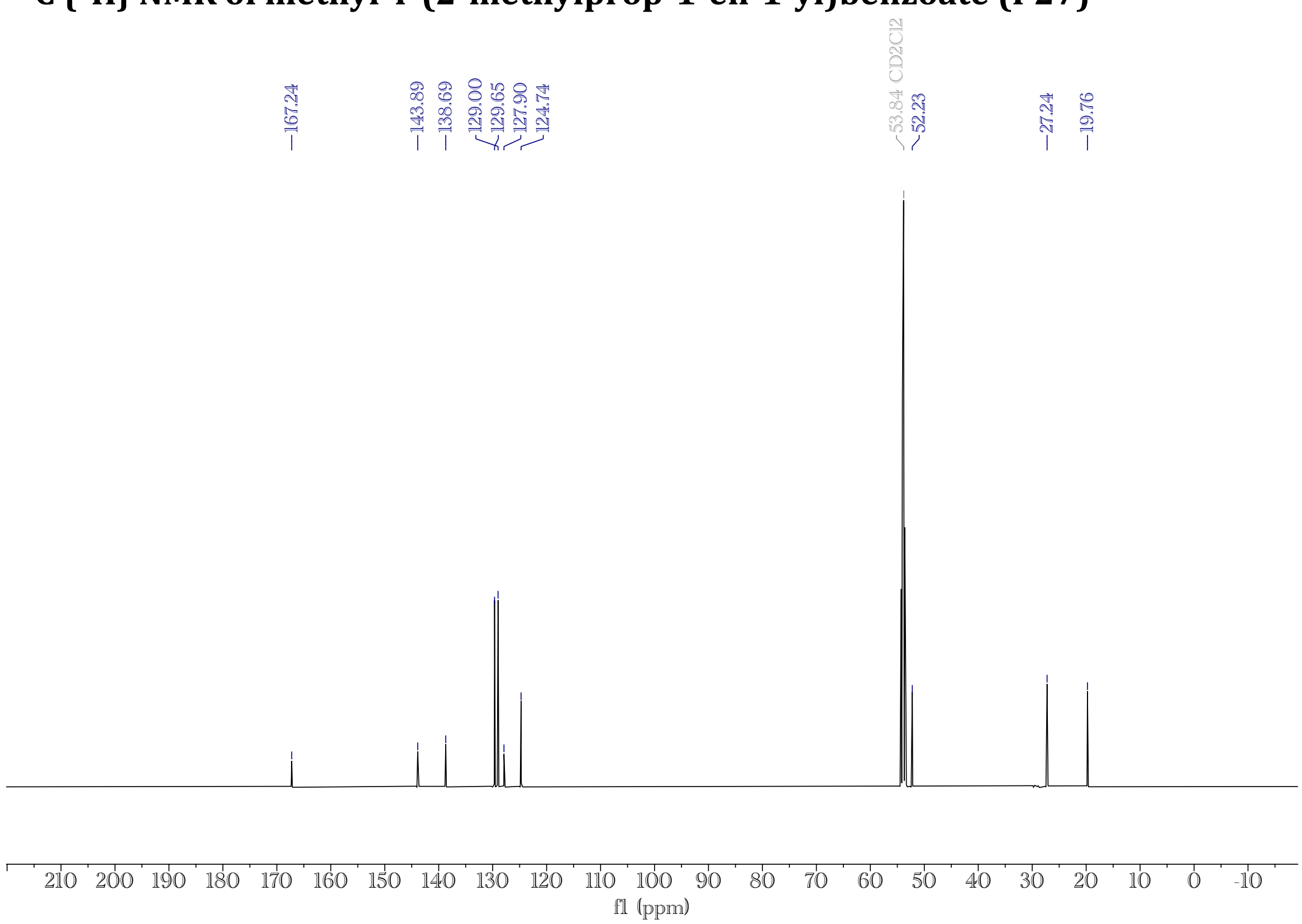

^{1}H NMR of dibenzyl 4-benzylideneheptanedioate (P28)

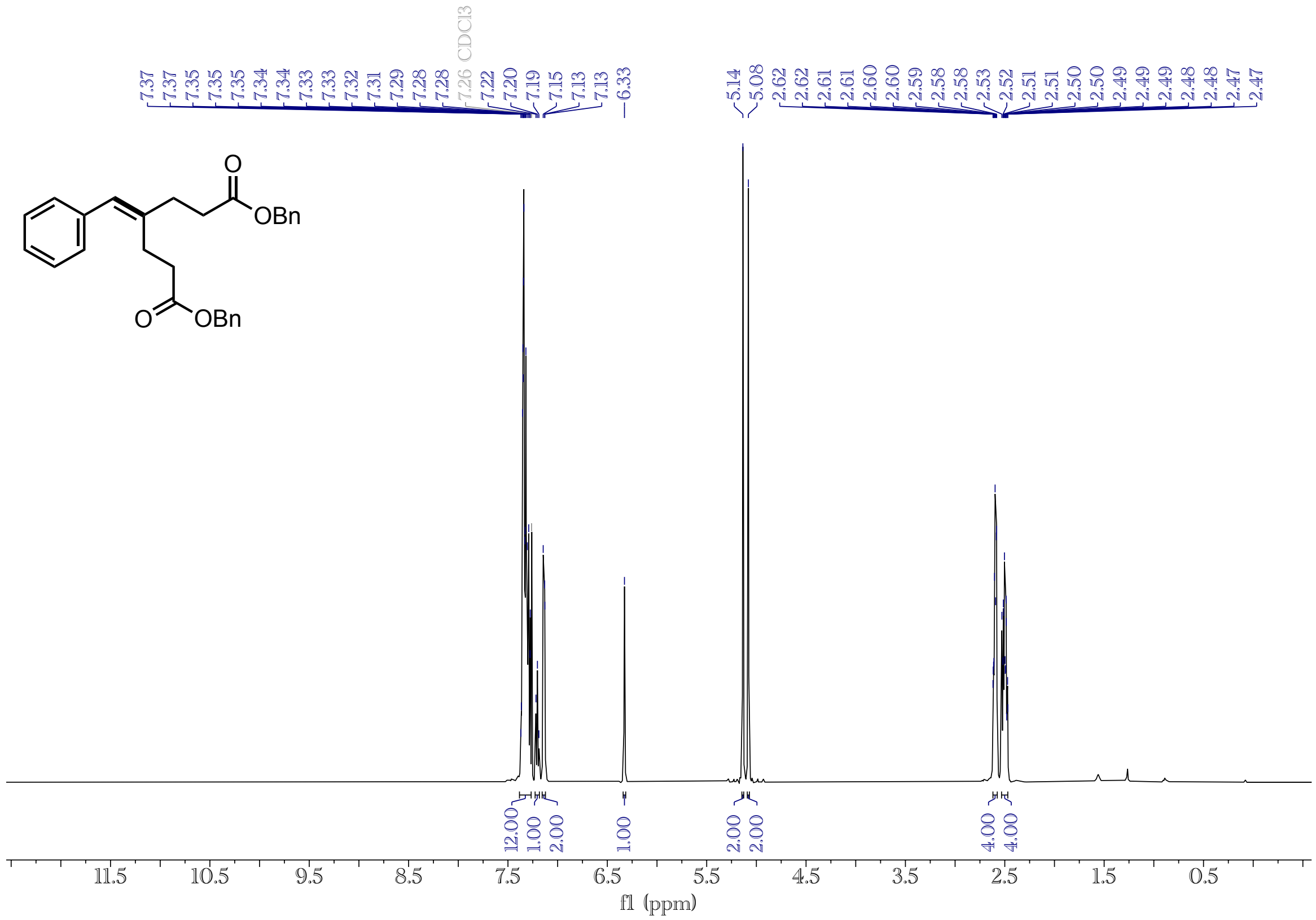

^{13}C {^{1}H} NMR of dibenzyl 4-benzylideneheptanedioate (P28)

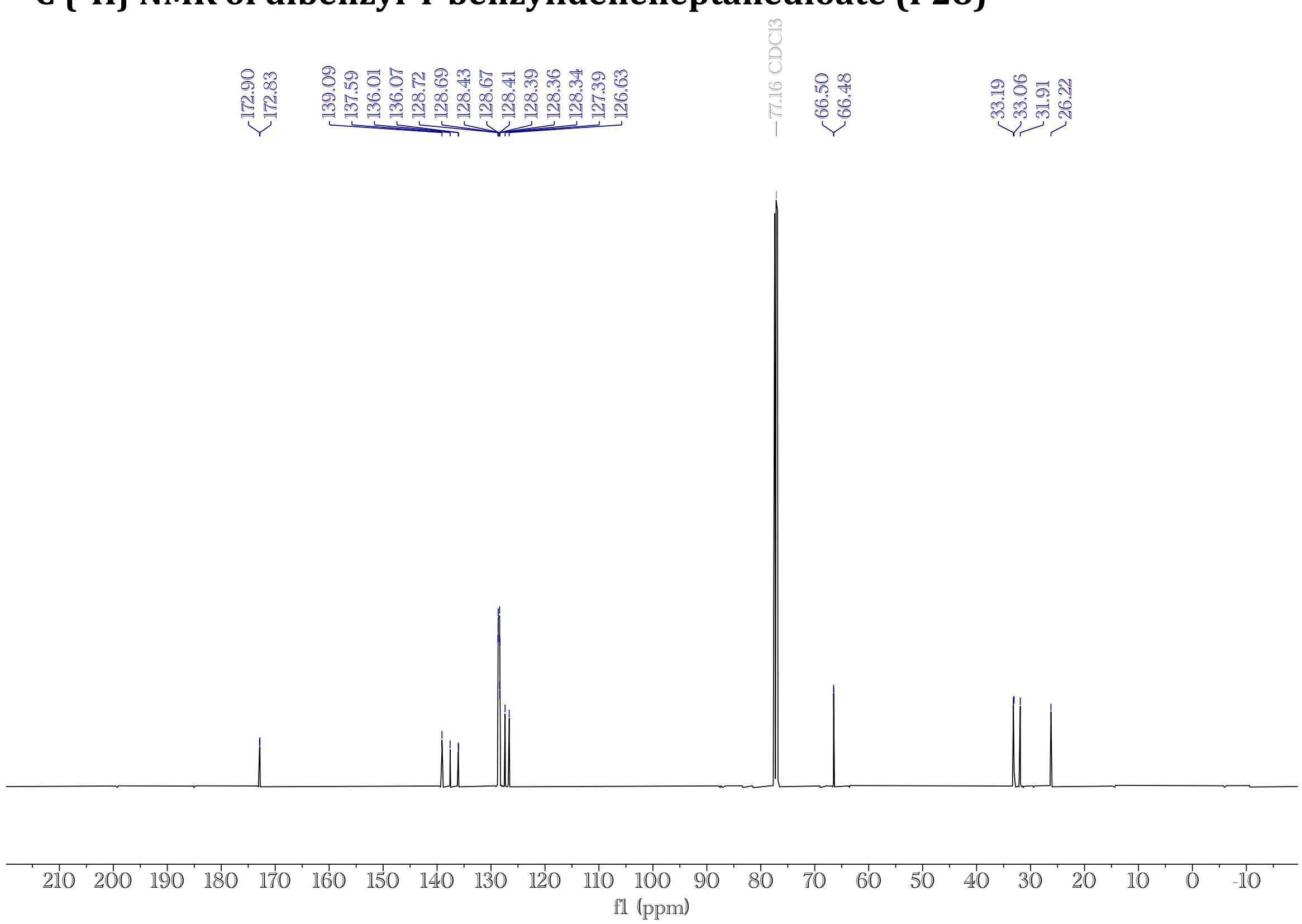

^1H NMR of tetraethyl (3-benzylidenepentane-1,5-diyl)bis(phosphonate) (P29)

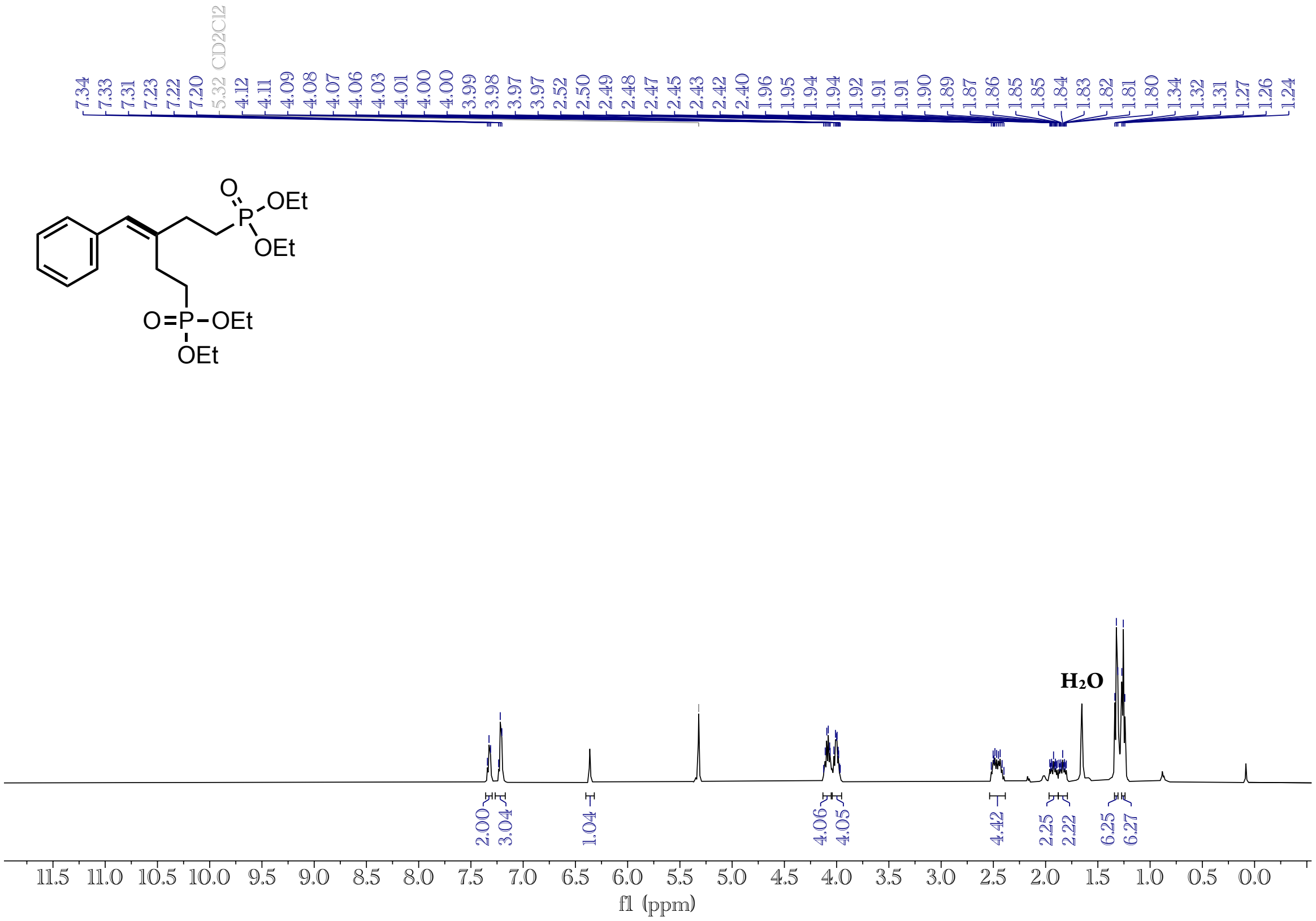

^{13}C {^1H} NMR of tetraethyl (3-benzylidenepentane-1,5-diyl)bis(phosphonate) (P29)

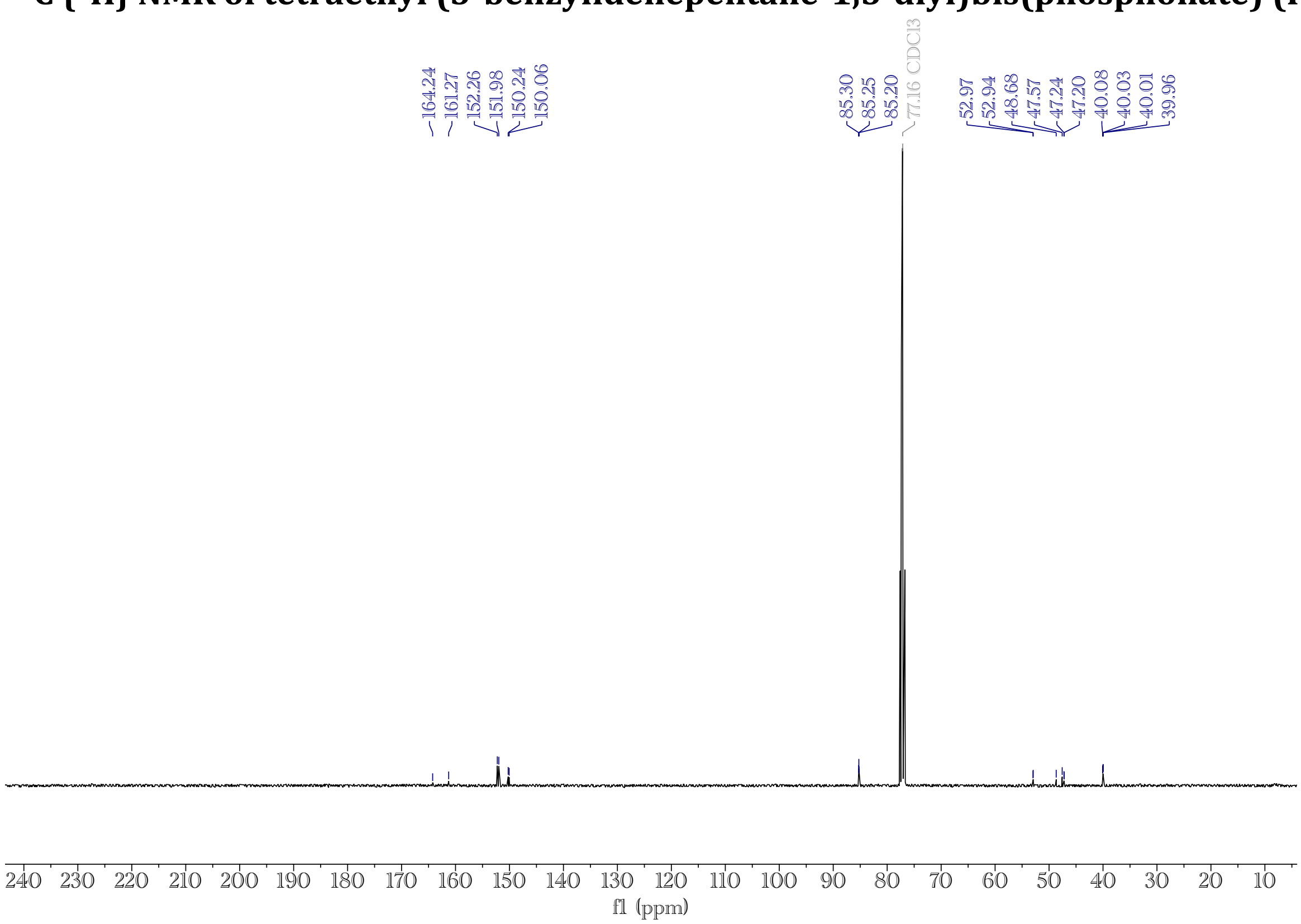

^{31}P {^{1}H} NMR of tetraethyl (3-benzylidenepentane-1,5-diyl)bis(phosphonate) (P29)

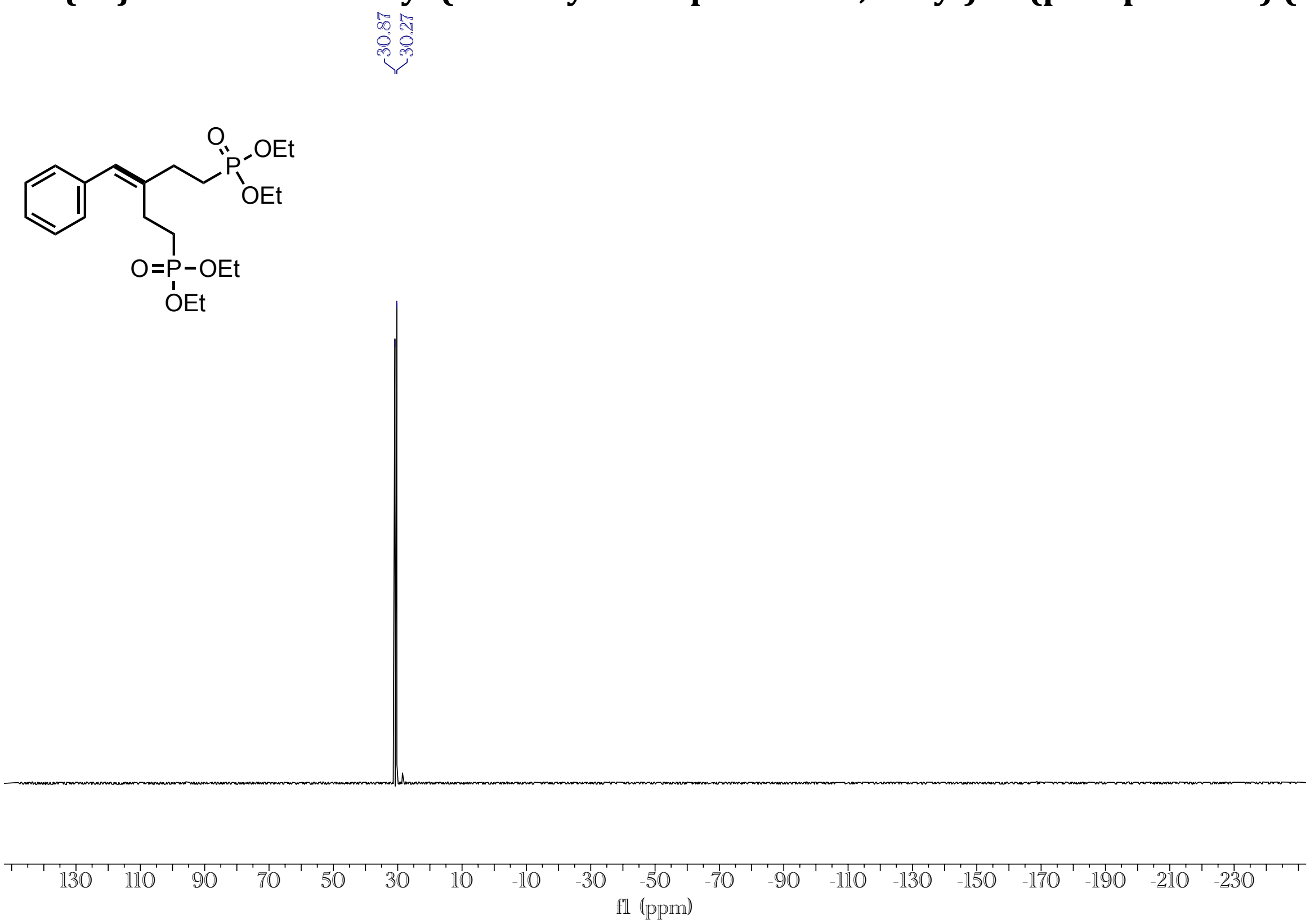

^{1}H NMR of (*E/Z*)-5-(2-(4-chlorobenzylidene)butyl)benzo[d][1,3]dioxole (P30)

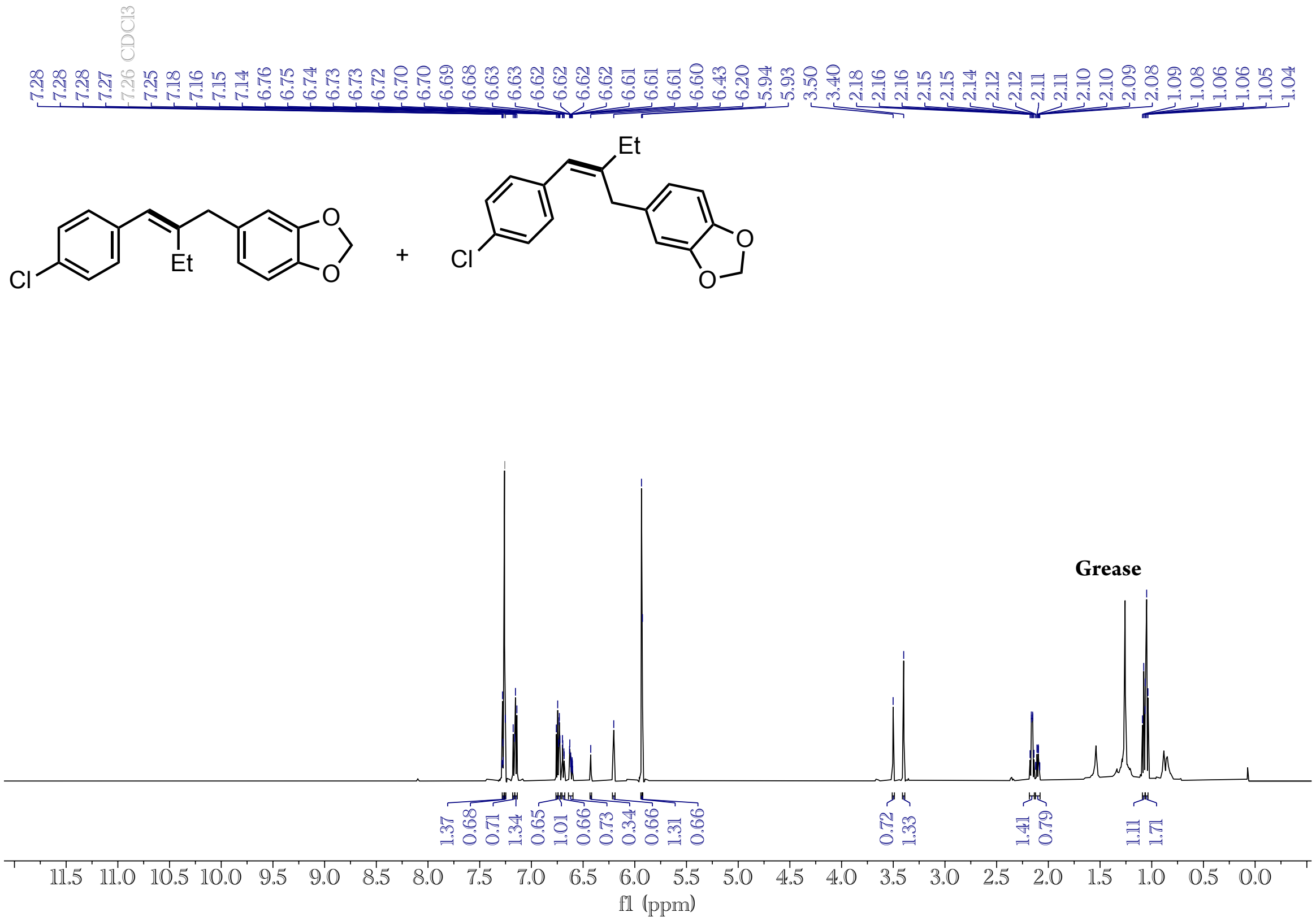

^{13}C {^{1}H} NMR of (*E/Z*)-5-(2-(4-chlorobenzylidene)butyl)benzo[d][1,3]dioxole (P30)

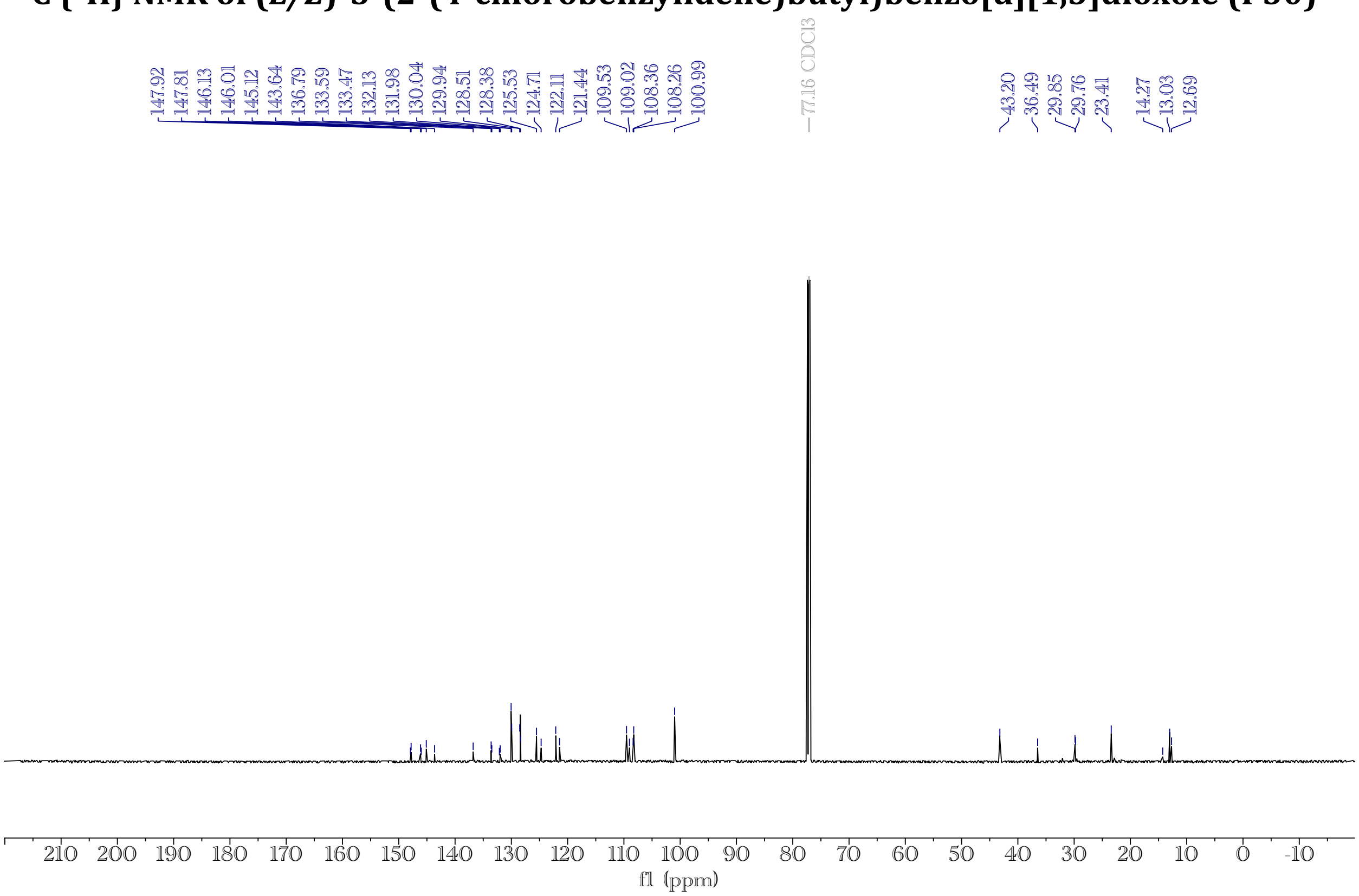

^{1}H NMR of (*E/Z*)-4-(2-(4-chlorobenzylidene)butyl)benzonitrile (P31)

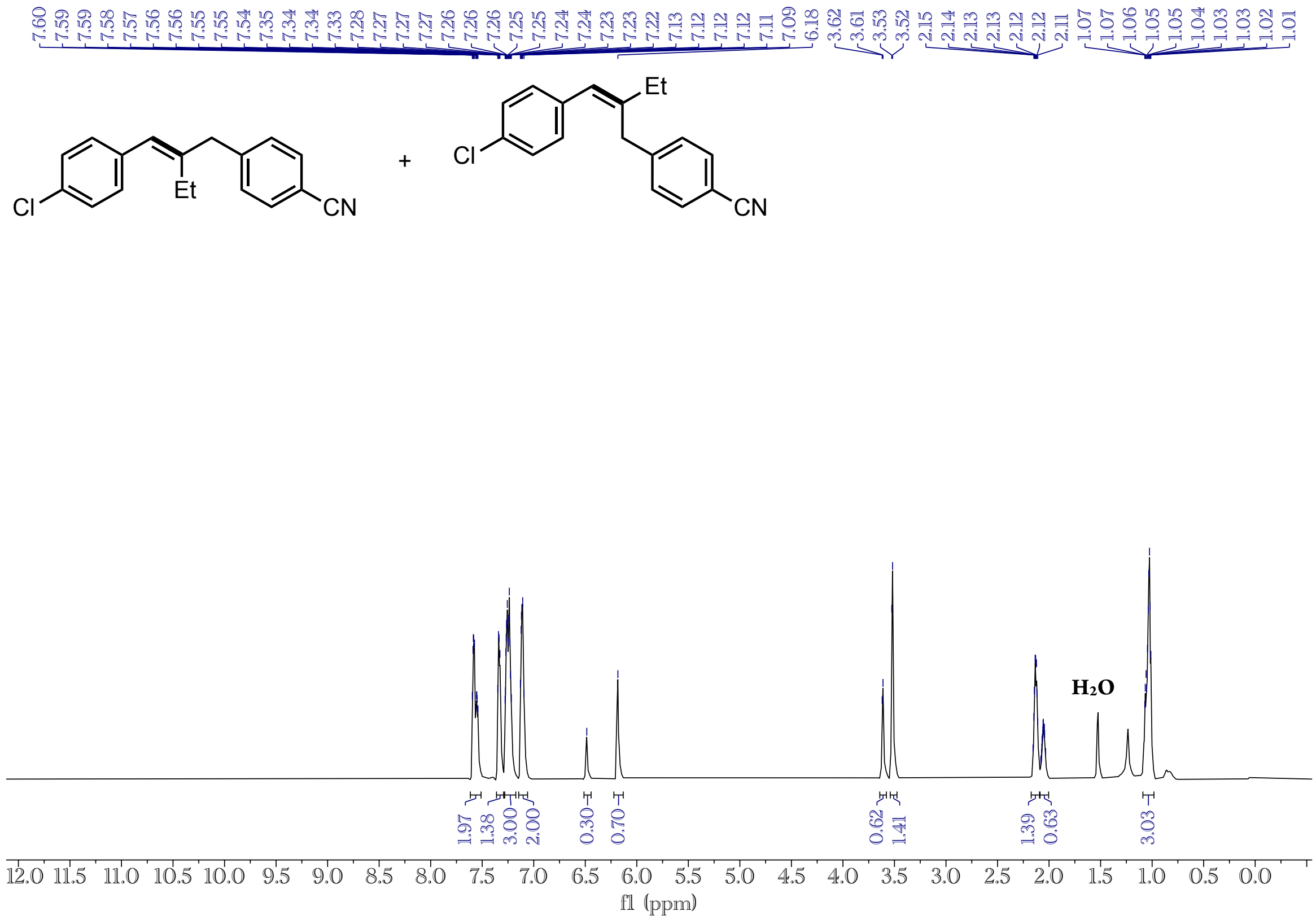

^{13}C {^{1}H} NMR of (*E/Z*)-4-(2-(4-chlorobenzylidene)butyl)benzonitrile (P31)

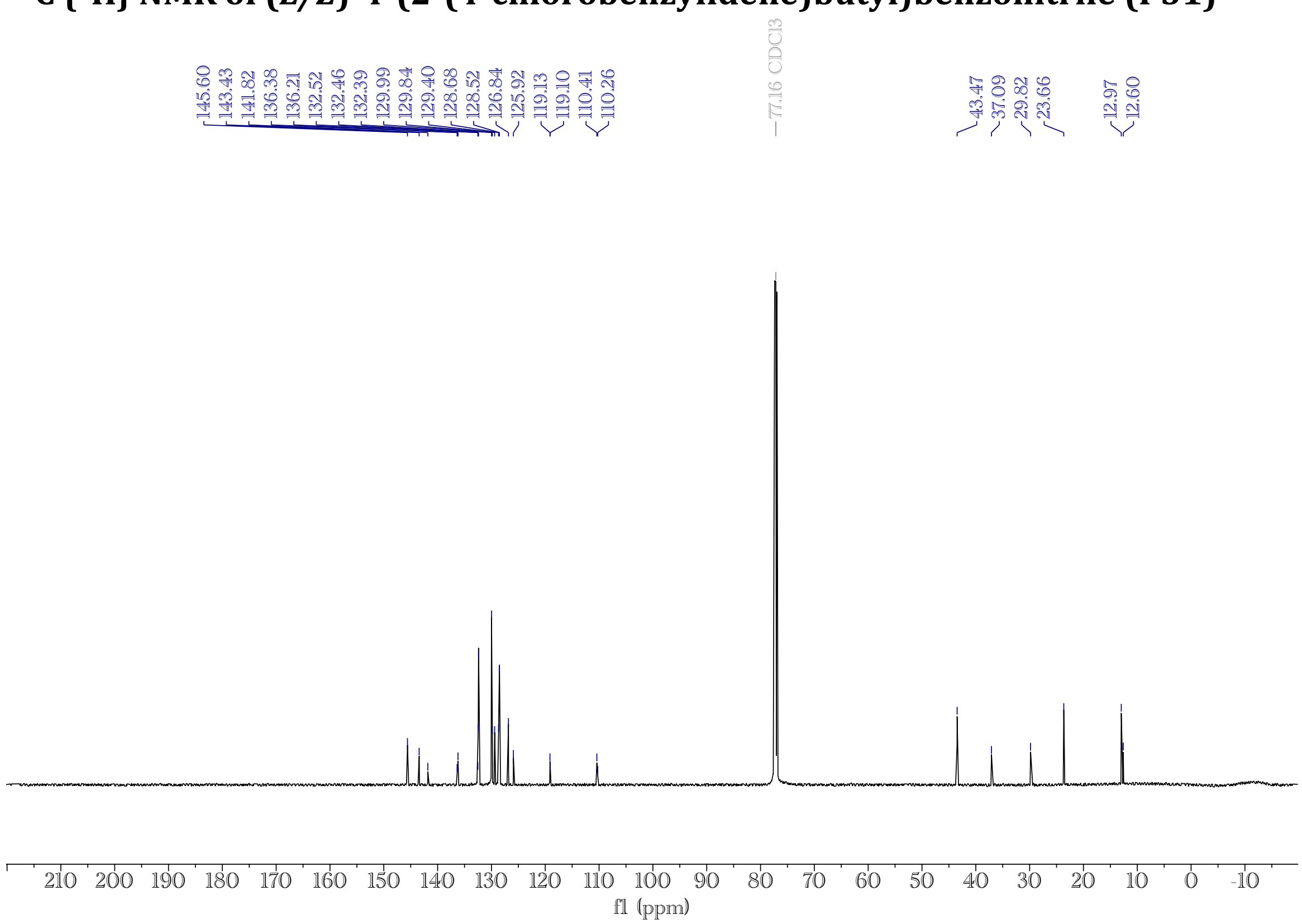

1H NMR of 2-cinnamyl-1,3-dioxolane (P32)

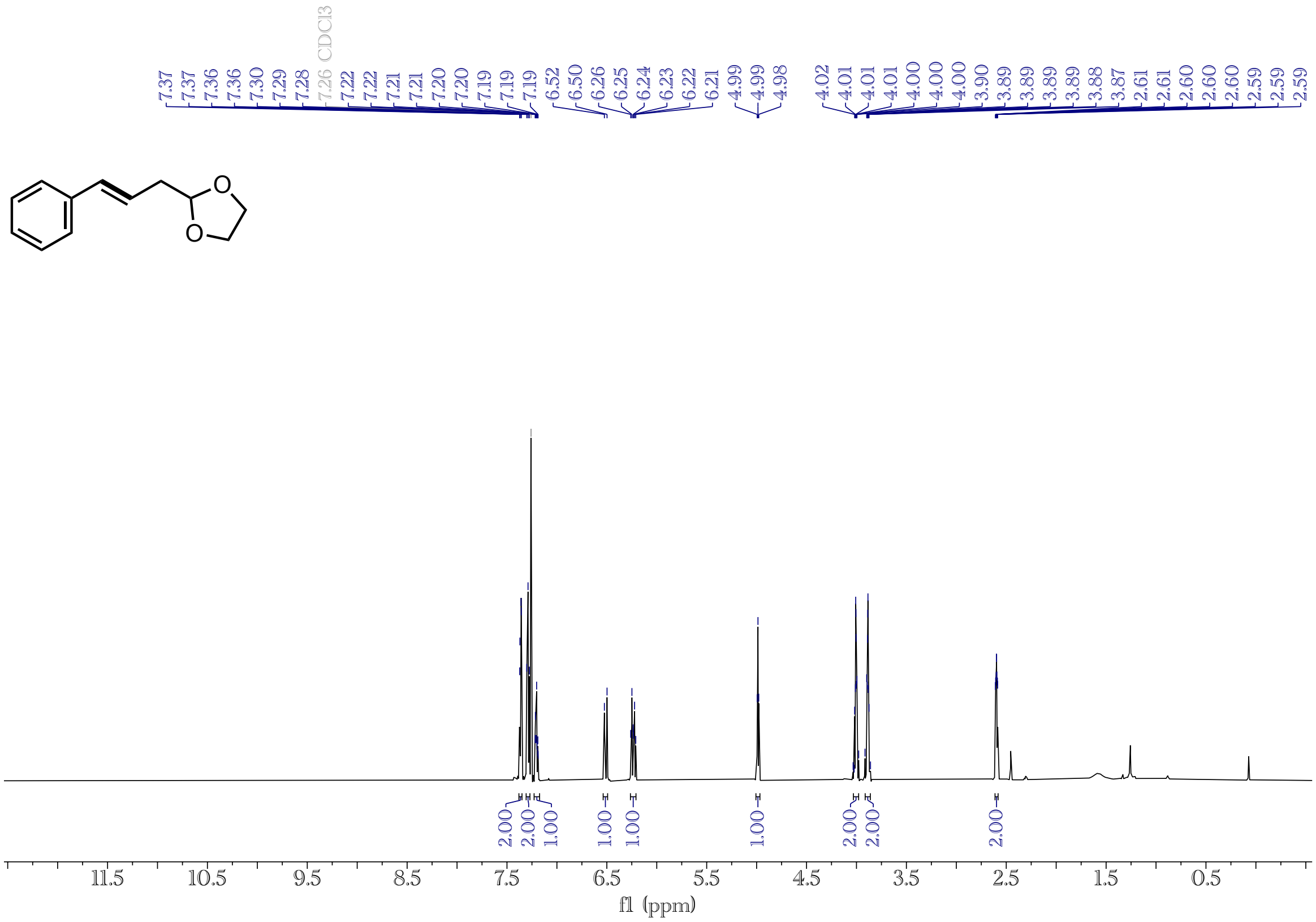

13C {1H} NMR of 2-cinnamyl-1,3-dioxolane (P32)

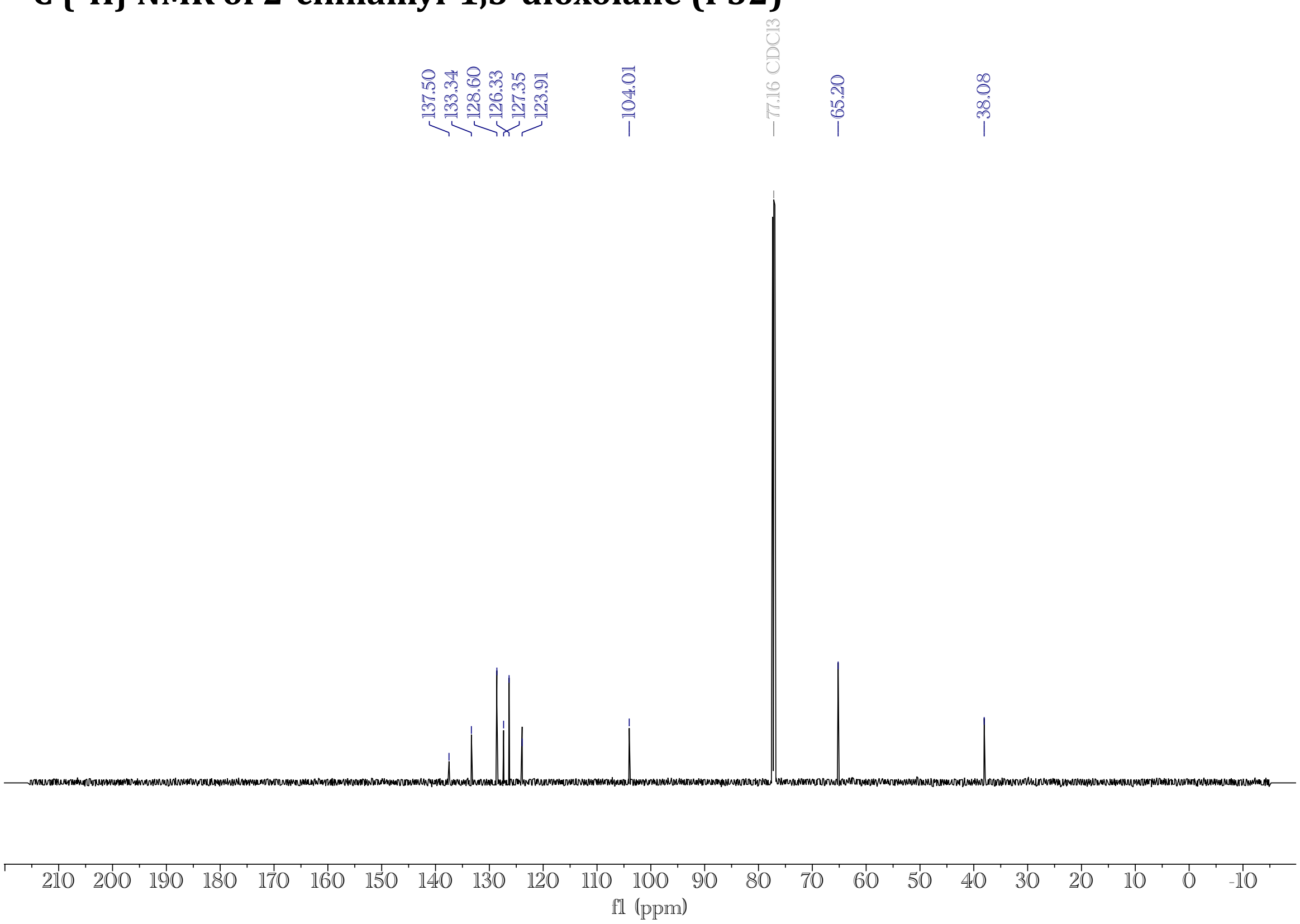

^{1}H NMR of dimethyl 4,4'-(but -1-ene-1,2-diyl)(*E/Z*)-dibenzoate (P36)

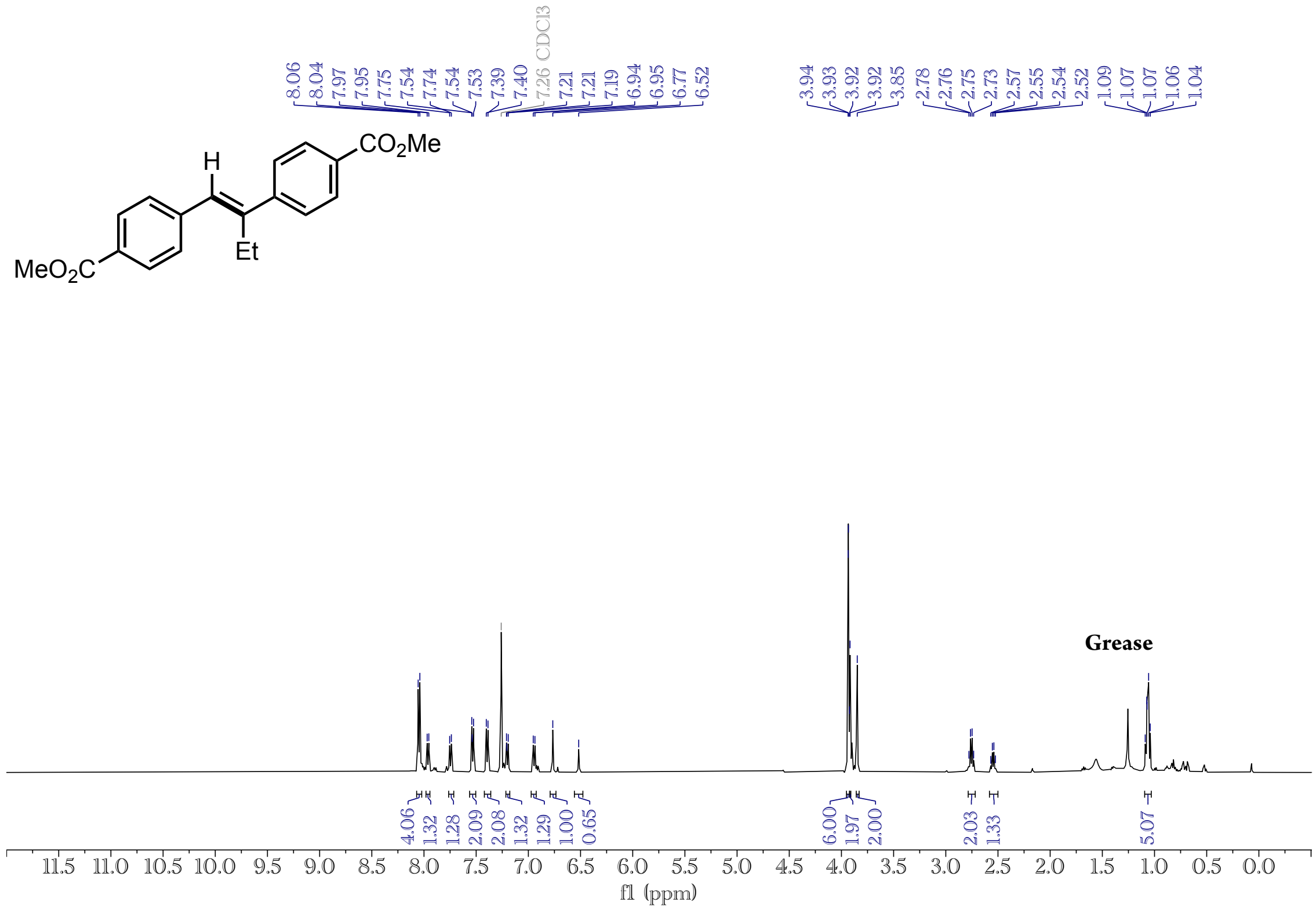

^{13}C {^{1}H} NMR of dimethyl 4,4'-(but-1-ene-1,2-diyl)(*E/Z*)-dibenzoate (P36)

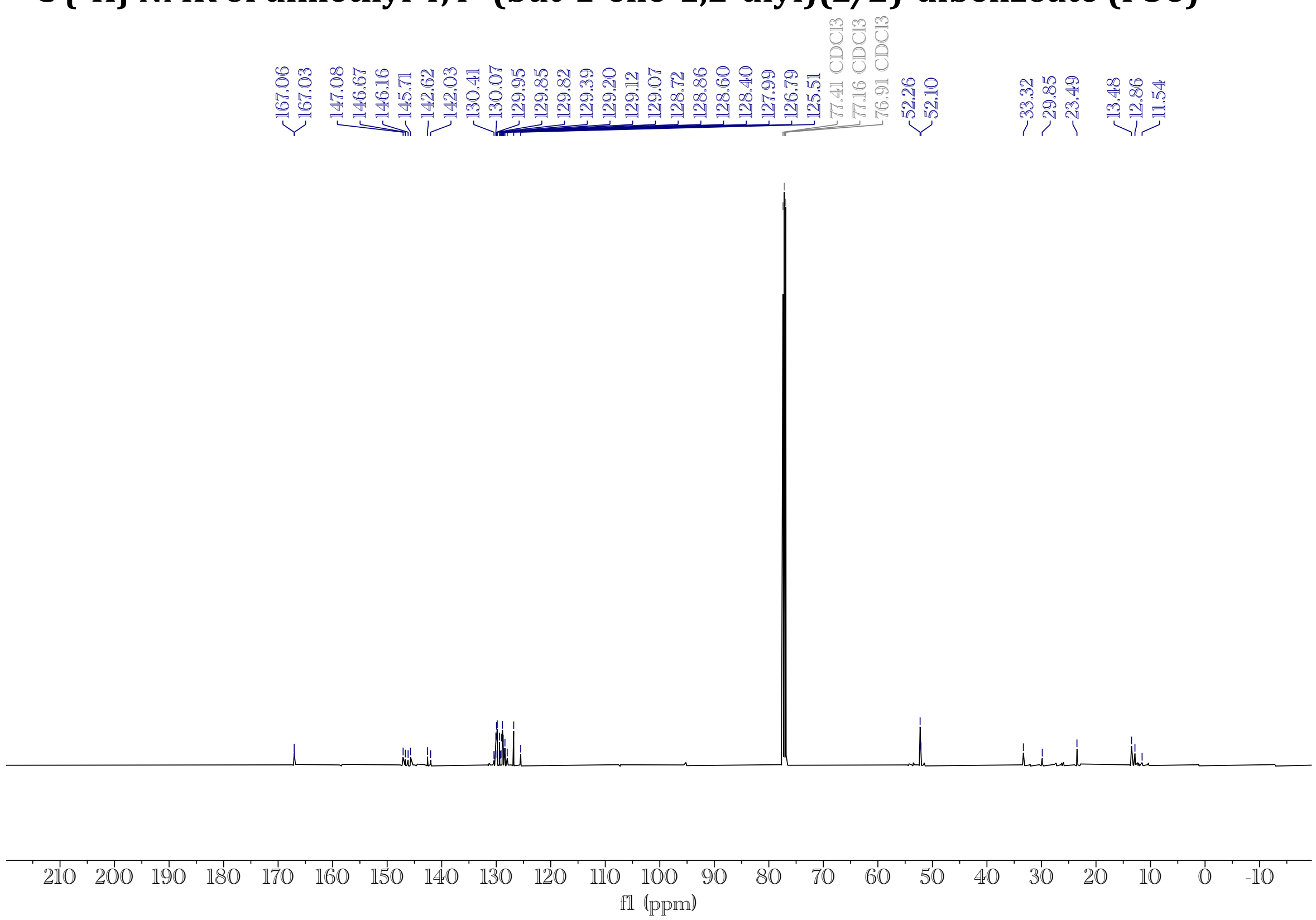

^{1}H NMR of 1-((*Z*)-2-(4-(((1*R*,2*S*,5*R*)-2-isopropyl-5-methylcyclohexyl)oxy)phenyl)but-1-en-1-yl)-3,5-bis(trifluoromethyl)benzene (P37)

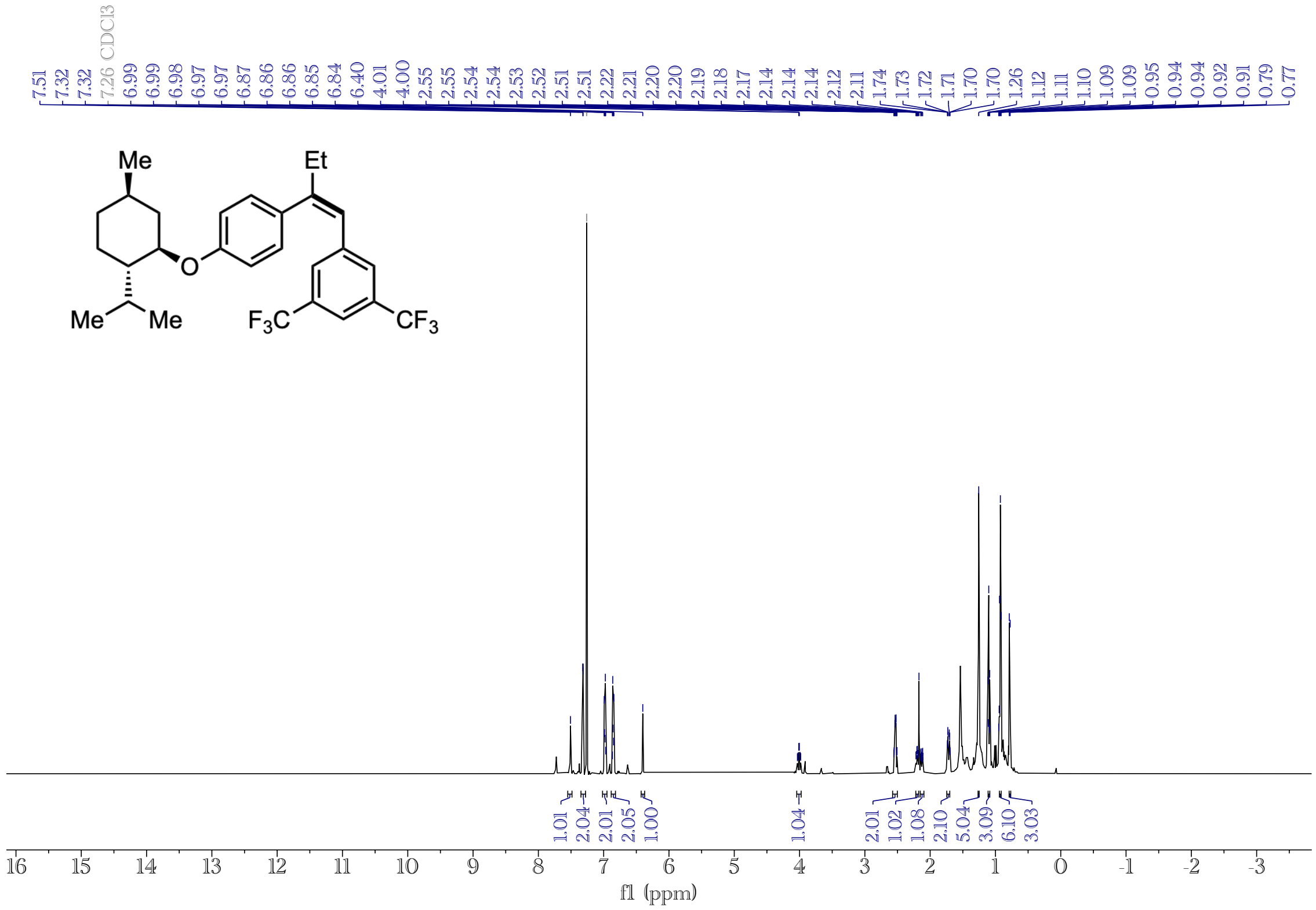

^{13}C {^{1}H} NMR of 1-((*Z*)-2-(4-(((1*R*,2*S*,5*R*)-2-isopropyl-5-methylcyclohexyl)oxy)phenyl)but-1-en-1-yl)-3,5-bis(trifluoromethyl)benzene (P37)

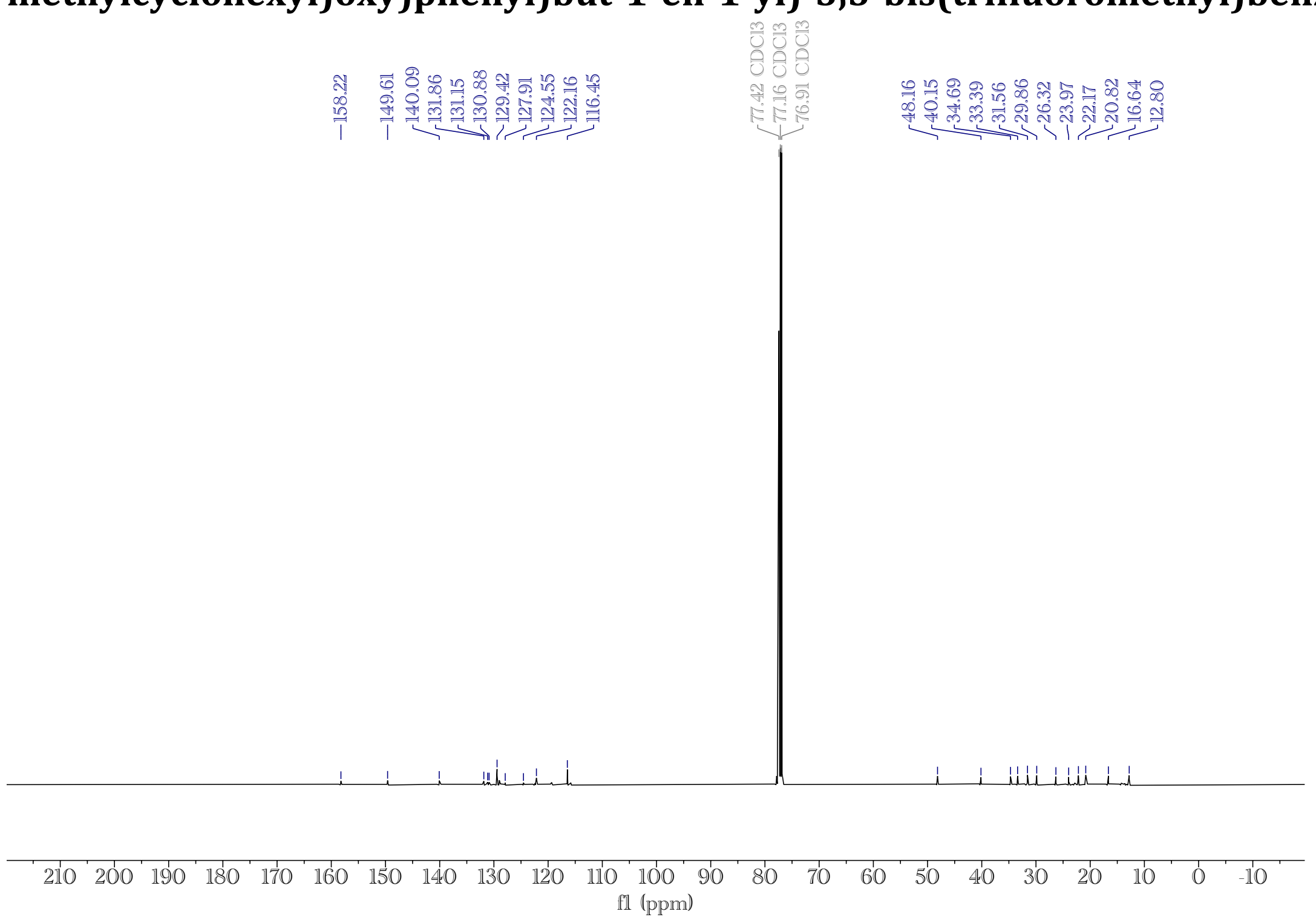

$^{19}F\ \{^{1}H\}$ NMR of 1-((*Z*)-2-(4-(((1*R*,2*S*,5*R*)-2-isopropyl-5-methylcyclohexyl)oxy)phenyl)but-1-en-1-yl)-3,5-bis(trifluoromethyl)benzene (P37)

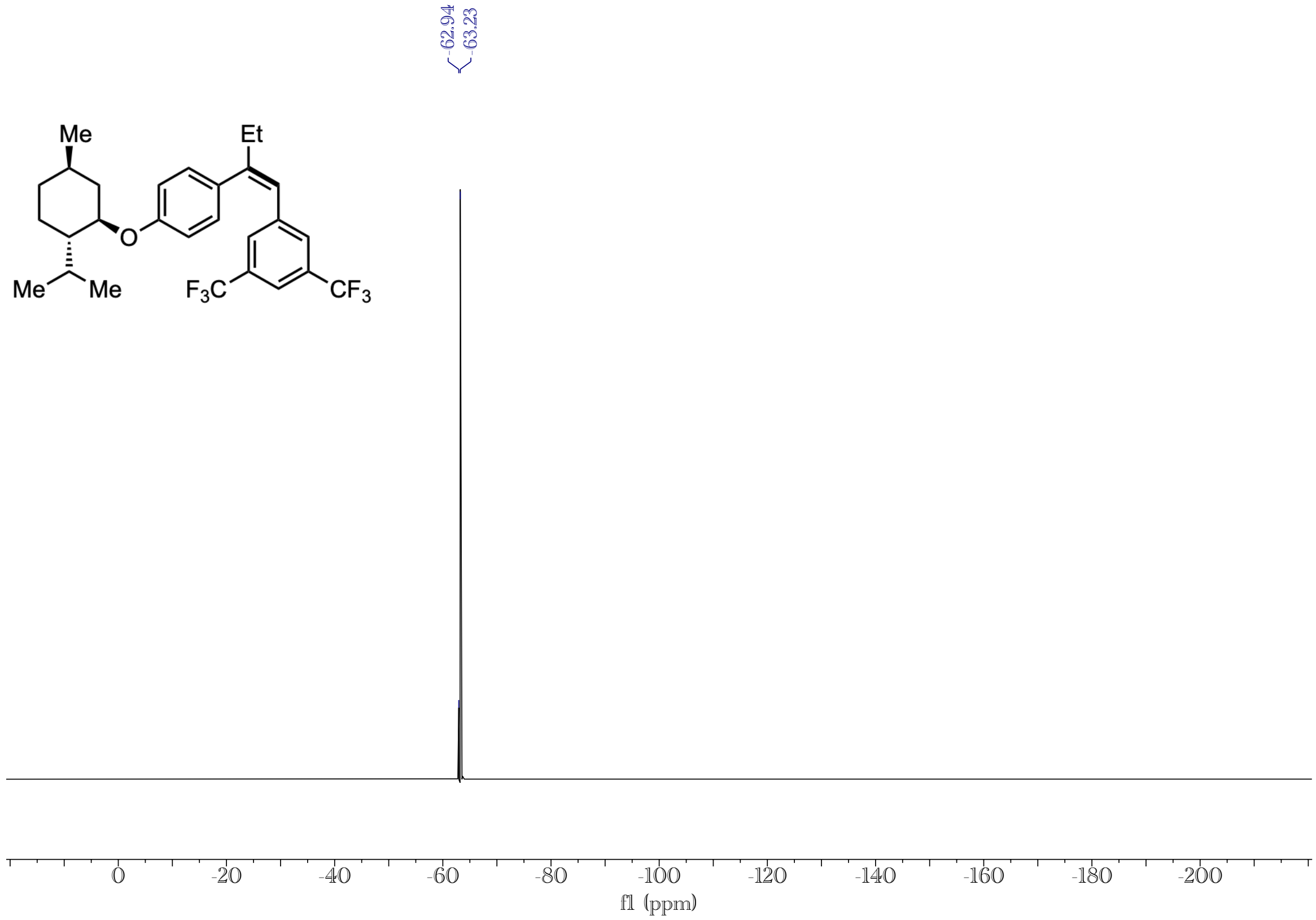

^{1}H NMR of (*E*/*Z*)-1-(2-phenylhepta-1,6-dien-1-yl)-3,5-bis(trifluoromethyl)benzene (P38)

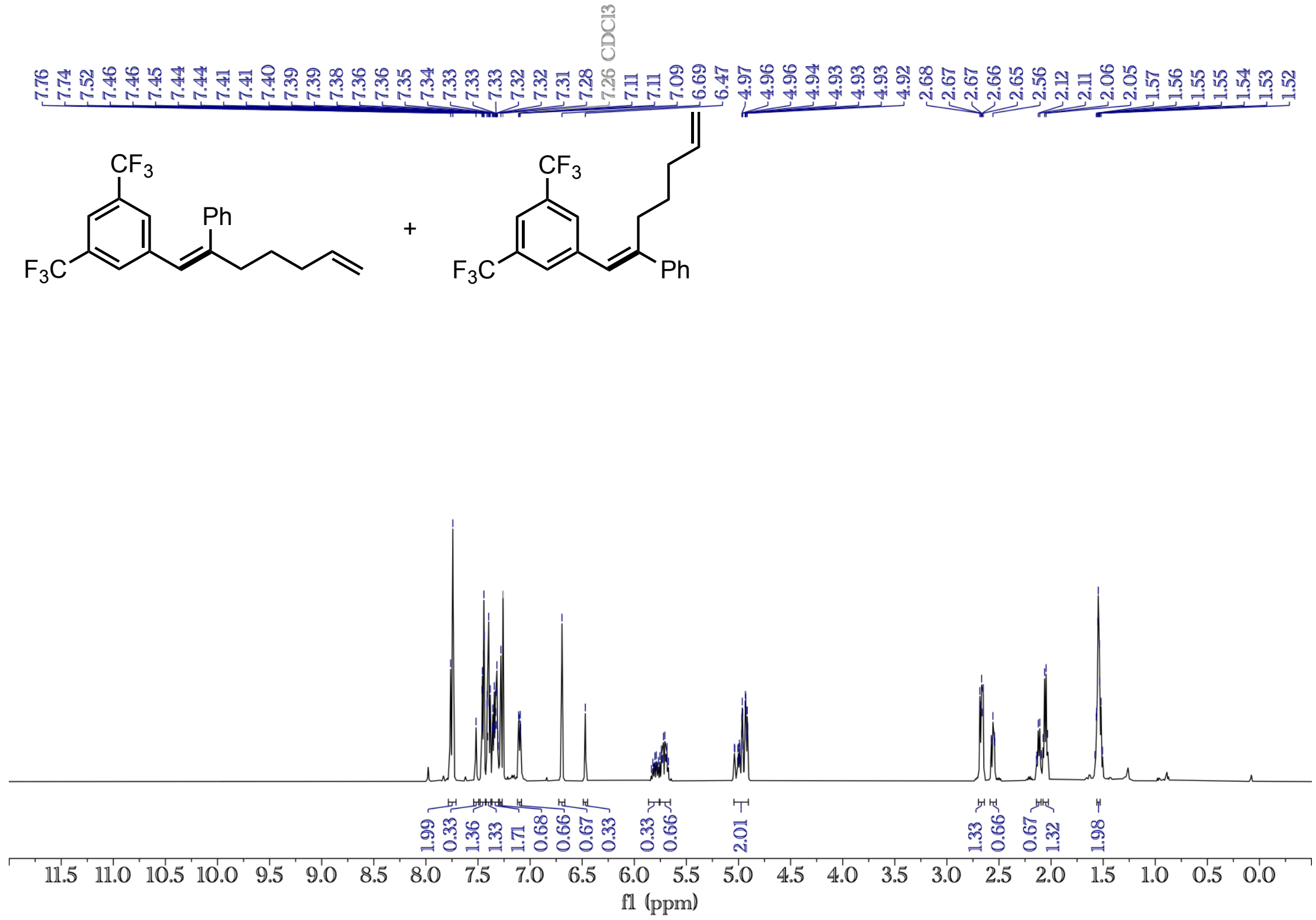

^{13}C {^{1}H} NMR of (*E*/*Z*)-1-(2-phenylhepta-1,6-dien-1-yl)-3,5-bis(trifluoromethyl)benzene (P38)

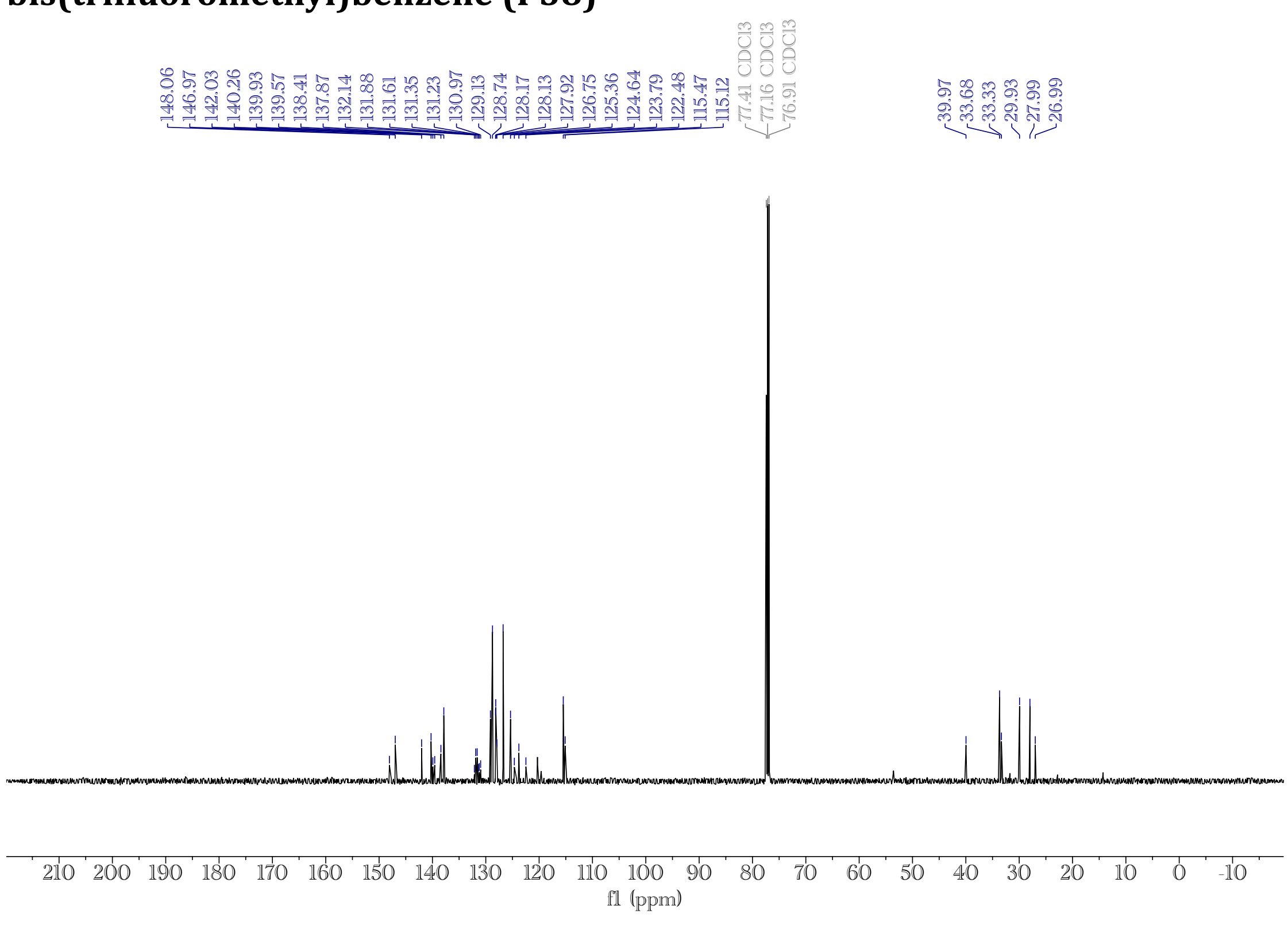

^{19}F {^{1}H} NMR of (*E*/*Z*)-1-(2-phenylhepta-1,6-dien-1-yl)-3,5-bis(trifluoromethyl)benzene (P38)

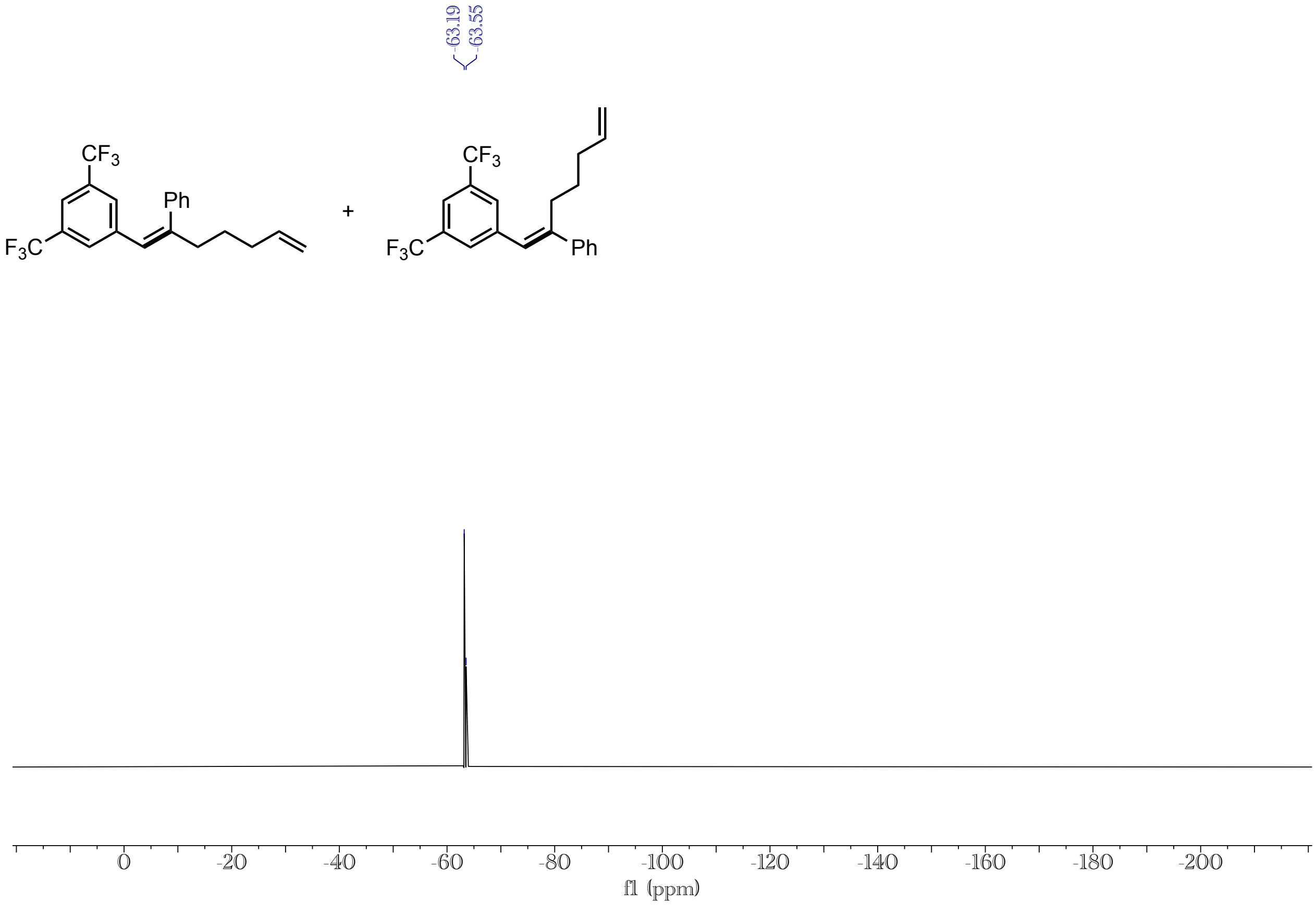

^{1}H NMR of (*Z*)-1-(4-(2-(1,2,3,6-tetrahydro-[1,1'-biphenyl]-4-yl)vinyl)phenyl)propan-2-one (*Z*-P39)

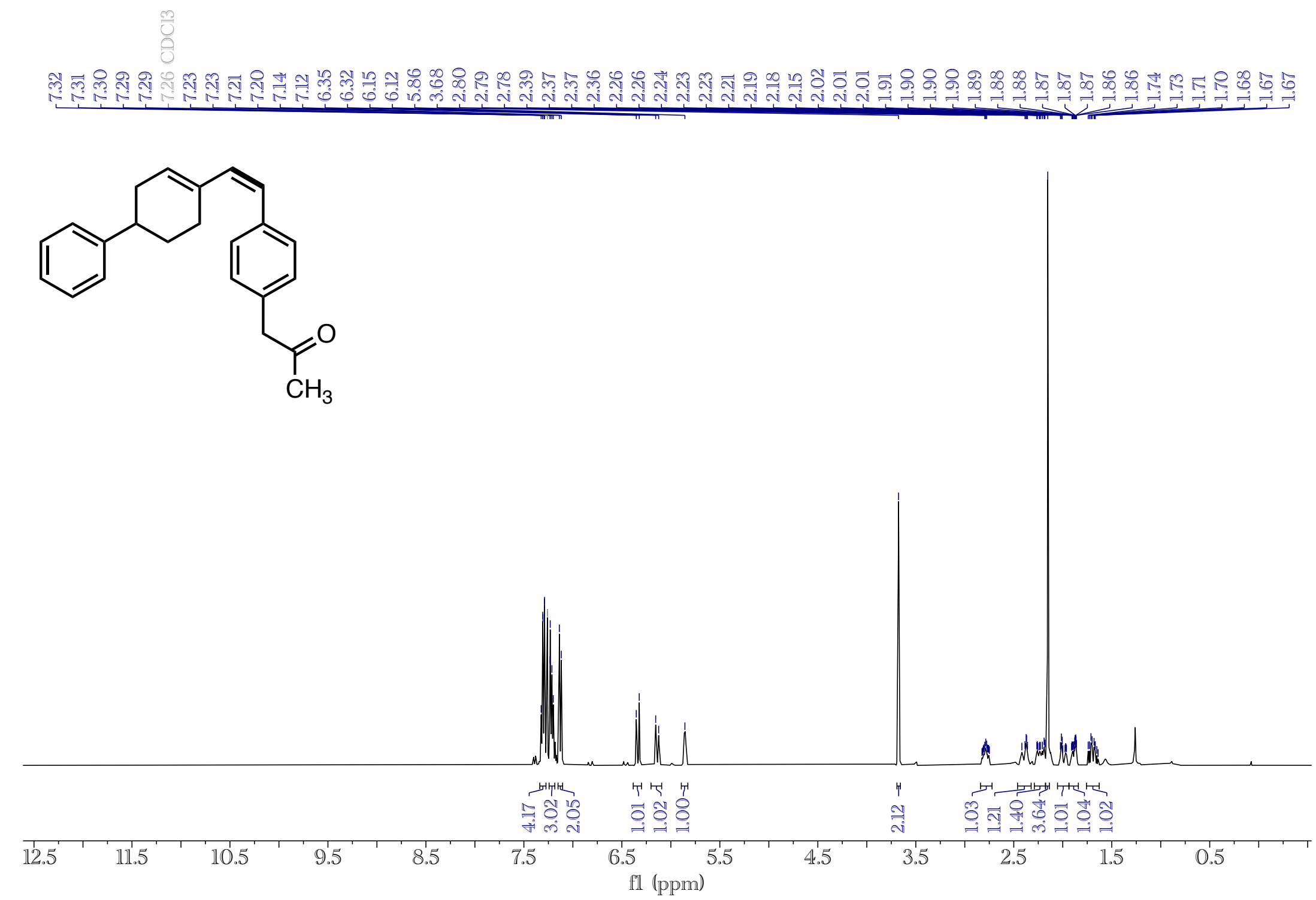

^{13}C {^{1}H} NMR of (*Z*)-1-(4-(2-(1,2,3,6-tetrahydro-[1,1'-biphenyl]-4-yl)vinyl)phenyl)propan-2-one (*Z*-P39)

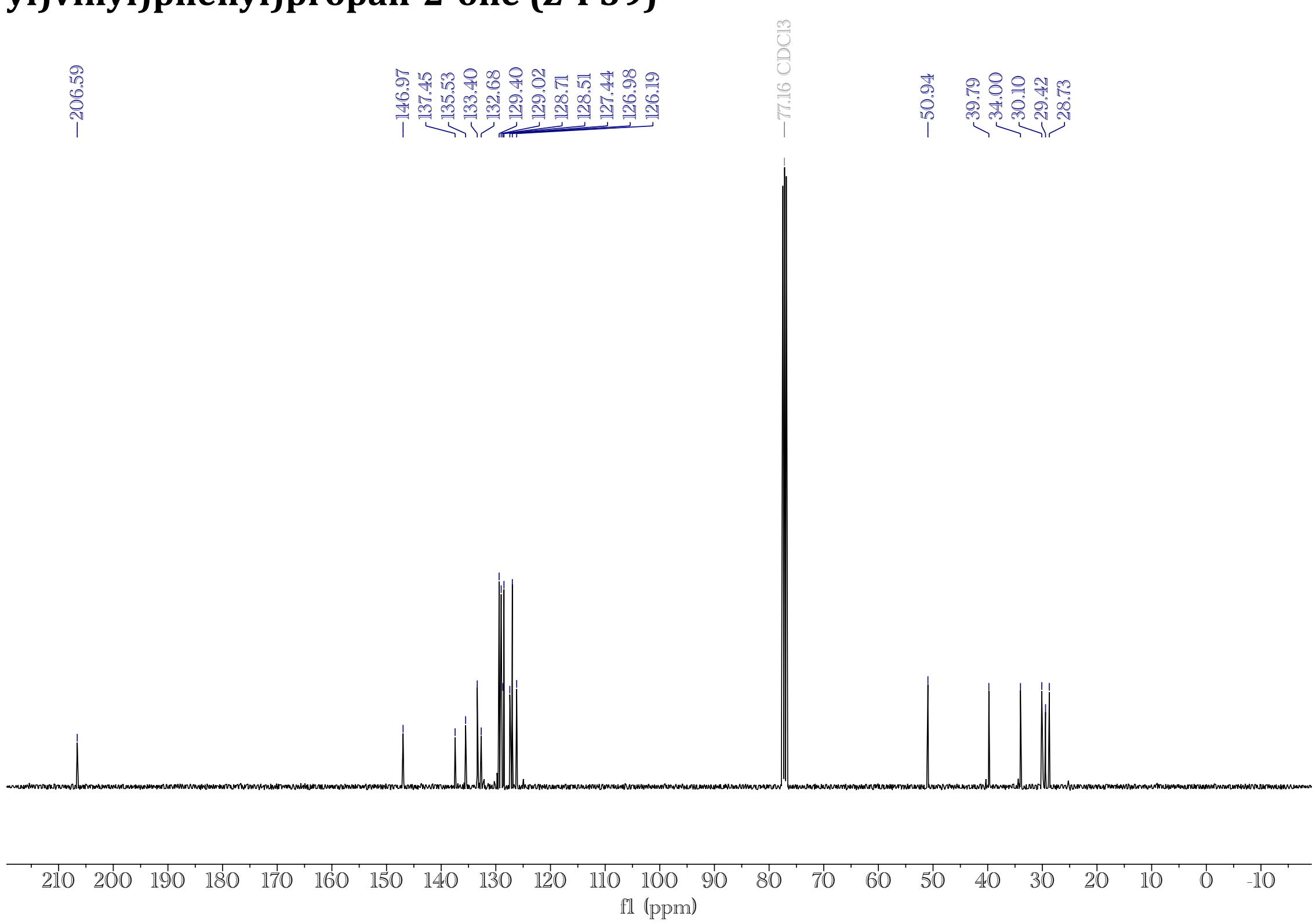

^{1}H NMR of (*E*)-1-(4-(2-(1,2,3,6-tetrahydro-[1,1'-biphenyl]-4-yl)vinyl)phenyl)propan-2-one (*E*-P39)

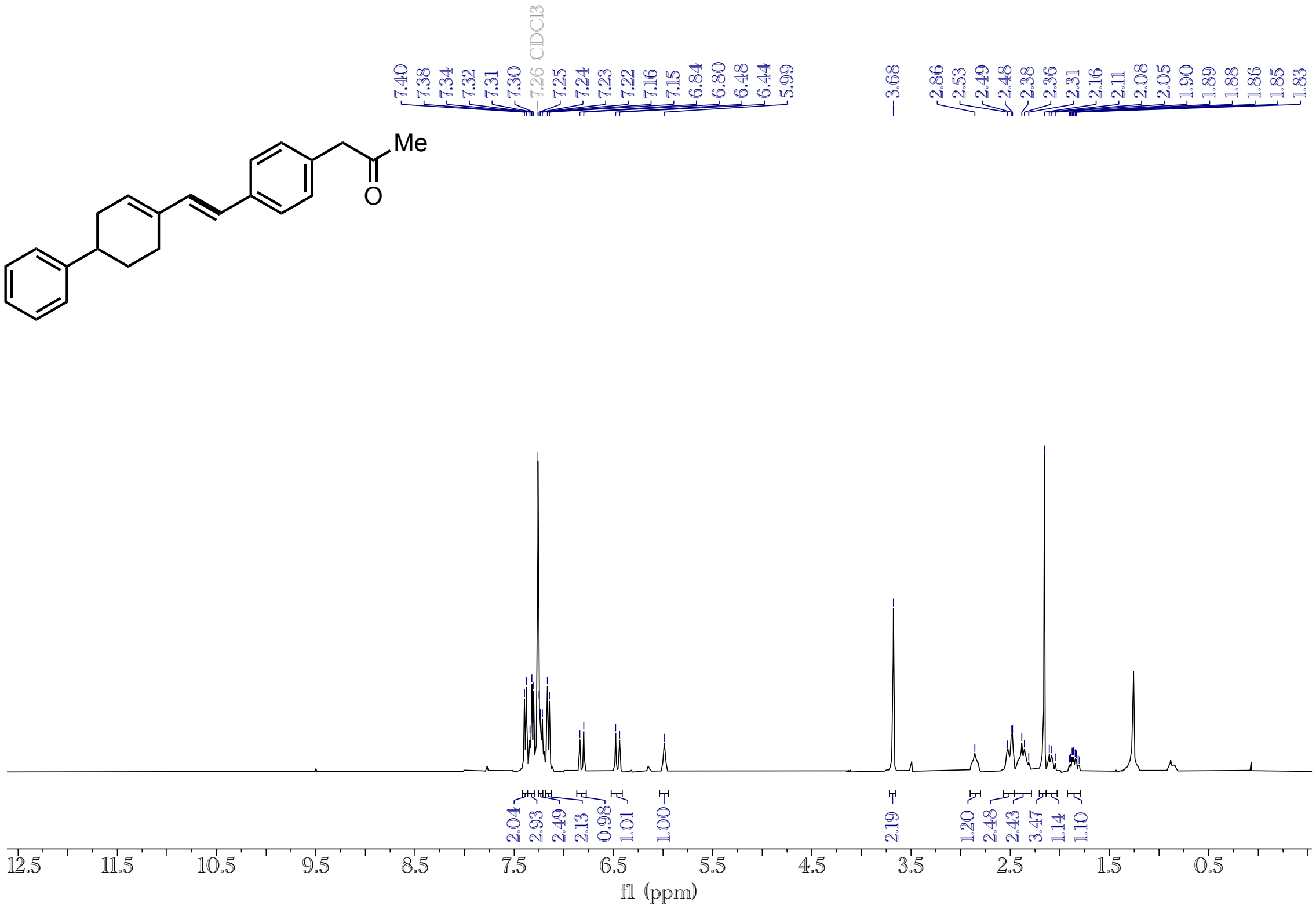

^{13}C NMR of (*E*)-1-(4-(2-(1,2,3,6-tetrahydro-[1,1'-biphenyl]-4-yl)vinyl)phenyl)propan-2-one (*E*-P39)

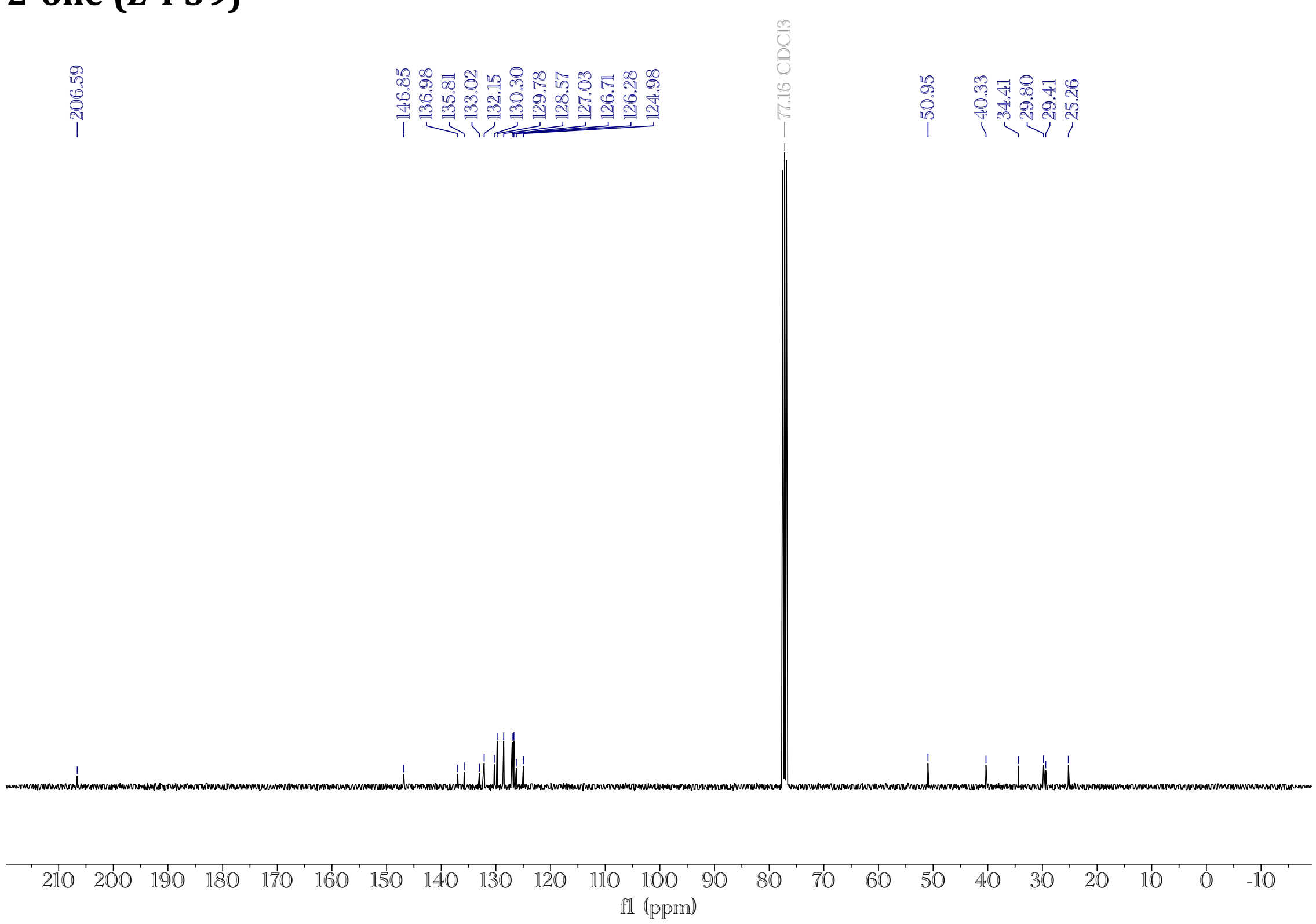

^{1}H NMR of (*E*)-2-(3-(1,2,3,6-tetrahydro-[1,1'-biphenyl]-4-yl)allyl)-1,3-dioxolane (P41)

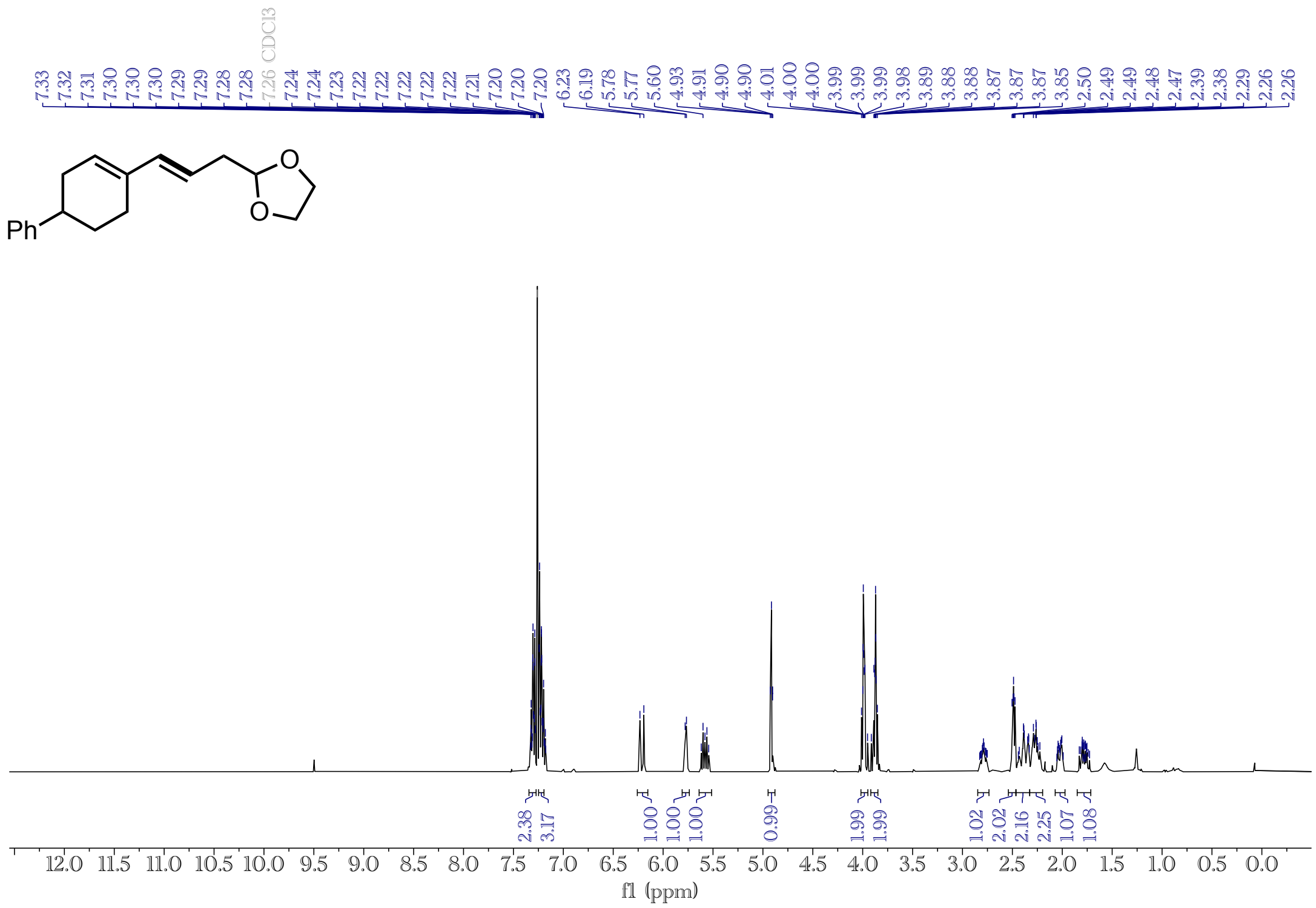

^{13}C {^{1}H} NMR of (*E*)-2-(3-(1,2,3,6-tetrahydro-[1,1'-biphenyl]-4-yl)allyl)-1,3-dioxolane (P41)

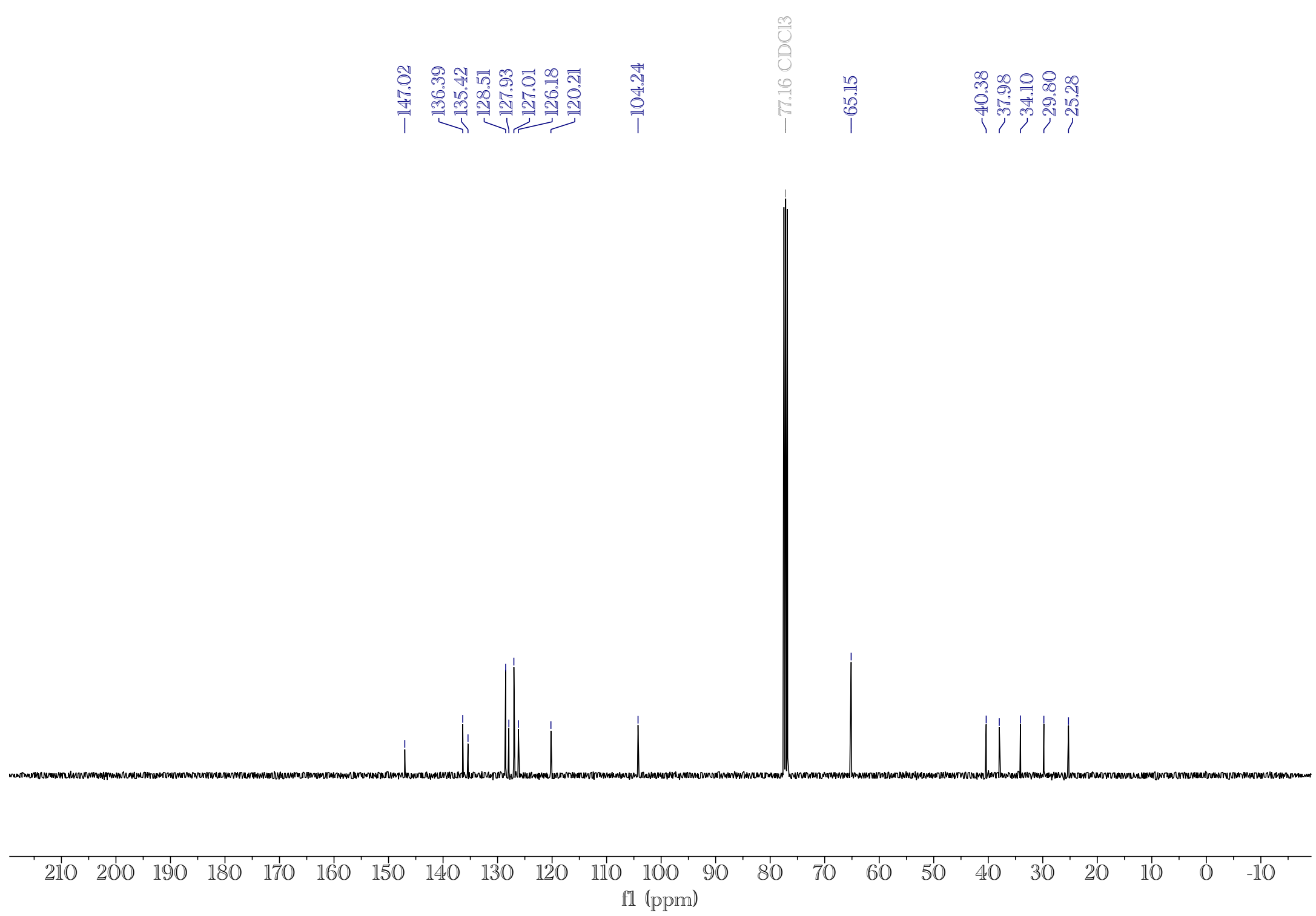

^{1}H NMR of (*E*)-1,2-diphenylethene (P42)

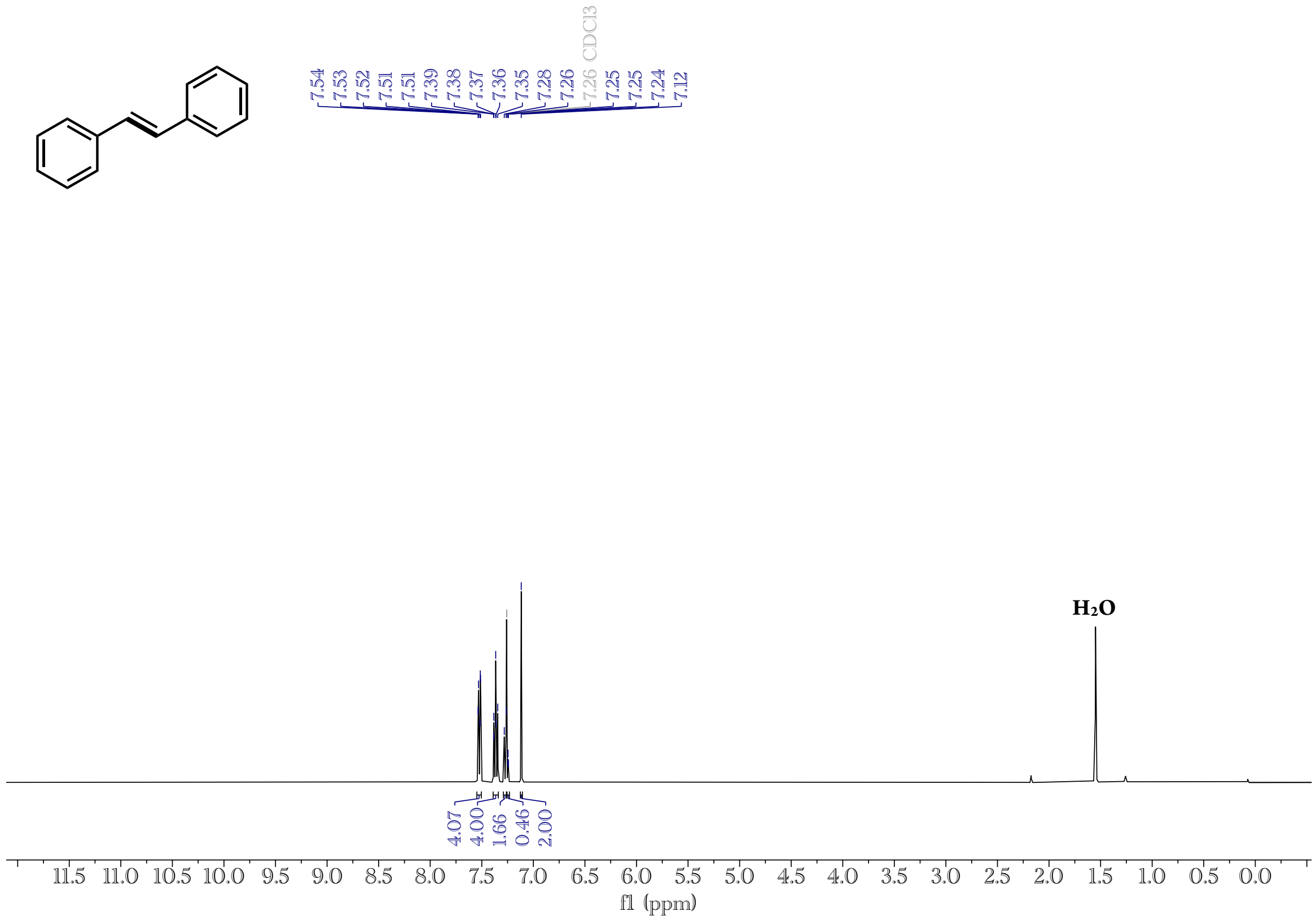

^{13}C {^{1}H} NMR of (*E*)-1,2-diphenylethene (P42)

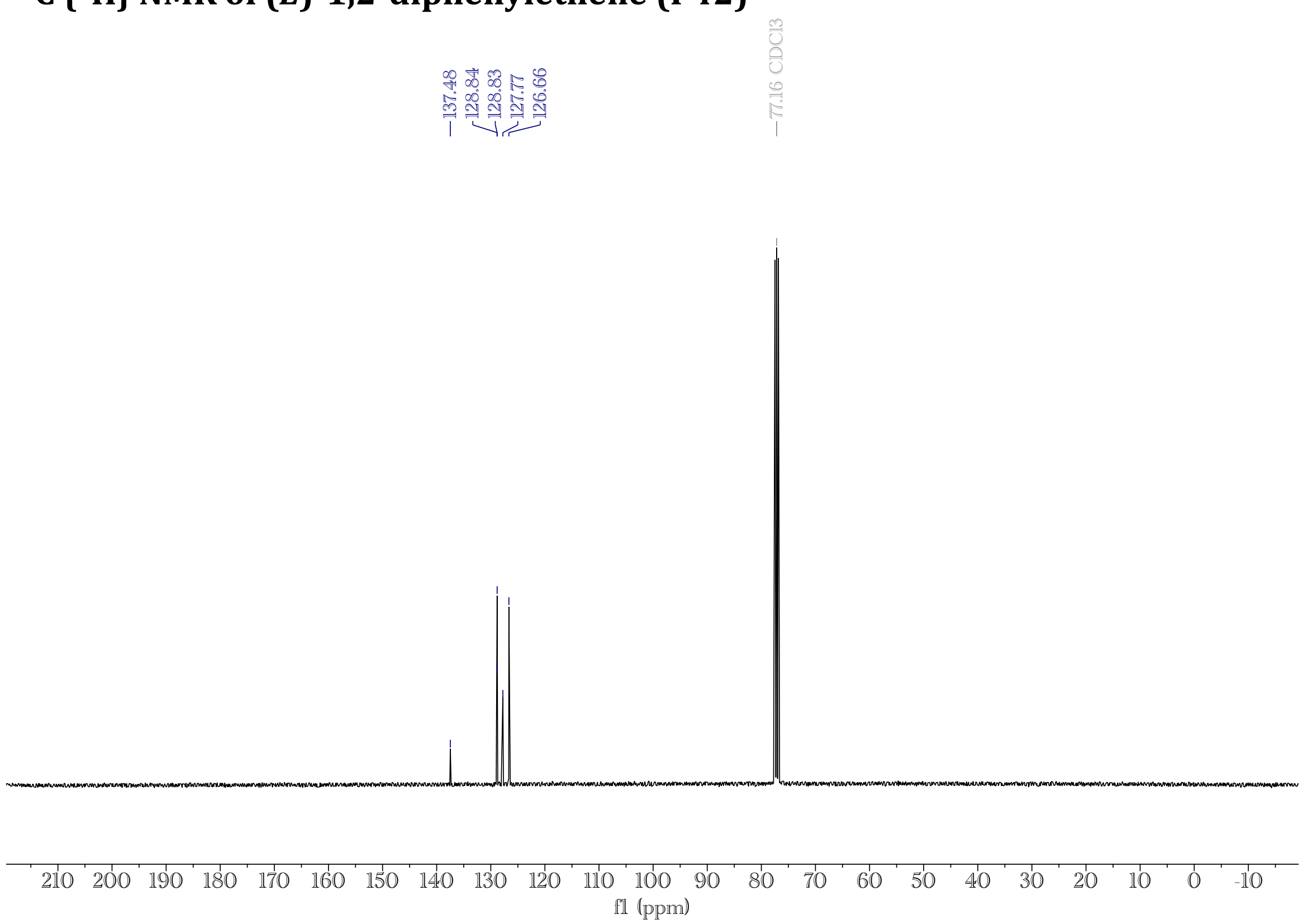

^{1}H NMR of (*E*)-4,4'-(ethene-1,2-diyl)dibenzaldehyde (P43)

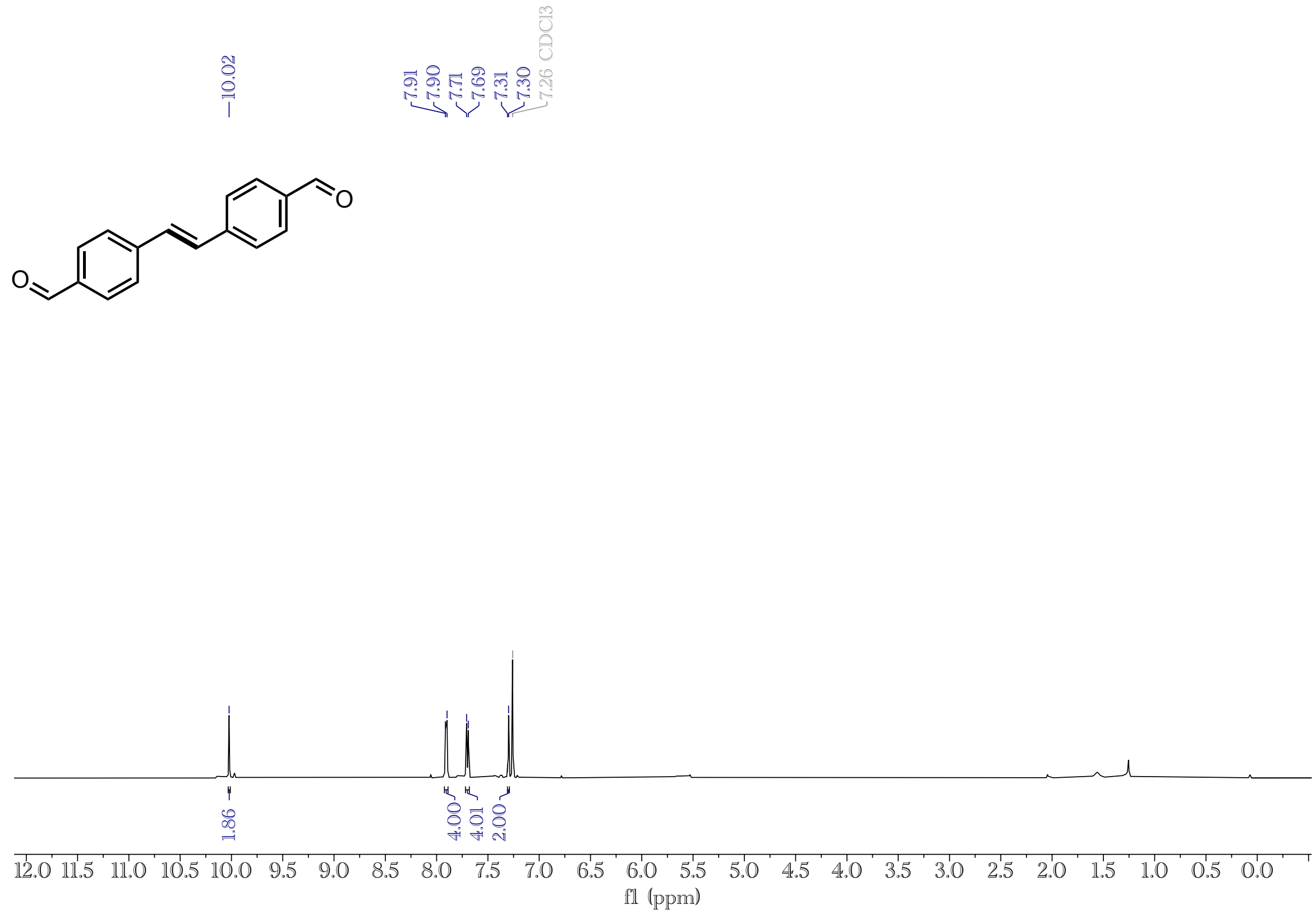

^{13}C {^{1}H} NMR of (*E*)-4,4'-(ethene-1,2-diyl)dibenzaldehyde (P43)

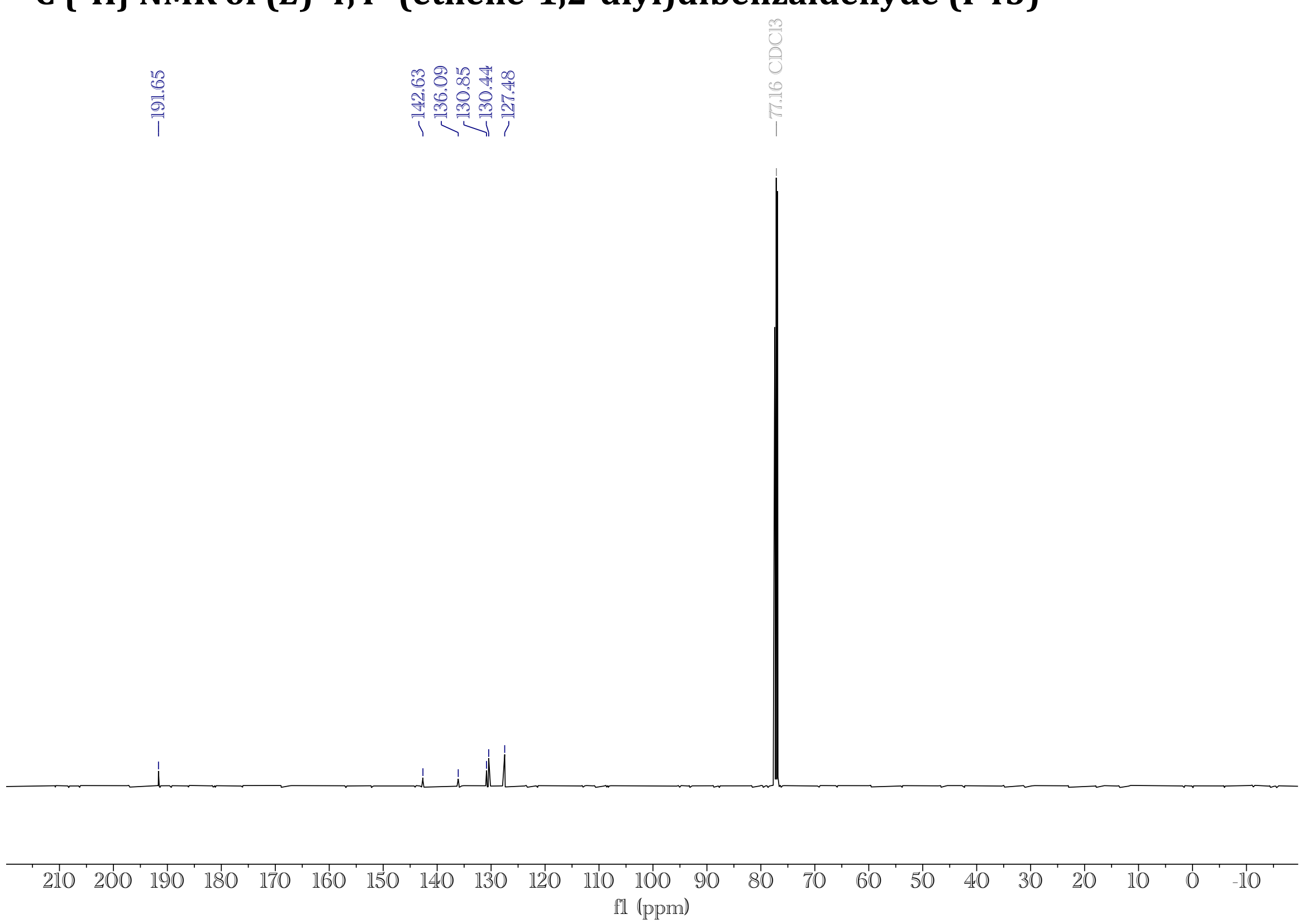

¹H NMR of (*E*/*Z*)-1,2-di(cyclohex-1-en-1-yl)ethene (P44)

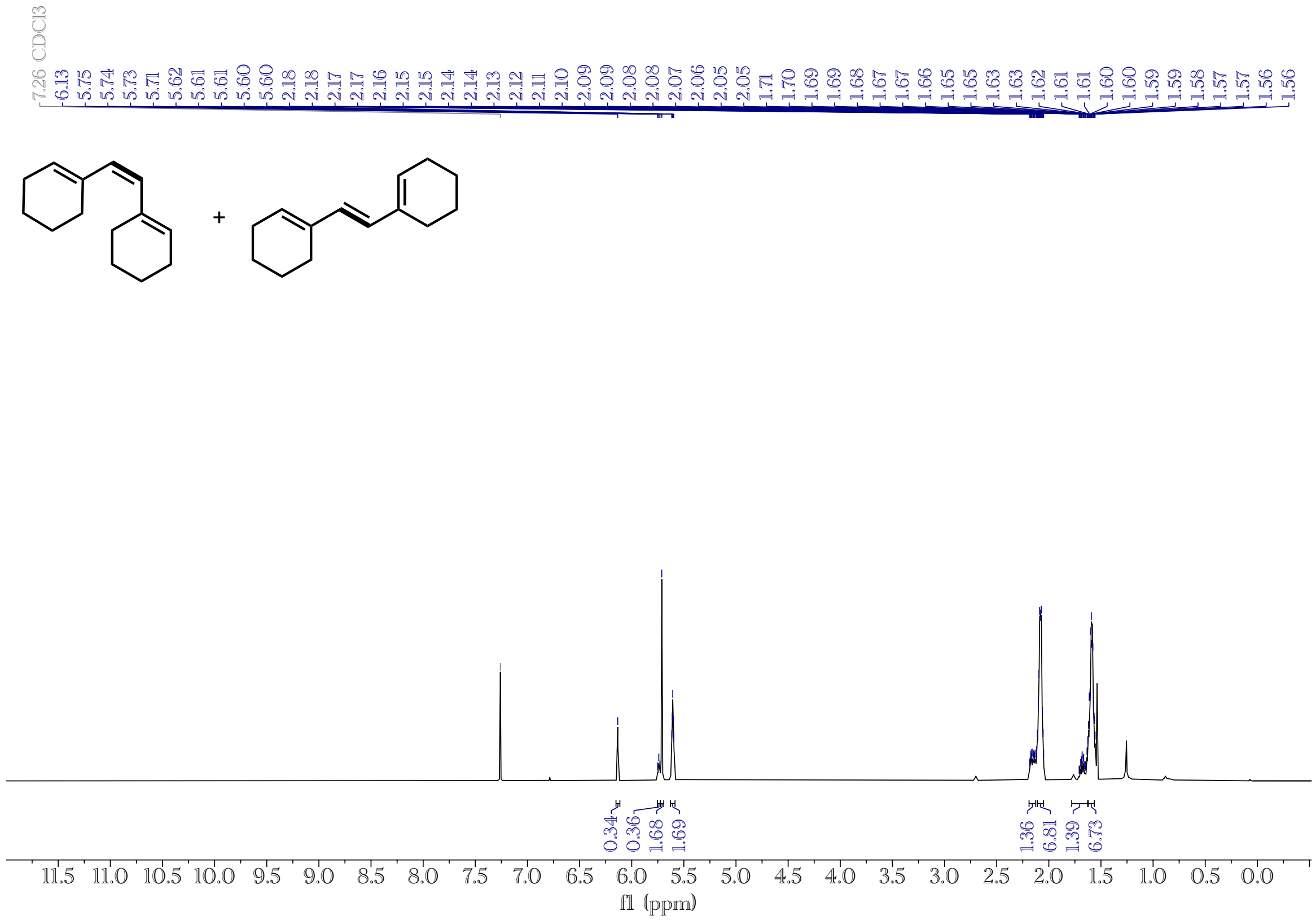

¹³C {¹H} NMR of (*E*/*Z*)-1,2-di(cyclohex-1-en-1-yl)ethene (P44)

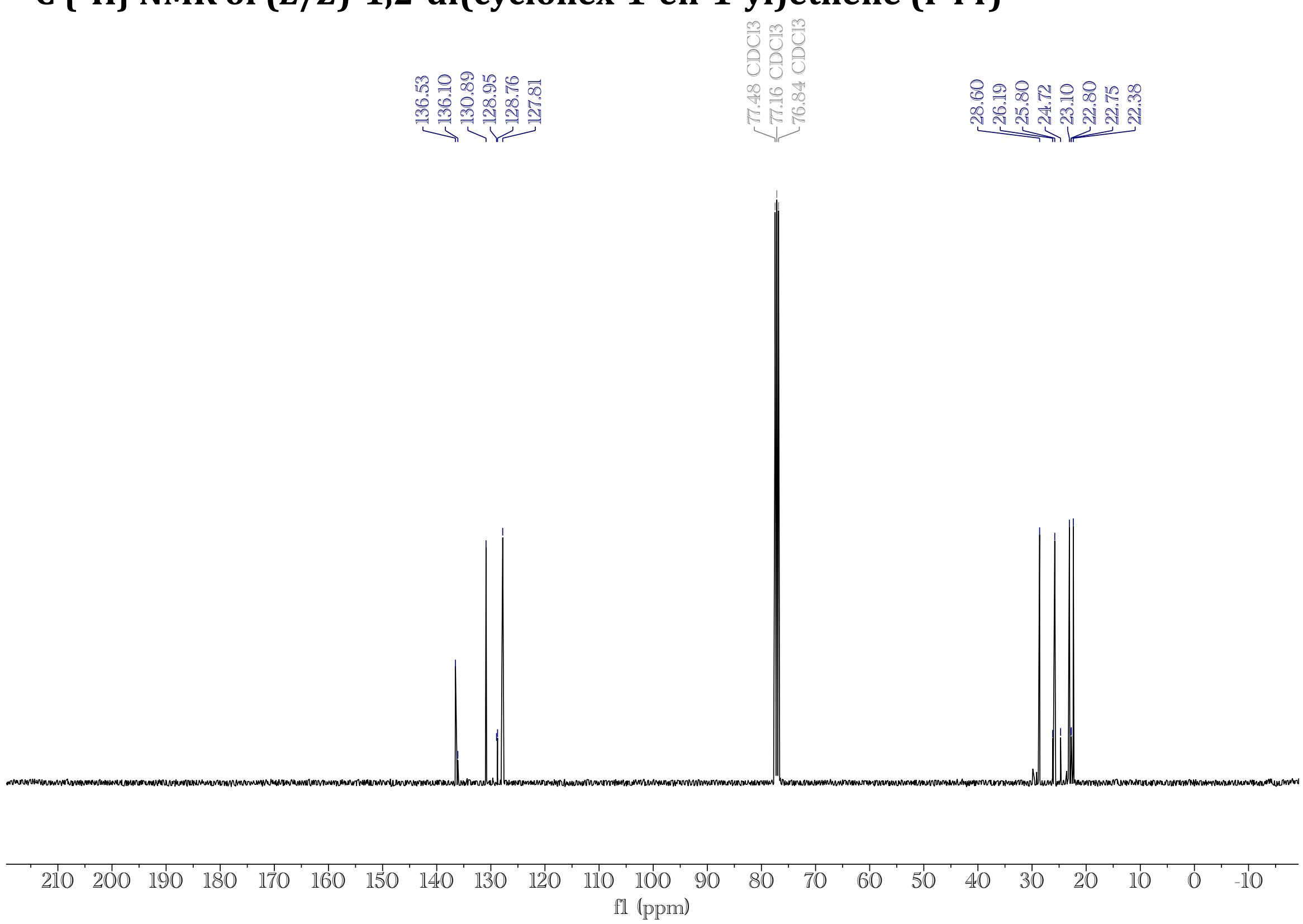

^{1}H NMR of 2,2',3,3',5,5',6,6'-octahydro-4,4'-bipyranylidene (P45)

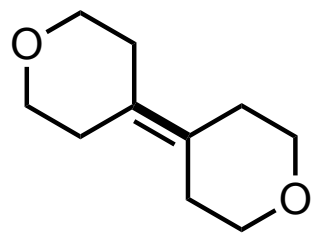

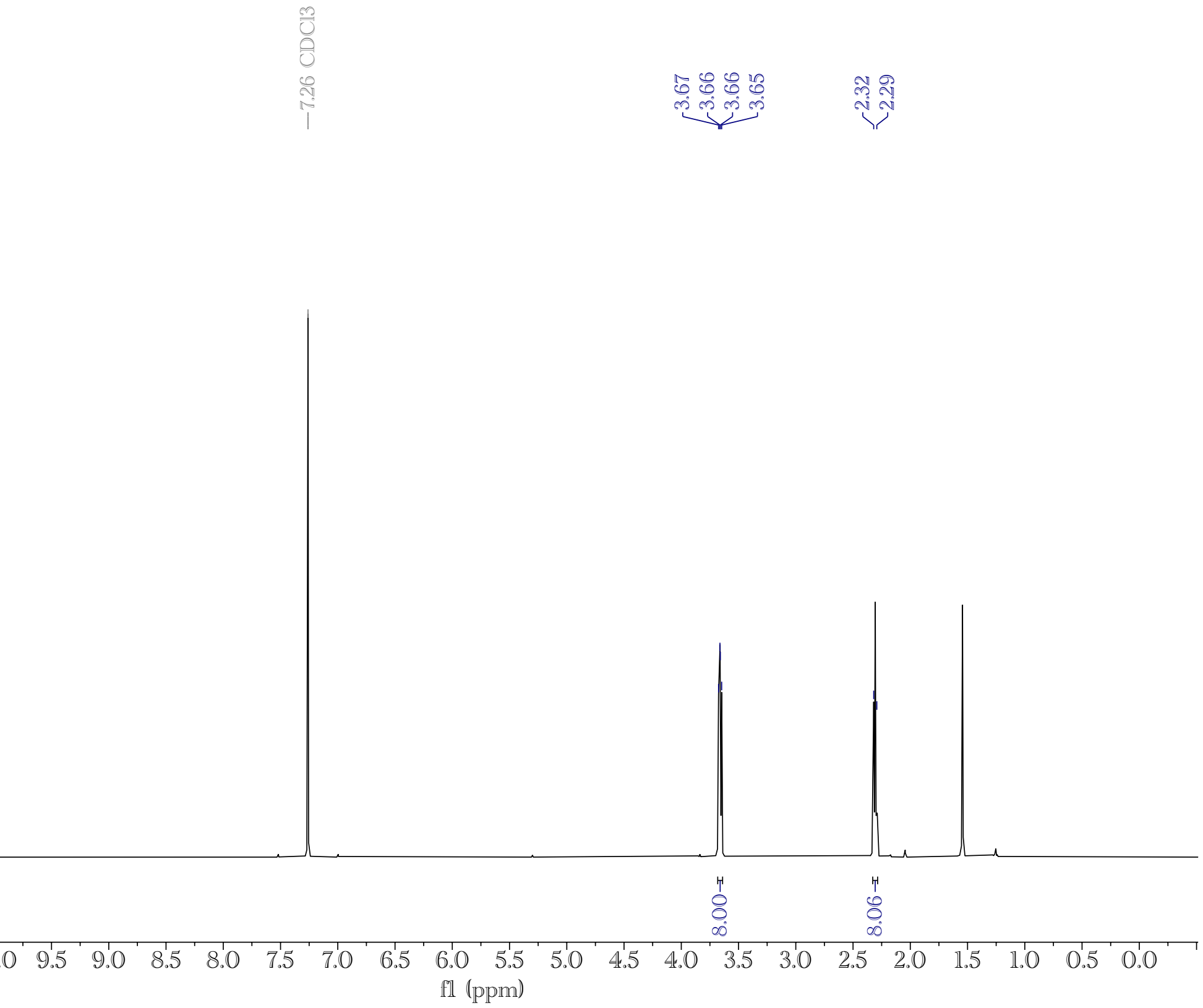

^{13}C {^{1}H} NMR of 2,2',3,3',5,5',6,6'-octahydro-4,4'-bipyranylidene (P45)

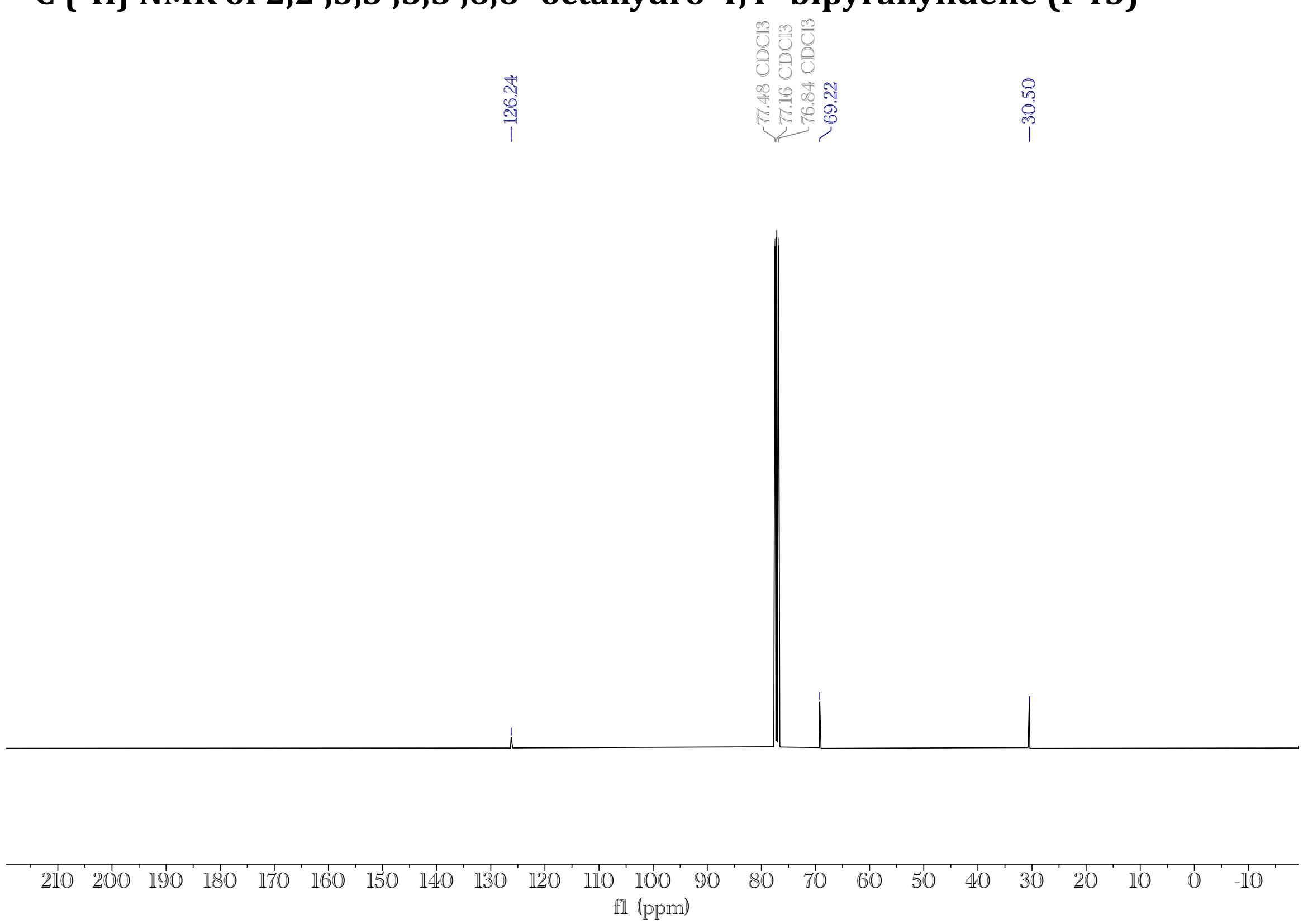

¹H NMR of (*Z*)-1,2-difluoro-1,2-diphenylethene (P46)

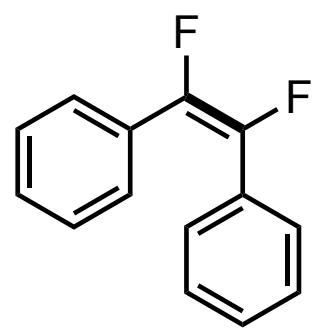

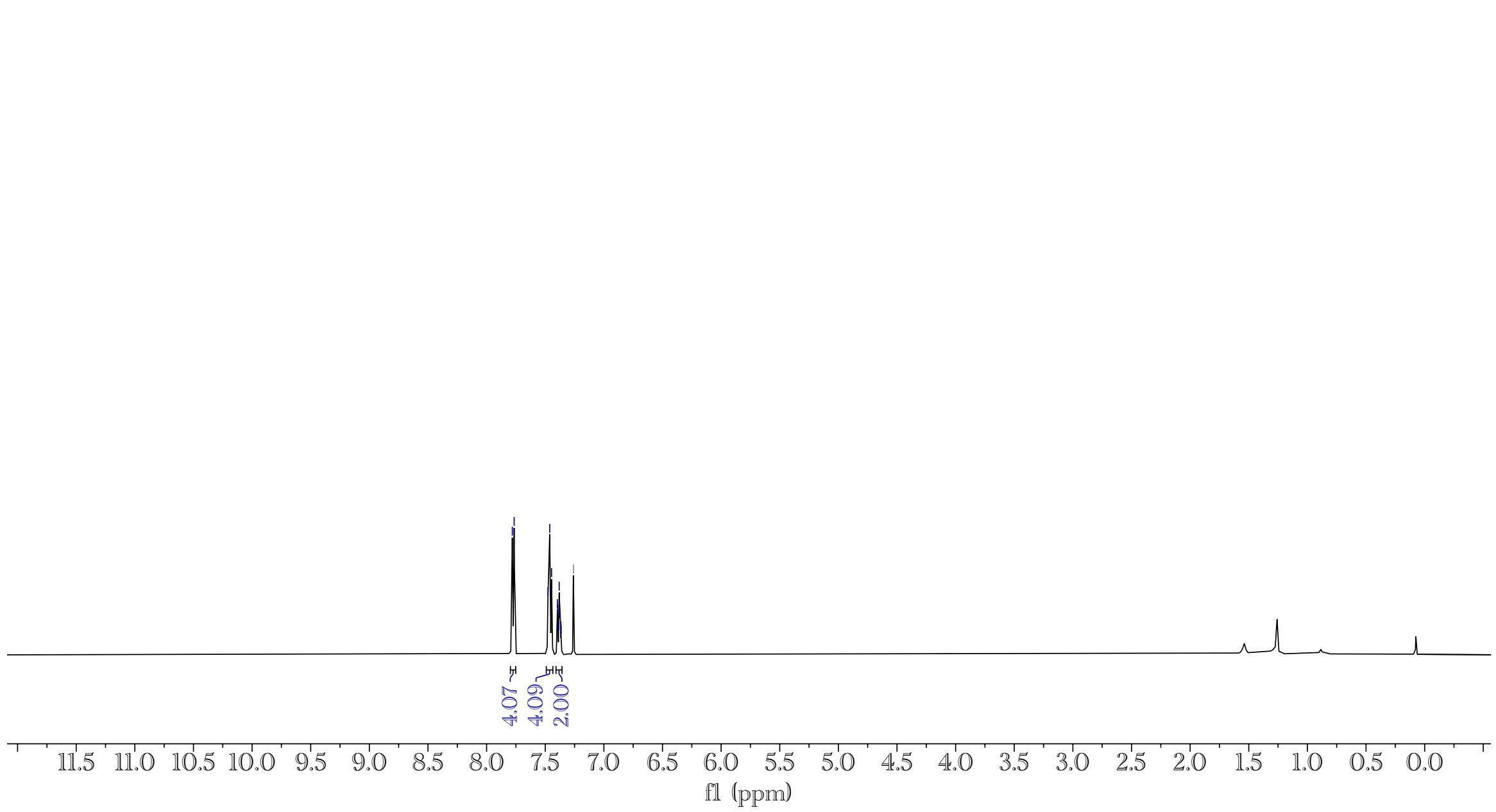

¹³C {¹H} NMR of (*Z*)-1,2-difluoro-1,2-diphenylethene (P46)

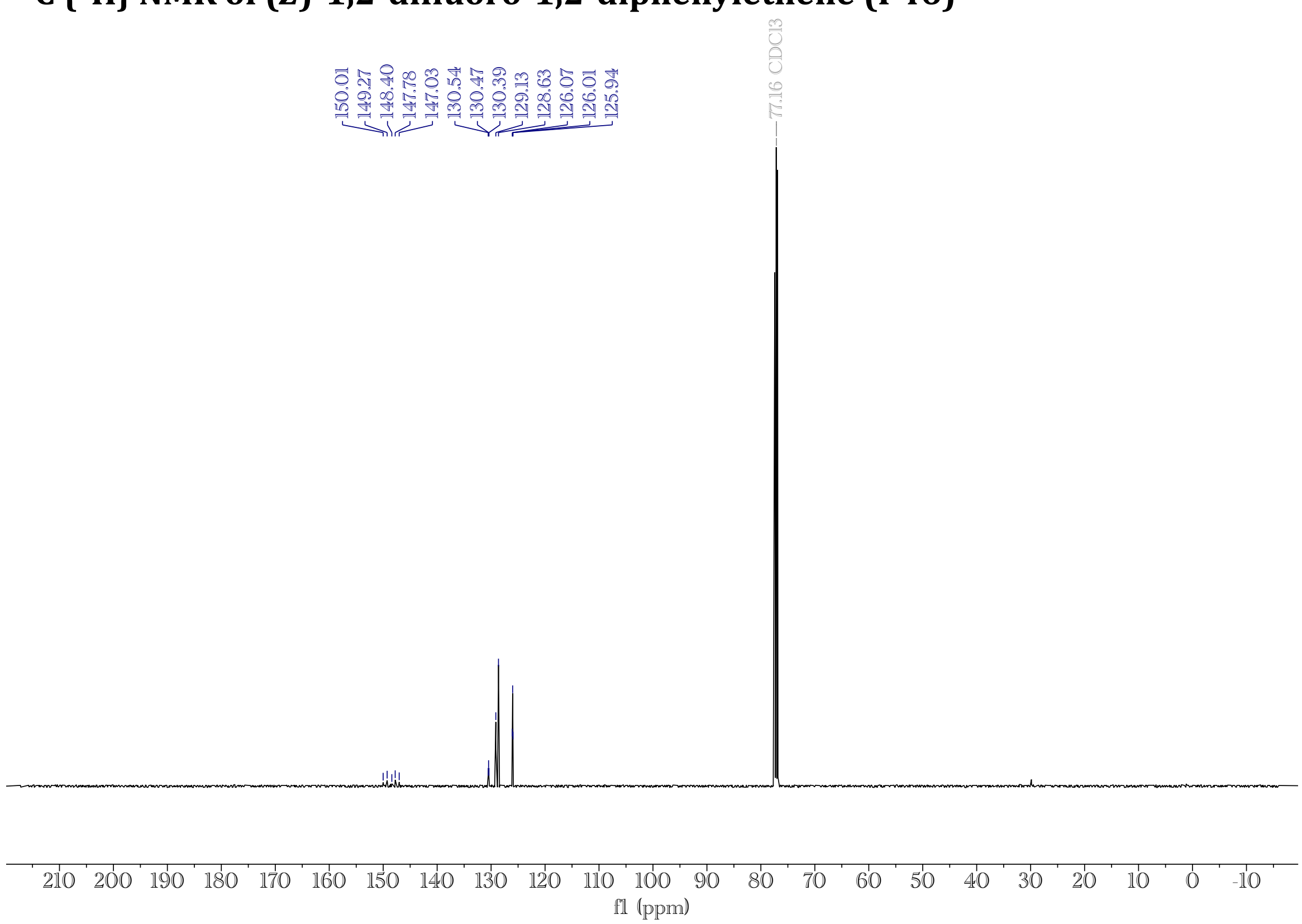

^{19}F {^{1}H} NMR of (*Z*)- 1,2-difluoro-1,2-diphenylethene (P46)

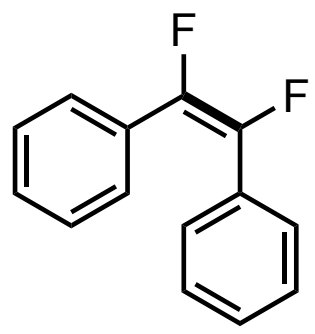

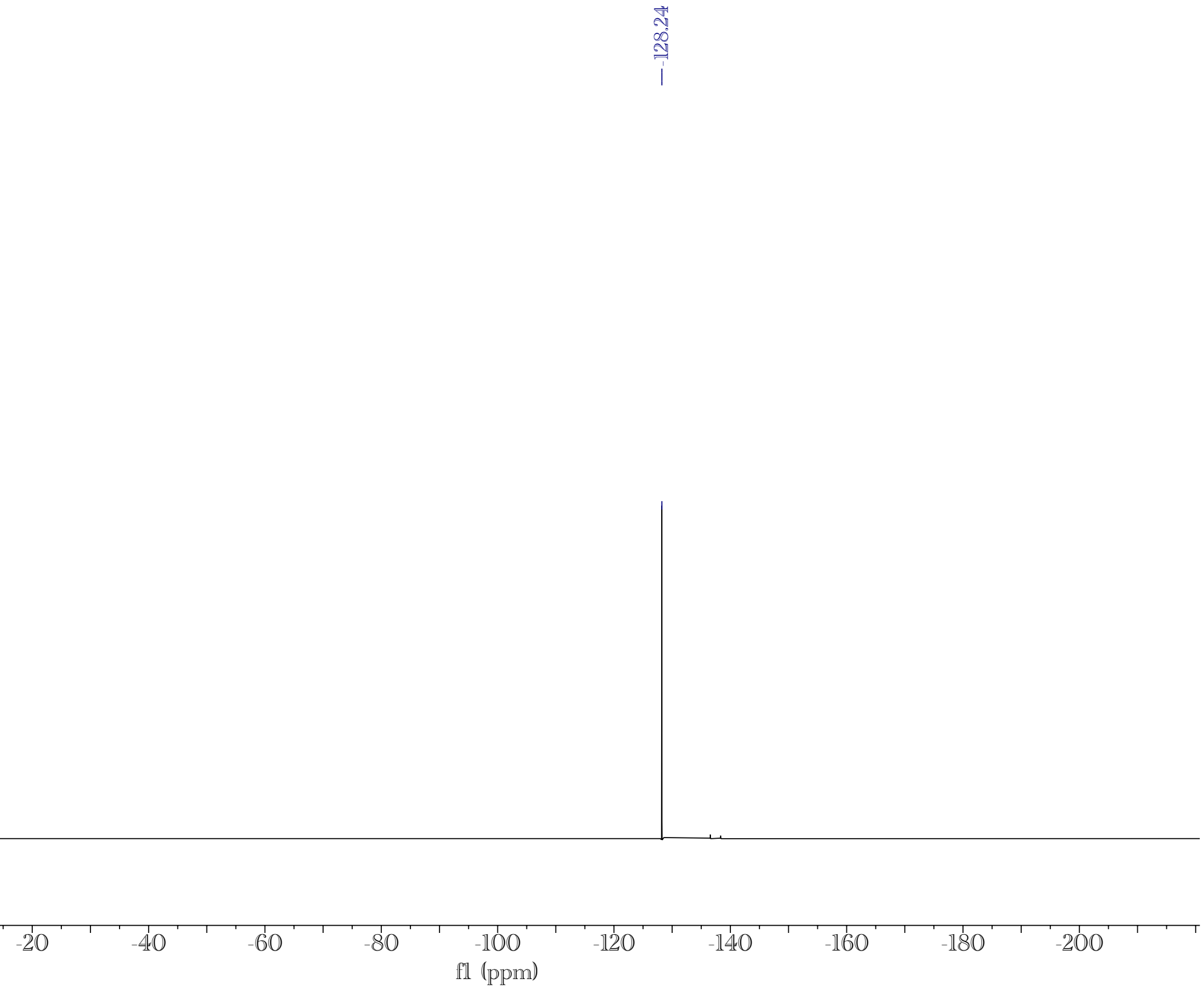

^{1}H NMR (*E*)-1,2-difluoro-1,2-bis(4-(trifluoromethyl)phenyl)ethene (P47)

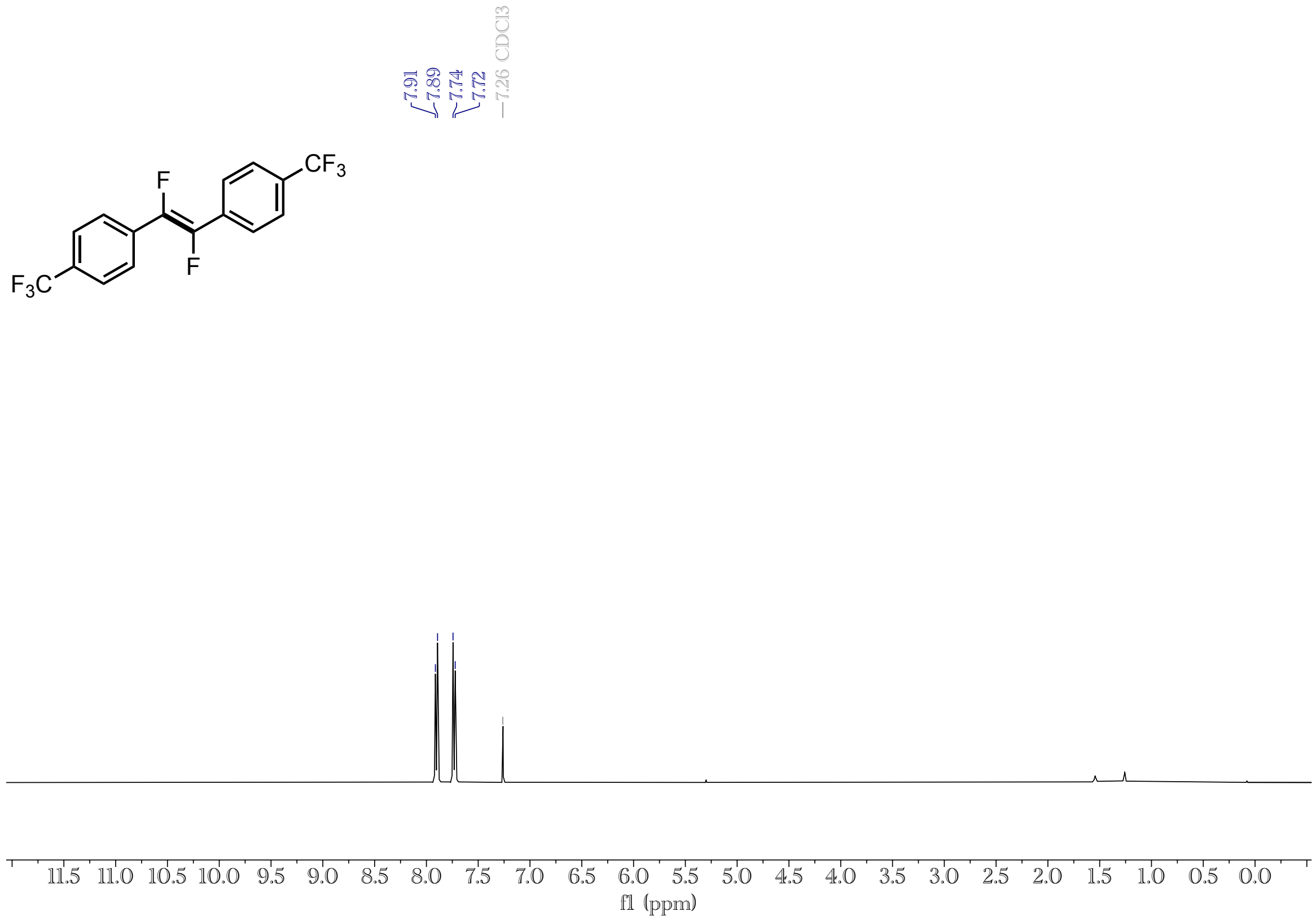

^{13}C {^{1}H} NMR of (*E*)-1,2-difluoro-1,2-bis(4-(trifluoromethyl)phenyl)ethene (P47)

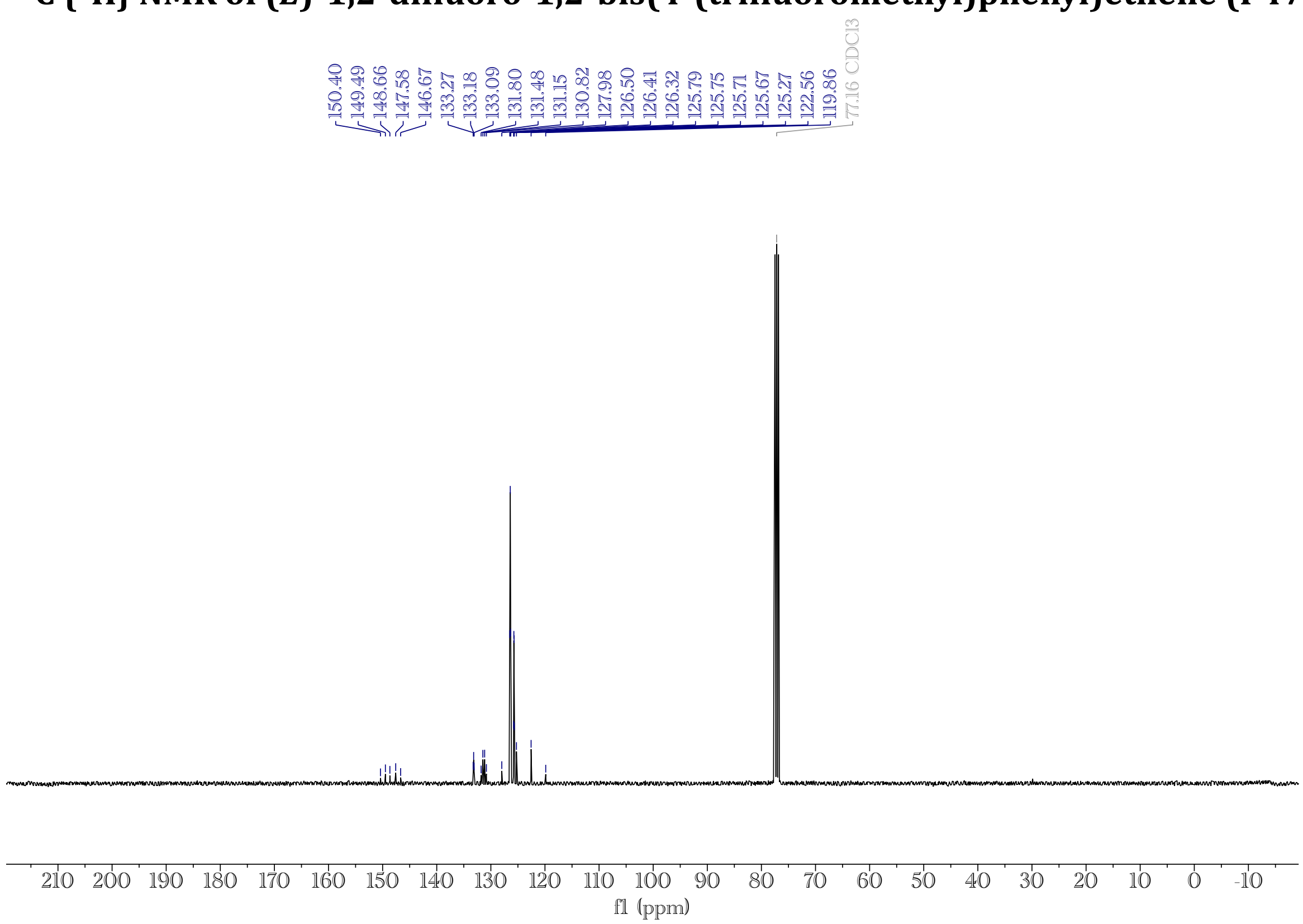

^{19}F {^{1}H} NMR of (*E*)-1,2-difluoro-1,2-bis(4-(trifluoromethyl)phenyl)ethene (P47)

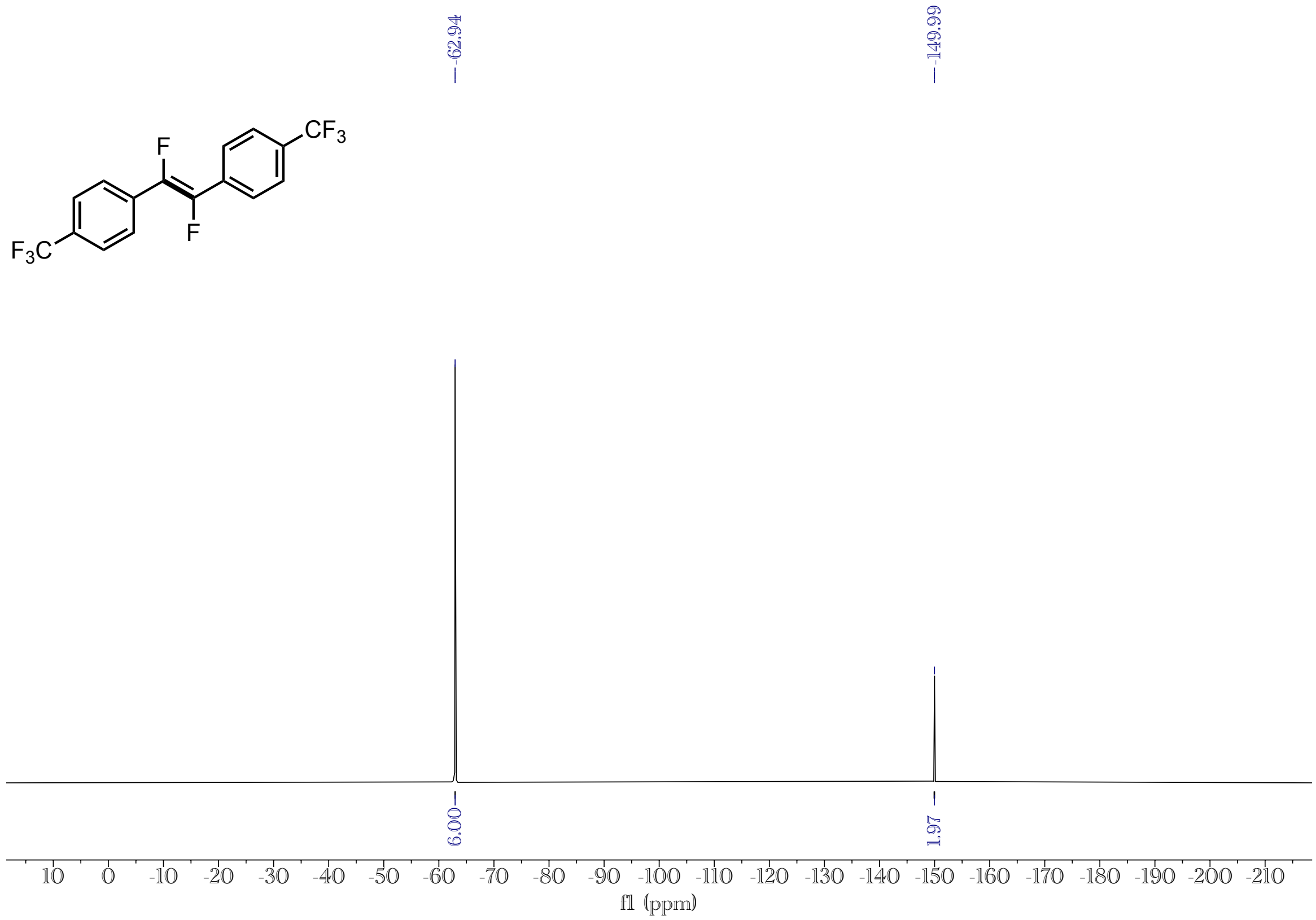

^{1}H NMR dimethyl 4-(fluoro(phenyl)methylene)heptanedioate (P48)

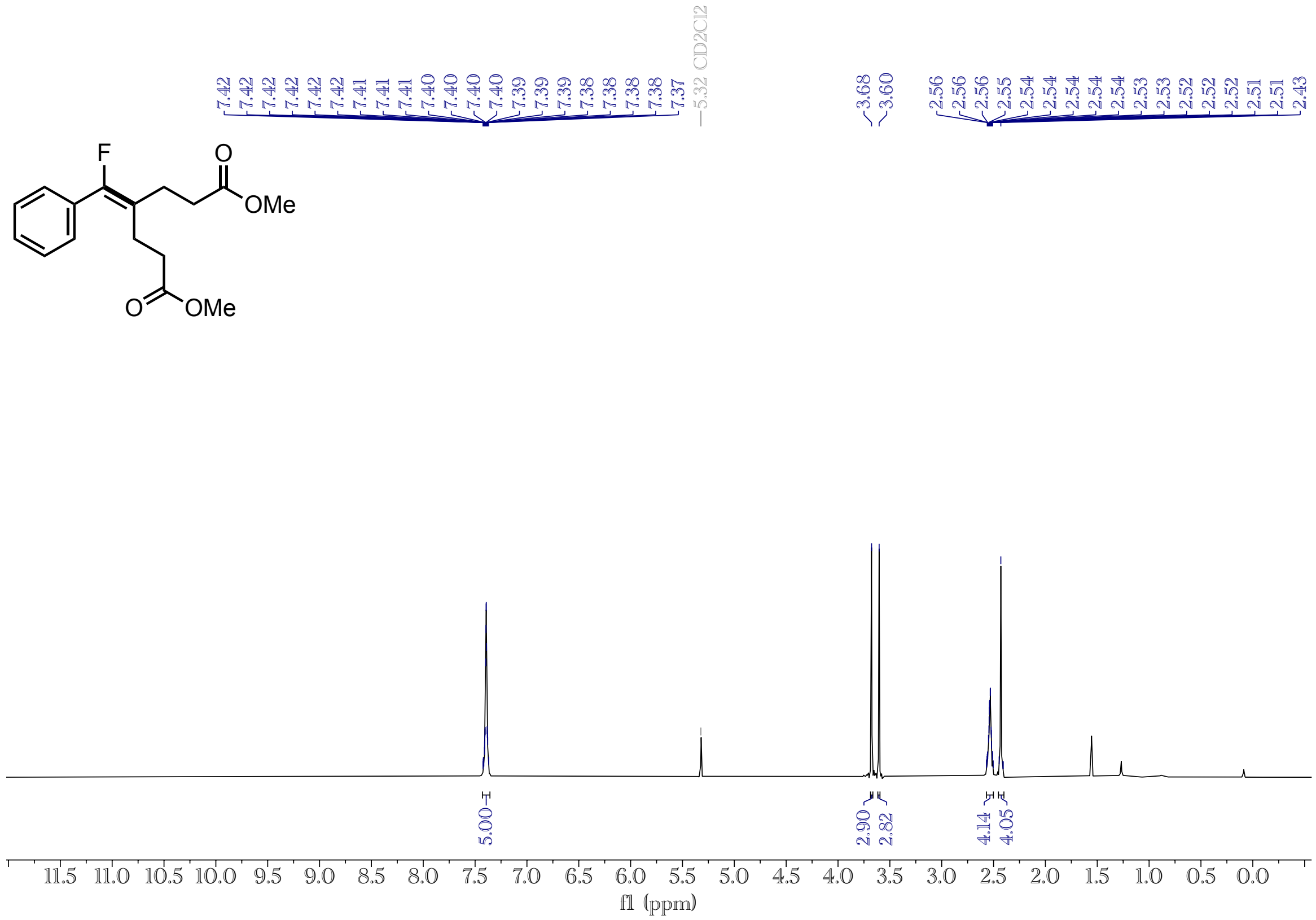

^{13}C {^{1}H} NMR of dimethyl 4-(fluoro(phenyl)methylene)heptanedioate (P48)

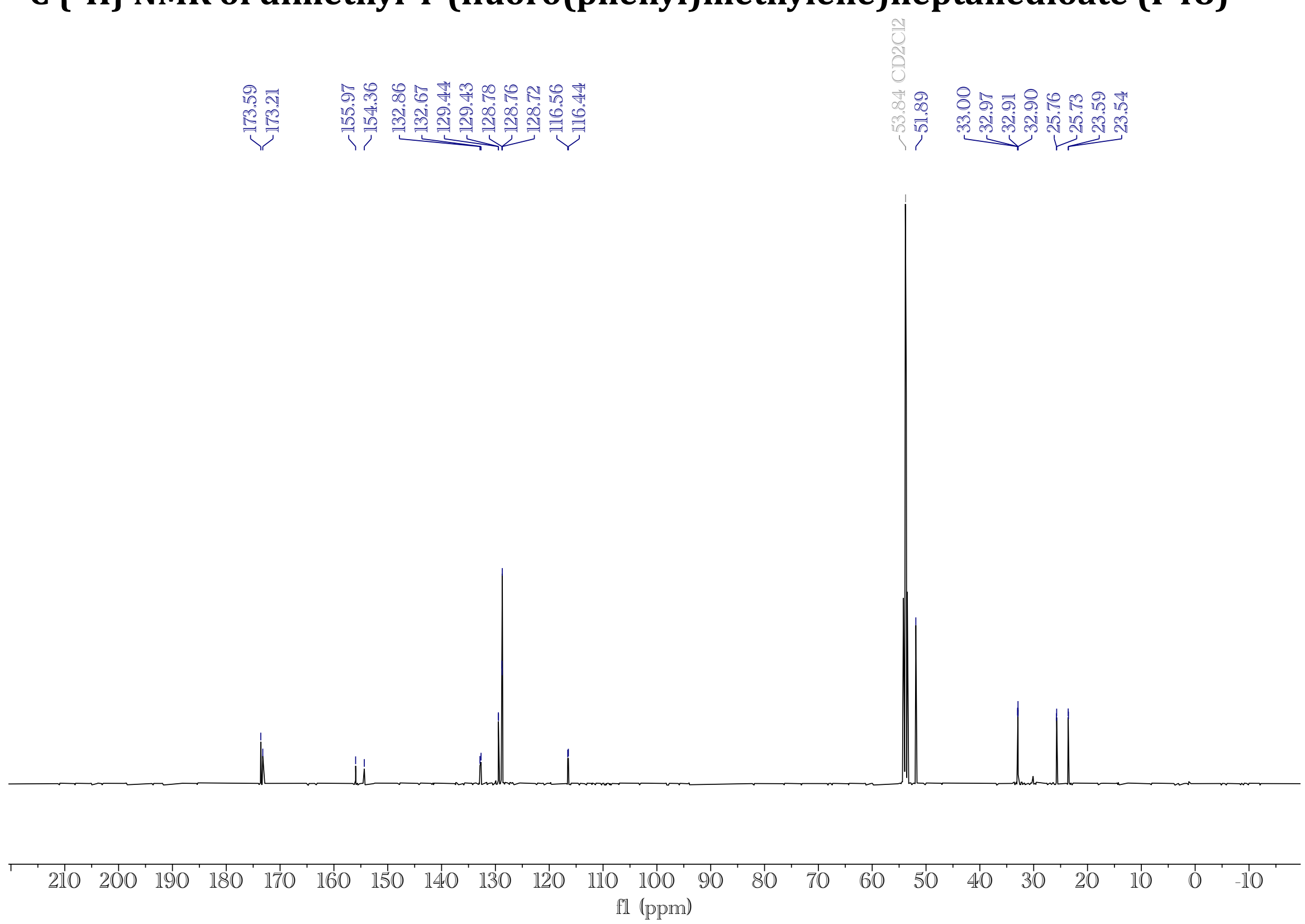

$^{19}F\ \{^{1}H\}$ NMR of dimethyl 4-(fluoro(phenyl)methylene)heptanedioate (P48)

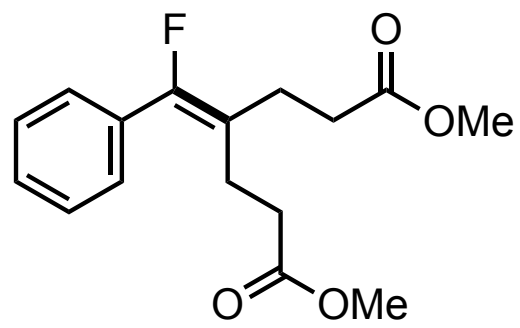

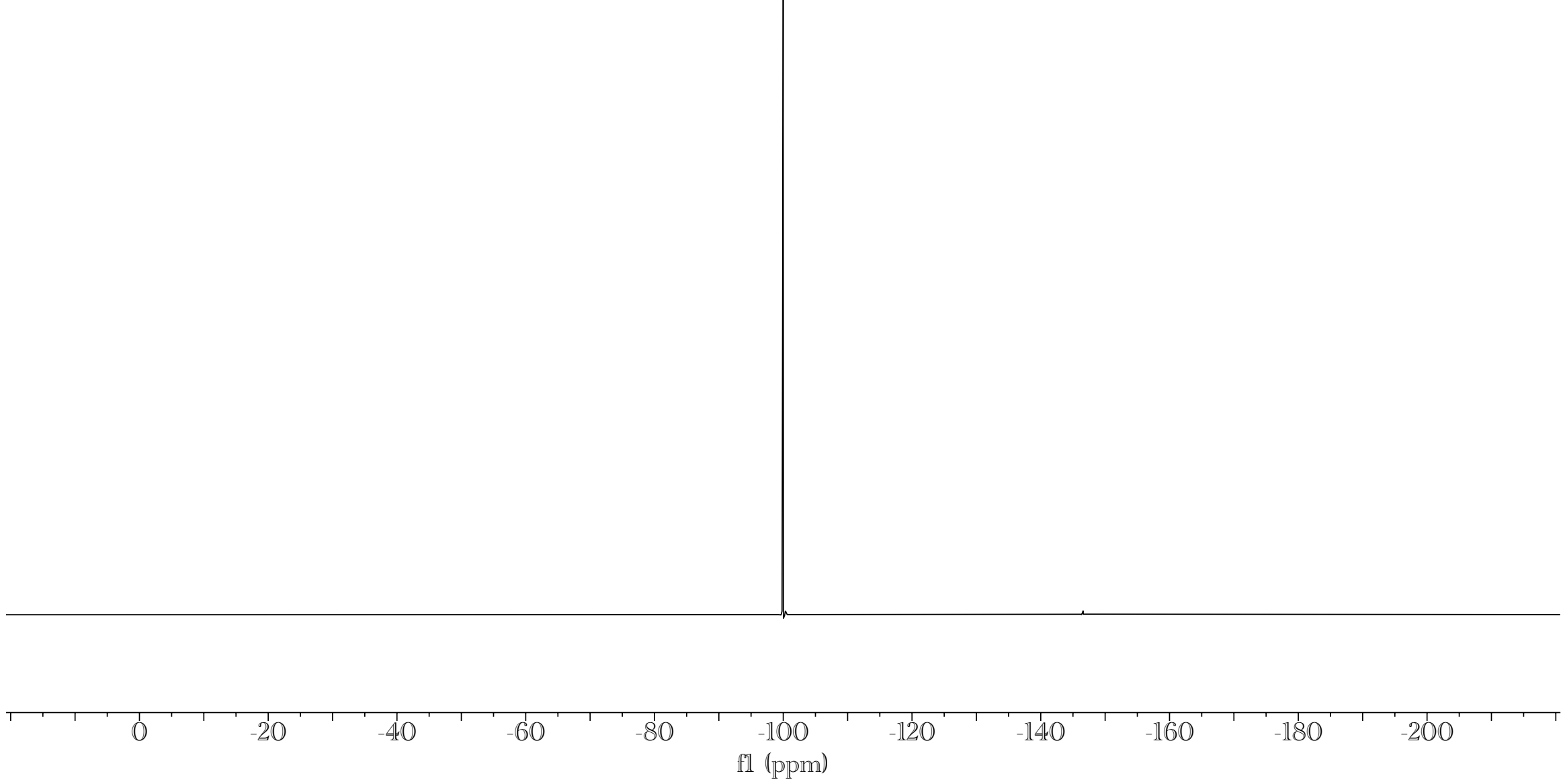

^{1}H NMR (*E*)-4-(2-fluoro-3-phenylprop-1-en-1-yl)benzonitrile (P49)

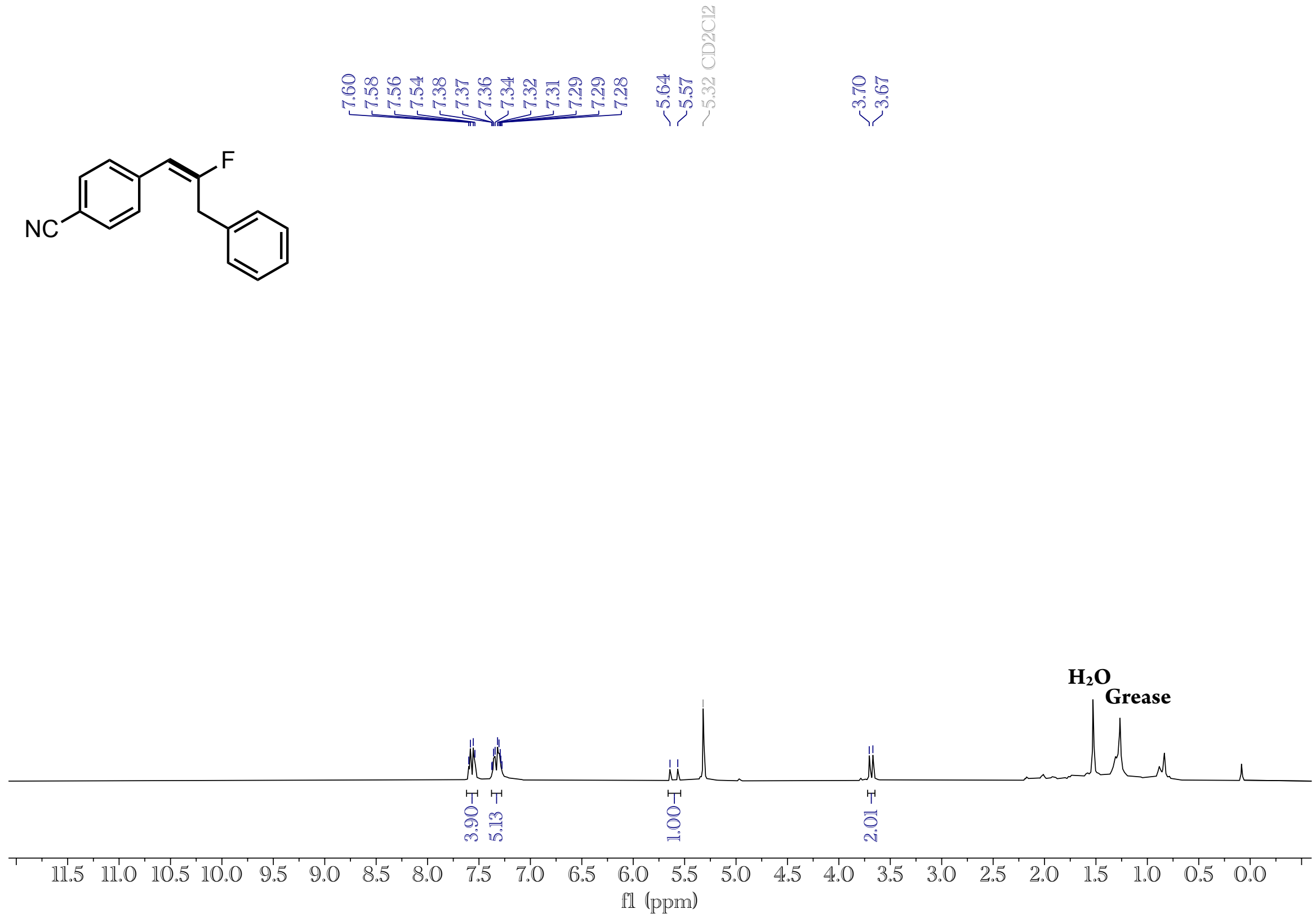

^{13}C {^{1}H} NMR of (*E*)-4-(2-fluoro-3-phenylprop-1-en-1-yl)benzonitrile (P49)

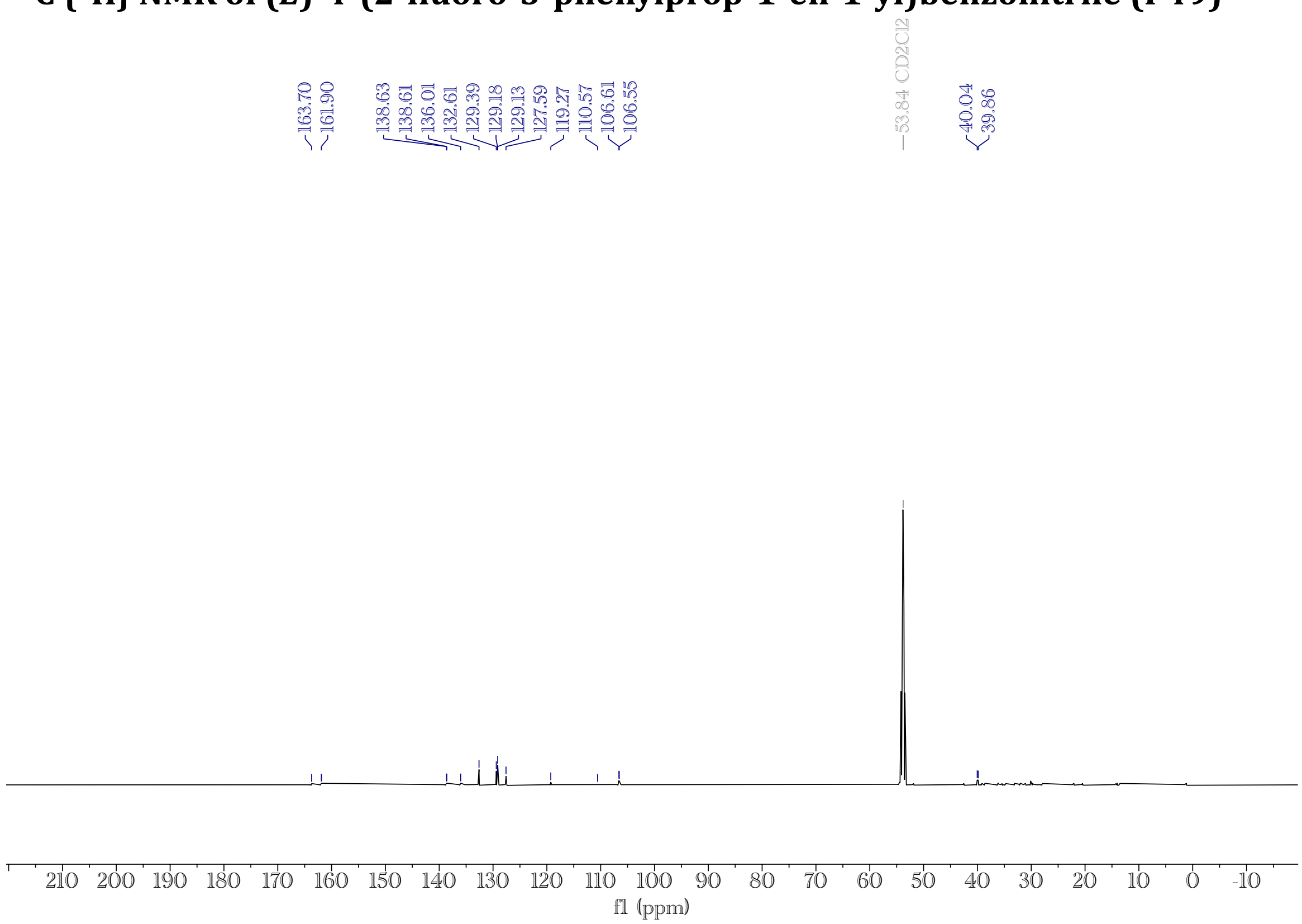

^{19}F {^{1}H} NMR of (*E*)-4-(2-fluoro-3-phenylprop-1-en-1-yl)benzonitrile (P49)

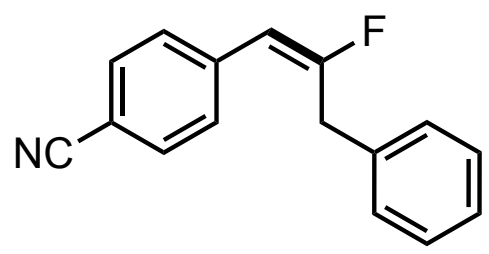

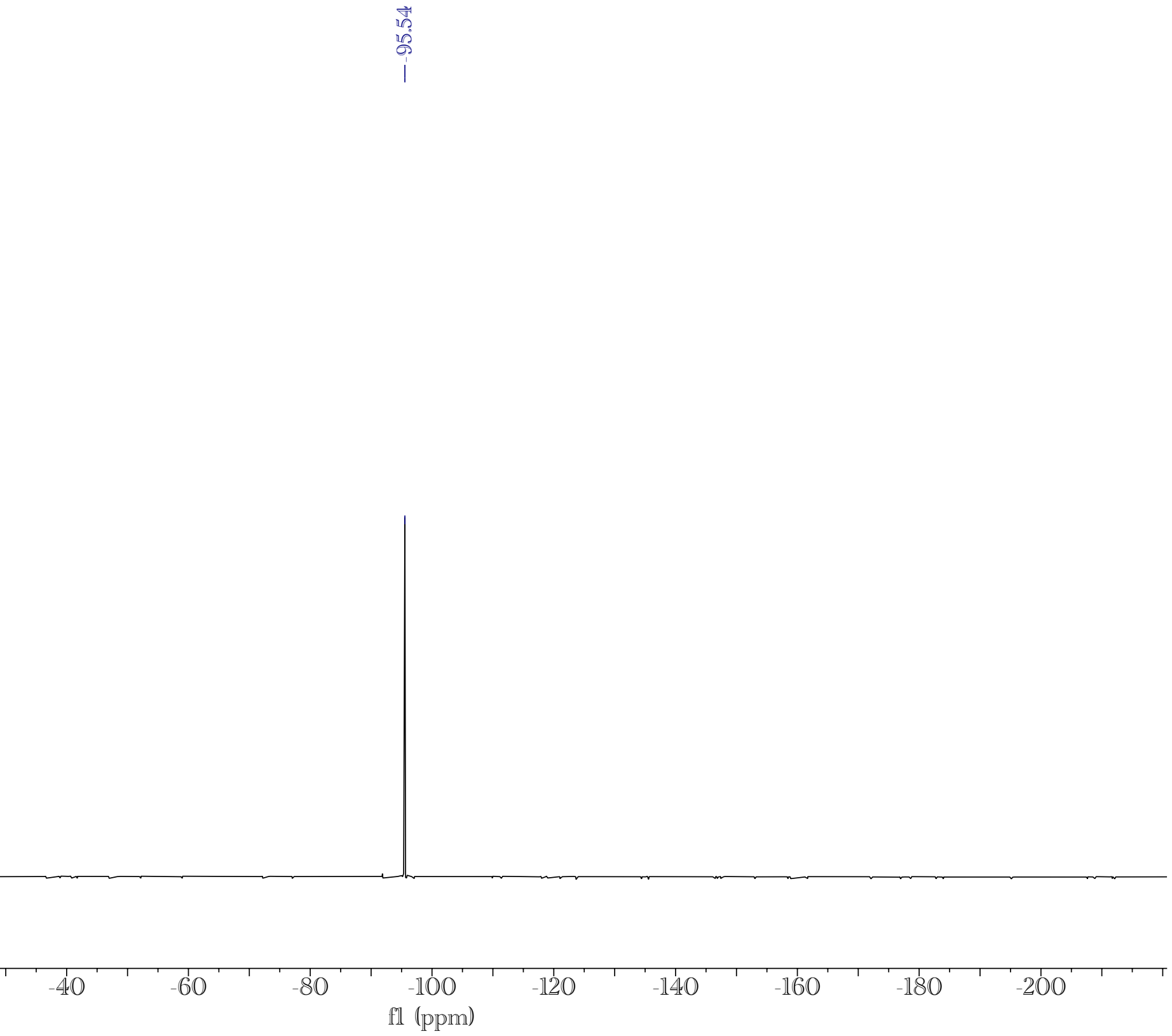

^{1}H NMR of (2-cyclohexylideneethyl)benzene (P50)

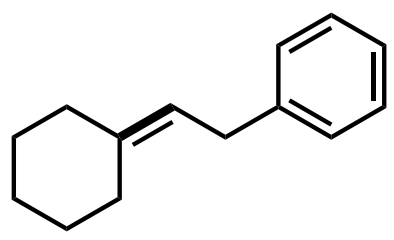

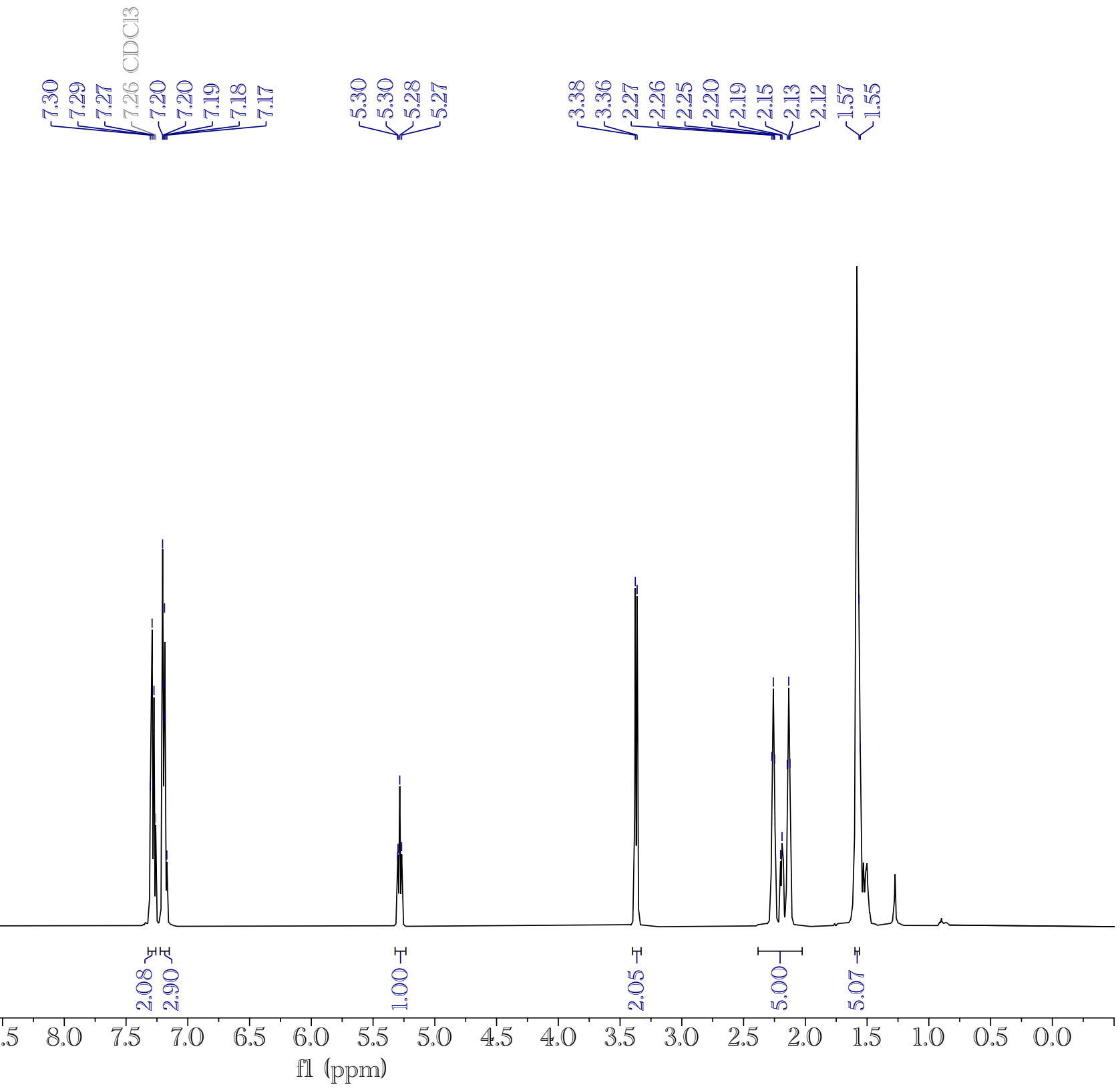

^{13}C {^{1}H} NMR of (2-cyclohexylideneethyl)benzene (P50)

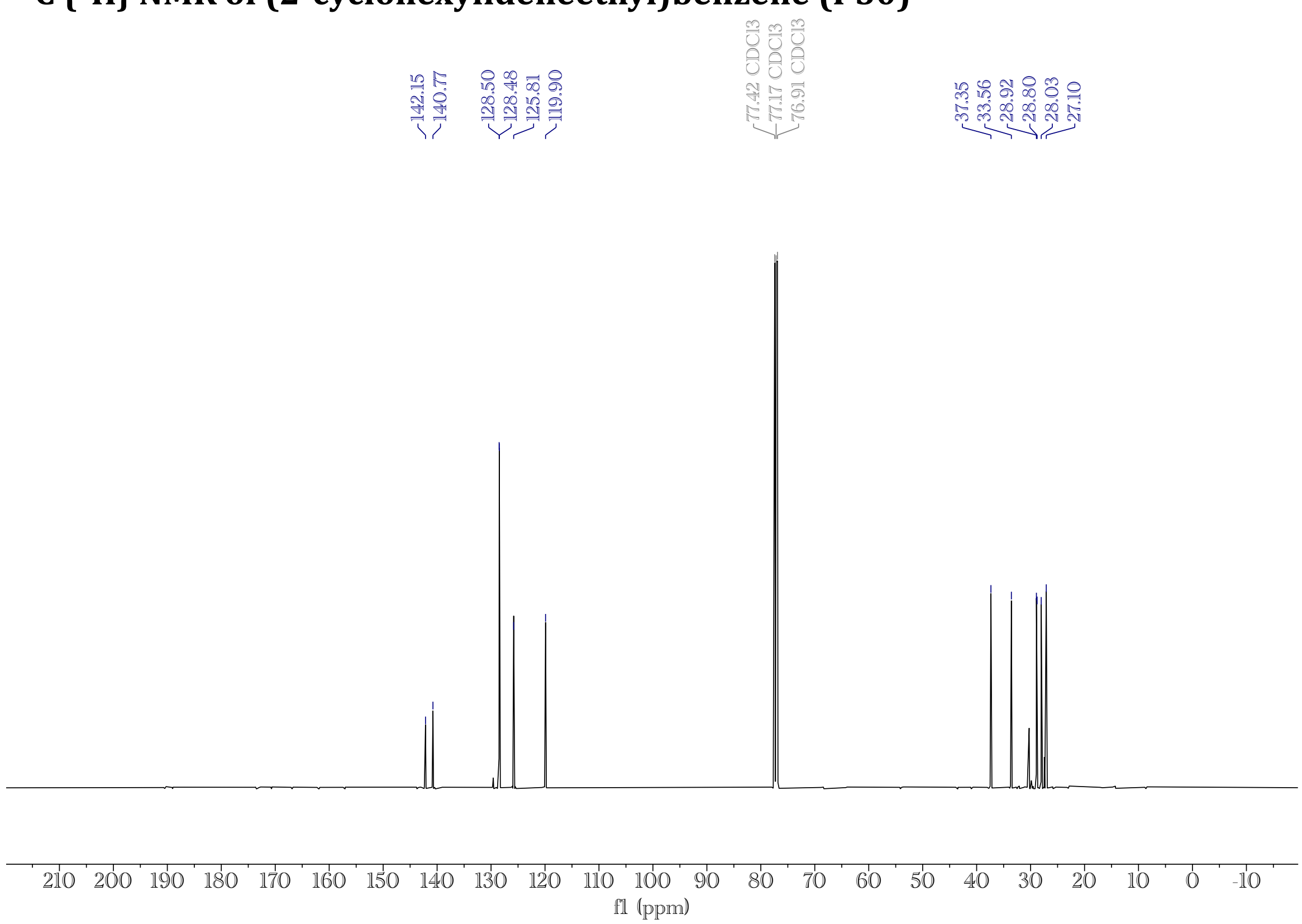

^{1}H NMR (2-cyclohexylidenepropyl)benzene (P51)

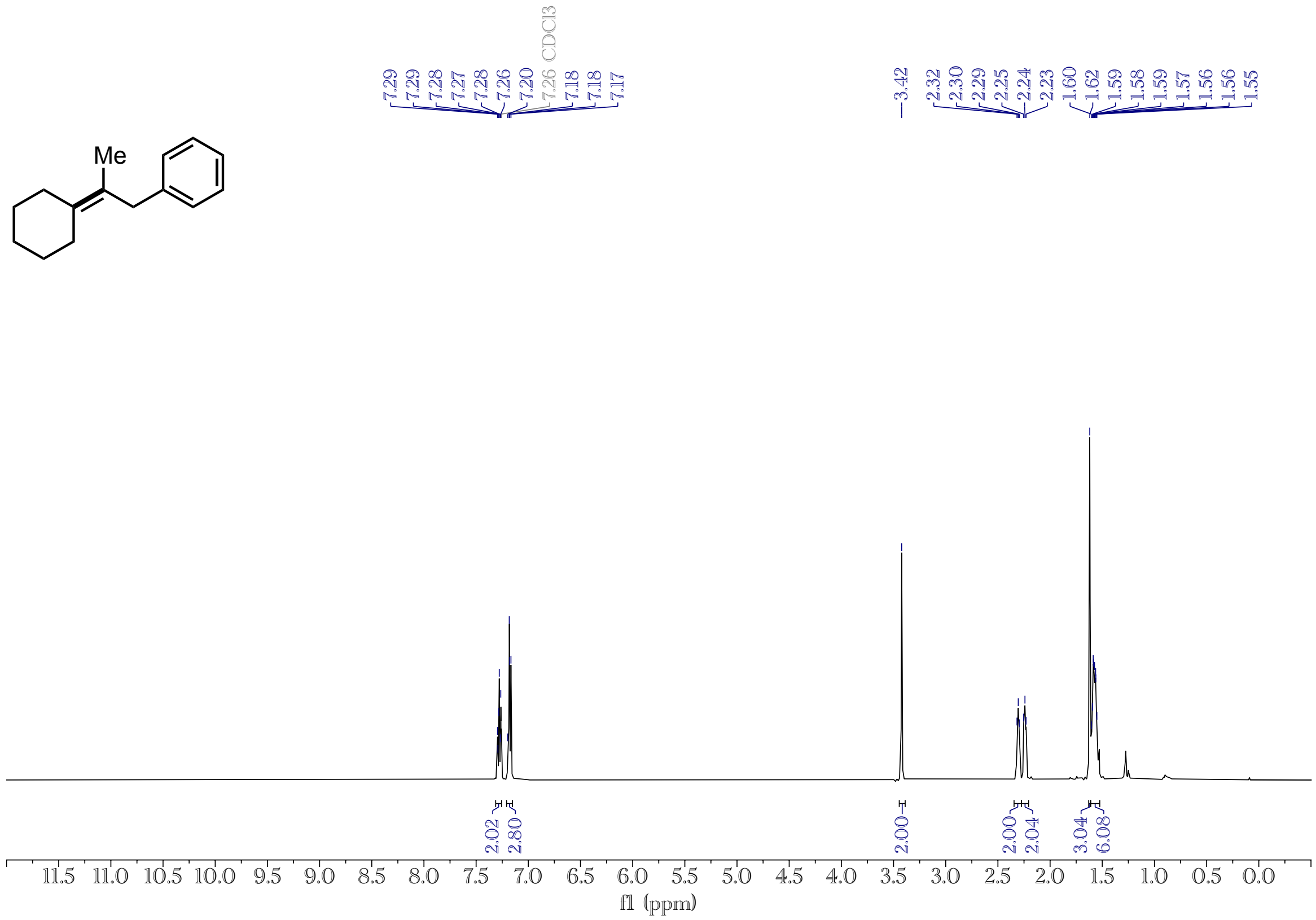

^{13}C {^{1}H} NMR of (2-cyclohexylidenepropyl)benzene (P51)

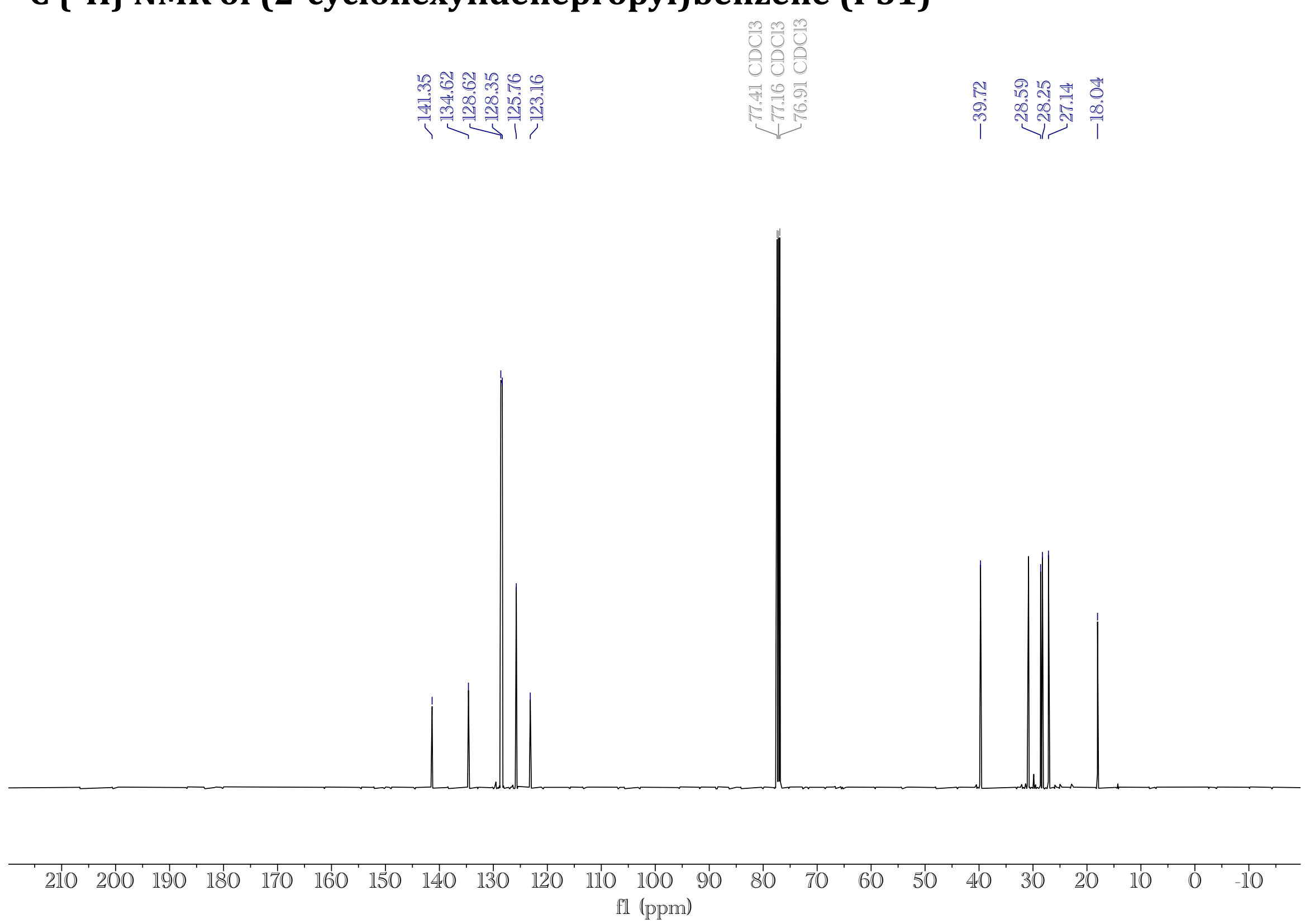

^1H NMR of (2,3-dimethylbut-2-en-1-yl)benzene (P52)

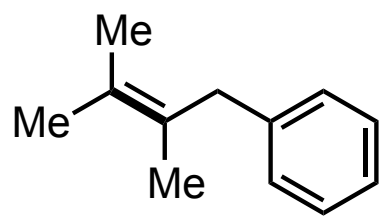

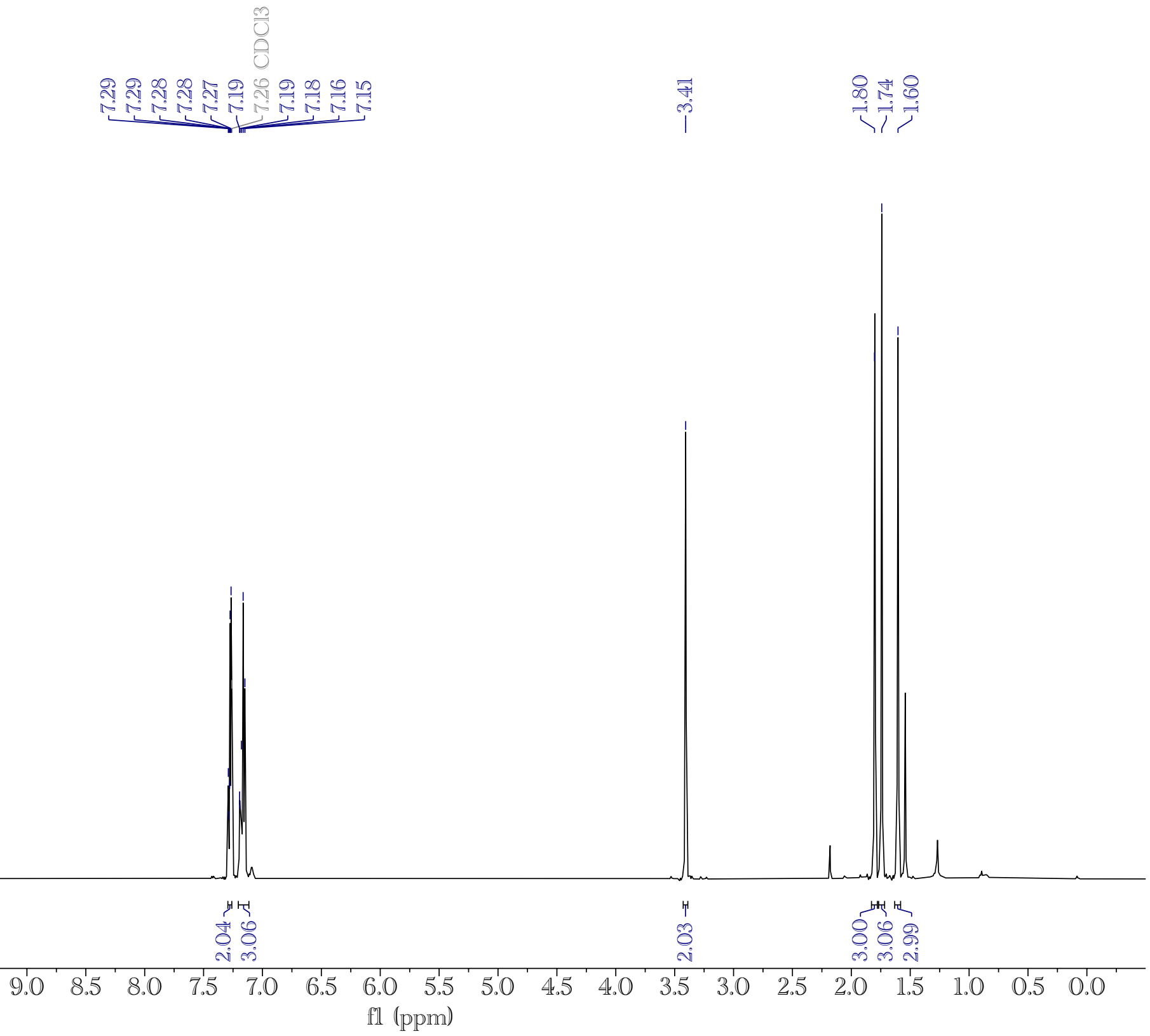

$^{13}C\{^1H\}$ NMR of (2,3-dimethylbut-2-en-1-yl)benzene (P52)

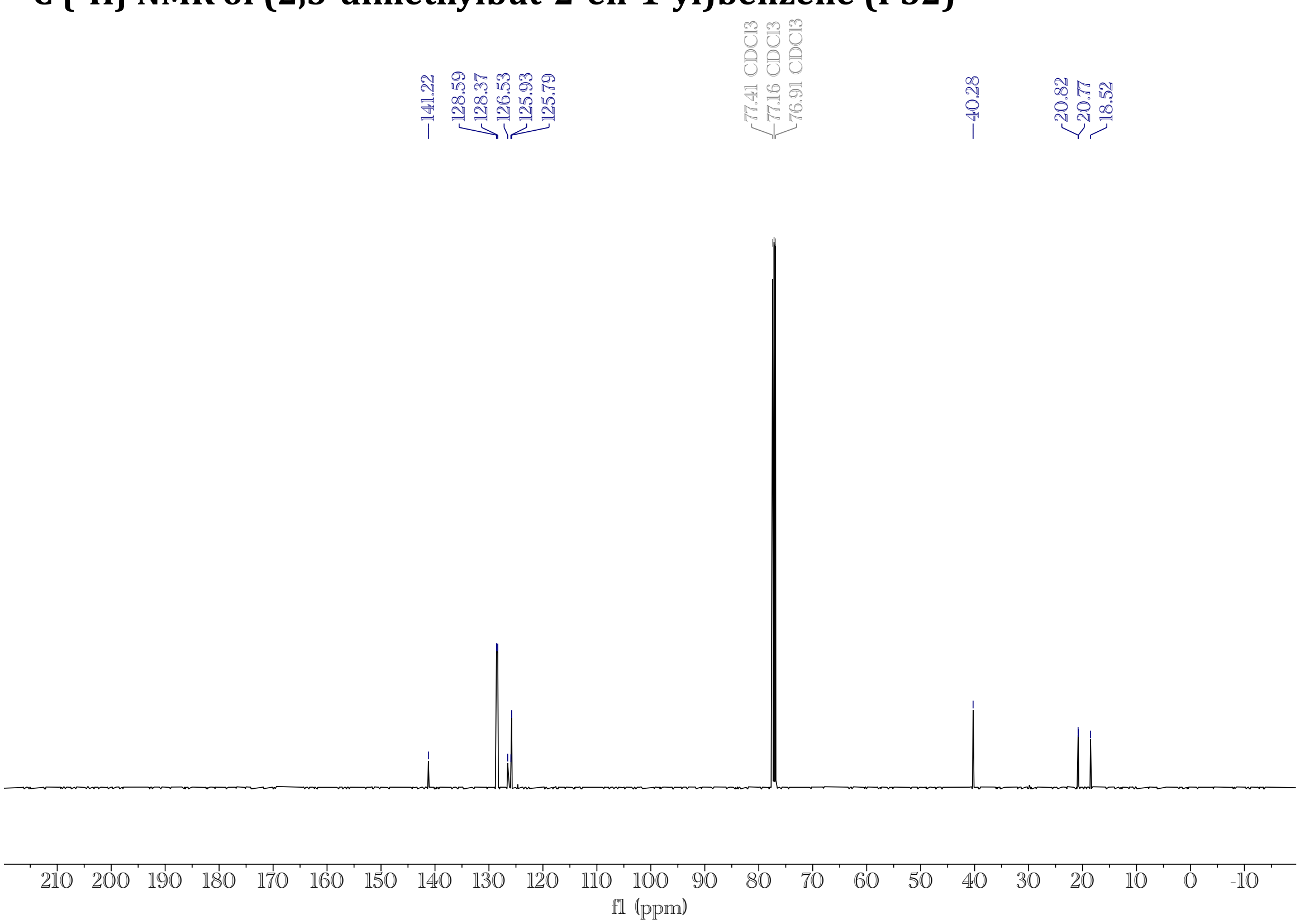

^{1}H NMR of (1R,4*S*, *E/Z*)-2-(1-phenylpropan-2-ylidene)bicyclo[2.2.1]heptane (P53)

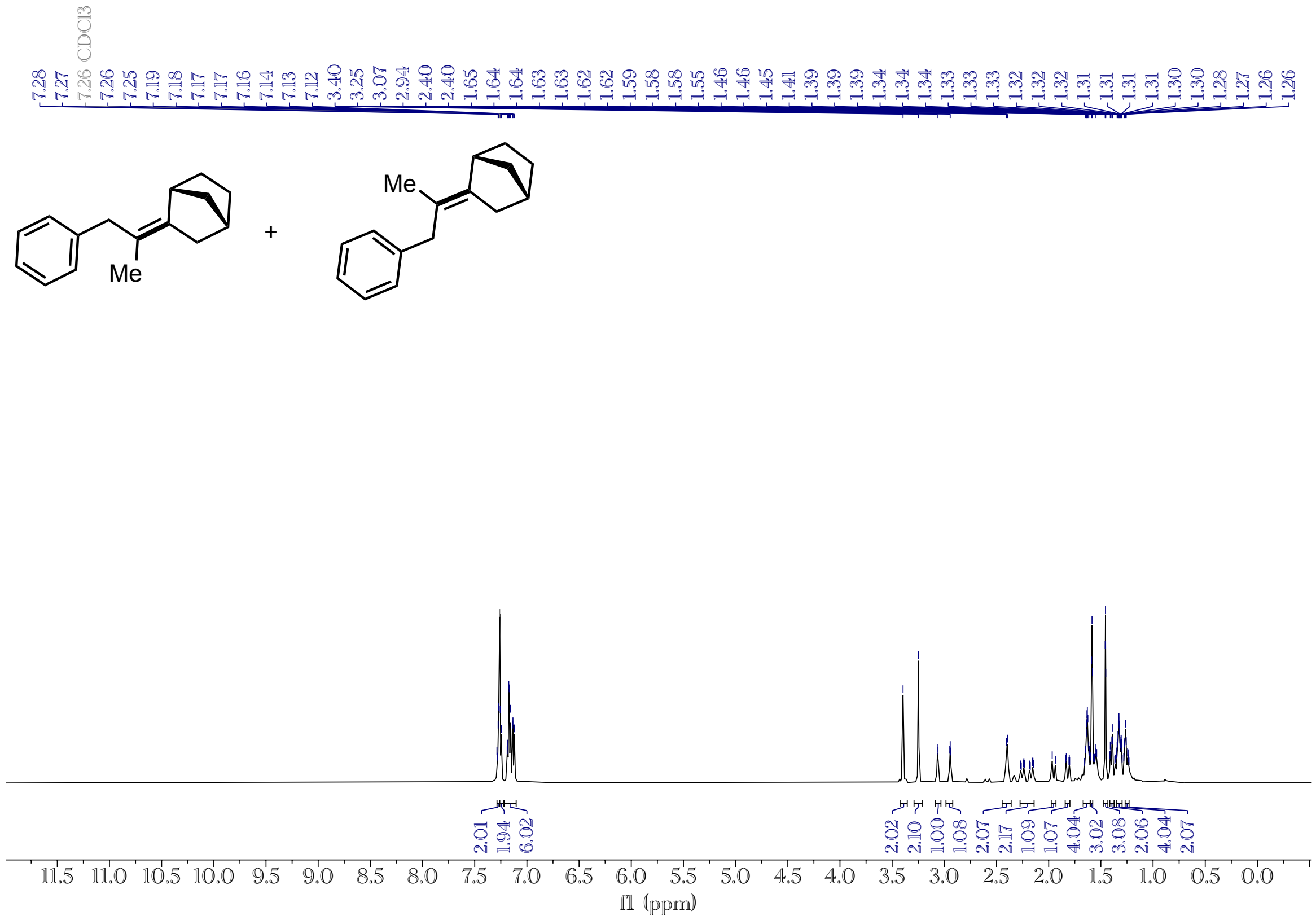

^{13}C {^{1}H} NMR of (1*R*,4*S*,*E*)-2-(1-phenylpropan-2-ylidene)bicyclo[2.2.1]heptane (P53)

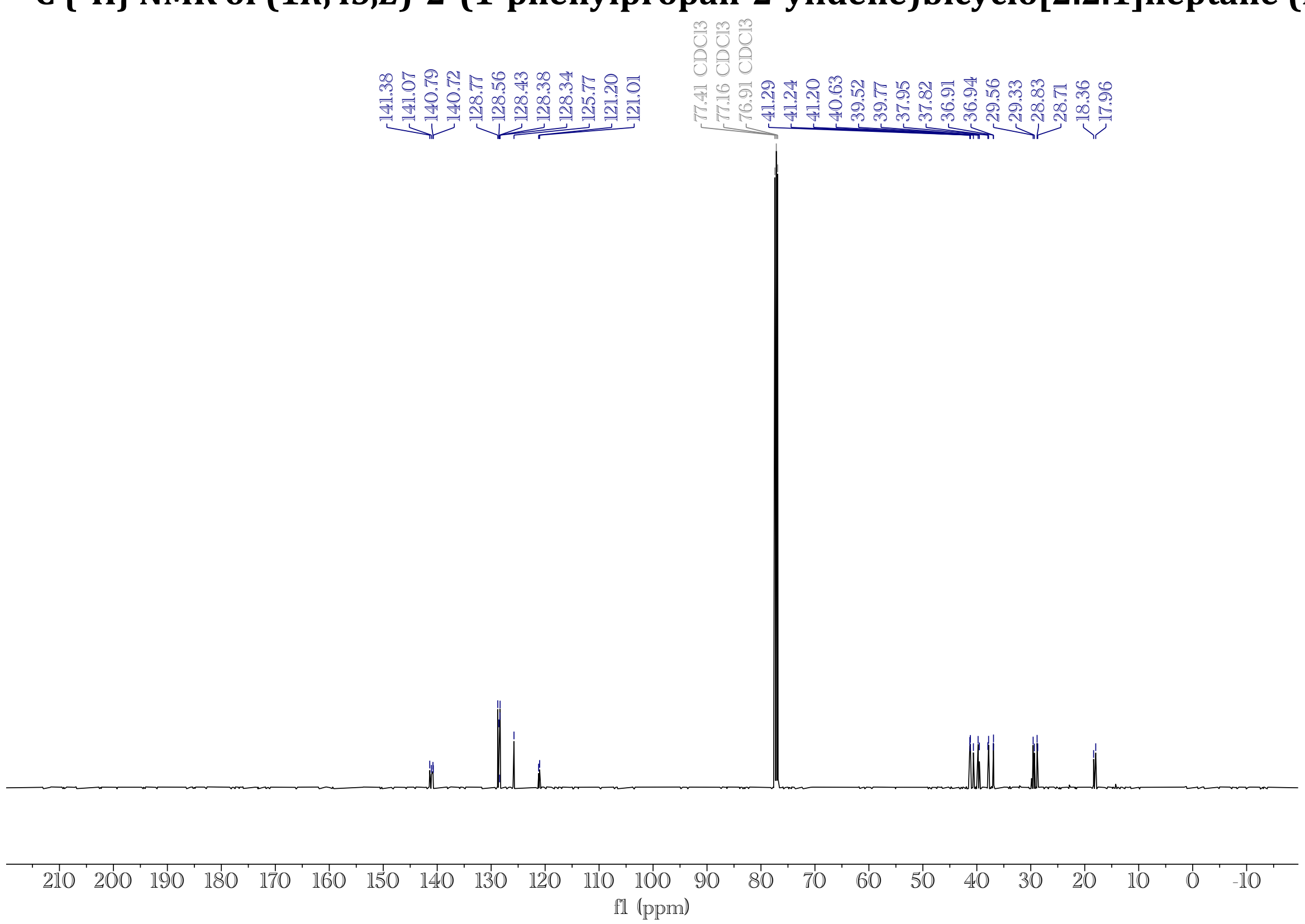

^{1}H NMR of *tert*-butyl 4-((1R,4*S*)-bicyclo[2.2.1]heptan-2-ylidene)piperidine-1-carboxylate (P54)

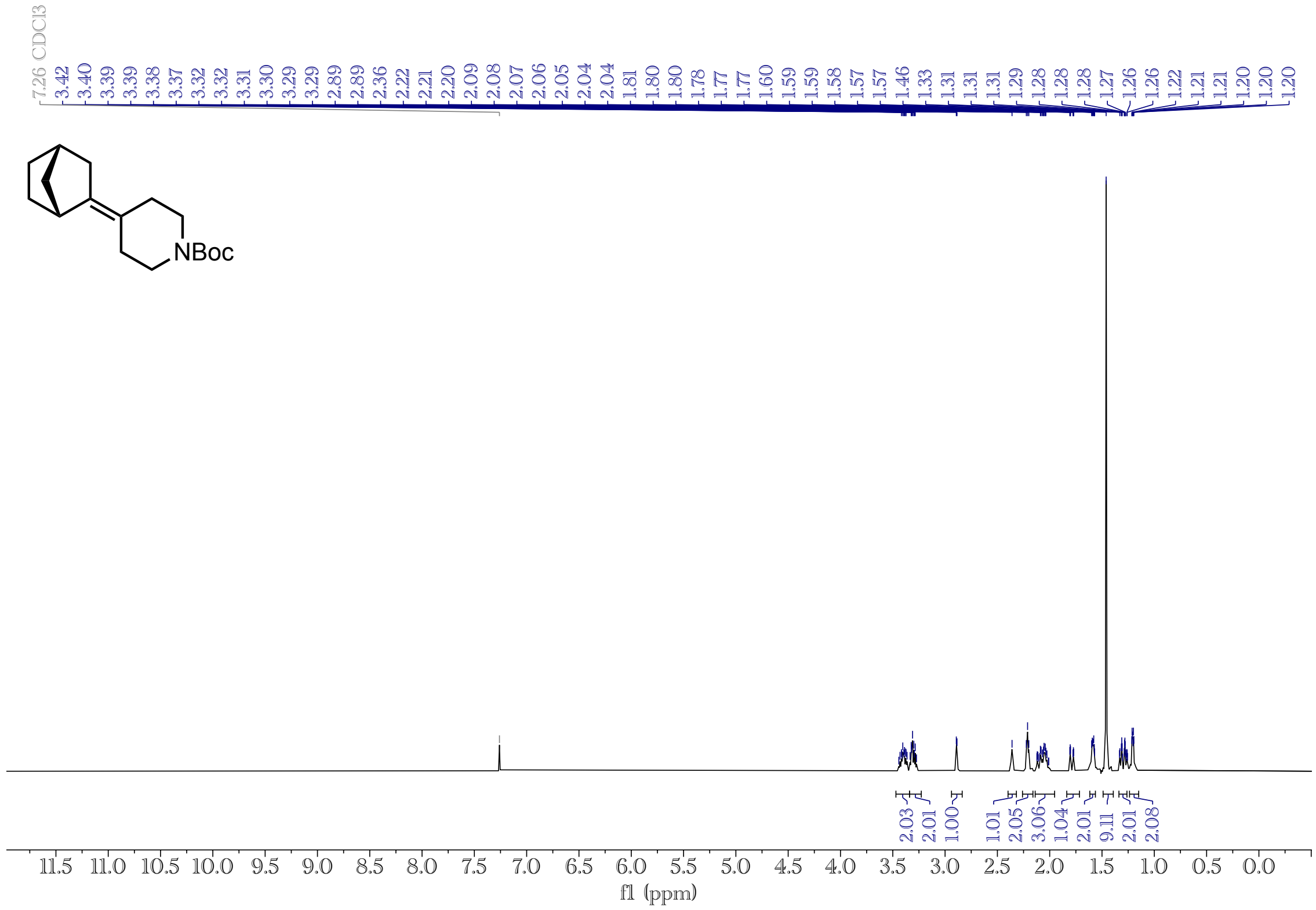

^{13}C {^{1}H} NMR of *tert*-butyl 4-((1R,4*S*)-bicyclo[2.2.1]heptan-2-ylidene)piperidine-1-carboxylate (P54)

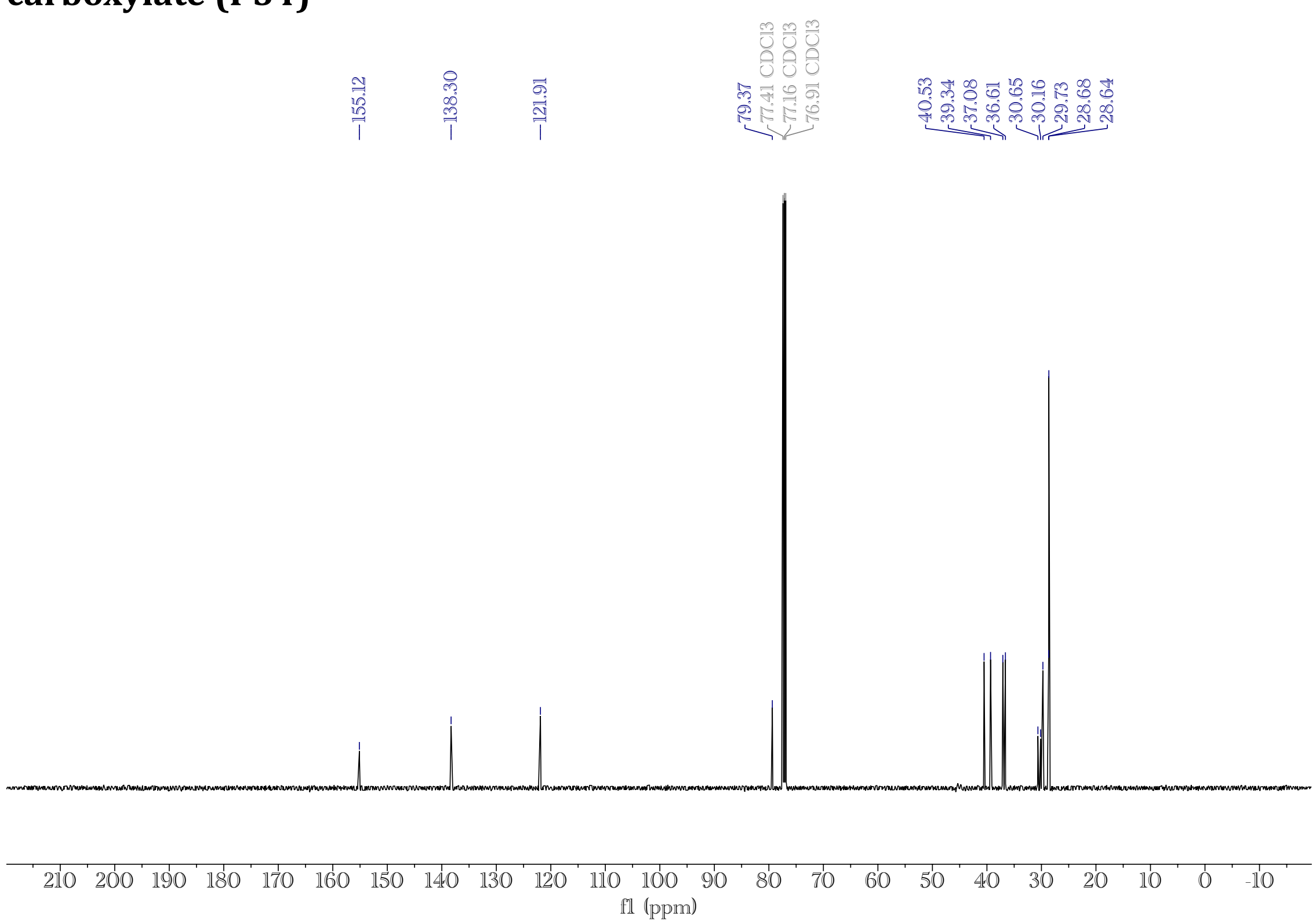

^{1}H NMR of dibenzyl 4-(propan-2-ylidene)heptanedioate (P55)

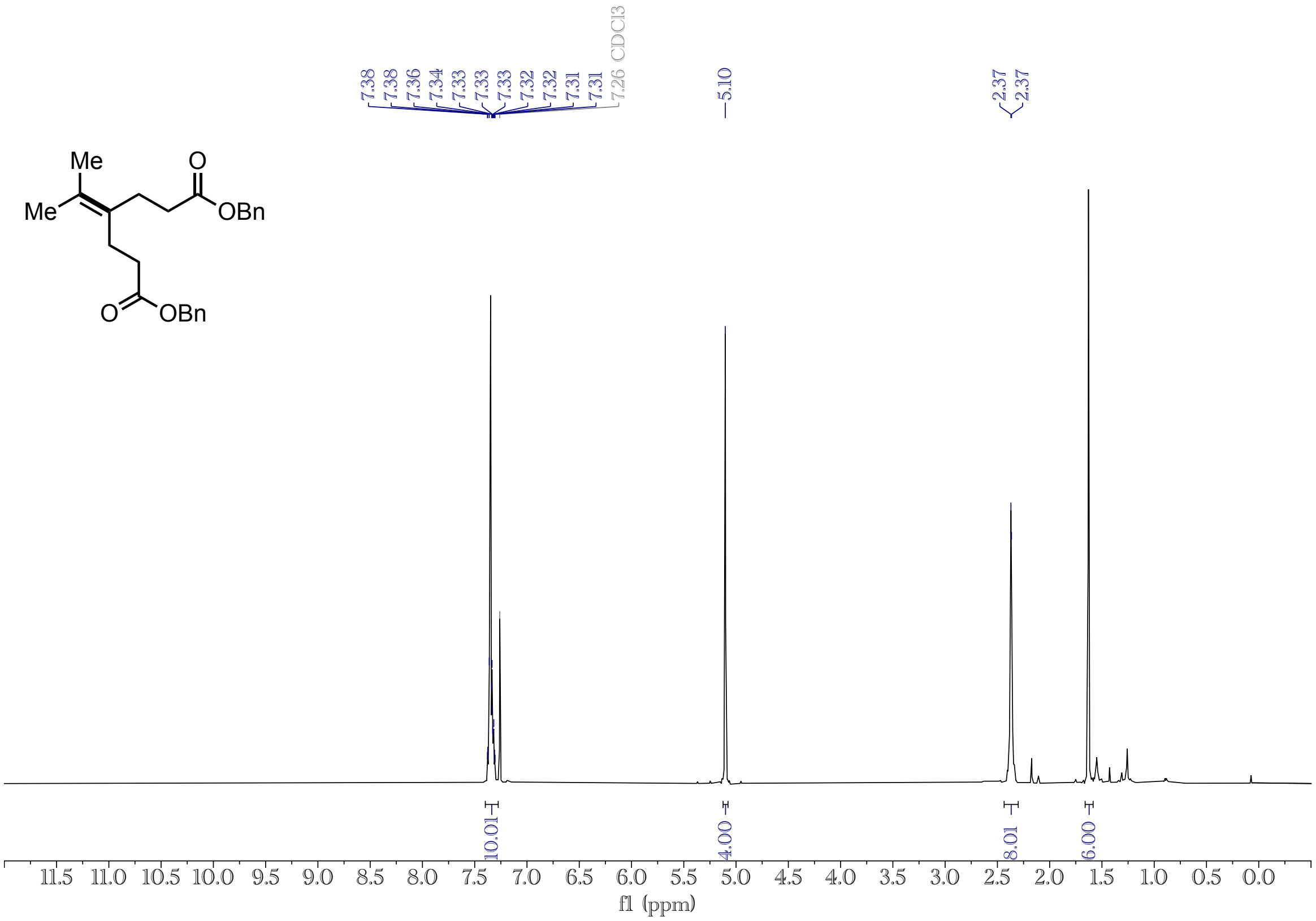

^{13}C {^{1}H} NMR of dibenzyl 4-(propan-2-ylidene)heptanedioate (P55)

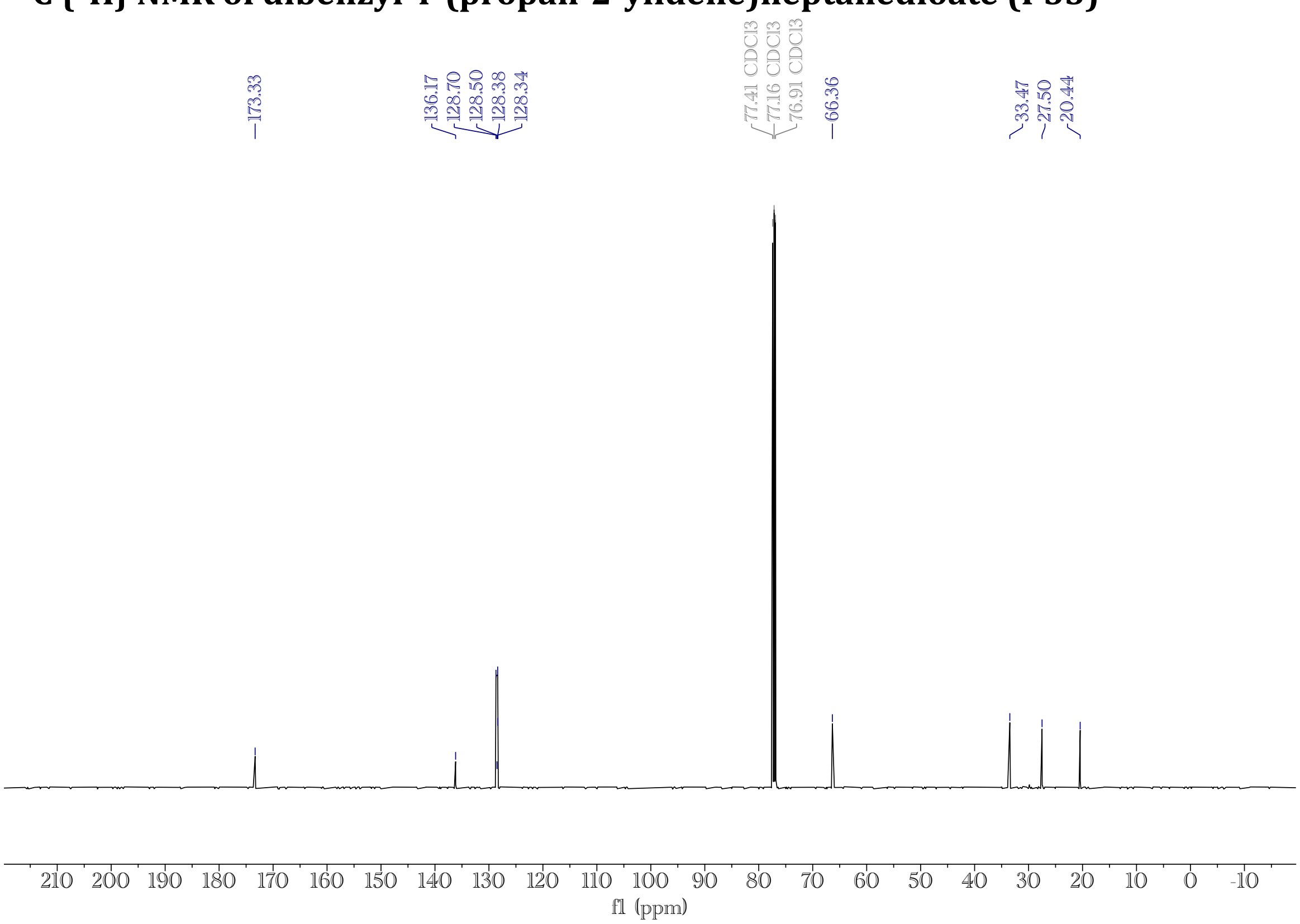

1H NMR of (*E*/*Z*)-(2-ethylhepta-1,6-dien-1-yl)benzene (P56)

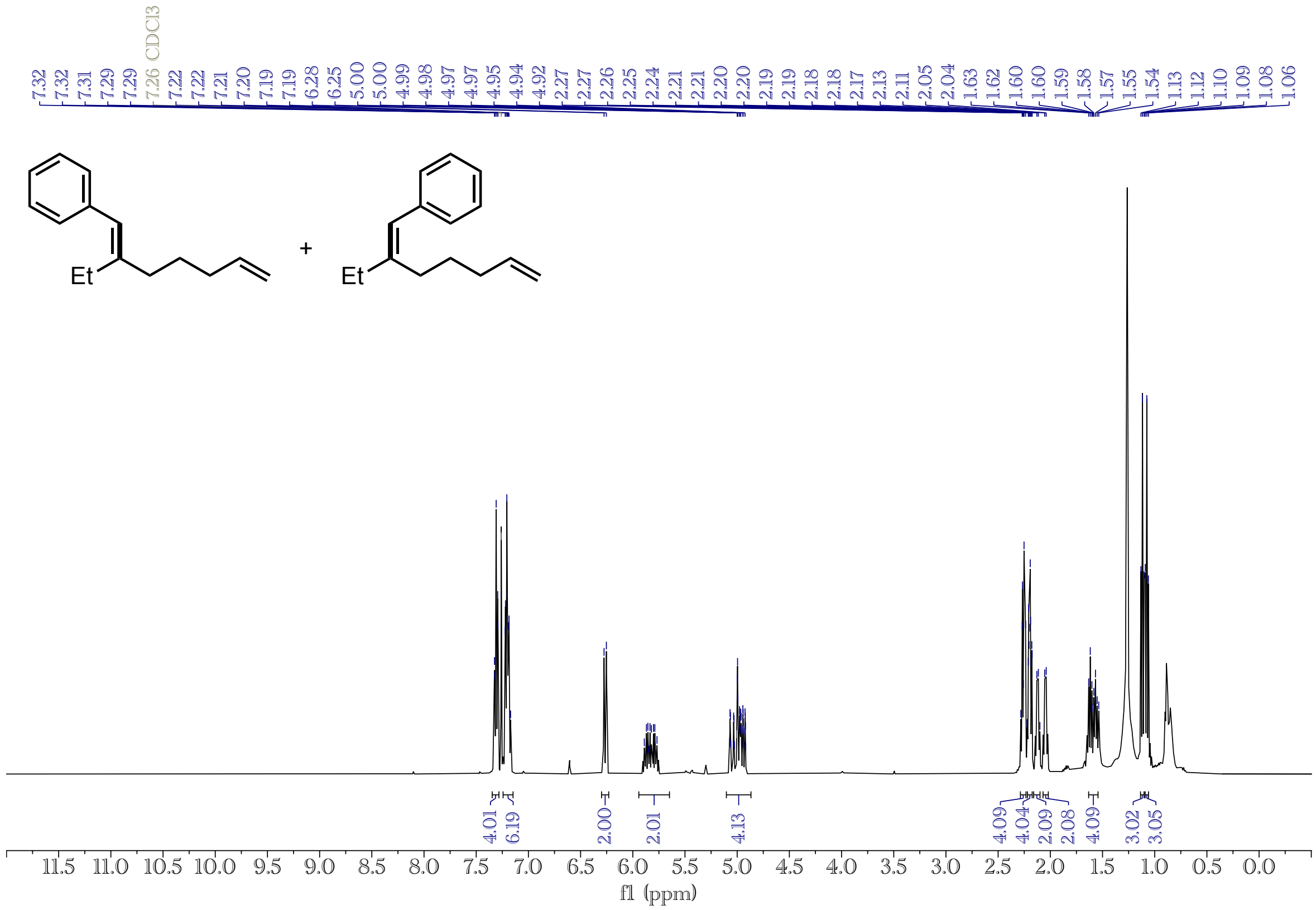

13C {1H} NMR of (*E*/*Z*)-(2-ethylhepta-1,6-dien-1-yl)benzene (P56)

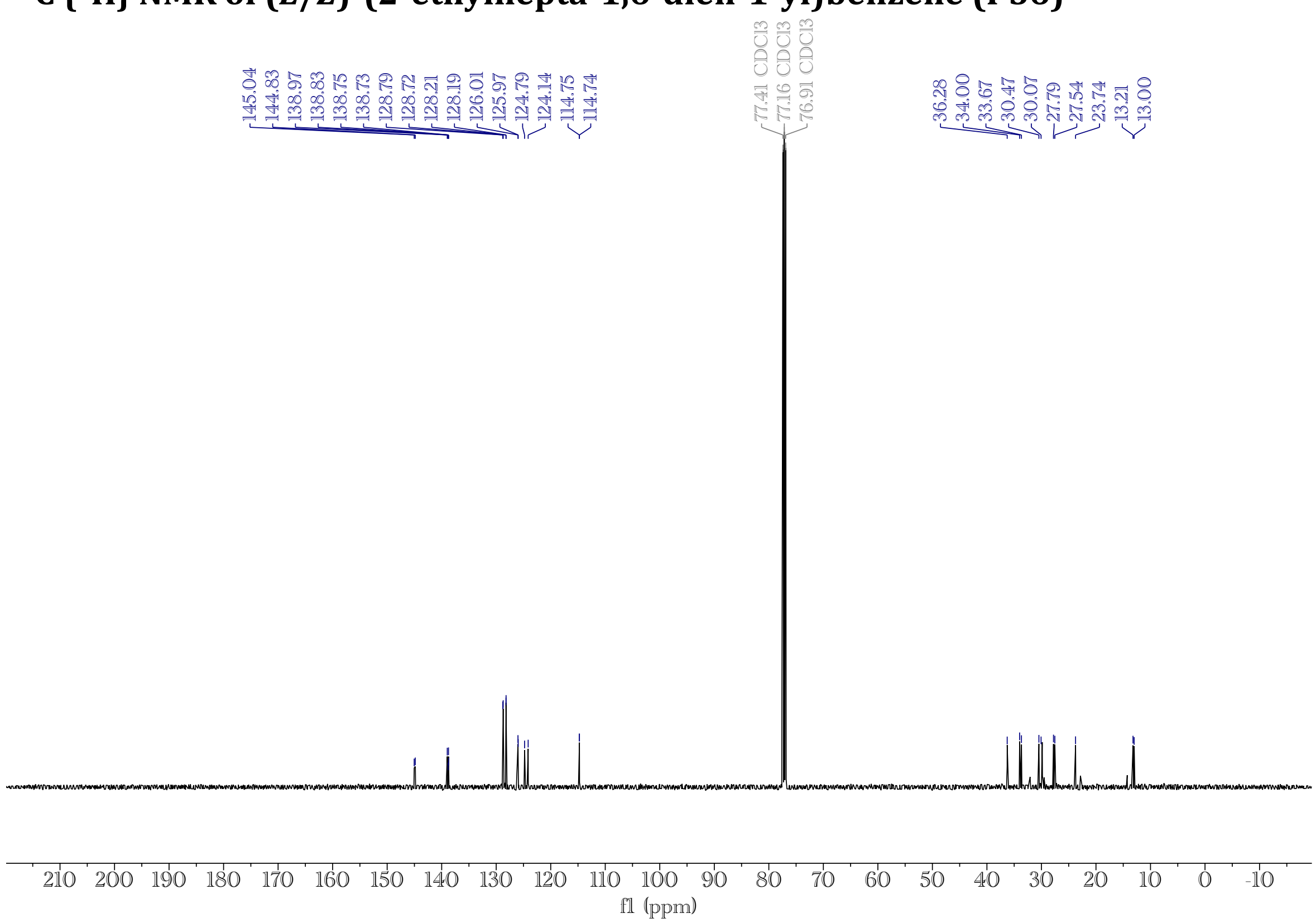

^{1}H NMR of (*E*)-1,1,4,4-tetramethyl-6-(1-phenylbut-1-en-2-yl)-1,2,3,4-tetrahydronaphthalene (P57)

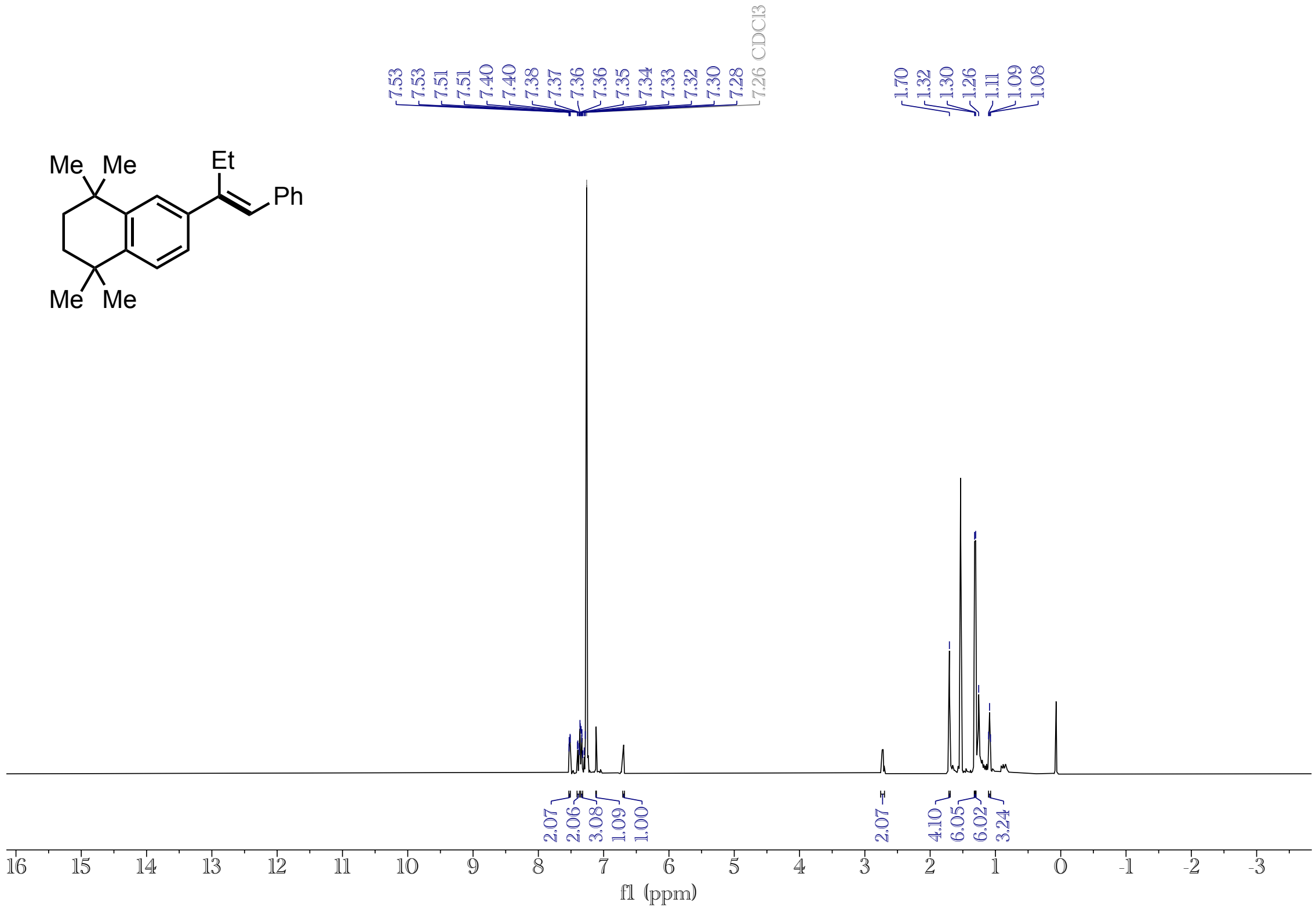

^{13}C {^{1}H} NMR of (*E*)-1,1,4,4-tetramethyl-6-(1-phenylbut-1-en-2-yl)-1,2,3,4-tetrahydronaphthalene (P57)

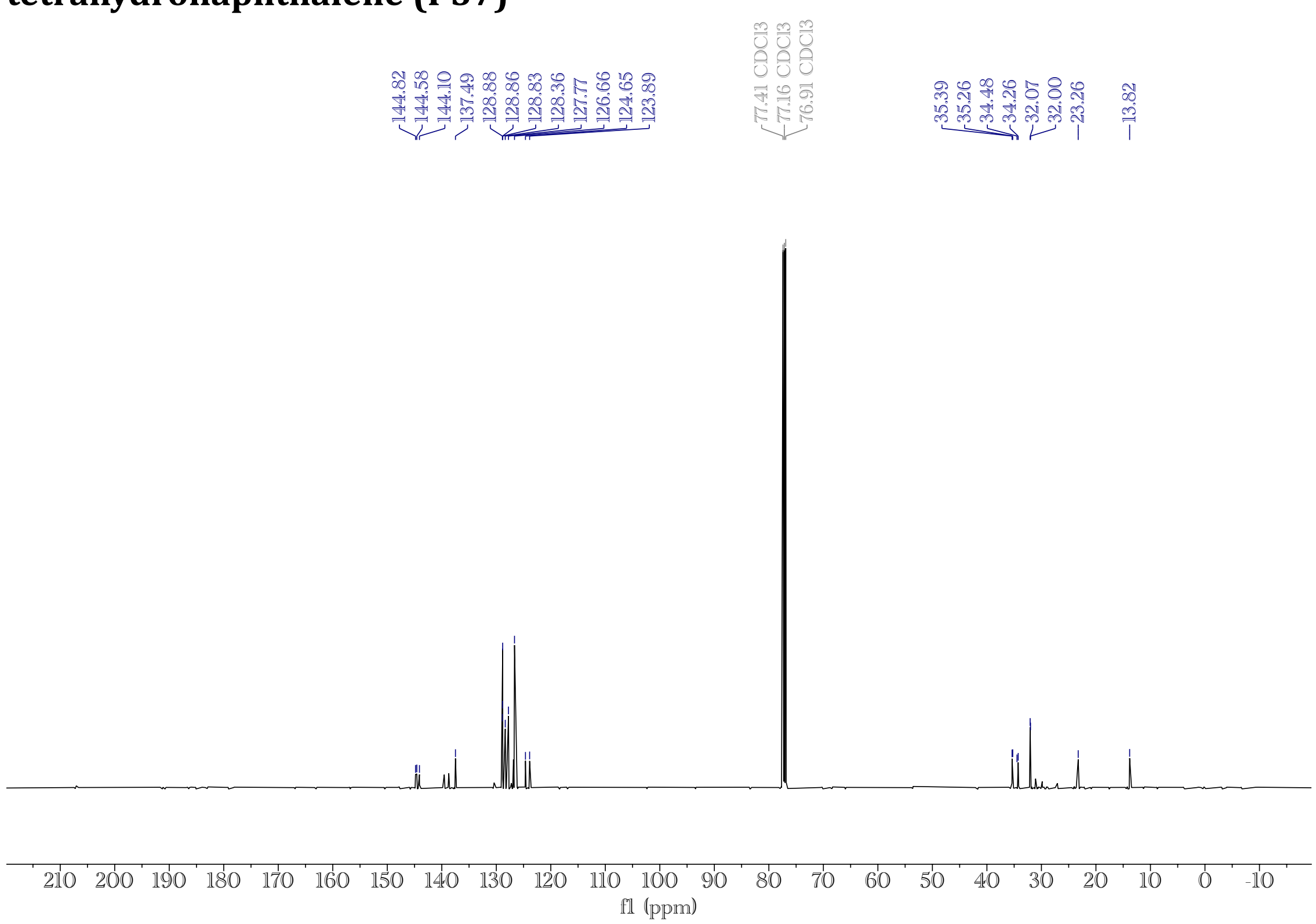

NMR Spectra of Starting Materials

¹H NMR of 3-(nitromethyl)bicyclo[4.2.0]octa-1(6),2,4-triene (S12)

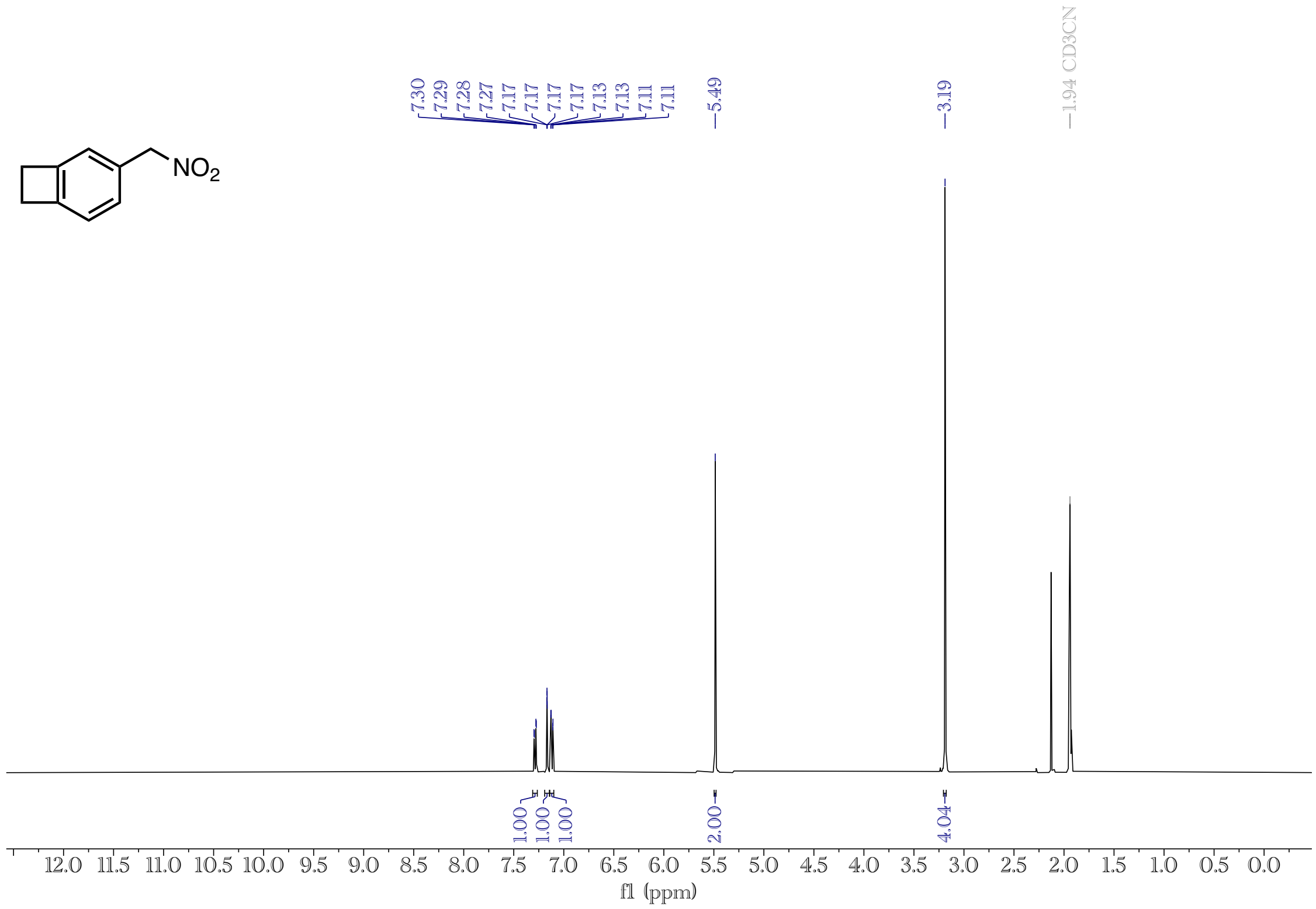

¹³C {¹H} NMR of 3-(nitromethyl)bicyclo[4.2.0]octa-1(6),2,4-triene (S12)

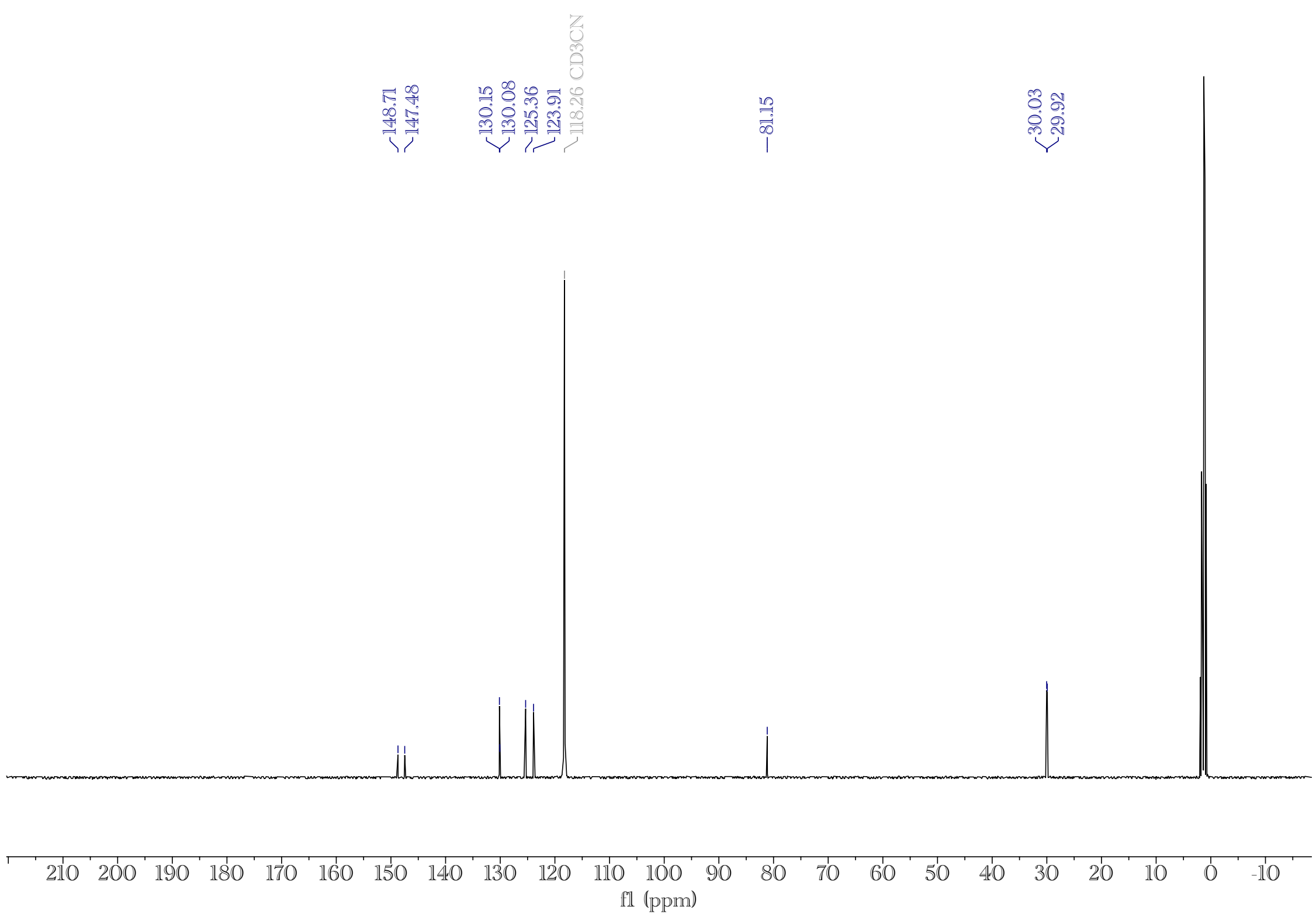

1H NMR of 1-(4-(nitromethyl)phenyl)propan-2-one (S13)

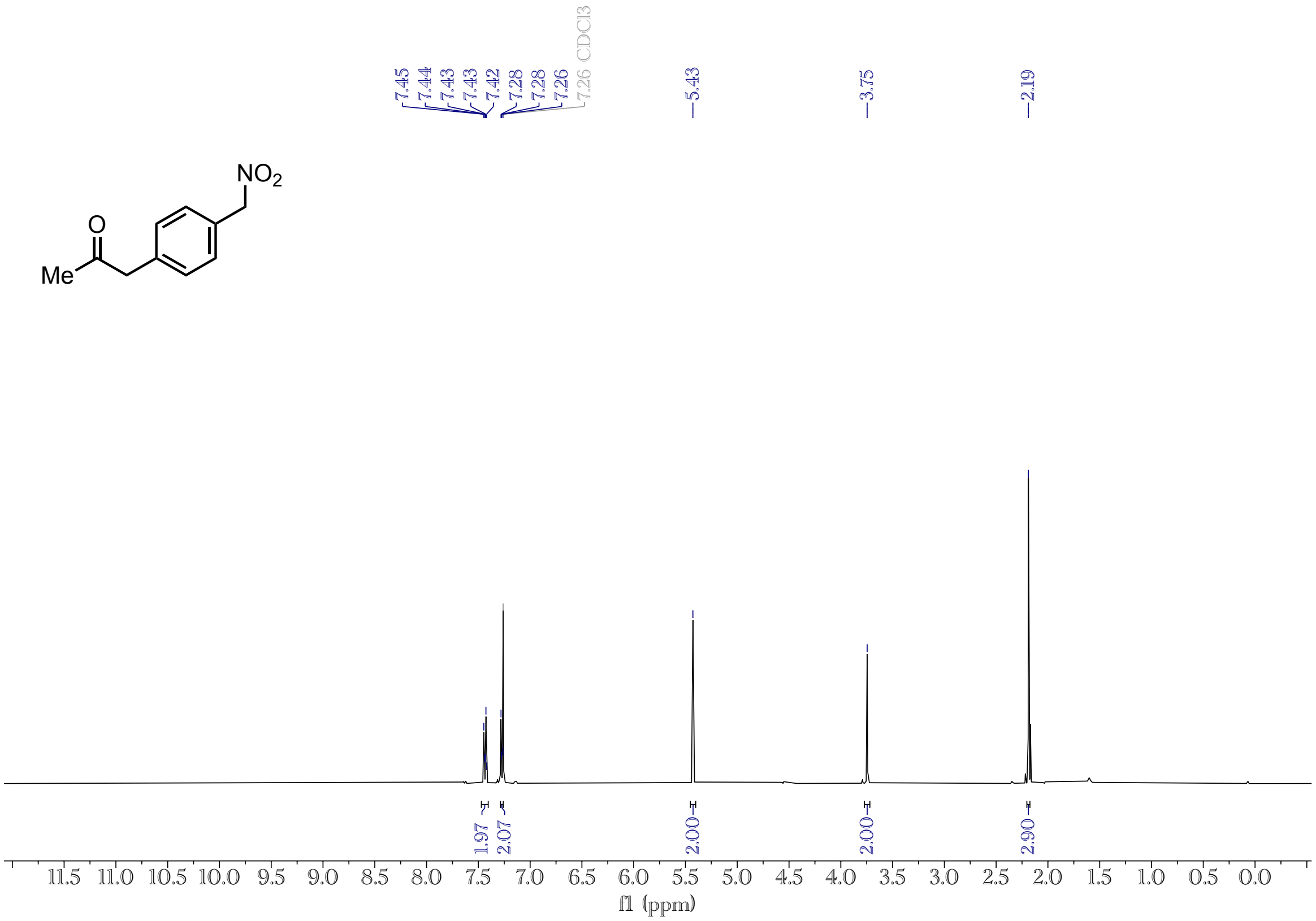

13C {1H} NMR of 1-(4-(nitromethyl)phenyl)propan-2-one (S13)

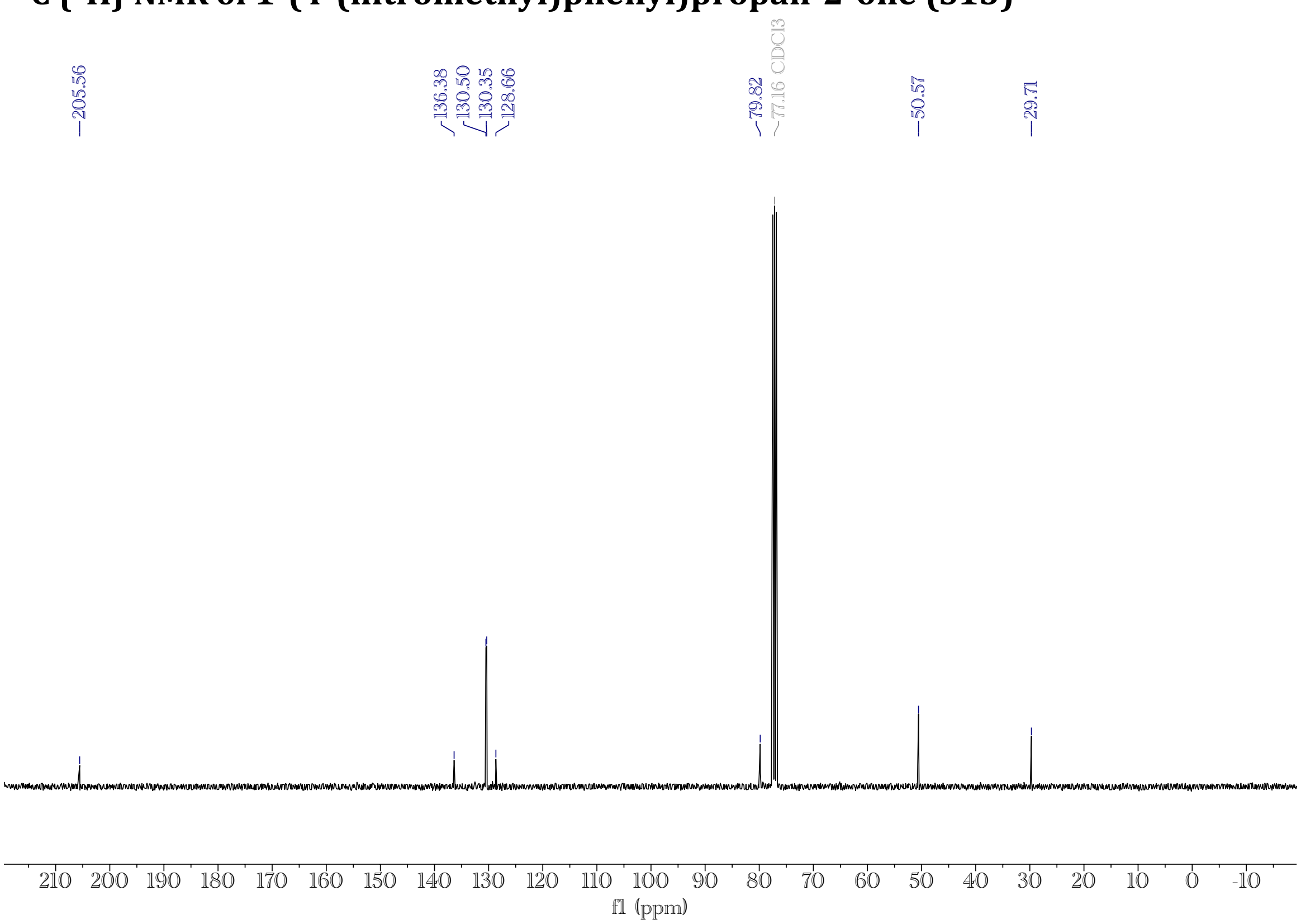

^{1}H NMR of 4-(nitromethyl)benzaldehyde (S14)

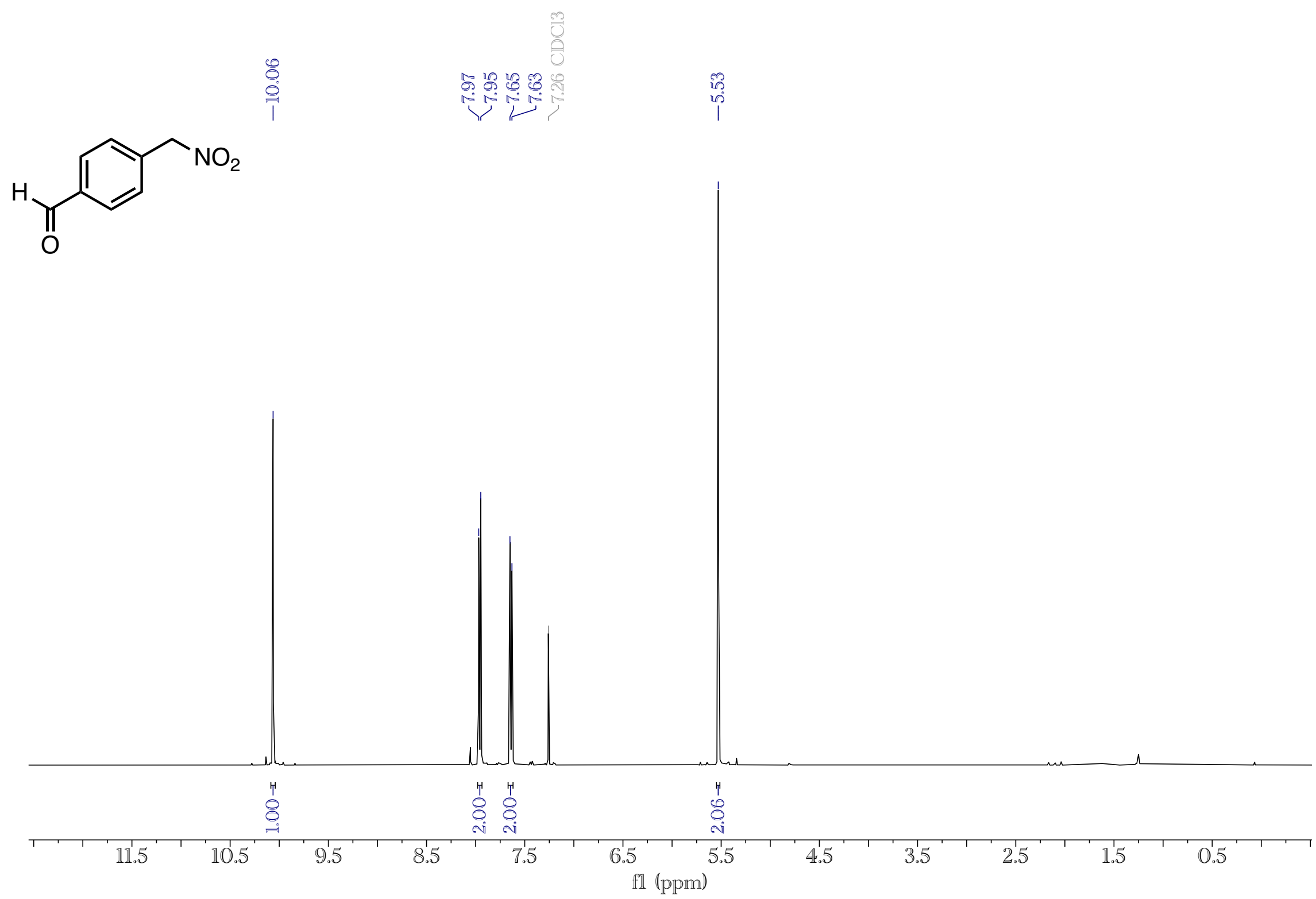

^{13}C {^{1}H} NMR of 4-(nitromethyl)benzaldehyde (S14)

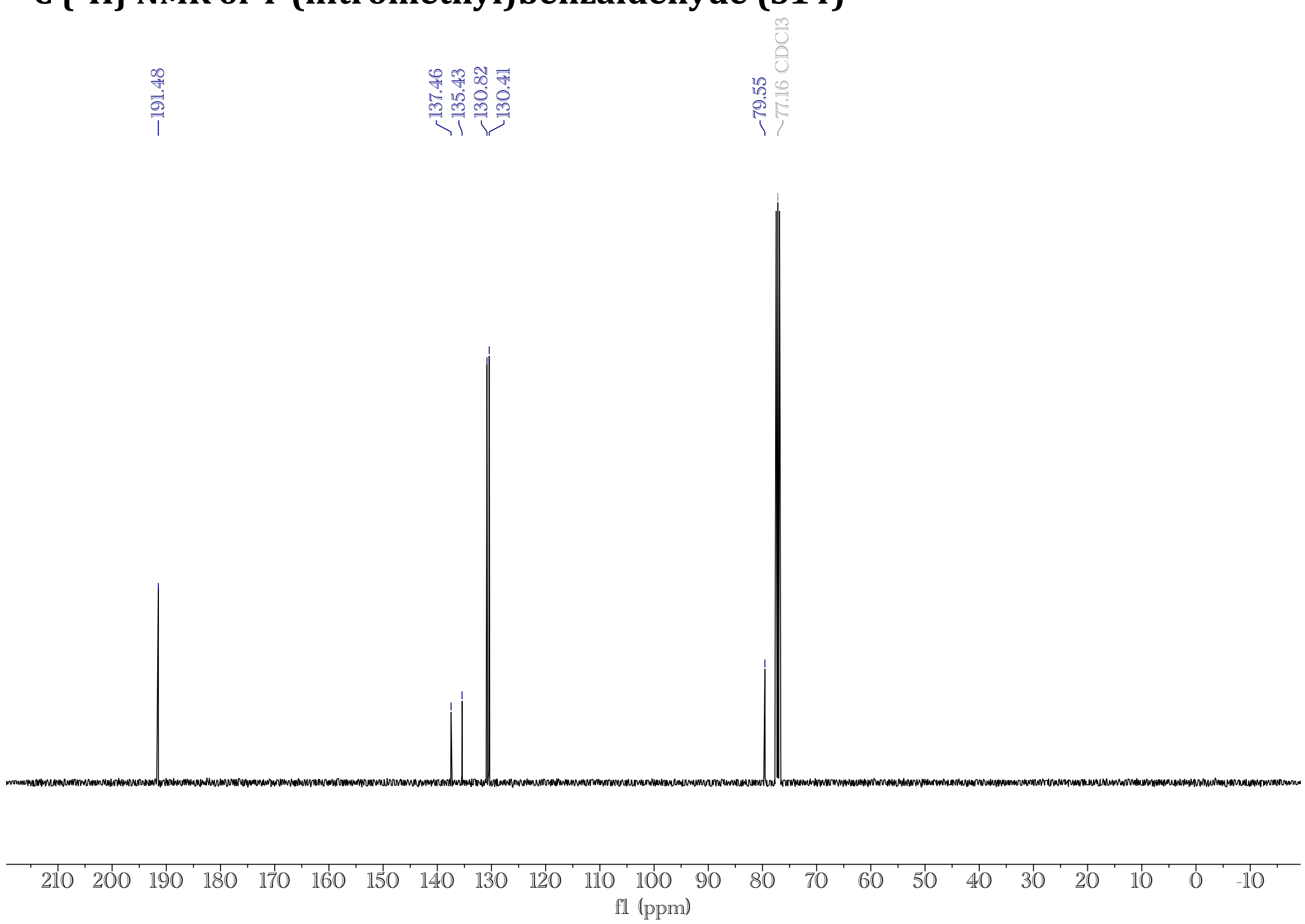

^{1}H NMR of 5-(nitromethyl)-2,3-dihydrobenzofuran (S16)

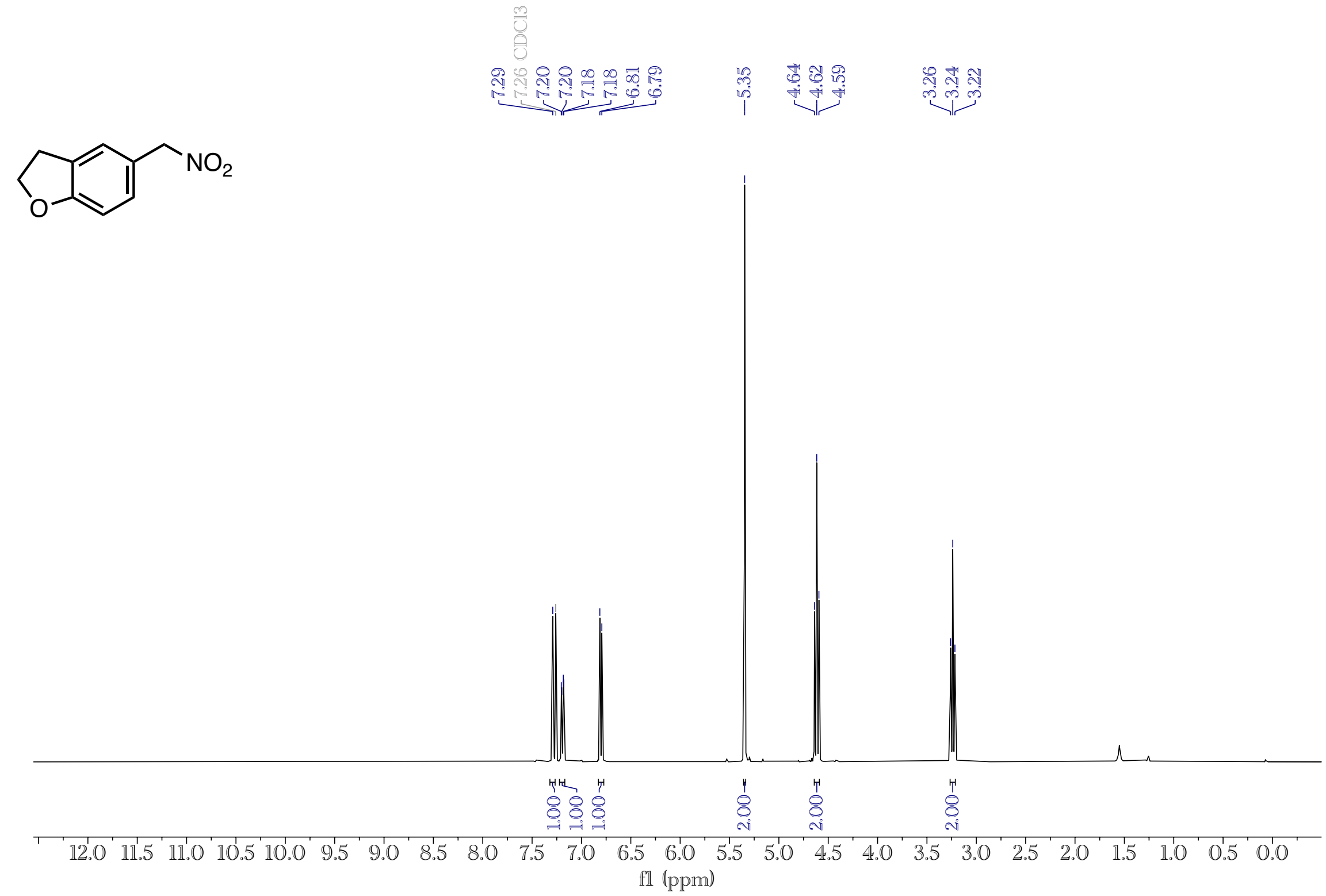

^{13}C {^{1}H} NMR of 5-(nitromethyl)-2,3-dihydrobenzofuran (S16)

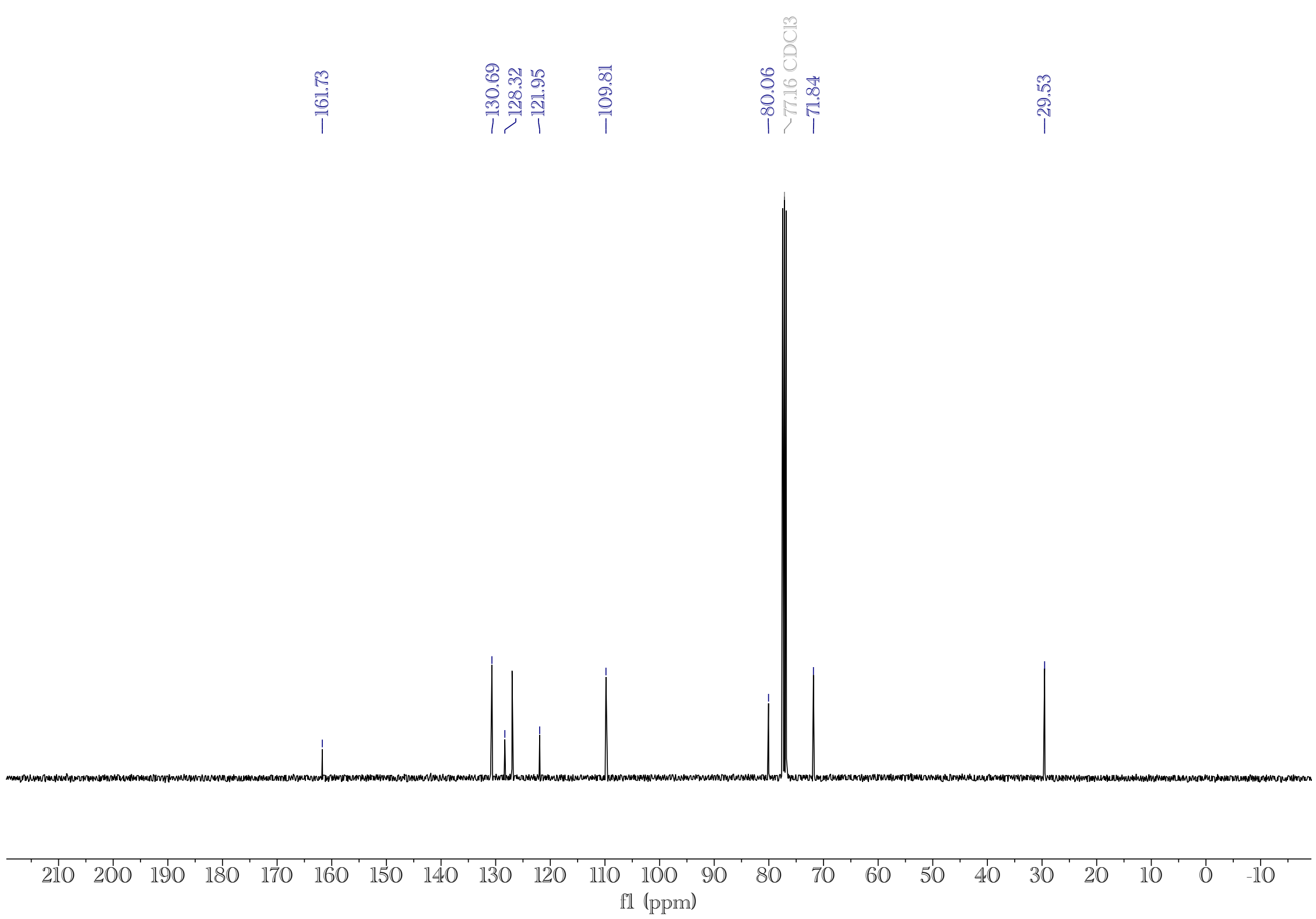

¹H NMR of tetraethyl (3-nitropentane-1,5-diyl)bis(phosphonate) (S29)

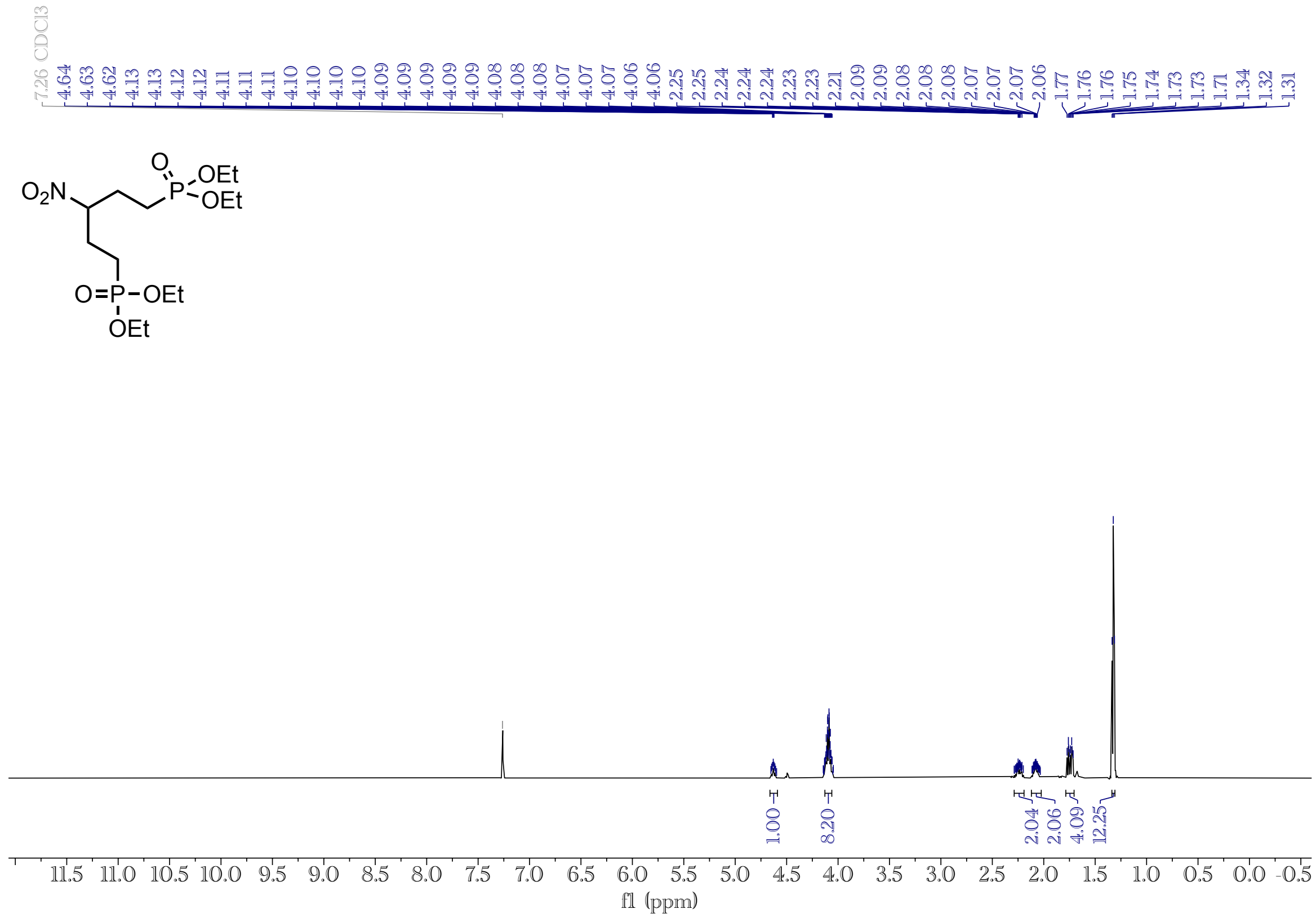

¹³C NMR of tetraethyl (3-nitropentane-1,5-diyl)bis(phosphonate) (S29)

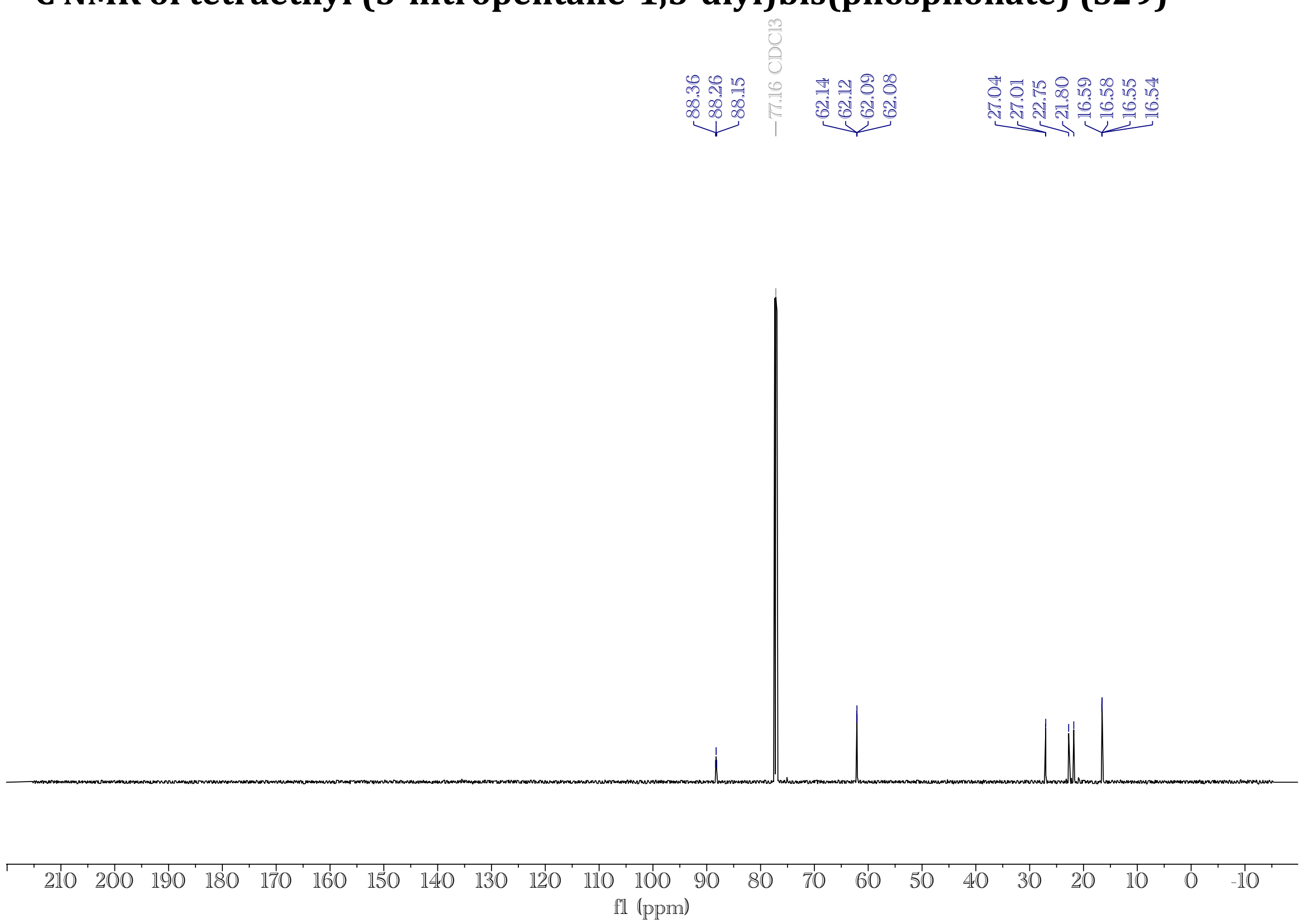

^{31}P NMR of tetraethyl (3-nitropentane-1,5-diyl)bis(phosphonate) (S29)

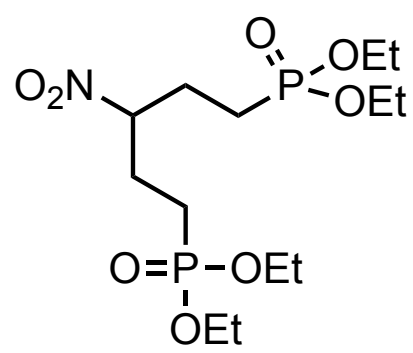

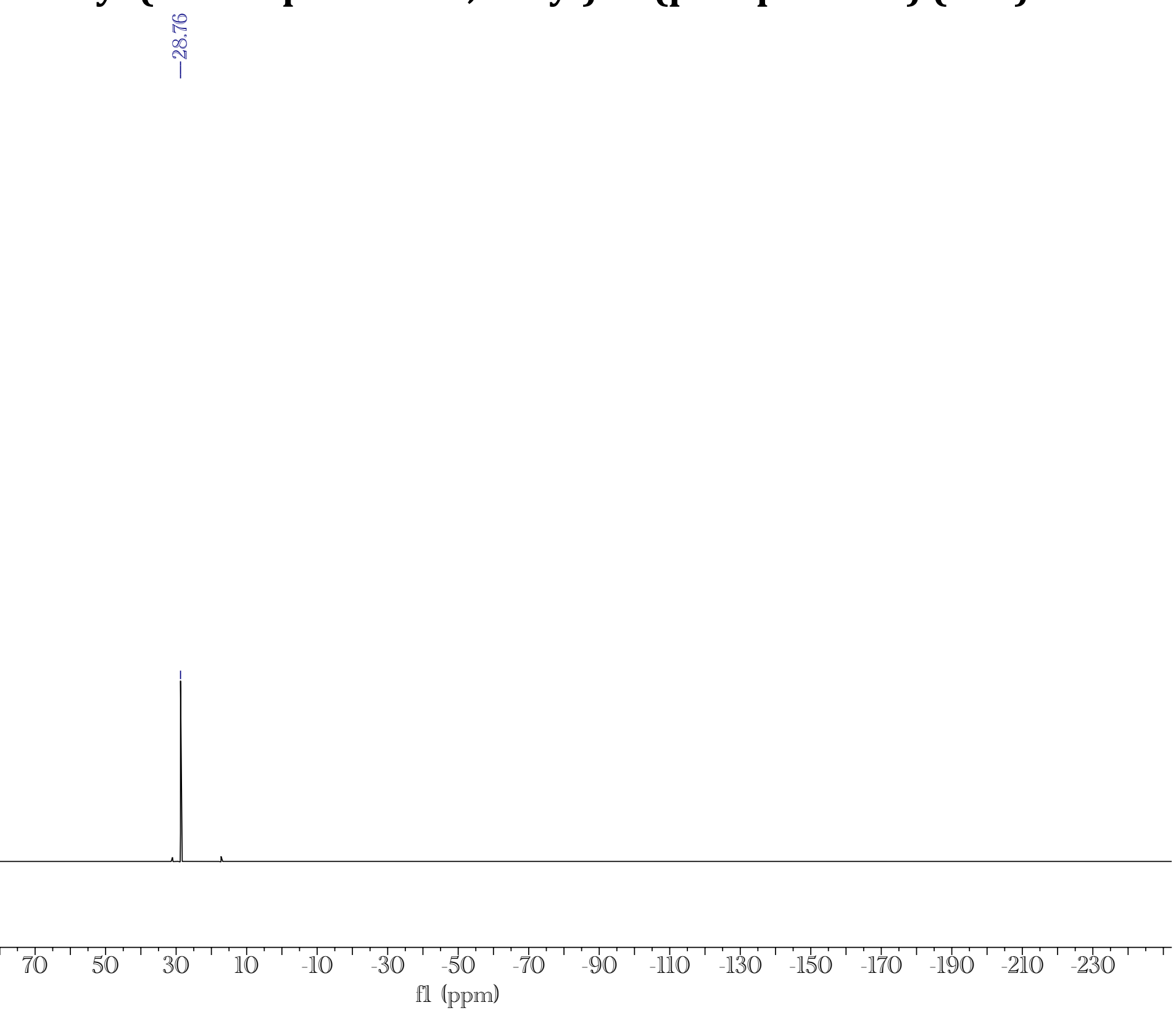

^{1}H NMR of 5-(2-nitrobutyl)benzo[d][1,3]dioxole (S30)

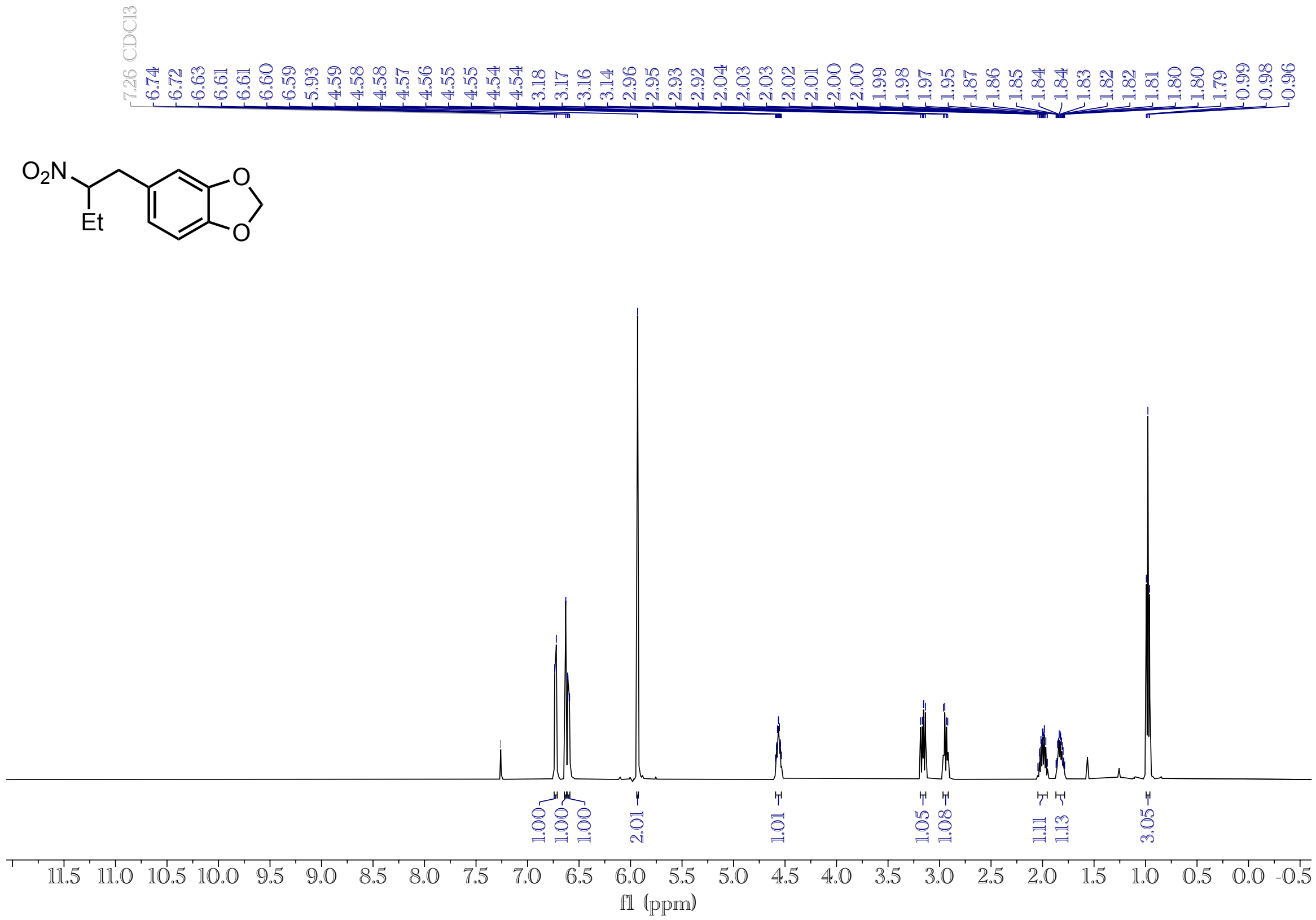

^{13}C NMR of 5-(2-nitrobutyl)benzo[d][1,3]dioxole (S30)

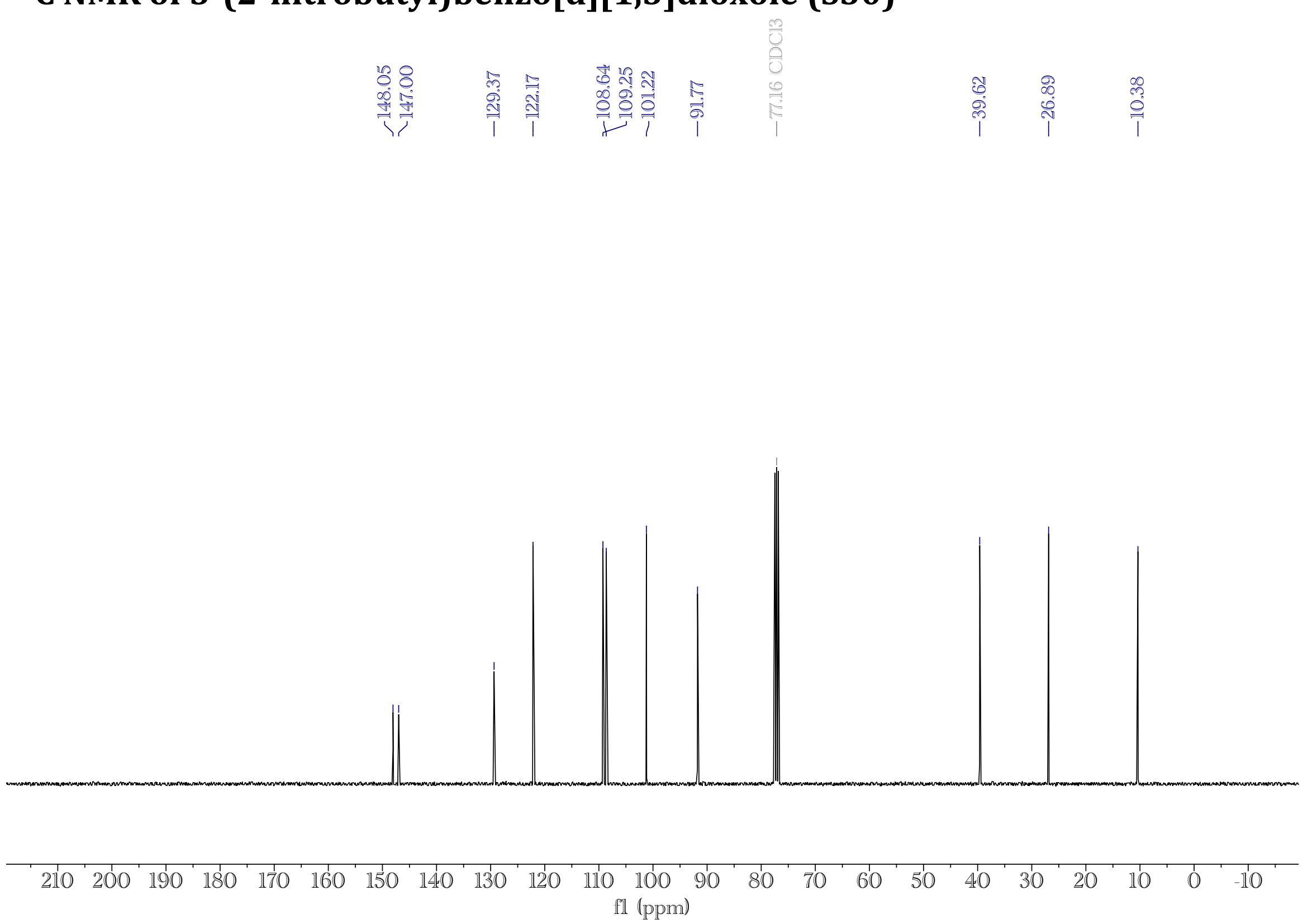

^{1}H NMR of 4-(2-nitrobutyl)benzonitrile (S31)

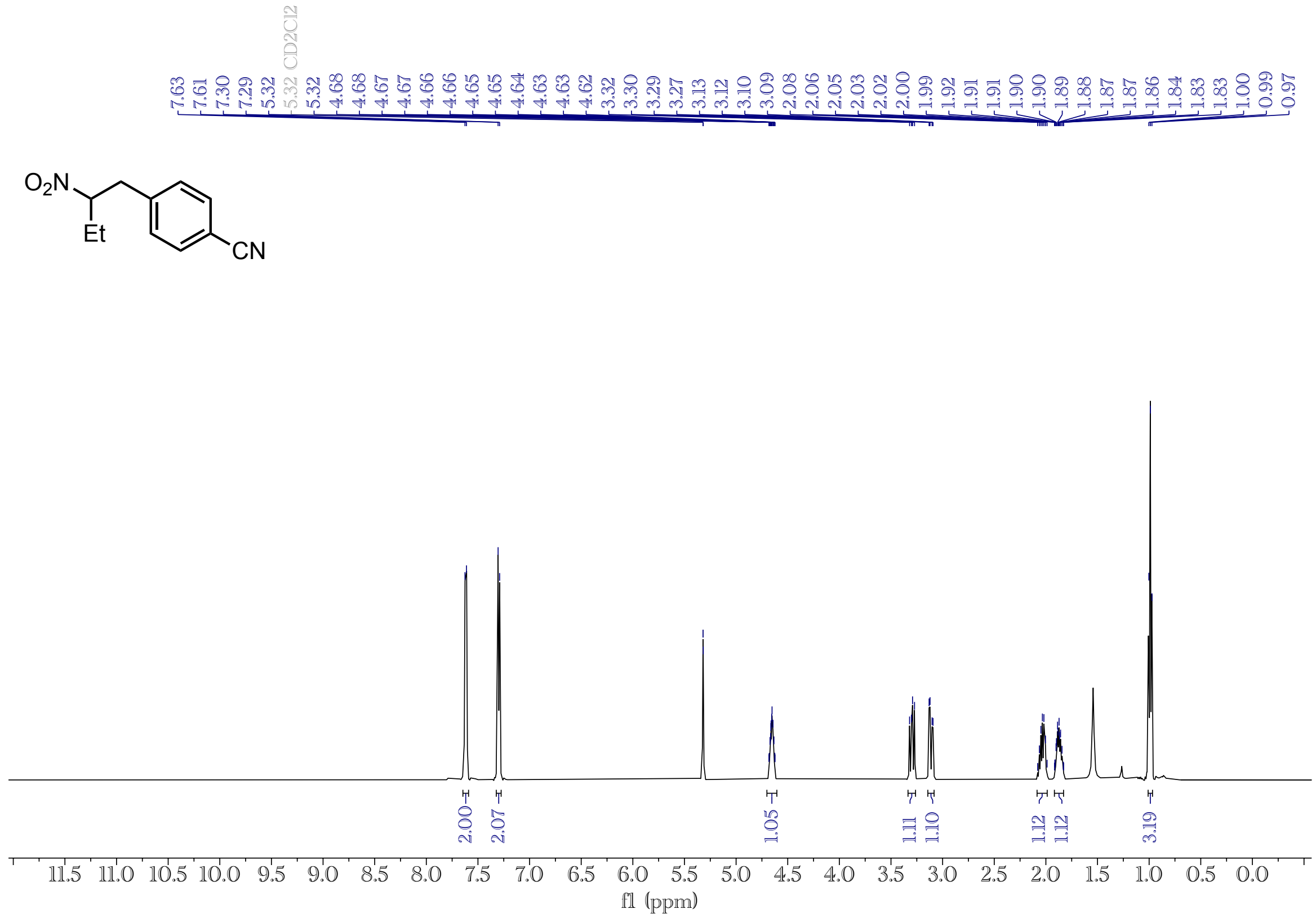

^{13}C NMR of 4-(2-nitrobutyl)benzonitrile (S31)

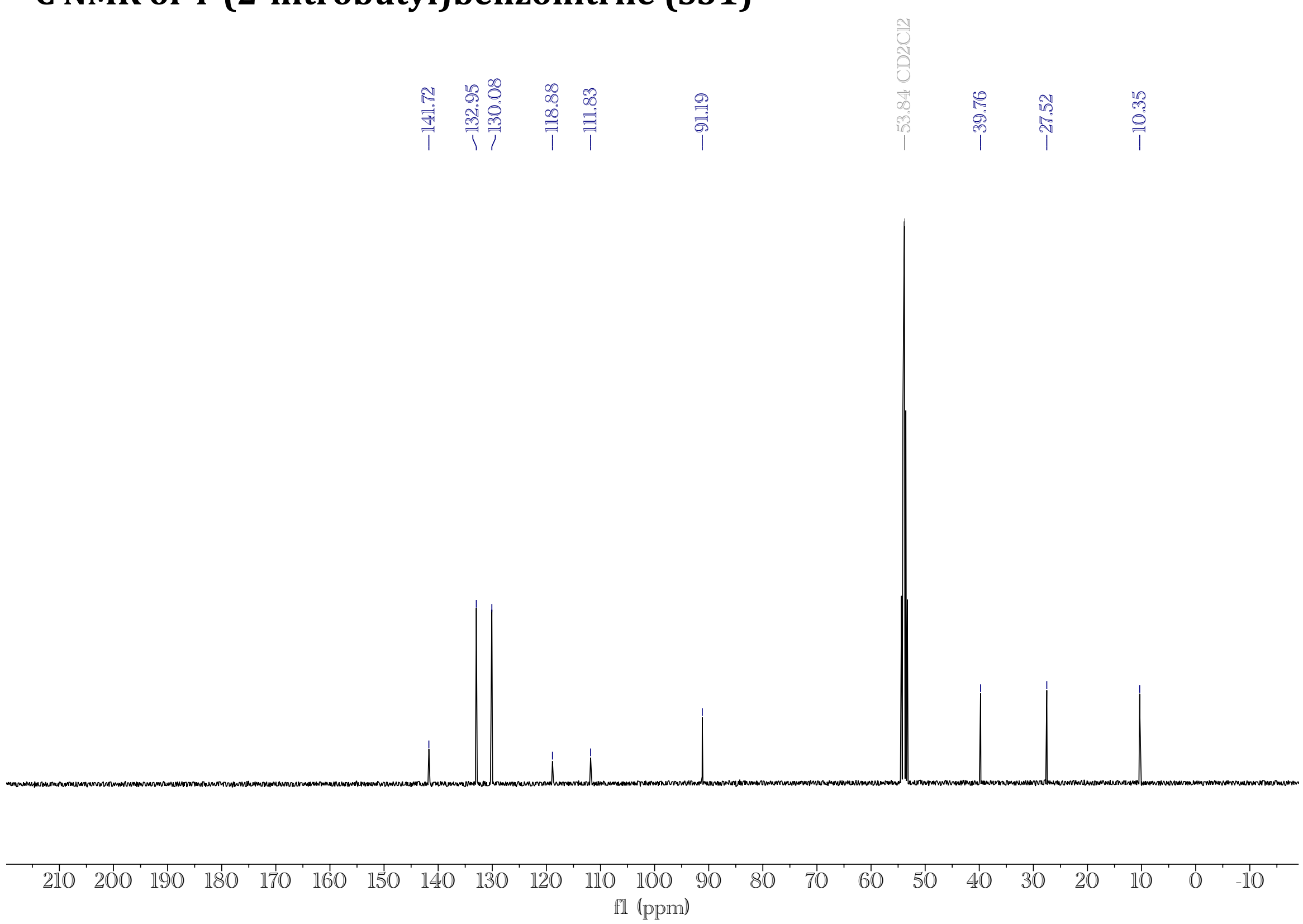

^{1}H NMR of (1-nitrohex-5-en-1-yl)benzene (S37)

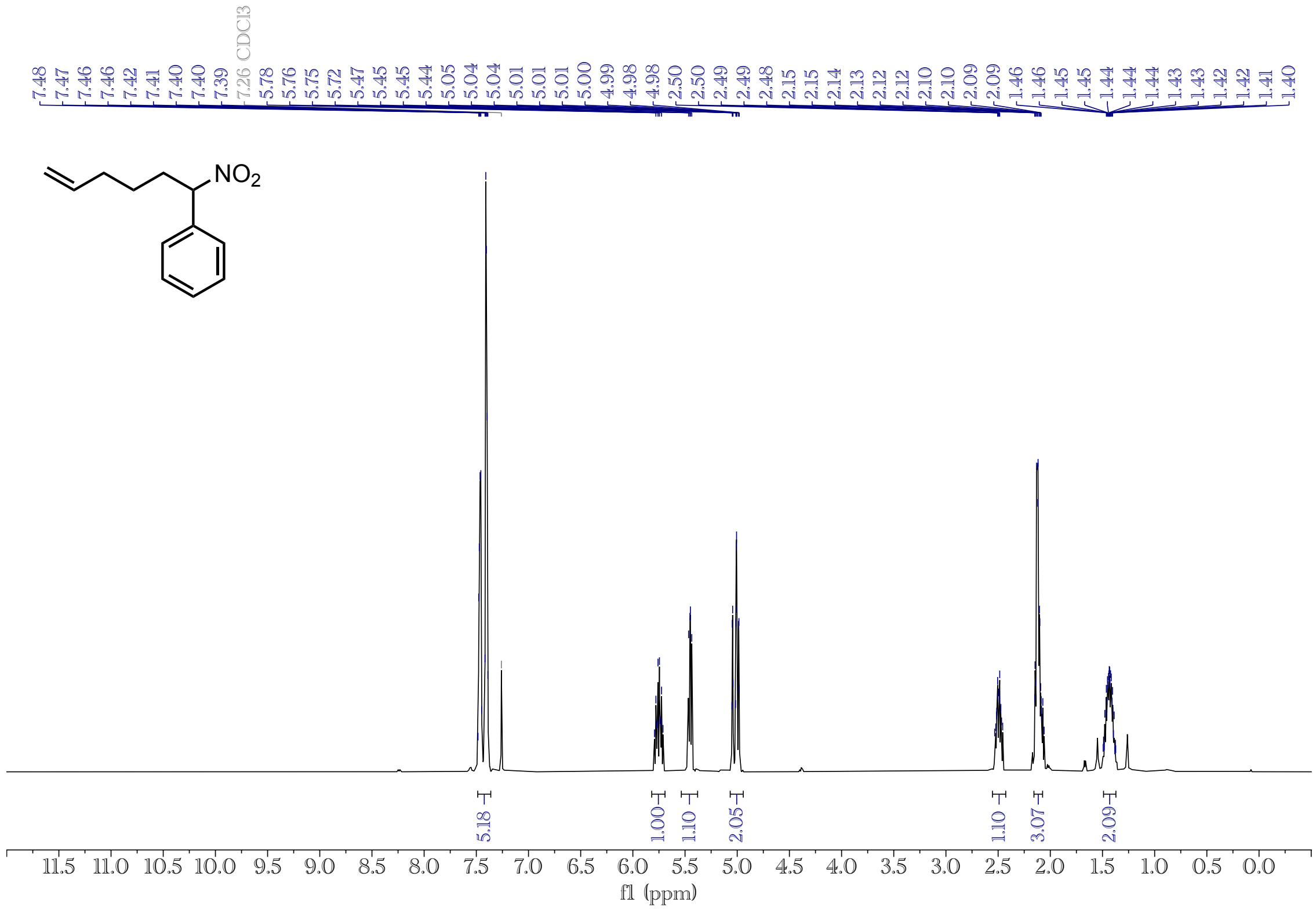

^{13}C {^{1}H} NMR of (1-nitrohex-5-en-1-yl)benzene (S37)

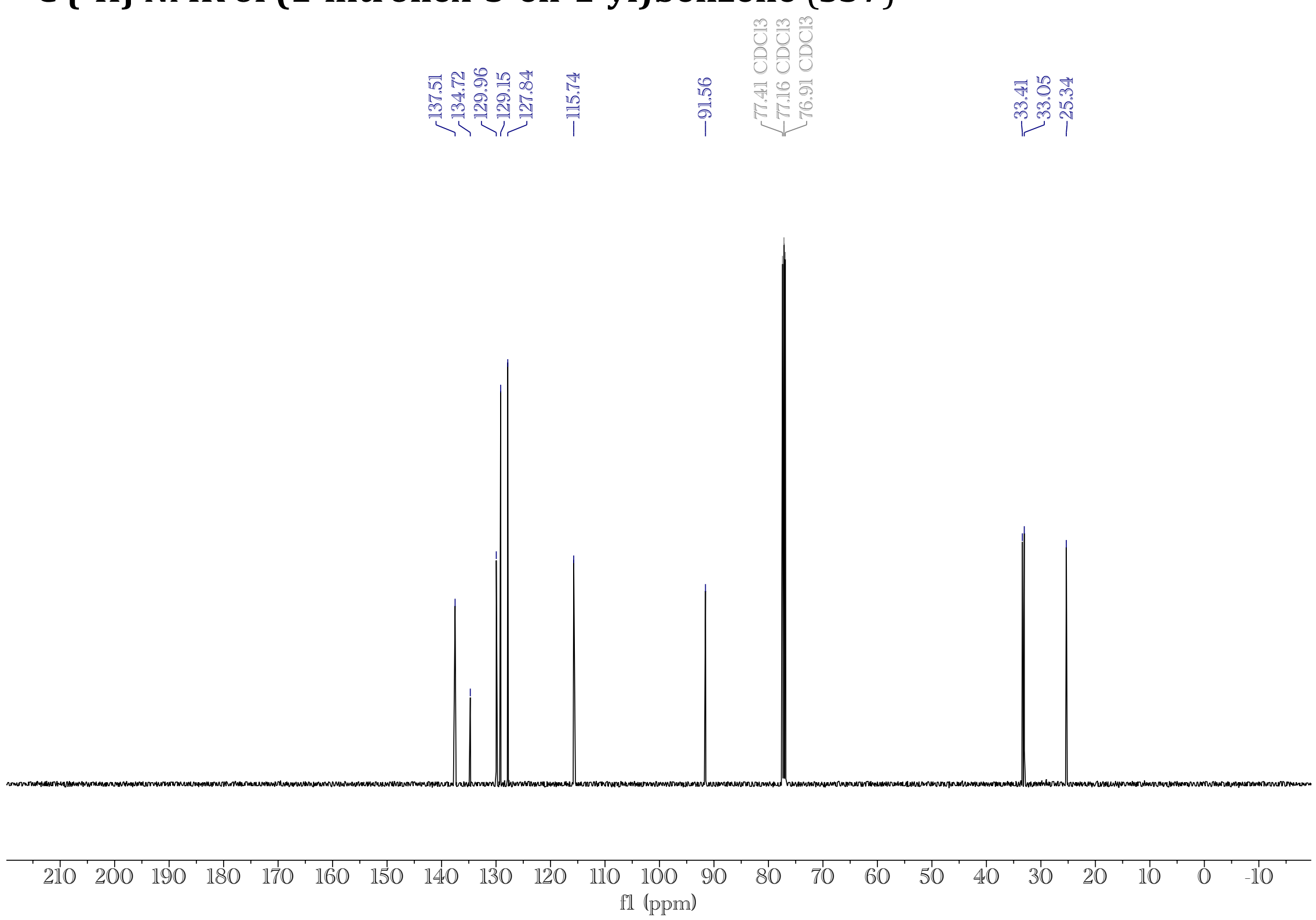

^{1}H NMR of 4-(nitromethyl)-1,2,3,6-tetrahydro-1,1'-biphenyl (S39)

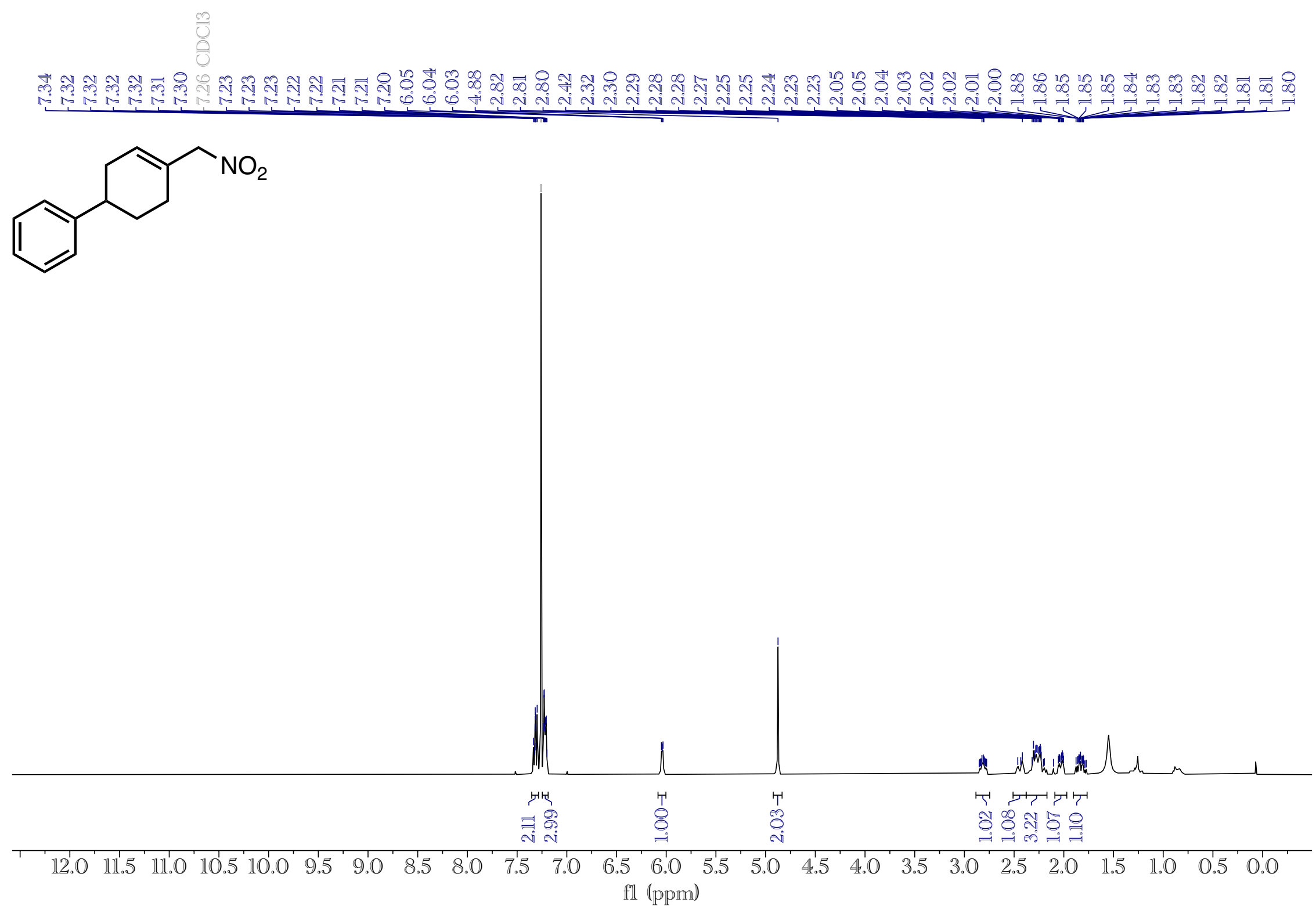

^{13}C {^{1}H} NMR of 4-(nitromethyl)-1,2,3,6-tetrahydro-1,1'-biphenyl (S39)

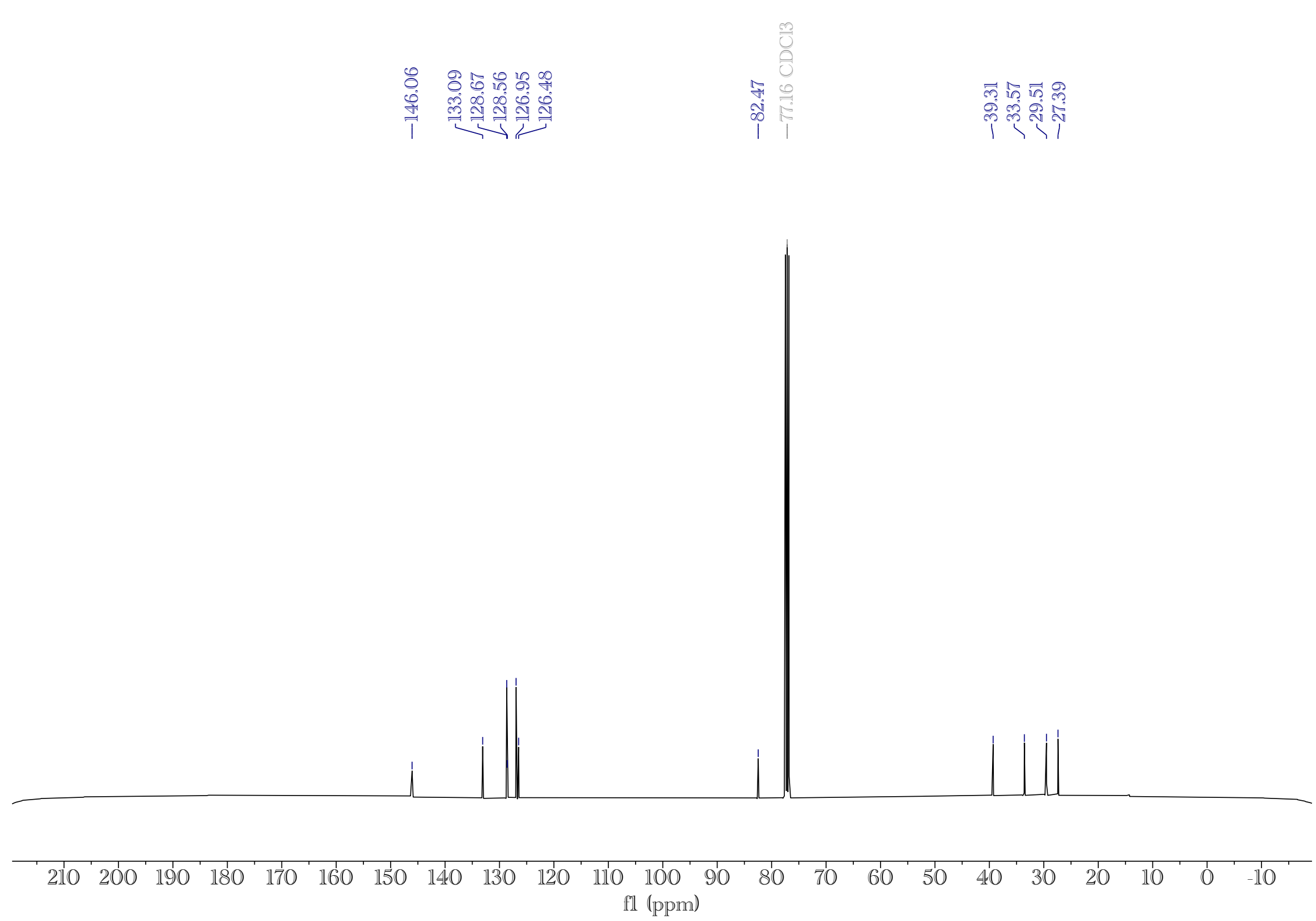

^{1}H NMR of 1-(fluoro(nitro)methyl)-4-(trifluoromethyl)benzene (S42)

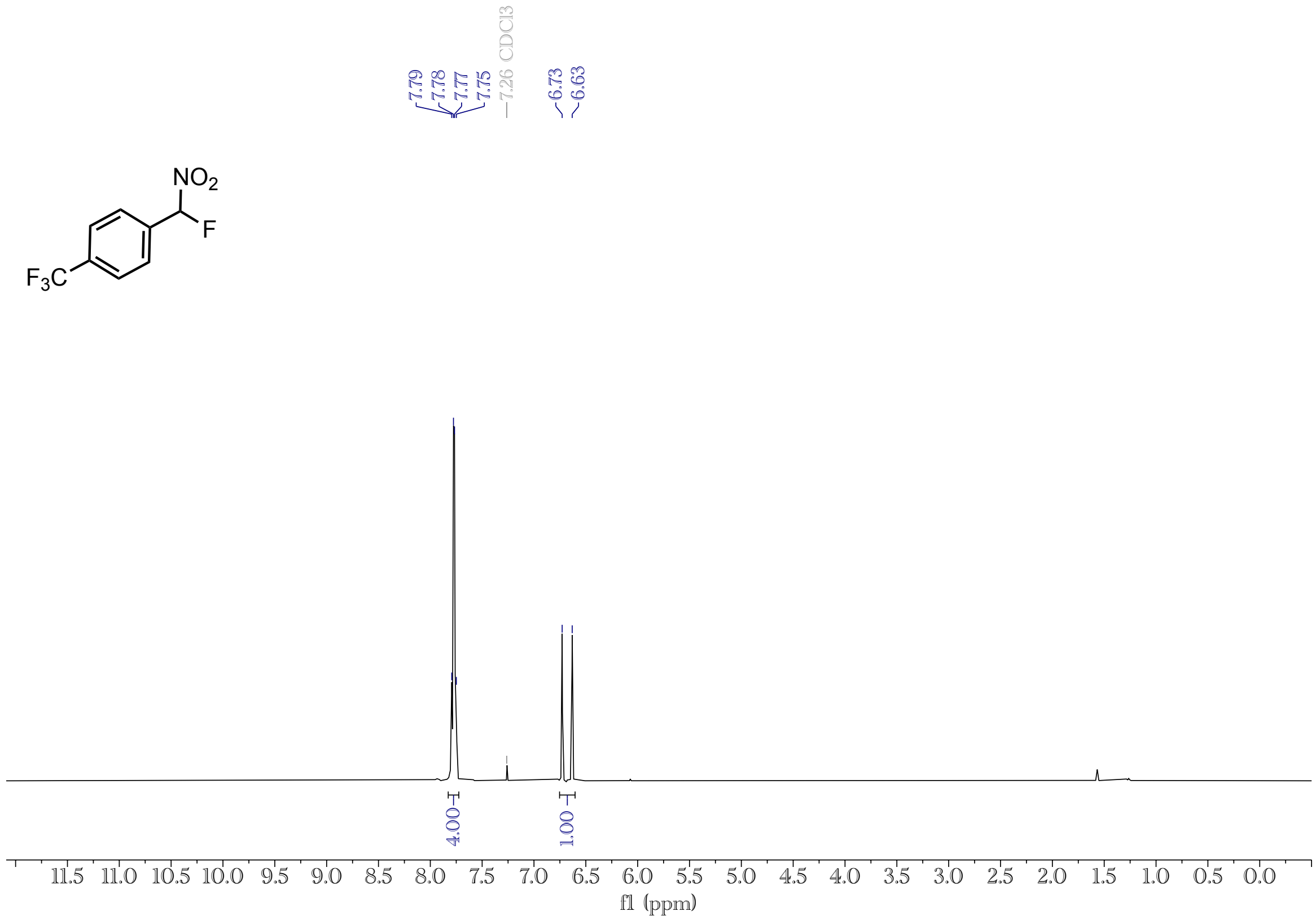

^{13}C {^{1}H} NMR of 1-(fluoro(nitro)methyl)-4-(trifluoromethyl)benzene (S42)

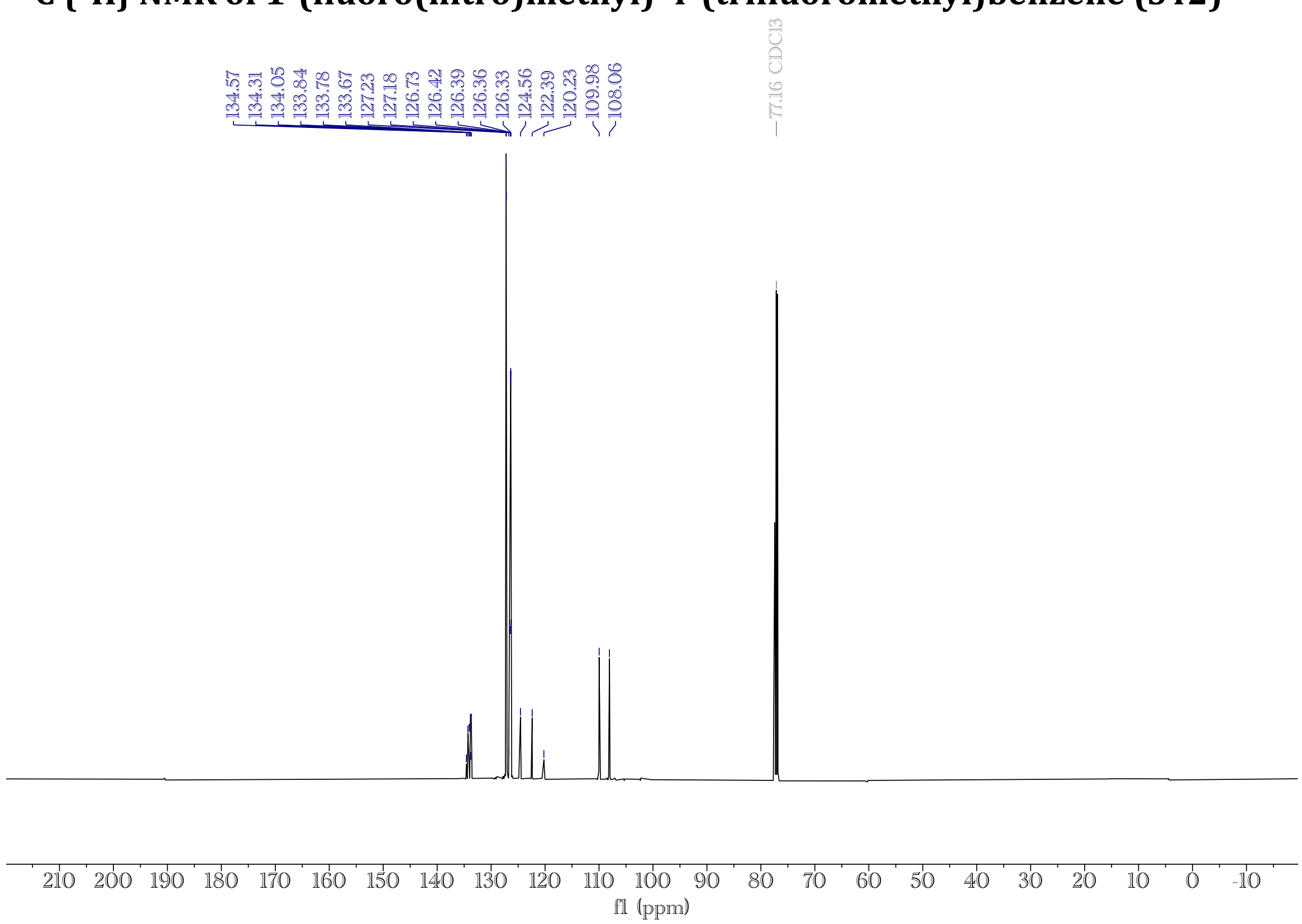

^{19}F {^{1}H} NMR of 1-(fluoro(nitro)methyl)-4-(trifluoromethyl)benzene (S42)

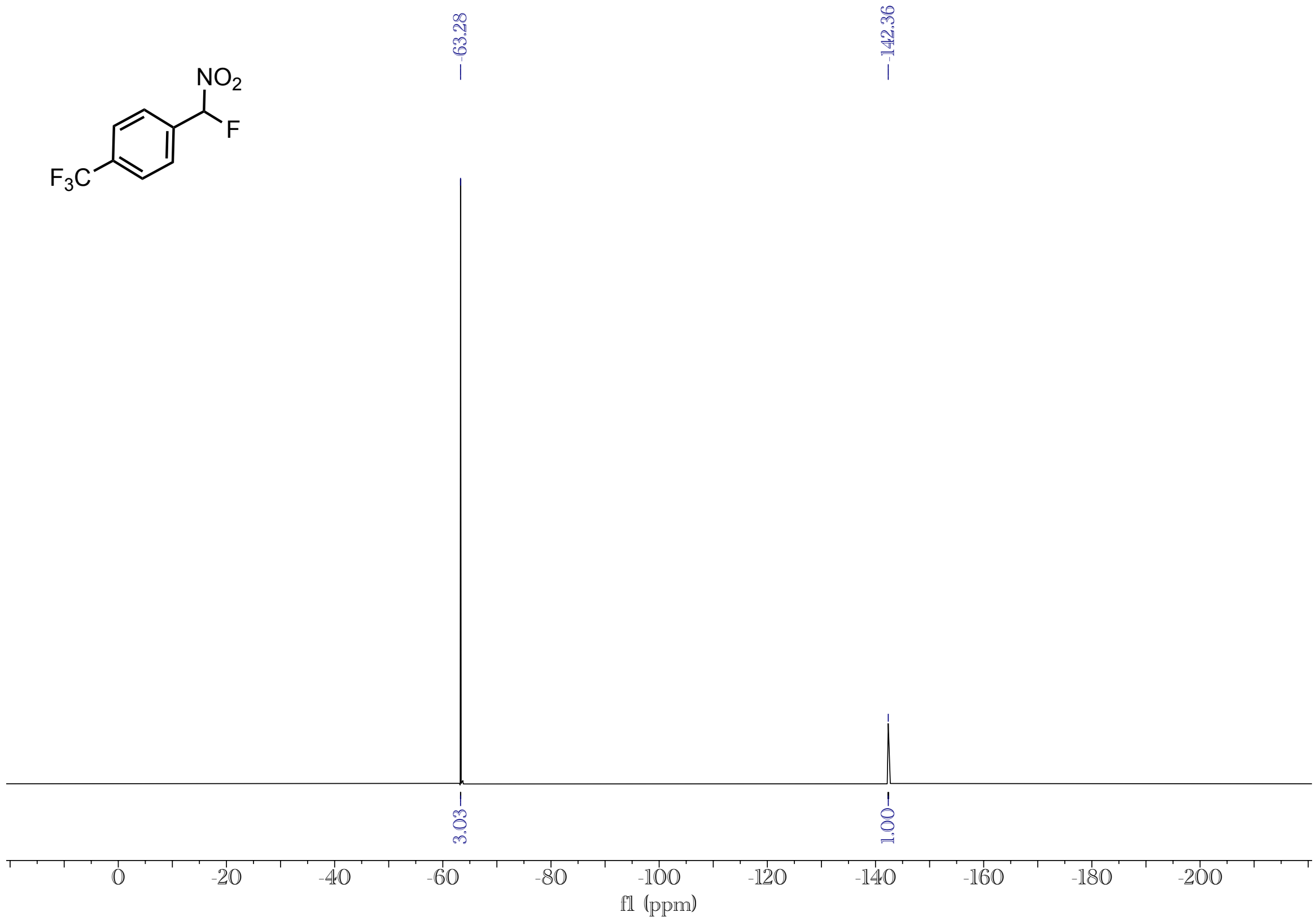

¹H NMR of 1-((3,5-dimethyl-1*H*-pyrazol-4-yl)sulfonyl)-4-nitropiperidine (*S*46)

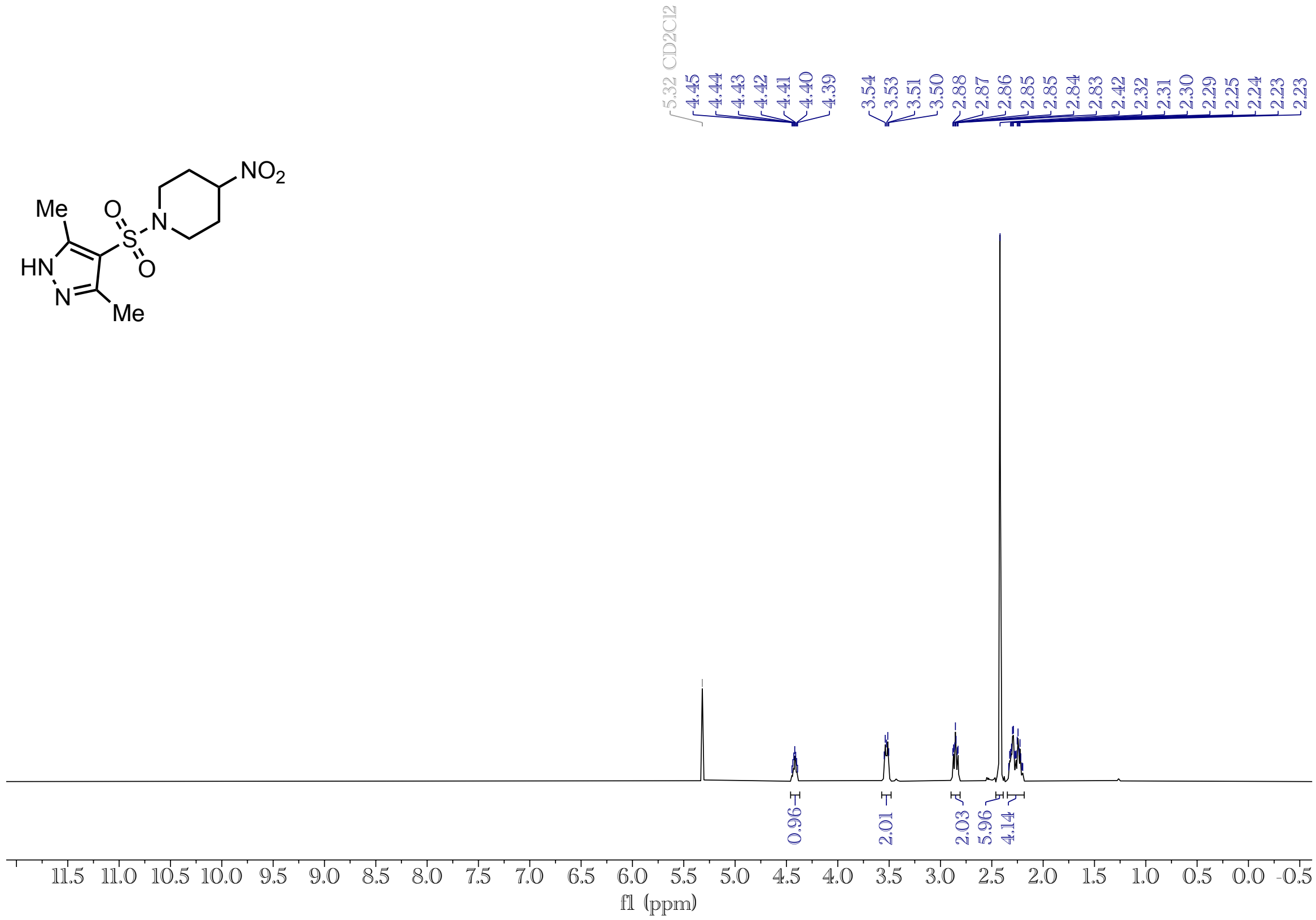

¹³C {¹H} NMR of 1-((3,5-dimethyl-1*H*-pyrazol-4-yl)sulfonyl)-4-nitropiperidine (*S*46)

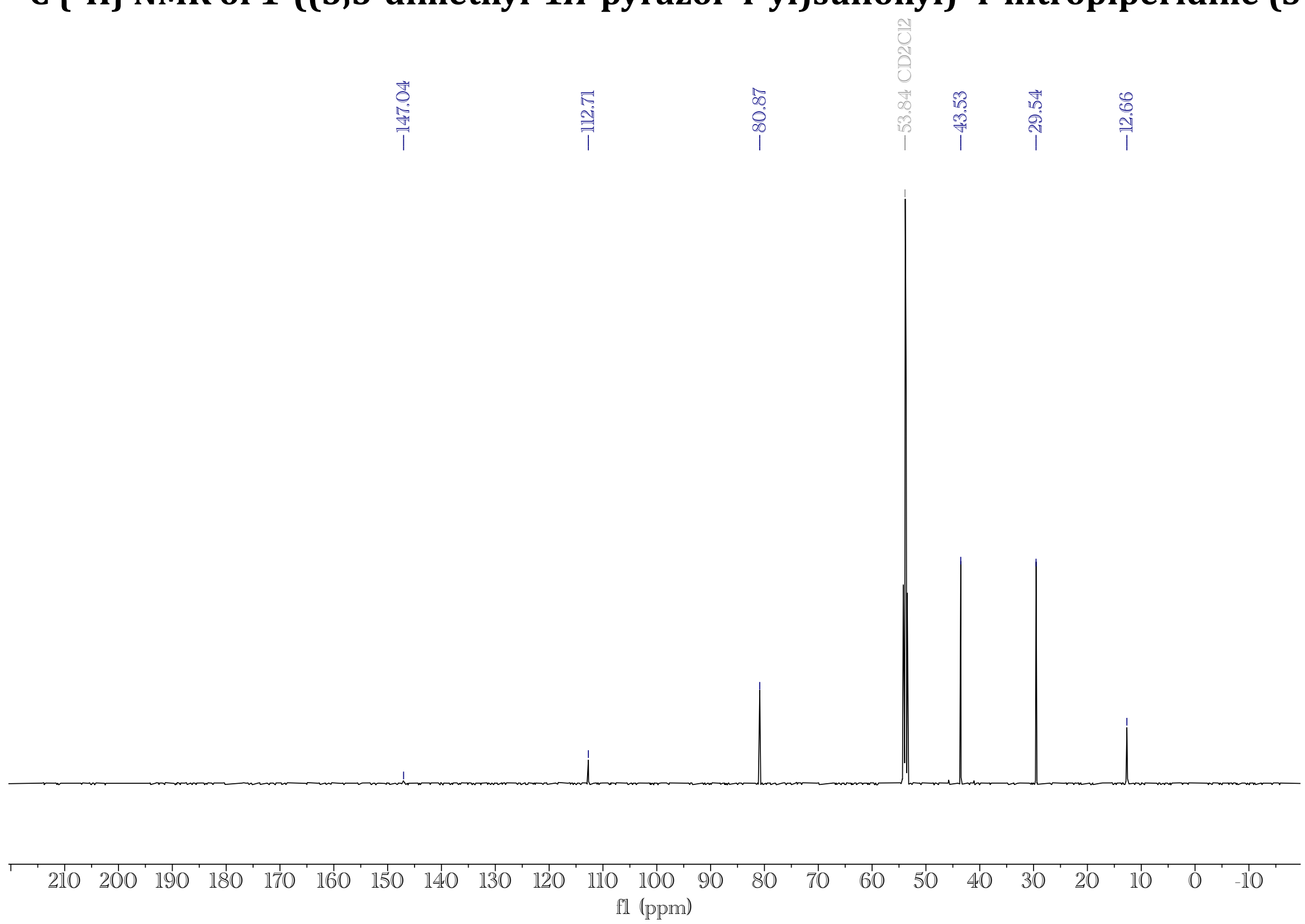

¹H NMR of 6-nitrooct-1-ene (S47)

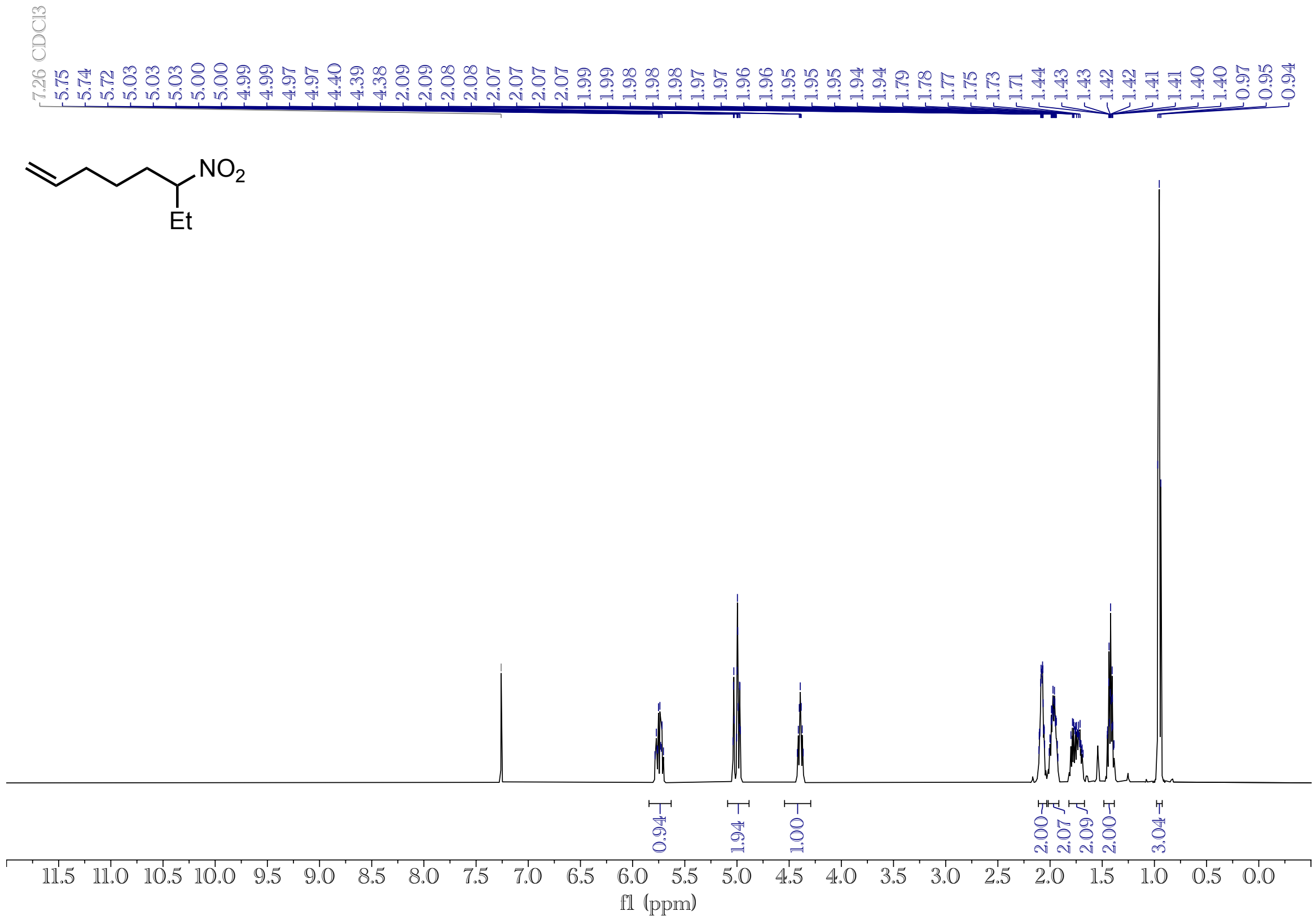

¹³C {¹H} NMR of 6-nitrooct-1-ene (S47)

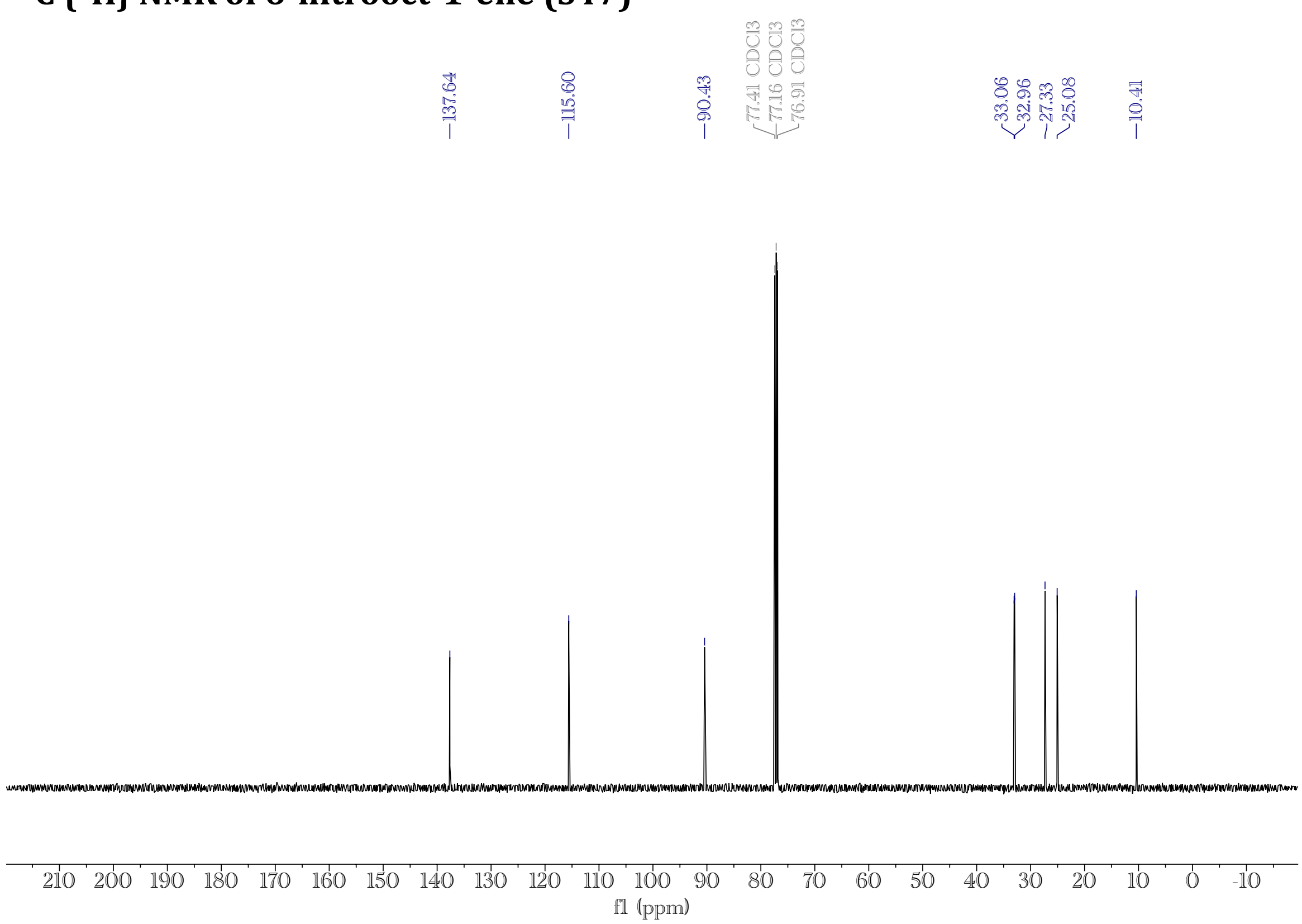

^{1}H NMR of 1,1,4,4-tetramethyl-6-(1-nitropropyl)-1,2,3,4-tetrahydronaphthalene (S48)

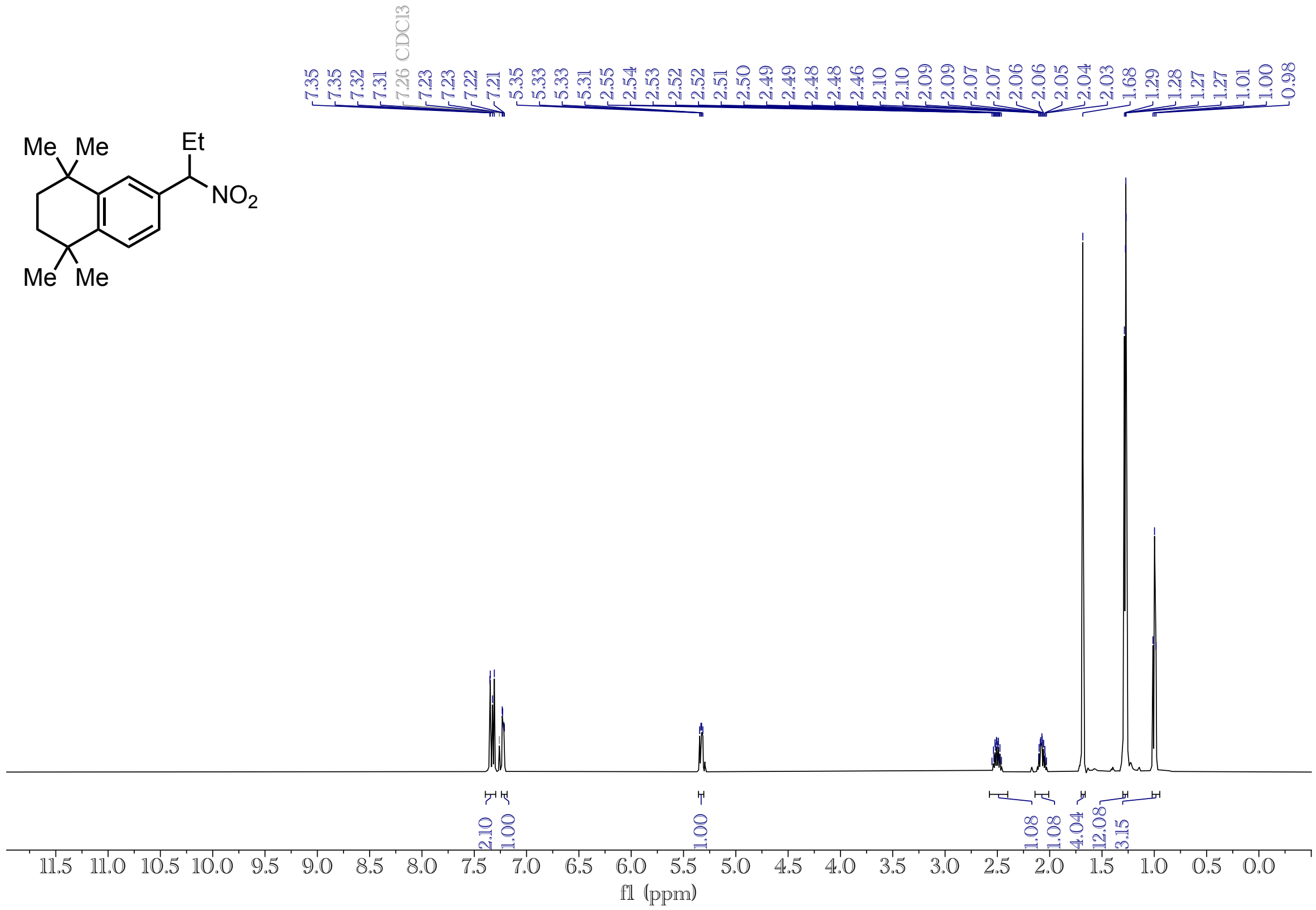

^{13}C {^{1}H} NMR of 1,1,4,4-tetramethyl-6-(1-nitropropyl)-1,2,3,4-tetrahydronaphthalene (S48)

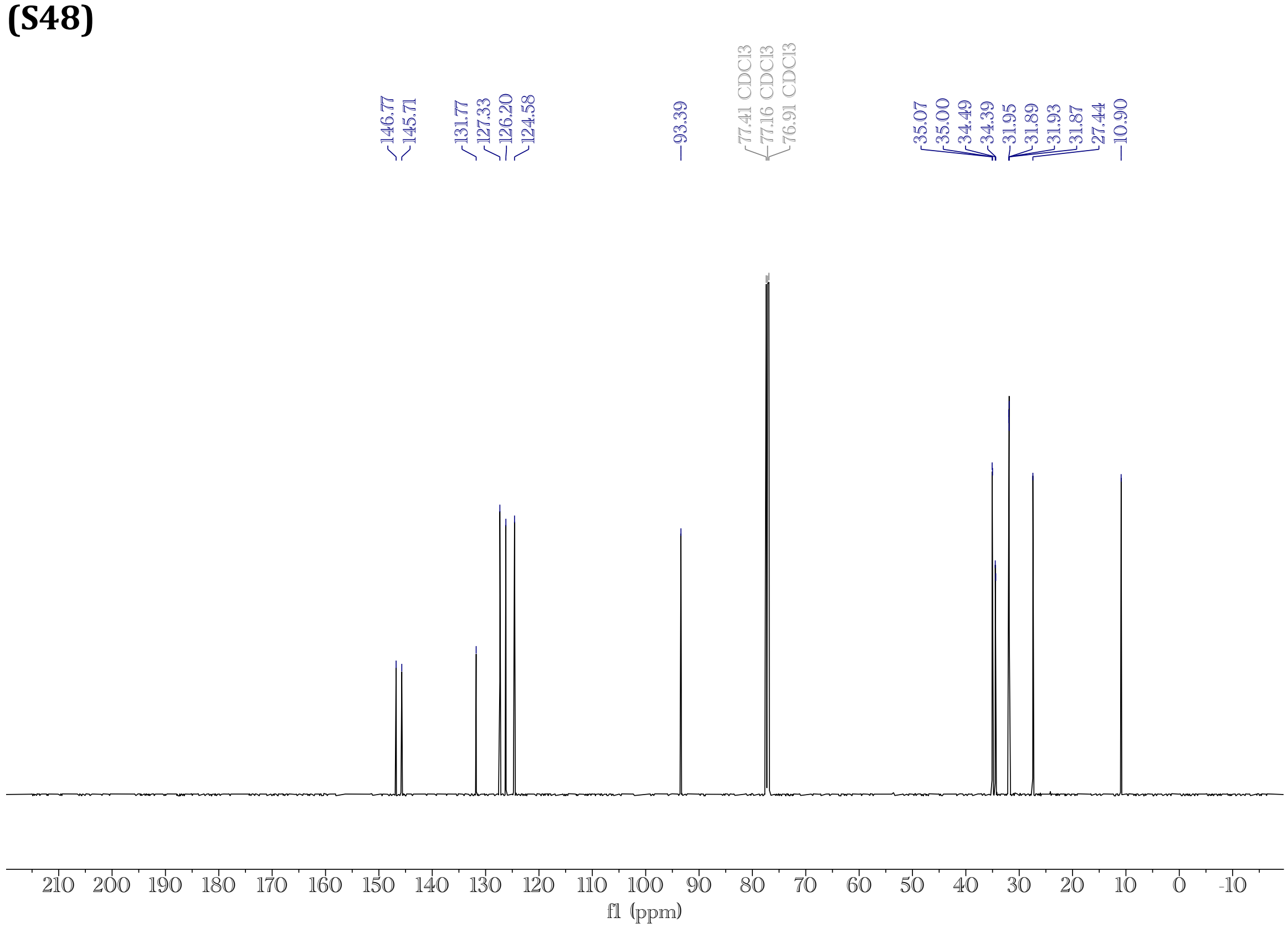

NMR Spectra of Crude Products

^{1}H NMR of crude 1-(cyclohexylidenemethyl)-3,5-bis(trifluoromethyl)benzene (P11)

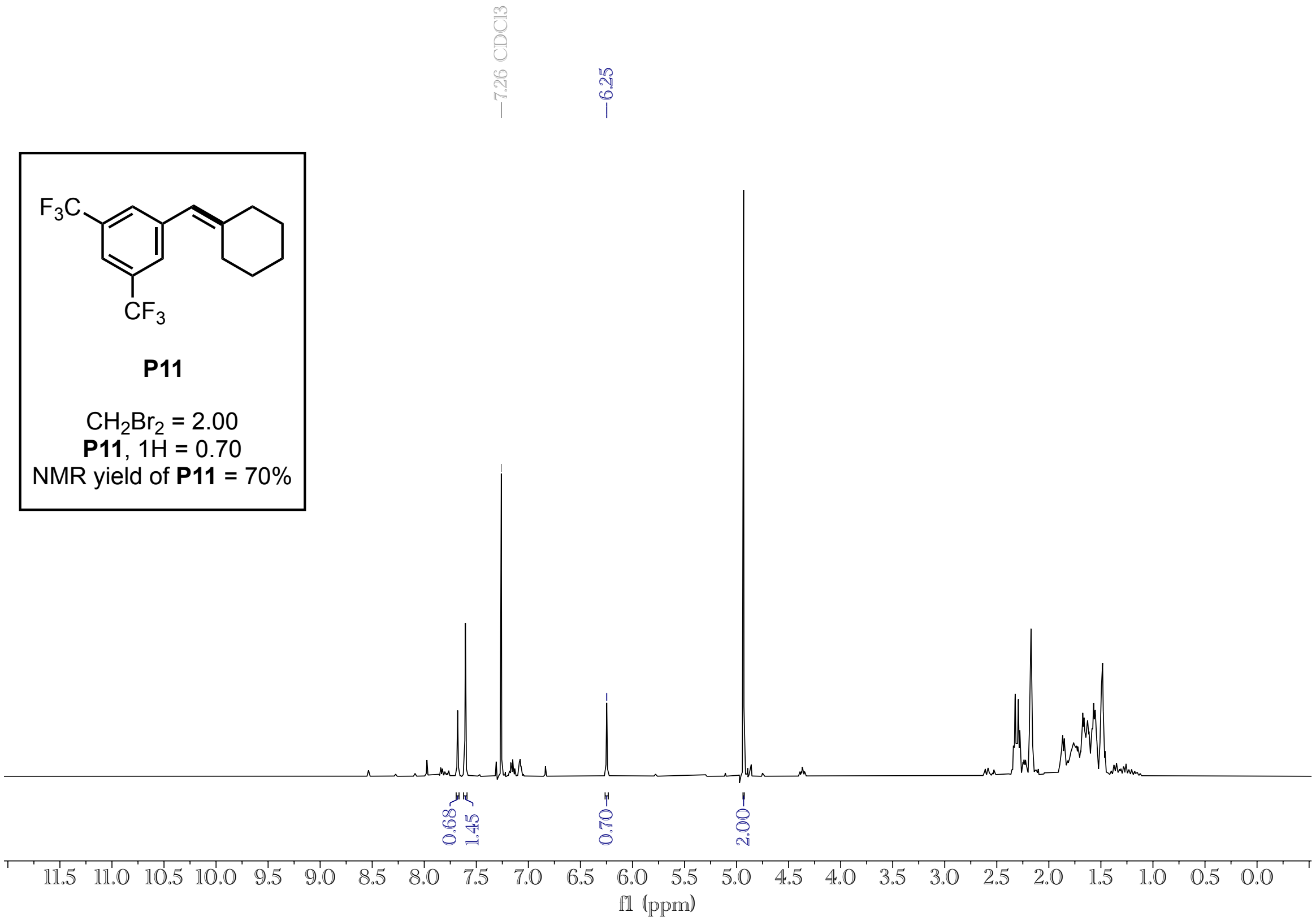

^{1}H NMR of crude 2-cinnamylfuran (P33)

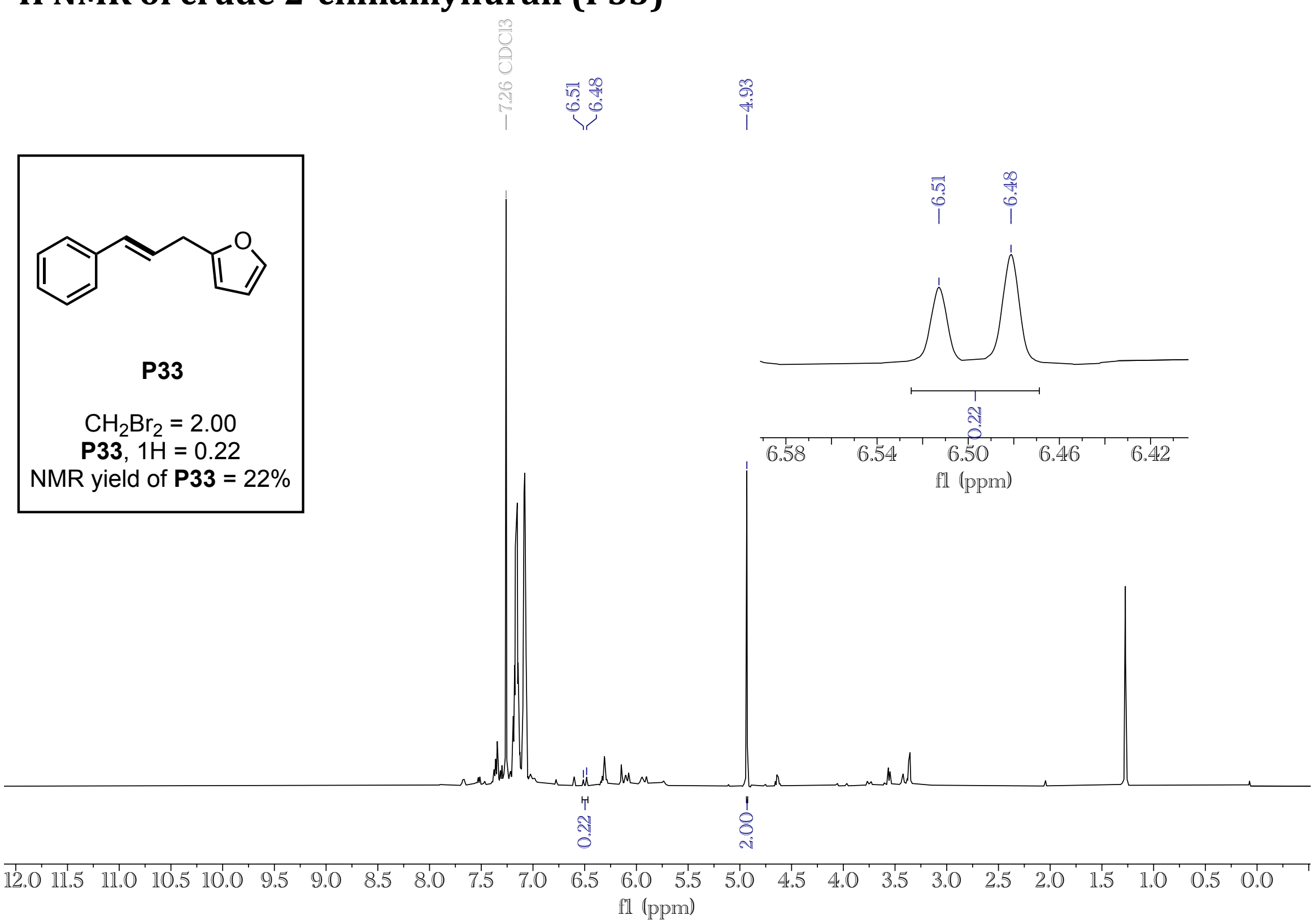

^{1}H NMR of crude 2-cinnamylthiophene (P34)

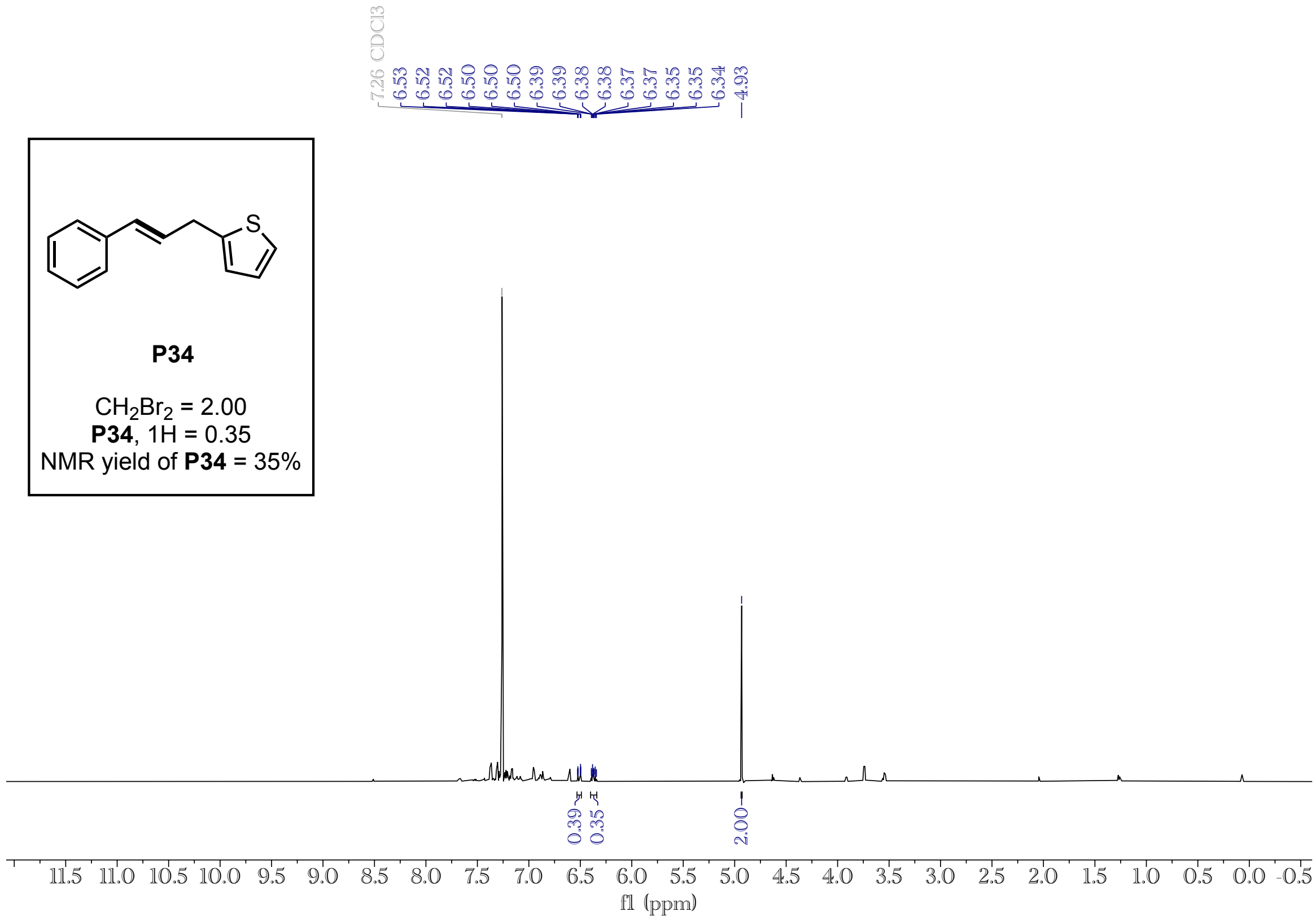

^{1}H NMR of crude 4-vinyl-1,1'-biphenyl (P35)

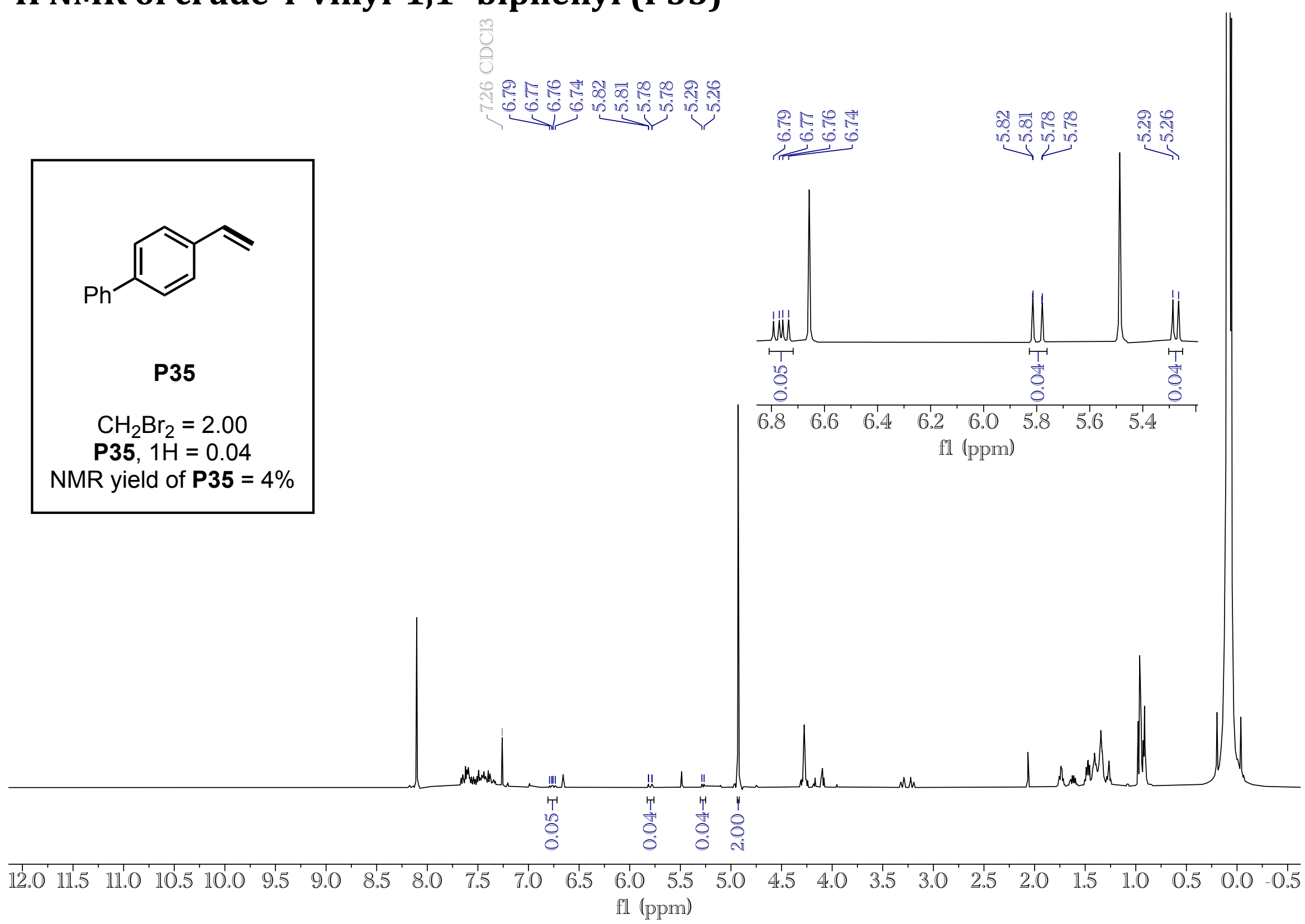

^{1}H NMR of crude 4-(cyclohexylidenemethyl)-1,2,3,6-tetrahydro-1,1'-biphenyl (P40)

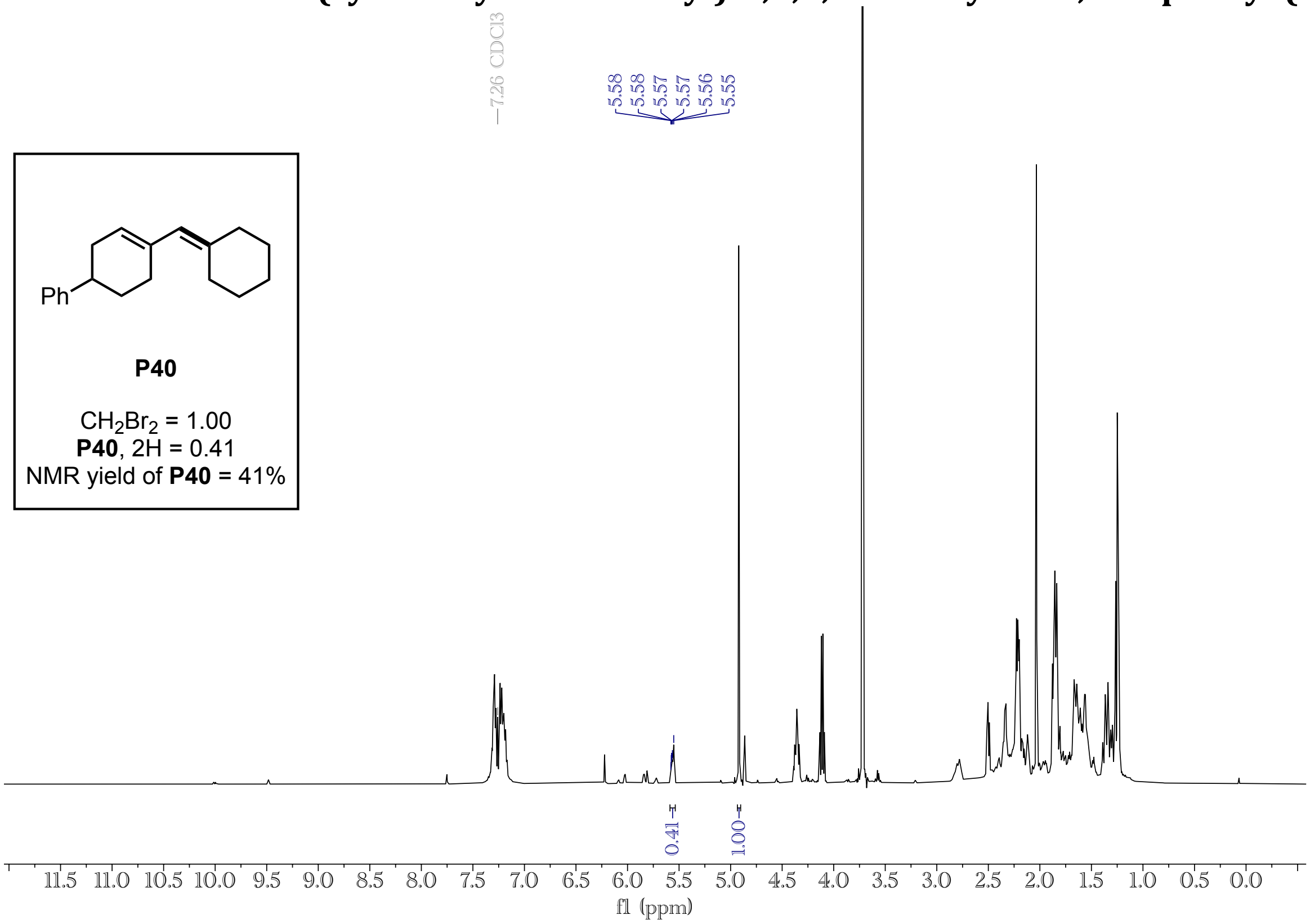

References

1. Imam, G. H. & Marples, B. A. Steroids. Part 21. Photorearrangement of steroidal nitronate salts and an N-butyl spiro-oxaziridine. *J. Chem. Soc., Perkin Trans. 1*, **1984**, 2319–2325. https://doi.org/10.1039/P19840002319

2. Trost, B. M. Decomposition of sulfur ylides. Evidence for carbene intermediates. *J. Am. Chem. Soc.* **1966**, *88*, 1587–1588. https://doi.org/10.1021/ja00959a071

3. Yamada, K., Kanekiyo, T., Tanaka, S., Naruchi, K. & Yamamoto, M. Novel intramolecular photorearrangement of alkane nitronate anions. *J. Am. Chem. Soc.* **1981**, *103*, 7003–7005. https://doi.org/10.1021/ja00413a056

4. Katritzky, A. R., Chen, J.-L., Marson, C. M., Maia, A. & Kashmiri, M. A. The non-chain radicaloid C-alkylation of nitronate anions: further evidence for the mechanism. *Tetrahedron* **1986,** *42*, 101–108. https://doi.org/10.1016/S0040-4020(01)87407-X

5. Vanelle, P., Rathelot, P., Maldonado, J. & Crozet, M. P. Consecutive $S_{RN}1$ and $E_{RC}1$ reactions in 5-nitroisoquinolines. *Tetrahedron Lett.* **1994**, *35*, 8385–8388. https://doi.org/10.1016/S0040-4039(00)74413-3

6. Fukunaga, K. & Kimura, M. Photoinduced reductive elimination of nitro groups from vicinal dinitro compounds. *Nippon Kagaku Kaishi* **1984**, *1984*, 145–151. https://doi.org/10.1246/nikkashi.1984.145

7. Norris, R. K. & Randles, D. Regiochemistry of the association step in $S_{RN}1$ reactions: kinetically controlled coupling of aci-nitronate ions and p-nitrobenzylic radicals. *J. Org. Chem.* **1982**, *47*, 1047–1051. https://doi.org/10.1021/jo00345a030

8. McMurry, J. E. & Hoz, T. Reduction of bromohydrins to olefins with low-valent titanium. *J. Org. Chem.* **1975**, *40*, 3797–3798. https://doi.org/10.1021/jo00913a047

9. Wu, Y., Kim, D. & Teets, T. S. Photophysical properties and redox potentials of photosensitizers for organic photoredox transformations. *Synlett* **2022**, *33*, 1154–1179. https://doi.org/10.1055/a-1390-9065

10. Rehm, D. & Weller, A. Kinetik und Mechanismus der Elektronübertragung bei der Fluoreszenzlöschung in Acetonitril. *Ber. Bunsenges. Phys. Chem.* **1969**, *73*, 834–839. https://doi.org/10.1002/bbpc.19690730818

11. Neese, F. Software update: the ORCA program system, version 6.0. *WIREs Comput. Mol. Sci.* **2025** *15*, e70019. https://doi.org/10.1002/wcms.70019

12. de Souza, B., Faginas-Lago, N., Sosa Carrizo, E., López, C. S. & Neese, F. GOAT: a global optimization algorithm for molecules and atomic clusters. *Angew. Chem. Int. Ed.* **2025**, *64*, e202500393. https://doi.org/10.1002/anie.202500393

13. Bannwarth, C., Ehlert, S. & Grimme, S. GFN2-xTB—an accurate and broadly parametrized self-consistent tight-binding quantum chemical method with multipole electrostatics and density-dependent dispersion contributions. *J. Chem. Theory Comput.* **2019**, *15*, 1652–1671. https://doi.org/10.1021/acs.jctc.8b01176

14. Bannwarth, E. *et al.* Extended tight-binding quantum chemistry methods. *WIREs Comput. Mol. Sci.* **2021**, *11*, e1493. https://doi.org/10.1002/wcms.1493

15. Kruse, H. & Grimme, S. A geometrical correction for the inter- and intramolecular basis set superposition error in Hartree–Fock and density functional theory calculations for large systems. *J. Chem. Phys.* **2012**, *136*, 154101. https://doi.org/10.1063/1.3700154

16. Grimme, S., Hansen, A., Ehlert, S. & Mewes, J. r^2SCAN-3c: a "Swiss army knife" composite electronic-structure method. *J. Chem. Phys.* **2021**, *154*, 064103. https://doi.org/10.1063/5.0040021

17. Adamo, C. & Barone, V. Toward reliable density functional methods without adjustable parameters: the PBE0 model. *J. Chem. Phys.* **1999**, *110*, 6158–6170. https://doi.org/10.1063/1.478522

18. Gomes, L. A. & Lopez, S. A. Benchmarking density functionals for ground- and excited-state reduction and oxidation potentials of organic photoredox catalysts. *J. Phys. Chem. A* **2025**, *129*, 6144–6154. https://doi.org/10.1021/acs.jpca.5c02406

19. Neese, F. & Olbrich, G. Efficient use of the resolution of the identity approximation in time-dependent density functional calculations with hybrid density functionals. *Chem. Phys. Lett.* **2002**, *362*, 170–178. https://doi.org/10.1016/S0009-2614(02)01053-9

20. Hanwell, M. D. *et al.* Avogadro: an advanced semantic chemical editor, visualization, and analysis platform. *J. Cheminform.* **2012**, *4*, 17. https://doi.org/10.1186/1758-2946-4-17

21. Gomes, L. A. & Lopez, S. A. Benchmarking density functionals for ground- and excited-state reduction and oxidation potentials of organic photoredox catalysts. *J. Phys. Chem. A* **2025**, *129*, 6144–6154. https://doi.org/10.1021/acs.jpca.5c02406

22. Trost, B. M., Richardson, J. & Yong, K. Palladium-catalyzed chemo- and enantioselective oxidation of allylic esters and carbonates. *J. Am. Chem. Soc.* **2006**, *128*, 2540–2541. https://doi.org/10.1021/ja057163d

23. Katritzky, A. R., Kashmiri, M. A. & Wittmann, D. K. The C-alkylation of nitroalkane anions by 1-substituted-2-tert-butyl-4-phenyl- and -2,4-diphenyl-5,6-dihydrobenzo[h]quinolinium cations. *Tetrahedron* **1984**, *40*, 1501–1510. https://doi.org/10.1016/S0040-4020(01)91797-1

24. Kornblum, N., Boyd, S. D. & Stuchal, F. W. New reaction of α-nitro esters, ketones, and nitriles and α,α-dinitro compounds. *J. Am. Chem. Soc.* **1970**, *92*, 5783–5784. https://doi.org/10.1021/ja00722a066

25. Brizzolari, A. *et al.* High-throughput Griess assay of nitrite and nitrate in plasma and red blood cells for human physiology studies under extreme conditions. *Molecules*. **2021**, *15*, 4569. https://doi.org/10.3390/molecules26154569

26. Forni, L. G., Mora-Arellano, V. O., Packer, J. E. & Willson, R. L. Aminopyrine and antipyrine free radical-cations: pulse radiolysis studies of one-electron transfer reactions. *J. Chem. Soc., Perkin Trans. 2* **1988**, 1579–1584. https://doi.org/10.1039/P29880001579

27. Eling, T. E., Mason, R. P. & Sivarajah, K. The formation of aminopyrine cation radical by the peroxidase activity of prostaglandin H synthase and subsequent reactions of the radical. *J. Biol. Chem.* **1985**, *260*, 1601–1607. https://doi.org/10.1016/S0021-9258(18)89636-3

28. Lasker, J. M., Sivarajah, K., Mason, R. P., Kalyanaraman, B., Abou-Donia, M. B. & Eling, T. E. A free radical mechanism of prostaglandin synthase-dependent aminopyrine demethylation. *J. Biol. Chem.* **1981**, *256*, 7764–7767. https://doi.org/10.1016/S0021-9258(18)43342-X

29. Zhou, S. *et al.* Switchable synthetic strategy toward trisubstituted and tetrasubstituted exocyclic alkenes. *Org. Lett.* **2018**, *20*, 6710–6714. https://doi.org/10.1021/acs.orglett.8b02801

30. Jeedimalla, N., Jacquet, C., Bahneva, D., Youte Tendoung, J.-J. & Roche, S. P. Synthesis of α-arylated cycloalkanones from congested trisubstituted spiro-epoxides: application of the House–Meinwald

rearrangement for ring expansion. *J. Org. Chem.* **2018**, *83*, 12357–12373. https://doi.org/10.1021/acs.joc.8b01448

31. Murray, P. R. D. *et al.* Intermolecular crossed [2 + 2] cycloaddition promoted by visible-light triplet photosensitization: expedient access to polysubstituted 2-oxaspiro[3.3]heptanes. *J. Am. Chem. Soc.* **2021**, *143*, 4055–4063. https://doi.org/10.1021/jacs.1c01173

32. Rivero-Crespo, M. Á., Tejeda-Serrano, M., Pérez-Sánchez, H., Cerón-Carrasco, J. P. & Leyva-Pérez, A. Intermolecular carbonyl–olefin metathesis with vinyl ethers catalyzed by homogeneous and solid acids in flow. *Angew. Chem. Int. Ed.* **2020**, *59*, 3846–3850. https://doi.org/10.1002/anie.201909597

33. Dai, W., Zhang, X., Zhang, J., Lin, Y. & Cao, S. Synthesis of exocyclic trisubstituted alkenes via nickel-catalyzed Kumada-type cross-coupling reaction of gem-difluoroalkenes with di-Grignard reagents. *Adv. Synth. Catal.* **2016**, *358*, 183–187 . https://doi.org/10.1002/adsc.201500889

34. Chen, X.-Y., Nie, X.-X., Wu, Y. & Wang, P. para-selective arylation and alkenylation of monosubstituted arenes using thianthrene S-oxide as a transient mediator. *Chem. Commun.* **2020**, *56*, 5058–5061. https://doi.org/10.1039/D0CC00641F

35. Dixon, G. J., Rodriguez, M. R., Chong, T. G., Kim, K. Y. & Downey, C. W. Synthesis of β,β-disubstituted styrenes via trimethylsilyl trifluoromethanesulfonate-promoted aldehyde–aldehyde aldol coupling–elimination. *J. Org. Chem.* **2022**, *87*, 14846–14854. https://doi.org/10.1021/acs.joc.2c01458

36. Zhang, P., Huang, D. & Newhouse, T. R. Aryl-nickel-catalyzed benzylic dehydrogenation of electron-deficient heteroarenes. *J. Am. Chem. Soc.* **2020**, *142*, 1757–1762. https://doi.org/10.1021/jacs.9b12706

37. Barluenga, J., Tomás-Gamasa, M., Moriel, P., Aznar, F. & Valdés, C. Pd-catalyzed cross-coupling reactions with carbonyls: application in a very efficient synthesis of 4-aryltetrahydropyridines. *Chem. Eur. J.* **2008**, *14*, 4792–4795. https://doi.org/10.1002/chem.200800390

38. Gardner, B. W., Chung, C. P., Pu, M. R. & Lalic, G. Synthesis of highly substituted alkenes from terminal alkynes. *J. Am. Chem. Soc.* **2025**, *147*, 33177–33184. https://doi.org/10.1021/jacs.5c11173

39. Mitchell, P. & Teall, M. Sulfonyl piperidine derivatives and their use for treating prokineticin mediated gastrointestinal disorders (WO2016075457A1, 2016).

40. Kang, T. *et al*. Multifaceted substrate–ligand interactions promote the copper-catalyzed hydroboration of benzylidenecyclobutanes and related compounds. *ACS Catal*. **2020**, *10,* 13075–13083. https://doi.org/10.1021/acscatal.0c03622

41. Nakao, Y., Imanaka, H., Sahoo, A. K., Yada, A. & Hiyama, T. Alkenyl- and aryl[2-(hydroxymethyl)phenyl]dimethylsilanes: an entry to tetraorganosilicon reagents for the silicon-based cross-coupling reaction. *J. Am. Chem. Soc.* **2005**, *127*, 6952–6953. https://doi.org/10.1021/ja051281j

42. Molander, G. A. & Argintaru, O. A. Stereospecific Ni-catalyzed cross-coupling of potassium alkenyltrifluoroborates with alkyl halides. *Org. Lett.* **2014**, *16*, 1904–1907. https://doi.org/10.1021/ol500408a

43. Li, M.-B., Wang, Y. & Tian, S.-K. Regioselective and stereospecific cross-coupling of primary allylic amines with boronic acids and boronates through palladium-catalyzed C–N bond cleavage. *Angew. Chem. Int. Ed.* **2012**, *51*, 2968–2971. https://doi.org/10.1002/anie.201109171

44. Zhang, Y., Tanabe, Y., Kuriyama, S. & Nishibayashi, Y. Photoredox- and nickel-catalyzed hydroalkylation of alkynes with 4-alkyl-1,4-dihydropyridines: ligand-controlled regioselectivity. *Chem. Eur. J.* **2022**, *28*, e202200727. https://doi.org/10.1002/chem.202200727

45. Zhou, M.-J., Zhang, L., Liu, G., Xu, C. & Huang, Z. Site-selective acceptorless dehydrogenation of aliphatics enabled by organophotoredox/cobalt dual catalysis. *Org. Lett.* **2017**, *19*, 2762–2765. https://doi.org/10.1021/jacs.1c05479

46. Chen, Z., Luo, M., Wen, Y., Luo, G. & Liu, L. Transition-metal-free semihydrogenation of diarylalkynes: highly stereoselective synthesis of trans-alkenes using Na2S·9H2O. *Org. Lett.* **2014**, *16*, 3020–3023. https://doi.org/10.1021/ol501137x

47. Mata, J. A., Falomir, E., Llusar, R. & Peris, E. Preparation, properties and coordination of new conjugated ferrocenyl-based ligands with an end-capped nitrile. *J. Organomet. Chem.* **2000**, *616*, 80–88. https://doi.org/10.1016/S0022-328X(00)00557-X

48. Dauben, W. G., Zhou, B. & Lam, J. Y. L. Photochemistry of structural analogues of previtamin D_3: generality of the wavelength-dependent triene photocyclization. *J. Org. Chem.* **1997**, *62*, 9005–9008. https://doi.org/10.1021/jo9622183

49. Liu, Q. & Burton, D. J. Stereoselective preparation of (*E*)-(1,2-difluoro-1,2-ethenediyl)bis[tributylstannane] and stereospecific synthesis of (*E*)-1,2-difluorostilbenes. *Org. Lett.* **2002**, *4*, 1483–1485. https://doi.org/10.1021/ol025686+

50. McEwen, W. E., Guzikowski, A. P. & Wolf, A. P. Addition of fluorine to tolanes. *J. Fluorine Chem.* **1984**, *25*, 169–193. https://doi.org/10.1016/S0022-1139(00)80947-1

51. Cook, A., Bezaire, M. & Newman, S. G. Nickel-catalyzed desulfonylative olefination of β-hydroxysulfones. *Org. Chem. Front.* **2023**, *10*, 1399–1404. https://doi.org/10.1039/D2QO01999J

52. Marcé, P., Lynch, J., Blacker, A. J. & Williams, J. M. J. Conversion of nitroalkanes into carboxylic acids via iodide catalysis in water. *Chem. Commun.* **2016**, *52*, 1013–1016. https://doi.org/10.1039/C5CC08681G

53. Song, K. S. *et al.* Porous polyisothiocyanurates for selective palladium recovery and heterogeneous catalysis. *Chem* **2022**, *8*, 2043–2059. https://doi.org/10.1016/j.chempr.2022.05.009

54. Jiang, M., Feng, L., Feng, J. & Jiao, P. Catalytic asymmetric cycloadditions of silyl nitronates bearing α-aryl group. *Org. Lett.* **2017**, *19*, 2210–2213. https://doi.org/10.1021/acs.orglett.7b00558

55. Alaime, T., Delots, A., Pasquinet, E., Suzenet, F. & Guillaumet, G. Scalable, easy synthesis, and efficient isolation of arylnitromethanes: a revival of the Victor Meyer reaction. *Tetrahedron* **2016**, *72*, 1337–1341. https://doi.org/10.1016/j.tet.2016.01.028

56. Matviitsuk, A. *et al.* Unanticipated silyl transfer in enantioselective α,β-unsaturated acyl ammonium catalysis using silyl nitronates. *Org. Lett.* **2020**, *22*, 335–339. https://doi.org/10.1021/acs.orglett.9b04404

57. Walvoord, R. R., Berritt, S. & Kozlowski, M. C. Palladium-catalyzed nitromethylation of aryl halides: an orthogonal formylation equivalent. *Org. Lett.* **2012**, *14*, 4086–4089. https://doi.org/10.1021/ol301713j

58. Qi, Y., Ayinla, M., Clifford, S. & Ramström, O. Spontaneous and selective macrocyclization in nitroaldol reaction systems. *J. Org. Chem.* **2023**, *89*, 1091–1098. https://doi.org/10.1021/acs.joc.3c02148

59. Ma, S. *et al.* Construction of 3-oxazolin-5-one via visible light–induced nondecarboxylative coupling and sequential reactions. *Org. Lett.* **2023**, *25*, 2098–2102. https://doi.org/10.1021/acs.orglett.3c00469

60. Vanelle, P., Madadi, N., Roubaud, C., Maldonado, J. & Crozet, M.-P. Synthèse par réaction $S_{RN}1$ de nouveaux dérivés en série imidazo[1,2-α]pyridine à potentialités pharmacologiques. *Tetrahedron* **1991**, *47*, 5173–5184. https://doi.org/10.1016/S0040-4020(01)87129-5

61. Li, G., Kanda, Y., Hong, S. Y. & Radosevich, A. T. Enabling reductive C–N cross-coupling of nitroalkanes and boronic acids by steric design of P(III)/P(V)=O catalysts. *J. Am. Chem. Soc.* **2022**, *144*, 8242–8248. https://doi.org/10.1021/jacs.2c01487

62. Crumbie, R. L., Nimitz, J. S. & Mosher, H. S. α-Nitro ketones and esters from acylimidazoles. *J. Org. Chem*. **1982**, *47*, 4040–4045. https://doi.org/10.1021/jo00142a008

63. Cai, S., Zhang, S., Zhao, Y. & Wang, D. Z. New approach to oximes through reduction of nitro compounds enabled by visible-light photoredox catalysis. *Org. Lett.* **2013**, *15*, 2660–2663. https://doi.org/10.1021/ol4009443

64. Huang, J., Li, T., Lu, X. & Ma, D. Copper-catalyzed α-arylation of nitroalkanes with (hetero)aryl bromides/iodides. *Angew. Chem. Int. Ed.* **2024**, *63*, e202315994. https://doi.org/10.1002/anie.202315994

65. Kerim, M. D., Boyode, P., Garrec, J. & El Kaïm, L. Metal-free addition of boronic acids to silylnitronates. *Synlett* **2020**, *31*, 856–860. https://doi.org/10.1055/s-0039-1690845

66. Hu, H., Huang, Y. & Guo, Y. Henry reaction of fluorinated nitro compounds. *J. Fluorine Chem.* **2012**, *133*, 108–114. https://doi.org/10.1016/j.jfluchem.2011.09.004

67. H. Fu, T. Qiao, J. M. Carceller, S. N. MacMillan, T. K. Hyster, Asymmetric C-alkylation of nitroalkanes via enzymatic photoredox catalysis. J. Am. Chem. Soc. **2023**, *145*, 787–793. https://doi.org/10.1021/jacs.2c12197

68. Zang, Z.-L. *et al.* Catalytic multisite-selective acetoxylation reactions at sp^2 vs sp^3 C–H bonds in cyclic olefins. *Org. Lett.* **2016**, *18*, 5014–5017. https://doi.org/10.1021/acs.orglett.6b02458

69. Orita, A., Yasui, Y. & Otera, J. Automated synthesis: utilization of MEDLEY in synthetic processes. *Org. Process Res.* Dev. **2000**, *5*, 337–341. https://doi.org/10.1021/op0000475
